# Lumicitabine, an orally administered nucleoside analog, in infants hospitalized with respiratory syncytial virus (RSV) infection: Safety, efficacy, and pharmacokinetic results

**DOI:** 10.1371/journal.pone.0288271

**Published:** 2023-07-19

**Authors:** Abbie Oey, Matthew McClure, Julian A. Symons, Sushmita Chanda, John Fry, Patrick F. Smith, Kathia Luciani, Michael Fayon, Kulkanya Chokephaibulkit, Rattapon Uppala, Jolanta Bernatoniene, Kenji Furuno, Thorsten Stanley, Dymphy Huntjens, James Witek, on behalf of the 503 and RSV2004 Study Groups

**Affiliations:** 1 Janssen Research & Development, LLC, South San Francisco, California, United States of America; 2 Certara Strategic Consulting, Parsippany, New Jersey, United States of America; 3 Department of Infectious Diseases Hospital de Especialidades Pediátricas Omar Torrijos Herrera, Panama City, Panama; 4 CHU de Bordeaux, Pneumologie pédiatrique, CIC 1401 (INSERM), Hôpital Pellegrin-Enfants, Bordeaux Cedex, France; 5 Department of Pediatrics, Faculty of Medicine Siriraj Hospital, Mahidol University, Bangkok, Thailand; 6 Department of Pediatrics, Khon Kaen University, Khon Kaen, Thailand; 7 Paediatric Infectious Disease and Immunology Department, Bristol Royal Hospital for Children, Bristol, United Kingdom; 8 General Pediatrics & Interdisciplinary Medicine Fukuoka Children’s Hospital, Fukuoka, Japan; 9 Department of Paediatrics, University of Otago, Wellington, New Zealand; 10 Janssen Research & Development, Beerse, Belgium; 11 Janssen Research & Development, LLC, Titusville, New Jersey, United States of America; SRM Medical College Hospital and Research Centre, INDIA

## Abstract

Respiratory syncytial virus (RSV) infection is the leading cause of infant hospitalizations and mortality. Lumicitabine, an oral nucleoside analog was studied for the treatment of RSV. The phase 1b and phase 2b studies reported here assessed the safety, pharmacokinetics, and pharmacodynamics of lumicitabine in infants/neonates hospitalized with RSV. In the phase 1b study, infants (≥1 to ≤12 months) and neonates (<28 days) received a single-ascending or multiple-ascending doses (single loading dose [LD] then 9 maintenance doses [MD] of lumicitabine, or placebo [3:1]). In the phase 2b study, infants/children (28 days to ≤36 months old) received lumicitabine 40/20 mg/kg, 60/40 mg/kg LD/MD twice-daily or placebo (1:1:1) for 5 days. Safety, pharmacokinetics, and efficacy parameters were assessed over 28 days. Lumicitabine was associated with a dose-related increase in the incidence and severity of reversible neutropenia. Plasma levels of ALS-008112, the active nucleoside analog, were dose-proportional with comparable mean exposure levels at the highest doses in both studies. There were no significant differences between the lumicitabine groups and placebo in reducing viral load, time to viral non-detectability, and symptom resolution. No emergent resistance-associated substitutions were observed at the RSV L-gene positions of interest. In summary, lumicitabine was associated with a dose-related increase in the incidence and severity of reversible neutropenia and failed to demonstrate antiviral activity in RSV-infected hospitalized infants. This contrasts with the findings of the previous RSV-A adult challenge study where significant antiviral activity was noted, without incidence of neutropenia.

Trial registration

ClinicalTrials.gov Identifier: NCT02202356 (phase 1b); NCT03333317 (phase 2b).

## Introduction

Respiratory Syncytial Virus (RSV), a ribonucleic acid (RNA) virus belonging to the *Pneumoviridae* family, has two serotypes, A and B, which co-circulate [1]. Lower respiratory tract infections due to RSV are among the leading global health ailments with an annual occurrence of 34 million cases, leading to significant morbidity and mortality [2]. According to the World Health Organization, RSV accounts for >60% of acute respiratory infections in children globally, and >80% of lower respiratory tract infections in infants annually during the peak of the viral season [3]. Children and infants with heart or lung medical conditions or adults with chronic obstructive pulmonary disease or asthma are the most vulnerable to lower respiratory tract-associated complications with increased risk of hospitalizations [4].

The management of RSV infection in children is mainly supportive; the current treatment of RSV is primarily symptomatic, including supplemental oxygen therapy, hydration, and supplemental nutrition [5]. Bronchodilators lack evidence of benefit in improving oxygen saturation or in reducing the time to resolution of illness in infants with RSV infection [5, 6]. The antiviral drug ribavirin may be considered for severe disease or for those at high risk for severe disease. However, safety concerns exist, along with a lack of convincing evidence of efficacy [7]. In addition to a lack of effective antiviral therapy, no approved vaccines are available for the prevention of RSV infection [7]. Palivizumab, an RSV-specific monoclonal antibody, is approved for prophylactic use in prematurely born infants and young children to prevent RSV infection and serious lower respiratory tract disease [3]. With these limited therapeutic options, there is an unmet need of novel agents to treat RSV infections.

Nucleoside analogs have been developed as direct-acting agents against multiple viral infections. However, these have not yet been successfully developed against acute infections caused by RSV [8]. Nucleoside analogs targeting viral polymerases have the ability to cover a broad spectrum of viral strains, while potentially increasing the barrier to drug resistance compared to other antivirals [8]. Lumicitabine (also referred to as ALS-8176, ALS-008176, or JNJ-64041575) is a 3’, 5’-bisisobutyrate nucleoside analog prodrug that is rapidly converted to the cytidine nucleoside analog ALS-008112 after oral administration and subsequently converted intracellularly to its nucleoside triphosphate (NTP), ALS-008136 [9]. This NTP is a selective inhibitor of RSV RNA polymerase activity via a classic chain termination mechanism. The active nucleoside analog of lumicitabine, ALS-008112, has been shown to impact a broad range of RSV strains of subtype A and B via inhibition of the L-protein, *in vivo* and *in vitro* [7, 9–12].

In a phase 1 study in healthy adults, lumicitabine was rapidly converted to ALS-008112 following a single oral fasted dose ranging from 40 mg to 750 mg, with measurable plasma concentrations detected within an approximate period of 15–30 minutes. Low levels of the drug plasma concentrations were detected within 6 hours post-dose. Multiple doses of lumicitabine, including two loading doses (LD; 375 mg and 750 mg) on day 1, followed by the twice-daily administration of maintenance doses (MD; 125 mg, 250 mg, and 500 mg) from days 2 to 5, resulted in no accumulation of ALS-008112 or its uridine metabolite (ALS-008144). The steady-state minimum observed concentration (C_min_) was reached by the second dose [12].

In a phase 1 RSV-A human challenge study, lumicitabine inhibited viral replication, reduced viral load, and enhanced viral clearance in healthy volunteers [11]. Patel et al. (2019) [12] developed a pharmacokinetic-pharmacodynamic (PK-PD) model that described the antiviral effect of lumicitabine in the adult challenge population during active RSV infection, with the aim of modeling efficacious plasma exposures and guiding dose selection in the adult and pediatric populations. Overall, lumicitabine was well-tolerated in both studies with no changes reported in hematological parameters.

Here, we present the results of ALS-8176-503 (Study 1; NCT02202356), a phase 1b study conducted to evaluate the safety, PK, PD (absolute viral load and effect of exposure on viral kinetics), and the emergence of viral resistance after single- and multiple-ascending doses of lumicitabine in hospitalized infants and neonates with RSV infection. Additionally, we report limited results of 64041575RSV2004 (Study 2; NCT03333317), a related phase 2b study of lumicitabine in a small number of hospitalized infants and children with RSV, terminated early by the sponsor.

## Materials and methods

### Study design

#### Single- and multiple-ascending dose study of oral lumicitabine in infants and neonates (Study 1)

Study 1 was a randomized, double-blind, placebo-controlled, 2-part, phase 1b study to assess the safety, PK and PD of single- and multiple-ascending doses (SAD/MAD) of oral lumicitabine in neonates and infants (aged 1 to 12 months) hospitalized with RSV infection. The starting dose of lumicitabine was selected based on an estimation of human efficacious doses projected to be the lowest potential therapeutic dose from the pediatric PK modeling.

In Part 1 (SAD part), infants received a single dose of 1 of 4 ascending dose levels of lumicitabine (1.37 mg/kg, 4.1 mg/kg, 12 mg/kg, and 25 mg/kg) or placebo. Within each dosing cohort, the patients were randomized to receive either lumicitabine or placebo (3:1). Except the neonate cohort, each dosing cohort was further stratified by age (≥1 to <2 months, ≥2 to <6 months and ≥6 to ≤12 months), with planned enrollment of approximately 8 patients in each stratum. An age de-escalation approach was used, such that infants between age ≥6 to ≤12 months were enrolled first, followed by those aged ≥2 to <6 months, and then ≥1 to <2 months. A sentinel group of 3 infants was enrolled initially in each age stratum and randomized (2:1) to receive either lumicitabine or placebo. An Independent Data Monitoring Committee (IDMC) reviewed the safety and PK data for the sentinel group prior to randomization (4:1 lumicitabine or placebo) of the remaining 5 infants in the age stratum. Following this assessment, enrollment in the next age stratum’s sentinel cohort(s) was initiated. Infants received the single oral dose of the study drug after randomization and were evaluated over a 7-day period. Infants discharged prior to day 7 were asked to return as an outpatient for assessment on day 7.

The Part 2 (MAD part) commenced after evaluation of the PK and safety data of Part 1 by the IDMC. Each infant received a total of 10 doses which included an initial loading dose (LD) on day 1 followed by 9 maintenance doses (MD) commencing 8 to 18 hours later, administered twice daily. This LD/MD regimen consisted of multiple-ascending doses of lumicitabine (4.1/1.37 mg/kg, 10/2 mg/kg, 30/6 mg/kg, 30/10 mg/kg, 40/20 mg/kg, and 60/40 mg/kg), or placebo. Each dosing cohort was further stratified into 3 age strata (≥1.0 to <2.0 months, ≥2 to <6 months and ≥6 to ≤12 months) and randomized (3:1) to receive either lumicitabine or placebo. The enrollment of neonates (<28 days old) occurred following the IDMC’s evaluation of the safety and PK data in 79 infants aged 1–12 months. The neonates were to be randomized (3:1) to receive either lumicitabine or placebo as 10 mg/kg LD on day 1, followed by 9 MD of 2 mg/kg administered twice daily. Only a single neonate was enrolled; subsequent enrollment of neonates was discontinued following a health authority request to generate more safety data in infants prior to the enrollment of additional neonates.

Infants were evaluated over a 28-day period from the time of randomization. All received standard supportive care during the study. Throughout both the SAD and MAD parts of the study, the IDMC reviewed unblinded PK of metabolites (ALS-008112 and ALS-008144) and the safety data on a regular basis and approved each dose escalation decision. Patient safety was monitored by regular assessment of the results of clinical laboratory tests, electrocardiograms, physical examinations, vital signs, and adverse events (AEs). In December 2016, the sponsor voluntarily halted the study due to a serious AE of severe neutropenia. The sponsor conducted a comprehensive review of the hematologic data from the lumicitabine development program. Following review by the IDMC, who determined the benefit-risk remained favorable, the sponsor resumed the study after notification/agreement of Health Authority/ethics committees.

#### Phase 2b study of lumicitabine in infants and children hospitalized with RSV (Study 2)

Study 2 was a randomized, double-blind, placebo-controlled, phase 2b study conducted in infants and children (aged 28 days to 36 months) hospitalized with RSV infection. The study consisted of the screening phase and the treatment phase. The initial LD on day 1 was followed by 9 MDs after 8-16 hours, each administered twice-daily (i.e., 5-day treatment duration). The follow-up phase ended 28 days post-randomization. Study 2 was initiated prior to the completion of Study 1. The dose regimen in Study 2 was influenced by the number of patients who were treated with the lumicitabine 40/20 mg/kg LD/ MD in Study 1, and the IDMC recommendations. In Study 2, infants/children were randomized (1:1:1) to receive either the 40/20 mg/kg LD/MD regimen, the 60/40 mg/kg LD/MD regimen, or lumicitabine-matched placebo. An unblinded IDMC planned to initially review the safety and PK data after the first 12 infants; this was to be repeated after 30 infants had completed treatment, to assess for the safe enrollment of additional patients in each arm.

In February 2018, further enrollment was voluntarily halted by the sponsor. On 17 October 2018, the study was terminated prematurely by the sponsor as a precautionary measure, to allow further evaluation and assessment of new nonclinical PK and safety findings and determine their relevance to the study in humans.

The study protocols (S1 and S2 Appendices) and amendments of both studies were reviewed and approved by the Institutional Review Board for each study site (S5 Appendix). The studies were conducted in accordance with the ethical principles communicated in the Declaration of Helsinki and in accordance with the International Conference on Harmonization (ICH) Good Clinical Practice guidelines, applicable regulatory requirements and in compliance with the protocol. Written informed consent was obtained from each infant’s parent(s)/legal guardian(s) prior to study enrollment.

### Study population and criteria

For the SAD/MAD Study 1, infants (≥1 to ≤12 months old) and neonates (<28 days old) of either sex, who were hospitalized and diagnosed RSV-positive based on a BINAX NOW RSV test, an RSV polymerase chain reaction (PCR), or any other RSV assay were eligible for enrollment. Hospitalized infants and children aged ≥28 days to ≤36 months with PCR-confirmed RSV infection were eligible for enrollment in Study 2.

For both studies, additional criteria were signs and symptoms of acute respiratory illness consistent with a viral infection including runny nose, cough, fever, or tachypnea with an onset of ≤5 days (following amendment in Study 1) from the anticipated time of randomization. Infants diagnosed with other respiratory viral or bacterial co-infections along with RSV infection were permitted study enrollment.

Infants with a history of cardiovascular, respiratory, renal, gastrointestinal, congenital, and immunodeficiency disorders were excluded. However, infants with specific comorbid conditions (prematurity at birth, bronchopulmonary dysplasia, congenital heart disease, other congenital diseases, Down syndrome, neuromuscular impairment, or cystic fibrosis) associated with severe RSV infection were permitted under Study 2. Infants who received chronic oxygen therapy or invasive endotracheal mechanical ventilation (SAD/MAD Study 1 only) or extracorporeal membrane oxygenation (Study 2 only) were excluded. Infants who received chronic immunomodulators, organic anion transporter 3 (OAT3) transporter inhibitors, recent measles, mumps, and rubella (MMR) vaccine, or prior lumicitabine or prescription medications intended to prevent or treat RSV infection itself (e.g., ribavirin, immunoglobulin, palivizumab) were also excluded.

### Study endpoints

The primary endpoint of the SAD/MAD Study 1 was to evaluate the safety and tolerability of single and multiple doses of lumicitabine as determined by AEs, physical examinations, vital signs, 12-lead electrocardiograms, and clinical laboratory results, including serum chemistry and hematological findings. In Study 2, the primary endpoint was RSV ribonucleic acid (RNA) log_10_ viral load AUC with safety assessments similar to those collected in Study 1 evaluated as a secondary endpoint.

Other secondary endpoints included the evaluation of PK parameters of lumicitabine metabolites (ALS-008112, ALS-008144) in blood. PK assessments included the maximum observed concentration (C_max_) and AUC_0-24h_ based on population PK modeling. Additionally, viral RNA concentrations in nasal swabs or aspirates as measured by quantitative reverse transcriptase PCR (qRT-PCR), changes in the RSV polymerase, and the emergence of resistant strains of RSV were evaluated. In Study 2, the time to resolution of RSV signs or symptoms, such as runny nose, wheeze, cough, or tachypnea was evaluated by the clinician and caregiver using an electronic clinical outcome assessment (eCOA) tool.

### Study assessments

Clinical laboratory variables, including hematology and serum biochemistry, were assessed at screening and periodically during the study as indicated in the protocol. AEs, including pre-treatment events, were recorded from the time of consent through the completion visit. AEs were reported using Medical Dictionary for Regulatory Activities (MedDRA) Version 16.1 or higher. Severity was graded using either the Division of Acquired Immunodeficiency Syndrome Grading Version 1.0 (Study 1) or the Division of Microbiology and Infectious Diseases toxicity scale (Study 2).

Nasal aspirates (except for the 40/20 mg/kg and 60/40 mg/kg groups in Study 1) or mid-turbinate nasal swab specimens (used for the 40/20 mg/kg and 60/40 mg/kg groups in Study 1 and Study 2) were collected at the baseline, during treatment, and post-treatment through day 7 (Study 1 SAD only) or day 28. The RSV RNA viral load in the nasal specimens was determined through day 28 using qRT-PCR assays performed at the University of Tennessee Children’s Foundation Research Institute for Study 1 (reported in plaque-forming-unit equivalents per milliliter [PFUe/mL]) or the DDL Diagnostic Laboratory (Rijswijk, The Netherlands) for Study 2 (RSV viral load lower limit of quantification (LLOQ) = 3.00 log_10_ copies/mL for RSV-A, LLOQ = 2.40 log_10_ copies/mL for RSV-B; limit of detection (LOD) = 2.75 log_10_ copies/mL for RSV-A, LOD = 1.90 log_10_ copies/mL for RSV-B. Local RSV diagnostic results were confirmed from the baseline sample by qualitative (Study 1; GenMark Respiratory Viral Panel) or quantitative (Study 2; DDL RSV viral load) PCR performed at the central laboratory. A multiplex PCR was performed at the baseline to determine the presence of other viruses or bacteria (Tables 1 and 2 in S4 Appendix).

Post-dose whole blood samples were collected from infants in all dosing cohorts in both studies at specified time points (twice in the first 24 hours’ post-dose, and a random sample at any time on the last day of dosing for multiple dose cohorts) for determining PK parameters. Whole blood concentrations of ALS-008112 and ALS-008144 and other metabolites were determined by a liquid chromatography tandem mass spectrometry (LC-MS/MS) method. The population PK model was developed to derive PK parameters following a single dose or at steady-state.

Next-generation sequencing (NGS; using 1% read-frequency cut-off) of the RSV L-gene was performed at DDL Diagnostic Laboratory on the baseline and post-baseline samples of patients in Study 1 (MAD) and Study 2. Post-baseline samples were selected from the last evaluable on-treatment time point with the RSV RNA levels above the sequencing limit. Sequencing analyses focused on the RSV L-amino acid positions 628, 789, 795, and 796, at which resistance-associated substitutions were identified in *in vitro* selection experiments with ALS-008112 [13]. An emerging amino acid substitution was defined as a substitution that was absent, i.e., with an NGS read frequency <3% at baseline, but present with an NGS read frequency ≥15% at a later post-baseline time point.

### Statistical analysis

In Study 1, all statistical analyses were performed using SAS Version 9.4 (SAS Institute, Inc., Cary, NC, USA). Based on clinical experience, in Study 1, a sample size of up to 260 patients was to be enrolled to enable the assessment of safety and PK parameters. In Study 2, a sample size of 180 patients was to be randomized to offer approximately 97% power to detect a positive dose-response relationship. However, considering the early termination of the study, statistical analysis was not performed as planned. Therefore, the data are presented descriptively.

All analyses were presented by dose groups and the combined age strata. In the final analysis, placebo data from the dose groups and/or age strata (being summarized) were pooled to form a larger comparative group. Regarding the efficacy analyses, the population was based on the intent-to-treat infected (ITT-i) analysis set that included all enrolled patients who had received at least one dose of the study drug, and were centrally confirmed PCR positive for RSV RNA. The mean RSV RNA log_10_ viral load values over time were analyzed using a restricted maximum likelihood-based repeated measures approach.

The time to non-detectability was assessed using the Kaplan-Meier analysis. Those patients who did not reach an undetectable viral load at the last observable assessment were censored. Kaplan-Meier plots were provided by the treatment group and the dose cohort within the lumicitabine group.

## Results

### Patient disposition

**Fig 1:**
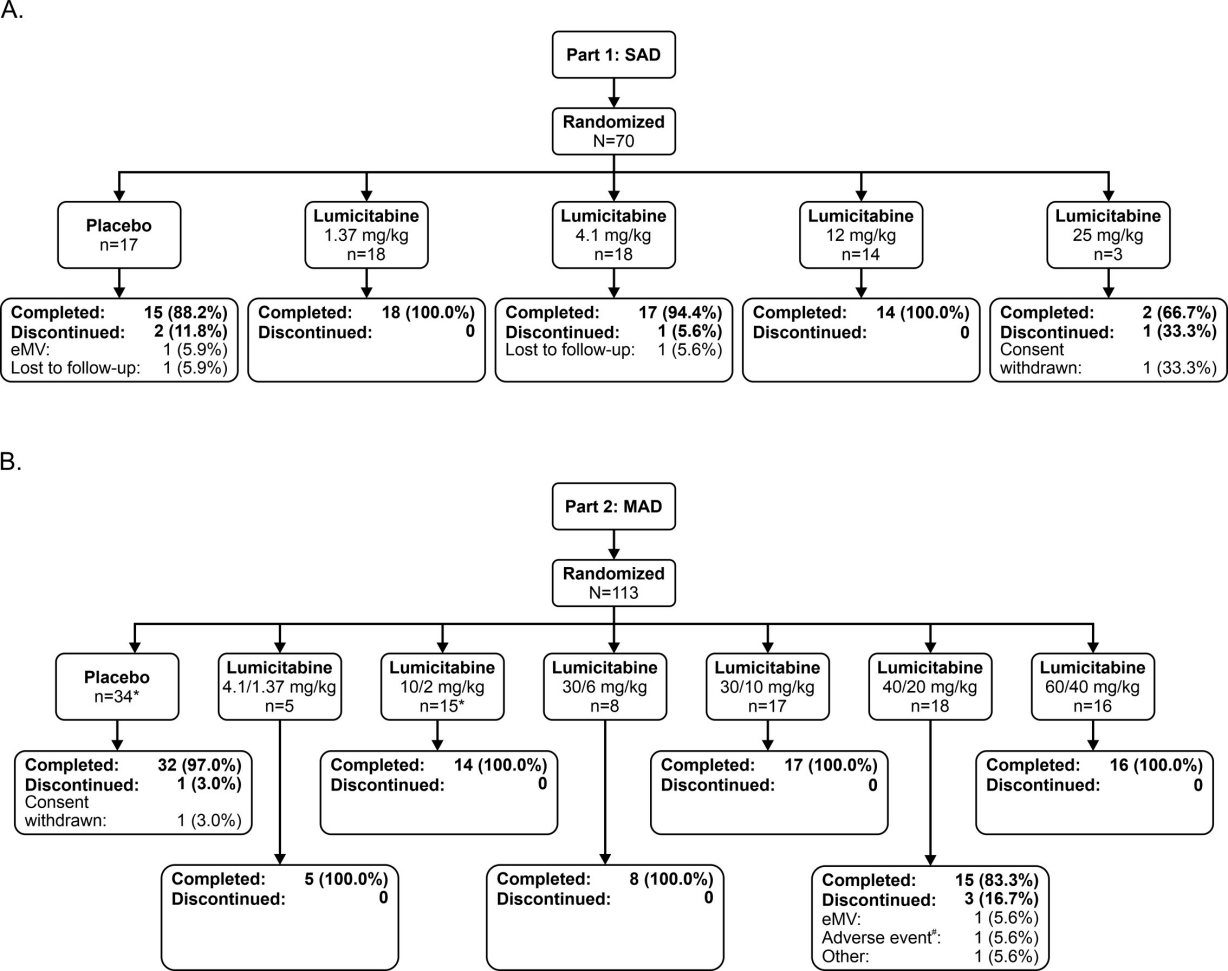
Patient disposition in SAD and MAD phases of Study 1. eMV, Need for endotracheal mechanical ventilation; ITT, intent-to-treat; MAD; multiple-ascending dose; SAD, single-ascending dose. Percentages based on the total number of patients randomized and treated in each treatment group. Patients completed SAD phase through Day 7 visit and completed MAD phase through Day 28 visit. *1 patient randomized but not treated. ^#^Event of Neutropenia.

In the SAD phase of Study 1, 70 patients were randomized and treated in 8 countries: United Kingdom (n = 16), Panama (n = 13), Thailand (n = 13), France (n = 10), Chile (n = 6), New Zealand (n = 6), Colombia (n = 5), and Australia (n = 1). Of these, 53 were randomized to 1 of 4 doses of lumicitabine (1.37 mg/kg, n = 18; 4.1 mg/kg, n = 18; 12 mg/kg, n = 14; 25 mg/kg, n = 3), while 17 were randomized to placebo. In all, 66 patients completed the SAD phase of the study (Fig 1). In the MAD phase, 113 patients were randomized in 11 countries: Japan (n = 38), USA (n = 22), Thailand (n = 14), Panama (n = 12), United Kingdom (n = 6), Chile (n = 6), Taiwan (n = 5), France (n = 4), New Zealand (n = 3), Colombia (n = 2), and Australia (n = 1). Of the 113 patients, 79 were randomized to 1 of 6 regimens of lumicitabine (4.1/1.37 mg/kg, n = 5; 10/2 mg/kg, n = 15; 30/6 mg/kg, n = 8; 30/10 mg/kg, n = 17; 40/20 mg/kg, n = 18; 60/40 mg/kg, n = 16), whereas 34 were randomized to placebo. Two patients did not receive any study treatment (1 each in the lumicitabine and placebo groups). In all, 107 patients completed the MAD phase of the study (Fig 1).

**Fig 2:**
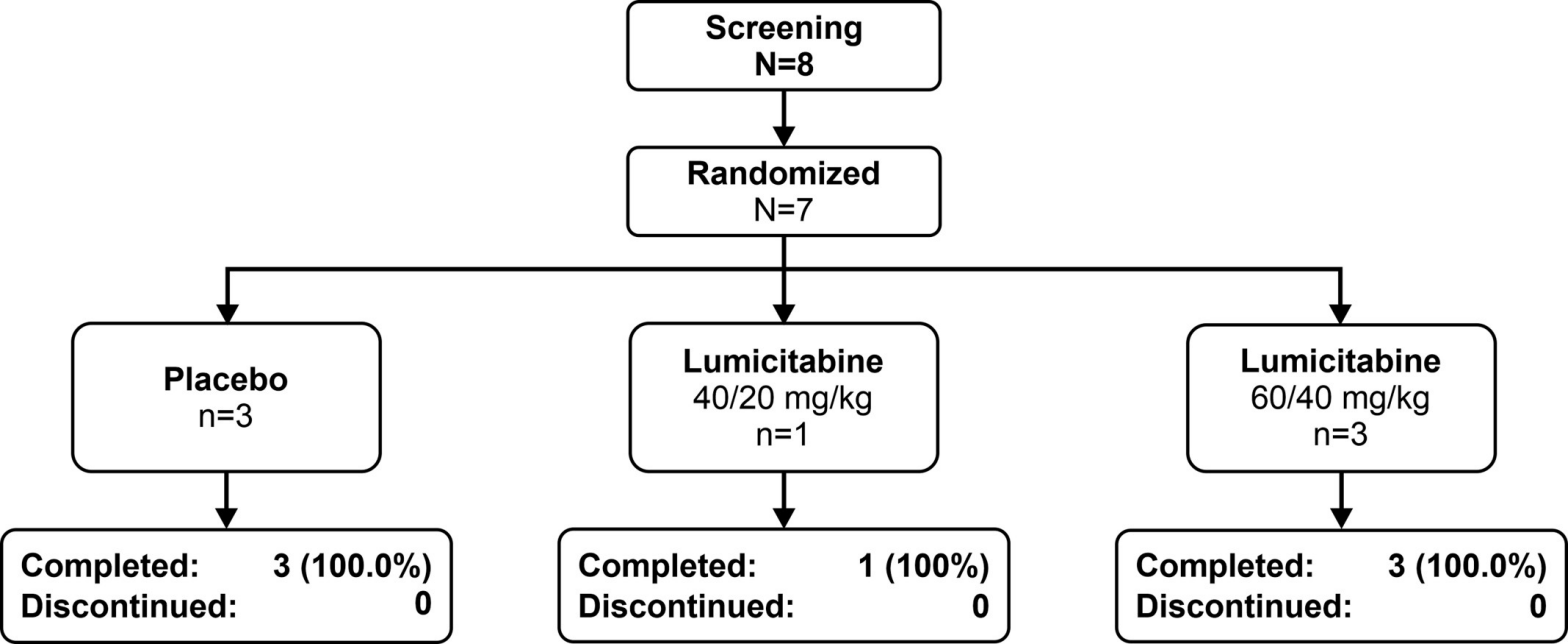
Patient disposition in Study 2.

In Study 2, 8 patients were screened from 4 study sites in Japan. Of these, 7 were randomized and treated (40/20 mg/kg, n = 1; 60/40 mg/kg, n = 3, placebo, n = 3). One eligible patient did not undergo randomization and treatment due to the unavailability of the study drug at the study site (Fig 2).

### Baseline and clinical characteristics

In both (SAD/ MAD) phases of Study 1, the lumicitabine and placebo groups were balanced for age and ethnicity (Table 1). The majority of the enrolled patients were male (SAD: 60.4% in lumicitabine groups, 64.7% in placebo group; MAD: 66.7% in lumicitabine groups, 48.5% in placebo group). The mean time between the onset of RSV symptoms and the first dose for the overall lumicitabine groups was 6.5 days (SAD) and 4.5 days (MAD); the corresponding placebo values were 4.3 and 4.4 days, respectively. The majority of patients in the lumicitabine groups (∼94% in both parts) had at least one concomitant medication, with the most common being salbutamol (SAD: 27 [50.9%]; MAD: 27 [34.6%]) and paracetamol (SAD: 21 [39.6%]; MAD: 27 [34.6%]). About half of the patients in the lumicitabine groups had at least one medical history finding reported, the most frequent being prior infections and infestations (SAD: 10 [18.9%]; MAD: 16 [20.5%]).

**Table 1:** Demographics and baseline characteristics of Study 1 (ITT Analysis Set).

	SAD phase						MAD phase							
	Lumicitabine dose (mg/kg)					Placebo (n = 17)	Lumicitabine dose (mg/kg)							Placebo (n = 33)
	1.37 (n = 18)	4.1 (n = 18)	12 (n = 14)	25 (n = 3)	Overall (n = 53)		4.1/1.37 (n = 5)	10/2 (n = 14)	30/6 (n = 8)	30/10 (n = 17)	40/20 (n = 18)	60/40 (n = 16)	Overall (n = 78)	
Age (months), Mean (SD)	4.0 (2.8)	4.2 (2.7)	5.7 (2.8)	5.0 (2.3)	4.6 (2.7)	4.6 (3.2)	5.0 (3.3)	3.2 (2.9)	2.3 (1.4)	3.5 (2.6)	4.2 (2.9)	4.2 (3.4)	3.7 (2.9)	3.9 (3.3)
Sex, n (%), Male	9 (50.0)	10 (55.6)	11 (78.6)	2 (66.7)	32 (60.4)	11 (64.7)	2 (40.0)	10 (71.4)	5 (62.5)	11 (64.7)	14 (77.8)	10 (62.5)	52 (66.7)	16 (48.5)
Ethnicity, n (%)														
Hispanic or Latino	5 (27.8)	7 (38.9)	4 (28.6)	2 (66.7)	18 (34.0)	6 (35.3)	0	3 (21.4)	2 (25.0)	7 (41.2)	5 (27.8)	4 (25.0)	21 (26.9)	8 (24.2)
Not Hispanic or Latino	13 (72.2)	11 (61.1)	10 (71.4)	1 (33.3)	35 (66.0)	11 (64.7)	5 (100)	11 (78.6)	6 (75.0)	10 (58.8)	13 (72.2)	12 (75.0)	57 (73.1)	25 (75.8)
Race, n (%)														
White	11 (61.1)	9 (50.0)	5 (35.7)	1 (33.3)	26 (49.1)	14 (82.4)	0	7 (50.0)	5 (62.5)	4 (23.5)	4 (22.2)	1 (6.3)	21 (26.9)	11 (33.3)
African American							0	0	0	0	0	2 (12.5)	2 (2.6)	2 (6.1)
Asian	3 (16.7)	4 (22.2)	5 (35.7)	0	12 (22.6)	1 (5.9)	5 (100)	7 (50.0)	3 (37.5)	6 (35.3)	11 (61.1)	9 (56.3)	41 (52.6)	17 (51.5)
NHOPI							0	0	0	1 (5.9)	0	0	1 (1.3)	0
Other	4 (22.2)	5 (27.8)	4 (28.6)	2 (66.7)	15 (28.3)	2 (11.8)	0	0	0	6 (35.3)	3 (16.7)	4 (25.0)	13 (16.7)	3 (9.1)
BMI (kg/m^2^) Mean (SD)	16.7 (2.1)	16.9 (1.9)	16.8 (2.0)	17.6 (0.8)	16.8 (1.9)	16.7 (2.3)	16.0 (1.6)	16.6 (2.1)	16.4 (3.0)	16.7 (2.0)	17.3 (2.0)	16.8 (2.2)	16.8 (2.1)	17.0 (5.0)
Head circumference (cm), Mean (SD)	40.5 (2.8)	40.1 (2.7)	42.0 (2.9)	41.5 (2.3)	40.8 (2.8)	41.8 (3.6)	41.8 (4.1)	40.2 (3.1)	40.1 (2.9)	40.1 (3.7)	41.7 (3.1)	41.6 (3.1)	40.9 (3.2)	40.6 (3.0)
Start of RSV symptoms to first dose (days)														
Mean (SD)	3.9 (1.1)	8.9 (13.9)	6.9 (6.2)	6.4 (3.3)	6.5 (8.8)	4.3 (1.5)	6.3 (2.5)	4.3 (3.0)	4.7 (1.7)	4.8 (1.8)	4.3 (1.0)	4.0 (0.7)	4.5 (1.8)	4.4 (2.1)
Median	3.80	4.90	4.80	7.50	4.60	4.10	6.70	4.60	4.55	4.95	4.00	4.15	4.40	4.20
IQR	1.5	4.5	4.5	6.4	3.2	1.8	3.9	3.4	2.1	2.2	1.1	0.7	1.8	1.4

BMI, body mass index; IQR, interquartile range; ITT, intent-to-treat; MAD, multiple-ascending dose; NHOP, Native Hawaiian or Other Pacific Islander; RSV, respiratory syncytial virus; SAD, single-ascending dose; SD, standard deviation.

In the SAD study phase, 45 (lumicitabine: 35 [66.0%]; placebo: 10 [58.8%]) patients were positive for RSV-A, 23 (lumicitabine: 16 [30.2%]; placebo: 7 [41.2%]) patients were positive for RSV-B, and 2 (3.8%) patients from the lumicitabine groups were positive for both RSV-A and RSV-B. The median viral load at the baseline was 5.58 log_10_ PFUe/mL for the overall lumicitabine groups, and 5.42 log_10_ PFUe/mL for the placebo group.

In the MAD study phase, for patients with nasal aspirate, 33 (lumicitabine: 24 [57.1%]; placebo: 9 [45.0%]) patients were positive for RSV-A, 28 (lumicitabine: 18 [42.9%]; placebo: 10 [50.0%]) patients were positive for RSV-B, and 1 (5.0%) patient from the placebo group was positive for both RSV-A and RSV-B. For patients with nasal swabs, 29 (lumicitabine: 24 [70.6%]; placebo: 5 [41.7%]) patients were positive for RSV-A and 17 (lumicitabine: 10 [29.4%]; placebo: 7 [58.3%]) patients were positive for RSV-B. The median viral load at the baseline for the overall lumicitabine groups was 5.71 log_10_ PFUe/mL for patients with nasal aspirate and 4.84 log_10_ PFUe/mL for patients with nasal swabs; the corresponding values for the placebo group were 5.32 and 4.13 log_10_ PFUe/mL, respectively.

In Study 2, all 7 patients were of Asian origin, the median age was 9 months, with most being male (n = 6, 85.7%). Mean duration of RSV symptoms from onset to time of randomization was 3.6 days. The median viral load at the baseline was 7.8 log_10_ copies/mL. Of the 7 patients, 4 (lumicitabine: 2/4 [50.0%]; placebo: 2/3 [66.7%]) were infected with RSV-A, and 3 (lumicitabine: 2/4 [50.0%]; placebo: 1/3 [33.3%]) with RSV-B.

### Safety and tolerability

In Study 1 SAD and MAD phases, 30 (56.6%) and 57 (73.1%) patients in the lumicitabine groups had at least one AE, as compared to 4 (23.5%) and 20 (60.6%) patients in the corresponding placebo groups.

In the SAD phase, the most frequently reported AEs included thrombocytosis (n = 7), followed by vomiting (n = 5) and diarrhea (n = 4). Overall, 8 (15.1%) patients in the lumicitabine group experienced at least 1 AE considered as possibly related to the study drug by the investigator. In the lumicitabine groups, 2 patients experienced serious treatment-emergent AEs of sinus tachycardia and phlebitis; one patient in the placebo group experienced Grade 3 bacterial pneumonia (Table 2). None of the serious events were considered related to the study drug by the investigator.

**Table 2:** Summary of reported treatment-emergent AEs by treatment groups in Study 1 (Safety analysis set).

	SAD phase, n (%)						MAD phase, n (%)							
	Lumicitabine dose (mg/kg)					Placebo (n = 17)	Lumicitabine dose (mg/kg)							Placebo (n = 33)
	1.37 (n = 18)	4.1 (n = 18)	12 (n = 14)	25 (n = 3)	Overall (n = 53)		4.1/1.37 (n = 5)	10/2 (n = 14)	30/6 (n = 8)	30/10 (n = 17)	40/20 (n = 18)	60/40 (n = 16)	Overall (n = 78)	
**Patients with ≥1 AE**	14 (77.8)	8 (44.4)	6 (42.9)	2 (66.7)	30 (56.6)	4 (23.5)	4 (80.0)	9 (64.3)	4 (50.0)	13 (76.5)	13 (72.2)	14 (87.5)	57 (73.1)	20(60.6)
**AEs occurring in >1 patient**														
Neutropenia	0	0	0	0	0	0	0	0	0	0	3 (16.7)	3 (18.8)	6 (7.7)	1 (3.0)
Thrombocytosis	4 (22.2)	1 (5.6)	1 (7.1)	0	6 (11.3)	1 (5.9)	1 (20.0)	1 (7.1)	1 (12.5)	0	0	1 (6.3)	4 (5.1)	0
Sinus tachycardia	0	1 (5.6)	0	1 (33.3)	2 (3.8)	0	0	0	0	0	0	0	0	0
Diarrhea	2 (11.1)	0	1 (7.1)	1 (33.3)	4 (7.5)	0	2 (40.0)	2 (14.3)	1 (12.5)	1 (5.9)	2 (11.1)	3 (18.8)	11 (14.1)	5 (15.2)
Instillation site swelling	0	0	0	0	0	0	0	0	0	0	2 (11.1)	0	2 (2.6)	0
Vomiting	2 (11.1)	1 (5.6)	0	1 (33.3)	4 (7.5)	1 (5.9)	0	1 (7.1)	1 (12.5)	3 (17.6)	2 (11.1)	5 (31.3)	12 (15.4)	1 (3.0)
Pyrexia	1 (5.6)	0	0	0	1 (1.9)	0	0	0	0	1 (5.9)	1 (5.6)	1 (6.3)	3 (3.8)	1 (3.0)
Nasopharyngitis	0	0	0	0	0	0	0	0	0	0	1 (5.6)	1 (6.3)	2 (2.6)	4 (12.1)
LRT infection	0	0	0	0	0	0	0	0	0	0	0	0	0	2 (6.1)
URT infection	0	0	0	0	0	0	0	1 (7.1)	0	1 (5.9)	0	0	2 (2.6)	2 (6.1)
Conjunctivitis	1 (5.6)	1 (5.6)	0	0	2 (3.8)	0	0	0	1 (12.5)	0	0	1 (6.3)	2 (2.6)	1 (3.0)
RSV bronchiolitis	0	0	0	0	0	0	0	0	0	2 (11.8)	0	0	2 (2.6)	0
ALT increased	0	0	0	0	0	0	1 (20.0)	1 (7.1)	0	0	1 (5.6)	3 (18.8)	6 (7.7)	0
AST increased	1 (5.6)	0	1 (7.1)	0	2 (3.8)	1 (5.9)	1 (20.0)	2(14.3)	0	0	3 (16.7)	3 (18.8)	9 (11.5)	1 (3.0)
Platelet count increased	1 (5.6)	0	0	0	1 (1.9)	0	0	0	0	0	0	0	0	2 (6.1)
Reticulocytes count increased	0	0	0	0	0	1 (5.9)	0	0	0	0	0	2 (12.5)	2 (2.6)	0
Blood CPK increased	1 (5.6)	0	1 (7.1)	0	2 (3.8)	1 (5.9)	0	0	0	0	0	0	0	0
Rhinorrhea	0	0	0	0	0	0	0	0	0	0	1 (5.6)	1 (6.3)	2 (2.6)	0
Dermatitis contact	0	0	0	0	0	0	0	1 (7.1)	0	0	0	1 (6.3)	2 (2.6)	0
Dermatitis diaper	0	1 (5.6)	0	0	1 (1.9)	0	2 (40.0)	2 (14.3)	1 (12.5)	1 (5.9)	0	3 (18.8)	9 (11.5)	4 (12.1)
Erythema	0	0	0	0	0	0	0	0	0	2 (11.8)	1 (5.6)	0	3 (3.8)	1 (3.0)
Eczema	1 (5.6)	0	0	0	1 (1.9)	0	2 (40.0)	1 (7.1)	0	1 (5.9)	1 (5.6)	1 (6.3)	6 (7.7)	1 (3.0)
Miliaria	0	0	0	0	0	0	0	0	0	1 (5.9)	1 (5.6)	0	2 (2.6)	0
Rash	1 (5.6)	0	1 (7.1)	0	2 (3.8)	0	0	1 (7.1)	0	0	1 (5.6)	1 (6.3)	3 (3.8)	2 (6.1)
**Patients with ≥1 treatment-related AE**	3 (16.7)	0	5 (35.7)	0	8 (15.1)	0	2 (40.0)	2 (14.3)	1 (12.5)	1 (5.9)	5 (27.8)	6 (37.5)	17 (21.8)	6 (18.2)
**Patients with ≥1 serious AE**	1 (5.6)	0	0	1 (33.3)	2 (3.8)	1 (5.9)	0	0	0	1 (5.9)	2 (11.1)	0	3 (3.8)	1 (3.0)
Sinus tachycardia	0	0	0	1 (33.3)	1 (1.9)	0	0	0	0	0	0	0	0	0
Pneumonia bacterial	0	0	0	0	0	1 (5.9)	0	0	0	0	0	0	0	0
Phlebitis	1 (5.6)	0	0	0	1 (1.9)	0	0	0	0	0	0	0	0	0
Lymphadenitis	0	0	0	0	0	0	0	0	0	0	0	0	0	1 (3.0)
Neutropenia	0	0	0	0	0	0	0	0	0	0	1 (5.6)	0	1 (1.3)	0
RSV bronchiolitis	0	0	0	0	0	0	0	0	0	1 (5.9)	0	0	1 (1.3)	0
Respiratory failure	0	0	0	0	0	0	0	0	0	0	1 (5.6)	0	1 (1.3)	0
**Patients with ≥1 AE leading to drug interruption**	0	0	0	0	0	0	0	0	0	0	0	0	0	0

AE, adverse event; ALT, alanine aminotransferase increased; AST, aspartate aminotransferase increased; CPK, creatinine phosphokinase; LRT, lower respiratory tract; MAD, multiple-ascending dose; RSV, respiratory syncytial virus; SAD, single-ascending dose; URT, upper respiratory tract.

In the MAD phase, diarrhea was the most frequently reported AE in 16 (11 [14.1%] patients in lumicitabine, 5 [15.0%] in placebo) patients, followed by vomiting and diaper dermatitis in 13 patients each (vomiting: 12 [15.4%] patients in lumicitabine; 1 [3.0%] patient in placebo; diaper dermatitis: 9 [11.5%] patients in lumicitabine and 4 [12.1%] patients in placebo). Increased aspartate aminotransferase was reported in 10 patients (9 [11.5%] patients in lumicitabine and 1 [3.0%] patient in placebo). None of the AEs led to drug interruptions. Regarding clinical laboratory evaluations reported as AEs, there were slight (all maximum Grade 1 or 2) dose-related transient (return to baseline by day 11) increases in alanine aminotransferase (6 [7.7%] in overall lumicitabine, and none in placebo) and aspartate aminotransferase (9 [11.5%] in overall lumicitabine, and 1 [3.0%] in placebo). However, there was no change in bilirubin in any group. Most of these increases were considered at least possibly related to treatment. Neutropenia was reported as an AE in 7 patients (3 [16.7%] in the lumicitabine 40/20 mg/kg group, 3 [18.8%] in the lumicitabine 60/40 mg/kg group and 1 [3.0%] in placebo), and was considered at least possibly related to the study drug in 3 of these patients (2 [11.1%] in the 40/20 mg/kg group and 1 [6.3%] in the 60/40 mg/kg group). Two (11.1%) patients in the 40/20 mg/kg LD/MD group experienced an AE that led to the study drug discontinuation: One patient experienced hypokalemia, hypomagnesemia, bacterial pneumonia, respiratory failure, and fluid overload; the other patient experienced neutropenia. AEs in both patients resolved after drug discontinuation. Grade 3 events reported were respiratory failure in two patients in 40/20 mg/kg group and anemia in one patient in the 30/10 mg/kg group. There were no trends in the vital sign values.

Serious treatment-emergent AEs were reported in 3 patients in the lumicitabine groups (respiratory failure and RSV bronchiolitis, considered not related to the study drug; neutropenia, considered possibly related to the study drug) and in 1 patient in the placebo group (lymphadenitis not related to the study drug).

All 7 patients treated in Study 2 experienced at least one AE, majority being Grade 1 or 2 in severity. There were no AEs leading to permanent withdrawal of the study treatment. Grade 3 neutropenia was followed by a Grade 3 febrile neutropenia; both were reported in one patient in the 60/40 mg/kg LD/MD lumicitabine group. Additionally, Grade 2 neutropenia was reported in 1 patient in the same treatment group. These neutropenia events were considered at least possibly related to lumicitabine by the investigator. No deaths were reported during the studies.

In both studies, a transient decrease in the absolute neutrophil count in the lumicitabine groups was observed, with higher grade neutropenia being observed with increased doses and exposures (Table 3). In the Study 1 SAD phase, Grade 3 treatment-emergent abnormalities in the absolute neutrophil count occurred in 2 (14.3%) patients in the 12 mg/kg lumicitabine arm. In the Study 1 MAD phase, treatment-emergent abnormalities in the absolute neutrophil count occurred in 4 (12.1%) patients in the placebo arm (1 [3.0%] with Grade 2 and 3 [9.1%] with Grade 3). In the 40/20 mg/kg LD/MD group, such abnormalities occurred in 9 (50.0%) patients (2 [11.1%] with Grade 1; 3 [16.7%] with Grade 2; 2 [11.1%] with Grade 3, and 2 [11.1%] with Grade 4). In the 60/40 mg/kg LD/MD group, such abnormalities occurred in 8 (50.0%) patients (2 [12.5%] with Grade 1; 1 [6.3%] with Grade 2, and 5 [31.3%] with Grade 4). These decreases typically became evident by day 11, and were resolved by day 28. They were not associated with other complications.

**Table 3:** Summary of neutropenia events based on laboratory values by treatment groups in Study 1 and Study 2 (Safety analysis set).

	Study 1*														Study 2^#^		
	SAD phase, n (%)						MAD phase, n (%)										
	Lumicitabine dose(mg/kg)					Placebo (n = 17)	Lumicitabine dose (mg/kg)							Placebo (n = 33)	Lumicitabine dose (mg/kg)		Placebo (n = 3)
	1.37 (n = 18)	4.1 (n = 18)	12 (n = 14)	25 (n = 3)	Overall (n = 53)		4.1/1.37 (n = 5)	10/2 (n = 14)	30/6 (n = 8)	30/10 (n = 17)	40/20 (n = 18)	60/40 (n = 16)	Overall (n = 78)		40/20 (n = 1)	60/40 (n = 3)	
Grade 1–2	0	1 (5.6)	1 (7.1)	0	2 (3.8)	1 (5.9)	1 (20.0)	4 (28.6)	3 (37.5)	1 (5.9)	5 (27.8)	3 (18.8)	17 (21.8)	1 (3.0)	0	1 (33.3)	1 (33.3)
Grade 3	0	0	2 (14.3)	0	2 (3.8)	0	0	0	0	1 (5.9)	2 (11.1)	0	3 (3.8)	3 (9.1)	0	1 (33.3)	0
Grade 4	0	0	0	0	0	0	0	0	0	0	2 (11.1)	5 (31.3)	7 (9.0)	0	0	1 (33.3)	0

MAD, multiple-ascending dose; SAD, single-ascending dose.

Neutropenia events were based on actual laboratory values and not per the investigator’s reporting.

*Grade was based on Division of Acquired Immunodeficiency Syndrome (DAIDS) Table for Grading the Severity of Adult and Pediatric Adverse Events Version 1.0—December 2004.

^#^Grade was based on Division of Microbiology and Infectious Diseases (DMID) Pediatric Toxicity Tables–November 2007.

Patients were only counted once at the worse grade or category at each direction (high and low) post-baseline.

In Study 2, the reduced absolute neutrophil count occurred in one patient (33.0%) in the placebo group (Grade 1) and in 3 patients (100.0%) in the 60/40 mg/kg LD/MD lumicitabine group (Grade 1, Grade 3 and Grade 4 severity in one patient each).

### Antiviral activity

#### Study 1

**Fig 3:**
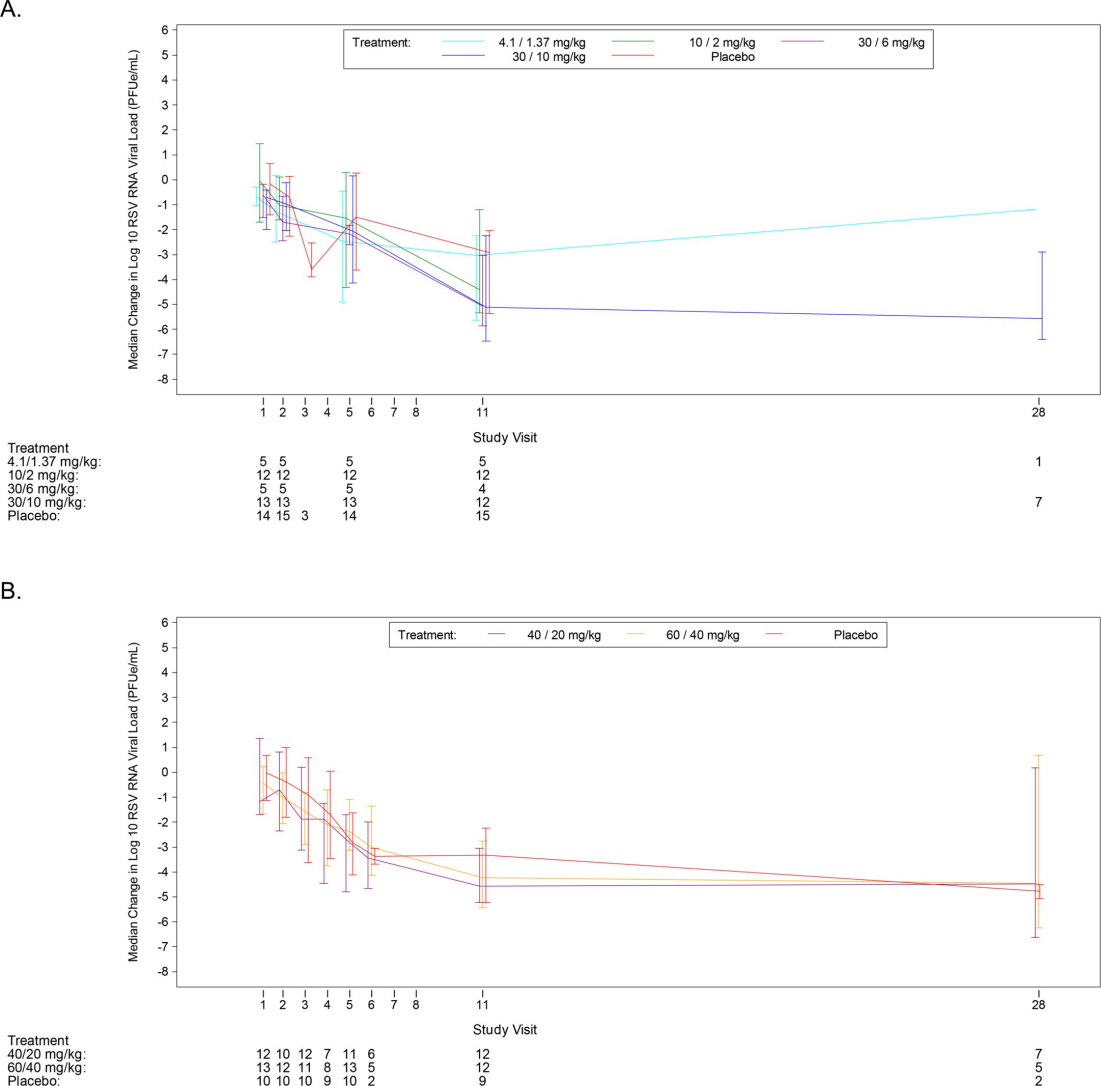
Median Change from Baseline in RSV RNA Log_10_ Viral Load Over Time in MAD Phase (Study 1, Full Analysis Set)–(A) Nasal Aspirates and (B) Nasal Swabs. MAD, multiple-ascending doses; PFUe, plaque-forming unit equivalents; RNA, ribonucleic acid; RSV, respiratory syncytial virus.

In the SAD and MAD phases, the baseline mean log_10_ RSV RNA in nasal aspirates ranged from 4.70 to 6.58 log_10_ PFUe/mL. The mean log_10_ RSV RNA viral load in nasal aspirates at completion (1.03 to 3.81 log_10_ PFUe/mL) were reduced from the baseline and were similar regardless of treatment (Table 4 and Fig 3).

**Table 4:** Antiviral activity and clinical outcomes by treatment groups in Study 1 (Full analysis set).

	SAD phase					MAD phase						
	Lumicitabine dose (mg/kg)				Placebo (n = 17)	Lumicitabine dose (mg/kg)						Placebo (n = 33)
	1.37 (n = 18)	4.1 (n = 18)	12 (n = 14)	25 (n = 3)		4.1/1.37 (n = 5)	10/2 (n = 14)	30/6 (n = 8)	30/10 (n = 17)	40/20 (n = 18)	60/40 (n = 16)	
**Log_10_ RSV RNA concentration (Log_10_ PFUe/mL)**												
Baseline, n	15	17	14	3	16	5	12	5	13	12	13	15
Mean (SD)	4.93 (1.31)	5.57 (1.33)	6.19 (0.79)	4.70 (1.36)	5.12 (1.64)	5.18 (1.09)	5.53 (1.70)	6.58 (0.96)	5.58 (1.38)	4.67 (1.14)	4.77 (0.88)	5.09 (1.42)
Completion visit, n	14	15	12	2	15	1	1	0	7	7	5	1
Mean (SD)	2.19 (1.45)	2.47 (1.52)	3.81 (1.57)	2.76 (2.27)	2.75 (1.97)	1.57 (1.23)	1.74 (1.58)	2.08 (1.66)	1.03 (1.25)	0.29 (0.60)	1.00 (1.77)	1.75 (1.55)
**Log**_**10**_ **RSV viral load parameters**												
AUC_0-24h_ of log_10_ viral load (log_10_ PFUe x day/mL), n	15	17	14	3	16	5	12	5	13	12	13	15
Mean (SD)	4.27 (1.63)	5.00 (1.31)	5.74 (1.23)	4.61 (0.37)	4.88 (1.80)	4.59 (0.97)	5.30 (1.34)	5.79 (1.34)	4.94 (1.45)	4.23 (1.34)	4.25 (1.04)	4.66 (1.34)
LS mean difference (active minus placebo) (95% CI)	−1.3 (−2.48, −0.19)	−0.3 (−1.06, 0.50)	−0.1 (−0.93, 0.72)	0.2 (−0.92, 1.41)		−0.1 (−0.79, 0.51)	0.3 (−0.22, 0.76)	−0.1 (−0.80, 0.55)	−0.1 (−0.60, 0.36)	−0.3 (−0.87, 0.27)	−0.4 (−0.94, 0.19)	
AUC_0-5_ days of log10 viral loads (log10 PFUe*Days 0-5/mL)^a,b^	-	-	-	-	-	5	12	5	13	12	13	15
Mean (SD)	-	-	-	-	-	16.69 (5.899)	20.27 (7.679)	23.53 (6.130)	19.50 (7.009)	13.25 (7.113)	14.92 (4.816)	18.77 (5.891)
Difference (95% CI)	-	-	-	-	-	−1.3 (−4.200, 1.659)	1.4 (−0.789, 3.532)	0.0 (−3.198, 3.270)	0.8 (−1.320 2.974)	−2.1 (−.681, 0.563)	−0.9 (−3.557, 1.741)	-
Peak viral load (log_10_ PFUe/mL), n	15	17	14	3	16	5	12	5	13	12	13	15
Mean (SD)	4.56 (1.74)	5.26 (1.31)	6.09 (1.23)	4.86 (0.16)	5.21 (1.63)	4.86 (0.93)	5.53 (1.17)	5.76 (1.53)	5.00 (1.47)	4.31 (1.66)	4.73 (1.05)	4.89 (1.17)
LS mean difference (active minus placebo) (95% CI)	−1.5 (−2.73, −0.31)	−0.3 (−1.12, 0.53)	−0.0 (−0.91, 0.84)	0.2 (−1.05, 1.41)	-	0.0 (−0.78, 0.83)	0.0 (−0.57, 0.66)	−0.4 (−1.23, 0.42)	−0.3 (−0.84, 0.30)	0.5 (−1.35, 0.38)	0.2 (−1.01, 0.70)	
Viral load slope (log_10_ PFUe/mL/hour), n	14	14	13	3	15	5	12	5	13	10	12	15
Mean (SD)	−0.030 (0.05)	−0.045 (0.05)	-0.029 (0.04)	-0.010 (0.04)	-0.029 (0.05)	-0.05 (0.05)	-0.03 (0.04)	-0.07 (0.04)	-0.04 (0.03)	-0.03 (0.05)	-0.04 (0.03)	-0.04 (0.04)
LS mean difference (active minus placebo) (95% CI)	−0.002 (−0.037, 0.034)	−0.015 (−0.050, 0.020)	0.002 (−0.035, 0.039)	0.018 (−0.042, 0.078)	-	−0.007 (−0.047, 0.033)	0.014 (−0.017, 0.044)	−0.019 (−0.061, 0.023)	0.002 (−0.028, 0.031)	−0.023 (−0.062, 0.016)	−0.035 (−0.073, 0.002)	
**Time to non-detectability (hours), n**	15	17	14	3	16	5	12	5	13	12	13	15
Log-rank p-value versus placebo	0.84	0.92	0.97	0.64	-	0.458	0.828	0.694	0.317	0.678	0.319	-
**Time to resolution of RSV symptoms, n**	18	18	14	3	17	5	13	8	16	18	16	32
Patients with RSV symptoms resolved n (%)	11 (61.1)	6 (33.3)	8 (57.1)	3 (100)	10 (58.8)	4 (80.0)	12 (92.3)	7 (87.5)	15 (93.8)	17 (94.4)	13 (81.3)	27 (84.4)
**Time to resolution from first dose of study drug, n**	11	6	8	2*	10	4	12	7	15	17	13	27
Mean (SD)	4.78 (2.32)	6.50 (3.41)	4.91 (1.58)	4.53 (1.88)	5.26 (3.02)	12.68 (7.63)	12.59 (10.05)	19.23 (8.47)	13.66 (7.63)	9.84 (9.56)	8.62 (2.64)	9.56 (6.60)
Log-rank p-value versus placebo	0.592	0.244	0.438	0.522	-	0.422	0.547	0.176	0.511	0.885	0.641	-

**Note:** Baseline is the last assessment prior to the first dose of study drug. Completion visit was day 7 for SAD phase and day 28 for MAD phase.

AUC_0-24h_, Area under the concentration-time curve from time zero to 24 hours post-dose; CI, confidence interval; LS, least squares; MAD, multiple-ascending dose; PFUe, plaque-forming unit equivalents; RNA, ribonucleic acid; RSV, respiratory syncytial virus; SAD, single-ascending dose; SD, standard deviation.

All the parameters were assessed using nasal aspirates. For doses 40/20 mg, 60/40 mg nasal swabs were used to assess log_10_ RSV RNA concentration and log_10_ RSV viral load parameters.

*Three patients had symptoms resolved, but only two had a time to resolution.

In the SAD phase, the baseline-adjusted least square (LS) mean for AUC_0-24h_ of log_10_ viral load increased across the lumicitabine doses; it was significantly lower in the 1.37 mg/kg group than the placebo group (adjusted LS mean difference of −1.3, 95% CI: −2.48 to −0.19). There was an increasing trend in the baseline-adjusted LS means peak viral load with greater lumicitabine doses (Table 4), with the 1.37 mg/kg group significantly lower than the placebo group (adjusted LS mean difference of −1.5, 95% CI: −2.73 to −0.31). The proportion of patients with resolved RSV symptoms in the lumicitabine groups ranged from 33.3% to 100%, compared to 58.8% in the placebo group. The mean time to symptom resolution from the first study drug dose ranged from 4.5 to 6.5 days in the lumicitabine groups, compared to 5.3 days in the placebo group.

In the MAD phase, the baseline-adjusted LS mean values for AUC_0-24h_, the peak viral load, and the viral load slope were similar across treatments, with no significant mean estimate differences between the lumicitabine groups and relative to placebo. The mean AUC_0-5days_ was similar across all groups; the estimated difference in means between each lumicitabine group and placebo was not statistically significant. The proportion of patients whose RSV symptoms resolved in the lumicitabine groups ranged from 80% to 94%, compared to 84.4% in the placebo group. The mean time to symptom resolution from the first study drug dose ranged from 8.6 to 19.2 days across the lumicitabine dose groups and was 9.6 days in the placebo group.

**Fig 4:**
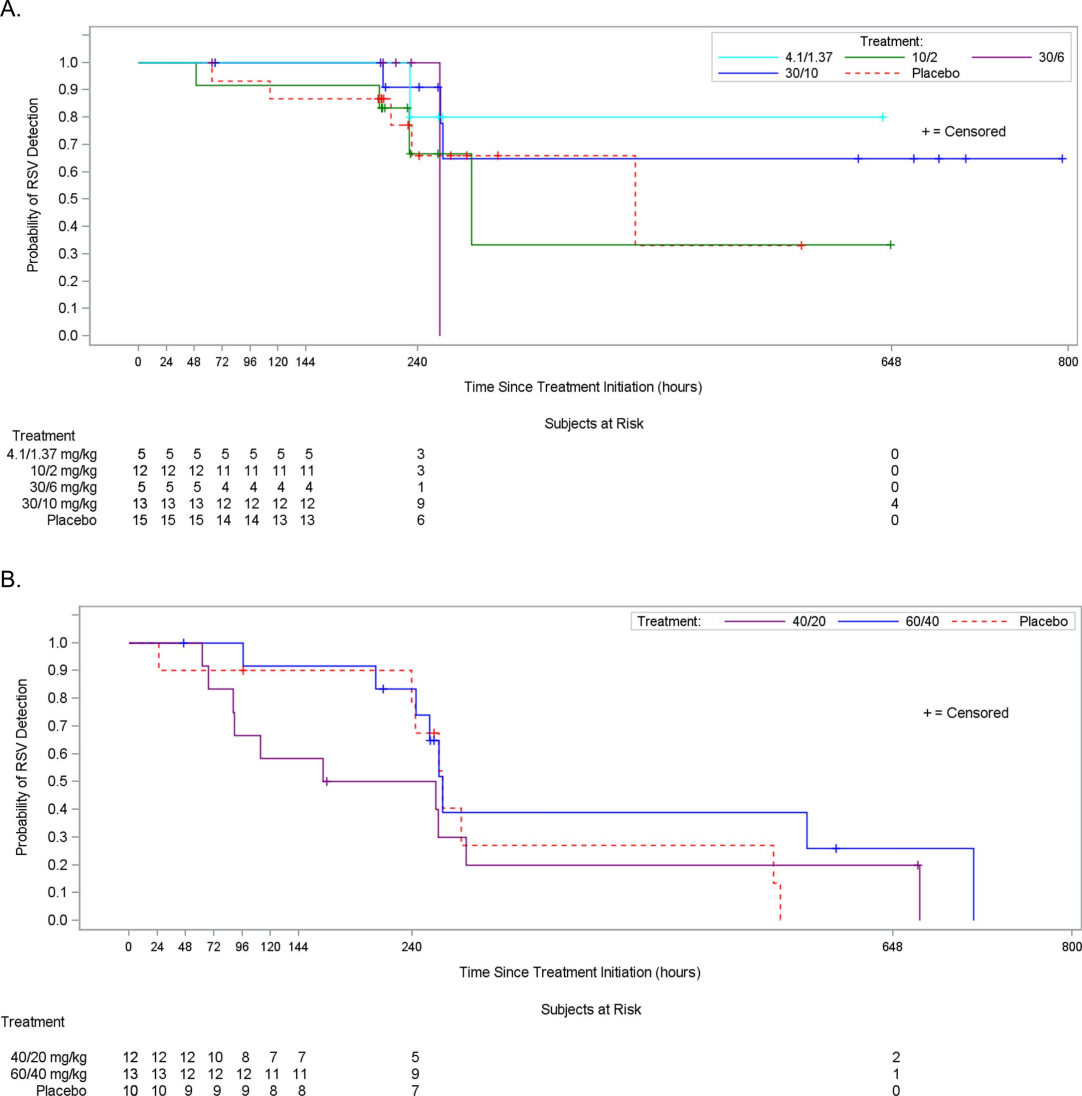
Time to RSV Non-Detectability in MAD Phase (Study 1, Full Analysis Set)–(A) Nasal Aspirates and (B) Nasal Swabs. MAD, multiple-ascending dose; RSV, respiratory syncytial virus. Time to non-detectability was defined as the relative time in hours from the first dose of study drug until the first post-baseline time point when the viral load reached non-detectability. Subjects whose viral load did not reach non-detectability were censored at their last RSV assessment. Time to non-detectability was not defined if a subject did not have any post-baseline viral load result.

In both phases of Study 1, the differences in the time to non-detectability between the lumicitabine groups and placebo were not significant, as demonstrated by log rank p-values (Fig 4).

**Fig 5:**
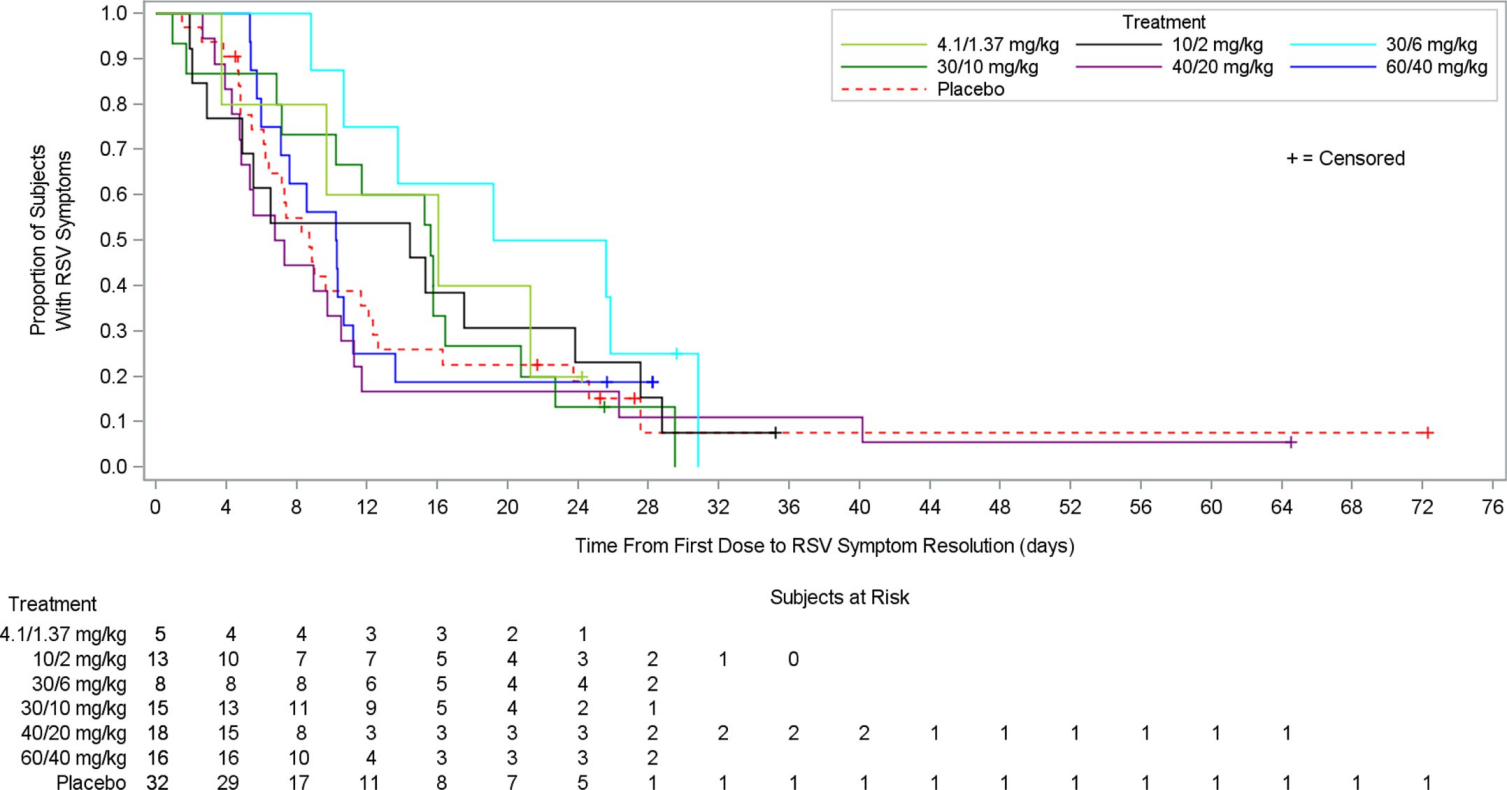
Time from first dose to RSV symptom resolution in MAD phase (Study 1, full analysis set). MAD, multiple-ascending dose; RSV, respiratory syncytial virus.

There was no significant difference in the time to symptom resolution between the lumicitabine groups and placebo (Fig 5).

#### Study 2

**Fig 6:**
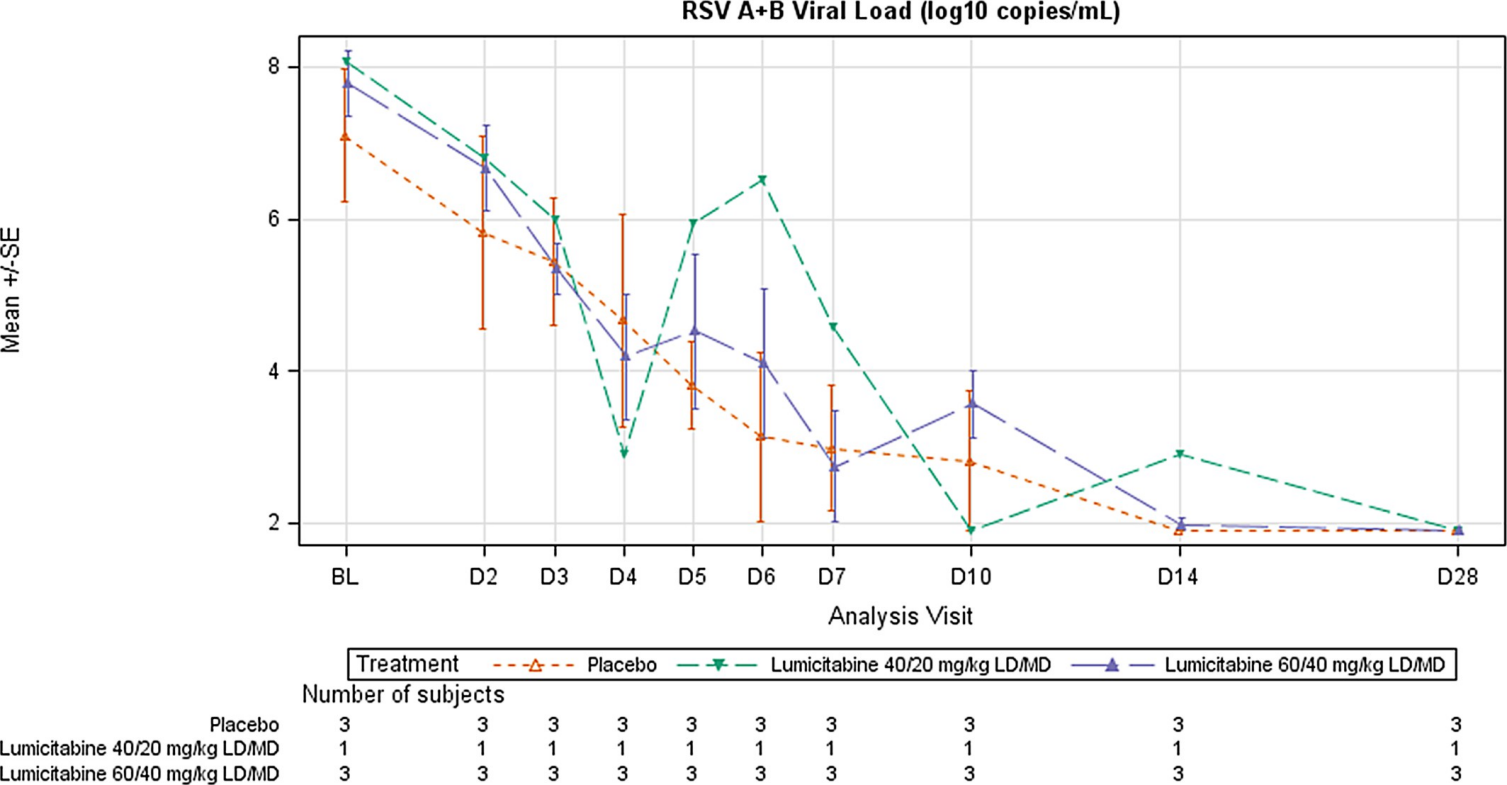
Mean (±SE) change from baseline in RSV RNA log_10_ viral load over time (Study 2, ITT analysis set). ITT, intent-to-treat; LD, loading dose; MD, maintenance dose; RNA, ribonucleic acid; RSV, respiratory syncytial virus; SE, standard error.RSV-A viral load LOQ (limit of quantification) = 3.00 log_10_ copies/mL., LOD (limit of detection) = 2.79 log_10_ copies/mL. RSV-B viral load LOQ = 2.40 log_10_ copies/mL., LOD = 1.90 log_10_ copies/mL. Results <LOQ and >LOD (target detected) are imputed with 2.90 or 2.15 log_10_ copies/mL for resp. RSV-A or RSV-B. Results <LOD (target not detected) are imputed with 1.90 log_10_ copies/mL.

The baseline RSV RNA viral load ranged from 5.71 to 8.71 log_10_ copies/mL in the placebo arm, compared to 7.03 to 8.53 log_10_ copies/mL in the lumicitabine arms. The pattern of viral load decay observed in the lumicitabine groups was similar as compared to the placebo group. The mean changes from the baseline in the RSV RNA viral load over time were similar in the lumicitabine 40/20 mg/kg LD/MD (n = 1) and the lumicitabine 60/40 mg/kg LD/MD (n = 3) arms compared to the placebo arm (n = 3) (Fig 6), with the viral load being undetectable by day 28 for all patients.

### Pharmacokinetics

The PK parameters, AUC and C_max_ were approximately dose-proportional over the range of doses studied in Study 1. There was a dose-dependent increase in the C_max_ of ALS-008112 across the tested MAD dose range; a 14.6-fold increase in LD (4.1 mg/kg to 60 mg/kg) resulted in a 16.3-fold increase in C_max_. An 18.3-fold increase in the day 1 total dose resulted in a 12.9-fold increase in the area under the plasma concentration-time curve over the day (AUC_0-24h_). The mean day 5 ALS-008112 AUC_0-24h_ at the highest dose level (60/40 mg/kg LD/MD) was 18,790 ng.h/mL (Table 5). The mean exposures of the lumicitabine groups in Study 1 and Study 2 were comparable.

**Table 5:** Population pharmacokinetic estimates of exposure of ALS-008112, the active nucleoside analog of lumicitabine, based on treatment groups (Study 1 and Study 2).

	Study 1										Study 2	
	SAD phase				MAD phase							
	Lumicitabine (mg/kg)				Lumicitabine (mg/kg)						Lumicitabine (mg/kg)	
	1.37 (n = 18)	4.1 (n = 17)	12 (n = 13)	25 (n = 3)	4.1/1.37 (n = 5)	10/2 (n = 14)	30/6 (n = 8)	30/10 (n = 17)	40/20 (n = 18)	60/40 (n = 16)	40/20 (n = 1)	60/40 (n = 3)
ALS-008112 C_max_	129.5 (43.19)	404.3 (63)	1269 (55.86)	1909 (20.91)	285 (25.81)	867 (49.66)	2431 (34.45)	1895 (44.08)	3142 (38.26)	4653 (51.44)	6184	7003 (54.35)
Day 1 (%CV), ng/mL												
ALS-008112 C_max_	-	-	-	-	172.1 (29.09)	211.8 (41.02)	591.2 (44.23)	991.3 (46.18)	1867 (41.22)	3380 (46.85)	3261	5112 (32.57)
Day 5 (%CV), ng/mL												
ALS-008112 AUC_0-24 h,_	350.9 (14.89)	959.9 (15.02)	2574 (10.09)	5122 (12.73)	1305 (10.04)	2466 (11.34)	6508 (8.843)	7459 (15.62)	10940 (11.88)	16770 (10.28)	12700	17800 (4.01)
Day 1 (%CV), ng.h/mL												
ALS-008112 AUC_0-24 h,_	-	-	-	-	1114 (7.467)	1529 (8.405)	3912 (8.483)	6117 (7.843)	10820 (13.76)	18790 (9.45)	11840	20500 (3.20)
Day 5 (%CV), ng.h/mL												

Data represented as mean (CV%).

AUC, area under the plasma concentration-time curve; C_max_, maximum plasma concentration; CV, coefficient of variation, MAD, multiple-ascending dose; SAD, single-ascending dose.

### Viral drug resistance

The baseline and post-baseline RSV L-gene sequencing data were available for 40/78 and 13/33 patients in the lumicitabine and placebo groups, respectively, in the MAD phase of Study 1. Data were similarly available for all patients in the lumicitabine (n = 4) and placebo (n = 3) groups in Study 2.

At the last evaluable on-treatment time point with sequencing data available, none of the patients in the lumicitabine groups, either in the MAD phase of Study 1 or in Study 2, had emerging amino acid substitutions at the 4 lumicitabine resistance-associated L-protein amino acid positions 628, 789, 795, or 796.

## Discussion

The given phase 1b and phase 2b studies evaluated the safety, PK, and efficacy of lumicitabine in infants hospitalized with RSV infection. They were undertaken following promising outcomes from preclinical studies and an RSV-A human challenge study in healthy adults [10, 11, 14]. This challenge study demonstrated that lumicitabine treatment resulted in more rapid RSV clearance, and a greater reduction in the RSV viral load and the RSV symptom score compared with placebo. Additionally, lumicitabine was found to be generally safe; AEs were balanced in terms of frequency and intensity across treatment arms with no evidence of drug-related hematologic abnormalities.

The RSV-A human challenge study results were incorporated into a population viral kinetic and PK/PD model to estimate the therapeutic effectiveness of lumicitabine, so as to guide dose selection in adult and pediatric patients [12]. The pediatric PK model was coupled with a semi-mechanistic pediatric lung model from monkeys, which estimated the formation of the pulmonary ALS-008112 and NTP at a given plasma exposure. For the proposed starting dose in infants, the maximum human plasma exposures obtained in adults, the lack of significant safety findings and the plasma exposures of ALS-008112 required to maintain NTP concentration in the target tissues were considered. The lumicitabine dose projections were used to establish the starting dose such that sub-therapeutic concentrations were avoided in infants and neonates.

Considering that RSV is an acute disease, the dosing regimen with LD and MD in the MAD phase was designed to rapidly achieve and maintain minimal therapeutic concentrations of the lung NTP required for the inhibition of viral replication. The highest dose in the SAD phase of Study 1 (25 mg/kg) resulted in an ALS-008112 mean plasma AUC_0-24h_ of approximately 5122 ng.h/mL, and an exposure range lower than studied in the RSV-A human challenge study [11]. In the MAD phase of Study 1, the ALS-008112 mean plasma AUC_0-24h_ for the highest dosing regimen (60/40 mg/kg) was 16,770 ng.h/mL on day 1 and 18,790 ng.h/mL on day 5. Both exceeded the exposures that were efficacious in the RSV-A human challenge study [11].

In Study 1 and Study 2, there were no significant differences between the lumicitabine-treated groups and the placebo groups in reducing the nasal RSV viral load, the time to viral undetectability, and the resolution of RSV symptoms despite exposures in some dosing cohorts being greater than those eliciting RSV viral load reduction in the RSV-A human challenge study. The semi-mechanistic pharmacokinetic model developed in adults was extrapolated to infant populations. However, the potential for reduced drug distribution in infant lungs, or lower intracellular active triphosphate formation (not featured in the model) cannot be excluded. Higher than anticipated doses might be required to achieve clinical benefit in children. However, a study of lumicitabine in hospitalized adults with natural RSV infection failed to demonstrate RSV antiviral activity, despite exposures exceeding those in the human challenge study (NCT02935673).

There are several plausible reasons for the discrepancy in the antiviral response seen between the RSV-A human challenge study in adults [11] and those in infants with natural RSV infection. In the human challenge study, treatment was initiated as soon as the virus was detectable. This occurred well before the observed peak of the viral load preceding the development of RSV symptoms in the placebo arm. In contrast, in self-limited natural infections, the development of RSV symptoms led to carers seeking later assistance, with early symptoms often being mild. In Study 1 and Study 2, the mean time between the onset of RSV symptoms and the first dose of treatment was about 4 days. Infants had peak viral load values prior to study treatment initiation. Thus, there was limited opportunity to demonstrate potential suppression of the virus by the investigational drug. The human challenge study was conducted in adult volunteers with pre-existing RSV immune memory [11]. However, in the infant studies, a primary RSV infection was considered most likely. In the human challenge study, infection was largely limited to the upper respiratory tract. Contrastingly, in these natural RSV infection studies, the more severe lower respiratory tract symptoms motivated parents to seek care for their infants, resulting in the need for hospitalization. The failure to achieve clinical efficacy despite demonstration of RSV antiviral activity has been observed in the development of other RSV antivirals [15].

Viral resistance to ALS-008112 in these studies was not observed at any of the RSV polymerase L-amino acid positions of interest in patients treated with lumicitabine [13]. The emergence of resistance could not be fully evaluated owing to the lack of availability of the paired baseline and the post-baseline RSV L-gene sequencing data in Study 1. Although the resistance profile of RSV following exposure to lumicitabine is not fully known [11], nucleoside analogues widely used in antiviral therapy exhibit higher barriers to the emergence of viral resistance compared to other compound classes [16]. In *in vitro* studie*s*, ALS-008112 demonstrated a higher barrier to viral resistance development compared to an RSV fusion inhibitor. Four amino acid substitutions in the RSV L-protein (M628L, A789V, L795I, or I796V) were identified to be associated with viral resistance to AL-008112 [13]. At the last evaluable on-treatment time point, none of the patients in the lumicitabine groups with sequencing data available, either in the MAD phase of Study 1 or Study 2, had emerging amino acid substitutions at these positions.

Phase 1 lumicitabine studies of healthy adult volunteers did not demonstrate hematologic abnormalities. In infants, for both Study 1 and Study 2, the accumulating safety data displayed a dose-related increase in the incidence and severity of reversible neutropenia. Except for this finding, the safety data observed in these studies were consistent with previous data of lumicitabine in adults in the human challenge study [11] and with natural RSV infection (NCT02935673).

Viral respiratory infections are reported to be associated with hematological abnormalities [17], complicating the interpretation of neutropenia in therapeutic clinical studies of RSV. This association was illustrated within Study 1 and Study 2. Graded neutropenia was observed at the study entry prior to treatment, with emergent graded neutropenia in some patients assigned to placebo. When the dose-related increase in reversible neutropenia became apparent, recruitment in Study 1 had already been completed. However, Study 2 was halted by the sponsor. Following consultation with independent experts, new non-clinical studies were undertaken to further evaluate and assess these safety findings. Additional animal studies determined that hematopoietic and clastogenic effects could occur at lower blood concentrations of lumicitabine with non-human primates as compared to the other animal species previously tested (data to be published separately). Subsequently, the sponsor terminated all lumicitabine studies and decided to discontinue the development of lumicitabine for all indications, including RSV and the human metapneumovirus infection.

Study limitations included the small sample sizes within the dose cohorts of Study 1. Furthermore, Study 2 was terminated early, impacting the ability to perform statistical analysis.

In summary, lumicitabine was associated with a dose-related increase in the incidence and severity of reversible neutropenia. However, it failed to demonstrate antiviral activity in hospitalized infants, contrary to the findings of the previous RSV-A adult human challenge study.

## Supporting information

S1 Appendix Protocol of Study 1. (PDF)

S2 Appendix Protocol of Study 2. (PDF)

S3 Appendix CONSORT checklist. (DOC)

S4 Appendix Summary of clinical parameters and respiratory viruses detected. (DOCX)

S5 Appendix List of Institutional Review Boards. (DOCX)
