# Supplementary material for: Lumicitabine, an orally administered nucleoside analog, in infants hospitalized with respiratory syncytial virus (RSV) infection: Safety, efficacy, and pharmacokinetic results
Source: PLoS One. 2023 Jul 19;18(7):e0288271. doi: 10.1371/journal.pone.0288271 (PMC10355467; doi:10.1371/journal.pone.0288271)
Supplement: S1 Appendix — (PDF) [file pone.0288271.s001.pdf]

Janssen Research and Development *
Alios BioPharma
260 E. Grand Ave
South San Francisco, CA 94080

Protocol Number: ALS-8176-503

A Randomized, Double-blind, Placebo-controlled, 2-Part Study of Orally Administered ALS-008176 to Evaluate the Safety, Tolerability, Pharmacokinetics and Pharmacodynamics of Single Ascending Dosing and Multiple Ascending Dosing in Infants Hospitalized with Respiratory Syncytial Virus (RSV) Infection

Drug name: ALS-008176

(Also known as AL-8176)

IND Number: 122,969

EudraCT Number: 2013-005104-33

Date: 13 September 2016

Version Number: 6.0

*Janssen Research & Development is a global organization that operates through different legal entities in various countries. Therefore, the legal entity acting as the sponsor for Janssen Research & Development studies may vary, such as, but not limited to Janssen Biotech, Inc.; Janssen Products, LP; Janssen Biologics, BV; Janssen-Cilag International NV; Janssen, Inc; Janssen Sciences Ireland UC; or Janssen Research & Development, LLC. The term “sponsor” is used throughout the protocol to represent these various legal entities; the sponsor is identified on the Contact Information page that accompanies the protocol.

CONTACT INFORMATION

For up-to-date contact information, see Study Binder: Emergency Contact Sheet.

All serious pretreatment and serious adverse events occurring **from the time of** consent up to Study Completion Visit, regardless of relationship to study drug, must be reported via email to Medical Monitor and Pharmacovigilance within 24 hours of knowledge of the event.

Please contact the Medical Monitor by telephone for any questions about serious adverse event reporting.

Any fatal or life-threatening event must also be reported within 24 hours by telephone (see table below).

Medical Monitors	Telephone No.	E-mail
**Americas**		
[redacted] MD, FACP, FIDSA<br>PPD, US	Office: [redacted]<br>Mobile: [redacted]	[redacted]
**Europe**		
[redacted], MD<br>SPAIN	[redacted]	[redacted]
**Asia-Pacific**		
[redacted], MD<br>TAIWAN	Office: [redacted]<br>Mobile: [redacted]	[redacted]

**Sponsor Medical Director**<br>[redacted], MD<br>PPD US	[redacted]	[redacted]

**Pharmacovigilance**
inVentiv Health Global Safety and Pharmacovigilance<br>[redacted]INDIA): Tel No.: [redacted]
Fax No:<br>Latin American sites [redacted]<br>UK sites [redacted]
Other sites: E-mail:<br>SAEReceipt.International@inventivhealth.com

TABLE OF CONTENTS

CONTACT INFORMATION..2
TABLE OF CONTENTS..3
ABBREVIATIONS..7
DEFINITIONS..9
LIST OF STUDY DRUGS AND METABOLITES..9
SYNOPSIS..10

1.0 BACKGROUND AND RATIONALE..20

1.1 Background Information..20
1.2 Rationale for the Study..28
1.3 Rationale for Starting Dose Selection..28
1.3.1 Single Ascending Dose (SAD):..30
1.3.2 Multiple Ascending Dose..30

2.0 STUDY DESIGN..31

2.1 Summary..31
2.2 Part 1: Single Ascending Dose (SAD)..33
2.3 Part 2: Multiple Ascending Dose (MAD)..34

3.0 STUDY OBJECTIVES AND ENDPOINTS..36

3.1 Study Objectives..36
3.1.1 Primary Objective..36
3.1.2 Secondary Objectives..36
3.1.3 Exploratory Objectives..36
3.2 Study Endpoints..37
3.2.1 Primary Endpoint..37
3.2.2 Secondary Endpoints..37
3.2.3 Exploratory Endpoints..37

4.0 SELECTION AND WITHDRAWAL OF SUBJECTS..38

4.1 Study Population..38
4.2 Inclusion Criteria..38
4.3 Exclusion Criteria..39
4.4 Subject Screening and Enrollment..41
4.5 Subject Withdrawal..42
4.6 Subject Discontinuation..42
4.6.1 Subject Discontinuation Criteria..42
4.6.2 Procedures for Subjects Who Discontinue..43
4.6.3 Documentation of Discontinuation of Subjects..43

4.7 Study Discontinuation ... 43
5.0 TREATMENT OF SUBJECTS ... 43
5.1 Treatment Regimens ... 43
5.1.1 Cohort Progression Guidelines ... 44
5.1.2 Treatment Delay or Missed Doses ... 46
5.1.3 Partial Doses ... 47
5.1.4 Dose Reductions ... 47
5.2 Description of Study Drug ... 47
5.2.1 Study Drug ... 47
5.3 Dose Preparation and Administration ... 48
5.4 Ordering Study Drug ... 49
5.5 Drug Accountability ... 49
5.6 Disposition of Used, Partially Used, and Unused Vials ... 49
5.7 Concomitant Medications ... 49
5.8 Prohibited Medications ... 50
6.0 STUDY PROCEDURES ... 51
6.1 On-study Evaluations, Procedures, and Dosing ... 58
6.1.1 Diet, Fluid, and Activity ... 58
6.1.2 Serum Chemistries and Complete Blood Count with Differential ... 58
6.1.3 Pharmacokinetic Sampling ... 58
6.1.4 Central Laboratory Evaluations ... 60
6.1.5 RSV Evaluations ... 60
6.1.6 12-Lead Electrocardiograms ... 62
6.1.7 Vital Signs and Oxygen Saturation ... 62
6.1.8 Physical Examinations ... 62
6.1.9 Clinical Evaluation ... 62
7.0 SAFETY MONITORING AND REPORTING ... 63
7.1 Definitions ... 63
7.1.1 Pretreatment Events ... 63
7.1.2 Adverse Events ... 63
7.1.3 Treatment Emergent Adverse Events ... 63
7.1.4 Serious Adverse Events ... 63
7.2 Documenting and Reporting of AEs (including saes) ... 64
7.2.1 Documenting and Reporting Pretreatment Events ... 64
7.2.2 Documenting and Reporting Adverse Events ... 64
7.2.3 Assigning Attribution of Adverse Events ... 65
7.2.4 Classifying Action Taken with Study Drug ... 66

7.2.5 Classifying Adverse Event Outcome 66
7.2.6 Documenting and Reporting Serious Pretreatment Events and Serious Adverse Events 66
7.3 Follow-up of Adverse Events and Serious Adverse Events 68
7.4 Sponsor's Review of Adverse Events and Serious Adverse Events 68
7.5 Independent Data Monitoring Committee 68
7.6 Unblinding Procedures 69
8.0 STUDY VARIABLES AND MEASUREMENTS 69
8.1 Efficacy Variables/Measurements 69
8.2 Safety Variables/Measurements 69
8.2.1 Adverse Events 70
8.2.2 Medical History 70
8.2.3 Clinical Laboratory Measurements 70
8.2.4 Prior and Concomitant Medications 71
8.3 Pharmacokinetic Measurements 71
8.4 Pharmacodynamic Measurements 72
9.0 STATISTICAL CONSIDERATIONS 73
9.1 Study Design and Objectives 73
9.2 General Considerations 73
9.3 Study Endpoints 74
9.3.1 Primary Endpoint 74
9.3.2 Secondary Endpoints 74
9.3.3 Exploratory Endpoints 74
9.4 Determination of Sample Size 75
9.5 Randomization 75
9.6 Subject Disposition 76
9.7 Analysis Data Sets 77
9.8 Demographics and Baseline Characteristics 77
9.9 Interim Analysis 77
9.10 Safety Analyses 77
9.10.1 Adverse Events 77
9.10.2 Death 77
9.10.3 Vital Signs, Physical Examination, and Laboratory Assessments 77
9.10.4 Prior and Concomitant Medications 78
9.11 Pharmacokinetic Analysis 78
9.12 Pharmacodynamic Analysis 78

10.0 ADMINISTRATIVE CONSIDERATIONS 78
10.1 Study Compliance 79
10.2 Informed Consent and Protected Subject Health Information Authorization............ 79
10.3 Subject Screening Log 79
10.4 Case Report Forms... 79
10.5 Study Monitoring Requirements.. 80
10.6 Retention of Records.. 80
10.7 Confidentiality and Publication Policy 80
10.8 Conduct of Study and Protection of Human Subjects 81
11.0 REFERENCES.. 82
12.0 APPENDICES 83

In-text Figures

Figure 1-1. Study ALS-8176-503 Enrollment Status as of 5 August 2016 22
Figure 1-2. RSV Viral Load Over Time Following Administration of ALS-008176 or Placebo for 5 Days in the Intent-to-Treat-infected Population in Study ALS-8176-502 26
Figure 1-3. ALS-008112 Exposures in Infants 1-12 Months of Age Receiving 30 mg/kg LD and 10 mg/kg MD Regimen in Study ALS-8176-503........ 28
Figure 2-1. Preliminary Dose Escalation Schema 32

In–text Tables

Table 1-1. Blinded Incidence and Severity of Treatment-emergent AEs in SAD Portion of Study ALS-8176-503 (as of 5 August2016) 24
Table 1-2. Blinded Incidence and Severity of Treatment-emergent AEs in MAD Portion of Study ALS-8176-503 (as of 5 August 2016) 25
Table 1-3. Parameters Used to Determine Projected Efficacious Doses in Infants* . 29
Table 5-1. Part 1 SAD Dosing Regimen 44
Table 5-2. Part 2 MAD Dosing Regimen.. 44
Table 6-1. Schedule of Events: SAD Phase (Part 1) 52
Table 6-2. Schedule of Events: MAD Phase (Part 2) Twice-Daily Dosing 54
Table 6-3. Schedule of Events: MAD Phase (Part 2) Once-Daily Dosing................. 56
Table 8-1. Blood Volume per Body Weight in a 3.2-kg Infant................................. 70
Table 8-2. Clinical Laboratory Evaluations 71

ABBREVIATIONS

AE	Adverse event
ALT	Alanine aminotransferase
AST	Aspartate aminotransferase
$AUC_{0\text{-}12h}$	Area under the concentration-time curve from time zero to 12 hours postdose
$AUC_{0\text{-}24h}$	Area under the concentration-time curve from time zero to 24 hours postdose
$AUC_{0\text{-}tau}$	Area under the concentration-time curve during the dosing interval
$AUC_{0\text{-}inf}$	Area under the concentration-time curve from time zero to infinity
$AUC_{0\text{-}last}$	Area under the concentration-time curve from time zero to last quantifiable concentration
BP	Blood Pressure
CBC	Complete blood count
C_{last}	Last measurable blood concentration
C_{max}	Maximum measured drug concentration
C_{min}	Minimum measured drug concentration
COPD	Chronic obstructive pulmonary disease
CRA	Clinical research associate
CRF	Case report form
CSR	Clinical Study Report
DNA	Deoxyribonucleic acid
EC	Ethics Committee
EC_{50}	Half maximal effective concentration
EU	European Union
ECG	Electrocardiogram
FA	Full Analysis Data Set
FDA	Food and Drug Administration
GCP	Good Clinical Practice
HR	Heart rate
ICH	International Conference on Harmonisation
ICU	Intensive Care Unit
IDMC	Independent Data Monitoring Committee
IEC	Independent Ethics Committee
INR	International Normalization Ratio
IRT	Interactive Response Technology
K_i	Dissociation constant for inhibitor binding
LD	Loading dose
LRI	Lower respiratory infection
MAD	Multiple ascending dose

MD	Maintenance dose
MedDRA	Medical Dictionary for Regulatory Activities
MMR	Measles, mumps, and rubella
NP	Nasopharyngeal
NTP	Nucleoside triphosphate
PD	Pharmacodynamic(s)
PE	Physical examination
PI	Principal investigator
PK	Pharmacokinetic(s)
PT/PTT	Prothrombin time/partial thromboplastin time
Q12	Every 12 hours
QAM	Every morning
RBC	Red blood cell
RBV	Ribavirin
RNA	Ribonucleic acid
RR	Respiratory rate
RSV	Respiratory syncytial virus
RT-PCR	Reverse transcription polymerase chain reaction
SAD	Single ascending dose
SAE	Serious adverse event
SAP	Statistical analysis plan
SC	Scavenged
SOC	System organ class
$t_{1/2}$	Terminal elimination half-life
TBD	To be determined
t_{last}	Time to last measurable blood concentration
t_{max}	Time of maximum concentration
ULN	Upper limit of normal
USA	United States of America
WBC	White blood cell
WHO	World Health Organization
Λz	Terminal elimination rate constant

DEFINITIONS

Study drug	ALS-008176 or placebo
End of clinical phase of the study	Last MAD subject Day 28 follow-up visit, or resolution/stabilization of all adverse events, whichever is later.
End of treatment	Permanent discontinuation or completion of dosing
Enrollment	Enrollment is defined by the confirmed randomization.
Discontinuation	Subject discontinues study drug. The subject may still complete some study activities after discontinuing study drug. Informed consent has NOT been withdrawn.
Withdrawal	The subject's parents/guardians have withdrawn consent and the subject is no longer participating in study activities or dosing as of the time of the withdrawal of consent.

LIST OF STUDY DRUGS AND METABOLITES

Compound No.	Comment
ALS-008176	3',5'-bisisobutyrate prodrug of ALS-008112
ALS-008112	Parent nucleoside, major metabolite of ALS-008176
ALS-008136	5'-triphosphate of ALS-008112 (NTP), the active metabolite formed within cells
ALS-008144	Uridine metabolite of ALS-008112; major metabolite

SYNOPSIS

A Randomized, Double-blind, Placebo-controlled, 2-Part Study of Orally Administered ALS-008176 to Evaluate the Safety, Tolerability, Pharmacokinetics and Pharmacodynamics of Single Ascending Dosing and Multiple Ascending Dosing in Infants Hospitalized with Respiratory Syncytial Virus (RSV) Infection

**Protocol No.:**	ALS-8176-503
**Phase:**	1b
**Study Drug:**	ALS-008176
**IND No.:**	122,969
**EudraCT No.:**	2013-005104-33
**Background Therapy:**	Standard of care supportive therapy
**Comparator:**	Placebo
**Indication:**	Respiratory Syncytial Virus (RSV) Infection
**Study Design:**	This randomized, double-blind, placebo-controlled, 2-part study will assess the safety, tolerability, pharmacokinetics (PK), and pharmacodynamics (PD) of single and multiple doses of orally administered ALS-008176 in infants hospitalized with RSV infection.<br><br>**Part 1 (single ascending dose – SAD):** Subjects will receive a single dose from 1 of 3 ascending dose levels of ALS-008176 (1.37 mg/kg, 4.1 mg/kg, and 12 mg/kg) or placebo. Dosing of study drug in the fed state is encouraged.<br><br>Three additional cohorts (Cohorts 4, 5, and 6) may be evaluated at the discretion of the Sponsor, upon approval by the Independent Data Monitoring Committee (IDMC), based on an evaluation of the emerging PK profile and the safety profile.<br><br>Within each dosing cohort, subjects will be randomized to receive either ALS-008176 or placebo (n=approximately 24 per cohort; randomized in a ratio of 3 ALS-008176:1 placebo). Randomization will be stratified by age at time of hospital admission (≥1.0 to <2.0 months =28 to 59 days old, ≥2.0 to <6.0 months =60 to 181 days old, and ≥6.0 to ≤12.0 months =182 to 365 days old), with approximately 8 subjects being enrolled in each age stratum.

Figure 1. Preliminary Dosing Schema*

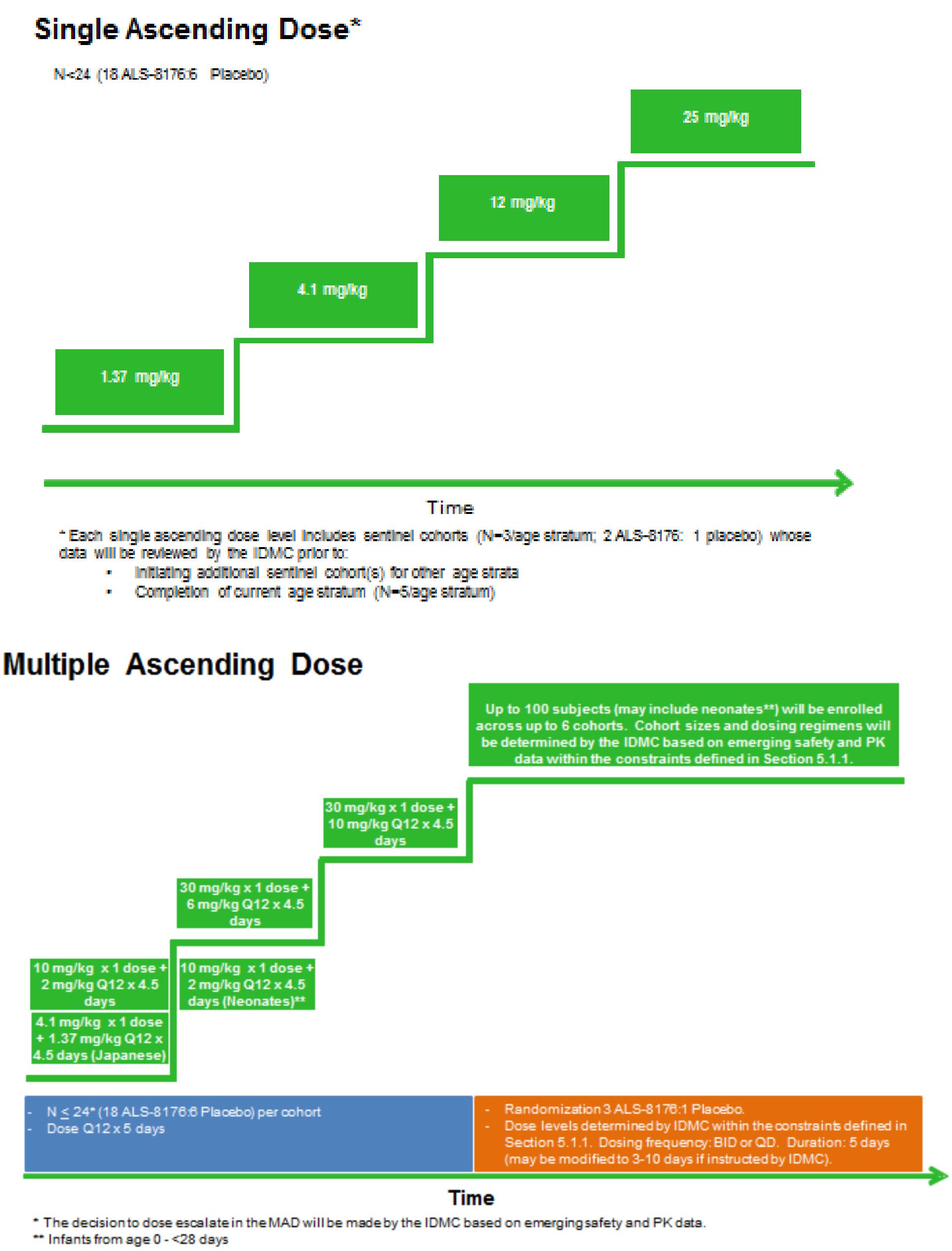

An age de-escalation approach will be utilized within each SAD dose cohort, i.e., subjects aged ≥6.0 to ≤12.0 months will be enrolled initially, followed by subjects ≥2.0 to <6.0 months, and then subjects ≥1.0 to <2.0 months, as follows:

- A sentinel group of 3 subjects in each age stratum will be enrolled first (randomized in a ratio of 2 ALS-008176:1 placebo).
- The IDMC will review the safety data through Day 7 and available

PK data for the sentinel subjects. Following this:

- The remaining 5 subjects in the age stratum will be enrolled (randomized in a ratio of 4 ALS-008176:1 placebo), and
- Enrollment in the next age stratum's sentinel cohort(s) will be initiated.

At no time will dose escalation occur for a younger age stratum before dosing in an older age stratum has been initiated for that dose, unless the IDMC determined that the safety profile supported such an approach.

Subjects will be evaluated over a 7-day period from the time of randomization. Additionally, all subjects will receive standard supportive care per local institution. Any subjects discharged from the hospital prior to Day 7 will be required to return as an outpatient for assessment on Day 7, when they will complete the study.

Dose escalation within Part 1 will not occur before the IDMC has reviewed and deemed acceptable:

- all safety data through study completion for all subjects in the current cohort or age stratum
- available PK data from the first 24 hours after dosing for at least 75% of the current cohorts' subjects (or a completed stratum within this cohort)

As of 25 April 2016, the SAD portion of the study has been completed and is now closed.

Part 2 (multiple ascending dose – MAD): Part 2 will commence when emerging PK and safety data from Part 1 have been evaluated and are deemed by the IDMC to be acceptable and sufficient to initiate multiple dosing.

Based on the lack of clinically relevant PK differences observed to date across the 3 age strata, infants of all 3 age strata (excluding neonates) will receive the same doses, unless otherwise instructed by the IDMC. Similarly, doses will be escalated in parallel in all 3 age strata for the second and subsequent MAD cohorts if the IDMC deems available PK and safety data in the current MAD cohort to be sufficient and authorizes dose escalation.

Hospitalized subjects with RSV infection will be assigned to 1 of up to 11 planned MAD regimens of ALS-008176 or placebo (see Figure 1). The number of dosing regimens and doses to be studied will be at the discretion of the Sponsor on the basis of the emerging PK and safety profiles, upon approval by the IDMC.

One or more cohorts will enroll (except when cohort enrollment is paused) neonates (<28 days old) if supported by emerging PK, safety, and efficacy

data in infants 1-12 months of age. The dose regimen(s) studied in neonates may be adjusted according to IDMC instructions.

In addition, Japanese infants will also be enrolled in Part 2 (see Section 1.1). Initially, Japanese patients received a 4.1 mg/kg loading dose followed by nine 1.37 mg/kg maintenance doses (4.1/1.37). The IDMC reviewed the emerging safety and PK data and, due to lack of differences in exposures in Japanese vs. non-Japanese subjects in this study, Japanese infants are now eligible for inclusion in all cohorts. There is no restriction on how many Japanese infants may be enrolled.

Within each dosing cohort, subjects will be randomized to receive either ALS-008176 or placebo in a ratio of 3 ALS-008176: 1 placebo. Randomization (except in the neonate cohort) will be stratified by age at time of hospital admission (≥1.0 to <2.0 months =28 to 59 days old, ≥2.0 to <6.0 months =60 to 181 days old, and ≥6.0 to ≤12.0 months =182 to 365 days old).

Up to 6 additional cohorts may be enrolled in the MAD Part 2 with a maximum of 100 additional subjects (including neonates) in these cohorts. Additionally, the following elements of the study may be adjusted, subject to approval by the IDMC (see also Sections 5.1.1 and 7.5):

- The size of each cohort and the frequency at which cohort data are reviewed. Enrollment will generally be allowed to continue during preparation and review of data by IDMC, unless otherwise instructed by the IDMC.
- Dosing frequency within a cohort may be once daily or twice daily
- The maintenance doses may be the same as the loading dose
- The duration of dosing within a cohort will be 5 days unless otherwise instructed by the IDMC, which may modify the duration within the range of 3 to 10 days, inclusive
- The duration of symptoms from first onset until randomization will be ≤5 days unless otherwise instructed by the IDMC, which may modify the duration to up to 7 days

Additionally, all subjects will receive standard supportive care as per local institution. Subjects will be evaluated over a 28-day period from the time of randomization. If they are discharged from the hospital prior to Day 28, they will be required to return for assessment as an outpatient on Day 5, if applicable, and on Days 11 and 28, when they will complete the study, assuming a 5-day treatment duration (see Table 6-2 for timing of visits for other treatment durations).

In both Parts 1 and 2, the planned dose escalation schema (Figure 1) may be modified by the Sponsor, upon approval by the IDMC, based on

	emerging PK and safety data. In all circumstances, however, there will be no more than a 3-fold increase in dose between dose levels.<br><br>Throughout the conduct of the study, the pharmacokinetics of ALS-008112 and ALS-008144 will be evaluated and reviewed. A pediatric population PK model will be updated with the additional data, and the plasma exposures of the subsequent dose will be simulated prior to dose escalation, as a safety check to ensure that the pharmacokinetics of ALS-008112 are not predicted to differ significantly from the intended exposures. Alterations in the planned dose escalation scheme will be made as necessary. In addition, under no circumstances will a planned pediatric dosing regimen exceed a projected average ALS-008112 $AUC_{0\text{-}24h}$ of 20,000 ng•h/mL. Furthermore, the decision to dose escalate between cohorts will be based on a review of safety and PK data by the Sponsor and IDMC.<br><br>The IDMC will review unblinded safety data on a regular basis and approve each dose escalation decision.<br><br>Subject safety will be monitored by regular assessment of the results of clinical laboratory tests, electrocardiograms (ECG), physical examination, vital signs, and adverse events as reported by parents/legal guardians/medical staff.
**Study Objectives:**	In neonates (<28 days old) and infants (≥1.0 to ≤12.0 months of age) who are hospitalized with RSV infection:<br><br>**Primary:**<br>• To evaluate the safety and tolerability of single and multiple doses of ALS-008176<br><br>**Secondary:**<br>• To evaluate the pharmacokinetics of ALS-008112 and ALS-008144 (and other metabolites, if applicable) in blood following single and multiple doses of ALS-008176<br>• To evaluate the antiviral activity of ALS-008176 after single and multiple doses of ALS-008176<br>• To determine if ALS-008176 exposure results in the emergence of resistant strains of RSV<br><br>**Exploratory:**<br>• To evaluate the impact of ALS-008176 on the clinical course of RSV infection<br>• To evaluate the relationship between viral kinetics and clinical outcomes<br>• To evaluate the impact of ALS-008176 on biomarkers potentially associated with the inflammatory response induced by acute RSV

	infection<br>• To evaluate the pharmacokinetics of ALS-008112 and ALS-008144 (and other metabolites, if applicable) in nasal swabs following multiple doses of ALS-008176, if an acceptable testing methodology can be established
**Endpoints**	**Primary:**<br>• Safety data including, but not limited to, adverse events, physical examinations, vital signs, 12-lead ECGs and clinical laboratory results (including chemistry and hematology)<br>**Secondary:**<br>• PK parameters of ALS-008112 and ALS-008144 (and other metabolites, as applicable) in blood following single dose administration: C_{max}, t_{max}, $t_{1/2}$, $AUC_{0\text{-}12h}$, $AUC_{0\text{-}24h}$, $AUC_{0\text{-}inf}$ or $AUC_{0\text{-}last}$<br>• PK parameters of ALS-008112 and ALS-008144 (and other metabolites as applicable) in blood following repeat dose administration: C_{max}, C_{min}, t_{max}, $t_{1/2}$, $AUC_{0\text{-}24h}$, $AUC_{0\text{-}tau}$, $AUC_{0\text{-}inf}$ or $AUC_{0\text{-}last}$<br>• RSV viral RNA concentrations in nasal swabs or aspirates as measured by quantitative RT-PCR<br>• Changes in the RSV polymerase that result in reduced sensitivity to ALS-008112<br>**Exploratory:**<br>• Length of hospital stay<br>• Need and duration of supplemental oxygen requirement<br>• Need and duration of ICU stay<br>• Need and duration of mechanical ventilation<br>• Need and duration of non-invasive ventilatory support<br>• Time to resolution of RSV signs or symptoms, such as runny nose, wheeze, cough, tachypnea<br>• Relationship between viral kinetics and various clinical outcome measures (e.g., the relationship between RSV RNA viral load and oxygen supplementation, duration of hospitalization)<br>• Biomarkers potentially associated with the inflammatory response induced by acute RSV infection<br>• Concentrations of ALS-008112 and ALS-008144 (and other metabolites as applicable) in nasal secretions, if an acceptable testing methodology can be established

**Duration of Treatment and Study Period:**	Part 1 (SAD): Single dose; study duration is approximately 7 days.<br>Part 2 (MAD): Twice daily or once daily dosing for 5 consecutive days (unless otherwise instructed by the IDMC, which may select a dosing duration of 3-10 days); study duration is approximately 28 days.
**Number of Sites and Location:**	Approximately 110, including but not limited to, sites in Europe (UK, France, Romania), Asia Pacific (Australia, New Zealand, Taiwan, Thailand, Japan), South Africa, and North/Latin America (Canada, United States, Panama, Colombia, Chile) will participate.
**Sample Size:**	Part 1 (SAD): Up to 72 subjects were to enroll in SAD cohorts. As of 25 April 2016, 70 subjects had enrolled in the SAD portion of the study, which is now closed.<br>Part 2 (MAD): As of 5 August 2016, 52 subjects have enrolled across 5 MAD cohorts, some of which (e.g., 30/10) are actively recruiting (see Figure 1). Up to an additional 100 subjects will enroll in up to 6 additional cohorts. The maximum anticipated enrollment in the MAD portion of the study, taking into account these numbers as well as anticipated enrollment in ongoing cohorts during the protocol version 6 review process is 190 subjects (52 enrolled subjects + up to 23 additional neonates + up to 15 additional subjects at 30/10 dose level+100 additional subjects in future cohorts).
**Inclusion Criteria:**	1. Subject's parent(s)/legal guardian(s) has provided signed and dated informed consent and authorization to use protected health information, as required by national and local regulations.<br>2. In the investigator's opinion, the subject's parent(s)/legal guardian(s) understands and is able to comply with protocol requirements, instructions, and protocol-stated restrictions, and is likely to complete the study as planned.<br>3. Male or female infant who<br>• is ≥1.0 to ≤12.0 months of age (inclusive), defined at the time of hospital admission, or <28 days of age (neonate cohort only). Note: all subjects, including neonates, must have been discharged from the hospital after birth and are now being admitted due to an RSV related illness<br>• has been diagnosed with RSV infection based on study-supplied BINAX NOW RSV test or an RSV PCR or any other RSV assay conducted at the clinical trial site. NOTE: A subject remains eligible if any RSV result is positive. (RSV-specific PCR run locally is strongly encouraged, but not required.) Coinfection with other respiratory viruses or bacterial coinfection in addition to RSV infection is permissible.<br>• has been hospitalized for <96 hours (at the time of randomization) for confirmed RSV infection (NOTE: nosocomial RSV infection is

<table>
<tr><td></td><td>excluded)
• has had symptoms consistent with RSV infection (e.g., runny nose, cough, sneezing, fever, or tachypnea) for ≤ 5 days at the time of randomization (unless otherwise instructed by the IDMC, which may modify the duration to up to 7 days)
4. With the exception of the RSV-related illness, the subject is in otherwise good health as deemed by the investigator, based on the findings of a medical evaluation including medical history, physical examination, laboratory tests, and ECG
5. Creatinine clearance is not below the lower limit of normal for the subject’s age (Schwartz equation calculation preferred, however alternative equations may be utilized to determine eligibility if deemed acceptable by the investigator and medical monitor).</td></tr>
<tr><td>Exclusion Criteria:</td><td>1. History of or concurrent clinically significant medical illness (not directly attributable to the acute RSV infection) – including, but not limited to cardiovascular, respiratory, renal, gastrointestinal, hematologic, neurologic, endocrinologic, immunologic, musculoskeletal, oncologic, or congenital disorders – as judged by the investigator. Specifically excluded conditions include but are not limited to:
a. Immunosuppressed state
b. Bronchopulmonary dysplasia
c. Congenital heart disease
d. Down’s syndrome
2. Prematurity, defined as gestational age <37 weeks at birth
3. Subjects receiving invasive endotracheal mechanical ventilation
4. Subjects who are thought to have a poorly functioning gastrointestinal tract (i.e., unable to absorb drugs or nutrition via enteral route). NOTE: The use of intravenous fluids is not exclusionary as long as the investigator believes the patient’s gastrointestinal tract still functions properly (i.e., is able to absorb drugs or nutrition).
5. Subjects with clinically significant laboratory abnormalities which are deemed by the investigator to represent a safety risk to participation in this study. Other laboratory parameters outside the reference range for the subject’s age may be included if the investigator considers the abnormalities unlikely to introduce additional risk factors and will not interfere with data interpretation. A single repeat laboratory evaluation (under appropriate conditions, e.g., not hemolyzed) is allowed for eligibility determination.
6. Any condition that, in the opinion of the investigator, would</td></tr>
</table>

compromise the study or the well-being of the subject or prevent the subject from meeting the study requirements

7. Clinically significant abnormal ECG findings, as judged by the investigator or qualified designee
8. Subjects anticipated to be discharged from the hospital in <24 hours from the time of randomization
9. Exclusionary medications include:
   a. Herbal supplements which have evidence of adversely affecting absorption and clearance mechanisms (e.g., strong inhibitors of OAT3) within 21 days prior to randomization
   b. The following prescription medications:
      - Use of systemic medications (either chronically (i.e., >14 days for neonates and infants <2 months old, or >28 days for subjects ≥2-≤12 months of age) or within the 21 days prior to randomization) which are known to modulate the host immune response and/or increase viral shedding such as corticosteroids or other immunomodulatory therapies. The only exception is systemic corticosteroids will be acceptable if they are not taken chronically for a non-RSV-related indication.
      - Prescription medications used within 14 days prior to randomization to treat the RSV infection itself (e.g., ribavirin, intravenous immunoglobulin). Prescription medications intended to treat the symptoms/sequelae of the RSV infection are permitted.
      - Prescription medications which are known to be strong inhibitors of the OAT3 transporter, within 21 days prior to randomization (see *Prohibited Medications*, Section 5.8)
   c. Investigational drug trial medications within 30 days or 5 half-lives (whichever is longer) prior to randomization
   d. Prior exposure to an investigational vaccine
   e. MMR vaccine within 1 week prior to screening, according to parent report
   f. Prior exposure to ALS-008176
   g. Prior exposure to palivizumab or other RSV prophylactic medication (approved or investigational)
10. Infants who are breastfeeding and their mother is taking any of the exclusionary medications described in exclusion criterion 9.

	11. Infants with another child in the household who has enrolled in the study.<br>12. Infants with known fructose intolerance (due to sorbitol in study medication)
**Dosage Form and Strength:**	ALS-008176 and placebo will be supplied in bottles as a powder, to be formulated as a suspension in sterile water. Instructions for preparation of study formulation can be found in the Pharmacy Manual.
**Dose Regimen:**	Each weight-based dose of ALS-008176 will be administered orally as a suspension.<br>Part 1 (SAD): single dose<br>Part 2 (MAD): Once daily or twice daily dosing for 5 consecutive days (for a 5-day treatment duration). Regardless of dosing frequency, the loading dose (Dose 1) should be administered as soon as possible after randomization.<br>In each twice daily dosing regimen, the loading dose (Dose 1) will be followed by a twice daily maintenance dose regimen (Doses 2–10; Figure 1). The first maintenance dose (Dose 2) will be given between 8-18 hours after the loading dose (Dose 1) to facilitate getting the subject on an established hospital dosing regimen. All subsequent doses will be given per the hospital's regular twice daily dosing times ±1 hour.<br>In once daily dosing regimens, Dose 2 will be given between 21-27 hours after Dose 1 to facilitate getting the subject on an established hospital dosing regimen. All subsequent doses will be given per the hospital's regular once daily dosing times ±1 hour.<br>Subjects will receive a total of 10 doses administered over 5 days if dosed twice daily or a total of 5 doses administered over 5 days if dosed once daily, assuming a 5-day treatment duration.<br>ALS-008176 can be administered without regard to food.<br>If instructed by the IDMC, the maintenance dose may be the same dose as the loading dose.
**Predosing and Concomitant Medications:**	Nil

1.0 BACKGROUND AND RATIONALE

1.1 BACKGROUND INFORMATION

RSV is an RNA virus and a member of the *Pneumoviridae* family, which also includes human metapneumovirus (hMPV). The RSV season occurs during winter months in regions with temperate climates in the Northern and Southern Hemispheres and throughout the year or peaks semiannually in tropical regions (Yusuf et al. 2007; Bloom-Feshbach et al. 2013).

RSV infection and resulting clinical sequelae usually lasts 1–2 weeks and results in mild cold-like symptoms in the majority of adults. However, infection can lead to severe lower respiratory infection (LRI) in vulnerable populations such as infants, the elderly, immunocompromised and subjects with chronic obstructive pulmonary disease (COPD) or asthma. RSV infection does not induce protective immunity, and neutralizing antibody responses diminish over time. As such, there is a potential for infants to become infected more than once, even within a single RSV season.

RSV is a leading cause of lower respiratory disease in infants (Nair et al. 2010). In 2005, an estimated 33.8 million episodes of RSV occurred worldwide in infants younger than 5 years old. Of these, at least 3.4 million severe cases of LRI required hospitalization, and an estimated 66,000 to 199,000 deaths occurred, mostly in the developing world (Nair et al. 2010).

There are no vaccines approved for the prevention of RSV infection. Palivizumab, a monoclonal antibody directed against RSV, is approved only for prophylaxis in order to prevent RSV-related hospitalizations (Synagis® Package Insert, April 2012) in high-risk infants.

Treatment of infants with severe RSV bronchiolitis is largely supportive, consisting of oxygen therapy, nutrition, and fluids. Aerosolized ribavirin, a synthetic guanosine analogue and broad spectrum antiviral agent, is approved for treatment of infants with severe LRIs, but its use is limited due to questionable efficacy and complexity of administration (Hall 2001; AAP 1998). There are only a few agents in development for the treatment of RSV infection. As such, there is a need for a novel therapeutic that can be used both in the outpatient setting to reduce the severity of infection and prevent possible hospital admissions, and in the hospital setting, to ameliorate the severity of symptoms and duration of time spent in the hospital.

ALS-008176 is a 3',5'-bisisobutyrate prodrug of the cytidine nucleoside analog, ALS-008112, which is being developed by Alios BioPharma as an orally administered antiviral therapy for the treatment of infants with RSV infection. Following oral administration, ALS-008176 is rapidly absorbed and efficiently converted to ALS-008112 in vivo. ALS-008112 and ALS-008176 are potent and highly selective inhibitors of both RSV laboratory-adapted A

and B strains as well as a range of diverse clinical isolates. In addition, both inhibit RSV replication in the sub-genomic replicon system with IC_{50} values of 0.15 µM and 0.26 µM, respectively. Inside cells, ALS-008112 is efficiently converted to ALS-008112-5′-triphosphate (ALS-008136 or NTP), the active metabolite of the compound, which is a potent and selective inhibitor of RSV RNA-dependent RNA polymerase activity, via a classic chain termination mechanism. High and sustained concentrations of the NTP were formed in monkey lung following single oral dose of ALS-008176, indicating efficient intracellular conversion to the active metabolite in tissues where RSV is known to replicate. The long in vitro half-life of the NTP and the observed sustained levels of the NTP in monkey lung support a dosing frequency of twice daily, or possibly once daily, oral dosing of ALS-008176 in human clinical trials.

In nonclinical juvenile rat studies with ALS-008176, high systemic exposures of ALS-008112 were achieved. Plasma exposure of ALS-008112 in juvenile rats was similar across age groups and to that of adults at all doses studied. In both adult and juvenile animals, the hematopoietic system, specifically the bone marrow and subsequent changes in hematological parameters (e.g., reticulocytes), was considered the target organ for toxicity. These effects were reversible and noted at similar ALS-008112 plasma concentrations across the age groups. Based on these results, following 14 days of dosing with ALS-008176, the no-observed-adverse-effect level (NOAEL) was determined to be 150 mg/kg twice daily or 300 mg/kg/day (ALS-008112 $AUC_{0\text{-}24\,h}$ 53,600 ng•h/mL) in adult rats; 75 mg/kg twice daily or 150 mg/kg/day (ALS-008112 $AUC_{0\text{-}24\,h}$ 192,500 ng•h/mL) in adult dogs. The NOAEL in juvenile rats dosed from PND1-28 was 150 mg/kg ALS-008176 twice daily or 300 mg/kg/day (ALS-008112 $AUC_{0\text{-}24\,h}$ 93,900 ng•h/mL), and for rats dosed from PND21-45 was 75 mg/kg ALS-008176 twice daily or 150 mg/kg/day (ALS-008112 $AUC_{0\text{-}24\,h}$ 32,950 ng•h/mL).

Neither ALS-008176 nor its metabolites are considered DNA reactive. Similar to other nucleoside analogs, including several approved drugs, ALS-008112 caused increases in structural chromosomal aberrations in human peripheral blood lymphocytes and induction of micronucleus in rat bone marrow at high concentrations. The no-observed-effect level (NOEL) for micronuclei induction was considered to be 200 mg/kg/day ALS-008176 (ALS-008112 $AUC_{0\text{-}24\,h}$ 57,595 ng•h/mL). The chromosomal aberrations and micronucleus formation were noted at high concentrations of ALS-008112, where it causes ≥50% depletion of natural nucleotide pools in cells. This suggests that genotoxicity is induced by an indirect, threshold-based mechanism of intracellular NTP pool disturbances rather than direct DNA reactivity. This mechanism of clastogenicity is well established for nucleoside analogs, many of which have been approved for chronic use in adults and children and have been prescribed for decades, and has regulatory precedence of a threshold-based risk assessment for humans.

Clinical Data (as of 5 August 2016)

Study Populations, Dosing Regimens Evaluated

As of 5 August 2016, 192 healthy volunteers have received ALS-008176 as single doses up to 3000 mg or multiple doses up to a 750 mg loading dose (LD) followed by a 500 mg maintenance dose (MD; dosed twice daily, 12 hours apart [Q12]) for a total duration of up to 14 days.

In ongoing Study ALS-8176-510, which is assessing the safety and tolerability of a 750 mg LD followed by nine 500 mg MDs of ALS-008176 or placebo in elderly subjects hospitalized with RSV infection, 6 subjects have been dosed.

In the current study (Study ALS-8176-503), 122 infants and neonates have been enrolled. Seventy (70) subjects have enrolled in the SAD portion of the study, with 24, 24, 18, and 4 subjects enrolled in the 1.37, 4.1, 12, and 25 mg/kg cohorts, respectively. Enrollment in the SAD portion of the study is now closed. In the multiple ascending dose (MAD) portion of the study, 6, 23, 14, and 9 subjects have enrolled in the 4.1/1.37, 10/2, 30/6, and 30/10 mg/kg cohorts, respectively. Enrollment by age stratum and dose level for the SAD and MAD portions of the study can be found in Figure 1-1.

In the MAD portion of the study, 6 Japanese subjects enrolled into the 4.1/1.37 dosing regimen before the IDMC instructed that Japanese subjects may enroll at the same dosing regimen as the rest of world.

Figure 1-1. Study ALS-8176-503 Enrollment Status as of 5 August 2016

» SAD

Dose (mg/kg)	0-1 mo	1-2 mos	2-6 mos	6-12 mos	TOTAL
1.37	-	8	8	8	24
4.1	-	8	8	8	24
12	-	0	9	9	18
25	-	0	3	1	4
TOTAL (%)	-	16 (23%)	28 (40%)	26 (37%)	70

» MAD

Dosing Regimen (LD/MD)	0-1 mo	1-2 mos	2-6 mos	6-12 mos	TOTAL
4.1/1.37	-	1	1	4	6
10/2	1	6	13	3	23
30/6	-	5	8	1	14
30/10	-	4	5	0	9
TOTAL (%)	1 (2%)	16 (31%)	27 (52%)	8 (15%)	52

Safety - Serious Adverse Events

In all studies conducted to date, 4 serious adverse events (SAE) have been reported, all of which occurred in the ongoing study in hospitalized infants. One event (phlebitis requiring intravenous antibiotics) occurred in this study 5 days after an infant received a single 1.37 mg/kg dose and was considered unrelated to study drug (ALS-008176 or Placebo). A second event (pneumonia with respiratory failure) occurred 2 days after an infant received a 12 mg/kg dose and was considered unrelated to study drug. The third SAE (sinus tachycardia during a renal ultrasound procedure that required observation in the intensive care unit) occurred 24 hours after receiving a 25 mg/kg dose of study drug and was considered unrelated to study drug. The fourth SAE (lymphadenitis) occurred with onset 16 days after an infant completed a 30/6 dosing regimen and became serious when the infant was hospitalized 24 days after completing treatment. The event was considered unrelated to study drug.

Safety - Adverse Events, Severities

To date, no adverse events (AEs) in any study involving ALS-008176 have led to study drug discontinuation.

All reported AEs in all studies in healthy volunteers have been mild or moderate in severity except 3 events, all of which were severe. Two of these severe events (increased alanine transaminase [ALT] and creatine kinase) occurred in Study ALS-8176-502; both events occurred in subjects dosed with ALS-008176 and neither was considered related to study drug (see discussion in Investigator's Brochure Section 6.5.1.2.2). The third severe event (increased low density lipoprotein) occurred in Study ALS-8176-511 (Japanese ethnic bridging study) 13 days after receiving a single dose of ALS-008176 and was considered possibly related to study drug. The most commonly reported AEs (i.e., ≥3% incidence in adult subjects receiving multiple doses of ALS-008176) which have been reported more often in ALS-008176-treated vs. placebo-treated healthy volunteers are: epistaxis, headache, oropharyngeal pain, and platelet count decreased. None of these events is considered suggestive of an emerging safety signal, as discussed in the current IB Section 6.5.1.2.2.

In Study ALS-8176-510, among the 6 elderly subjects enrolled as of 5 August 2016, all AEs (N=7), have been mild (N=6) or severe (N=1) in intensity. The severe event (neutropenia) occurred 23 days after the last dose of study drug and was considered by the investigator to be possibly related to study drug. The event has since resolved without sequelae.

As of 5 August 2016, subjects reported 117 AEs (SAD N=57; MAD N=60) in Study ALS-8176-503. All AEs, except the pneumonia SAE (N=1), which was considered severe, have been mild (N=95) or moderate (N=21) in severity. A blinded summary table of all treatment-emergent AEs reported as of 5 August 2016 is presented in Table 1-1 (SAD) and Table 1-2 (MAD).

Unblinded safety data from this study are regularly reviewed by an Independent Data Monitoring Committee (IDMC), which has expressed no safety concerns based on a cumulative review of safety and PK data as recently as 15 August 2016.

Table 1-1. Blinded Incidence and Severity of Treatment-emergent AEs in SAD Portion of Study ALS-8176-503 (as of 5 August2016)

AE Term	No. of Subjects	Severity (Mild, Moderate, Severe)
Thrombocytosis	8	3 Moderate, 5 Mild
Vomiting	5	Mild
Diarrhoea	4	Mild
Aspartate aminotransferase increased	3	Mild
Blood creatine phosphokinase increased	3	1 Moderate, 2 Mild
Bacterial Pneumonia	2	1 Severe*, 1 Moderate
Otitis Media	2	1 Moderate, 1 Mild
Conjunctivitis	2	Mild
White blood cell count increased	2	Mild
Rash	2	Mild
Sinus tachycardia*	2	Mild
Anaemia	1	Moderate
Blood bicarbonate decreased	1	Moderate
Eye infection bacterial	1	Moderate
Phlebitis*	1	Moderate
Abnormal faeces	1	Mild
Bradycardia	1	Mild
Candida infection	1	Mild
Constipation	1	Mild
Dermatitis diaper	1	Mild
Dry skin	1	Mild
Eczema	1	Mild
Electrocardiogram QT prolonged	1	Mild
Hepatic enzyme increased	1	Mild
Lymphocyte count increased	1	Mild
Monocyte count increased	1	Mild
Pyrexia	1	Mild
Reticulocyte count increased	1	Mild
Roseola	1	Mild
Skin lesion	1	Mild
Tachycardia	1	Mild
Ventricular extrasystoles	1	Mild
Weight decreased	1	Mild

*One adverse event was reported as a serious adverse event

Table 1-2. Blinded Incidence and Severity of Treatment-emergent AEs in MAD Portion of Study ALS-8176-503 (as of 5 August 2016)

AE Term**	No. of Subjects	Severity (Mild, Moderate, Severe)
Dermatitis diaper	7	7 Mild
Diarrhoea	6	2 Moderate, 4 Mild
Thrombocytosis	4	2 Moderate, 2 Mild
Vomiting	4	1 Moderate, 3 Mild
Aspartate aminotransferase increased	4	Mild
Eczema	4	Mild
Rash	3	1 Moderate, 2 Mild
Alanine aminotransferase increased	2	Mild
Erythema	2	Mild
Nasopharyngitis	2	Mild
Adenovirus Infection	1	Moderate
Epistaxis	1	Moderate
Cough	1	Moderate
Hyperglycaemia	1	Moderate
Lymphadenitis*	1	Moderate
Blood creatine phosphokinase increased	1	Mild
Blood potassium increased	1	Mild
Cardiac murmur	1	Mild
Conjunctivitis	1	Mild
Dermatitis contact	1	Mild
Extrasystoles	1	Mild
Eye discharge	1	Mild
Hepatocellular injury	1	Mild
Lower respiratory tract infection viral	1	Mild
Neutropenia	1	Mild
Pyrexia	1	Mild
Rash erythematous	1	Mild
Rash papular	1	Mild
Respiratory tract infection	1	Mild
Seborrhoeic dermatitis	1	Mild
Upper respiratory tract infection	1	Mild
White blood cell count increased	1	Mild

*One adverse event was reported as a serious adverse event.

Safety – Laboratories, ECGs, Vital Signs, and Physical Examinations

In all studies of ALS-008176 in healthy volunteers and naturally infected elderly subjects and infants, no clinically significant laboratory, electrocardiogram (ECG), vital sign, or physical examination findings suggestive of a safety concern have been identified.

Clinical Data - Efficacy

The efficacy of ALS-008176 in naturally infected patient populations has not been defined; however, it has been assessed in healthy volunteers infected with RSV in a human challenge model (Study ALS-8176-502). In this study, in 62 healthy volunteers inoculated with RSV and dosed with ALS-008176 or placebo, preliminary data demonstrate that maintenance doses of 150 to 500 mg of ALS-008176, particularly following a 750 mg LD, resulted in rapid, substantial declines in RSV viral load with an accompanying comparable improvement in signs and symptoms of RSV infection compared to placebo-treated subjects. The effect of ALS-008176 on RSV viral load in the challenge model can be found in Figure 1-2. There was an accompanying decrease in RSV signs and symptoms that corresponded to these reductions in viral load (Investigator's Brochure Section 6.3).

Figure 1-2. RSV Viral Load Over Time Following Administration of ALS-008176 or Placebo for 5 Days in the Intent-to-Treat-infected Population in Study ALS-8176-502

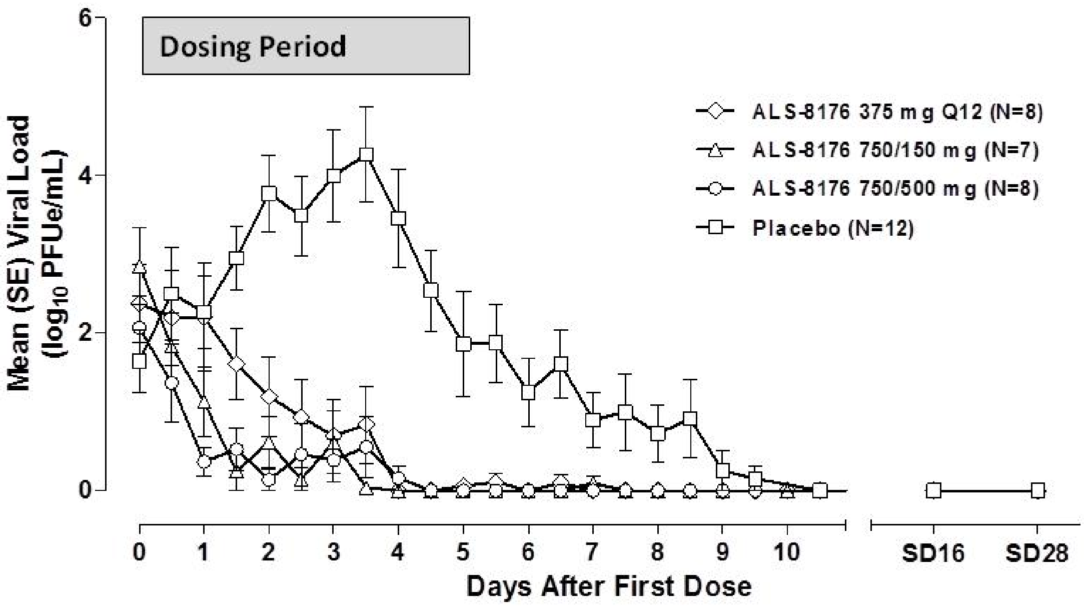

PFUe: plaque-forming unit equivalents; Q12: every 12 hours; SD: Study Day

Clinical Data – Pharmacokinetics

For a detailed discussion of the pharmacokinetics of ALS-008176 and its metabolites in healthy volunteers and infants, please refer to Section 6.2 of the current Investigator's Brochure (v6).

In Study ALS-8176-503, ALS-008176 is rapidly converted to ALS-008112. A population pharmacokinetic (popPK) model has been created to characterize infant pharmacokinetics. Currently available preliminary infant PK data indicate that the observed blood exposures for the parent nucleoside, ALS-008112, are within those predicted by the popPK model for single doses at the 1.37, 4.1, 12 and 25 mg/kg dose levels and are generally dose proportional. Additionally, for a given dose, no significant age-related differences in ALS-008112, ALS-008144 and ALS-008206 exposures have been observed.

Preliminary PK data after multiple doses are also available for the cohorts which evaluated 4.1/1.37, 10/2, 30/6, and 30/10 (Figure 1-3) dosing regimens. These data suggest:

- Multiple-dose PK is similar to that projected based on the popPK model for ALS-008112
- No evidence of clinically important accumulation is present for either ALS-008112 or ALS-008144 over 5 days of dosing; some accumulation of ALS-008144 with multiple dosing may be observed, but requires confirmation in a larger number of patients
- There are no apparent ethnic differences in pharmacokinetics identified between Japanese and non-Japanese subjects in the subjects evaluated to date
- No age related PK differences have been observed

Figure 1-3. ALS-008112 Exposures in Infants 1-12 Months of Age Receiving 30 mg/kg LD and 10 mg/kg MD Regimen in Study ALS-8176-503

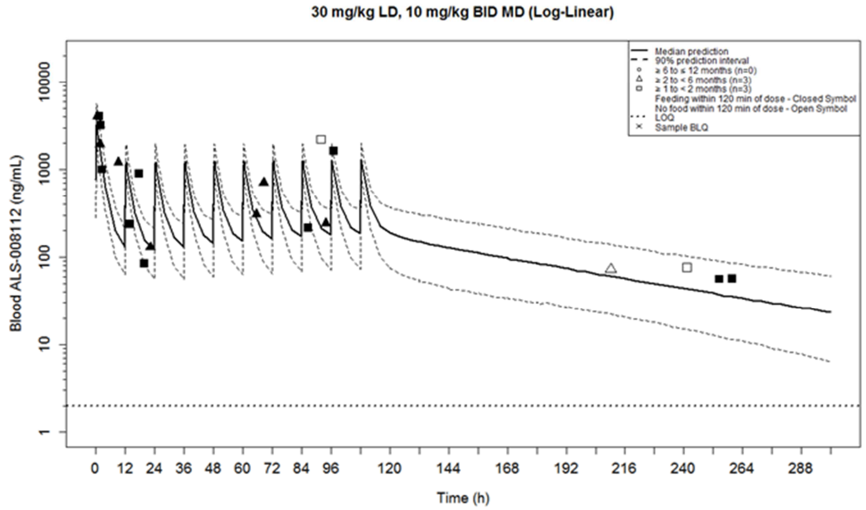

1.2 RATIONALE FOR THE STUDY

This study is being performed to determine preliminary safety, tolerability, PK, and PD data after single and multiple doses of ALS-008176 given to infants ≥1.0 to ≤12.0 months of age and neonates who are hospitalized with RSV infection. The study is being amended (v6) to allow for additional subjects to be enrolled in order to continue to define the safety, pharmacokinetics, and viral kinetics of ALS-008176 and its metabolites throughout the previously defined permissible exposures range (i.e., mean projected plasma ALS-008112 $AUC_{0\text{-}24h}$ up to 20,000 ng•h/mL). An additional 100 subjects may be needed to fully characterize this exposure range depending on the rate of dose escalation, as defined by the IDMC, and in the event the Sponsor is also authorized by IDMC to explore additional dosing regimens (e.g., once daily).

1.3 RATIONALE FOR STARTING DOSE SELECTION

For estimation of starting doses, an integrated approach was utilized. Literature regarding similar approved nucleoside analogs for pediatrics was reviewed. Adult plasma and urinary PK data from Study ALS-8176-501 were used to build a robust pediatric population pharmacokinetic model. For predicting plasma exposures in infants, the adult PK model was used to establish a pediatric PK model. By integrating allometric scaling of the known physiological differences in maturation of a 1-month-old to 2-year-old infant, the plasma exposures of ALS-008112 and ALS-008144 across the age groups proposed in the protocol were predicted for a variety of dosing regimens. In order to estimate therapeutic doses and/or plasma exposures, the pediatric PK model was coupled with a semi-mechanistic pediatric lung model which estimated the formation of the active 5’-triphosphate of ALS--008112 (ALS-008136 or NTP) in lung at a given ALS-008112 plasma exposure. The efficacious dose

projections were used to justify the starting dose such that subtherapeutic concentrations are avoided in infants and neonates. A list of parameters used for dose estimations are listed in Table 1-3. The highest proposed doses were estimated based on the maximum plasma exposure and safety data obtained in adults, the nonclinical NOELs/thresholds and the predicted variability from the pediatric PK model. Considering that RSV is an acute disease, and rapid therapeutic levels of NTP are needed in target tissues to stop viral replication, a single loading dose followed by maintenance dose strategy will be employed in the MAD phase and future therapeutic trials. Due to the inherent limitations regarding intensive PK sampling in infants and neonates, a population PK approach with optimal PK sampling calculations will be utilized.

The proposed starting dose in infants takes into consideration the following:

- The maximum human plasma exposures obtained in adults, and the lack of any significant safety findings in adults following doses of 750 mg Q12 on Day 1 followed by 500 mg Q12 for up to 13 days
- Projected human efficacious plasma exposures of ALS-008112 needed to maintain C_{min} NTP concentrations in target tissues
- Pediatric PK projections and variability within the model
- Reversible and monitorable nonclinical toxicology profile of ALS-008112 and margins from the nonclinical safety studies, specifically to limit the average projected plasma exposure of ALS-008112 AUC_{0-24} to <20,000 ng•h/mL. (Note: All references to average plasma exposures in infants refer to the geometric mean exposure.)

Table 1-3. Parameters Used to Determine Projected Efficacious Doses in Infants*

Parameter	Value
K_i for RSV polymerase inhibition	0.09 µM
Concentration of NTP at antiviral IC_{50}	0.23 µM
Concentration of NTP at antiviral IC_{90}	3 µM
Infant equivalent dose to achieve lung NTP of ≥1 × IC_{50} or 0.23 µM at 24 hours postdose (pediatric PK and semi-mechanistic modeling)	1.5 mg/kg
Infant equivalent LD and MD to maintain lung NTP of 1 × IC_{90} or 3 µM (pediatric PK and semi-mechanistic modeling)	10 mg/kg, followed by 2 mg/kg, twice daily
Infant equivalent LD and MD to maintain lung C_{min} NTP levels 3 × IC_{90} or 9 µM or higher (pediatric PK and semi-mechanistic lung modeling)	10 mg/kg followed by 6 mg/kg, twice daily

C_{min}: minimum concentration; IC_{50}/IC_{90}: concentration resulting in 50%/90% effectiveness; K_i: inhibition constant: LD: loading dose; MD: maintenance dose; NTP: nucleotide triphosphate ALS-008136.
*Initial projections before availability of human challenge (ALS-8176-502) and infant (ALS-8176-503) data

1.3.1 Single Ascending Dose (SAD):

Based on pediatric PK modelling and an estimation of human efficacious doses, a starting dose of 1.5 mg/kg ALS-008176 was selected for this study. This dose in infants was projected to maintain ALS-008112 exposures significantly below exposures obtained with a 750 mg dose in adults that was well tolerated with no significant AEs. Although population PK modeling indicates that there will be minimal influence of age or maturation factors on the plasma exposure of ALS-008112 or ALS-008144, the proposed dose is low enough as not to exceed the limits of exposure. In addition, this dose is the lowest projected therapeutic dose thereby minimizing the risk of exposing participating RSV-infected infants to subtherapeutic levels of ALS-008112. At this dose, the average C_{min} NTP levels (defined as the NTP concentration at 24 hours after the single dose) are projected to be at least above the IC_{50} NTP levels needed for antiviral activity.

The proposed dose escalation allows for understanding of dose and age related changes in pharmacokinetics. Based on population PK modeling, the highest dose of 25 mg/kg is projected to have a median plasma ALS-008112 $AUC_{0\text{-}24h}$ of approximately 9,001–10,038 ng•h/mL; exposures that are similar to those which have been studied in adults. For ALS-008144, plasma exposures are also projected to be well within those obtained in adults.

1.3.2 Multiple Ascending Dose

Doses for the MAD phase are designed to rapidly achieve and maintain a sustained level of lung NTP C_{min} over the course of treatment. A loading dose is required to rapidly achieve these NTP levels followed by maintenance doses (twice daily) which will maintain the constant levels of NTP that are required to inhibit viral replication. The planned dosing regimens span the projected therapeutic dose ranges.

For Cohort 1, a single loading dose of 10 mg/kg followed by 2 mg/kg twice daily as maintenance doses is projected to achieve lung NTP levels within 2 hours that are needed for EC_{99} antiviral activity. The maximum possible dose for Cohort 2, 30 mg/kg LD followed by a 6 mg/kg MD is projected to achieve >3× EC_{99} within 2 hours and maintain C_{min} >3× EC_{99} throughout the dosing cycle. Based on observed differences in pharmacokinetics between Japanese and non-Japanese subjects, the initial Japanese infant regimen of 4.1 mg/kg LD followed by a 1.37 mg/kg MD was estimated to approximate the exposures of the 10 mg/kg LD followed by 2 mg/kg MD in (non-Japanese) MAD Cohort 1.

The geometric mean plasma ALS-008112 $AUC_{0\text{-}24h}$ for the 30/6 mg/kg dosing regimen in MAD Cohort 2 is estimated to be approximately 11,538 ng•h/mL on Day 1 and 6,025 ng•h/mL on Day 5. These exposures are similar to (at Day 1) and approximately half (Day 5) of the exposures that were found to be efficacious in adult healthy volunteers in Study ALS-8176-502 (Challenge Study). The 30/10 mg/kg dosing regimen is projected to deliver a geometric mean Day 1 and Day 5 ALS-008112 $AUC_{0\text{-}24h}$ plasma exposure of 12,683 and 9579 ng•h/mL, respectively. Based on the pediatric population PK model, the highest

dosing regimen which is projected to deliver an average ALS-008112$_{0\text{-}24h}$ AUC <20,000 ng•h/mL is a 40/20 mg/kg regimen, which is projected to have geometric mean Day 1 and Day 5 exposures of 19,062 ng•h/mL and 18,929 ng•h/mL, respectively. These exposures maintain a safety margin of >2.7-fold relative to the lowest NOAEL of the most sensitive toxicology species.

For neonates, the planned dose is 10 mg/kg LD followed by 2 mg/kg maintenance dose MAD regimen because, to date, this regimen has been well tolerated in infants 1-12 months of age. Furthermore, given that no age dependent differences in pharmacokinetics have been observed in 1-12 month olds, this dosing regimen is projected to deliver similar exposures in neonates as 1-12 month olds. The actual dose for the neonate cohort(s) will be determined by the IDMC based on emerging PK and safety ± viral kinetics data from 1-12 month old cohorts and cannot exceed the doses, regimens and durations that are under evaluation in ≥1-≤12 month old subjects at that time.

2.0 STUDY DESIGN

2.1 SUMMARY

This randomized, double-blind, placebo-controlled, 2-part study will assess the safety, tolerability, PK, and PD of single and multiple doses of orally administered ALS-008176 in infants hospitalized with RSV infection.

The most current preliminary dose escalation schema is presented in Figure 2-1. Study procedures are described in Table 6-1, *Schedule of Events SAD Phase (Part 1)*, Table 6-2: *Schedule of Events MAD Phase (Part 2) Twice-Daily Dosing* and Table 6-3. *Schedule of Events: MAD Phase (Part 2) Once-Daily Dosing*.

Figure 2-1. Preliminary Dose Escalation Schema

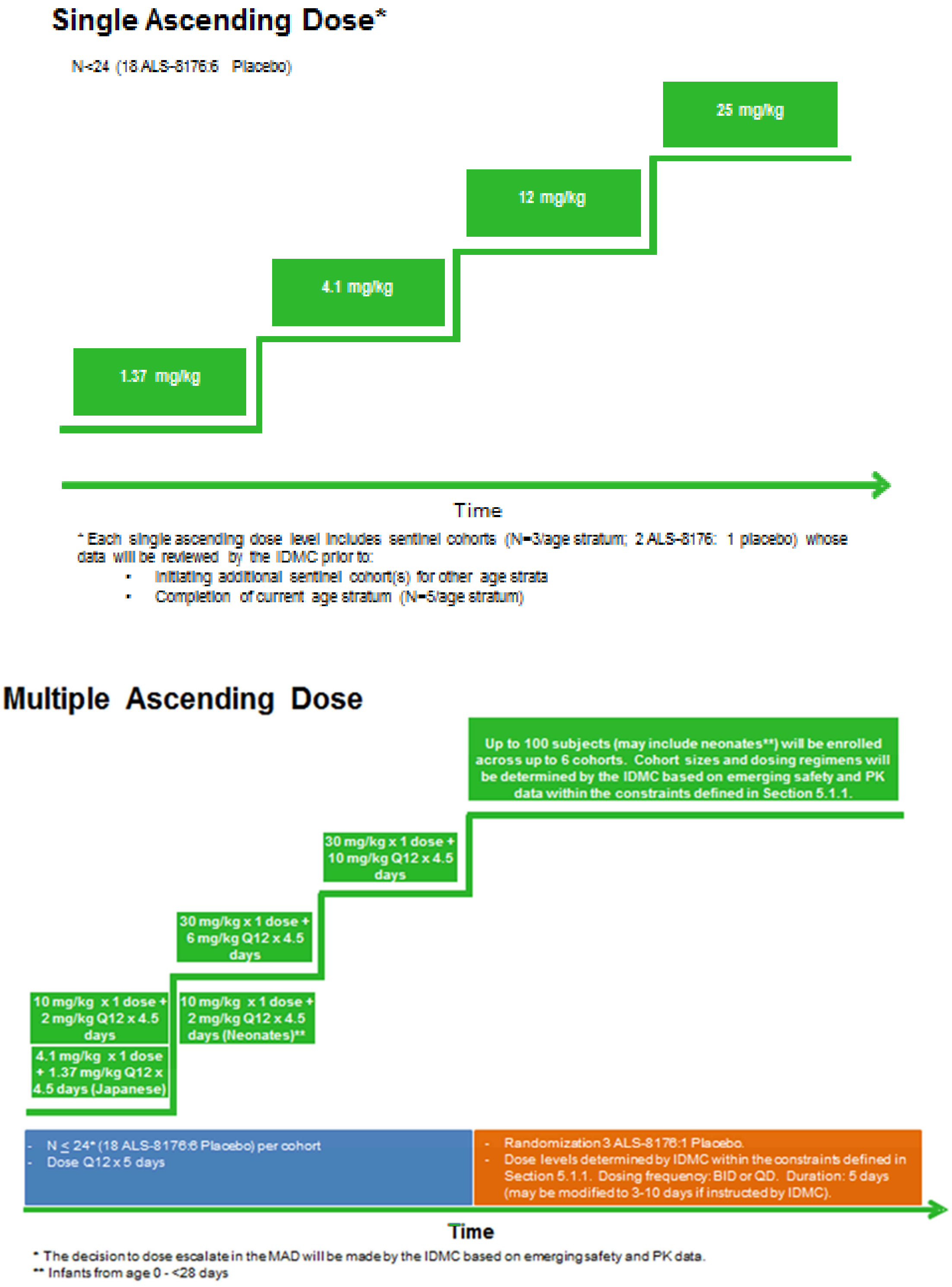

2.2 PART 1: SINGLE ASCENDING DOSE (SAD)

Subjects will receive a single dose from 1 of 3 ascending dose levels of ALS-008176.

Within each dosing cohort, subjects will be randomized to receive either ALS-008176 or placebo (n=approximately 24 per cohort; randomized in a ratio of 3 ALS-008176:1 placebo). Randomization will be stratified by age at time of hospital admission (≥1.0 to <2.0 months =28 to 59 days old, ≥2.0 to <6.0 months =60 to 181 days old, and ≥6.0 to ≤12.0 months =182 to 365 days old), with approximately 8 subjects being enrolled in each stratum. Additionally, all subjects will receive standard supportive care.

An age de-escalation approach will be utilized within each SAD dose cohort, i.e., subjects aged ≥6.0 to ≤12.0 months will be enrolled initially, followed by subjects ≥2.0 to <6.0 months, and then subjects ≥1.0 to <2.0 months, as follows:

- A sentinel group of 3 subjects in each age stratum will be enrolled first (randomized in a ratio of 2 ALS-008176:1 placebo).
- The IDMC will review the safety data through Day 7 and available PK data for the sentinel subjects. Following this:
  - The remaining 5 subjects in the age stratum will be enrolled (randomized in a ratio of 4 ALS-008176:1 placebo), and
  - Enrollment in the next age stratum’s sentinel cohort(s) will be initiated.

At no time will dose escalation occur for a younger age stratum before dosing in an older age stratum has been initiated for that dose, unless the IDMC determined that the safety profile supported such an approach.

Subjects will be evaluated over a 7-day period from the time of randomization. If they are discharged from the hospital prior to Day 7, they will be required to return as an outpatient for assessment on Day 7, when they will complete the study.

Dose escalation within Part 1 will not occur before the IDMC has reviewed and deemed acceptable:

- all safety data through study completion for all subjects in the current cohort or age stratum
- available PK data from the first 24 hours after dosing for at least 75% of the current cohorts’ subjects (or a completed stratum within this cohort)

As of 25 April 2016, the SAD portion of the study has been completed and is now closed.

A fourth, fifth and sixth cohort may be evaluated at the discretion of the Sponsor, upon approval by the IDMC, based on an evaluation of the emerging PK profile and safety profile.

The planned dose escalation schema may be modified by the Sponsor, upon approval by the IDMC, based on emerging PK and safety data. In all circumstances, the dosing schema in Part 1 will have no more than a 3-fold increase in dose between dose levels (as outlined in Section 5.1.1, *Cohort Progression Guidelines)*. In addition, under no circumstances will a potential dose be expected to exceed a projected average ALS-008112 $AUC_{0\text{-}24h}$ of 20,000 ng•h/mL. Throughout the conduct of the study, the pharmacokinetics of ALS-008112 and ALS-008144 will be evaluated and reviewed. An established pediatric PK model will be updated with the additional data, and the plasma exposures of the subsequent dose will be simulated prior to dose escalation, as a safety check to ensure that the pharmacokinetics of ALS-008112 are not predicted to differ significantly from the intended exposures.

See also Section 5.1.1, *Cohort Progression Guidelines*.

2.3 PART 2: MULTIPLE ASCENDING DOSE (MAD)

Part 2 will commence when emerging PK and safety data from Part 1 have been evaluated and are deemed by the IDMC to be acceptable and sufficient to initiate multiple dosing.

Based on the lack of clinically relevant PK differences observed to date across the 3 age strata, infants of all 3 age strata will receive the same doses, unless otherwise instructed by the IDMC. Similarly, doses will be escalated in parallel in all 3 age strata for the second and subsequent MAD Cohorts if the IDMC deems available PK and safety data in the current MAD cohort to be sufficient and authorizes dose escalation.

Hospitalized subjects with RSV infection will be assigned to 1 of up to 11 planned MAD regimens of ALS-008176 or placebo (see Figure 2-1). The number of dosing regimens and doses to be studied will be at the discretion of the Sponsor on the basis of the emerging PK and safety profiles, upon approval by the IDMC.

One or more cohorts will enroll (except when cohort enrollment is paused) neonates (<28 days old) if supported by emerging PK, safety, and efficacy data in infants 1-12 months of age. The dose regimen(s) studied in neonates may be adjusted according to IDMC instructions.

Japanese infants will also be enrolled in Part 2 (see Section 1.1). Initially, Japanese patients received a 4.1 mg/kg loading dose followed by nine 1.37 mg/kg maintenance doses (4.1/1.37). The IDMC reviewed emerging safety and PK data and due to lack of differences in exposures in Japanese vs. non-Japanese subjects in this study, Japanese infants are now eligible for inclusion in all cohorts and there is no restriction on how many Japanese infants may be enrolled.

Within each dosing cohort, subjects will be randomized to receive either ALS-008176 or placebo in a ratio of 3 ALS-008176: 1 placebo. Randomization (except in the neonate cohort) will be stratified by age at time of hospital admission (≥1.0 to <2.0 months =28 to 59 days

old, ≥2.0 to <6.0 months =60 to 181 days old, and ≥6.0 to ≤12.0 months =182 to 365 days old). Additionally, all subjects will receive standard supportive care as per local institution. Subjects will be evaluated over a 28-day period from the time of randomization. If they are discharged from the hospital prior to Day 28, they will be required to return for assessment as an outpatient on Day 5, if applicable, and on Day 11 and 28, when they will complete the study, assuming a 5-day treatment duration (see Schedule of Events for timing of visits for other treatment durations).

Up to 6 additional cohorts may be enrolled with a maximum of 100 additional subjects (including neonates) in these cohorts. Additionally, the following elements of the study may be adjusted subject to approval by the IDMC (see also Sections 5.1.1 and 7.5):

- The size of each cohort and the frequency at which cohort data are reviewed. Enrollment will generally be allowed to continue during preparation and review of data by IDMC, unless otherwise instructed by the IDMC.
- Dosing frequency within a cohort may be once daily or twice daily
- The maintenance doses may be the same as the loading dose
- The duration of dosing within a cohort will be 5 days unless otherwise instructed by the IDMC, which may modify the duration within the range of 3 to 10 days, inclusive
- The duration of symptoms from first onset until randomization will be ≤5 days unless otherwise instructed by the IDMC, which may modify the duration to up to 7 days

Subjects will receive a total of 10 doses administered over 5 days if dosed twice daily or a total of 5 doses administered over 5 days if dosed once daily, assuming a 5-day treatment duration.

Regardless of dosing frequency, the first dose of study drug should be administered to the subject as soon as possible after randomization and not timed relative to regular hospital dosing times.

In each twice-daily dosing regimen, the loading dose (Dose 1) will be followed by a twice daily maintenance dose regimen (Doses 2–10; Figure 2-1). The first maintenance dose (Dose 2) will be given between 8-18 hours after the loading dose (Dose 1) to facilitate getting the subject on an established hospital dosing regimen. All subsequent doses will be given per the hospital's regular twice daily dosing times ±1 hour.

In once daily dosing regimens, the second dose will be given between 21-27 hours after Dose 1 to facilitate getting the subject on an established hospital dosing regimen. All subsequent doses will be given per the hospital's once daily dosing times ±1 hour.

In both Parts 1 and 2, the planned dose escalation schema (Figure 2-1) may be modified by the Sponsor, upon approval by the IDMC, based on emerging PK and safety data. In all

circumstances, however, there will be no more than a 3-fold increase in dose between dose levels (as outlined in Section 5.1.1, *Cohort Progression Guidelines*).

Throughout the conduct of the study, the pharmacokinetics of ALS-008112 and ALS-008144 will be evaluated and reviewed. A pediatric population PK model will be updated with the additional data, and the plasma exposures of the subsequent dose will be simulated prior to dose escalation as a safety check to ensure that the pharmacokinetics of ALS-008112 are not predicted to differ significantly from the intended exposures. Alterations in the planned dose escalation scheme will be made as necessary. In addition, under no circumstances will a planned pediatric dosing regimen exceed a projected average ALS-008112 $AUC_{0\text{-}24h}$ of 20,000 ng•h/mL. Furthermore, the decision to dose escalate between cohorts will be based on a review of safety and PK data by the Sponsor and IDMC.

The IDMC will review unblinded safety data on a regular basis and approve each dose escalation decision.

Subject safety will be monitored by regular assessment of the results of clinical laboratory tests, ECGs, physical examination, vital signs, and AE assessments as reported by parents/legal guardians/medical staff.

Clinical course of RSV infection will be assessed by quantitative polymerase chain reaction (PCR) measurement of RSV RNA viral load.

3.0 STUDY OBJECTIVES AND ENDPOINTS

3.1 STUDY OBJECTIVES

In neonates (<28 days old) and infants (≥1.0 to ≤12.0 months of age) who are hospitalized with RSV infection:

3.1.1 Primary Objective

- To evaluate the safety and tolerability of single and multiple doses of ALS-008176

3.1.2 Secondary Objectives

- To evaluate the pharmacokinetics of ALS-008112 and ALS-008144 (and other metabolites, if applicable) in blood following single and multiple doses of ALS-008176 To evaluate the antiviral activity of ALS-008176 after single and multiple doses of ALS-008176
- To determine if ALS-008176 exposure results in the emergence of resistant strains of RSV

3.1.3 Exploratory Objectives

- To evaluate the impact of ALS-008176 on the clinical course of RSV infection

- To evaluate the relationship between viral kinetics and clinical outcomes
- To evaluate the impact of ALS-008176 on biomarkers potentially associated with the inflammatory response induced by acute RSV infection

To evaluate the pharmacokinetics of ALS-008112 and ALS-008144 (and other metabolites, if applicable) in nasal swabs following multiple doses of ALS-008176, if an acceptable testing methodology can be established.

3.2 STUDY ENDPOINTS

3.2.1 Primary Endpoint

- Safety data including, but not limited to, adverse events, physical examinations, vital signs, 12-lead ECGs and clinical laboratory results (including chemistry and hematology)

3.2.2 Secondary Endpoints

- PK parameters of ALS-008112 and ALS-008144 (and other metabolites, as applicable) in blood following single dose administration: C_{max}, t_{max}, $t_{1/2}$, $AUC_{0\text{-}24h}$, $AUC_{0\text{-}inf}$ or $AUC_{0\text{-}last}$
- PK parameters of ALS-008112 and ALS-008144 (and other metabolites as applicable) in blood following repeat dose administration: C_{max}, C_{min}, t_{max}, $t_{1/2}$, $AUC_{0\text{-}12h}$, $AUC_{0\text{-}24h}$, $AUC_{0\text{-}tau,}$ $AUC_{0\text{-}inf}$ or $AUC_{0\text{-}last}$
- RSV viral RNA concentrations in nasal swabs or aspirates as measured by quantitative RT-PCR
- Changes in the RSV polymerase that result in reduced sensitivity to ALS-008112

3.2.3 Exploratory Endpoints

- Length of hospital stay
- Need and duration of supplemental oxygen requirement
- Need and duration of ICU stay
- Need and duration of mechanical ventilation
- Need and duration of non-invasive ventilator support
- Time to resolution of RSV signs or symptoms, such as runny nose, wheeze, cough, tachypnea
- Relationship between viral kinetics and various clinical outcome measures (e.g., the relationship between RSV RNA viral load and oxygen supplementation, duration of hospitalization)

- Biomarkers potentially associated with the inflammatory response induced by acute RSV infection
- Concentrations of ALS-008112 and ALS-008144 (and other metabolites as applicable) in nasal secretions, if an acceptable testing methodology can be established

4.0 SELECTION AND WITHDRAWAL OF SUBJECTS

4.1 STUDY POPULATION

Part 1 (SAD): Up to 72 subjects were to enroll in SAD cohorts. As of 25 April 2016, 70 subjects had enrolled in the SAD portion of the study, which is now closed.

Part 2 (MAD): As of 5 August 2016, 52 subjects have enrolled across 5 MAD cohorts, some of which (e.g., 30/10) are actively recruiting (see Figure 2-1). Up to an additional 100 subjects will enroll in up to 6 additional cohorts. The maximum anticipated enrollment in the MAD portion of the study, taking into account these numbers as well as anticipated enrollment in ongoing cohorts during the protocol version 6 review process, is 190 subjects (52 enrolled subjects + up to 23 additional neonates + up to 15 additional subjects at 30/10 dose level + 100 additional subjects in future cohorts).

Due to lack of ethnic differences in exposures in this study, Japanese infants are now assigned to the same dose levels as infants in the rest of the world. There is no restriction on how many Japanese infants can be enrolled.

One or more cohorts will enroll (except when cohort enrollment is paused) neonates (<28 days old) if supported by emerging PK, safety, and efficacy data in infants 1–12 months of age. The dose regimen(s) studied in neonates may be adjusted according to IDMC instructions.

4.2 INCLUSION CRITERIA

Subjects must meet all of the following criteria to be included in the study:

1. Subject's parent(s)/legal guardian(s) has provided signed and dated informed consent and authorization to use protected health information, as required by national and local regulations.

2. In the investigator's opinion, the subject's parent(s)/legal guardian(s) understands and is able to comply with protocol requirements, instructions, and protocol-stated restrictions, and is likely to complete the study as planned.

3. Male or female infant who

   - is ≥1.0 to ≤12.0 months of age (inclusive), defined at the time of hospital admission,

or <28 days of age (neonate cohort only). Note: all subjects, including neonates, must have been discharged from the hospital after birth and are now being admitted due to an RSV related illness

- has been diagnosed with RSV infection based on study-supplied BINAX NOW RSV test or an RSV PCR or any other RSV assay conducted at the clinical trial site. NOTE: A subject remains eligible if any RSV result is positive. (RSV-specific PCR run locally is strongly encouraged, but not required.) Coinfection with other respiratory viruses or bacterial coinfection in addition to RSV infection is permissible.
- has been hospitalized for <96 hours (at time of randomization) for confirmed RSV infection (NOTE: nosocomial RSV infection is excluded)
- has had symptoms consistent with RSV infection (e.g., runny nose, cough, sneezing, fever, or tachypnea) for ≤ 5 days at the time of randomization (unless otherwise instructed by the IDMC, which may modify the duration to up to 7 days)

4. With the exception of the RSV-related illness, the subject is in otherwise good health as deemed by the investigator, based on the findings of a medical evaluation including medical history, physical examination, laboratory tests, and ECG
5. Creatinine clearance is not below the lower limit of normal for the subject's age (Schwartz equation calculation preferred, however alternative equations may be utilized to determine eligibility if deemed acceptable by the investigator and medical monitor)

4.3 EXCLUSION CRITERIA

Subjects will be ineligible for this study if they meet **any one** of the following criteria:

1. History of or concurrent clinically significant medical illness (not directly attributable to the acute RSV infection) – including, but not limited to cardiovascular, respiratory, renal, gastrointestinal, hematologic, neurologic, endocrinologic, immunologic, musculoskeletal, oncologic, or congenital disorders, as judged by the investigator. Specifically excluded conditions include but are not limited to:
   a. Immunosuppressed state
   b. Bronchopulmonary dysplasia
   c. Congenital heart disease
   d. Down's syndrome
2. Prematurity, defined as gestational age <37 weeks at birth
3. Subjects receiving invasive endotracheal mechanical ventilation

4. Subjects who are thought to have a poorly functioning gastrointestinal tract (i.e., unable to absorb drugs or nutrition via enteral route). NOTE: The use of intravenous fluids is not exclusionary so long as the investigator believes the patient's gastrointestinal tract still functions properly (i.e., is able to absorb drugs or nutrition).

5. Subjects with clinically significant laboratory abnormalities which are deemed by the investigator to represent a safety risk to participation in this study. Other laboratory parameters outside the reference range for the subject's age may be included if the investigator considers the abnormalities unlikely to introduce additional risk factors and will not interfere with data interpretation. A single repeat laboratory evaluation (under appropriate conditions, e.g., not hemolyzed) is allowed for eligibility determination.

6. Any condition that, in the opinion of the investigator, would compromise the study or the well-being of the subject or prevent the subject from meeting the study requirements

7. Clinically significant abnormal ECG findings, as judged by the investigator or qualified designee

8. Subjects anticipated to be discharged from the hospital in <24 hours from the time of randomization

9. Exclusionary medications include:

    a. Herbal supplements which have evidence of adversely affecting absorption and clearance mechanisms (e.g., strong inhibitors ofOAT3) within 21 days prior to randomization

    b. The following prescription medications:

        - Use of systemic medications (either chronically (i.e., >14 days for neonates and infants <2 months old, or >28 days for subjects ≥2-≤12 months of age) or within the 21 days prior to randomization) which are known to modulate the host immune response and/or increase viral shedding such as corticosteroids or other immunomodulatory therapies. The only exception is systemic corticosteroids will be acceptable if they are not taken chronically for a non-RSV-related indication.

        - Prescription medications used within 14 days prior to randomization to treat the RSV infection itself (e.g., ribavirin, intravenous immunoglobulin). Prescription medications intended to treat the symptoms/sequelae of the RSV infection are permitted.

        - Prescription medications which are known to be strong inhibitors of the OAT3 transporter, within 21 days prior to randomization (see *Prohibited Medications*, Section 5.8)

c. Investigational drug trial medications within 30 days or 5 half-lives (whichever is longer) prior to randomization
d. Prior exposure to an investigational vaccine
e. MMR vaccine within 1 week prior to screening, according to parent report
f. Prior exposure to ALS-008176
g. Prior exposure to palivizumab or other RSV prophylactic medication (approved or investigational)

10. Infants who are breastfeeding and their mother is taking any of the exclusionary medications described in exclusion criterion 9.
11. Infants with another child in the household who has enrolled in the study
12. Infants with known fructose intolerance (due to sorbitol in study medication)

4.4 SUBJECT SCREENING AND ENROLLMENT

Screening procedures will occur when a subject's parent(s)/guardian(s) signs and dates an Independent Ethics Committee-approved informed consent form and provides authorization to use protected health information. The informed consent form will be completed prior to any study-specific procedures.

All screening procedures will generally need to be completed within one 96 hour period. Procedures that are standard of care and performed within 96 hours prior to randomization may be used in determining protocol eligibility.

The investigator will verify subject eligibility according to all inclusion and exclusion criteria (Sections 4.2 and 4.3) and randomize the subject using an Interactive Response Technology (IRT) system (see Reference Binder). Randomization will assign the subject to a dose cohort, study drug or placebo, and PK sample collection schedule prior to dosing. Enrollment is defined by the confirmed randomization.

Subjects must receive the first dose of study drug within 18 hours after randomization.

The IRT system will control the number of subjects enrolled in each age group in each dose cohort. Cohort sizes and dosing regimens will be determined by the IDMC based on emerging safety and PK data within the constraints defined in Section 5.1.1.

If multiple potential subjects are being screened on the same day for the final position in a cohort, over-enrollment of the cohort will be allowed, up to a maximum of 4 extra subjects per cohort. Any additional subjects enrolled will be randomized according to the same randomization scheme.

4.5 SUBJECT WITHDRAWAL

Parents are free to withdraw their consent for their child's participation at any time during this clinical trial, with or without a stated reason. Investigative site personnel should encourage appropriate procedures to ensure safety of a withdrawn subject.

4.6 SUBJECT DISCONTINUATION

Parents are free to discontinue their child's participation at any time during this clinical trial. Additionally, the investigator has the right to remove any subject from treatment with study drug or participation in the study. However, Alios requests that the investigator make reasonable efforts to consult with the medical monitor before removing a subject.

If study drug dosing is discontinued early, every effort should be made for the subject to continue all study assessments to the completion of the study. Subjects who are discontinued from the study early, or subjects who meet the discontinuation criteria specified below, should undergo early discontinuation procedures (Section 4.6.2). Subjects discontinued for safety reasons will not be replaced. Subjects who discontinue study drug for a reason other than an adverse event may be replaced at the discretion of the Alios medical monitor.

4.6.1 Subject Discontinuation Criteria

The primary consideration in any determination to discontinue a subject's participation must be the health and welfare of the subject. Reasons for subject discontinuation may include, but are not limited, to the following:

1) Need for endotracheal mechanical ventilation
2) An adverse or serious adverse event (AE/SAE), drug reaction or complication, whether related or not to study drug, which precludes continuation of treatment with study drug. This includes the development of allergic reactions or other potentially serious drug reactions to the study medication.
3) An adverse event which is considered clinically significant (e.g., meeting Grade 3 (severe) or 4 (potentially life threatening) criteria (see Appendix C) and is attributed to study drug and/or which is thought to preclude further safe administration of study drug.
4) Noncompliance with study drug dosing.
5) Noncompliance with study procedures.
6) Lost to follow-up.
7) The principal investigator's opinion that it is not in the subject's best interest to continue study participation.
8) Sponsor's decision to terminate the study.

4.6.2 Procedures for Subjects Who Discontinue

Subjects who discontinue from treatment should undergo the Study Completion Visit evaluations tabulated in the Schedule of Events for the purpose of safety monitoring within 6 days after their last dose of study medication. If a subject is unable to complete the Completion Visit evaluations within 6 days after their last dose, a telephone visit should be completed within 6 days of discontinuation of study drug and the Completion Visit should be completed as soon as practical after the telephone visit. Any subject that discontinues with ongoing AEs should be followed until resolution of the AE(s) or until the PI has determined that the AE(s) has stabilized.

4.6.3 Documentation of Discontinuation of Subjects

Document the reasons for early discontinuation of any subject from the study on the appropriate case report form (CRF). If the reason for early discontinuation is an AE or an abnormal laboratory value, record the specific event or test result on the AE CRF, and monitor the subject until the event is resolved or deemed stable by the investigator.

4.7 STUDY DISCONTINUATION

The Sponsor has the right to terminate this study or remove a participating site at any time. Reasons for terminating the study or site may include, but are not limited to, the following:

1) The incidence or severity of AEs in this or other studies indicates a potential health hazard to subjects.
2) Subject enrollment is unsatisfactory.
3) Data recording is inaccurate or incomplete.
4) Investigator does not adhere to the protocol or applicable regulatory guidelines in conducting the study.
5) A recommendation by the IDMC to terminate the study.
6) A decision from the Independent Ethics Committee (IEC) or regulatory authority to terminate the study.

5.0 TREATMENT OF SUBJECTS

5.1 TREATMENT REGIMENS

Subjects must receive the first dose of study drug within 18 hours after randomization. Doses of ALS-008176 will be administered orally in a suspension on a weight basis. Most subjects' dose volumes will be less than 5 mL; however the maximum dose volume is not expected to exceed 10.5 mL.

The planned treatment regimens are shown in Table 5-1, Table 5-2, and in Figure 2-1.

Table 5-1. Part 1 SAD Dosing Regimen

Cohort No.	Study Drug Dose Level and Frequency
1	24 subjects (3 ALS-008176:1 placebo)- 18 subjects to receive single oral 1.37 mg/kg dose of ALS-008176 with 6 subjects receiving placebo
2	Up to 24 subjects* (3 ALS-008176:1 placebo)- 18 subjects to receive single oral 4.1 mg/kg dose of ALS-008176 with 6 subjects receiving placebo
3	Up to 24 subjects* (3 ALS-008176:1 placebo)- 18 subjects to receive single oral 12 mg/kg dose of ALS-008176 with 6 subjects receiving placebo
4	Up to 24 subjects* (3 ALS-008176:1 placebo)- 18 subjects to receive a single oral 25 mg/kg dose of ALS-008176 (3 ALS-008176:1 placebo) with 6 subjects receiving placebo.
	As of 25 April 2016, the SAD portion of the study is closed.

*Assuming the IDMC does not initiate Part 2 before completion of the cohort.

Table 5-2. Part 2 MAD Dosing Regimen

Cohort No.	Study Drug Dose Level and Frequency
Japanese Cohort	Up to 24 subjects (3 ALS-008176:1 placebo)- Subjects will initially receive a loading dose of 4.1 mg/kg for Dose 1 on Day 1 followed by 1.37 mg/kg given twice daily for 5 consecutive days (Doses 2-10). The IDMC may dose escalate subsequent patients in the cohort to a dose regimen that is no more than 3-fold higher.
1	Up to 24 subjects (3 ALS-008176:1 placebo) - A loading dose of 10 mg/kg will be administered for Dose 1 on Day 1 followed by 2 mg/kg given twice daily for 5 consecutive days (Doses 2-10).
2	Up to 24 subjects (3 ALS-008176:1 placebo) - A loading dose of 30 mg/kg will be administered for Dose 1 on Day 1 followed by 6 mg/kg given twice daily for 5 consecutive days (Doses 2-10).
3	Neonate cohort - Up to 24 subjects (3 ALS-008176:1 placebo) A loading dose of 10 mg/kg will be administered for Dose 1 on Day 1 followed by 2 mg/kg given twice daily for 5 consecutive days (Doses 2-10).
4	Up to 24 subjects (3 ALS-008176:1 placebo) - A loading dose of 30 mg/kg will be administered for Dose 1 on Day 1 followed by 10 mg/kg given twice daily for 5 consecutive days (Doses 2-10).
5 – 10	Up to 100 subjects (including neonates) may be enrolled across up to 6 cohorts, if authorized by the IDMC. Cohort sizes and dosing regimens will be determined by the IDMC based on emerging safety and PK data within the constraints defined in Section 5.1.1 (3 ALS-008176:1 placebo) - Doses will be given once daily, or twice daily over 3-10 consecutive days.

5.1.1 Cohort Progression Guidelines

In both Parts 1 and 2, the planned dose escalation schema (Figure 2-1) may be modified by the Sponsor, upon approval by the IDMC, based on emerging PK and safety data. In all circumstances, however, the dosing schema will have no more than a 3-fold increase in dose between dose levels. In addition, under no circumstances will a planned pediatric dosing regimen exceed a projected average ALS-008112 $AUC_{0\text{-}24h}$ of 20,000 ng•h/mL. Furthermore,

the decisions to advance the study will be based on a review of safety and PK data by the Sponsor and IDMC as follows:

- Part 1 – Trigger the enrollment of the remainder of each SAD age stratum, and the next age stratum sentinel group, following the evaluation of each Sentinel Group (see Section 2.2)– all available safety data through study completion for all 3 subjects in the age stratum, as well as all available PK data for the first 24 hours after dosing will be reviewed.
- Part 1 –Dose Escalations - all available safety data through study completion for all subjects, as well as available PK data for the first 24 hours after dosing for at least 75% of subjects in the most recent cohort will be reviewed.
- Part 2 - Initiation of the MAD will occur when emerging PK and safety data from Part 1 have been evaluated and are deemed by the IDMC to be acceptable and sufficient to initiate multiple dosing.
- Part 2 – Dose Escalation - any MAD cohort will commence once authorized by the IDMC based on emerging safety and PK data.

Effective after approval of protocol version 6, up to 6 additional cohorts may be enrolled with a maximum of 100 additional subjects (including neonates). The following elements of the study may be adjusted, subject to approval by the IDMC:

- The size of each cohort and the frequency at which cohort data are reviewed. Enrollment will generally be allowed to continue during preparation and review of data by IDMC, unless otherwise instructed by the IDMC.
- Dosing frequency within a cohort may be once daily or twice daily
- The maintenance doses may be the same as the loading dose
- The duration of dosing within a cohort will be 5 days unless otherwise instructed by the IDMC, which may modify the duration within the range of 3 to 10 days, inclusive
- The duration of symptoms from first onset until randomization will be ≤5 days unless otherwise instructed by the IDMC, which may modify the duration to up to 7 days

In Part 1, the Sponsor, upon approval by the IDMC, may advance an older age group(s) to the next dosing cohort before enrollment in younger age group(s) within that dose cohort is complete. However, no age stratum will be advanced to the next dose until that entire age stratum has completed dosing at the current dose level and safety and PK data have been assessed. At no time will dose escalation occur for a younger age stratum before dosing in an older age stratum has been initiated for that dose, unless the IDMC determined that the safety profile supported such an approach.

If enrollment in later cohorts of Part 1 is occurring concurrently with enrollment in Part 2, the Sponsor will determine which study sites will enroll which Part of this protocol.

5.1.1.1 *Stopping Criteria*

For an individual subject:

See Section 4.6.1, *Subject Discontinuation Criteria*.

For a dosing level

Further enrollment within a cohort or dose escalation to subsequent cohorts will be discontinued once it has been established that safety risk(s) such as the following occur:

- If any unacceptable toxicity (as determined by the Sponsor Medical Monitor) occurs
- If the IDMC considers an event or multiple events to represent an unacceptable risk to the health and well-being of subjects at that or higher dose levels
- If ≥2 Grade 3 or 4 adverse events occur which are attributed to study medication by the investigator, Sponsor medical monitor, or IDMC. In this situation, the IDMC will confirm that the subjects who experienced the events were assigned to receive ALS-008176.
- Exposures of a given dosing regimen are projected to deliver a mean AUC of ≥20,000 ng•h/mL for ALS-008112. In such a case, a lower dose may be selected for escalation to maintain a mean ALS-008112 AUC less than 20,000 ng•h/mL.
- A dosing level may also be discontinued by the Sponsor at any time for administrative reasons – e.g., enrollment in a SAD cohort is minimal before the cohort is full because all sites are enrolling in the MAD.

For study

Further enrollment or dosing in the study will be discontinued once it has been established that safety risk(s) such as the following occur:

- Development of clinically important AEs while receiving ALS-008176 that represent a safety risk to subjects and which are without apparent dose response
- The IDMC considers an event or multiple events to represent an unacceptable risk to the health and well-being of subjects at any dose

5.1.2 Treatment Delay or Missed Doses

For Part 2 (MAD), delays in dose administration should be avoided, when possible. All doses should be administered as close to the scheduled dosing time as possible - i.e., ±30 minutes of the scheduled dosing time. Actual dose administration times will be recorded. Dose administration occurring after hospital discharge will be recorded in a subject diary, and transcribed to the CRF.

For twice daily dosing, if a dose is missed, then it should be administered within 6 hours after the scheduled dosing time. If a missed dose cannot be administered within 6 hours after the

scheduled dosing time, then it should be skipped, and the next dose should be given at the scheduled time.

For once daily dosing, if a dose is missed, then it should be administered within 12 hours after the scheduled dosing time. If a missed dose cannot be administered within 12 hours after the scheduled dosing time, then it should be skipped, and the next dose should be given at the scheduled time.

5.1.3 Partial Doses

If a subject is dosed but is believed to have received a partial dose (e.g., due to spitting up study drug or vomiting soon after dosing), do not repeat dosing. The subject should be assumed to have received their full dose and should continue their normal dosing schedule.

5.1.4 Dose Reductions

No dose modification is allowed or required.

5.2 DESCRIPTION OF STUDY DRUG

5.2.1 Study Drug

Study drug will be supplied as a powder, which will be formulated as a suspension in sterile water by the clinical site pharmacist.

Study drug will be packaged in blinded, numbered, child-resistant capped, polyethylene, amber-colored bottles containing the active pharmaceutical ingredient (API) or placebo, to be formulated as a suspension in sterile water.

Study drug bottles containing powder must be stored refrigerated between 2°C and 8°C. After the suspension is prepared, refrigeration of the study drug bottle is recommended but not required.

Each dose will be administered using an appropriate volume colored polypropylene oral dosing syringe.

Complete instructions are located in the study specific Pharmacy Manual. Study drug or placebo will be administered to subjects in a blinded fashion. Doses administered in the hospital will be prepared by pharmacy personnel. The pharmacy personnel will apply a label to the dosing syringe which identifies the study and subject number.

The site will provide parents/guardians with dosing syringes and instructions for dosing at home. Dosing syringes for doses administered in the subject’s home will be prepared by the parent/guardian using the provided instructions.

5.3 DOSE PREPARATION AND ADMINISTRATION

Study drug preparation must be performed by the licensed investigational pharmacist or other authorized personnel with appropriate training. The principal investigator (PI), subinvestigator, or PI-designated health professional should be available during and after study drug administration occurring in the hospital.

For detailed instructions on preparation and administration of study drug, refer to the Pharmacy Manual.

Although prepared study drug suspension and placebo suspension are stable between 15°C and 30°C, refrigeration (2°C and 8°C) of prepared study drug(s) is recommended. Refer to Pharmacy Manual for the most current stability information.

ALS-008176 can be administered without regard to food. The date(s) and time(s) of feeding and dose administration will be recorded (in a diary for MAD subjects).

Study drug must not be added to a feeding bottle or pacifier.

First study drug dose should be given as soon as possible but no later than 18 hours after randomization.

For MAD:

In each twice-daily dosing regimen, a single loading dose (Dose 1) will be followed by a twice daily maintenance dose regimen (Doses 2–10; Preliminary Dosing Schema*

. The 1st maintenance dose (Dose 2) will be given between 8-18 hours after the loading dose (Dose 1) to facilitate getting the subject on an established hospital dosing regimen. All subsequent doses will be given per the hospital's regular twice daily dosing times ±1 hour.

In once daily dosing regimens, the second dose will be given between 21-27 hours after Dose 1 to facilitate getting the subject on an established hospital daily dosing regimen. All subsequent doses will be given per the hospital's regular once daily dosing times ±1 hour.

Based on emerging data from this study, the dosing duration may be 3-10 days if instructed by the IDMC.

If a subject is discharged from the hospital before the last dose is administered, the remaining study drug doses will be administered on the same schedule at home by parent/legal guardian.

5.4 ORDERING STUDY DRUG

Study drug initial site supply and site resupply will be automated by the IRT system, and will be dependent on site activation and prior subject enrollments. Refer to the Pharmacy Manual or contact your clinical research associate (CRA) with any drug supply questions.

5.5 DRUG ACCOUNTABILITY

Study site personnel will maintain adequate records of the receipt and disposition of all study medication shipped to the site. Records must include dates, lot numbers, quantities received, quantities dispensed, date and time of preparation, date and time of administration, date dispensed and date returned (if applicable), and the identification number of each subject who has received each lot of study drug.

The investigator or designee will administer study drug only to subjects enrolled in this protocol. The investigator will not supply study drug to any person not authorized to receive it.

5.6 DISPOSITION OF USED, PARTIALLY USED, AND UNUSED VIALS

All used and unused study drug supplied by Alios BioPharma must be retained by the pharmacist. Periodically throughout and at the conclusion (or suspension, termination, or discontinuation) of the study, a representative of Alios BioPharma or its designated agent will conduct inventories and accountability of unassigned study materials only. Once accountability is completed, an Alios BioPharma representative or designee will authorize the return of all used and unused bottles to the Alios BioPharma-contracted destruction facility or authorize the destruction of the bottles by the study site. Adequate documentation to support destruction is required prior to destruction of the bottles, and a certificate of destruction or written documentation that specifies the date, quantity, lot numbers, and method of destruction that is traceable to the study drug must be provided to Alios BioPharma. For bottles returned to Alios BioPharma or its designated agent, records will include dates, lot numbers, and quantities of study drug returned.

5.7 CONCOMITANT MEDICATIONS

Concomitant medications, except those listed in Section 5.8, *Prohibited Medications* are allowed during this study. Record **all** concomitant medications and supportive therapy from the date the informed consent is signed through to the Completion Visit. Note the dosage, route of administration, frequency, start and stop dates, and the indication in the source documentation.

Prescription medications intended to treat the symptoms/sequelae of the RSV infection are permitted, including:

- Inhaled β-agonists
- Inhaled corticosteroids

- Antibiotics such as beta-lactams and cephalosporins

NOTE: The temporary use of over the counter medications in the 14 days prior to randomization is permitted. The use of vitamins and mineral supplements is also permitted.

5.8 PROHIBITED MEDICATIONS

Prohibited medications during the conduct of this study include:

- Herbal supplements which have evidence of adversely affecting absorption and clearance mechanisms (e.g., strong inhibitors of OAT3). Herbal supplements which are not likely to adversely affect either absorption or clearance mechanism of ALS-008176 during dosing may be permitted on a case by case basis. These cases will need to be reviewed and approved by the Sponsor Medical Monitor. The decision to allow the use of a particular herbal supplement will be based on a review of the available scientific information on the product.
- Prescription medications which are known to modulate the host immune response and/or increase viral shedding such as immunomodulatory therapies. (Systemic and inhaled corticosteroids are permitted, however).
- Prescription medications to treat the RSV infection itself (e.g., inhaled/oral ribavirin, RSV intravenous immunoglobulin).
- Prescription medications which are known to be strong inhibitors of the OAT3 transporter are prohibited:
    - Cimetidine
    - Diclofenac
    - Probenecid
- Palivizumab or other RSV prophylactic medication (approved or investigational)
- Investigational vaccine
- Any other investigational medication

NOTE: The list of Prohibited Medications above is subject to change (e.g., if new OAT3 substrates or inhibitors are identified in the medical literature after finalization of the protocol). Any revisions to the list above will be available in the Study Manual.

If a breast-feeding subject's mother requires any of the above prohibited medications, contact the Medical Monitor to discuss the appropriateness of continuing the subject in the study.

Subjects that receive a prohibited medication may be required, after a discussion between the PI and Sponsor Medical Monitor, to discontinue study treatment. If a subject is to discontinue treatment, follow the subject as outlined in Section 4.6.2, *Procedures for Subjects who Discontinue.* Alert the medical monitor if a subject receives a prohibited medication.

6.0 STUDY PROCEDURES

Subjects must receive the first dose of study drug within 18 hours after randomization. See Table 6-1, *Schedule of Events SAD Phase (Part 1)* and Table 6-2, *Schedule of Events MAD Phase (Part 2) Twice Daily Dosing* and Table 6-3, *Schedule of Events MAD Phase (Part 2) Once Daily Dosing*, for an overview of the study procedures.

Table 6-1. Schedule of Events: SAD Phase (Part 1)

**Days**	**Screen**	**Hospitalized Study Days**							**Completion Visit [2]**
		**Day 1**					**Day 2**	**Days[1] 3 to 6**	
**Assessments**	**(-3) to 1**	**Predose (-0.5 hr)**	**Dose**	**0.5-1 hr Postdose**	**3 (±0.5) hr Postdose**	**7 (±2) hr Postdose**	**24 (±2) hr Postdose**	**QAM**	**Day 7 (±1 day)**
Obtain informed consent	X								
Demographics/Eligibility	X								
Medical History	X								
Length, Head Circumference	X								
Weight	X						X		X
Complete Physical Exam	X								X
Directed Physical Exam				X [3]			X	X	
Vital Signs, Oxygen Saturation [4]	X			X			X	X	X
12-Lead electrocardiogram [4]	X			X					X
Randomization		≤8 hours predose							
**Study drug administration [5]**			X						
AE Evaluation/Medications/Clinical Evaluation	X———————————————————————X								
Nasopharyngeal Swab or Nasal Aspirate: RSV Diagnosis	X								
Nasal Aspirate: PD (RSV Viral RNA), resistance, biomarkers		X				X	X	X[8]	X
PK Samples [6]: Group A	Optional			X	X				Optional
PK Samples [6]: Group B	Optional			X		X			Optional
Serum Chemistries7	X				X			X[8]	X
Hematology [7]	X				X			X[8]	X

AE: adverse event; QAM: every morning.

All study procedures should be conducted as close as possible to protocol-specified times, but also in a manner that minimizes stress to the subject.

1. Assessments to be performed once daily in the AM while still hospitalized
2. If subject remains hospitalized after Day 6, conduct the Completion Visit assessments on Day 7 (±1 day). Hospitalization duration will not be extended solely for study purposes. Telephone calls to parents to facilitate safety monitoring of outpatients between study visits are permitted.
3. The postdosing directed physical exam on Day 1 must be performed between 1-3 hours after administration of the study drug dose.
4. 12-lead ECG and then Vital signs (BP, HR, RR, body temperature, oxygen saturation) should be performed prior to invasive procedures such as blood draws and nasal aspirates
5. Study drug should be given as soon as possible but no later than 8 hours after randomization. Feeding is recommended within 1 hour prior to study drug administration. The date and time of feeding and dose administration will be recorded.
6. PK may be assessed using blood collected for screening and Day 7 safety assessments (blood volume permitting). In addition, at randomization, each subject will be randomly assigned (stratified by age group) to 1 of 2 PK sampling schemes (Group A or B): 2 PK time points in the first 24 hours postdose/Group. Based on a review of prior cohorts' PK data, the time points of blood draws for PK assessments may change (see Section 6.1.3). If blood collection volume is limited, the order of priority is the following: safety>PK (Screening and Completion Visit), PK>safety (Day 1).
7. Safety labs (serum chemistries + hematology) will be assessed predose, 3-7 hours postdose, and Day 7.
8. Nasal aspirates, serum chemistries, hematology during hospitalization are not required after 24 hours postdose. However, if blood collection or nasal aspiration is performed during routine clinical care while hospitalized, "scavenging" of such specimens for additional viral, safety, PK and biomarker analysis is permitted.

Table 6-2. Schedule of Events: MAD Phase (Part 2) Twice-Daily Dosing

Days	Screen	Hospitalized Study Days						Safety Visit[2]	Completion Visit	Follow-up Visit
		Day 1-2					Day 3 [1]until Completion Visit			
		Dose 1			Dose 2	Dose 3				
**Assessments**	**Day (-3) to 1**	**Pre-Dose 1 (-0.5 hr)**	**Dose 1**	**0.25-2 hr Post-Dose 1**	**0.5-1 hr Post-Dose 2 OR 7 (±4) hr Post-Dose 2**	**12 (±4) hr Post-Dose 2**	**QAM**	**Last day of dosing (-2 day)**	**6 (±1) days after the last day of dosing**	**Day 28 (±7 days)**
Obtain informed consent	X									
Demographics/Eligibility	X									
Medical History	X									
Length, Head Circumference	X									
Weight	X					X		X		X
Complete Physical Exam	X									X
Directed Physical Exam				X		X	X[1]	X	X	
Vital Signs, Oxygen Saturation[3]	X			X		X	X[1]	X	X	X[8]
12-Lead electrocardiogram[3]	X			X				X	X	X[8]
Randomization		≤18 hr predose								
**Study drug administration [4]**			X[4]							
AE Evaluation/Medications/ Clinical Evaluation	X——————————————————————————X									
Nasopharyngeal Swab or Nasal Aspirate: RSV Diagnosis	X									
NasalSwab[10] or Aspirate: PD (RSV Viral RNA), resistance, PK[11], biomarkers		X			X (0.5-1 hr)	X	X[10]	X	X	X[8]
PK Samples[5]: Group A	Optional			X	X (0.5–1 hr)			X	Optional	
PK Samples[5]: Group B	Optional			X	X (7±4 hr)			X	Optional	
Serum Chemistries[6]	X				X (same as PK)		X[7]	X	X	X[8]
Hematology[6]	X				X (same as PK)		X[7]	X	X	X[8]
Blood Biomarkers[6]	X				X (same as PK)		X[7]	X	X	X[9]

All study procedures should be conducted as close as possible to protocol-specified times, but also in a manner that minimizes stress to the subject.

1. Assessments to be performed once daily in the AM while still hospitalized. Hospitalization duration will not be extended solely for study purposes. Telephone calls to parent/guardian to facilitate safety monitoring of outpatients between discharge and Completion visit are permitted.
2. The Safety Visit will occur on the last day of dosing unless not feasible due to clinic availability, in which case it should occur as close as possible to the last day of dosing with a visit window of no more than -2 days. If the IDMC requires that the treatment duration be 9 or 10 days, the Safety Visit should be completed both on Day 5 and the last day of dosing (i.e., Day 9 or 10 (-2 day window depending on clinic availability), based on IDMC instructions).
3. 12-lead ECG and then Vital signs (BP, HR, RR, body temperature, oxygen saturation) should be performed prior to invasive procedures such as blood draws and nasal swabs/aspirates
4. Study drug should be given as soon as possible but no later than 18 hours after randomization. In each dosing regimen, a single loading dose (Dose 1) will be followed by a twice daily maintenance dose regimen (Doses 2–10 for a 5 day dosing regimen). The first maintenance dose (Dose 2) may be given between 8-18 hours after the loading dose (Dose 1) to facilitate getting the subject on an established hospital dosing regimen. The date and time of feeding and dosing will be recorded (using a dosing diary for doses administered after hospital discharge).
5. PK may be assessed using blood collected for screening and Completion Visit safety assessments (blood volume permitting). In addition, at randomization, each subject will be randomly assigned (stratified by age group) to 1 of 2 PK sampling schemes (Group A or B): 3 PK time points per group (twice in the first 24 hours postdose, and a random sample at any time on Last day of dosing. Based on a review of prior cohorts' PK data, the time points of blood draws for PK assessments may change (see Section 6.1.3). If blood collection volume is limited, the order of priority is the following: safety>PK>Biomarkers (Screening, Safety Visit, and Completion Visit), PK>safety>Biomarkers (Day 1).
6. If the MAD blood volume exceeds local blood volume limits, do not collect biomarker samples.
7. Serum chemistries, hematology, and biomarker assessments during hospitalization are not required >24 hours after the first dose. However, if blood collection is performed during routine clinical care while hospitalized, "scavenging" of such specimens for safety, PK and biomarker analysis is permitted.
8. Follow Up visit may be conducted as a home visit by a physician. Complete physical exam and weight are required. All other safety assessments need only be collected if deemed clinically necessary based on an assessment of the subject's safety (e.g., adverse events) or as follow up of an earlier adverse event (e.g., thrombocytosis). A nasal swab aspirate specimen is encouraged but not required.
9. Optional: If a blood specimen is collected for safety assessments during the follow-up visit, enough blood should be collected to assess blood biomarkers
10. Nasal swabs will be used for all future cohorts initiated after approval of protocol version 6. Nasal swabs are required to be collected daily through the last day of dosing and, if local regulations permit and the parents are willing/able, should continue to be collected through to the Completion Visit. Ideally, post-discharge swabs will be collected by site staff in the clinic or at a home visit. If this is not possible, the parent may collect the swab. The site will provide parents/guardians with supplies and instructions for collecting nasal swabs at home.
11. Nasal PK may be analyzed if a suitable assay is available.

Table 6-3. Schedule of Events: MAD Phase (Part 2) Once-Daily Dosing

Days	Screen	Hospitalized Study Days					Safety Visit[2]	Completion Visit	Follow-up Visit
		Day 1			Day 2	Day 3 [1] until Completion Visit			
		Dose 1			Dose 2				
**Assessments**	**Day (-3) to 1**	**Predose 1 (-0.5 hr)**	**Dose 1**	**0.25-2 hr Post-Dose 1 OR 12(±2) hr Post-Dose 1**	**0.5-1 hr Post-Dose 2 OR 7 (±4) hr Post-Dose 2**	**QAM**	**Last Day of Dosing (-2 day)**	**6 (±1) Days After the Last Day of Dosing**	**Day 28 (±7 days)**
Obtain informed consent	X								
Demographics/Eligibility	X								
Medical History	X								
Length, Head Circumference	X								
Weight	X				X (0.5–1 hr)		X		X
Complete Physical Exam	X								X
Directed Physical Exam				X (0.25-2 hr)	X (0.5–1 hr)	X[1]	X	X	
Vital Signs, Oxygen Saturation[3]	X			X (0.25-2 hr)	X (0.5–1 hr)	X[1]	X	X	X[8]
12-Lead electrocardiogram[3]	X			X (0.25-2 hr)			X	X	X[8]
Randomization		≤18 hr predose							
**Study drug administration [4]**			X[4]						
AE Evaluation/Medications/ Clinical Evaluation	X	—	—	—	—	—	—	—	X
Nasopharyngeal Swab or Nasal Aspirate: RSV Diagnosis	X								
NasalSwab[10] or Aspirate: PD (RSV Viral RNA), resistance, PK[11], biomarkers		X		X[10] (12 hr)	X[10] (24 hr post-dose 1)	X[10]	X[10]	X[10]	X[8]
PK Samples[5]: Group A	Optional			X (0.25-2 hr)	X (0.5–1 hr)		X	Optional	
PK Samples[5:] Group B	Optional			X (0.25-2 hr)	X (7±4 hr)		X	Optional	
Serum Chemistries[6]	X				X (same as PK)	X[7]	X	X	X[8]
Hematology[6]	X				X (same as PK)	X[7]	X	X	X[8]
Blood Biomarkers[6]	X				X (same as PK)	X[7]	X	X	X[9]

All study procedures should be conducted as close as possible to protocol-specified times, but also in a manner that minimizes stress to the subject.

1. Assessments to be performed once daily in the AM while still hospitalized. Hospitalization duration will not be extended solely for study purposes. Telephone calls to parent/guardian to facilitate safety monitoring of outpatients between discharge and Completion visit are permitted.
2. The Safety Visit will occur on the last day of dosing unless not feasible due to clinic availability, in which case it should occur as close as possible to the last day of dosing with a visit window of no more than -2 days. If the IDMC requires that the treatment duration be 9 or 10 days, the Safety Visit should be completed both on Day 5 and the last day of dosing (i.e., Day 9 or 10 (-2 day window depending on clinic availability), based on IDMC instructions).
3. 12-lead ECG and then Vital signs (BP, HR, RR, body temperature, oxygen saturation) should be performed prior to invasive procedures such as blood draws and nasal swabs/aspirates
4. Study drug should be given as soon as possible but no later than 18 hours after randomization. In each dosing regimen, a single loading dose (Dose 1) will be followed by a once daily maintenance dose regimen. The first maintenance dose (Dose 2) will be given 21-27 hours after Dose 1. The total number of doses and days study drug is to be administered will vary depending on IDMC instructions for a cohort. The date and time of feeding and dosing will be recorded (using a dosing diary for doses administered after hospital discharge).
5. PK may be assessed using blood collected for screening and Completion Visit safety assessments (blood volume permitting). In addition, at randomization, each subject will be randomly assigned (stratified by age group) to 1 of 2 PK sampling schemes (Group A or B): 3 PK time points per group (twice in the first 36 hours postdose, and a random sample at any time on Last day of dosing. Based on a review of prior cohorts' PK data, the time points of blood draws for PK assessments may change (see Section 6.1.3). If blood collection volume is limited, the order of priority is the following: safety>PK>Biomarkers (Screening, Safety Visit, and Completion Visit), PK>safety>Biomarkers (Day 1).
6. If the MAD blood volume exceeds local blood volume limits, do not collect biomarker samples.
7. Serum chemistries, hematology, and biomarker assessments during hospitalization are not required >24 hours after the first dose. However, if blood collection is performed during routine clinical care while hospitalized, "scavenging" of such specimens for safety, PK and biomarker analysis is permitted.
8. Follow-up Visit may also be conducted as a home visit by a physician. Complete physical exam and weight are required. All other safety assessments need only be collected if deemed clinically necessary based on an assessment of the subject's safety (e.g., adverse events) or as follow up of an earlier adverse event (e.g., thrombocytosis). A nasal swab specimen is encouraged but not required.
9. Optional: If a blood specimen is collected for safety assessments during the follow-up visit, enough blood should be collected to assess blood biomarkers
10. Nasal swabs will be used for all future cohorts initiated after approval of protocol version 6. Nasal swabs are required to be collected daily through the last day of dosing and, if local regulations permit and the parents are willing/able, should continue to be collected through to the Completion Visit. Ideally, post-discharge swabs will be collected by site staff in the clinic or at a home visit. If this is not possible, the parent/guardian may collect the swab. The site will provide parents/guardians with supplies and instructions for collecting nasal swabs at home.
11. Nasal PK may be analyzed if a suitable assay is available.

6.1 ON-STUDY EVALUATIONS, PROCEDURES, AND DOSING

Where multiple procedures are scheduled at the same time point(s) relative to dosing, the following chronology of events should be adhered to, where possible:

- ECGs: obtain prior to vital signs and as close as possible to scheduled time, but prior to invasive procedures such as blood draws and nasal swabs or aspirates
- Blood pressure/pulse rate: obtain as close as possible to scheduled time, but prior to blood specimen collection
- PK blood specimens: obtain within the time window stated in the Schedule of Events
- Nasal swabs or aspirates: obtain after safety and/or PK assessments
- All other procedures should be obtained as close as possible to the scheduled time, and may be obtained before or after blood specimen collection

6.1.1 Diet, Fluid, and Activity

ALS-008176 can be administered without regard to food. Feeding times will be recorded. Study drug must not be added to a feeding bottle or pacifier.

6.1.2 Serum Chemistries and Complete Blood Count with Differential

Required safety laboratory variables are defined in Section 8.2.3, *Clinical Laboratory Measurements*, and will be assessed by the site's local laboratory.

Blood for serum chemistries, CBC with differential sampling time points: All subjects will have a predose sample obtained to establish a safety baseline. (If all safety labs were obtained within 96 hours of randomization as a part of routine clinical care, the screening safety labs do not need to be collected.)

If blood collection volume is limited, the order of priority is the following: safety>PK>biomarkers (Screening, Safety Visit/Last Day of Dosing and Day 28 Follow-up Visit (MAD only), and Completion Visit), PK>safety>biomarkers (Day 1).

Serum chemistries, CBC with differential, and PK assessments during hospitalization are not required >24 hours after dosing, however, if blood samples are obtained as a part of routine clinical care, every effort should be made to "scavenge" left over blood sample for PK, safety, or biomarker analysis. The date and time the scavenged blood specimen was obtained should be documented.

6.1.3 Pharmacokinetic Sampling

Sponsor will provide kits for all PK sample collections.

Each subject will have a maximum of 4 (for SAD) or 6 (for MAD for dosing duration <9 days) or 7 (for MAD dosing duration of 9-10 days) study-mandated blood samples

collected over the course of the study in order to determine the safety and pharmacokinetics of ALS-008176. Within each age stratum, subjects will be randomly assigned to one of 2 different PK sampling schedules to minimize the number of samples required from each subject. When combined, this will result in a more complete PK profile.

- At randomization, within each age stratum, each subject will be randomly assigned to 1 of 2 collection schedules for 2 postdose PK assessments. See Schedule of Events. Any of these samples may also be used to evaluate safety laboratory parameters, if needed.
- Subjects participating in Part 2 (MAD) will have one additional PK lab sample collected on Last Day of Dosing(and one on Day 5 if dosing duration is 9-10 days) and one blood sample, if necessary, for safety labs and biomarkers collected on Day 28.

Based on a review of prior cohorts' PK data, the time points of blood draws for PK assessments may change; additionally, if emerging PK data suggest fewer blood draws for PK assessments are needed, the number of blood draws for PK time points in future cohorts may decrease. The total number of blood draws for safety and PK assessments combined will not increase, however, without a protocol amendment.

Blood samples for PK analysis may be collected at the scheduled times through micropuncture (e.g., heel stick) or intravenous blood draws, based on subject/parent/investigator preference, using K_2 EDTA Vacutainer collection tubes. Saline or heparin flushes may be used to maintain viability of indwelling catheters, if used. Additional information may be found in the Laboratory Manual.

If blood samples are obtained at other times as a part of routine clinical care, every effort should be made to "scavenge" left over blood samples for PK or safety analysis. All scavenged samples used for PK analysis will be reported and included in the PK calculations. The date and time of the scavenged blood specimen collection should be documented. All scavenged samples should be stored at -80°C (preferred) or -20°C as soon as possible.

If blood collection volume is limited, the order of priority is the following: safety>PK>biomarkers (Screening, Last Day of Dosing/Day 5 for 9-10 days dosing duration and Day 28 Follow-up Visit (MAD only), and Completion Visit), PK>safety>biomarkers (Day 1).Sample processing and shipping instructions are detailed in the Laboratory Manual.

Blood concentrations of ALS-008112 and ALS-008144 (and other metabolites as applicable) will be determined using a validated liquid chromatography tandem mass spectrometry method. Concentrations of ALS-008112 and ALS-008144 (and other metabolites as applicable) may also be evaluated in nasal swabs if a sensitive method can be established. Samples may be used for future metabolite identification and/or further evaluation of the bioanalytical method. These data will be used for internal exploratory purposes and will not be included in the clinical study report.

To maintain the study blind, results of the individual PK analyses will not be shared with the PI or Sponsor representatives directly involved in managing the study. Unblinded dose level PK data will be provided to the IDMC and select Sponsor representatives to assist with dose escalation decisions during the course of the study.

An unblinded Sponsor representative not directly involved in managing the study may have access to individual subject level PK data, if needed.

6.1.4 Central Laboratory Evaluations

Central laboratories will be used for confirmatory qualitative PCR RSV diagnostic confirmation, quantitative PCR RSV RNA concentrations, resistance monitoring, analyses of PK samples, and testing of biomarkers (as applicable).

Sponsor will provide kits for all non-safety sample collections.

Sites will ship all non-safety study samples to a single laboratory repository. The repository will further distribute the samples to the appropriate analyzing laboratories.

Local laboratories will be used for all routine screening and study related safety laboratory evaluations.

6.1.5 RSV Evaluations

Nasal Swabs

Upon receiving approval of protocol v6, nasal swab specimens will be collected. The procedure for sample collection is defined in the Study Manual. Supplies to perform the nasal swab procedure will be provided to sites by the Sponsor. Parents/guardians may be asked to collect nasal swab specimens; site personnel will provide all needed supplies, procedure instructions, and storage requirements, all of which will be defined in the Study Manual.

RSV Diagnosis

RSV diagnosis will be confirmed prior to randomization by rapid diagnostic assay using the study supplied Binax NOW RSV or any other RSV diagnostic test available at the site. The Alere Binax NOW RSV Card is a rapid immunochromatographic assay for the qualitative detection of RSV fusion protein antigen in nasal wash and nasopharyngeal swab specimens. Note: Informed consent must be obtained before conducting an RSV diagnostic test that is not a standard of care procedure.

Sponsor will provide kits for all RSV-related sample collections.

Qualitative PCR

Qualitative PCR will be performed by a central lab, using predose nasal swab or nasal aspirate sample, to confirm RSV diagnosis, however, study eligibility is determined by the results of the local RSV test.

Samples from BinaxNOW RSV negative screening tests may be sent to the central lab for evaluation using a qualitative PCR assay. This optional sample evaluation process will be conducted at selected centers.

GenMark's Respiratory Viral Panel (RVP) is an assay which utilizes competitive DNA hybridization and electrochemical detection of the following virus types and subtypes:

Influenza A<br>Influenza A H1<br>Influenza A H3<br>Influenza A 2009 H1N1<br>Influenza B	Respiratory Syncytial Virus (RSV) A<br>Respiratory Syncytial Virus (RSV) B<br>Parainfluenza Virus (PIV) 1<br>Parainfluenza Virus (PIV) 2<br>Parainfluenza Virus (PIV) 3	Human Metapneumovirus (hMPV)<br>Human Rhinovirus (HRV)<br>Adenovirus B/E<br>Adenovirus C

Quantitative RT-PCR Assay for RSV

Quantitative assessment of RSV levels in nasal secretions will be assessed using a previously described RT-PCR assay (Perkins et al. 2005).

To maintain the study blind, results of the individual RSV viral load analyses will not be shared with the PI or Sponsor representatives involved in managing the study. The Statistician will manage the processing and distribution of RSV viral load results.

Biomarkers

Nasal secretion (SAD and MAD) and plasma (MAD only) soluble proteins may be assayed using a multiplexed immunoassay technology. These assays evaluate the levels of a range of proteins including interferons, (e.g., interferon gamma), immunoglobulins (e.g., IgA, IgG, IgM), cytokines (e.g., macrophage inflammatory protein-1 beta), interleukins (e.g., interleukin 1, 2, etc), chemokines (e.g., RANTES, Eotaxin-1), growth factors (e.g., vascular endothelial growth factor) and other proteins potentially associated with the inflammatory response induced by acute RSV infection. Whole blood samples may also be tested for mRNA levels using a microarray profiling system which includes genes thought to be involved in the response to RSV infection and any other differentially expressed genes relative to treatment response or dose response.

If total blood volume for study exceeds limits defined by local regulations, or collected blood is limited, blood biomarkers should be omitted.

RSV Resistance Monitoring

Sponsor will provide kits for all RSV-related sample collections.

Nasal secretion samples will be collected for monitoring of potential viral resistance to ALS-008176 by population sequencing of the RSV polymerase coding region (amino acids

550–1100 of the RSV L protein) at baseline, during treatment and posttreatment with ALS-008176. Other regions of the RSV genome may also be sequenced. Specific RSV polymerase amino acid sequence variations detected in samples from a majority of treated subjects will then be selected for the generation of site-directed mutants in the RSV reverse-genetics system. The inhibitory potency of ALS-008112 will then be determined vs. the RSV site-directed mutants compared to a wild-type RSV.

6.1.6 12-Lead Electrocardiograms

The investigator or qualified designee will be responsible for evaluating the results and determining if any findings are of clinical significance. In the event that an invasive procedure such as a blood draw or nasal swab or aspirate and an ECG are required at the same time, ECGs should be collected first, but the blood draw should be obtained as close the scheduled time as possible. ECGs may be repeated at the investigator discretion to account for erroneous readings.

6.1.7 Vital Signs and Oxygen Saturation

Vital signs (body temperature, respiratory rate, and automated supine blood pressure and heart rate) and oxygen saturation will be obtained according to local practice. In the event that an invasive procedure such as a blood draw or nasal swab or aspirate and vital signs are required at the same time, vital signs should be collected first, but the blood draw should be obtained as close the scheduled time as possible.

6.1.8 Physical Examinations

Subjects will have complete or directed physical examinations at the times defined in the Schedule of Events. Complete physical examinations are a comprehensive examination of all major organ systems, while directed physical examinations can be focused on relevant organ systems as determined by AEs, symptoms, laboratory assessments, etc.

6.1.9 Clinical Evaluation

A variety of clinical parameters will be assessed regularly to determine any clinical effects ALS-008176 might have on the course of RSV infection. Clinical parameters to be assessed include, but are not limited to:

- Symptoms (e.g., runny nose, wheezing, cough)
- Oxygen requirement (e.g., supplemental oxygen, non-invasive positive pressure ventilation, mechanical ventilation)
- Level of hospital care (e.g., intensive care unit, transitional care unit, ward floor)
- Duration of hospitalization

7.0 SAFETY MONITORING AND REPORTING

7.1 DEFINITIONS

7.1.1 Pretreatment Events

A pretreatment event is any event that meets the criteria for an AE/SAE and occurs after the subject signs the informed consent form but before receiving the first administration of study drug.

7.1.2 Adverse Events

An AE is any event, side effect, or untoward medical occurrence in a subject enrolled in a clinical trial whether or not it is considered to have a causal relationship to one or more of the study drugs. An AE can therefore be any new, or worsening of an existing, unfavorable and unintended sign, symptom, laboratory finding outside of normal range , physical examination finding, or disease temporally associated with the use of the study drug, whether or not the event is considered related to the study drug.

Planned hospital admissions or surgical procedures for an illness or disease that existed before the subject was enrolled in the study are not to be considered AEs unless the condition deteriorated in an unexpected manner during the study (e.g., surgery was performed earlier than planned).

7.1.3 Treatment Emergent Adverse Events

A treatment emergent adverse event is an adverse event that occurs after the first dose of study drug is administered.

7.1.4 Serious Adverse Events

A Serious Adverse Event (SAE) is any untoward medical occurrence at any dose that:

1) Results in death: This includes deaths that appear to be completely unrelated to study medication (e.g., a car accident).
2) Is life-threatening Event: An event that places the subject at immediate risk of death at the time of the event; it does not refer to an event that hypothetically might have caused death if it were more severe.
3) Requires inpatient hospitalization or prolonged hospitalization of an existing hospitalization.
4) Results in permanent or prolonged (at least 28 days in duration) disability or incapacity.
5) Is a congenital anomaly or birth defect in the offspring of a study subject.
6) Medically important event: An event that may not be immediately life-threatening, or result in death or hospitalization, or require intervention to prevent one of the outcomes

listed above, but is considered medically significant for other reasons. An opportunistic or otherwise unusual infection for the investigator's practice, such as tuberculosis, will be considered medically significant.

7) EU and Japan: transmission of infectious agents

The term severe is used to describe the intensity of a specific event (as in mild, moderate, or severe); the event itself, however, may be of minor medical significance (such as severe headache). This is not the same as serious, which is based on outcome of the event, as described above. Seriousness, not intensity, serves as a guide for defining regulatory reporting obligations.

7.2 DOCUMENTING AND REPORTING OF AES (INCLUDING SAES)

Adverse Events will be evaluated and documented using the grading scales contained in the Division of AIDS Table for Grading the Severity of Adult and Pediatric AEs (December 2004, Clarification August 2009) and shown in Appendix C.

7.2.1 Documenting and Reporting Pretreatment Events

For enrolled subjects, record all pretreatment AEs that occur after the parent/guardian signs the informed consent form but before the first study drug administration on the AE CRF and Clinical Trials SAE form (if applicable). The AE CRF and SAE form will indicate that the event occurred prior to the first dose of study drug.

7.2.2 Documenting and Reporting Adverse Events

Telephone calls to parents to facilitate safety monitoring of outpatients between study visits are permitted.

Record all AEs that occur from start of study medication administration to the Completion Visit, regardless of the intensity, seriousness, or relationship to study drug, on the AE CRF, for all enrolled subjects.

Once an event has resolved, any recurrence will be reported as a new event with a corresponding grade.

Whenever possible, report AEs as a specific diagnosis or syndrome (e.g., flu syndrome) rather than as individual signs or symptoms. If no specific diagnosis or syndrome is identified, AEs should be reported as separate and individual events.

An AE includes the following:

- Preexisting event that increases in frequency or intensity.
- Condition detected or diagnosed during the study period, even though it may have been present, in retrospect, prior to the first dose of study drug.

- New or worsening laboratory abnormalities
- An overdose of the study drug without any signs or symptoms will be considered an AE. A calculated dose that exceeds its correct dose by 10% or more and is administered to the subject will be considered an overdose and documented as an AE.

The following events **will not** be identified as AEs in this study:

- Progression or exacerbation of the subject's underlying disease. However, clinical sequelae that result from disease progression are reportable as AEs.
- Medical or surgical procedures (e.g., surgery, endoscopy, tooth extraction, etc); however, the condition (the "triggering event") that leads to the procedure may be an AE.
- Preexisting conditions present or detected prior to the first dose of study drug that do not worsen.

7.2.3 Assigning Attribution of Adverse Events

The investigator **must** attempt to determine the cause of each event. To ensure consistency of AE/SAE causality assessments, investigators should apply the following guideline:

Related:

There is an association between the event and the administration of investigational study drug, a plausible mechanism for the event to be related to the investigational study drug and causes other than the investigational study drug have been ruled out, and/or the event re-appeared on re-exposure to the investigational study drug.

Possibly Related:

There is an association between the event and the administration of the investigational study drug and there is a plausible mechanism for the event to be related to investigational study drug, but there may also be alternative etiology, such as characteristics of the subject's clinical status or underlying disease.

Unlikely Related:

The event is unlikely to be related to the investigational study drug and likely to be related to factors other than investigational study drug.

Not Related:

The event is related to an etiology other than the investigational study drug (the alternative etiology must be documented in the study subject's medical record).

7.2.4 Classifying Action Taken with Study Drug

Classification	Definition
Dose Not Changed	Study Drug dose not changed in response to the AE
Drug Interrupted	Study drug administration interrupted in response to an AE
Drug Discontinued	Study drug administration permanently discontinued in response to an AE
Not Applicable	Action taken regarding study drug administration does not apply. “Not applicable” should be used in circumstances such as when the investigational treatment had been completed before the adverse event began and no opportunity to decide whether to continue, interrupt or discontinue treatment is possible.

7.2.5 Classifying Adverse Event Outcome

Classification	Definition
Recovered/Resolved	Resolution of an AE with no residual signs or symptoms
Recovered/Resolved with sequelae	Resolution of an AE with residual signs or symptoms
Not Recovered/ Not resolved (continuing)	Either incomplete improvement or no improvement of an AE, such that it remains ongoing
Fatal	Outcome of an AE is death. “Fatal” should be used when death is at least possibly related to the adverse event.
Unknown	Outcome of an AE is not known (e.g., a subject lost to follow up)

7.2.6 Documenting and Reporting Serious Pretreatment Events and Serious Adverse Events

Report all Serious Pretreatment Events that occur after the subject signs the informed consent form, but before the first administration of study drug on the SAE form and AE CRF. The SAE form and AE CRF will indicate that the event occurred prior to the first dose of study drug.

Report all SAEs that occur from start of study medication administration to the Completion Visit on the SAE form and AE CRF.

Take the following steps to document and promptly report serious pretreatment events and SAEs, even if the event may not appear to be related to the study drug:

- Submit all available information to INVENTIV HEALTH Global Safety and Pharmacovigilance **by facsimile (preferred)** or email/telephone **within 24 hours** of becoming aware of the SAE, using the INVENTIV HEALTH Serious Adverse Event form, and inform the Medical Monitor via email (see contact information on page 2).
- Report all fatal and life-threatening events (as defined previously) **within 24 hours by facsimile (preferred)** or email/telephone to INVENTIV HEALTH Global Safety and Pharmacovigilance.

- If complete information is not available, provide, as a minimum, subject identifier, suspect drug, site identifier, event or outcome, and investigator assessment of causal relationship to study drug.
- If reporting a serious adverse event, complete the AE CRF and submit immediately.
- Provide follow-up reports on the original INVENTIV HEALTH SAE Form as new information becomes available. A query regarding a follow-up report from INVENTIV HEALTH should be answered within 5 working days from receipt of the query.
- Perform appropriate diagnostic tests and therapeutic measures, if necessary.
- Review the SAE report with INVENTIV HEALTH staff and/or the medical monitor, if needed.
- Report all SAEs promptly to the IEC, if applicable. See International Conference on Harmonisation Good Clinical Practice (ICH GCP) E6, Section 4.11.1; this information can be accessed at: *http://www.fda.gov/cder/guidance/959fnl.pdf*.

Also note the following:

- If a subject is hospitalized with several active medical conditions, the SAE is only that condition (the triggering event) that results in the hospitalization.
- Complications that occur during hospitalization are AEs. These events are only SAEs if they prolong the hospitalization or meet any of the other criteria rendering an AE as serious.
- File an SAE report if a subject is hospitalized for any period of time (even if the subject is admitted to the hospital during the day, but doesn’t spend the night). An SAE report does not need to be filed for a visit to an outpatient clinic or emergency room that is not associated with a hospitalization, unless one of the other serious outcomes (e.g., life-threatening event, event requiring intervention to prevent permanent impairment or damage, etc) occurs during the visit.
- Whenever possible, report SAEs as a specific diagnosis or syndrome (e.g., flu syndrome), rather than as individual signs or symptoms (fever, myalgia, etc). If no specific diagnosis or syndrome is identified, report the primary symptom. Capture additional symptoms in the *SAE Description* field on the SAE form.
- Do not report a hospitalization that is strictly due to disease progression or exacerbation as an SAE. However, do report identifiable clinical sequelae that result from the disease progression (e.g., pleural effusion or a small bowel obstruction).
- Always include a rationale for the causality assessment of an SAE, so that a better understanding of the event can be compiled.

7.3 FOLLOW-UP OF ADVERSE EVENTS AND SERIOUS ADVERSE EVENTS

Follow all AEs (serious and non-serious) until resolution or otherwise explained, the subject dies, the event stabilizes and is not expected to further resolve, or when alternative therapy is instituted, whichever occurs first. AEs that are non-serious and stable do not need to be followed after the last study visit - they should instead be marked as continuing as of the final study visit. Alios BioPharma may request that the investigator perform or arrange for supplemental measurements or evaluations to further clarify the nature of the event.

7.4 SPONSOR'S REVIEW OF ADVERSE EVENTS AND SERIOUS ADVERSE EVENTS

Alios BioPharma will maintain an ongoing review of all AEs and SAEs.

7.5 INDEPENDENT DATA MONITORING COMMITTEE

An IDMC consisting of at least a statistician and three physicians with appropriate backgrounds in the care of infants with RSV infection will be formed. The IDMC will conduct unblinded reviews of safety data on an ongoing basis. The sponsor will make recommendations to the IDMC based on reviews of blinded data. The IDMC will approve decisions on the following topics:

- Opening further enrollment based on acceptable safety and PK profile in SAD Sentinel groups
- Continuation of the study
- Any modification of the dose escalation scheme
- Advancement from one cohort to the next throughout the study
- Opening MAD Part 2 of study based on an acceptable safety profile in SAD Part 1

Effective after approval of protocol version 6, up to 6 additional cohorts may be enrolled with a maximum of 100 additional subjects (including neonates). The following elements of the study may be adjusted, subject to approval by the IDMC:

- The size of each cohort and the frequency at which cohort data are reviewed. Enrollment will generally be allowed to continue during preparation and review of data by IDMC, unless otherwise instructed by the IDMC.
- Dosing frequency within a cohort may be once daily or twice daily
- The maintenance doses may be the same as the loading dose
- The duration of dosing within a cohort will be 5 days unless otherwise instructed by the IDMC, which may modify the duration within the range of 3 to 10 days, inclusive
- The duration of symptoms from first onset until randomization will be ≤5 days unless otherwise instructed by the IDMC, which may modify the duration to up to 7 days

Further details will be defined in the IDMC Charter.

7.6 UNBLINDING PROCEDURES

The treatment assignments will be made available to the PI, through the IRT system, in the event of a medical emergency or an adverse event that necessitates identification for the welfare of a subject. Whenever possible, the PI will contact the Medical Monitor to discuss the need to break the blind prior to proceeding with unblinding. The PI will notify the Medical Monitor as soon as is practical in the event of the study blind being broken and will document the reason.

Randomization information will be made available to the inVentiv Global Safety and Pharmacovigilance Project Lead in order to break the blind when required for safety reporting to regulatory authorities.

8.0 STUDY VARIABLES AND MEASUREMENTS

8.1 EFFICACY VARIABLES/MEASUREMENTS

Antiviral activity is a secondary study objective. RSV RNA will be measured at multiple times by a quantitative PCR method.

Exploratory efficacy measures include collection of data on: length of hospital stay, need and duration of supplemental oxygen requirement, need and duration of ICU stay, need and duration of mechanical ventilation, need and duration of non-invasive ventilator support, time to resolution of RSV symptoms and various biomarkers potentially associated with the inflammatory response induced by acute RSV infection.

8.2 SAFETY VARIABLES/MEASUREMENTS

Safety evaluation will include AEs and SAEs, vital signs, laboratory tests (including hematology and serum chemistries), ECG, and physical examination.

In this study, an estimated total of up to 5.65 or 16.85 mL of blood will be drawn in each SAD or MAD study subject (dosing duration <9 days), respectively, over the course of the study. This blood volume represents 1.77 or 5.27 mL/kg for a 3.2-kg infant (smallest subject enrolled (neonate) as of 15 August 2016) and is within the generally accepted range for blood draws for pediatric clinical research studies (Howie 2011). If the IDMC requires that the MAD treatment duration be 9 or 10 days, a maximum of 20.3 mL of blood will be drawn per subject, over the course of the study. This blood volume represents 6.34 mL/kg for a 3.2-kg infant. Actual blood volumes may vary at each institution.

If the MAD blood volume exceeds local blood volume limits, do not collect biomarker samples. Then the total MAD blood volume will be 9.35 mL (which is 2.92 mL/kg for a 3.2-kg child; 5-day dosing duration) or 11.3 mL (which is 3.53 mL/kg for a 3.2-kg child; 9- or 10-day dosing duration).

Table 8-1. Blood Volume per Body Weight in a 3.2-kg Infant

	SAD	MAD<br>5 Days of Dosing	MAD<br>9-10 Days of Dosing
Total blood volume collected	5.65 mL	16.85 mL	20.3 mL
Blood volume per kg in a 3.2 kg infant	1.77 mL/kg	5.27 mL/kg	6.34 mL/kg
Total blood volume collected if blood biomarker samples not collected	Not applicable	9.35 mL	11.3 mL
Blood volume per kg in a 3.2 kg infant if blood biomarker samples not collected	Not applicable	2.92 mL/kg	3.53 mL/kg

8.2.1 Adverse Events

Adverse events, including pretreatment events, will be recorded from the time of consent through the Completion Visit. All AEs/SAEs will be coded using the *Medical Dictionary for Regulatory Activities (MedDRA)*.

8.2.2 Medical History

All medical history will be coded using the Medical Dictionary for Regulatory Activities (MedDRA).

8.2.3 Clinical Laboratory Measurements

Clinical laboratory variables, including hematology and serum chemistries will be assessed at screening and periodically during the study as indicated in this protocol. Required laboratory variables are defined in Table 8-2, *Clinical Laboratory Evaluations*.

Table 8-2. Clinical Laboratory Evaluations

Chemistries:	Hematology (CBC):
Alkaline phosphatase	Hematocrit
ALT	Hemoglobin
AST	MCH
Bicarbonate	MCHC
BUN	MCV
Chloride	MPV
Creatine Kinase (CK)	Platelet count
Creatinine	RDW
Glucose	Red blood cell count
Potassium	Reticulocytes
Sodium	White blood cell count
Total bilirubin (direct and indirect)	White blood cell differentiation
	(Percentage and absolute):
	Basophils
	Eosinophils
	Lymphocytes
	Monocytes
	Neutrophils

Sites should attempt to collect all safety labs listed in Table 8-2 as defined in this protocol. In some instances, individual laboratory assessments in Table 8-2 are not routinely run at a local site. If it is not practical to obtain the results for a particular assessment due to local considerations the Sponsor Medical Monitor may on a case by case basis permit individual sites to not collect that assessment.

8.2.4 Prior and Concomitant Medications

Use of all medications and supportive therapy from the date the informed consent is signed through study completion will be recorded. All concomitant medications will be mapped using the World Health Organization (WHO) Drug Dictionary.

8.3 PHARMACOKINETIC MEASUREMENTS

Postdose whole blood samples will be collected from subjects in all dosing cohorts at specified times throughout the study for the determination of PK parameters (time points specified in *Schedule of Events*) for ALS-008112 and ALS-008144 (and other metabolites, if applicable).

Whole blood concentrations of ALS-008112 and ALS-008144 (and other metabolites, if applicable) will be assessed by a liquid chromatography tandem mass spectrometry method and used to calculate the values of PK parameters, including:

$AUC_{0\text{-}inf}$	Area under concentration-time curve from time zero to infinity
$AUC_{0\text{-}last}$	Area under the concentration-time curve from time zero to last quantifiable concentration
$AUC_{0\text{-}12h}$	Area under concentration-time curve from time zero to 12 hours postdose
$AUC_{0\text{-}24h}$	Area under concentration-time curve from time zero to 24 hours postdose
$AUC_{0\text{-}tau}$	Area under the concentration-time curve during the dosing interval
C_{last}	Last measurable blood concentration
C_{max}	Maximum observed concentration
C_{min}	Minimum observed concentration (for repeated doses)
Λz	terminal elimination rate constant
$t_{1/2}$	Terminal elimination half-life
t_{last}	Time to last measurable blood concentration
t_{max}	Time of maximum concentration

Nasal samples may be analyzed to determine exposures of ALS-008112 and ALS-008144 (and other metabolites, if applicable) if a sensitive method can be established.

8.4 PHARMACODYNAMIC MEASUREMENTS

Samples for PD measurements will be collected for assessment by qualitative and quantitative PCR as outlined in Table 6-1, *Schedule of Events SAD Phase (Part 1)*, Table 6-2, *Schedule of Events MAD Phase (Part 2) Twice Daily Dosing* and Table 6-3, *Schedule of Events MAD Phase (Part 2) Once Daily Dosing*.

Qualitative measures (i.e., PCR assay) will confirm the local RSV results and determine whether coinfection with other detectable virus(es) has occurred.

Quantitative PCR determines the absolute viral load of RSV RNA and can be used to determine what effect exposure to ALS-008176 has on RSV viral kinetics.

Nasal secretion samples will be collected for monitoring of potential viral resistance to ALS-008176 by population sequencing of the RSV polymerase coding region (amino acids 550–1100 of the RSV L protein) at baseline, during treatment and posttreatment with ALS-008176. Other regions of the RSV genome may also be sequenced. Specific RSV polymerase amino acid sequence variations detected in samples from a majority of treated subjects will then be selected for the generation of site-directed mutants in the RSV reverse-genetics system. The inhibitory potency of ALS-008112 will then be determined vs. the RSV site-directed mutants compared to a wild-type RSV.

See also Section 6.1.5, *RSV Evaluations* for test descriptions.

9.0 STATISTICAL CONSIDERATIONS

9.1 STUDY DESIGN AND OBJECTIVES

This randomized, double-blind, placebo-controlled, 2-part study will assess the safety, tolerability, pharmacokinetics, and pharmacodynamics (PD) of single and multiple doses of orally administered ALS-008176 in neonates and infants hospitalized with RSV infection. Each subject will only be enrolled in a single cohort and a single part of the study. Within each dosing cohort, subjects will be randomized to receive either ALS-008176 or placebo in a ratio of 3 ALS-008176: 1 placebo. Randomization (except in the neonate cohort) will be stratified by age at time of hospital admission (≥1.0 to <2.0 months =28 to 59 days old, ≥2.0 to <6.0 months =60 to 181 days old, and ≥6.0 to ≤12.0 months =182 to 365 days old).

The primary objective of this trial is to evaluate the safety and tolerability of single and multiple doses of ALS-008176. Secondary objectives are as follows:

- To evaluate the pharmacokinetics of ALS-008112 and ALS-008144 (and other metabolites, if applicable) in blood following single and multiple doses of ALS-008176
- To evaluate the antiviral activity of ALS-008176 after single and multiple doses
- To determine if ALS-008176 exposure results in the emergence of resistant strains of RSV

The following exploratory objectives will also be addressed:

- To evaluate the impact of ALS-008176 on the clinical course of RSV infection
- To evaluate the relationship between viral kinetics and clinical outcomes
- To evaluate the impact of ALS-008176 on biomarkers potentially associated with the inflammatory response induced by acute RSV infection
- To evaluate the pharmacokinetics of ALS-008112 and ALS-008144 (and other metabolites, if applicable) in nasal swabs following multiple doses of ALS-008176, if an acceptable testing methodology can be established

9.2 GENERAL CONSIDERATIONS

It is anticipated that all statistical summaries will be performed using SAS Version 9.3 (SAS Institute, Inc., Cary, NC, USA) or higher. Additional software may be used for the production of graphics.

Continuous data will be summarized by descriptive statistics, including number of subjects, mean, standard deviation, median, minimum, and maximum. Categorical data will be summarized by the number and percentage of subjects. All analyses will be presented by dose group and the combined strata. Stratified analyses, as appropriate, will be conducted if permitted by the sample size. In the final analysis, placebo subjects from the dose groups and/or age strata being summarized will be pooled to form a larger comparative group.

A Statistical Analysis Plan (SAP) will be created for the final analysis of this study with complete details and definitions of all planned summaries. Strategies for handling missing, unused, or spurious data will be specified in the SAP.

Since all primary and secondary objectives in this trial are focused on safety, pharmacokinetics, and PD, all analyses and summaries will be based on treatment received rather than treatment randomized (should they be different).

The trial will be blinded to both the site and the subjects' families. The Sponsor will be blinded to all individual infant data, but will have access to unblinded aggregate summaries at the end of each cohort, as well as any safety results of concern during study conduct.

9.3 STUDY ENDPOINTS

9.3.1 Primary Endpoint

- Safety data including, but not limited to, adverse events, physical examinations, vital signs, 12-lead ECGs and clinical laboratory results (including chemistry and hematology)

9.3.2 Secondary Endpoints

- PK parameters of ALS-008112 and ALS-008144 (and other metabolites, as applicable) in blood following single dose administration: C_{max}, t_{max}, $t_{1/2}$, $AUC_{0\text{-}24h}$, $AUC_{0\text{-}inf}$ or $AUC_{0\text{-}last}$
- PK parameters of ALS-008112 and ALS-008144 (and other metabolites as applicable) in blood following repeat dose administration: C_{max}, C_{min}, t_{max}, $t_{1/2}$, $AUC_{0\text{-}12h,}$ $AUC_{0\text{-}24h}$, $AUC_{0\text{-}tau}$, $AUC_{0\text{-}inf}$ or $AUC_{0\text{-}last}$
- RSV viral RNA concentrations in nasal swabs or aspirates as measured by quantitative RT-PCR
- Changes in RSV polymerase that result in reduced sensitivity to ALS-008112

9.3.3 Exploratory Endpoints

- Length of hospital stay
- Need and duration of supplemental oxygen requirement
- Need and duration of ICU stay
- Need and duration of mechanical ventilation
- Need and duration of non-invasive ventilator support
- Time to resolution of RSV signs or symptoms, such as runny nose, wheeze, cough, tachypnea

- Relationship between viral kinetics and various clinical outcome measures (e.g., the relationship between RSV RNA viral load and oxygen supplementation, duration of hospitalization)
- Biomarkers potentially associated with the inflammatory response induced by acute RSV infection
- Concentrations of ALS-008112 and ALS-008144 (and other metabolites as applicable) in nasal secretions if an acceptable testing methodology can be established

9.4 DETERMINATION OF SAMPLE SIZE

Up to 260 subjects will be enrolled in the study.

For Part 1 (SAD), up to 72 subjects were to enroll in SAD cohorts. As of 25 April 2016, 70 subjects have been enrolled in the SAD portion of the study, which is now closed.

For Part 2 (MAD), as of 5 August 2016, 52 subjects have enrolled across 5 MAD cohorts, some of which (e.g., 30/10) are actively recruiting (see Figure 1-1). Up to an additional 100 subjects will enroll in up to 6 additional cohorts. The maximum anticipated enrollment in the MAD portion of the study, taking into account these numbers as well as anticipated enrollment in ongoing cohorts during the protocol version 6 review process is 190 subjects (52 + up to 23 additional neonates + up to 15 additional subjects at 30/10 dose level + 100 additional subjects in future cohorts).

9.5 RANDOMIZATION

In each cohort of both Part 1 (SAD) and Part 2 (MAD), a stratified randomization will be used on the basis of age at the time of hospital admission, with the 3 strata being defined as follows: (≥1.0 to <2.0 months =28 to 59 days old, ≥2.0 to <6.0 months =60 to 181 days old, and ≥6.0 to ≤12.0 months =182 to 365 days old). Each stratum within each ascending dose cohort will consist of approximately 8 and up to 16 infants being randomized to receive ALS-008176 or placebo in a 3:1 ratio.

Effective after approval of protocol version 6, the following characteristics of cohorts may be implemented, subject to approval by the IDMC:

- Up to 6 additional cohorts may be enrolled with a maximum of 100 subjects enrolled across these cohorts
- The size of each cohort and the frequency at which cohort data are reviewed will be determined by the IDMC

If multiple subjects are eligible for randomization on the same day to the last slot within a stratum/cohort, then up to an additional 4 subjects will be allowed to be randomized on that day to that stratum/cohort (3:1 allocation).

No stratification for age will be performed in the neonate cohort. An age de-escalation approach will be utilized within each SAD dose cohort, i.e., subjects aged ≥6.0 to ≤12.0 months will be enrolled initially, followed by subjects ≥2.0 to <6.0 months, and then subjects ≥1.0 to <2.0 months, as follows:

- A sentinel group of 3 subjects in each age stratum will be enrolled first (randomized in a ratio of 2 ALS-008176:1 placebo).
- The IDMC will review the safety data through Day 7 and available PK data for the sentinel subjects. Following this:
  - The remaining 5 subjects in the age stratum will be enrolled (randomized in a ratio of 4 ALS-008176:1 placebo), and
  - Enrollment in the next age stratum's sentinel cohort(s) will be initiated.

At no time will dose escalation occur for a younger age stratum before dosing in an older age stratum has been initiated for that dose, unless the IDMC determined that the safety profile supported such an approach.

If a subject discontinues prematurely due to a non-safety related reason, that subject may be replaced.

As part of the randomization schedule for treatment assignment, all subjects will also be randomized to a PK sample collection schedule within each of the 2 treatment groups in each of the 3 age strata. The pharmacokinetics of ALS-008112 and ALS-008144 (and other metabolites, if applicable) will be determined using a sparse sampling strategy whereby each subject within an age stratum will be assigned to have PK samples drawn at specific time points which, when combined across subjects, will result in full PK profiles for the stratum.

The subjects within a single age stratum and cohort will be randomized to a PK sample collection schedule in the following ratio: 3 ALS-008176 PK Group A:3 ALS-008176 PK Group B: 1 Placebo PK Group A: 1 Placebo PK Group B.

Among the 2 subjects randomized to ALS-008176 treatment in each sentinel group, 1 subject each will be randomized to PK Group A and PK Group B.

9.6 SUBJECT DISPOSITION

An accounting of all subjects over the course of the study including enrollment, study drug administration, subject completion, premature discontinuation, and major protocol violations will be tabulated and summarized by dose cohort. If warranted by the data, further breakdowns by age strata will be provided.

9.7 ANALYSIS DATA SETS

The Full Analysis Data Set (FA) is defined to include all enrolled subjects who have received at least one dose of study medication.

The PK Population will include all subjects in the FA population who received ALS-008176 and who have at least one PK specimen collected and assayed after study drug administration.

The PK Per Protocol population is defined as all subjects in the PK population who missed <2 doses and received all doses within 6 hours of scheduled administration times.

9.8 DEMOGRAPHICS AND BASELINE CHARACTERISTICS

Demographic data (age, sex, ethnicity, body weight) and baseline disease characteristics will be tabulated and summarized by dose cohort and presented in data listings.

9.9 INTERIM ANALYSIS

Interim analyses may be conducted during the conduct of the study if deemed necessary for reasons such as regulatory reporting requirements, Sponsor decision making, etc. An unblinded Sponsor team may review efficacy data on an ongoing basis. Study team members involved in study management will remain blinded to individual subject information and not participate in data reviews which could potentially unblind them.

9.10 SAFETY ANALYSES

9.10.1 Adverse Events

The MedDRA medical dictionary will be used to map the AE/SAE verbatim terms to specific system organ classes (SOC) and preferred terms. Adverse events and SAEs will be summarized by treatment received in summary tables and tabulated in by-subject listings by SOC and preferred term for each dosing cohort, with a further breakdown by age stratum. Placebo subjects will be pooled across dosing cohorts. Incidence rates will be presented.

An adverse event is considered associated with the use of the study drug if the attribution is related or possibly related by the definitions listed in Section 7.2.3, *Assigning Attribution of Adverse Events*.

9.10.2 Death

The number and percent of subjects who died will be summarized by dose group and cause of the death. A data listing will also be provided.

9.10.3 Vital Signs, Physical Examination, and Laboratory Assessments

Vital signs, including resting pulse, blood pressure, and body temperature, will be summarized and tabulated by dosing cohort and for all subjects combined. The proportion of

subjects with abnormal findings based on physical examinations over time will be listed by dosing cohort, by age stratum, and for all subjects combined by treatment received. Laboratory values will be graded and summarized based on the Division of AIDS Table for Grading the Severity of Adult and Pediatric AEs. Number and percentage of subjects with Grades 3 and 4 laboratory abnormalities will be tabulated by dosing cohort, by age stratum, and for all subjects combined by treatment received.

9.10.4 Prior and Concomitant Medications

Use of all medications administered from the date the informed consent is signed through to the Completion Visit will be recorded. All reported concomitant medications will be mapped using the WHO Drug Dictionary. Concomitant medications will be tabulated in summary tables and by-subject listings.

9.11 PHARMACOKINETIC ANALYSIS

The calculated values for all PK parameters will be tabulated by subject, by age stratum, and by dosing cohort, and the PK profile of study medication will be presented as graphs and by-subject listings. Summary statistics will be computed by dosing cohort by treatment and by age stratum.

9.12 PHARMACODYNAMIC ANALYSIS

Changes in PD endpoints will be examined as they relate to dose, response, and toxicity of study medication.

Changes in RSV RNA concentration will be listed, tabulated and presented in graphs by treatment assignment, by dosing cohort, age stratum, and by individual. All individual values will be presented in subject listings. Generally medians will be used in the aggregate summaries.

Changes in RSV polymerase that potentially result in reduced sensitivity to ALS-008112 will be listed.

10.0 ADMINISTRATIVE CONSIDERATIONS

The investigator and/or Sponsor, consistent with local regulatory practice, will submit this protocol, the informed consent, investigator's brochure, and any other relevant supporting information to the competent authority and appropriate IEC for review and approval prior to study initiation. A letter confirming IEC approval of the protocol and informed consent, a statement that the IEC is organized and operates according to GCP and the applicable laws and regulations **must** be forwarded to Alios BioPharma prior to screening subjects for the study. Additionally, sites must forward a signed Investigator Obligation Form to Alios BioPharma **prior to** screening subjects for study enrollment. Amendments to the protocol must also be approved by the IEC and local regulatory agency, as appropriate, prior to the implementation of changes in this study.

10.1 STUDY COMPLIANCE

The study will be conducted in compliance with this protocol, principles of ICH GCP, Declaration of Helsinki, and all applicable national regulations governing clinical trials.

10.2 INFORMED CONSENT AND PROTECTED SUBJECT HEALTH INFORMATION AUTHORIZATION

A copy of the IEC-approved informed consent must be forwarded to Alios BioPharma for regulatory purposes. The investigator or designee **must** explain to the subject's parent(s)/guardian(s) the purpose and nature of the study, the study procedures, the possible adverse effects, and all other elements of consent as defined in § 21CFR Part 50, EU regulations (for EU sites), and other applicable national and local regulations governing informed consent. Each subject's parent(s)/guardian(s) must provide a signed and dated informed consent prior to enrollment into this study. Signed consent forms must remain in each subject's study file and be available for verification by study monitors at any time.

In accordance with individual local and national subject privacy regulations, the investigator or designee **must** explain to each subject's parent(s)/guardian(s) prior to screening that for the evaluation of study results, the subject's protected health information obtained during the study may be shared with Alios BioPharma and its designees and/or partners, regulatory agencies, and IECs. As the study Sponsor, Alios BioPharma will not use the subject's protected health information or disclose it to a third party without applicable subject's parent(s)/guardian(s) authorization. It is the investigator's or designee's responsibility to obtain written permission to use protected health information from each subject, or if appropriate, the subject's legal guardian. If a subject or subject's legal guardian withdraws permission to use protected health information, it is the investigator's responsibility to obtain the withdrawal request in writing from the subject or subject's legal guardian **and** to ensure that no further data will be collected from the subject. Any data collected on the subject prior to withdrawal will be used in the analysis of study results.

10.3 SUBJECT SCREENING LOG

The investigator **must** keep a record that lists **all** subjects considered for screening in the study. For those subjects subsequently excluded, record the reason(s) for exclusion.

10.4 CASE REPORT FORMS

Study site personnel will complete CRFs designed for this study according to the completion guidelines that will be provided. An electronic CRF (eCRF) will be used for this study. Study site personnel will be trained and authorized to use the system in compliance with 21CFR Part 11 prior to recording data on eCRFs. All corrections to eCRFs will be made by authorized users, and the changes will be automatically logged in the system.

The investigator will ensure that the eCRFs are accurate, complete, legible, and completed in a timely fashion. Separate source records are required to support all eCRF entries. The eCRF

is not to be used to document data without prior written or electronic records. Case report forms should be completed for every subject enrolled in the study. At the study's conclusion, a PDF file will be created for each site containing their subjects' data submitted on eCRFs. In the event of an audit or regulatory authority inspection, copies of the eCRFs will be printed.

10.5 STUDY MONITORING REQUIREMENTS

Representatives of Alios BioPharma or its designee will monitor this study until completion. Monitoring will be conducted through personal visits with the investigator and site staff as well as any appropriate communications by mail, fax, e-mail, or telephone. The purpose of monitoring is to ensure compliance with the protocol and the quality and integrity of the data. This study is also subject to Quality Assurance reviews and/or audits under the Alios BioPharma Clinical Quality Assurance program.

Every effort will be made to maintain the anonymity and confidentiality of all subjects during this clinical study. However, because of the experimental nature of this treatment, the investigator agrees to allow the IEC, representatives of Alios BioPharma, its designated agent, and authorized employees of the appropriate regulatory agencies to inspect the facilities used in this study and, for purposes of verification, allows direct access to the hospital or clinic records of all subjects enrolled into this study. A statement to this effect will be included in the informed consent and permission form authorizing the use of protected health information.

10.6 RETENTION OF RECORDS

The investigator must retain a copy of all documents relating to this clinical trial for a minimum of 5 years after a marketing application is approved for the drug, unless Alios BioPharma notifies the investigator in writing that the documents no longer need to be retained because a marketing application will not be filed. The investigator must retain the documents for a longer period, where so required by other applicable requirements. Essential documents shall be archived in a way that ensures that they are readily available, upon request, to the competent authorities and appropriate regulatory authorities. The medical files of trial subjects shall be retained in accordance with national legislation and the maximum period of time permitted by the hospital, institution, or private practice. The investigator is responsible for contacting Alios BioPharma before any study-related documents are moved to another location or destroyed, and he or she must receive written approval from Alios BioPharma before such relocation or destruction of documents proceeds.

10.7 CONFIDENTIALITY AND PUBLICATION POLICY

By conducting this study, the investigator affirms to Alios BioPharma that all study results and information furnished by Alios BioPharma will be maintained in strict confidence. Such information will be communicated to the investigator's IEC under an appropriate understanding of confidentiality.

A published summary of the results of this study is, however, permissible according to Alios BioPharma and is not inconsistent with the preceding affirmation of confidentiality. Any publication of data collected as a result of this study will be considered a joint publication by the investigator and appropriate Alios BioPharma personnel. Authorship, including order, will be determined by Alios BioPharma in consultation with the principal investigator. Contribution of the author to the study design, enrollment, data review, and manuscript preparation and review will be considered when determining the order of authorship for multicenter studies. Alios BioPharma **must** receive a copy of any presentation, manuscript, or abstract for review **at least 45 days prior** to public presentation or submission for publication. Any publication outside of this agreement is not permitted.

10.8 CONDUCT OF STUDY AND PROTECTION OF HUMAN SUBJECTS

The principal investigator must ensure the following (unless Sponsor is required per local regulations):

1. He or she will personally conduct or supervise the study.
2. His or her staff and all persons who assist in the conduct of the study clearly understand their responsibilities and have their names included in the Study Staff Signature and Delegation of Authority log. The investigator will sign the authorization log whenever it is updated with new responsibilities or staff membership.
3. The study is conducted according to the protocol and all applicable regulations.
4. The protection of each subject's rights and welfare is maintained.
5. Signed and dated informed consent and permission to use protected health information are obtained from each subject's parent(s)/guardian(s) prior to conducting study procedures. If a subject or subject's [parent or] legal guardian withdraws permission to use protected health information, the investigator will obtain a written request from the subject or subject's [parent or] legal guardian and will ensure that no further data be collected from the subject.
6. The consent process is conducted in compliance with all applicable regulations and privacy acts.
7. The IEC complies with applicable regulations and conducts initial and ongoing reviews and approvals of the study.
8. Any amendment to the protocol is submitted promptly to the competent authority and IEC.
9. Any significant protocol deviations are reported to Alios BioPharma, the local competent authority and the IEC according to the guidelines at each study site.
10. All Safety Reports are submitted promptly to the local competent authority and IEC in accordance with the institution's internal policy.

11. All SAEs are reported to INVENTIV HEALTH Global Safety and Pharmacovigilance within 24 hours of knowledge of the event, and to the local competent authority and the IEC.

11.0 REFERENCES

1. [AAP] American Academy of Pediatrics, Committee on Infectious Diseases and Committee on Fetus and Newborn. Prevention of respiratory syncytial virus infections: indications for the use of palivizumab and update on the use of RSVIGIV. Pediatrics 1998;102:1211–6.
2. Bloom-Feshbach K, Alonso WJ, Charu V, et al. Latitudinal variations in seasonal activity of influenza and respiratory syncytial virus (RSV): A global comparative review. PLoS ONE 2013;8(2):e54445.
3. Hall CB. Respiratory syncytial virus and parainfluenza virus. N Engl J Med 2001;344:1917–28.
4. Howie SRC. Blood sample volumes in child health research: review of safe limits. Bull World Health Organ 2011;89:46–53.
5. Nair H, Nokes DJ, Gessner BD, et al. Global burden of acute lower respiratory infections due to respiratory syncytial virus in young infants: a systematic review and meta-analysis. Lancet 2010;375(9725):1545–55.
6. Perkins SM, Webb DL, Torrance SA, et al. Comparison of a real-time reverse transcriptase PCR assay and a culture technique for quantitative assessment of viral load in children naturally infected with respiratory syncytial virus. J Clin Microbiol 2005;43:2356–62.
7. Synagis® (palivizumab) for intramuscular administration. Package Insert issued April 2012. MedImmune, LLC Gaithersburg, MD.
8. Yusuf S, Piedimonte G, Auais A, et al. The relationship of meteorological conditions to the epidemic activity of respiratory syncytial virus. Epidemiol Infect 2007;135:1077–90.

12.0 APPENDICES

Appendix A. Investigator Signature Page 84

Appendix B. Estimated Blood Volumes 85

Appendix C. Division of AIDS Tables for Grading the Severity of Adult and Pediatric Adverse Events. Publish Date: December 2004, Clarification August 2009 89

Appendix D. Summary of Protocol Changes from Version 1.0 dated 12 December 2013 to Version 2.0 111

Appendix E. Summary of Protocol Changes from Version 2.0 dated 19 December 2013 to Version 3.0 121

Appendix F. Summary of Protocol Changes from Version 3.0 dated 17 December 2014 to Version 4.0 133

Appendix G. Summary of Protocol Changes from Version 4.0 dated 24 August 2015 to Version 5.0 155

Appendix H. Summary of Protocol Changes from Version 5.0 dated 22 January 2016 to Version 6.0 169

Appendix A. Investigator Signature Page

STUDY ACKNOWLEDGEMENT

A Randomized, Double-blind, Placebo-controlled, 2-Part Study of Orally Administered ALS-008176 to Evaluate the Safety, Tolerability, Pharmacokinetics and Pharmacodynamics of Single Ascending Dosing and Multiple Ascending Dosing in Infants Hospitalized with Respiratory Syncytial Virus (RSV) Infection

VERSION 6.0, 13 September 2016

This protocol has been approved by Alios BioPharma Inc. The following signature documents this approval.

**Name (Printed)**	**Signature**
**Date**	

INVESTIGATOR STATEMENT

I have read the protocol, including all appendices, and I agree that it contains all necessary details for me and my staff to conduct this study as described. I will conduct this study as outlined in *Section 10.8, Conduct of Study and Protection of Human Subjects*, and will make every effort to complete the study within the time designated.

**Principal Investigator Name (Printed)**	**Signature**
**Date**	**Site Name**

Appendix B. Estimated Blood Volumes

Appendix Table 12-1. Maximum Estimated Blood Volumes (SAD Part 1 PK Group A)*

		Hospitalized Study Days				Completion Visit
Days	Screen	Day 1		Day 2	Days 3 to 6	Day 7 (±1 day)
Assessments	Day -3 to 1	0.5-1.0 hours postdose	3 (±0.5) hours postdose	24 (±2) hours postdose	QAM	
PK Samples Group A 0.2 mL each	Optional^	0.2	0.2	Only if blood collected for other standard care reasons is available for extra PK and safety analysis		Optional^
Serum Chemistries 1 mL each	1		1			1
Hematology + Reticulocytes 0.75 mL each	0.75		0.75			0.75
Total	1.75	0.2	1.95			1.75
**Study Total**	**5.65 mL**					

*Sites must consider the volume requirements of their local laboratory for serum chemistry, CBC with differential, and reticulocytes when considering the actual blood volume totals.

^ These PK assessments will only be performed if sufficient blood volume remains after performing other blood analyses.

Appendix Table 12-2. Maximum Estimated Blood Volumes (SAD Part 1 PK Group B)*

		Hospitalized Study Days				Completion Visit
**Days**	**Screen**	**Day 1**		**Day 2**	**Days 3 to 6**	**Day 7 (±1 day)**
**Assessments**	**Day -3 to 1**	**0.5-1.0 hours postdose**	**7 (±2) hours postdose**	**24 (±2) hours postdose**	**QAM**	
PK Samples Group B 0.2 mL each	Optional^	0.2	0.2	Only if blood collected for other standard care reasons is available for extra PK and safety analysis		Optional^
Serum Chemistries 1 mL each	1		1			1
Hematology + Reticulocytes 0.75 mL each	0.75		0.75			0.75
Total	1.75	0.2	1.95			1.75
**Study Total**	**5.65 mL**					

*Sites must consider the volume requirements of their local laboratory for serum chemistry, CBC with differential, and reticulocytes when considering the actual blood volume totals.
^ These PK assessments will only be performed if sufficient blood volume remains after performing other blood analyses.

Appendix Table 12-3. Maximum Estimated Blood Volumes (MAD Part 2 PK Group A)*

		Hospitalized Study Days			Safety Visit	Completion Visit	Follow-Up Visit
**Days**	**Screen**	**Day 1**		**Days 3 to 10**	**Last day of dosing (-2 day)**	**Day 6 (±1) days after the last day of dosing**	**Day 28 (±7 days)**
**Assessments**	**(-3) to 1**	**0.25-2 hr Post-Dose 1 (Loading Dose)**	**0.5-1 hr Post-Dose 2 (Maintenance dose)**	**QAM**			
PK Samples: Group A 0.2 mL each	Optional^	0.2	0.2		0.2	Optional^	
Serum Chemistries 1 mL each	1		1	Only if blood collected for other standard care reasons is available for extra PK and safety analysis	1	1	1
Hematology 0.75 mL each	0.75		0.75		0.75	0.75	0.75
Biomarkers 1.5 mL each**	1.5		1.5		1.5	1.5	1.5
Total	3.25	0.2	3.45		3.45	3.25	3.25
**Study Total**	16.85 mL&						

*Sites must consider the volume requirements of their local laboratory for serum chemistry, CBC with differential, and reticulocytes when considering the actual blood volume totals.

^ These PK assessments will only be performed if sufficient blood volume remains after performing other blood analyses.

**If total blood volume for study exceeds limits defined by local regulations, blood biomarkers should be omitted.

&If the IDMC requires that the treatment duration be 9 or 10 days, the additional blood volume collected on Day 5 would result in total blood volume collected of 16.85 mL + 3.45 mL=20.3 mL

Appendix Table 12-4. Maximum Estimated Blood Volumes (MAD Part 2 PK Group B)*

Days	Screen	Hospitalized Study Days: Day 1		Hospitalized Study Days: Days 3 to 10	Safety Visit: Last day of dosing (-2 day)	Completion Visit: Day 6 (±1) days after the last day of dosing	Follow-Up Visit: Day 28 (±7 days)
**Assessments**	**(-3) to 1**	**0.25-2 hours Post-Dose 1 (LD)**	**7 (±4 hours) Post-Dose 2 (MDe)**	**QAM**			
PK Samples: Group B 0.2 mL each	Optional^	0.2	0.2		0.2	Optional^	
Serum Chemistries 1 mL each	1		1	Only if blood collected for other standard care reasons is available for extra PK and safety analysis	1	1	1
Hematology 0.75 mL each	0.75		0.75		0.75	0.75	0.75
Biomarkers 1.5 mL each**	1.5		1.5		1.5	1.5	1.5
Total	3.25	0.2	3.45		3.45	3.25	3.25
**Study Total**	**16.85 mL[&]**						

*Sites must consider the volume requirements of their local laboratory for serum chemistry, CBC with differential, and reticulocytes when considering the actual blood volume totals.

^ These PK assessments will only be performed if sufficient blood volume remains after performing other blood analyses.

**If total blood volume for study exceeds limits defined by local regulations, blood biomarkers should be omitted.

[&]If the IDMC requires that the treatment duration be 9 or 10 days, the additional blood volume collected on Day 5 would result in total blood volume collected of 16.85 mL + 3.45 mL=20.3 mL

Appendix C. Division of AIDS Tables for Grading the Severity of Adult and Pediatric Adverse Events. Publish Date: December 2004, Clarification August 2009

The Division of AIDS Table for Grading the Severity of Adult and Pediatric Adverse Events ("DAIDS AE Grading Table") is a descriptive terminology which can be utilized for Adverse Event (AE) reporting. A grading (severity) scale is provided for each AE term.

This clarification of the DAIDS Table for Grading the Severity of Adult and Pediatric AE's provides additional explanation of the DAIDS AE Grading Table and clarifies some of the parameters.

I. Instructions and Clarifications

Grading Adult and Pediatric AEs

The DAIDS AE Grading Table includes parameters for grading both Adult and Pediatric AEs. When a single set of parameters is not appropriate for grading specific types of AEs for both Adult and Pediatric populations, separate sets of parameters for Adult and/or Pediatric populations (with specified respective age ranges) are given in the Table. If there is no distinction in the Table between Adult and Pediatric values for a type of AE, then the single set of parameters listed is to be used for grading the severity of both Adult and Pediatric events of that type.

Note: In the classification of adverse events, the term "**severe**" is not the same as "**serious**." Severity is an indication of the intensity of a specific event (as in mild, moderate, or severe chest pain). The term "**serious**" relates to a participant/event outcome or action criteria, usually associated with events that pose a threat to a participant's life or functioning.

Addenda 1-3 Grading Tables for Microbicide Studies

For protocols involving topical application of products to the female genital tract, male genital area or rectum, strong consideration should be given to using Appendices I-III as the primary grading scales for these areas. The protocol would need to specifically state that one or more of the Appendices would be primary (and thus take precedence over the main Grading Table) for items that are listed in both the Appendix and the main Grading Table.

- Addendum 1 - Female Genital Grading Table for Use in Microbicide Studies - PDF
- Addendum 2 - Male Genital Grading Table for Use in Microbicide Studies - PDF
- Addendum 3 - Rectal Grading Table for Use in Microbicide Studies - PDF

Grade 5

For any AE where the outcome is death, the severity of the AE is classified as Grade 5.

Estimating Severity Grade for Parameters Not Identified in the Table

In order to grade a clinical AE that is not identified in the DAIDS AE grading table, use the category "Estimating Severity Grade" located on Page 3.

Determining Severity Grade for Parameters "Between Grades"

If the severity of a clinical AE could fall under either 1 of 2 grades (e.g., the severity of an AE could be either Grade 2 or Grade 3), select the higher of the 2 grades for the AE. If a laboratory value that is graded as a multiple of the ULN or LLN falls between 2 grades, select the higher of the 2 grades for the AE. For example, Grade 1 is 2.5 × ULN and Grade 2 is 2.6 × ULN for a parameter. If the lab value is 2.53 × ULN (which is between the 2 grades), the severity of this AE would be Grade 2, the higher of the 2 grades.

Values Below Grade 1

Any laboratory value that is between either the LLN or ULN and Grade 1 should not be graded.

Determining Severity Grade when Local Laboratory Normal Values Overlap with Grade 1 Ranges

In these situations, the severity grading is based on the ranges in the DAIDS AE Grading Table, even when there is a reference to the local lab LLN.

For example: Phosphate, Serum, Low, Adult and Pediatric >14 years (Page 20) Grade 1 range is 2.50 mg/dL - < LLN. A particular laboratory's normal range for Phosphate is 2.1 – 3.8 mg/dL. A participant's actual lab value is 2.5. In this case, the value of 2.5 exceeds the LLN for the local lab, but will be graded as Grade 1 per DAIDS AE Grading Table.

II. Definitions of terms used in the Table:

Basic Self-care Functions	Adult<br>Activities such as bathing, dressing, toileting, transfer/movement, continence, and feeding.
Young Children<br>Activities that are age and culturally appropriate (e.g., feeding self with culturally appropriate eating implement).	
LLN	Lower limit of normal
Medical Intervention	Use of pharmacologic or biologic agent(s) for treatment of an AE.
NA	Not Applicable
Operative Intervention	Surgical OR other invasive mechanical procedures.
ULN	Upper limit of normal
Usual Social & Functional Activities	Adult<br>Adaptive tasks and desirable activities, such as going to work, shopping, cooking, use of transportation, pursuing a hobby, etc.
Young Children<br>Activities that are age and culturally appropriate (e.g., social interactions, play activities, learning tasks, etc).	

PARAMETER	GRADE 1<br>MILD	GRADE 2<br>MODERATE	GRADE 3<br>SEVERE	GRADE 4<br>POTENTIALLY LIFE-THREATENING
**ESTIMATING SEVERITY GRADE**				
Clinical adverse event NOT identified elsewhere in this DAIDS AE Grading Table	Symptoms causing no or minimal interference with usual social & functional activities	Symptoms causing greater than minimal interference with usual social & functional activities	Symptoms causing inability to perform usual social & functional activities	Symptoms causing inability to perform basic self-care functions OR Medical or operative intervention indicated to prevent permanent impairment, persistent disability, or death
**SYSTEMIC**				
Acute systemic allergic reaction	Localized urticaria (wheals) with no medical intervention indicated	Localized urticaria with medical intervention indicated OR Mild angioedema with no medical intervention indicated	Generalized urticaria OR Angioedema with medical intervention indicated OR Symptomatic mild bronchospasm	Acute anaphylaxis OR Life-threatening bronchospasm OR laryngeal edema
Chills	Symptoms causing no or minimal interference with usual social & functional activities	Symptoms causing greater than minimal interference with usual social & functional activities	Symptoms causing inability to perform usual social & functional activities	NA
Fatigue<br>Malaise	Symptoms causing no or minimal interference with usual social & functional activities	Symptoms causing greater than minimal interference with usual social & functional activities	Symptoms causing inability to perform usual social & functional activities	Incapacitating fatigue/ malaise symptoms causing inability to perform basic self-care functions
Fever (nonaxillary)	37.7 – 38.6°C	38.7 – 39.3°C	39.4 – 40.5°C	> 40.5°C
Pain (indicate body site)<br>DO NOT use for pain due to injection (See Injection Site Reactions: Injection site pain)<br>See also Headache, Arthralgia, and Myalgia	Pain causing no or minimal interference with usual social & functional activities	Pain causing greater than minimal interference with usual social & functional activities	Pain causing inability to perform usual social & functional activities	Disabling pain causing inability to perform basic self-care functions OR Hospitalization (other than emergency room visit) indicated

Basic Self-care Functions – Adult: Activities such as bathing, dressing, toileting, transfer/movement, continence, and feeding.
Basic Self-care Functions – Young Children: Activities that are age and culturally appropriate (e.g., feeding self with culturally appropriate eating implement).
Usual Social & Functional Activities – Adult: Adaptive tasks and desirable activities, such as going to work, shopping, cooking, use of transportation, pursuing a hobby, etc.
Usual Social & Functional Activities – Young Children: Activities that are age and culturally appropriate (e.g., social interactions, play activities, learning tasks, etc).

PARAMETER		GRADE 1 MILD	GRADE 2 MODERATE	GRADE 3 SEVERE	GRADE 4 POTENTIALLY LIFE-THREATENING
Unintentional weight loss		NA	5 – 9% loss in body weight from baseline	10 – 19% loss in body weight from baseline	≥ 20% loss in body weight from baseline OR Aggressive intervention indicated [e.g., tube feeding or total parenteral nutrition (TPN)]
**INFECTION**					
Infection (any other than HIV infection)		Localized, no systemic antimicrobial treatment indicated AND Symptoms causing no or minimal interference with usual social & functional activities	Systemic antimicrobial treatment indicated OR Symptoms causing greater than minimal interference with usual social & functional activities	Systemic antimicrobial treatment indicated AND Symptoms causing inability to perform usual social & functional activities OR Operative intervention (other than simple incision and drainage) indicated	Life-threatening consequences (e.g., septic shock)
**INJECTION SITE REACTIONS**					
Injection site pain (pain without touching) Or Tenderness (pain when area is touched)		Pain/tenderness causing no or minimal limitation of use of limb	Pain/tenderness limiting use of limb OR Pain/tenderness causing greater than minimal interference with usual social & functional activities	Pain/tenderness causing inability to perform usual social & functional activities	Pain/tenderness causing inability to perform basic self-care function OR Hospitalization (other than emergency room visit) indicated for management of pain/tenderness
Injection site reaction (localized)					
	**Adult > 15 years**	Erythema OR Induration of 5x5 cm–9x9 cm (or 25 cm^2-81 cm^2)	Erythema OR Induration OR Edema > 9 cm any diameter (or > 81 cm^2)	Ulceration OR Secondary infection OR Phlebitis OR Sterile abscess OR Drainage	Necrosis (involving dermis and deeper tissue)

Basic Self-care Functions – Adult: Activities such as bathing, dressing, toileting, transfer/movement, continence, and feeding.
Basic Self-care Functions – Young Children: Activities that are age and culturally appropriate (e.g., feeding self with culturally appropriate eating implement).
Usual Social & Functional Activities – Adult: Adaptive tasks and desirable activities, such as going to work, shopping, cooking, use of transportation, pursuing a hobby, etc.
Usual Social & Functional Activities – Young Children: Activities that are age and culturally appropriate (e.g., social interactions, play activities, learning tasks, etc).

PARAMETER	GRADE 1 MILD	GRADE 2 MODERATE	GRADE 3 SEVERE	GRADE 4 POTENTIALLY LIFE-THREATENING
**Pediatric ≤15 years**	Erythema OR Induration OR Edema present but ≤2.5 cm diameter	Erythema OR Induration OR Edema > 2.5 cm diameter but < 50% surface area of the extremity segment (e.g., upper arm/thigh)	Erythema OR Induration OR Edema involving ≥ 50% surface area of the extremity segment (e.g., upper arm/thigh) OR Ulceration OR Secondary infection OR Phlebitis OR Sterile abscess OR Drainage	Necrosis (involving dermis and deeper tissue)
Pruritis associated with injection See also Skin: Pruritis (itching - no skin lesions)	Itching localized to injection site AND Relieved spontaneously or with < 48 hours treatment	Itching beyond the injection site but not generalized OR Itching localized to injection site requiring ≥48 hours treatment	Generalized itching causing inability to perform usual social & functional activities	NA
**SKIN – DERMATOLOGICAL**				
Alopecia	Thinning detectable by study participant (or by caregiver for young children and disabled adults)	Thinning or patchy hair loss detectable by health care provider	Complete hair loss	NA
Cutaneous reaction – rash	Localized macular rash	Diffuse macular, maculopapular, or morbilliform rash OR Target lesions	Diffuse macular, maculopapular, or morbilliform rash with vesicles or limited number of bullae OR Superficial ulcerations of mucous membrane limited to one site	Extensive or generalized bullous lesions OR Stevens-Johnson syndrome OR Ulceration of mucous membrane involving two or more distinct mucosal sites OR Toxic epidermal necrolysis (TEN)
Hyperpigmentation	Slight or localized	Marked or generalized	NA	NA
Hypopigmentation	Slight or localized	Marked or generalized	NA	NA
Pruritis (itching – no skin lesions) (See also Injection Site Reactions: Pruritis associated with injection)	Itching causing no or minimal interference with usual social & functional activities	Itching causing greater than minimal interference with usual social & functional activities	Itching causing inability to perform usual social & functional activities	NA

Basic Self-care Functions – Adult: Activities such as bathing, dressing, toileting, transfer/movement, continence, and feeding.
Basic Self-care Functions – Young Children: Activities that are age and culturally appropriate (e.g., feeding self with culturally appropriate eating implement).
Usual Social & Functional Activities – Adult: Adaptive tasks and desirable activities, such as going to work, shopping, cooking, use of transportation, pursuing a hobby, etc.
Usual Social & Functional Activities – Young Children: Activities that are age and culturally appropriate (e.g., social interactions, play activities, learning tasks, etc).

PARAMETER		GRADE 1 MILD	GRADE 2 MODERATE	GRADE 3 SEVERE	GRADE 4 POTENTIALLY LIFE-THREATENING
**CARDIOVASCULAR**					
Cardiac arrhythmia (general)<br>(By ECG or physical exam)		Asymptomatic AND No intervention indicated	Asymptomatic AND Non-urgent medical intervention indicated	Symptomatic, non-life-threatening AND Non-urgent medical intervention indicated	Life-threatening arrhythmia OR Urgent intervention indicated
Cardiac-ischemia/infarction		NA	NA	Symptomatic ischemia (stable angina) OR Testing consistent with ischemia	Unstable angina OR Acute myocardial infarction
Hemorrhage (significant acute blood loss)		NA	Symptomatic AND No transfusion indicated	Symptomatic AND Transfusion of ≤2 units packed RBCs (for children ≤10 cc/kg) indicated	Life-threatening hypotension OR Transfusion of > 2 units packed RBCs (for children > 10 cc/kg) indicated
**Hypertension**					
	**Adult > 17 years**<br>(with repeat testing at same visit)	140–159 mmHg systolic OR 90–99 mmHg diastolic	160–179 mmHg systolic OR 100–109 mmHg diastolic	≥ 180 mmHg systolic OR<br>≥ 110 mmHg diastolic	Life-threatening consequences (e.g., malignant hypertension) OR Hospitalization indicated (other than emergency room visit)
	**Correction**: in Grade 2 to 160 - 179 from > 160-179 (systolic) and to ≥ 100 -109 from > 100-109 (diastolic) and in Grade 3 to ≥ 180 from > 180 (systolic) and to ≥ 110 from > 110 (diastolic).				
	**Pediatric ≤ 17 years**<br>(with repeat testing at same visit)	NA	91st – 94th percentile adjusted for age, height, and gender (systolic and/or diastolic)	≥ 95th percentile adjusted for age, height, and gender (systolic and/or diastolic)	Life-threatening consequences (e.g., malignant hypertension) OR Hospitalization indicated (other than emergency room visit)
Hypotension		NA	Symptomatic, corrected with oral fluid replacement	Symptomatic, IV fluids indicated	Shock requiring use of vasopressors or mechanical assistance to maintain blood pressure
Pericardial effusion		Asymptomatic, small effusion requiring no intervention	Asymptomatic, moderate or larger effusion requiring no intervention	Effusion with non-life threatening physiologic consequences OR Effusion with non-urgent intervention indicated	Life-threatening consequences (e.g., tamponade) OR Urgent intervention indicated

Basic Self-care Functions – Adult: Activities such as bathing, dressing, toileting, transfer/movement, continence, and feeding.
Basic Self-care Functions – Young Children: Activities that are age and culturally appropriate (e.g., feeding self with culturally appropriate eating implement).
Usual Social & Functional Activities – Adult: Adaptive tasks and desirable activities, such as going to work, shopping, cooking, use of transportation, pursuing a hobby, etc.
Usual Social & Functional Activities – Young Children: Activities that are age and culturally appropriate (e.g., social interactions, play activities, learning tasks, etc).

PARAMETER		GRADE 1 MILD	GRADE 2 MODERATE	GRADE 3 SEVERE	GRADE 4 POTENTIALLY LIFE-THREATENING
Prolonged PR interval					
	**Adult > 16 years**	PR interval 0.21 – 0.25 sec	PR interval > 0.25 sec	Type II 2nd degree AV block OR Ventricular pause > 3.0 sec	Complete AV block
	**Pediatric ≤ 16 years**	1st degree AV block (PR > normal for age and rate)	Type I 2nd degree AV block	Type II 2nd degree AV block	Complete AV block
Prolonged QTc					
	**Adult > 16 years**	Asymptomatic, QTc interval 0.45–0.47 sec OR Increase interval < 0.03 sec above baseline	Asymptomatic, QTc interval 0.48–0.49 sec OR Increase in interval 0.03–0.05 sec above baseline	Asymptomatic, QTc interval ≥ 0.50 sec OR Increase in interval ≥ 0.06 sec above baseline	Life-threatening consequences, e.g., Torsade de pointes or other associated serious ventricular dysrhythmia
	**Pediatric ≤ 16 years**	Asymptomatic, QTc interval 0.450–0.464 sec	Asymptomatic, QTc interval 0.465–0.479 sec	Asymptomatic, QTc interval ≥ 0.480 sec	Life-threatening consequences, e.g., Torsade de pointes or other associated serious ventricular dysrhythmia
Thrombosis/ embolism		NA	Deep vein thrombosis AND No intervention indicated (e.g., anticoagulation, lysis filter, invasive procedure)	Deep vein thrombosis AND Intervention indicated (e.g., anticoagulation, lysis filter, invasive procedure)	Embolic event (e.g., pulmonary embolism, life-threatening thrombus)
Vasovagal episode (associated with a procedure of any kind)		Present without loss of consciousness	Present with transient loss of consciousness	NA	NA
Ventricular dysfunction (congestive heart failure)		NA	Asymptomatic diagnostic finding AND intervention indicated	New onset with symptoms OR Worsening symptomatic congestive heart failure	Life-threatening congestive heart failure

Basic Self-care Functions – Adult: Activities such as bathing, dressing, toileting, transfer/movement, continence, and feeding.
Basic Self-care Functions – Young Children: Activities that are age and culturally appropriate (e.g., feeding self with culturally appropriate eating implement).
Usual Social & Functional Activities – Adult: Adaptive tasks and desirable activities, such as going to work, shopping, cooking, use of transportation, pursuing a hobby, etc.
Usual Social & Functional Activities – Young Children: Activities that are age and culturally appropriate (e.g., social interactions, play activities, learning tasks, etc).

PARAMETER	GRADE 1<br>MILD	GRADE 2<br>MODERATE	GRADE 3<br>SEVERE	GRADE 4<br>POTENTIALLY LIFE-THREATENING
**GASTROINTESTINAL**				
Anorexia	Loss of appetite without decreased oral intake	Loss of appetite associated with decreased oral intake without significant weight loss	Loss of appetite associated with significant weight loss	Life-threatening consequences OR Aggressive intervention indicated [e.g., tube feeding or total parenteral nutrition (TPN)]
**Comment:** Please note that, while the grading scale provided for Unintentional Weight Loss may be used as a guideline when grading anorexia, this is not a requirement and should not be used as a substitute for clinical judgment.				
Ascites	Asymptomatic	Symptomatic AND Intervention indicated (e.g., diuretics or therapeutic paracentesis)	Symptomatic despite intervention	Life-threatening consequences
Cholecystitis	NA	Symptomatic AND Medical intervention indicated	Radiologic, endoscopic, or operative intervention indicated	Life-threatening consequences (e.g., sepsis or perforation)
Constipation	NA	Persistent constipation requiring regular use of dietary modifications, laxatives, or enemas	Obstipation with manual evacuation indicated	Life-threatening consequences (e.g., obstruction)
Diarrhea				
**Adult and Pediatric ≥ 1 year**	Transient or intermittent episodes of unformed stools OR Increase of ≤ 3 stools over baseline per 24-hour period	Persistent episodes of unformed to watery stools OR Increase of 4–6 stools over baseline per 24-hour period	Bloody diarrhea OR Increase of ≥ 7 stools per 24-hour period OR IV fluid replacement indicated	Life-threatening consequences (e.g., hypotensive shock)
**Pediatric < 1 year**	Liquid stools (more unformed than usual) but usual number of stools	Liquid stools with increased number of stools OR Mild dehydration	Liquid stools with moderate dehydration	Liquid stools resulting in severe dehydration with aggressive rehydration indicated OR Hypotensive shock

Basic Self-care Functions – Adult: Activities such as bathing, dressing, toileting, transfer/movement, continence, and feeding.
Basic Self-care Functions – Young Children: Activities that are age and culturally appropriate (e.g., feeding self with culturally appropriate eating implement).
Usual Social & Functional Activities – Adult: Adaptive tasks and desirable activities, such as going to work, shopping, cooking, use of transportation, pursuing a hobby, etc.
Usual Social & Functional Activities – Young Children: Activities that are age and culturally appropriate (e.g., social interactions, play activities, learning tasks, etc).

PARAMETER	GRADE 1 MILD	GRADE 2 MODERATE	GRADE 3 SEVERE	GRADE 4 POTENTIALLY LIFE-THREATENIN
Dysphagia-Odynophagia	Symptomatic but able to eat usual diet	Symptoms causing altered dietary intake without medical intervention indicated	Symptoms causing severely altered dietary intake with medical intervention indicated	Life-threatening reduction in oral intake
Mucositis/stomatitis (clinical exam) Indicate site (e.g., larynx, oral) See Genitourinary for Vulvovaginitis See also Dysphagia-Odynophagia and Proctitis	Erythema of the mucosa	Patchy pseudomembranes or ulcerations	Confluent pseudomembranes or ulcerations OR Mucosal bleeding with minor trauma	Tissue necrosis OR Diffuse spontaneous mucosal bleeding OR Life-threatening consequences (e.g., aspiration, choking)
Nausea	Transient (< 24 hours) or intermittent nausea with no or minimal interference with oral intake	Persistent nausea resulting in decreased oral intake for 24-48 hours	Persistent nausea resulting in minimal oral intake for > 48 hours OR Aggressive rehydration indicated (e.g., IV fluids)	Life-threatening consequences (e.g., hypotensive shock)
Pancreatitis	NA	Symptomatic AND Hospitalization not indicated (other than emergency room visit)	Symptomatic AND Hospitalization indicated (other than emergency room visit)	Life-threatening consequences (e.g., circulatory failure, hemorrhage, sepsis)
Proctitis (functional-symptomatic) Also see Mucositis/stomatitis for clinical exam	Rectal discomfort AND No intervention indicated	Symptoms causing greater than minimal interference with usual social & functional activities OR Medical intervention indicated	Symptoms causing inability to perform usual social & functional activities OR Operative intervention indicated	Life-threatening consequences (e.g., perforation)
Vomiting	Transient or intermittent vomiting with no or minimal interference with oral intake	Frequent episodes of vomiting with no or mild dehydration	Persistent vomiting resulting in orthostatic hypotension OR Aggressive rehydration indicated (e.g., IV fluids)	Life-threatening consequences (e.g., hypotensive shock)

Basic Self-care Functions – Adult: Activities such as bathing, dressing, toileting, transfer/movement, continence, and feeding.
Basic Self-care Functions – Young Children: Activities that are age and culturally appropriate (e.g., feeding self with culturally appropriate eating implement).
Usual Social & Functional Activities – Adult: Adaptive tasks and desirable activities, such as going to work, shopping, cooking, use of transportation, pursuing a hobby, etc.
Usual Social & Functional Activities – Young Children: Activities that are age and culturally appropriate (e.g., social interactions, play activities, learning tasks, etc).

PARAMETER	GRADE 1 MILD	GRADE 2 MODERATE	GRADE 3 SEVERE	GRADE 4 POTENTIALLY LIFE-THREATENING
**NEUROLOGIC**				
Alteration in personality-behavior or in mood (e.g., agitation, anxiety, depression, mania, psychosis)	Alteration causing no or minimal interference with usual social & functional activities	Alteration causing greater than minimal interference with usual social & functional activities	Alteration causing inability to perform usual social & functional activities	Behavior potentially harmful to self or others (e.g., suicidal & homicidal ideation or attempt, acute psychosis) OR Causing inability to perform basic self-care functions
Altered Mental Status For Dementia, see Cognitive & behavioral/ attentional disturbance (including dementia & attention deficit disorder)	Changes causing no or minimal interference with usual social & functional activities	Mild lethargy or somnolence causing greater than minimal interference with usual social & functional activities	Confusion, memory impairment, lethargy, or somnolence causing inability to perform usual social & functional activities	Delirium OR obtundation, OR coma
Ataxia	Asymptomatic ataxia detectable on exam OR Minimal ataxia causing no or minimal interference with usual social & functional activities	Symptomatic ataxia causing greater than minimal interference with usual social & functional activities	Symptomatic ataxia causing inability to perform usual social & functional activities	Disabling ataxia causing inability to perform basic self-care functions
Cognitive & behavioral/attentional disturbance (including dementia & attention deficit disorder)	Disability causing no or minimal interference with usual social & functional activities OR Specialized resources not indicated	Disability causing greater than minimal interference with usual social & functional activities OR Specialized resources on part-time basis indicated	Disability causing inability to perform usual social & functional activities OR Specialized resources on a full-time basis indicated	Disability causing inability to perform basic self-care functions OR Institutionalization indicated
CNS ischemia (acute)	NA	NA	Transient ischemic attack	Cerebral vascular accident (CVA, stroke) with neurological deficit

Basic Self-care Functions – Adult: Activities such as bathing, dressing, toileting, transfer/movement, continence, and feeding.
Basic Self-care Functions – Young Children: Activities that are age and culturally appropriate (e.g., feeding self with culturally appropriate eating implement).
Usual Social & Functional Activities – Adult: Adaptive tasks and desirable activities, such as going to work, shopping, cooking, use of transportation, pursuing a hobby, etc.
Usual Social & Functional Activities – Young Children: Activities that are age and culturally appropriate (e.g., social interactions, play activities, learning tasks, etc).

PARAMETER	GRADE 1<br>MILD	GRADE 2<br>MODERATE	GRADE 3<br>SEVERE	GRADE 4<br>POTENTIALLY LIFE-THREATENING
Developmental delay– **Pediatric ≤ 16 years**	Mild developmental delay, either motor or cognitive, as determined by comparison with a developmental screening tool appropriate for the setting	Moderate developmental delay, either motor or cognitive, as determined by comparison with an appropriate developmental screening tool	Severe developmental delay, either motor or cognitive, as determined by comparison with a developmental screening tool appropriate for the setting	Developmental regression, either motor or cognitive, as determined by comparison with a developmental screening tool appropriate for the setting
Headache	Symptoms causing no or minimal interference with usual social & functional activities	Symptoms causing greater than minimal interference with usual social & functional activities	Symptoms causing inability to perform usual social & functional activities	Symptoms causing inability to perform basic self-care functions OR Hospitalization indicated (other than emergency room visit) OR Headache with significant impairment of alertness or other neurologic function
Insomnia	NA	Difficulty sleeping causing greater than minimal interference with usual social & functional activities	Difficulty sleeping causing inability to perform usual social & functional activities	Disabling insomnia causing inability to perform basic self-care functions
Neuromuscular weakness (including myopathy & neuropathy)	Asymptomatic with decreased strength on exam OR Minimal muscle weakness causing no or minimal interference with usual social & functional activities	Muscle weakness causing greater than minimal interference with usual social & functional activities	Muscle weakness causing inability to perform usual social & functional activities	Disabling muscle weakness causing inability to perform basic self-care functions OR Respiratory muscle weakness impairing ventilation
Neurosensory alteration (including paresthesia and painful neuropathy)	Asymptomatic with sensory alteration on exam or minimal paresthesia causing no or minimal interference with usual social & functional activities	Sensory alteration or paresthesia causing greater than minimal interference with usual social & functional activities	Sensory alteration or paresthesia causing inability to perform usual social & functional activities	Disabling sensory alteration or paresthesia causing inability to perform basic self-care functions

Basic Self-care Functions – Adult: Activities such as bathing, dressing, toileting, transfer/movement, continence, and feeding.
Basic Self-care Functions – Young Children: Activities that are age and culturally appropriate (e.g., feeding self with culturally appropriate eating implement).
Usual Social & Functional Activities – Adult: Adaptive tasks and desirable activities, such as going to work, shopping, cooking, use of transportation, pursuing a hobby, etc.
Usual Social & Functional Activities – Young Children: Activities that are age and culturally appropriate (e.g., social interactions, play activities, learning tasks, etc).

PARAMETER	GRADE 1 MILD	GRADE 2 MODERATE	GRADE 3 SEVERE	GRADE 4 POTENTIALLY LIFE-THREATENING
Seizure: (new onset) – **Adult ≥ 18 years** See also Seizure: (known preexisting seizure disorder)	NA	1 seizure	2 – 4 seizures	Seizures of any kind which are prolonged, repetitive (e.g., status epilepticus), or difficult to control (refractory epilepsy)
Seizure: (known preexisting seizure disorder) – **Adult ≥ 18 years** For worsening of existing epilepsy the grades should be based on an increase from previous level of control to any of these levels.	NA	Increased frequency of preexisting seizures (non-repetitive) without change in seizure character OR Infrequent break-through seizures while on stable medication in a previously controlled seizure disorder	Change in seizure character from baseline either in duration or quality (e.g., severity or focality)	Seizures of any kind which are prolonged, repetitive (e.g., status epilepticus), or difficult to control (e.g., refractory epilepsy)
Seizure **– Pediatric < 18 years**	Seizure, generalized onset with or without secondary generalization, lasting < 5 minutes with < 24 hours post- ictal state	Seizure, generalized onset with or without secondary generalization, lasting 5–20 minutes with < 24 hours post- ictal state	Seizure, generalized onset with or without secondary generalization, lasting > 20 minutes	Seizure, generalized onset with or without secondary generalization, requiring intubation and sedation
Syncope (not associated with a procedure)	NA	Present	NA	NA
Vertigo	Vertigo causing no or minimal interference with usual social & functional activities	Vertigo causing greater than minimal interference with usual social & functional activities	Vertigo causing inability to perform usual social & functional activities	Disabling vertigo causing inability to perform basic self-care functions
**RESPIRATORY**				
Bronchospasm (acute)	FEV1 or peak flow reduced to 70 – 80%	FEV1 or peak flow 50-69%	FEV1 or peak flow 25-49%	Cyanosis OR FEV1 or peak flow < 25% OR Intubation

Basic Self-care Functions – Adult: Activities such as bathing, dressing, toileting, transfer/movement, continence, and feeding.
Basic Self-care Functions – Young Children: Activities that are age and culturally appropriate (e.g., feeding self with culturally appropriate eating implement).
Usual Social & Functional Activities – Adult: Adaptive tasks and desirable activities, such as going to work, shopping, cooking, use of transportation, pursuing a hobby, etc.
Usual Social & Functional Activities – Young Children: Activities that are age and culturally appropriate (e.g., social interactions, play activities, learning tasks, etc).

PARAMETER	GRADE 1<br>MILD	GRADE 2<br>MODERATE	GRADE 3<br>SEVERE	GRADE 4<br>POTENTIALLY<br>LIFE-THREATENING
**Dyspnea or respiratory distress**				
**Adult ≥ 14 years**	Dyspnea on exertion with no or minimal interference with usual social & functional activities	Dyspnea on exertion causing greater than minimal interference with usual social & functional activities	Dyspnea at rest causing inability to perform usual social & functional activities	Respiratory failure with ventilatory support indicated
**Pediatric < 14 years**	Wheezing OR minimal increase in respiratory rate for age	Nasal flaring OR Intercostal retractions OR Pulse oximetry 90–95%	Dyspnea at rest causing inability to perform usual social & functional activities OR Pulse oximetry < 90%	Respiratory failure with ventilatory support indicated
**MUSCULOSKELETAL**				
Arthralgia<br>See also Arthritis	Joint pain causing no or minimal interference with usual social & functional activities	Joint pain causing greater than minimal interference with usual social & functional activities	Joint pain causing inability to perform usual social & functional activities	Disabling joint pain causing inability to perform basic self-care functions
Arthritis<br>See also Arthralgia	Stiffness or joint swelling causing no or minimal interference with usual social & functional activities	Stiffness or joint swelling causing greater than minimal interference with usual social & functional activities	Stiffness or joint swelling causing inability to perform usual social & functional activities	Disabling joint stiffness or swelling causing inability to perform basic self-care functions
**Bone Mineral Loss**				
**Adult ≥ 21 years**	BMD t-score -2.5 to -1.0	BMD t-score < -2.5	Pathological fracture (including loss of vertebral height)	Pathologic fracture causing life-threatening consequences
**Pediatric < 21 years**	BMD z-score -2.5 to -1.0	BMD z-score < -2.5	Pathological fracture (including loss of vertebral height)	Pathologic fracture causing life-threatening consequences
Myalgia<br>(non-injection site)	Muscle pain causing no or minimal interference with usual social & functional activities	Muscle pain causing greater than minimal interference with usual social & functional activities	Muscle pain causing inability to perform usual social & functional activities	Disabling muscle pain causing inability to perform basic self-care functions
Osteonecrosis	NA	Asymptomatic with radiographic findings AND No operative intervention indicated	Symptomatic bone pain with radiographic findings OR Operative intervention indicated	Disabling bone pain with radiographic findings causing inability to perform basic self-care functions

Basic Self-care Functions – Adult: Activities such as bathing, dressing, toileting, transfer/movement, continence, and feeding.
Basic Self-care Functions – Young Children: Activities that are age and culturally appropriate (e.g., feeding self with culturally appropriate eating implement).
Usual Social & Functional Activities – Adult: Adaptive tasks and desirable activities, such as going to work, shopping, cooking, use of transportation, pursuing a hobby, etc.
Usual Social & Functional Activities – Young Children: Activities that are age and culturally appropriate (e.g., social interactions, play activities, learning tasks, etc).

PARAMETER	GRADE 1<br>MILD	GRADE 2<br>MODERATE	GRADE 3<br>SEVERE	GRADE 4<br>POTENTIALLY LIFE-THREATENING
**GENITOURINARY**				
Cervicitis (symptoms) (For use in studies evaluating topical study agents) For other cervicitis see Infection: Infection (any other than HIV infection)	Symptoms causing no or minimal interference with usual social & functional activities	Symptoms causing greater than minimal interference with usual social & functional activities	Symptoms causing inability to perform usual social & functional activities	Symptoms causing inability to perform basic self-care functions
Cervicitis (clinical exam) (For use in studies evaluating topical study agents) For other cervicitis see Infection: Infection (any other than HIV infection)	Minimal cervical abnormalities on examination (erythema, mucopurulent discharge, or friability) OR Epithelial disruption < 25% of total surface	Moderate cervical abnormalities on examination (erythema, mucopurulent discharge, or friability) OR Epithelial disruption of 25 – 49% total surface	Severe cervical abnormalities on examination (erythema, mucopurulent discharge, or friability) OR Epithelial disruption 50 – 75% total surface	Epithelial disruption > 75% total surface
Inter-menstrual bleeding (IMB)	Spotting observed by participant OR Minimal blood observed during clinical or colposcopic examination	Inter-menstrual bleeding not greater in duration or amount than usual menstrual cycle	Inter-menstrual bleeding greater in duration or amount than usual menstrual cycle	Hemorrhage with life-threatening hypotension OR Operative intervention indicated
Urinary tract obstruction (e.g., stone)	NA	Signs or symptoms of urinary tract obstruction without hydronephrosis or renal dysfunction	Signs or symptoms of urinary tract obstruction with hydronephrosis or renal dysfunction	Obstruction causing life-threatening consequences
Vulvovaginitis (symptoms) (Use in studies evaluating topical study agents) For other vulvovaginitis see Infection: Infection (any other than HIV infection)	Symptoms causing no or minimal interference with usual social & functional activities	Symptoms causing greater than minimal interference with usual social & functional activities	Symptoms causing inability to perform usual social & functional activities	Symptoms causing inability to perform basic self-care functions

Basic Self-care Functions – Adult: Activities such as bathing, dressing, toileting, transfer/movement, continence, and feeding.
Basic Self-care Functions – Young Children: Activities that are age and culturally appropriate (e.g., feeding self with culturally appropriate eating implement).
Usual Social & Functional Activities – Adult: Adaptive tasks and desirable activities, such as going to work, shopping, cooking, use of transportation, pursuing a hobby, etc.
Usual Social & Functional Activities – Young Children: Activities that are age and culturally appropriate (e.g., social interactions, play activities, learning tasks, etc).

PARAMETER	GRADE 1 MILD	GRADE 2 MODERATE	GRADE 3 SEVERE	GRADE 4 POTENTIALLY LIFE-THREATENING
Vulvovaginitis (clinical exam) (Use in studies evaluating topical study agents) For other vulvovaginitis see Infection: Infection (any other than HIV infection)	Minimal vaginal abnormalities on examination OR Epithelial disruption < 25% of total surface	Moderate vaginal abnormalities on examination OR Epithelial disruption of 25–49% total surface	Severe vaginal abnormalities on examination OR Epithelial disruption 50–75% total surface	Vaginal perforation OR Epithelial disruption > 75% total surface
**OCULAR/VISUAL**				
Uveitis	Asymptomatic but detectable on exam	Symptomatic anterior uveitis OR Medical intervention indicated	Posterior or pan-uveitis OR Operative intervention indicated	Disabling visual loss in affected eye(s)
Visual changes (from baseline)	Visual changes causing no or minimal interference with usual social & functional activities	Visual changes causing greater than minimal interference with usual social & functional activities	Visual changes causing inability to perform usual social & functional activities	Disabling visual loss in affected eye(s)
**ENDOCRINE/METABOLIC**				
Abnormal fat accumulation (e.g., back of neck, breasts, abdomen)	Detectable by study participant (or by caregiver for young children and disabled adults)	Detectable on physical exam by health care provider	Disfiguring OR Obvious changes on casual visual inspection	NA
Diabetes mellitus	NA	New onset without need to initiate medication OR Modification of current medications to regain glucose control	New onset with initiation of medication indicated OR Diabetes uncontrolled despite treatment modification	Life-threatening consequences (e.g., ketoacidosis, hyperosmolar non-ketotic coma)
Gynecomastia	Detectable by study participant or caregiver (for young children and disabled adults)	Detectable on physical exam by health care provider	Disfiguring OR Obvious on casual visual inspection	NA

Basic Self-care Functions – Adult: Activities such as bathing, dressing, toileting, transfer/movement, continence, and feeding.
Basic Self-care Functions – Young Children: Activities that are age and culturally appropriate (e.g., feeding self with culturally appropriate eating implement).
Usual Social & Functional Activities – Adult: Adaptive tasks and desirable activities, such as going to work, shopping, cooking, use of transportation, pursuing a hobby, etc.
Usual Social & Functional Activities – Young Children: Activities that are age and culturally appropriate (e.g., social interactions, play activities, learning tasks, etc).

PARAMETER	GRADE 1 MILD	GRADE 2 MODERATE	GRADE 3 SEVERE	GRADE 4 POTENTIALLY LIFE-THREATENING
Hyperthyroidism	Asymptomatic	Symptomatic causing greater than minimal interference with usual social & functional activities OR Thyroid suppression therapy indicated	Symptoms causing inability to perform usual social & functional activities OR Uncontrolled despite treatment modification	Life-threatening consequences (e.g., thyroid storm)
Hyperthyroidism	Asymptomatic	Symptomatic causing greater than minimal interference with usual social & functional activities OR Thyroid suppression therapy indicated	Symptoms causing inability to perform usual social & functional activities OR Uncontrolled despite treatment modification	Life-threatening consequences (e.g., thyroid storm)
Hypothyroidism	Asymptomatic	Symptomatic causing greater than minimal interference with usual social & functional activities OR Thyroid replacement therapy indicated	Symptoms causing inability to perform usual social & functional activities OR Uncontrolled despite treatment modification	Life-threatening consequences (e.g., myxedema coma)
Lipoatrophy (e.g., fat loss from the face, extremities, buttocks)	Detectable by study participant (or by caregiver for young children and disabled adults)	Detectable on physical exam by health care provider	Disfiguring OR Obvious on casual visual inspection	NA

Basic Self-care Functions – Adult: Activities such as bathing, dressing, toileting, transfer/movement, continence, and feeding.
Basic Self-care Functions – Young Children: Activities that are age and culturally appropriate (e.g., feeding self with culturally appropriate eating implement).
Usual Social & Functional Activities – Adult: Adaptive tasks and desirable activities, such as going to work, shopping, cooking, use of transportation, pursuing a hobby, etc.
Usual Social & Functional Activities – Young Children: Activities that are age and culturally appropriate (e.g., social interactions, play activities, learning tasks, etc).

LABORATORY				
**PARAMETER**	**GRADE 1 MILD**	**GRADE 2 MODERATE**	**GRADE 3 SEVERE**	**GRADE 4 POTENTIALLY LIFE-THREATENING**
**HEMATOLOGY**	**Standard International Units are listed in italics**			
Absolute CD4+ count – **Adult and Pediatric > 13 years** (HIV NEGATIVE ONLY)	300 – 400/mm^3 *300 – 400/µL*	200 – 299/mm^3 *200 – 299/µL*	100 – 199/mm^3 *100 – 199/µL*	< 100/mm^3 *< 100/µL*
Absolute lymphocyte count – **Adult and Pediatric > 13 years** (HIV NEGATIVE ONLY)	600 – 650/mm^3 0.600 x 10^9 – 0.650 x 10^9*/L*	500 – 599/mm^3 0.500 x 10^9 – 0.599 x 10^9*/L*	350 – 499/mm^3 0.350 x 10^9 – 0.499 x 10^9*/L*	< 350/mm^3 < 0.350 x 10^9/L
**Comment:** Values in children ≤ 13 years are not given for the two parameters above because the absolute counts are variable.				
Absolute neutrophil count (ANC)				
**Adult and Pediatric, > 7 days**	1,000 – 1,300/mm^3 1.000 x 10^9 – 1.300 x 10^9/L	750 – 999/mm^3 0.750 x 10^9 – 0.999 x 10^9/L	500 – 749/mm^3 0.500 x 10^9 – 0.749 x 10^9/L	< 500/mm^3 < 0.500 x 10^9/L
**Infant*†, 2 – ≤ 7 days**	1,250 – 1,500/mm^3 1.250 x 10^9 – 1.500 x 10^9/L	1,000 – 1,249/mm^3 1.000 x 10^9 – 1.249 x 10^9/L	750 – 999/mm^3 0.750 x 10^9 – 0.999 x 10^9/L	< 750/mm^3 < 0.750 x 10^9/L
**Infant*†, ≤1 day**	4,000 – 5,000/mm^3 4.000 x 10^9 – 5.000 x 10^9/L	3,000 – 3,999/mm^3 3.000 x 10^9 – 3.999 x10^9/L	1,500 – 2,999/mm^3 1.500 x 10^9 – 2.999 x 10^9/L	< 1,500/mm^3 < 1.500 x 10^9/L
**Comment:** Parameter changed from "Infant, < 1 day" to "Infant, ≤1 day"				
Fibrinogen, decreased	100 – 200 mg/dL *1.00 – 2.00 g/L* OR 0.75 – 0.99 x LLN	75 – 99 mg/dL 0.75 – 0.99 g/L OR 0.50 – 0.74 x LLN	50 – 74 mg/dL 0.50 – 0.74 g/L OR 0.25 – 0.49 x LLN	< 50 mg/dL *< 0.50 g/L* OR < 0.25 x LLN OR Associated with gross bleeding

* Values are for term infants. Preterm infants should be assessed using local normal ranges.
† Use age and sex appropriate values (e.g., bilirubin).

LABORATORY					
**PARAMETER**		**GRADE 1 MILD**	**GRADE 2 MODERATE**	**GRADE 3 SEVERE**	**GRADE 4 POTENTIALLY LIFE-THREATENING**
Hemoglobin (Hgb)					
**Comment**: The Hgb values in mmol/L have changed because the conversion factor used to convert g/dL to mmol/L has been changed from 0.155 to 0.6206 (the most commonly used conversion factor). For grading Hgb results obtained by an analytic method with a conversion factor other than 0.6206, the result must be converted to g/dL using the appropriate conversion factor for that lab.					
	**Adult and Pediatric ≥ 57 days** (HIV POSITIVE ONLY)	8.5 – 10.0 g/dL<br>*5.24 – 6.23 mmol/L*	7.5 – 8.4 g/dL<br>*4.62–5.23 mmol/L*	6.50 – 7.4 g/dL<br>*4.03–4.61 mmol/L*	< 6.5 g/dL<br>*< 4.03 mmol/L*
	**Adult and Pediatric ≥ 57 days** (HIV NEGATIVE ONLY)	10.0 – 10.9 g/dL<br>6.18 – 6.79 mmol/L<br>OR<br>Any decrease<br>2.5 – 3.4 g/dL<br>1.58 – 2.13 mmol/L	9.0 – 9.9 g/dL<br>5.55 - 6.17 mmol/L<br>OR<br>Any decrease<br>3.5 – 4.4 g/dL<br>2.14 – 2.78 mmol/L	7.0 – 8.9 g/dL<br>4.34 - 5.54 mmol/L<br>OR<br>Any decrease<br>≥ 4.5 g/dL<br>> 2.79 mmol/L	< 7.0 g/dL<br>*< 4.34 mmol/L*
**Comment**: The decrease is a decrease from baseline					
	**Infant∗†, 36 – 56 days** (HIV POSITIVE OR NEGATIVE)	8.5 – 9.4 g/dL<br>*5.24 – 5.86 mmol/L*	7.0 – 8.4 g/dL<br>*4.31 – 5.23 mmol/L*	6.0 – 6.9 g/dL<br>*3.72 – 4.30 mmol/L*	< 6.00 g/dL<br>*< 3.72 mmol/L*
	**Infant∗†, 22 – 35 days** (HIV POSITIVE OR NEGATIVE)	9.5 – 10.5 g/dL<br>*5.87 - 6.54 mmol/L*	8.0 – 9.4 g/dL<br>*4.93 – 5.86 mmol/L*	7.0 – 7.9 g/dL<br>*4.34 – 4.92 mmol/L*	< 7.00 g/dL<br>*< 4.34 mmol/L*
	**Infant∗†, ≤ 21 days** (HIV POSITIVE OR NEGATIVE)	12.0 – 13.0 g/dL<br>*7.42 – 8.09 mmol/L*	10.0 – 11.9 g/dL<br>*6.18 – 7.41 mmol/L*	9.0 – 9.9 g/dL<br>*5.59- 6.17 mmol/L*	< 9.0 g/dL<br>*< 5.59 mmol/L*
**Correction**: Parameter changed from "Infant < 21 days" to "Infant ≤ 21 days"					
International Normalized Ratio of prothrombin time (INR)		1.1 – 1.5 x ULN	1.6 – 2.0 x ULN	2.1 – 3.0 x ULN	> 3.0 x ULN
Methemoglobin		5.0 – 10.0%	10.1 – 15.0%	15.1 – 20.0%	> 20.0%
Prothrombin Time (PT)		1.1 – 1.25 x ULN	1.26 – 1.50 x ULN	1.51 – 3.00 x ULN	> 3.00 x ULN
Partial Thromboplastin Time (PTT)		1.1 – 1.66 x ULN	1.67 – 2.33 x ULN	2.34 – 3.00 x ULN	> 3.00 x ULN
Platelets, decreased		100,000– 124,999/mm^3<br>100.000 x 10^9 – 124.999 x 10^9/L	50,000 –99,999/mm^3<br>50.000 x 10^9 – 99.999 x 10^9/L	25,000 – 49,999/mm^3<br>25.000 x 10^9 – 49.999 x 10^9/L	< 25,000/mm^3<br>< 25.000 x 10^9/L
WBC, decreased		2,000 – 2,500/mm^3<br>2.000 x 10^9 – 2.500 x 10^9/L	1,500 – 1,999/mm^3<br>1.500 x 10^9 – 1.999 x 10^9/L	1,000 – 1,499/mm^3<br>1.000 x 10^9 – 1.499 x 10^9/L	< 1,000/mm^3<br>< 1.000 x 10^9/L

∗ Values are for term infants. Preterm infants should be assessed using local normal ranges.
† Use age and sex appropriate values (e.g., bilirubin).

LABORATORY				
**PARAMETER**	**GRADE 1 MILD**	**GRADE 2 MODERATE**	**GRADE 3 SEVERE**	**GRADE 4 POTENTIALLY LIFE-THREATENING**
**CHEMISTRIES Standard International Units are listed in italics**				
Acidosis	NA	pH < normal, but ≥ 7.3	pH < 7.3 without life- threatening consequences	pH < 7.3 with life-threatening consequences
Albumin, serum, low	3.0 g/dL – < LLN<br>*30 g/L – < LLN*	2.0 – 2.9 g/dL<br>*20 – 29 g/L*	< 2.0 g/dL<br>*< 20 g/L*	NA
Alkaline Phosphatase	1.25 – 2.5 x ULN†	2.6 – 5.0 x ULN†	5.1 – 10.0 x ULN†	> 10.0 x ULN†
Alkalosis	NA	pH > normal, but ≤ 7.5	pH > 7.5 without life- threatening consequences	pH > 7.5 with life-threatening consequences
ALT (SGPT)	1.25 – 2.5 x ULN	2.6 – 5.0 x ULN	5.1 – 10.0 x ULN	> 10.0 x ULN
AST (SGOT)	1.25 – 2.5 x ULN	2.6 – 5.0 x ULN	5.1 – 10.0 x ULN	> 10.0 x ULN
Bicarbonate, serum, low	16.0 mEq/L – < LLN<br>*16.0 mmol/L – < LLN*	11.0 – 15.9 mEq/L<br>*11.0 – 15.9 mmol/L*	8.0 – 10.9 mEq/L<br>*8.0 – 10.9 mmol/L*	< 8.0 mEq/L<br>*< 8.0 mmol/L*
**Comment:** Some laboratories will report this value as Bicarbonate (HCO_3) and others as Total Carbon Dioxide (CO_2). These are the same tests; values should be graded according to the ranges for Bicarbonate as listed above.				
Bilirubin (Total)				
**Adult and Pediatric >14 days**	1.1 – 1.5 x ULN	1.6 – 2.5 x ULN	2.6 – 5.0 x ULN	> 5.0 x ULN
**Infant*†, ≤ 14 days (non-hemolytic)**	NA	20.0 – 25.0 mg/dL<br>*342 – 428 µmol/L*	25.1 – 30.0 mg/dL<br>*429 – 513 µmol/L*	> 30.0 mg/dL<br>*> 513.0 µmol/L*
**Infant*†, ≤ 14 days (hemolytic)**	NA	NA	20.0 – 25.0 mg/dL<br>*342 – 428 µmol/L*	> 25.0 mg/dL<br>*> 428 µmol/L*
Calcium, serum, high				
**Adult and Pediatric ≥ 7 days**	10.6 – 11.5 mg/dL<br>*2.65 – 2.88 mmol/L*	11.6 – 12.5 mg/dL<br>*2.89 – 3.13 mmol/L*	12.6 – 13.5 mg/dL<br>*3.14 – 3.38 mmol/L*	> 13.5 mg/dL<br>*> 3.38 mmol/L*
**Infant*†, < 7 days**	11.5 – 12.4 mg/dL<br>*2.88 – 3.10 mmol/L*	12.5 – 12.9 mg/dL<br>*3.11 – 3.23 mmol/L*	13.0 – 13.5 mg/dL<br>*3.245 – 3.38 mmol/L*	> 13.5 mg/dL<br>*> 3.38 mmol/L*
Calcium, serum, low				
**Adult and Pediatric ≥ 7 days**	7.8 – 8.4 mg/dL<br>1.95 – 2.10 mmol/L	7.0 – 7.7 mg/dL<br>1.75 – 1.94 mmol/L	6.1 – 6.9 mg/dL<br>1.53 – 1.74 mmol/L	< 6.1 mg/dL<br>< 1.53 mmol/L
**Infant*†, < 7 days**	6.5 – 7.5 mg/dL<br>1.63 – 1.88 mmol/L	6.0 – 6.4 mg/dL<br>1.50 – 1.62 mmol/L	5.50 – 5.90 mg/dL<br>1.38 – 1.51 mmol/L	< 5.50 mg/dL<br>< 1.38 mmol/L
**Comment:** Do not adjust Calcium, serum, low or Calcium, serum, high for albumin				

∗ Values are for term infants. Preterm infants should be assessed using local normal ranges.
† Use age and sex appropriate values (e.g., bilirubin).

LABORATORY				
**PARAMETER**	**GRADE 1 MILD**	**GRADE 2 MODERATE**	**GRADE 3 SEVERE**	**GRADE 4 POTENTIALLY LIFE-THREATENING**
Cardiac troponin I (cTnI)	NA	NA	NA	Levels consistent with myocardial infarction or unstable angina as defined by the manufacturer
Cardiac troponin T (cTnT)	NA	NA	NA	≥ 0.20 ng/mL<br>OR<br>Levels consistent with myocardial infarction or unstable angina as defined by the manufacturer
Cholesterol (fasting)				
**Adult ≥ 18 years**	200 – 239 mg/dL<br>5.18 – 6.19 mmol/L	240 – 300 mg/dL<br>6.20 – 7.77 mmol/L	> 300 mg/dL<br>> 7.77 mmol/L	NA
**Pediatric < 18 years**	170 – 199 mg/dL<br>4.40 – 5.15 mmol/L	200 – 300 mg/dL<br>5.16 – 7.77 mmol/L	> 300 mg/dL<br>> 7.77 mmol/L	NA
Creatine Kinase	3.0 – 5.9 x ULN†	6.0 – 9.9 x ULN†	10.0 – 19.9 x ULN†	≥ 20.0 x ULN†
Creatinine	1.1 – 1.3 x ULN†	1.4 – 1.8 x ULN†	1.9 – 3.4 x ULN†	≥ 3.5 x ULN†

∗ Values are for term infants. Preterm infants should be assessed using local normal ranges.
† Use age and sex appropriate values (e.g., bilirubin).

LABORATORY				
**PARAMETER**	**GRADE 1 MILD**	**GRADE 2 MODERATE**	**GRADE 3 SEVERE**	**GRADE 4 POTENTIALLY LIFE-THREATENING**
Glucose, serum, high				
Nonfasting	116 – 160 mg/dL *6.44 – 8.88 mmol/L*	161 – 250 mg/dL *8.89 – 13.88 mmol/L*	251 – 500 mg/dL *13.89 – 27.75 mmol/L*	> 500 mg/dL *> 27.75 mmol/L*
Fasting	110 – 125 mg/dL *6.11 – 6.94 mmol/L*	126 – 250 mg/dL *6.95 – 13.88 mmol/L*	251 – 500 mg/dL *13.89 – 27.75 mmol/L*	> 500 mg/dL *> 27.75 mmol/L*
Glucose, serum, low				
**Adult and Pediatric ≥ 1 month**	55 – 64 mg/dL 3.05 – 3.55 mmol/L	40 – 54 mg/dL 2.22 – 3.06 mmol/L	30 – 39 mg/dL 1.67 – 2.23 mmol/L	< 30 mg/dL < 1.67 mmol/L
**Infant*†, < 1 month**	50 – 54 mg/dL 2.78 – 3.00 mmol/L	40 – 49 mg/dL 2.22 – 2.77 mmol/L	30 – 39 mg/dL 1.67 – 2.21 mmol/L	< 30 mg/dL < 1.67 mmol/L
Lactate	ULN - < 2.0 x ULN without acidosis	≥ 2.0 x ULN without acidosis	Increased lactate with pH < 7.3 w/o life- threatening consequences	Increased lactate with pH < 7.3 with life-threatening consequences
**Comment:** Added ULN to Grade 1 parameter				
LDL cholesterol (fasting)				
**Adult ≥ 18 years**	130 – 159 mg/dL *3.37 – 4.12 mmol/L*	160 – 190 mg/dL *4.13 – 4.90 mmol/L*	≥ 190 mg/dL ≥ 4.91 mmol/L	NA
**Pediatric > 2– < 18 years**	110 – 129 mg/dL *2.85 – 3.34 mmol/L*	130 – 189 mg/dL *3.35 – 4.90 mmol/L*	≥ 190 mg/dL *≥ 4.91 mmol/L*	NA
Lipase	1.1 – 1.5 x ULN	1.6 – 3.0 x ULN	3.1 – 5.0 x ULN	> 5.0 x ULN
Magnesium, serum, low	1.2 – 1.4 mEq/L *0.60 – 0.70 mmol/L*	0.9 – 1.1 mEq/L *0.45 – 0.59 mmol/L*	0.6 – 0.8 mEq/L *0.30 – 0.44 mmol/L*	< 0.60 mEq/L *< 0.30 mmol/L*
Pancreatic amylase	1.1 – 1.5 x ULN	1.6 – 2.0 x ULN	2.1 – 5.0 x ULN	> 5.0 x ULN
Phosphate, serum, low				
**Adult and Pediatric > 14 years**	2.5 mg/dL – < LLN *0.81 mmol/L – < LLN*	2.0 – 2.4 mg/dL 0.65 – 0.80 mmol/L	1.0 – 1.9 mg/dL 0.32 – 0.64 mmol/L	< 1.00 mg/dL < 0.32 mmol/L
**Pediatric 1 year – 14 years**	3.0 – 3.5 mg/dL *0.97 – 1.13 mmol/L*	2.5 – 2.9 mg/dL 0.81 – 0.96 mmol/L	1.5 – 2.4 mg/dL 0.48 – 0.80 mmol/L	< 1.50 mg/dL *< 0.48 mmol/L*
**Pediatric < 1 year**	3.5 – 4.5 mg/dL 1.13 – 1.45 mmol/L	2.5 – 3.4 mg/dL 0.81 – 1.12 mmol/L	1.5 – 2.4 mg/dL 0.48 – 0.80 mmol/L	< 1.50 mg/dL < 0.48 mmol/L
Potassium, serum, high	5.6 – 6.0 mEq/L 5.6 – 6.0 mmol/L	6.1 – 6.5 mEq/L 6.1 – 6.5 mmol/L	6.6 – 7.0 mEq/L 6.6 – 7.0 mmol/L	> 7.0 mEq/L > 7.0 mmol/L
Potassium, serum, low	3.0 – 3.4 mEq/L *3.0 – 3.4 mmol/L*	2.5 – 2.9 mEq/L 2.5 – 2.9 mmol/L	2.0 – 2.4 mEq/L 2.0 – 2.4 mmol/L	< 2.0 mEq/L *< 2.0 mmol/L*
Sodium, serum, high	146 – 150 mEq/L 146 – 150 mmol/L	151 – 154 mEq/L 151 – 154 mmol/L	155 – 159 mEq/L 155 – 159 mmol/L	≥ 160 mEq/L ≥ 160 mmol/L
Sodium, serum, low	130 – 135 mEq/L *130 – 135 mmol/L*	125 – 129 mEq/L 125 – 129 mmol/L	121 – 124 mEq/L 121 – 124 mmol/L	≤ 120 mEq/L ≤ 120 mmol/L

* Values are for term infants. Preterm infants should be assessed using local normal ranges.
† Use age and sex appropriate values (e.g., bilirubin).

LABORATORY				
**PARAMETER**	**GRADE 1 MILD**	**GRADE 2 MODERATE**	**GRADE 3 SEVERE**	**GRADE 4 POTENTIALLY LIFE-THREATENING**
Triglycerides (fasting)	NA	500 – 750 mg/dL<br>5.65 – 8.48 mmol/L	751 – 1,200 mg/dL<br>8.49 – 13.56 mmol/L	> 1,200 mg/dL<br>> 13.56 mmol/L
Uric acid	7.5–10.0 mg/dL<br>*0.45-0.59 mmol/L*	10.1 – 12.0 mg/dL<br>*0.60–0.71 mmol/L*	12.1 – 15.0 mg/dL<br>*0.72 – 0.89 mmol/L*	> 15.0 mg/dL<br>*> 0.89 mmol/L*
**URINALYSIS**	***Standard International Units are listed in italics***			
Hematuria (microscopic)	6 – 10 RBC/HPF	> 10 RBC/HPF	Gross, with or without clots OR with RBC casts	Transfusion indicated
Proteinuria, random collection	1 +	2 – 3 +	4 +	NA
Proteinuria, 24 hour collection				
**Adult and Pediatric ≥ 10 years**	200–999 mg/24 h<br>*0.200 – 0.999 g/d*	1,000–1,999 mg/24 h<br>*1.000 – 1.999 g/d*	2,000-3,500 mg/24 h<br>*2.000 – 3.500 g/d*	> 3,500 mg/24 h<br>*> 3.500 g/d*
**Pediatric > 3 mo-< 10 years**	201-499 mg/m^2/24 h<br>*0.201 – 0.499 g/d*	500-799 mg/m^2/24 h<br>*0.500 – 0.799 g/d*	800-1,000 mg/m^2/24 h<br>*0.800 – 1.000 g/d*	> 1,000 mg/ m^2/24 h<br>*> 1.000 g/d*

∗ Values are for term infants. Preterm infants should be assessed using local normal ranges.
† Use age and sex appropriate values (e.g., bilirubin).

Appendix D. Summary of Protocol Changes from Version 1.0 dated 12 December 2013 to Version 2.0

Section	Original Text in Version 1.0	Revised Text in Version 2.0	Reason for Change
**Cover page**<br>Date and Version Number	12 December 2013<br>Version 2.0	19 December 2013<br>Version 3.0	To reflect revised version of protocol
**Synopsis**<br>*Study Design, Figure 1.1*	Footnote to Figure read:<br>*Includes a sentinel cohort of 5 subjects aged 6–12 months of age (4 ALS-8176: 1 placebo)	Footnote to Figure reads:<br>*Each single ascending dose level includes sentinel cohorts (N= 3/age stratum; 2 ALS-8176: 1 placebo) whose data will be reviewed by the IDMC prior to:<br>• initiating additional sentinel cohort(s) for other age strata<br>• completion of current age stratum (N=5/age stratum)	Based on internal review and scientific advice from health authorities, sentinel groups and age de-escalation in the SAD part have been added as an additional safety precaution.
Part 1 (single ascending dose – SAD)<br>*(¶2 onward)*	Two additional cohorts (Cohorts 5 and 6) may be evaluated at the discretion of the Sponsor, upon approval by the Independent Data Monitoring Committee (IDMC), based on an evaluation of the emerging PK profile and the safety profile. Within each dosing cohort, subjects will be randomized to receive either ALS-008176 or placebo (n = approximately 24 per cohort; randomized in a ratio of 3 ALS-008176:1 placebo). Randomization will be stratified by age range (≥ 1.0 to < 3.0 months, ≥ 3.0 to < 6.0 months, and ≥ 6.0 to ≤ 12.0 months), with approximately 8 subjects being enrolled in each age stratum.<br>Initially, a sentinel group of 5 subjects aged ≥ 6.0 to ≤ 12.0 months will be enrolled in the first SAD dosing cohort (randomized in a ratio of 4 ALS-008176:1 placebo). The PK data from the sentinel group will be used to confirm or adjust the predictions of the pediatric PK model before any additional subjects are enrolled (see also Section 1.3, Rationale for Dose Selection). After IDMC review of these data, the remaining 3 subjects in the first ≥ 6.0 to ≤ 12.0 months SAD cohort will be randomized (in a ratio of	Two additional cohorts (Cohorts 5 and 6) may be evaluated at the discretion of the Sponsor, upon approval by the Independent Data Monitoring Committee (IDMC), based on an evaluation of the emerging PK profile and the safety profile.<br>Within each dosing cohort, subjects will be randomized to receive either ALS-008176 or placebo (n = approximately 24 per cohort; randomized in a ratio of 3 ALS-008176:1 placebo). Randomization will be stratified by age range (≥ 1.0 to < 2.0 months, ≥ 2.0 to < 6.0 months, and ≥ 6.0 to ≤ 12.0 months), with approximately 8 subjects being enrolled in each age stratum.<br>An age de-escalation approach will be utilized within each SAD dose cohort, i.e. subjects aged ≥ 6.0 to ≤ 12.0 months will be enrolled initially, followed by subjects ≥ 2.0 to < 6.0 months, and then subjects ≥ 1.0 to < 2.0 months, as follows:<br>• A sentinel group of 3 subjects in each age stratum will be enrolled first (randomized in a ratio of 2 ALS-008176:1 placebo).<br>• The IDMC will review the safety data through Day 7 and available PK data for the sentinel	Based on internal review and scientific advice from health authorities, sentinel groups and age de-escalation in the SAD part have been added as an additional safety precaution.

Section	Original Text in Version 1.0	Revised Text in Version 2.0	Reason for Change
	2 ALS-008176:1 placebo) and all subjects in the lower age groups of the first dosing cohort will be enrolled. The dose for the remaining subjects in this and future dosing cohorts may be revised based on data derived from the sentinel subjects.<br>Subjects will be evaluated over a 7-day period from the time of randomization. Additionally, all subjects will receive standard supportive care per local institution. Any subjects discharged from the hospital prior to Day 7 will be required to return as an outpatient for assessment on Day 7, when they will complete the study.<br>Dose escalation within Part 1 will not occur before the IDMC has reviewed and deemed acceptable:<br>• all safety data through study completion for all subjects in the current cohort or age stratum<br>• PK data from the first 24 hours after dosing for at least 75% of the current cohorts' subjects (or a completed stratum within this cohort)	subjects. Following this:<br>o The remaining 5 subjects in the age stratum will be enrolled (randomized in a ratio of 4 ALS-008176:1 placebo), and<br>o Enrollment in the next age stratum's sentinel cohort(s) will be initiated.<br>At no time will dose escalation occur for a younger age stratum before dosing in an older age stratum has been initiated for that dose.<br>Subjects will be evaluated over a 7-day period from the time of randomization. Additionally, all subjects will receive standard supportive care per local institution. Any subjects discharged from the hospital prior to Day 7 will be required to return as an outpatient for assessment on Day 7, when they will complete the study.<br>Dose escalation within Part 1 will not occur before the IDMC has reviewed and deemed acceptable:<br>• all safety data through study completion for all subjects in the current cohort or age stratum<br>• available PK data from the first 24 hours after dosing for at least 75% of the current cohorts' subjects (or a completed stratum within this cohort)	
**Part 2 (multiple ascending dose – MAD):**<br>*(¶6 and ¶8)*	Within each dosing cohort, subjects will be randomized to receive either ALS-008176 or placebo (n = approximately 24 per cohort; randomized in a ratio of 3 ALS-008176: 1 placebo). Randomization will be stratified by age at time of hospital admission (≥ 1.0 to < 3.0 months, ≥ 3.0 to < 6.0 months, and ≥ 6.0 to ≤ 12.0 months), with approximately 8 subjects being enrolled in each stratum. Additionally, all subjects will receive standard supportive care as per local institution. Subjects will be evaluated over an 11-day period from the time of randomization. If they are discharged from the	Within each dosing cohort, subjects will be randomized to receive either ALS-008176 or placebo (n = approximately 24 per cohort; randomized in a ratio of 3 ALS-008176: 1 placebo). Randomization will be stratified by age at time of hospital admission (≥ 1.0 to < 2.0 months, ≥ 2.0 to < 6.0 months, and ≥ 6.0 to ≤ 12.0 months), with approximately 8 subjects being enrolled in each stratum. Additionally, all subjects will receive standard supportive care as per local institution. Subjects will be evaluated over an 11-day period from the time of randomization. If they are	Based on internal review and scientific advice from health authorities, the age strata were refined.<br><br>Clarification

Section	Original Text in Version 1.0	Revised Text in Version 2.0	Reason for Change
	hospital prior to Day 11, they will be required to return for assessment as an outpatient on Day 5, if applicable, and on Day 11, when they will complete the study.<br>Following completion of each age group within a cohort, the pharmacokinetics of ALS-008112 and ALS-008144 will be evaluated and reviewed. An established pediatric PK model will be updated with the additional data, and the plasma exposures of the subsequent dose will be simulated prior to dosing, as a safety check to ensure that the pharmacokinetics of ALS-008112 are not predicted to differ significantly from the intended exposures. Alterations in the planned dose escalation scheme will be made as necessary. In addition, under no circumstances will a planned pediatric dose exceed a projected average ALS-008112 AUC_{0-24} of 20,000 ng•h/mL. Furthermore, the decision to dose escalate between cohorts will be based on a review of safety and PK data by the Sponsor and IDMC.	discharged from the hospital prior to Day 11, they will be required to return for assessment as an outpatient on Day 5, if applicable, and on Day 11, when they will complete the study.<br>Throughout the conduct of the study, the pharmacokinetics of ALS-008112 and ALS-008144 will be evaluated and reviewed. An established pediatric PK model will be updated with the additional data, and the plasma exposures of the subsequent dose will be simulated prior to dose escalation, as a safety check to ensure that the pharmacokinetics of ALS-008112 are not predicted to differ significantly from the intended exposures. Alterations in the planned dose escalation scheme will be made as necessary. In addition, under no circumstances will a planned pediatric dose exceed a projected average ALS-008112 AUC_{0-24} of 20,000 ng•h/mL. Furthermore, the decision to dose escalate between cohorts will be based on a review of safety and PK data by the Sponsor and IDMC.	
**2.1 Summary**<br>*Figure 2.1*	Footnote to Figure read:<br>Includes a sentinel cohort of 5 subjects aged 6–12 months of age (4 ALS-8176: 1 placebo)	Footnote to Figure reads:<br>Each single ascending dose level includes sentinel cohorts (N= 3/age stratum; 2 ALS-8176: 1 placebo) whose data will be reviewed by the IDMC prior to:<br>• initiating additional sentinel cohort(s) for other age strata<br>• completion of current age stratum (N=5/age stratum)	Based on internal review and scientific advice from health authorities, sentinel groups and age de-escalation in the SAD part have been added as an additional safety precaution.
**2.2 *Part 1: Single Ascending Dose (SAD)***	Subjects will receive a single dose from 1 of 4 ascending dose levels of ALS-008176.<br>Within each dosing cohort, subjects will be randomized to receive either ALS-008176 or placebo (n = approximately 24 per cohort; randomized in a	Subjects will receive a single dose from 1 of 4 ascending dose levels of ALS-008176.<br>Within each dosing cohort, subjects will be randomized to receive either ALS-008176 or placebo (n = approximately 24 per cohort;	Based on internal review and scientific advice from health authorities, the age strata were refined.

Section	Original Text in Version 1.0	Revised Text in Version 2.0	Reason for Change
	ratio of 3 ALS-008176:1 placebo). Randomization will be stratified by age at time of hospital admission (≥ 1.0 to < 3.0 months, ≥ 3.0 to < 6.0 months, and ≥ 6.0 to ≤ 12.0 months), with approximately 8 subjects being enrolled in each stratum. Additionally, all subjects will receive standard supportive care.<br><br>Initially, a sentinel group of 5 subjects aged ≥ 6.0 to ≤ 12.0 months will be enrolled in the first SAD dosing cohort (randomized in a ratio of 4 ALS-008176:1 placebo). The PK data from the sentinel group will be used to confirm or adjust the predictions of the pediatric PK model before any additional subjects are enrolled (see also Section 1.3, Rationale for Dose Selection). After IDMC review of these data, the remaining 3 subjects in the first ≥ 6.0 to ≤ 12.0 months SAD cohort will be randomized (in a ratio of 2 ALS-008176:1 placebo) and all subjects in the lower age groups of the first dosing cohort will be enrolled. The dose for the remaining subjects in this and future dosing cohorts may be revised based on data derived from the sentinel subjects. Subjects will be evaluated over a 7-day period from the time of randomization. If they are discharged from the hospital prior to Day 7, they will be required to return as an outpatient for assessment on Day 7, when they will complete the study.<br><br>Dose escalation within Part 1 will not occur before the IDMC has reviewed and deemed acceptable:<br>• all safety data through study completion for all subjects in the current cohort or age stratum<br>• PK data from the first 24 hours after dosing for at least 75% of the current cohorts' subjects (or a completed stratum within this cohort)<br><br>A fifth and sixth cohort may be evaluated at the	randomized in a ratio of 3 ALS-008176:1 placebo). Randomization will be stratified by age at time of hospital admission (≥ 1.0 to < 2.0 months, ≥ 2.0 to < 6.0 months, and ≥ 6.0 to ≤ 12.0 months), with approximately 8 subjects being enrolled in each stratum. Additionally, all subjects will receive standard supportive care.<br><br>An age de-escalation approach will be utilized within each SAD dose cohort, i.e. subjects aged ≥ 6.0 to ≤ 12.0 months will be enrolled initially, followed by subjects ≥ 2.0 to < 6.0 months, and then subjects ≥ 1.0 to < 2.0 months, as follows:<br>• A sentinel group of 3 subjects in each age stratum will be enrolled first (randomized in a ratio of 2 ALS-008176:1 placebo).<br>• The IDMC will review the safety data through Day 7 and available PK data for the sentinel subjects. Following this:<br>o The remaining 5 subjects in the age stratum will be enrolled (randomized in a ratio of 4 ALS-008176:1 placebo), and<br>o Enrollment in the next age stratum's sentinel cohort(s) will be initiated.<br><br>At no time will dose escalation occur for a younger age stratum before dosing in an older age stratum has been initiated for that dose.<br><br>Subjects will be evaluated over a 7-day period from the time of randomization. If they are discharged from the hospital prior to Day 7, they will be required to return as an outpatient for assessment on Day 7, when they will complete the study.<br><br>Dose escalation within Part 1 will not occur before the IDMC has reviewed and deemed acceptable:	Based on internal review and scientific advice from health authorities, sentinel groups and age de-escalation in the SAD part have been added as an additional safety precaution.<br><br>Clarification

Section	Original Text in Version 1.0	Revised Text in Version 2.0	Reason for Change
	discretion of the Sponsor, upon approval by the IDMC, based on an evaluation of the emerging PK profile and safety profile.<br><br>The planned dose escalation schema may be modified by the Sponsor, upon approval by the IDMC, based on emerging PK and safety data. In all circumstances, the dosing schema in Part 1 will have no more than a three-fold increase in dose between dose levels (as outlined in Section 5.1.1, Cohort Progression Guidelines). In addition, under no circumstances will a potential dose be expected to exceed a projected average ALS-008112 AUC_{0-24} of 20,000 ng•h/mL. Following completion of each age group within a cohort, the pharmacokinetics of ALS-008112 and ALS-008144 will be evaluated and reviewed. An established pediatric PK model will be updated with the additional data, and the plasma exposures of the subsequent dose will be simulated prior to dosing, as a safety check to ensure that the pharmacokinetics of ALS-008112 are not predicted to differ significantly from the intended exposures.<br><br>See also Section 5.1.1, Cohort Progression Guidelines.	• all safety data through study completion for all subjects in the current cohort or age stratum<br>• available PK data from the first 24 hours after dosing for at least 75% of the current cohorts' subjects (or a completed stratum within this cohort)<br><br>A fifth and sixth cohort may be evaluated at the discretion of the Sponsor, upon approval by the IDMC, based on an evaluation of the emerging PK profile and safety profile.<br><br>The planned dose escalation schema may be modified by the Sponsor, upon approval by the IDMC, based on emerging PK and safety data. In all circumstances, the dosing schema in Part 1 will have no more than a three-fold increase in dose between dose levels (as outlined in Section 5.1.1, Cohort Progression Guidelines). In addition, under no circumstances will a potential dose be expected to exceed a projected average ALS-008112 AUC_{0-24} of 20,000 ng•h/mL. Throughout the conduct of the study, the pharmacokinetics of ALS-008112 and ALS-008144 will be evaluated and reviewed. An established pediatric PK model will be updated with the additional data, and the plasma exposures of the subsequent dose will be simulated prior to dose escalation, as a safety check to ensure that the pharmacokinetics of ALS-008112 are not predicted to differ significantly from the intended exposures.<br><br>See also Section 5.1.1, Cohort Progression Guidelines.	
**2.3 *Part 2: Multiple Ascending Dose (SAD)***	Within each dosing cohort, subjects will be randomized to receive either ALS-008176 or placebo (n = approximately 24 per cohort; randomized in a ratio of 3 ALS-008176:1 placebo). Randomization	Within each dosing cohort, subjects will be randomized to receive either ALS-008176 or placebo (n = approximately 24 per cohort; randomized in a ratio of 3 ALS-008176:1 placebo).	Based on internal review and scientific advice from health authorities, the age strata were

Section	Original Text in Version 1.0	Revised Text in Version 2.0	Reason for Change
(¶4)	will be stratified by age at time of hospital admission (≥ 1.0 to < 3.0 months, ≥ 3.0 to < 6.0 months, and ≥ 6.0 to ≤ 12.0 months), with approximately 8 subjects being enrolled in each stratum. Additionally, all subjects will receive standard supportive care as per local institution. Subjects will be evaluated over an 11-day period from the time of randomization. If they are discharged from the hospital prior to Day 11, they will be required to return for assessment as an outpatient on Day 5, if applicable, and on Day 11, when they will complete the study.	Randomization will be stratified by age at time of hospital admission (≥ 1.0 to < 2.0 months, ≥ 2.0 to < 6.0 months, and ≥ 6.0 to ≤ 12.0 months), with approximately 8 subjects being enrolled in each stratum. Additionally, all subjects will receive standard supportive care as per local institution. Subjects will be evaluated over an 11-day period from the time of randomization. If they are discharged from the hospital prior to Day 11, they will be required to return for assessment as an outpatient on Day 5, if applicable, and on Day 11, when they will complete the study.	refined.
**3.2.3** ***Exploratory Endpoints*** *(last bullet(s) of list)*	• Time to resolution of RSV symptoms, such as runny nose, wheeze, cough, tachypnea Biomarkers potentially associated with the inflammatory response induced by acute RSV infection	• Time to resolution of RSV symptoms, such as runny nose, wheeze, cough, tachypnea<br>• Biomarkers potentially associated with the inflammatory response induced by acute RSV infection	Typographical error
**5.1.1 Cohort Progression Guidelines** *(¶s 1–5)*	In both Parts 1 and 2, the planned dose escalation schema (Figure 2-1) may be modified by the Sponsor, upon approval by the IDMC, based on emerging PK and safety data. In all circumstances, however, the dosing schema will have no more than a three-fold increase in dose between dose levels. In addition, under no circumstances will a planned pediatric dose exceed a projected average ALS-008112 AUC_{0-24} of 20,000 ng•h/mL. Furthermore, in both Parts 1 and 2, the decision to 1) trigger the remainder of the first SAD cohort following the evaluation of the Sentinel group (see Section 2.2) and 2) dose escalate between cohorts will be based on a review of safety and PK data by the Sponsor and IDMC:<br>• Part 1 – Sentinel Group – all available safety data through study completion for all 5 subjects, as well as all available PK data for the first 24 hours after	In both Parts 1 and 2, the planned dose escalation schema (Figure 2-1) may be modified by the Sponsor, upon approval by the IDMC, based on emerging PK and safety data. In all circumstances, however, the dosing schema will have no more than a three-fold increase in dose between dose levels. In addition, under no circumstances will a planned pediatric dose exceed a projected average ALS-008112 AUC_{0-24} of 20,000 ng•h/mL. Furthermore, the decisions to advance the study will be based on a review of safety and PK data by the Sponsor and IDMC as follows:<br>• Part 1 – Trigger the enrollment of the remainder of each SAD age stratum, and the next age stratum sentinel group, following the evaluation of each Sentinel Group (see Section 2.2)– all available safety data through study completion for all 3 subjects in the age stratum, as well as	The criteria for advancing the study were modified to reflect the updated design of the study after addition of age de-escalation strategy and use of sentinel cohorts.

Section	Original Text in Version 1.0	Revised Text in Version 2.0	Reason for Change
	dosing.<br>• Part 1 – all available safety data through study completion for all subjects, as well as PK data for the first 24 hours after dosing for at least 75% of subjects in the most recent cohort<br>• Part 2 – all available safety data through study completion for all subjects and PK data up to 120 hours post-dose (5 days) for 75% of the subjects in the most recent cohort<br>The Sponsor, upon approval by the IDMC, may advance an age group(s) to the next dosing cohort before enrollment in other age groups within that dose cohort is complete. However, no age stratum will be advanced until that entire age stratum has completed dosing at the current dose level and safety and PK data have been assessed.	all available PK data for the first 24 hours after dosing will be reviewed.<br>• Part 1 –Dose Escalations - all available safety data through study completion for all subjects, as well as available PK data for the first 24 hours after dosing for at least 75% of subjects in the most recent cohort will be reviewed.<br>• Part 2 – Dose Escalations - all available safety data through study completion for all subjects, as well as available PK data up to 120 hours post-dose (5 days) for at least 75% of the subjects in the most recent cohort will be reviewed.<br>The Sponsor, upon approval by the IDMC, may advance an older age group(s) to the next dosing cohort before enrollment in younger age group(s) within that dose cohort is complete. However, no age stratum will be advanced to the next dose until that entire age stratum has completed dosing at the current dose level and safety and PK data have been assessed. At no time will dose escalation occur for a younger age stratum before dosing in an older age stratum has been initiated for that dose.	
**5.2.1 Study Drug** (¶ 4)	Each dose will be administered using an appropriate volume amber-colored polypropylene oral dosing syringe.	Each dose will be administered using an appropriate volume colored polypropylene oral dosing syringe.	To allow for differences in country or site requirements
**6.0 Study Schedule** Table 6-1 Schedule of Events SAD Phase (Part 1) and Table 6-2 Schedule of Events MAD Phase (Part 2) footnote 2	2. If subject remains hospitalized after Day 6, conduct the Completion Visit assessments on Day 7 (±1 day). Hospitalization duration will not be extended solely for study purposes.	2. If subject remains hospitalized after Day 6, conduct the Completion Visit assessments on Day 7 (±1 day). Hospitalization duration will not be extended solely for study purposes. Telephone calls to parents to facilitate safety monitoring of outpatients between study visits are permitted.	Clarification that telephone calls between study visits are permitted

Section	Original Text in Version 1.0	Revised Text in Version 2.0	Reason for Change
**7.2.2 Documenting and Reporting Adverse Events** (new ¶ 1)	NA	Telephone calls to parents to facilitate safety monitoring of outpatients between study visits are permitted.	Clarification that telephone calls between study visits are permitted
**7.5 Independent Data Monitoring Committee (1st bullet)**	• Opening further enrollment based on acceptable safety and PK profile in SAD Cohort 1 Sentinel group	• Opening further enrollment based on acceptable safety and PK profile in SAD Sentinel groups	Modified to reflect the updated design of the study after addition of age de-escalation strategy and use of sentinel cohorts.
**9.1 Study Design and Objectives** (¶ 1)	This randomized, double-blind, placebo-controlled, 2-part study will assess the safety, tolerability, pharmacokinetics (PK), and pharmacodynamics (PD) of single and multiple doses of orally administered ALS-008176 in infants hospitalized with RSV infection. Each infant will only be enrolled in a single cohort and a single part of the study. In each cohort of both Part 1 (single ascending dose – SAD) and Part 2 (multiple ascending dose – MAD), a stratified randomization will be used on the basis of age, with the 3 strata being defined as follows: infants (≥ 1.0 to < 3.0 months, ≥ 3.0 to < 6.0 months, and ≥ 6.0 to ≤ 12.0 months). Each stratum within each ascending dose cohort will consist of 8 infants, with 6 being randomized to receive ALS-008176 and 2 being randomized to receive placebo. If multiple subjects are eligible for randomization on the same day to the last slot within a stratum, then up to an additional 4 subjects will be allowed to be randomized on that day to that stratum (3:1 allocation).	This randomized, double-blind, placebo-controlled, 2-part study will assess the safety, tolerability, pharmacokinetics (PK), and pharmacodynamics (PD) of single and multiple doses of orally administered ALS-008176 in infants hospitalized with RSV infection. Each infant will only be enrolled in a single cohort and a single part of the study. In each cohort of both Part 1 (single ascending dose – SAD) and Part 2 (multiple ascending dose – MAD), a stratified randomization will be used on the basis of age, with the 3 strata being defined as follows: infants (≥ 1.0 to < 2.0 months, ≥ 2.0 to < 6.0 months, and ≥ 6.0 to ≤ 12.0 months). Each stratum within each ascending dose cohort will consist of 8 infants, with 6 being randomized to receive ALS-008176 and 2 being randomized to receive placebo. If multiple subjects are eligible for randomization on the same day to the last slot within a stratum, then up to an additional 4 subjects will be allowed to be randomized on that day to that stratum (3:1 allocation).	Based on internal review and scientific advice from health authorities, the age strata were refined.
**9.5 Randomization**	In each cohort of both Part 1 (single ascending dose – SAD) and Part 2 (multiple ascending dose – MAD), a stratified randomization will be used on the basis of age at the time of hospital admission, with the 3 strata	In each cohort of both Part 1 (single ascending dose – SAD) and Part 2 (multiple ascending dose – MAD), a stratified randomization will be used on the basis of age at the time of hospital admission,	Based on internal review and scientific advice from health authorities, the age strata were

Section	Original Text in Version 1.0	Revised Text in Version 2.0	Reason for Change
	being defined as follows: (≥ 1.0 to < 3.0 months, ≥ 3.0 to < 6.0 months, and ≥ 6.0 to ≤ 12.0 months). Each stratum within each ascending dose cohort will consist of approximately 8 infants, with 6 being randomized to receive ALS-008176 and 2 being randomized to receive placebo. If multiple subjects are eligible for randomization on the same day to the last slot within a stratum, then up to an additional 4 subjects will be allowed to be randomized on that day to that stratum (3:1 allocation).<br><br>Initially, a sentinel group of 5 subjects aged ≥ 6.0 to ≤ 12.0 months will be enrolled in the first SAD dosing cohort (randomized in a ratio of 4 ALS-008176:1 placebo). The PK data from the sentinel group will be used to confirm or adjust the predictions of the pediatric PK model before any additional subjects are enrolled (see also Section 1.3, Rationale for Dose Selection). After IDMC review of these data, the remaining 3 subjects in the first ≥ 6.0 to ≤ 12.0 months SAD cohort will be randomized (in a ratio of 2 ALS-008176:1 placebo) and all subjects in the lower age groups of the first dosing cohort will be enrolled.<br><br>The dose for the remaining subjects in this and future dosing cohorts may be revised based on data derived from the sentinel subjects.	with the 3 strata being defined as follows: (≥ 1.0 to < 2.0 months, ≥ 2.0 to < 6.0 months, and ≥ 6.0 to ≤ 12.0 months). Each stratum within each ascending dose cohort will consist of approximately 8 infants, with 6 being randomized to receive ALS-008176 and 2 being randomized to receive placebo. If multiple subjects are eligible for randomization on the same day to the last slot within a stratum, then up to an additional 4 subjects will be allowed to be randomized on that day to that stratum (3:1 allocation).<br><br>An age de-escalation approach will be utilized within each SAD dose cohort, i.e. subjects aged ≥ 6.0 to ≤ 12.0 months will be enrolled initially, followed by subjects ≥ 2.0 to < 6.0 months, and then subjects ≥ 1.0 to < 2.0 months, as follows:<br>• A sentinel group of 3 subjects in each age stratum will be enrolled first (randomized in a ratio of 2 ALS-008176:1 placebo).<br>• The IDMC will review the safety data through Day 7 and available PK data for the sentinel subjects. Following this:<br>○ The remaining 5 subjects in the age stratum will be enrolled (randomized in a ratio of 4 ALS-008176:1 placebo), and<br>○ Enrollment in the next age stratum's sentinel cohort(s) will be initiated.<br>At no time will dose escalation occur for a younger age stratum before dosing in an older age stratum has been initiated for that dose.<br><br>If a subject discontinues prematurely due to a non-safety related reason, that subject may be replaced.<br><br>As part of the randomization schedule for treatment	refined.<br>Based on internal review and scientific advice from health authorities, sentinel groups and age de-escalation in the SAD part have been added as an additional safety precaution.<br>Clarification of the randomization scheme.

Section	Original Text in Version 1.0	Revised Text in Version 2.0	Reason for Change
		assignment, all subjects will also be randomized to a PK sample collection schedule within each of the 2 treatment groups in each of the 3 age strata. The PK of ALS-008112 and ALS-008144 (and other metabolites, if applicable) will be determined using a sparse sampling strategy whereby each subject within an age stratum will be assigned to have PK samples drawn at specific time points which, when combined across subjects, will result in full PK profiles for the stratum.<br><br>The subjects within a single age stratum and cohort will be randomized to a PK sample collection schedule in the following ratio: 3 ALS-008176 PK Group A: 3 ALS-008176 PK Group B: 1 Placebo PK Group A: 1 Placebo PK Group B.<br><br>Among the two subjects randomized to ALS-008176 treatment in each sentinel group, 1 subject each will be randomized to PK Group A and PK Group B.	
**Appendix A,** Investigator Signature Page	Version 1.0, 12 December 2013	Version 2.0, 19 December 2013	Reflects new protocol version number.

Appendix E. Summary of Protocol Changes from Version 2.0 dated 19 December 2013 to Version 3.0

Section	Original Text in Version 2.0	Revised Text in Version 3.0	Reason for Change
**Cover page**<br>*Date and Version Number*<br>**Appendix A,**<br>*Investigator Signature Page*	19 December 2013<br>Version 2.0<br><br>Drug name: ALS-008176	17 December 2014<br>Version 3.0<br><br>Drug name: ALS-008176<br>(Also known as AL-8176)	To reflect revised version of protocol<br><br>Added another name for study drug
**CONTACT INFORMATION**	[redacted]	[redacted]	Administrative update
	One hundred healthy adult volunteers were enrolled in the first-in-human single ascending dose (SAD), food effect, and multiple ascending dose (MAD) study (Study ALS-8176-501); 75 and 25 subjects received ALS-008176 and placebo, respectively. Single doses up to 750 mg and multiple doses up to 750 mg twice daily, 12 hours apart (Q12) on Day 1 followed by 500 mg Q12 from Day 2 to Day 5 (MAD Cohorts 1-4) or Day 2 to Day 14 (MAD Cohort 5) have been administered in the SAD and MAD, respectively. In this study, unblinded data are available for all SAD and food effect cohorts and MAD Cohorts 1–4. In these cohorts, ALS-008176 was well tolerated, with no serious adverse events being reported and no adverse events leading to discontinuation or requiring concomitant medications. All adverse events in these groups were mild in severity and the only adverse events which occurred more than once were headache (7 events – 5 ALS-008176 vs. 2 Placebo) and hot flushes (3 events - 2 ALS-008176 vs. 1 Placebo). Single adverse events of toothache and sore throat were also reported once each in ALS-008176	Clinical Data – Safety<br><br>One hundred one healthy adult volunteers were enrolled in the first-in-human single ascending dose (SAD), food effect, and multiple ascending dose (MAD) study (Study ALS-8176-501); 76~~5~~ and 25 subjects received ALS-008176 and placebo, respectively. Single doses up to 750 mg and multiple doses up to 750 mg twice daily, 12 hours apart (Q12) on Day 1 followed by 500 mg Q12 from Day 2 to Day 5 (MAD Cohorts 1-4) or Day 2 to Day 14 (MAD Cohort 5) have been administered in the SAD and MAD, respectively. ~~In this study, unblinded data are available for all SAD and food effect cohorts and MAD Cohorts 1–4.~~ In ~~these cohorts~~this study, ALS-008176 was well tolerated, with no serious adverse events being reported and no adverse events leading to discontinuation ~~or requiring concomitant medications~~of study drug. All reported adverse events (N=18) ~~in these groups~~ were mild (N=14) or moderate (N=4) in severity, with the following mild adverse events being reported:	Updated clinical safety and pharmacokinetics data from other ALS-008176 clinical trials is provided

Section	Original Text in Version 2.0	Revised Text in Version 3.0	Reason for Change
	treatment groups.<br>In MAD Cohort 5, 14 days of dosing with ALS-008176 was associated with the following blinded adverse event profile (N = 12 subjects): headache (2 events); soft stools (2 events); intermittent abdominal pain (1 event); and paresthesias in bilateral calf muscles (1 event). Four of these events (headache, intermittent abdominal pain, soft stools, paresthesias) were moderate in severity, with the remaining events being mild. None of these adverse events was considered an SAE or led to study drug discontinuation.<br>In all cohorts up to and including MAD Cohort 5, no clinically significant laboratory, electrocardiogram (ECG), vital sign, or physical exam findings were noted at any dose evaluated.	headache (8 events), hot flush (3 events), and toothache, sore throat, and diarrhea, each of which occurred as a single event. The 4 moderate events reported were paresthesia and headache in 2 subjects treated with ALS-008176 and abdominal pain and diarrhea in a placebo-treated subject. No important imbalances in any adverse events were observed in ALS-008176-treated vs. placebo-treated subjects. in severity and the only adverse events which occurred more than once were headache (7 events – 5 ALS-008176 vs. 2 Placebo) and hot flushes (3 events - 2 ALS-008176 vs. 1 Placebo). Single adverse events of toothache and sore throat were also reported once each in ALS-008176 treatment groups.<br>In MAD Cohort 5, 14 days of dosing with ALS-008176 was associated with the following blinded adverse event profile (N = 12 subjects): headache (2 events); soft stools (2 events); intermittent abdominal pain (1 event); and paresthesias in bilateral calf muscles (1 event). Four of these events (headache, intermittent abdominal pain, soft stools, paresthesias) were moderate in severity, with the remaining events being mild. None of these adverse events was considered an SAE or led to study drug discontinuation.<br>In all cohorts up to and including MAD Cohort 5Additionally, no clinically significant laboratory, electrocardiogram (ECG), vital sign, or physical exam findings were noted at	

Section	Original Text in Version 2.0	Revised Text in Version 3.0	Reason for Change
		any dose evaluated.<br><br>An RSV challenge study (Study ALS-8176-502) evaluating three different 5 day ALS-008176 regimens vs. placebo was also conducted in 62 (44 ALS-008176: 18 Placebo) healthy volunteers. Preliminary data indicate that ALS-008176 rapidly and potently inhibited viral replication with an accompanying reduction in signs and symptoms of RSV among infected subjects (for a detailed discussion, see the Addendum to V4 of the Investigator's Brochure). Safety data indicate that there were no serious adverse events (SAEs), premature discontinuations of study drug, or clinically significant, treatment related adverse events in any study participants. Seventy-nine treatment-emergent AEs were reported; these were generally balanced for frequency and intensity across ALS-008176 and placebo recipients and all but two were mild (N=72) or moderate (N=5) in severity. The 2 severe (Grade 3) adverse events were asymptomatic laboratory abnormalities: increased ALT (in a subject treated with ALS-008176 - 750 mg loading dose/500 mg maintenance dose) and increased creatine kinase (in a subject treated with ALS-008176 750 mg loading dose/150 mg maintenance dose, who had experienced strenuous exercise prior to the event). These 2 events were considered by the blinded Investigator to be unlikely and not related to study drug, respectively. The most commonly reported AEs (≥2 participants reporting an event in any one treatment group) are summarized in Table 5 of the Addendum to V4 of the Investigator's	

Section	Original Text in Version 2.0	Revised Text in Version 3.0	Reason for Change
		Brochure. No clinically relevant laboratory, electrocardiogram, vital sign, or physical exam findings were identified.<br><br>An open label, single dose study to evaluate the absolute bioavailability and mass balance of ALS-008176 (Study ALS-8176-504) was conducted in 12 healthy volunteers. Preliminary data indicate that no adverse events were reported in this study.	
	In healthy adult subjects, ALS-008176 was not detected in plasma after dosing. Time to the maximum plasma concentration (Tmax) of ALS-008112 was reached rapidly, within approximately 15-30 minutes, indicating good oral absorption of ALS-008176 and effective conversion to ALS-008112. The only other major metabolite in plasma was ALS-008144, the inactive uridine metabolite of ALS-008112. While the terminal half life of ALS-008112 was calculated to be 63 hours, ALS-008112 plasma concentrations decreased to very low levels within 6 hours post dose, indicating rapid and extensive distribution of ALS-008112. Following single and multiple doses, the AUC of ALS-008112 and ALS-008144 increased linearly but less than dose proportionally with increasing doses. The highest exposure of ALS-008112 obtained in adults was an average Cmax of 4069 ng/mL (fasted state at 750 mg) and an average AUC0-24h of 12135 ng·h///mL (fed state 750 mg Q12). No marked accumulation of either ALS-008112 or ALS-008144 was noted following multiple doses of ALS-008176. Administration of ALS-008176 following either a high fat, high	Clinical Data – Pharmacokinetics<br><br>In healthy adult subjects, ALS-008176 was not detected in plasma after dosing. Time to the maximum plasma concentration (T_{max}) of ALS-008112 was reached rapidly, within approximately 15-30 minutes, indicating good oral absorption of ALS-008176 and effective conversion to ALS-008112. The only other major metabolite in plasma was ALS-008144, the inactive uridine metabolite of ALS-008112. While the terminal half life of ALS-008112 was calculated to be 63 hours, ALS-008112 plasma concentrations decreased to very low levels within 6 hours post dose, indicating rapid and extensive distribution of ALS-008112. Following single and multiple doses, the AUC of ALS-008112 and ALS-008144 increased linearly but less than dose proportionally with increasing doses. The highest exposure of ALS-008112 obtained in adults was an average C_{max} of 4069 ng/mL (fasted state at 750 mg) and an average AUC_{0-24h} of 12135 ng·h/mL (fed state 750 mg Q12). No marked accumulation of either	Updated clinical safety and pharmacokinetics data from other ALS-008176 clinical trials is provided.

Section	Original Text in Version 2.0	Revised Text in Version 3.0	Reason for Change
	calorie diet (after a single dose) or a normal diet (after multiple doses), lowered the plasma Cmax of ALS-008112 by approximately 50% without affecting the AUC. Approximately 25% of the administered dose is eliminated in the urine.	ALS-008112 or ALS-008144 was noted following multiple doses of ALS-008176. Administration of ALS-008176 following either a high fat, high calorie diet (after a single dose) or a normal diet (after multiple doses), lowered the plasma C_{max} of ALS-008112 by approximately 50% without affecting the AUC. ~~Approximately 25% of the administered dose is eliminated in the urine.~~Preliminary data from Study ALS-8176-504 indicate that ALS-008176 is ~64% bioavailable and primarily (~80%) eliminated through the kidneys.	
**SYNOPSIS, Figures 1-1 and 2-1,** *Dosing Schema;*<br>**1.3.1** *Single Ascending Dose (SAD);*<br>**1.3.2** *Multiple Ascending Dose (MAD);*<br>**2.1** *SUMMARY;*<br>**Table 5-1** *Part 1 SAD Dosing Regimen;*<br>**Table 5-2** *Part 2 MAD Dosing Regimen;*	Figure Title: Dosing Schema<br><br>SAD Dose Level 2 is 3.5 mg/kg<br>MAD Dose Level 1 is 3.5mg/kg x 1 dose + 1.5 mg/kg Q12 x 4.5 days<br><br>1.3.1 (SAD): The proposed dose escalation to 3.5, 7 and 10 mg/kg in subsequent cohorts…<br><br>1.3.2 (MAD): For cohort 1, a single loading dose of 3.5 mg/kg followed by 1.5 mg/kg…<br><br>2.1: The dose escalation schema is presented in…<br><br>Table 5-1:	Figure Title: Preliminary Dosing Schema*<br>Added to footnote*:<br>Doses subject to change based on IDMC review of emerging safety and PK data.<br>SAD Dose Level 2 is 4.5 mg/kg<br>MAD Dose Level 1 is 4.5mg/kg x 1 dose + 1.5 mg/kg Q12 x 4.5 days<br><br>1.3.1 (SAD): The proposed dose escalation to ~~3.5~~4.5, 7 and 10 mg/kg in subsequent cohorts…<br><br>1.3.2 (MAD): For cohort 1, a single loading dose of ~~3.5~~4.5 mg/kg followed by 1.5 mg/kg…<br><br>2.1: The preliminary dose escalation schema is presented in…	The dosing schema in Figure 1-1 and 2-1 is a preliminary schema based on expected (modeled) PK exposures and acceptable safety. The actual doses to be administered at each dose level are subject to IDMC instructions based on a review of emerging actual PK and safety data. The figure was updated to reflect that the IDMC instructed the 2nd dose level to be increased to 4.5 mg/kg. The rationale for this change is based on the fact that, in general, lower doses are expected to expose more study participants to potentially subtherapeutic levels of ALS-008176 and the observation that some exposures were slightly lower than expected based on the current pediatric population PK model. The figure was also updated to more explicitly reflect the idea that the

Section	Original Text in Version 2.0	Revised Text in Version 3.0	Reason for Change
	SAD Cohort No. 2: 18 subjects to receive single oral 3.5 mg/kg dose of ALS-008176 with 6 subjects receiving placebo<br><br>Table 5-2:<br>MAD Cohort No. 1:…A loading dose of 3.5 mg/kg will be administered for Dose 1…	Table 5-1:<br>SAD Cohort No. 2: 18 subjects to receive single oral ~~3.5~~4.5 mg/kg dose of ALS-008176 with 6 subjects receiving placebo<br><br>Table 5-2:<br>MAD Cohort No. 1:…A loading dose of ~~3.5~~4.5 mg/kg will be administered for Dose 1…	doses depicted are preliminary and subjects to change based on IDMC review.
**SYNOPSIS**, *Study Design,* **2.2**, *Part 1: Single Ascending Dose (SAD),* **2.3**, *Part 2: Multiple Ascending Dose (MAD),* **9.5**, *Randomization*	Randomization will be stratified by age at time of hospital admission (≥ 1.0 to < 2.0 months, ≥ 2.0 to < 6.0 months, and ≥ 6.0 to ≤ 12.0 months), with approximately 8 subjects being enrolled in each stratum.	Randomization will be stratified by age at time of hospital admission (≥ 1.0 to < 2.0 months = 28 to 59 days old, ≥ 2.0 to < 6.0 months = 60 to 181 days old, and ≥ 6.0 to ≤ 12.0 months = 182 to 365 days old), with approximately 8 subjects being enrolled in each stratum.	Definition of the age groups in days
**SYNOPSIS**, *Study Design,* **2.2**, *Part 1: Single Ascending Dose (SAD),***5.1.1**, *Cohort Progression Guidelines*	At no time will dose escalation occur for a younger age stratum before dosing in an older age stratum has been initiated for that dose.	At no time will dose escalation occur for a younger age stratum before dosing in an older age stratum has been initiated for that dose, unless the IDMC determined that the safety profile supported such an approach.	The intent of the protocol is to employ age de-escalation strategy throughout the protocol. This change was added to the protocol in the unlikely event that enrollment in the trial is stalled for a prolonged period because older otherwise healthy infants are not available to be randomized and all younger groups are fully enrolled. This is a theoretical risk in certain regions of the world (e.g., the UK), where the average

Section	Original Text in Version 2.0	Revised Text in Version 3.0	Reason for Change
			age of enrollment for otherwise healthy infants can be 1-2 months. This option would only be utilized with the approval of the IDMC.
**SYNOPSIS,** Number of Sites and Location	Approximately 28 sites in Europe, Asia Pacific, and Latin America (specific countries to be determined) will participate.	Approximately 50~~28~~ sites in Europe, Asia Pacific, South Africa, and North/Latin America (specific countries to be determined) will participate.	More sites and South Africa will be added to the study to assist with enrollment.
**SYNOPSIS, 4.2,** *Inclusion Criteria*	3. Male or female infant who<br>• has been diagnosed with RSV infection based on study-supplied BINAX NOW RSV test. NOTE: A subject remains eligible if the BINAX NOW RSV result is negative but a RSV-specific PCR assay run locally is positive. (RSV-specific PCR run locally is not required.) Coinfection with other respiratory viruses or bacterial coinfection in addition to RSV infection is permissible.<br>• has been hospitalized for < 48 hours for confirmed RSV infection<br>• has had symptoms consistent with RSV infection (e.g., runny nose, cough,	3. Male or female infant who<br>• has been diagnosed with RSV infection based on study-supplied BINAX NOW RSV test or an RSV PCR assay conducted at the clinical trial site. NOTE: A subject remains eligible if the BINAX NOW RSV result is negative but a RSV-specific PCR assay run locally is positive. RSV-specific PCR run locally is not required.) Coinfection with other respiratory viruses or bacterial coinfection in addition to RSV infection is permissible.<br>• has been hospitalized for < 96~~48~~ hours for confirmed RSV infection (NOTE: nosocomial RSV infection is excluded)<br>~~• has had symptoms consistent with RSV infection (e.g., runny nose, cough, sneezing, fever, and tachypnea) for ≤ 5 days~~	If hospital lab has RSV PCR capability and this test is standard of care at the hospital, an additional procedure to collect specimen for BINAX NOW RSV test is not required.<br><br>The duration of hospitalization was changed from <48 hours to <96 hours because many sites were reporting an inability to enroll subjects admitted over weekends due to lack of weekend staff coverage. The note that nosocomial RSV infection was excluded was added to be explicit that children should have been admitted due to their RSV infection, not for other reasons, which were then complicated subsequently by a nosocomial infection.<br>The requirement for symptoms<5 days was deleted based on feedback from sites across the globe that first symptom onset has been difficult to ascertain and has

Section	Original Text in Version 2.0	Revised Text in Version 3.0	Reason for Change
	sneezing, fever, and tachypnea) for ≤ 5 days		often been confounded by infants who appear to have had multiple "back to back," overlapping infections such that parents cannot identify the first symptom for the RSV infection per se.<br>Deletion of this requirement is not considered to adversely affect subjects because, clinically, subjects with symptoms for >5 days who present to the hospital with ongoing symptoms, an indication for hospitalization, and confirmed RSV infection are highly likely to remain acutely infected and thus to potentially benefit from the antiviral properties of ALS-008176.
**SYNOPSIS, 4.3,** *Exclusion Criteria*	1.3. Body weight < 10th percentile for age	~~1.3. Body weight < 10th percentile for age~~	This criterion has resulted in the exclusion of small, full term otherwise healthy infants, which is unnecessary. This is particularly true since study drug dose takes into account body weight.
**SYNOPSIS, 4.3,** *Exclusion Criteria*	10. Exclusionary medications include:<br>• Herbal supplements within 21 days prior to randomization	10. Exclusionary medications include:<br>• Herbal supplements which have evidence of adversely affecting absorption and clearance mechanisms (e.g., strong inhibitors/inducers of CYP450) within 21 days prior to randomization<br>• The following prescription medications:<br>a. Any chronically used, systemic	Clarification of excluded herbal supplements to be clear that only those herbals with known potential to interact with physiologic absorption and clearance mechanisms, which may result in drug-drug interactions, are prohibited.

Section	Original Text in Version 2.0	Revised Text in Version 3.0	Reason for Change
	• The following prescription medications:<br>a. Any chronically used, systemic prescription medications<br>b. Use of systemic medications (either chronically or within the 21 days prior to randomization) which are known to modulate the host immune response and/or increase viral shedding such as corticosteroids or other immunomodulatory therapies.	prescription medications<br>b. Use of systemic medications (either chronically or within the 21 days prior to randomization) which are known to modulate the host immune response and/or increase viral shedding such as corticosteroids or other immunomodulatory therapies. Systemic corticosteroids administered before study randomization will be acceptable if:<br>• The dose is ≤ 2 mg/kg and ≤20 mg/day of prednisolone or equivalent, and<br>• The corticosteroids are given as a treatment for the subject's RSV symptoms, and<br>• The corticosteroids have not been administered for more than a total of 4 days.<br>• NOTE: Eligible subjects should not receive study medication for at least 12 hours after the last dose of any systemically administered corticosteroids, and no planned further administration of	Globally, patients have been commonly receiving a few, low doses of corticosteroids (generally due to wheezing) which are unlikely to result in clinical immunsuppression. Nevertheless, these subjects are required to be excluded on the grounds that they may be immunosuppressed. The exclusion criterion was thus updated to permit low doses of systemically administered corticosteroids, so long as the doses are considered to be unlikely to result in clinical immunosuppression. The permitted doses of corticosteroids were chosen based on the fact that the United States Centers for Disease Control (CDC) considers that doses equivalent to either ≥ 2 mg/kg of body weight or ≥ 20 mg/day of prednisone or equivalent for persons who weigh >10 kg when administered for ≥14 days as sufficiently immunosuppressive to raise concern about the safety of vaccination with live-virus vaccines.[1]<br>[1] General recommendations on immunization: recommendations of the Advisory Committee on Immunization Practices (ACIP). *MMWR* 2011;60 (no.2) – http://www.cdc.gov/mmwr/pdf/rr/

Section	Original Text in Version 2.0	Revised Text in Version 3.0	Reason for Change
		corticosteroids is permitted during the study.	rr6002.pdf
**5.3**, *Dose Preparation and Administration*	Although prepared study drug suspension and placebo suspension are stable between 15°C and 30°C for 5 days in stability testing, refrigeration (2°C and 8°C) of prepared study drug(s) is recommended.	Although prepared study drug suspension and placebo suspension are stable between 15°C and 30°C ~~for 5 days in stability testing~~, refrigeration (2°C and 8°C) of prepared study drug(s) is recommended. Refer to Pharmacy Manual for the most current stability information.	Clarification that current stability information will be reflected in the Pharmacy Manual.
**5.7**, *Concomitant Medications*	Prescription medications intended to treat the symptoms/sequelae of the RSV infection are permitted, including:<br>• Oral antibiotics such as beta-lactams and cephalosporins	Prescription medications intended to treat the symptoms/sequelae of the RSV infection are permitted, including:<br>• ~~Oral a~~Antibiotics such as beta-lactams and cephalosporins	Clarification that use of antibiotics is permitted, regardless of route of administration, so long as they are not on the prohibited medication list.
**5.8**, *Prohibited Medications*	Prohibited medications during the conduct of this study include:<br>• Herbal supplements	Prohibited medications during the conduct of this study include:<br>• Herbal supplements which have evidence of adversely affecting absorption and clearance mechanisms (e.g., strong inhibitors/inducers of CYP450). Herbal supplements which are not likely to adversely affect either absorption or clearance mechanism of ALS-008176 during dosing may be permitted on a case by case basis. These cases will need to be	Clarification of excluded herbal supplements due to the potential to interact with physiologic absorption and clearance mechanisms which may result in drug-drug interactions

Section	Original Text in Version 2.0	Revised Text in Version 3.0	Reason for Change
		reviewed and approved by the Sponsor Medical Monitor. The decision to allow the use of a particular herbal supplement will be based on a review of the available scientific information on the product.	
**Table 6-1** *Schedule of Events SAD Phase (Part 1),* **Table 6-2** *Schedule of Events MAD Phase (Part 2)*	Nasopharyngeal Swab: RSV Diagnosis (Binax NOW RSV)	Nasopharyngeal Swab or Nasal Aspirate: RSV Diagnosis (Binax NOW RSV)	BinaxNOW RSV test package insert allows sample collection via nasopharyngeal swab or nasal wash (which study instructions refer to as nasal aspirate).
**Table 6-1** *Schedule of Events SAD Phase (Part 1) Footnote 8,* **Table 6-2** *Schedule of Events MAD Phase (Part 2) Footnote 7*	Nasal aspirates, serum chemistries, hematology during hospitalization are not required after 24 hours post-dose. However, if blood collection or nasal aspiration is performed during routine clinical care while hospitalized, "scavenging" of such specimens for additional viral, safety, and biomarker analysis is permitted.	Nasal aspirates, serum chemistries, hematology during hospitalization are not required after 24 hours post-dose. However, if blood collection or nasal aspiration is performed during routine clinical care while hospitalized, "scavenging" of such specimens for additional viral, safety, PK and biomarker analysis is permitted.	Added the possibility of PK analysis of scavenged samples.
**6.1.5,** *RSV Evaluations: Qualitative PCR*	Qualitative PCR will be performed by a central lab, using pre-dose nasal aspirate sample, to confirm RSV diagnosis, however, study eligibility is determined by the Binax NOW RSV rapid test result.	Qualitative PCR will be performed by a central lab, using pre-dose nasal aspirate sample, to confirm RSV diagnosis, however, study eligibility is determined by the results of the Binax NOW RSV rapid test or a RSV PCR assay conducted at the clinical trial site~~result~~. Samples from BinaxNOW RSV negative screening tests may be sent to the central lab for evaluation using a qualitative PCR assay.	Consistency with inclusion criterion 3<br><br>The BINAX NOW test is an easy to use rapid diagnostic test that is comparatively less sensitive than PCR testing. The option to test samples that were BINAX NOW negative via a central-lab performed PCR assay was added to enable the sponsor to determine

Section	Original Text in Version 2.0	Revised Text in Version 3.0	Reason for Change
		This optional sample evaluation process will be conducted at selected centers.	the robustness of the BINAX NOW assay and estimate the false negative rate for BINAX NOW in this trial.
**9.9.4**, *Prior and Concomitant Medications*	Use of all medications administered from within 30 days prior to randomization will be recorded.	Use of all medications administered from the date the informed consent is signed through to the Completion Visit~~within 30 days prior to randomization~~ will be recorded.	Correction to be consistent with section 5.7, *Concomitant Medications* and 8.2.4, *Prior and Concomitant Medications*

Appendix F. Summary of Protocol Changes from Version 3.0 dated 17 December 2014 to Version 4.0

Section	Original Text in Version 3.0	Revised Text in Version 4.0	Reason for Change
Cover Page<br>IND Number<br>(cover page and page 11)	IND Number: This is a non-IND study	IND Number: Pending: Pre-IND Number 122,969~~This is a non IND Study~~	To reflect revised version
Date	Date: 17 December 2014	~~17 December 2014~~ 24 August 2015	
Version	Version 3.0	Version ~~3~~ 4.0	
Contact Information<br>*Medical Monitors*	Americas<br>[redacted] MD, FACP, [redacted], US<br>Telephone No.<br>Office: +[redacted]<br>Mobile: [redacted]<br>Email:<br>[redacted]m	Americas<br>[redacted], MD, FACP<br>[redacted], US<br>Office: +[redacted]<br>Mobile: [redacted]<br>Email: [redacted]	Administrative Change
	Europe<br>[redacted], MD, PhD<br>POLAND<br>Telephone No. [redacted]<br>Email:<br>[redacted]	Europe<br>[redacted], MD<br>SPAIN<br>Telephone No.: [redacted]	Administrative Change
	Sponsor Medical Director<br>[redacted]MD<br>[redacted], US<br>Telephone No. [redacted]	Sponsor Medical Director<br>[redacted], MD<br>[redacted] US<br>Telephone No. +[redacted]	

Section	Original Text in Version 3.0	Revised Text in Version 4.0	Reason for Change
Pharmacovigilance	[redacted] Tel No.: +[redacted]	[redacted] Tel No.: +[redacted]	Administrative Change
Abbreviations, 8.3			
Pharmacokinetic Measurements	CL/F Apparent oral clearance<br>Vd Volume of distribution<br>Vd_{ss} Steady-state volume of distribution<br>VD_{ss}/F The apparent volume of distribution at steady state	~~CL/F Apparent oral clearance~~<br>~~Vd Volume of distribution~~<br>~~Vd_{ss} Steady-state volume of distribution~~<br>~~VD_{ss}/F The apparent volume of distribution at steady state~~	Not applicable terms have been deleted.
Definitions			
A Day 28 Safety follow-up visit is being added to the MAD	End of clinical phase of study:<br>6 days after the last dose of study medication for the last subject treated, or resolution/stabilization of all AEs, whichever is later.	End of clinical phase of study:<br><u>Last MAD subject Day 28 follow up visit</u> ~~6 days after the last dose of study medication for the last subject treated,~~ or resolution/stabilization of all AEs, whichever is later.	The pediatric committee (PDCO) within the European Medicines Agency (EMA) and the Japanese health authority have requested that a longer follow up (i.e., 28 days) be conducted in the multiple dose part of this study. As a result, a MAD Day 28 visit is being added to further assess safety.
Figure 1-1, Figure 2-1 Preliminary Dosing Schema	Single Dose:<br>1.5, 4.5, 7, 10 mg/kg<br>Multiple Dose<br>N=24	Single Dose<br>~~1.5~~<u>1.37</u>, ~~4.5~~<u>4.1</u>, ~~7, 10~~<u>12</u> mg/kg<br>Multiple Dose<br>N=<u>≤</u>24<u>^</u><br><u>**Japanese infants will initially be enrolled in MAD in a separate cohort of up to 24 infants at a dose of 4.1 mg/kg x 1 dose + 1.37 mg/kg Q12 x 4.5 days</u><br><u>***Actual dose will be determined by the IDMC based on emerging data, but cannot exceed 3</u>	Diagram has been updated to reflect current plans for dose escalation.

Section	Original Text in Version 3.0	Revised Text in Version 4.0	Reason for Change
		times the dose evaluated in the first MAD cohort.<br>^ The decision to dose escalate in the MAD will be made by the IDMC based on emerging safety and PK data.	
SYNOPSIS,<br>***2.2 Study Design***<br>*Part 1 SAD*	**Part 1 (single ascending dose – SAD):**<br>Subjects will receive a single dose from 1 of 4 ascending dose levels of ALS-008176 (1.5 mg/kg, 4.5 mg/kg, 7mg/kg and 10mg/kg) or placebo. Dosing of study drug in the fed state is encouraged.<br>Two additional cohorts (Cohorts 5 and 6) may be evaluated at the discretion of the Sponsor, upon arrival by the Independent Data Monitoring Committee (IDMC), based on an evaluation of the emerging PK profile and the safety profile.	**Part 1 (single ascending dose -SAD):**<br>Subjects will receive a single dose from 1 of 3 ~~4~~ ascending dose levels of ALS-008176 (1.37 ~~5~~mg/kg, 4.1~~5~~ mg/kg, and 12 ~~7~~ mg/kg ~~and 10 mg/kg~~) or placebo. Dosing of study drug in the fed state is encouraged.<br>~~Two~~ Three additional cohorts (Cohorts 4, 5 and 6) may be evaluated at the discretion of the Sponsor, upon approval by the Independent Data Monitoring Committee (IDMC), based on an evaluation of the emerging PK profile and the safety profile.	During conduct of the second cohort of the SAD in Study ALS-8176-503, it was recognized that study documents instructed sites to re-suspend study drug with a greater volume than required to generate the target 10 mg/mL concentration. As a result, the actual concentration of ALS-008176 study drug was 9.1 mg/mL. Patients in the first 2 study cohorts were therefore administered 1.37 and 4.1 mg/kg instead of the intended doses of 1.5 and 4.5 mg/kg, respectively.<br>12 mg/kg is the third SAD dose level approved by the IDMC.
SYNOPSIS,<br>***2.3 Study Design,***<br>***Part 2 MAD***<br>Permitting IDMC to start MAD based on emerging safety and PK data	Part 2 will not commence before all safety data through study completion for all subjects in cohorts 1, 2 and 3 of Part 1, and PK data from the first 24 hours after dosing for at least 75% of the current cohorts' subjects (or a completed stratum within this cohort) have been evaluated and are deemed acceptable by the IDMC. MAD Cohort 2 will commence no earlier than after completion of SAD Cohort 4 and MAD Cohort 1.	Part 2 will commence when emerging PK and safety data from Part 1 have been evaluated and are deemed by the IDMC to be acceptable and sufficient to initiate the MAD.<br>~~Part 2 will not commence before all safety data through study completion for all subjects in cohorts 1, and 2 and 3 of Part 1, and PK data from the first 24 hours after dosing for at least 75% of the current cohort's subjects (or a completed stratum within this cohort) have been evaluated and are deemed acceptable by the IDMC. MAD Cohort 2 will commence no~~	Clinical trials in pediatrics have an obligation to advance to potentially efficacious dosing regimens as soon as the safety data suggest the risk profile of these regimens appears acceptable. The Sponsor considers the IDMC best suited to make this decision because it has the most comprehensive understanding of the unblinded safety profile of ALS-008176. The IDMC can best

Section	Original Text in Version 3.0	Revised Text in Version 4.0	Reason for Change
	Based on an acceptable safety profile in Part 1, as determined by the Sponsor and upon approval by the IDMC, subjects will receive 1 of 3 multiple ascending dose regimens of ALS-008176 or a placebo.<br><br>Hospitalized subjects with RSV infection will receive 1 of 3 multiple ascending dose regimens of ALS-008176 or placebo.<br><br>Study drug will be dosed twice daily (Q12) for a total of 10 doses administered over 5 days. Dosing of study drug in the fed state is encouraged.<br><br>In each dosing regimen, a single loading dose will be given as Dose 1 on Day 1. Following this, maintenance doses will be given Q12 (Doses 2–10) (Figure 2-1**Error! Reference source not found.**).<br><br>Part 2 will commence when emerging	earlier than after completion of SAD Cohort 4 and MAD Cohort 1.<br><br>Based on the lack of clinically relevant PK differences observed to date across the three age strata, infants of all 3 age strata will receive the same doses, unless otherwise instructed by the IDMC. Similarly, doses will be escalated in parallel in all three age strata for the second and subsequent MAD cohorts if the IDMC deems available PK and safety data in the current MAD cohort to be sufficient and authorizes dose escalation.<br><br>Based on an acceptable safety profile in Part 1, as determined by the Sponsor and upon approval by the IDMC, subjects will receive 1 of 3 multiple ascending dose regimens of ALS-008176 or a placebo.<br><br>Hospitalized subjects with RSV infection will receive 1 of 32 planned multiple ascending dose regimens of ALS-008176 or placebo.<br><br>Study drug will be dosed twice daily (Q12) for a total of 10 doses administered over 5 days. Dosing of study drug in the fed state is encouraged.<br><br>In each dosing regimen, a single loading dose will be given as Dose 1 on Day 1. Following this, maintenance doses will be given Q12 (Doses 2–10) (Figure 2-1). In each dosing regimen, a single loading dose (Dose 1) will be followed 12 hours later by a twice daily (Q12h) maintenance dose regimen (Doses 2-10; Figure 1-1Preliminary Dosing Schema*<br><br>).	decide when these data are sufficient to support the initiation of potentially more efficacious dosing regimens (i.e., cohorts in the MAD). The proposed doses provide careful dose escalation in infants to doses which are projected to achieve an efficacious exposure (i.e., ≥EC_{99} throughout the dosing cycle).

Section	Original Text in Version 3.0	Revised Text in Version 4.0	Reason for Change
Synopsis<br>***Number of Sites and Location***	Approximately 50 sites in Europe, Asia Pacific, South Africa, and North/Latin America (specific countries to be determined) will participate.	Approximately ~~50~~70 sites in Europe (UK, France, Romania), Asia Pacific (Australia, New Zealand, Taiwan, Thailand, Japan), South Africa, and North/Latin America ~~(specific countries to be determined)~~ (Canada, United States, Panama, Colombia, Chile) will participate.	Additional sites and countries are being added to ensure the study meets its objectives within targeted timelines.
2.3<br>*Study Design, Part 2,*<br>*Sample Size*<br>*Clinical Data*<br>*Pharmokinetics,* ***9.4***<br>*Determination of Sample Size*<br>The addition of a Japan-specific cohort in the MAD part of the study	Study Design Part 2	Study Design Part 2<br>Up to two additional cohorts may be evaluated in Part 2 at the discretion of the Sponsor, upon approval by the IDMC, based on the emerging PK profile and safety profile.<br>In addition, up to 24 Japanese infants will also be enrolled in Part 2 (See Section 1.1). Initially, Japanese patients will receive a 4.1 mg/kg loading dose followed by a 1.37 mg/kg maintenance dose. This dosing regimen is expected to deliver approximately similar exposures as those anticipated in non-Japanese infants receiving the first MAD dosing regimen (i.e., 10 mg/kg LD/2 mg/kg MD). The IDMC will review emerging safety and PK data and dose escalate as appropriate.	Preliminary PK data in healthy Japanese adults suggest that there is a 1.5 to 2.3 fold increase in the ALS-008112 exposure (C_{max} and AUC) in Japanese adult healthy subjects compared with non-Japanese adult healthy subjects for a given ALS-008176 dose. Based on these data, a dosing regimen expected to deliver exposures in Japanese infants that are approximately similar to the exposures anticipated in non-Japanese infants receiving the first MAD dosing regimen (i.e., 10 mg/kg LD/2 mg/kg MD) will be evaluated i.e., single loading dose of 4.1 mg/kg loading dose (LD) followed by nine 1.37 mg/kg maintenance doses<br>The Japanese cohort will be dosed in parallel to dosing of non-Japanese subjects in the 10 mg/kg LD/2 mg/kg MD cohort.
**SYNOPSIS, Sample Size**<br>**Section 2.0 Study Design**	Sample Size<br>Part 1 (SAD): Approximately 96 subjects. Up to 48 more subjects may be enrolled into up to two additional cohorts.	Sample Size<br>Part 1 (SAD): ~~Approximately~~:Up to 72~~96~~ subjects will enroll in the ongoing planned cohorts (1.37, 4.1, and 12 mg/kg). Up to 72~~48~~	

Section	Original Text in Version 3.0	Revised Text in Version 4.0	Reason for Change
**Section 9.4 Determination of Sample Size**	Part 2 (MAD): Approximately 72 subjects (not previously randomized in Part 1). Up to 48 more subjects may be enrolled into up to two additional dose cohorts.	more subjects may be enrolled into up to ~~two~~three additional cohorts.<br>Part 2 (MAD): ~~Approximately 72~~Up to 48 subjects (not previously randomized in Part 1) will enroll in 2 MAD cohorts (N=≤24/cohort). Up to 24 additional Japanese subjects will be enrolled in a separate MAD cohort. Up to 48 more subjects may be enrolled into up to two additional dose cohorts.	
**Section 1.1,** Background	Clinical Data Safety:<br>One hundred one healthy adult volunteers were enrolled in the first-in-human single ascending dose (SAD), food effect, and multiple ascending dose (MAD) study (Study ALS-8176-501); 76 and 25 subjects received ALS-008176 and placebo, respectively. Single doses up to 750 mg and multiple doses up to 750 mg twice daily, 12 hours apart (Q12) on Day 1 followed by 500 mg Q12 from Day 2 to Day 5 (MAD Cohorts 1-4) or Day 2 to Day 14 (MAD Cohort 5) have been administered in the SAD and MAD, respectively. In this study, ALS-008176 was well tolerated, with no serious adverse events being reported and no adverse events leading to discontinuation of study drug. All reported adverse events (N=18) were mild (N=14) or moderate (N=4) in severity, with the following mild adverse events being reported: headache (8 events), hot flush (3 events), and toothache, sore throat, and diarrhea, each of which occurred as a single event. The 4 moderate events reported	*Clinical Data - Safety*<br>As of 21 August 2015, 150 healthy volunteers have received ALS-008176 as single doses up to 750 mg or multiple doses up to a 750 mg loading dose (LD) followed by a 500 mg maintenance dose (MD; dosed twice daily, 12 hours apart [Q12]) for a total duration of up to 14 days. Additionally, in this study 41 infants 1–12 months of age have received single doses of up to 12 mg/kg of ALS-008176 or placebo.<br>In all studies conducted to date, ALS-008176 has been well tolerated. Two serious adverse events (SAE) have been reported in the ongoing study in hospitalized infants. One event (phlebitis requiring IV antibiotics) occurred in this study five days after an infant received a single 1.37 mg/kg dose and was considered unrelated to study drug (ALS-008176 or Placebo). A second event (pneumonia with respiratory failure) occurred 2 days after an infant received a 12 mg/kg dose, and was considered unrelated to study drug. To date, no adverse events (AEs) in any study have led to study drug discontinuation.<br>All reported AEs in all studies in healthy volunteers have been mild or moderate in severity except 3 events, all of which were	Updated clinical safety, efficacy and PK data and enrollment status have been provided.

Section	Original Text in Version 3.0	Revised Text in Version 4.0	Reason for Change
	were paresthesia and headache in 2 subjects treated with ALS-008176 and abdominal pain and diarrhea in a placebo-treated subject. No important imbalances in any adverse events were observed in ALS-008176-treated vs. placebo-treated subjects. Additionally, no clinically significant laboratory, electrocardiogram (ECG), vital sign, or physical exam findings were noted at any dose evaluated.<br><br>An RSV challenge study (Study ALS-8176-502) evaluating three different 5 day ALS-008176 regimens vs. placebo was also conducted in 62 (44 ALS-008176: 18 Placebo) healthy volunteers. Preliminary data indicate that ALS-008176 rapidly and potently inhibited viral replication with an accompanying reduction in signs and symptoms of RSV among infected subjects (for a detailed discussion, see the Addendum to V4 of the Investigator's Brochure). Safety data indicate that there were no serious adverse events (SAEs), premature discontinuations of study drug, or clinically significant, treatment related adverse events in any study participants. Seventy-nine treatment-emergent AEs were reported; these were generally balanced for frequency and intensity across ALS-008176 and placebo recipients and all but two were mild (N=72) or moderate (N=5) in severity. The 2 severe (Grade 3) adverse events were asymptomatic laboratory	severe. Two of these severe events (increased alanine transaminase [ALT] and creatinine kinase) occurred in Study ALS-8176-502; both events occurred in subjects dosed with ALS-008176 and neither was considered related to study drug (See discussion in IB v5 Section 6.5.1.2.2). The third severe event (increased cholesterol) occurred in Study ALS-8176-511/ 64041575RSV1001 (Japanese ethnic bridging study) 13 days after receiving a single dose of ALS-008176/placebo (the study remains blinded) and was considered possibly related to study drug. The most commonly reported AEs (i.e., ≥3% incidence in adult subjects receiving multiple doses of ALS-008176) which have been reported more often in ALS-008176-treated vs. placebo-treated healthy volunteers are: epistaxis, headache, oropharyngeal pain, and platelet count decreased. None of these events is considered suggestive of an emerging safety signal, as discussed in the current IB (v5, Section 6.5.1.2.2).<br>To date, all AEs (N=41) in Study ALS-8176-503 except the pneumonia SAE, which was considered severe, have been mild or moderate in severity. A blinded summary table of all treatment emergent AEs reported as of 21 August 2015 can be found in Table 1-1. Unblinded safety data from this study are regularly reviewed by an Independent Data Monitoring Committee (IDMC), which has expressed no safety concerns based on a cumulative review of safety and PK data as recently as 31 July 2015.<br>Table 1 - Incidence and Severity of Treatment Emergent AEs in Study ALS-8176-503 (As of 18 August 2015)	

Section	Original Text in Version 3.0	Revised Text in Version 4.0	Reason for Change
	abnormalities: increased ALT (in a subject treated with ALS-008176 - 750 mg loading dose/500 mg maintenance dose) and increased creatine kinase (in a subject treated with ALS-008176 750 mg loading dose/150 mg maintenance dose, who had experienced strenuous exercise prior to the event). These 2 events were considered by the blinded Investigator to be unlikely and not related to study drug, respectively. The most commonly reported AEs (≥2 participants reporting an event in any one treatment group) are summarized in Table 5 of the Addendum to V4 of the Investigator's Brochure. No clinically relevant laboratory, electrocardiogram, vital sign, or physical exam findings were identified.<br><br>An open label, single dose study to evaluate the absolute bioavailability and mass balance of ALS-008176 (Study ALS-8176-504) was conducted in 12 healthy volunteers. Preliminary data indicate that no adverse events were reported in this study.<br><br>Clinical Data – Pharmacokinetics<br>In healthy adult subjects, ALS-008176 was not detected in plasma after dosing. Time to the maximum plasma concentration (T_{max}) of ALS-008112 was reached rapidly, within approximately 15-30 minutes, indicating	In all studies of ALS-008176 in both healthy volunteers and infants, no clinically significant laboratory, electrocardiogram (ECG), vital sign, or physical exam findings suggestive of a safety concern have been identified.<br><br>Clinical Data - Efficacy<br>The efficacy of ALS-008176 in naturally infected patient populations has not been defined; however, it has been assessed in healthy volunteers infected with RSV in a human challenge model (Study ALS-8176-502). In this study, in 62 healthy volunteers inoculated with RSV and dosed with ALS-008176 or placebo, preliminary data demonstrate that maintenance doses of 150 to 500 mg of ALS-008176, particularly following a 750-mg loading dose, resulted in rapid, substantial declines in RSV viral load with an accompanying comparable improvement in signs and symptoms of RSV infection compared to placebo-treated subjects. The effect of ALS-008176 on RSV viral load in the challenge model can be found in Figure 1-2. There was an accompanying decrease in RSV signs and symptoms that corresponded to these reductions in viral load (IB v5 Section 6.3).<br><br>Clinical Data - Pharmacokinetics<br>For a detailed discussion of the pharmacokinetics of ALS-008176 and its metabolites in healthy volunteers and infants, please refer to Section 6.2 of the current Investigator's Brochure (V5).<br>The following additional PK information has	

Section	Original Text in Version 3.0	Revised Text in Version 4.0	Reason for Change
	good oral absorption of ALS-008176 and effective conversion to ALS-008112. The only other major metabolite in plasma was ALS-008144, the inactive uridine metabolite of ALS-008112. While the terminal half-life of ALS-008112 was calculated to be 63 hours, ALS-008112 plasma concentrations decreased to very low levels within 6 hours post dose, indicating rapid and extensive distribution of ALS-008112. Following single and multiple doses, the AUC of ALS-008112 and ALS-008144 increased linearly but less than dose proportionally with increasing doses. The highest exposure of ALS-008112 obtained in adults was an average C_{max} of 4069 ng/mL (fasted state at 750 mg) and an average AUC_{0-24h} of 12135 ng·h/mL (fed state 750 mg Q12). No marked accumulation of either ALS-008112 or ALS-008144 was noted following multiple doses of ALS-008176. Administration of ALS-008176 following either a high fat, high calorie diet (after a single dose) or a normal diet (after multiple doses), lowered the plasma C_{max} of ALS-008112 by approximately 50% without affecting the AUC. Preliminary data from Study ALS-8176-504 indicate that ALS-008176 is ~64% bioavailable and primarily (~80%) eliminated through the kidneys.	become available since the IB was finalized:<br>• Japanese PK - Based on preliminary PK data from Study ALS-8176-511 /64041575RSV1001, a dose proportional increase in plasma exposure of ALS-008112 was noted in Japanese healthy adult volunteers receiving single doses of ALS-008176 suspension at 250 mg, 500 mg and 750 mg in fasted condition. There is a 1.5 to 2.3 fold increase in the ALS-008112 exposure (C_{max} and AUC) in Japanese adult subjects compared to non-Japanese healthy adult subjects at a given ALS-008176 dose. A similar trend of increase in ALS-008144 and ALS-008206 exposure was noted between the populations. However, metabolite ratios of ALS-008144 and ALS-008206 to ALS-008112 remained similar between the two populations. This difference in exposure appears to be due to better absorption of ALS-008176 in Japanese subjects.<br>• Infant PK - In Study ALS-8176-503, ALS-008176 is rapidly converted to ALS-008112. A population pharmacokinetic (popPK) model has been established to predict infant pharmacokinetics. Currently available preliminary infant PK data indicate that the observed blood exposures for the parent nucleoside, ALS-008112, are within those predicted by the popPK model for both the 4.1 mg/kg and 1.37 mg/kg dose levels () and are generally dose proportional. Additionally, for a given dose, no significant age-related differences in ALS-008112, ALS-008144 and ALS-008206 exposures have been observed.	

Section	Original Text in Version 3.0	Revised Text in Version 4.0	Reason for Change
	Based on the observed effects of ALS-008112 on clastogenicity, a maximum threshold of exposure for ALS-008112 in humans has been defined as an average AUC_{0-24h} of 20,000 ng•h/mL, a limit that is 2.7-fold lower than lowest no-observed-effect level (NOEL)/no-observed-adverse-effect level (NOAEL) observed in nonclinical toxicity studies.<br>Additional information regarding ALS-008176 is available in the ALS-008176 Investigator's Brochure.	Figure 1-2<br><br>Based on the observed effects of ALS-008112 on clastogenicity, a maximum threshold of exposure for ALS-008112 in humans has been defined as an average AUC_{0-24h} of 20,000 ng•h/mL, a limit that is 2.7-fold lower than lowest no-observed-effect level (NOEL)/no-observed-adverse-effect level (NOAEL) observed in nonclinical toxicity studies.<br><br>A study to determine absolute bioavailability of ALS-008112 and mass balance of ALS-008176 in healthy volunteers has been completed and detailed in Section 6.2 of the IB v5. ~~Additional information regarding ALS-008176 is available in the ALS-008176 Investigator's Brochure~~For results from CYP450 inhibition and induction studies and transporter profiling, please refer to Section 5.2.1 in the current IB (v5). Studies to evaluate the potential for ALS-008112 and ALS-008144 as substrates or inhibitors of BSEP transporter and the induction potential for ALS-008176, ALS-008112, and ALS-008144 on CYP1A2 and CYP2B6 are ongoing.<br><br>Enrollment Status<br>As of 21 August 2015, 1/8 subjects have enrolled at the current 12 mg/kg dose level in the 6-12 month old cohort. In the 2-6 month old and 1-2 month old cohorts, 6/8 and 2/8 subjects, respectively, have enrolled at the current 4.1 mg/kg dose level (**Error! Reference source not found.**).	
Table 1-2	**Parameters Used to Determine**	**Parameters Used to Determine Projected**	Clarification

Section	Original Text in Version 3.0	Revised Text in Version 4.0	Reason for Change
	**Projected Efficacious Doses in Infants**	**Efficacious Doses in Infants*** *Initial projections before availability of human challenge (ALS-8176-502) and infant (ALS-8176-503) data	
1.3.1 *Single Ascending Dose (SAD)*	The proposed dose escalation to 4.5, 7 and 10 mg/kg in subsequent cohorts allows for understanding of dose and age related changes in pharmacokinetics. The highest dose of 10 mg/kg is projected to have an average ALS-008112 C_{max} of 1877 ng/mL and an average ALS-008112 AUC_{0-24h} of 3545 ng•h/mL; exposures that are well within those that were obtained in adults. For ALS-008144, plasma exposures are also projected to be well within those obtained in adults.	The proposed dose escalation to ~~4.5, 7 and 10 mg/kg in subsequent cohorts~~ allows for understanding of dose and age related changes in pharmacokinetics. The highest dose of ~~10~~ 12 mg/kg is projected to have an average ALS-008112 C_{max} of ~~1877~~ 2232 ng/mL and an average ALS-008112 AUC_{0-24h} of ~~3545~~ 2403 ng•h/mL; exposures that are well within those that were obtained in adults. For ALS-008144, plasma exposures are also projected to be well within those obtained in adults.	Update
1.3.2 *Multiple Ascending Dose*	The 3 dose regimens span the projected therapeutic ranges. <br>For cohort 1, a single loading dose of 4.5 mg/kg followed by 1.5 mg/kg Q12 as maintenance doses is projected to provide average steady state C_{min} lung NTP concentrations that are between IC_{50} and IC_{90} antiviral activity. For cohorts 2 and 3, a single loading dose of 10 mg/kg is projected to achieve lung NTP levels within 12 hours that are needed for IC_{90} antiviral activity. The maintenance doses of either 4.5 or 7 mg/kg are projected to provide steady state C_{min} lung NTP levels to be $\geq 3 \times IC_{90}$. At all proposed doses the C_{max} and AUC of ALS-008112 (and ALS-008144) are projected to be well	The 3 planned dose regimens span the projected therapeutic ranges. <br>For cohort 1, a single loading dose of ~~4.5~~ 10 mg/kg followed by ~~1.5~~ 2 mg/kg Q12 as maintenance doses ~~is projected to provide average steady state C_{min} lung NTP concentrations that are between IC_{50} and IC_{90} antiviral activity. For cohorts 2 and 3, a single loading dose of 10 mg/kg~~ is projected to achieve lung NTP levels within ~~12~~ 2 hours that are needed for ~~IC_{90}~~ EC_{99} antiviral activity. ~~The maintenance doses of either 4.5 or 7 mg/kg are projected to provide steady state C_{min} lung NTP levels to be $\geq 3 \times EC_{90}$.~~ The maximum possible dose for cohort 2, 30 mg/kg LD followed by a 6 mg/kg MD is projected to achieve ≥ 3 x EC_{99} within 2 hours and maintain Cmin >3 x EC_{99} throughout the dosing cycle. Based on observed	

Section	Original Text in Version 3.0	Revised Text in Version 4.0	Reason for Change
	within what has been studied in adult healthy volunteers in study ALS-8176-501. Two optional cohorts may be studied to further define dose-response relationships.	differences in PK between Japanese and non-Japanese subjects, the initial Japanese infant regimen of 4.1 mg/kg LD followed by a 1.37 mg/kg MD is estimated to approximate the exposures of the 10 mg/kg LD followed by 2 mg/kg MD in (non-Japanese) MAD cohort 1.<br>The plasma ALS-008122 AUC_{0-24} for highest permissable dose in MAD cohort 2, 30 mg/kg LD/6 mg/kg MD, is projected to be 7,122 ng.h/ml on Day 1 and 2,810 ng.h/ml at steady state. ~~At all proposed doses the C_{max} and AUC of ALS-008112 (and ALS-008144) are projected to be~~ These exposures are well within what has been studied in adult healthy volunteers in study ALS-8176-501. Two optional cohorts may be studied to further define dose-response relationships.	
SYNOPSIS, 3.2.2, 9.3.2<br>*Secondary Endpoints*	• PK parameters of ALS-008112 and ALS-008144 (and other metabolites, as applicable) in blood following single dose administration: C_{max}, t_{max}, $t_{1/2}$, CL/F and Vd_{ss}/F (excluding metabolites), AUC_{0-12h}, AUC_{0-24h}, AUC_{0-inf} or AUC_{0-last}<br>• PK parameters of ALS-008112 and ALS-008144 (and other metabolites as applicable) in blood following repeat dose administration: C_{max}, C_{min}, t_{max}, $t_{1/2}$, CL/F and Vdss/F (excluding metabolites), AUC_{0-24h}, AUC_{0-tau}, AUC_{0-inf} or AUC_{0-last}	• PK parameters of ALS-008112 and ALS-008144 (and other metabolites, as applicable) in blood following single dose administration: C_{max}, t_{max}, $t_{1/2}$, ~~CL/F and Vd_{ss}/F (excluding metabolites),~~ AUC_{0-12h}, AUC_{0-24h}, AUC_{0-inf} or AUC_{0-last}<br>• PK parameters of ALS-008112 and ALS-008144 (and other metabolites as applicable) in blood following repeat dose administration: C_{max}, C_{min}, t_{max}, $t_{1/2}$, ~~CL/F and Vdss/F (excluding metabolites),~~ AUC_{0-24h}, AUC_{0-tau}, AUC_{0-inf} or AUC_{0-last}	CL/F, Vd and Vd_{ss}/F are not applicable for ALS-008112, ALS-008144, and other metabolites.
**4.1** *Study Population*	Part 1 (SAD): Approximately 96 and up to 144 subjects (n = 24 per cohort) will	Part 1 (SAD): ~~Approximately 96~~ Up to 72 subject will enroll in the ongoing planned	Consistency with updates in other sections

Section	Original Text in Version 3.0	Revised Text in Version 4.0	Reason for Change
	be enrolled.<br><br>Part 2 (MAD): Approximately 72 and up to 120 subjects (n = 24 per cohort) will be enrolled.	cohorts (1.37, 4.1, and 12 mg/kg) and up to 144 subjects (if an additional 3 cohorts are enrolled; n = 24 per cohort) will be enrolled.<br><br>Part 2 (MAD): ~~Approximately 72~~ Up to 48 subjects not previously randomized in Part 1 will enroll in the 2 MAD cohorts (N ≤24/cohort). Up to 24 additional Japanese subjects will be enrolled in a separate MAD cohort. Up to 48 more subjects may be enrolled into up to 2 additional dose cohorts. ~~and up to 120 subjects (n = 24 per cohort) will be enrolled.~~	
**4.2.3b** *Inclusion Criteria,* BINAX NOW RSV test or an RSV PCR or any other RSV assay may be used to determine eligibility	Has been diagnosed with RSV infection based on study-supplied BINAX NOW RSV test or an RSV PCR assay conducted at the clinical trial site. NOTE: A subject remains eligible if the BINAX NOW RSV result is negative but a RSV-specific PCR assay run locally is positive. (RSV-specific PCR run locally is not required.) Coinfection with other respiratory viruses or bacterial coinfection in addition to RSV infection is permissible.	Has been diagnosed with RSV infection based on study-supplied BINAX NOW RSV test or an RSV PCR or any other RSV assay conducted at the clinical trial site. NOTE: A subject remains eligible if ~~the BINAX NOW~~ any RSV result is ~~negative but a RSV-specific PCR assay locally is~~ positive. (RSV-specific PCR run locally is strongly encouraged, but not required.) Coinfection with other respiratory viruses or bacterial coinfection in addition to RSV infection is permissible.	As the study has been conducted, it has become clear that false negatives are occurring commonly with the BINAX NOW test. In addition, many sites already use local RSV testing as a part of their standard of care. By permitting a positive BINAX NOW or RSV PCR or any other RSV assay to determine RSV positivity, the risk of BINAX NOW related false negatives is decreased and greater flexibility is allowed to accommodate local practice differences in this global study.
**4.2.5** *Inclusion Criteria,* Creatinine Clearance (Schwartz equation)	Creatinine clearance is within the normal range for the subject's age (Schwartz equation)	Creatinine clearance is ~~within the normal range~~ not below the lower limit of normal for the subject's age (Schwartz equation calculation preferred, however alternative equations may be utilized to determine eligibility if deemed acceptable by the Investigator and Medical Monitor).	Some sites report that they do not have a normal range for creatinine clearance as defined by the Schwartz equation. In these instances, an alternative determination of creatinine clearance is permitted to ensure patients can be properly assessed for their underlying renal function.

Section	Original Text in Version 3.0	Revised Text in Version 4.0	Reason for Change
**4.3.9b** *Exclusion Criteria and*<br>**5.8** *Prohibited Medications,*<br>Corticosteroids	b.Use of systemic medications (either chronically or within the 21 days prior to randomization) which are known to modulate the host immune response and/or increase viral shedding such as corticosteroids or other immunomodulatory therapies. Systemic corticosteroids administered before randomization will be acceptable if:<br>• The dose is ≤ 2 mg/kg and ≤20 mg/day of prednisolone or equivalent, and<br>• The corticosteroids are given as a treatment for the subject's RSV symptoms, and<br>• The corticosteroids have not been administered for more than a total of 4 days.<br>• NOTE: Eligible subjects should not receive study medication for at least 12 hours after the last dose of any systemically administered corticosteroids, and no planned further administration of corticosteroids is permitted during the study.	b. Use of systemic medications (either chronically or within the 21 days prior to randomization) which are known to modulate the host immune response and/or increase viral shedding such as corticosteroids or other immunomodulatory therapies. The only exception is ~~S~~systemic corticosteroids ~~administered before randomization~~ will be acceptable if they are not taken chronically for a non-RSV-related indication.<br>~~• The dose is ≤ 2 mg/kg and ≤20 mg/day of prednisolone or equivalent, and~~<br>~~• The corticosteroids are given as a treatment for the subject's RSV symptoms, and~~<br>~~• The corticosteroids have not been administered for more than a total of 4 days at the time of randomization.~~<br>• ~~NOTE: Eligible subjects should not receive study medication for at least 12 hours after the last dose of any systematically administered corticosteroids, and no planned further administration of corticosteroids is permitted during the study~~.	The use of corticosteroids is considered ineffective and not recommended by major academic medical societies. Nevertheless in some countries corticosteroids are commonly used. There is no reason to think that brief use of corticosteroids prior to and after randomization will undermine the ability to interpret the study's primary objectives (safety and PK). As a result, to accommodate the clinical reality of the common use of systemic corticosteroid in infants hospitalized with RSV infection, the non-chronic use of systemic and inhaled corticosteroids will be permitted.
**5.8** *Prohibited Medications,*<br>Corticosteroids	Prohibited medications during the conduct of this study include:<br>• Prescription medications which are known to modulate the host immune response and/or increase viral shedding such as systemic corticosteroids or other immunomodulatory therapies	Prohibited medications during the conduct of this study include:<br>Prescription medications which are known to modulate the host immune response and/or increase viral shedding such as ~~systemic corticosteroids or other~~ immunomodulatory therapies. (Systemic and inhaled corticosteroids	The use of corticosteroids is considered ineffective and not recommended by major academic medical societies. Nevertheless in some countries corticosteroids are commonly used. There is no reason to think that brief use of corticosteroids prior to and after

Section	Original Text in Version 3.0	Revised Text in Version 4.0	Reason for Change
	(inhaled corticosteroids are permitted, however)	are permitted, however).	randomization will undermine the ability to interpret the study's primary objectives (safety and PK). As a result, to accommodate the clinical reality of the common use of systemic corticosteroid in infants hospitalized with RSV infection, the non-chronic use of systemic and inhaled corticosteroids will be permitted.
**4.3.** *Exclusion Criteria*	9d) Prescription medications which are known to be a strong inducer or inhibitor of CYP450 enzymes, within 21 days prior to randomization<br><br>10) Infants who are breastfeeding and their mother is taking any of the exclusionary medications described in exclusion criterion 9.	9d) Prescription medications which are known to be a strong inducer or inhibitor of CYP450 enzymes, within 21 days prior to randomization (See Prohibited Medication list in the Study Manual)<br><br>10) Infants who are breastfeeding and their mother is taking any of the exclusionary medications described in exclusion criterion ~~10~~9.<br><br>12) Infants with known fructose intolerance (due to sorbitol in study medication)<br><br>13) Ethnically Japanese infants will not be enrolled outside of Japan	Clarifications
**4.4** *Subject Screening and Enrollment*	All screening procedures will generally need to be completed within one 48 hour period. Procedures that are standard of care and performed within 48 hours prior to Screening may be used in determining protocol eligibility. Exception: If a different RSV diagnostic test was performed prior to obtaining informed consent for this study, the BINAX NOW RSV test must also be performed after obtaining informed	All screening procedures will generally need to be completed within one ~~48~~ 96 hour period. Procedures that are standard of care and performed within ~~48~~ 96 hours prior to Screening may be used in determining protocol eligibility. ~~Exception: If a different RSV diagnostic test was performed prior to obtaining informed consent for this study, the BINAX NOW RSV test must also be performed after obtaining informed consent.~~	Screening window expanded

Section	Original Text in Version 3.0	Revised Text in Version 4.0	Reason for Change
	consent.		
**Table 5-1 Part 1 SAD Dosing Regimen**	18 subjects to receive single oral X mg/kg dose of ALS-008176 with 6 subjects receiving placebo	Up to 24 subjects* (3 ALS-008176:1 placebo) - 18 subjects to receive single oral X mg/kg dose of ALS-008176 with 6 subjects receiving placebo<br>*Assuming the IDMC does not initiate Part 2 before completion of the cohort.	Table updated for consistency with other sections
**Table 5-2 Part 2 MAD Dosing Regimen**	18 subjects will be assigned to receive oral ALS-008176 and 6 subjects will be assigned to receive placebo. A loading dose of X mg/kg will be administered for Dose 1 on Day 1 followed by X mg/kg given Q12 for 5 consecutive days (Doses 2-10).	Japanese Cohort: Up to 24 subjects (3 ALS-008176:1 placebo)- Subjects will initially receive a loading dose of 4.1 mg/kg for Dose 1 on Day 1 followed by 1.37 mg/kg given Q12 for 5 consecutive days (Doses 2-10). The IDMC may dose escalate subsequent patients in the cohort to a dose regimen that is no more than 3-fold higher.	Table updated for consistency with other sections
**5.1.1***Cohort Progression Guidelines*	The Sponsor, upon approval by the IDMC, may advance an older age group(s) to the next dosing cohort before enrollment in younger age group(s) within that dose cohort is complete.<br>Part 2 will not commence before all safety data through study completion for all subjects in cohorts 1, 2 and 3 of Part 1, and PK data from the first 24 hours after dosing for at least 75% of the current cohort's subjects (or a completed stratum within this cohort) have been evaluated and are deemed acceptable by the IDMC. MAD Cohort 2 will commence no earlier than after completion of SAD Cohort 4 and MAD Cohort 1.	• Part 2 - Initation of the MAD will occur when emerging PK and safety data from Part 1 have been evaluated and are deemed by the IDMC to be acceptable and sufficient to initiate multiple dosing.<br>• Part 2 Dose Escalations – any MAD cohort will commence once authorized by the IDMC based on emerging safety and PK data. all available safety data through study completion for all subjects, as well as available PK data for the first 24 hours after dosing for at least 75% of subjects in the most recent cohort will be reviewed.	Clinical trials in pediatrics have an obligation to advance to potentially efficacious dosing regimens as soon as the safety data suggest the risk profile of these regimens appears acceptable. The Sponsor considers the IDMC best suited to make this decision because it has the most comprehensive understanding of the unblinded safety profile of ALS-008176. The IDMC can best decide when these data are sufficient to support the initiation of potentially more efficacious dosing regimens (i.e., cohorts in the MAD). The proposed doses provide careful dose escalation in infants to doses which are projected to achieve an

Section	Original Text in Version 3.0	Revised Text in Version 4.0	Reason for Change
			efficacious exposure (i.e., ≥EC_{99} throughout the dosing cycle).
**5.1.1** *Cohort Progression Guidelines*		In Part 1, ~~T~~the Sponsor, upon approval by the IDMC, may advance an older age group(s) to the next dosing cohort before enrollment in younger age group(s) within that dose cohort is complete.<br>~~Part 2 will not commence before all safety data through study completion for all subjects in cohorts 1, 2 and 3 of Part 1, and PK data from the first 24 hours after dosing for at least 75% of the current cohort's subjects (or a completed stratum within this cohort) have been evaluated and are deemed acceptable by the IDMC. MAD Cohort 2 will commence no earlier than after completion of SAD Cohort 4 and MAD Cohort 1.~~	
**Table 6-1** *Schedule of Events SAD Phase (Part 1)*	Screen (-1) to 1	Screen (-3~~1~~) to 1	Screening window expanded
**Table 6-2** *Schedule of Events MAD Phase (Part 2)*	Screen (-1) to 1	Screen (-3~~1~~) to 1<br>Follow Up Visit Day 28 (±7 days)<br>8. Day 28 visit may be conducted as a home visit by a physician. Complete physical exam and weight are required. All other safety assessments need only be collected if deemed clinically necessary based on an assessment of the subject's safety (e.g., adverse events) or as follow up of an earlier adverse event (e.g., thrombocytosis). A nasal aspirate specimen is encouraged but not required.<br>9. Optional: If a blood specimen is collected for safety assessments during the follow up visit, enough blood should be collected to assess	Screening window expanded<br>The pediatric committee (PDCO) within the European Medicines Agency (EMA) and the Japanese health authority have requested that a longer follow up (i.e. 28 days) be conducted in the multiple dose part of this study. As a result, a MAD Day 28 visit is being added to further assess safety.

Section	Original Text in Version 3.0	Revised Text in Version 4.0	Reason for Change
		blood biomarkers	
**6.1.2** *Serum Chemistries and Complete Blood Count with Differential*	Required safety laboratory variables are defined in Section 8.2.2, *Clinical Laboratory Evaluations*, and will be assessed by the site's local laboratory.<br><br>Blood for serum chemistries, CBC with differential sampling time points: All subjects will have a pre-dose sample obtained to establish a safety baseline. (If all safety labs were obtained within 48 hours of randomization as a part of routine clinical care, the pre-dose safety labs do not need to be collected.)<br><br>If blood collection volume is limited, the order of priority is the following: safety>PK>biomarkers (Screening, Day 5 Safety Visit (MAD only), & Completion Visit), PK>safety>biomarkers (Day 1).	Required safety laboratory variables are defined in Section 8.2.38.2.2, *Clinical Laboratory ~~Evaluations~~ Measurements*, and will be assessed by the site's local laboratory.<br><br>Blood for serum chemistries, CBC with differential sampling time points: All subjects will have a pre-dose sample obtained to establish a safety baseline. (If all safety labs were obtained within 4896 hours of randomization as a part of routine clinical care, the ~~pre-dose~~screening safety labs do not need to be collected.)<br><br>If blood collection volume is limited, the order of priority is the following: safety>PK>biomarkers (Screening, Day 5 Safety Visit and Follow Up Visit (MAD only), & Completion Visit), PK>safety>biomarkers (Day 1).	Typographical corrections and consistency with Tables 6-1 and 6-2
**6.1.3** *Pharmacokinetic Blood Sampling*	Each subject will have a maximum of 4 (for SAD) or 5 (for MAD) study-mandated blood samples collected over the course of the study in order to determine the safety and PK of ALS-008176.<br><br>• Subjects participating in Part 2 (MAD) will have one additional PK lab sample collected on Day 5.	Each subject will have a maximum of 4 (for SAD) or 56 (for MAD) study-mandated blood samples collected over the course of the study in order to determine the safety and PK of ALS-008176.<br><br>• Subjects participating in Part 2 (MAD) will have one additional PK lab sample collected on Day 5 and one blood sample, if necessary, for safety labs and biomarkers collected on Day 28.	Updated according to new MAD Day 28 visit.

Section	Original Text in Version 3.0	Revised Text in Version 4.0	Reason for Change
**6.1.5** *RSV Evaluations*	RSV Diagnosis<br><br>RSV diagnosis will be confirmed prior to randomization by rapid diagnostic assay using the Binax NOW RSV test. The Alere Binax NOW RSV Card is a rapid immunochromatographic assay for the qualitative detection of RSV fusion protein antigen in nasal wash and nasopharyngeal swab specimens. If a different RSV diagnostic test (other than PCR) was performed prior to obtaining informed consent for this study, the Binax NOW RSV test must also be performed after obtaining consent.	RSV Diagnosis<br><br>RSV diagnosis will be confirmed prior to randomization by rapid diagnostic assay using the study supplied Binax NOW RSV or any other RSV diagnostic test. The Alere Binax NOW RSV Card is a rapid immunochromatographic assay for the qualitative detection of RSV fusion protein antigen in nasal wash and nasopharyngeal swab specimens. ~~If a different RSV diagnostic test (other than PCR) was performed prior to obtaining informed consent for this study, the Binax NOW RSV test must also be performed after obtaining consent.~~	Consistency with other sections.
	Sponsor will provide kits for all RSV-related sample collections.	Sponsor will provide kits for all RSV-related sample collections.	Consistency with other sections
	NOTE: In the event that the Binax NOW RSV test result is negative, a subject may still be eligible to enroll if an optional RSV-specific PCR assay conducted by local laboratory is positive for RSV infection and the subject meets all other inclusion/exclusion criteria	~~NOTE: In the event that the Binax NOW RSV test result is negative, a subject may still be eligible to enroll if an optional RSV-specific PCR assay conducted by local laboratory is positive for RSV infection and the subject meets all other inclusion/exclusion criteria.~~	
	Qualitative PCR<br><br>Qualitative PCR will be performed by a central lab, using pre-dose nasal aspirate sample, to confirm RSV diagnosis, however, study eligibility is determined by the results of the BinaxNOW RSV rapid test or a RSV PCR assay conducted at the clinical trial site.	Qualitative PCR<br><br>Qualitative PCR will be performed by a central lab, using pre-dose nasal aspirate sample, to confirm RSV diagnosis, however, study eligibility is determined by the results of the local ~~BinaxNOW~~ RSV ~~rapid~~ test ~~or a RSV PCR assay conducted at the clinical trial site~~.	Consistency with other sections.
**7.1.2** *Adverse Event,*<br><br>**7.2.2** *Documenting &*	An AE can therefore be any new, or worsening of an existing, unfavorable and unintended sign, symptom,	An AE can therefore be any new, or worsening of an existing, unfavorable and unintended sign, symptom, laboratory finding outside of normal	The AE definition was updated to more closely match the definition of AE as defined in ICH Guidance

Section	Original Text in Version 3.0	Revised Text in Version 4.0	Reason for Change
Reporting Adverse Events<br>AE definition	laboratory finding outside of normal range with associated clinical symptoms or suspected latent clinical symptoms in the opinion of the investigator, physical examination finding, or disease temporally associated with the use of the study drug, whether or not the event is considered related to the study drug.	range ~~with associated clinical symptoms or suspected latent clinical symptoms in the opinion of the investigator,~~ , physical examination finding, or disease temporally associated with the use of the study drug, whether or not the event is considered related to the study drug.	E2A - Clinical Safety Data Management: Definitions and Standards for Expedited Reporting
Section 7.2.2	An AE includes the following:<br>Laboratory abnormalities outside of normal limits and requiring therapeutic intervention.	An AE includes the following:<br>New or worsening ~~L~~laboratory abnormalities ~~outside of normal limits and requiring therapeutic intervention.~~	The AE definition was updated to more closely match the definition of AE as defined in ICH Guidance E2A - Clinical Safety Data Management: Definitions and Standards for Expedited Reporting
**7.3** *Follow up of Adverse Events and Serious Adverse Events*	Follow all AEs (serious and nonserious) until resolution or otherwise explained, the subject dies, the event stabilizes and is not expected to further resolve, or when alternative therapy is instituted, whichever occurs first. If alternative therapy is instituted, it should be documented. Alios BioPharma may request that the investigator perform or arrange for supplemental measurements or evaluations to further clarify the nature of the event.	Follow all AEs (serious and nonserious) until resolution or otherwise explained, the subject dies, the event stabilizes and is not expected to further resolve, or when alternative therapy is instituted, whichever occurs first. AEs that are non-serious and stable do not need to be followed after the last study visit - they should instead be marked as continuing as of the final study visit. ~~If alternative therapy is instituted, it should be documented.~~ Alios BioPharma may request that the investigator perform or arrange for supplemental measurements or evaluations to further clarify the nature of the event.	With the exception of serious adverse events, it is not practical to follow all adverse events after completion of the last study visit. Non-serious AEs ongoing at the time of the last visit will simply be recorded as ongoing in the case report forms.
**8.2** *Safety Variables/Measurements*	In this study, an estimated total of up to 5.65 or 13.60 mL of blood will be drawn in each SAD or MAD study subject, respectively, over the course of the study. This blood volume represents 1.66 or 4mL/kg for a 3.4 kg infant (~10th percentile for a 1 month old) and is within the generally accepted range for	In this study, an estimated total of up to 5.65 or ~~13.60~~16.85 mL of blood will be drawn in each SAD or MAD study subject, respectively, over the course of the study. This blood volume represents 1.66 or 4.96 mL/kg for a 3.4 kg infant (~10th percentile for a 1 month old) and is within the generally accepted range for blood draws for pediatric clinical research studies (Howie 2011).	Blood volumes updated to reflect new MAD Day 28 visit

Section	Original Text in Version 3.0	Revised Text in Version 4.0	Reason for Change
	blood draws for pediatric clinical research studies (Howie 2011). Actual blood volumes may vary at each institution.<br>If the MAD blood volume exceeds local blood volume limits, do not collect biomarker samples. Then total MAD volume 7.60 mL represents 2.23 mL/kg for a 3.4 kg child.	Actual blood volumes may vary at each institution.<br>If the MAD blood volume exceeds local blood volume limits, do not collect biomarker samples. Then total MAD volume ~~7.60~~ 9.35 mL represents ~~2.23~~2.75 mL/kg for a 3.4 kg child.	
**8.2.3** *Clinical Laboratory Measurements*		Sites should attempt to collect all safety labs listed in Table 8-1 as defined in this protocol. In some instances, individual laboratory assessments in Table 8-1 are not routinely run at a local site. If it is not practical to obtain the results for a particular assessment due to local considerations the Sponsor Medical Monitor may on a case by case basis permit individual sites to not collect that assessment.	Some sites have reported an inability to evaluate certain laboratory parameters required by the protocol, as defined in Table 8-1. While the intention is for all subjects to have all safety laboratory data, in some instances it is not feasible to collect a particular parameter (e.g., mean platelet volume) due to local considerations at the site. The Sponsor will review these on a case by case basis and determine if not collecting a particular assessment is permissible in certain circumstances.
**8.4** *Pharmacodynamic Measurements*	Qualitative measures (i.e., PCR assay) will confirm the rapid Binax NOW RSV results and determine whether coinfection with other detectable virus(es) has occurred.	Qualitative measures (i.e., PCR assay) will confirm the ~~rapid Binax NOW~~local RSV results and determine whether coinfection with other detectable virus(es) has occurred.	Consistency with other sections
**9.4** *Determination of Sample Size*	Based on clinical experience, these sample sizes are expected to be sufficient to enable determination of PK parameters in each of the pediatric age strata.	Based on clinical experience, these sample sizes are expected to be sufficient to enable an assessment of safety and tolerability at a given dose level and determination of PK parameters in each of the pediatric age strata.	Clarification

Section	Original Text in Version 3.0	Revised Text in Version 4.0	Reason for Change
**9.5** Randomization	At no time will dose escalation occur for a younger age stratum before dosing in an older age stratum has been initiated for that dose.	At no time will dose escalation occur for a younger age stratum before dosing in an older age stratum has been initiated for that dose, unless the IDMC determined that the safety profile supported such an approach.	Consistency with Sections 2.2, 5.1.1.
**9.9.1** *Safety Analyses - Adverse Events*		An adverse event is considered associated with the use of the study drug if the attribution is related or possibly related by the definitions listed in Section 7.2.3, *Assigning Attribution of Adverse Events*.	Clarification
**Appendix A**<br>Investigator Signature Page	Version: 3.0, 17 December 2014	Version: 4 ~~3~~.0, ~~17 December 2014~~ 24 August 2015	To reflect revised version
**Appendix B**<br>**Estimated Blood Volumes**<br>**Table 12-3 and 12-4**		Study Total: 16.85~~13.60~~ mL	Tables updated to reflect addition of MAD Day 28 visit.

Appendix G. Summary of Protocol Changes from Version 4.0 dated 24 August 2015 to Version 5.0

Section	Original Text in Version 4.0	Revised Text in Version 5.0	Reason for Change
Cover Page, Appendix A IND Number (cover page and page 11)	IND Number: Pending: Pre-IND Number 122,969	IND Number: ~~Pending: Pre-IND Number~~ 122,969	To reflect revised version and IND Number
Date	Date: 24 August 2015	~~24 August 2015~~ 22 January 2016	
Version	Version 4.0	Version 5~~4~~.0	
**Figure 1-1, Figure 2-1** *Preliminary Dosing Schema*		Figure updated	Figure updated to reflect SAD 25 mg/kg cohort, MAD Japanese 4.1/1.37 mg/kg cohort and reference to neonate cohort
**1.1 Background Information – Clinical Data – Safety**	As of 21 August 2015, 150 healthy volunteers have received ALS-008176… Additionally, in this study 41 infants 1-12 months of age have received single doses of up to 12 mg/kg of ALS-008176 or placebo… Two serious adverse events (SAE) have been reported in the ongoing study in hospitalized infants. To date, all AEs (N=41 in Study ALS-8176-503 except the pneumonia SAE, which was considered severe, have been mild or	As of ~~21 August 2015~~20 January 2016, ~~150~~165 healthy volunteers have received ALS-008176… Additionally, in this study ~~41~~ 79 infants 1-12 months of age have received single doses (N=67) of up to ~~12~~25 mg/kg or multiple doses (N=12) consisting of up to a 10 mg/kg loading dose followed by up to 2 mg/kg maintenance doses of ALS-008176 or placebo… ~~Two~~ Three serious adverse events (SAE) have been reported in the ongoing study in hospitalized infants…The third SAE (sinus tachycardia during a renal ultrasound procedure that required observation in the intensive care unit) occurred 24 hours after receiving a 25 mg/kg dose of study drug and was considered unrelated to study drug... To date, all AEs (N=~~41~~73: SAD N=57; MAD N=16) in Study ALS-8176-503 except the pneumonia SAE,	Updated safety information as of 20 January 2016

Section	Original Text in Version 4.0	Revised Text in Version 5.0	Reason for Change
	moderate in severity. A blinded summary table of all treatment emergent AEs reported as of 21 August 2015 can be found in Table 1-1.<br><br>Unblinded safety data from study study are regularly reviewed by an Independent Data Monitoring Committee (IDMC), which has expressed no safety concerns based on a cumulative review of safety and PK data as recently as 31 July 2015.	which was considered severe, have been mild (N=58) or moderate (N=14) in severity. A blinded summary table of all treatment emergent AEs reported as of ~~21 August 2015~~ 20 January 2016 can be found in Table 1-1 (SAD) and Table 1-2 (MAD). Unblinded safety data from study study are regularly reviewed by an Independent Data Monitoring Committee (IDMC), which has expressed no safety concerns based on a cumulative review of safety and PK data as recently as ~~31 July~~ 17 December 2015.<br><br>**Table 1-1** *Incidence and Severity of Treatment Emergent AEs in SAD portion of Study ALS-8176-503*<br><br>**New Table 1-2** *Incidence and Severity of Treatment Emergent AEs in MAD portion of Study ALS-8176-503*	
**1.1 Background Information – Clinical Data – Pharmacokinetics**	Japanese PK –<br>There is a 1.5 to 2.3 fold increase in the ALS-008112 exposure (Cmax and AUC) in Japanese adult subjects compared to non-Japanese healthy adult subjects at a given ALS-008176 dose.<br><br>Infant PK –<br>A population pharmacokinetic (popPK) model has been established to predict infant pharmacokinetics. Currently available preliminary infant PK data indicate that the observed blood exposures for the parent nucleoside, ALS-008112, are within those	Japanese PK –<br>There is a 1.5 to 2.3 fold increase in the ALS-008112 exposure (C_{max} and AUC, respectively) in Japanese adult subjects compared to non-Japanese healthy adult subjects at a given ALS-008176 dose.<br>Infant PK –<br>A population pharmacokinetic (popPK) model has been established to ~~predict~~ characterize infant pharmacokinetics. Currently available preliminary infant PK data indicate that the observed blood exposures for the parent nucleoside, ALS-008112, are within those predicted by the popPK model for ~~both the 4.1 and~~ single doses at the 1.37, 4.1 and 12 mg/kg dose levels (Figure 1-2) and are generally	Updated and clarifying PK information

Section	Original Text in Version 4.0	Revised Text in Version 5.0	Reason for Change
	predicted by the popPK model for both the 4.1 and 1.37 mg/kg dose levels (Figure 1-2) are generally dose proportional.<br><br>Figure 1-2. ALS-008112 Exposures in Infants 1-12 months of age receiving 1.37 or 4.1 mg/kg doses in Study ALS-8176-503<br><br>Based on the observed effects of ALS-008112 on clastogenicity, a maximum threshold of exposure for ALS-008112 in	dose proportional.<br>Preliminary PK data after multiple doses are also available for the 4.1 mg/kg LD followed by 1.35 mg/kg MD (4.1/1.37) regimen in Japanese subjects (N=6) as well as a 10/2 regimen in non-Japanese subjects (N=2). These data suggest:<br><br>o Multiple dose PK is similar to that projected based on the popPK model for ALS-008112<br><br>o There are no apparent ethnic differences in PK between Japanese and non-Japanese subjects in the patients evaluated to date<br><br>o No evidence of clinically important accumulation is present for either ALS-008112 or ALS-008144; some accumulation of ALS_008144 with multiple dosing may be observed, but requires confirmation in a larger number of patients<br><br>o No age related PK differences have been observed<br><br>Figure 1-2. ALS-008112 Exposures in Infants 1-12 months of age receiving 1.37, ~~or~~ 4.1, or 12 mg/kg doses in Study ALS-8176-503<br><br>Based on the observed effects of ALS-008112 on clastogenicity, a maximum threshold of plasma exposure for ALS-008112 in humans has been defined as…	

Section	Original Text in Version 4.0	Revised Text in Version 5.0	Reason for Change
	humans has been defined as…		
**1.1 Background Information – Enrollment Status, Figure 1.3**	As of 21 August 2015, 1/8 subjects have enrolled at the current 12 mg/kg dose level in the 6-12 month old cohort. In the 2-6 month old and 1-2 month old cohorts, 6/8 and 2/8 subjects, respectively, have enrolled at the current 4.1 mg/kg dose level (Figure 1-3).	See Figure 1-3 for a summary of enrollment as of 20 January 2016.<br><br>As of 21 August 201520 January 2016, 10/8 and 1/8 subjects have enrolled at the current 12 mg/kg dose level in the 6-12 month old and 2-6 month old cohorts, respectively, at the current 25 mg/kg dose level in the SAD portion of the study. In the 2-6 month old and 1-2 month old cohorts, 6/8 and 2/8 0/8 subjects, respectively, have enrolled at the current 4.1 12 mg/kg dose level (Figure 1-3).<br>In the MAD portion of the study, 6 Japanese subjects enrolled into the 4.1/1.37 dosing regimen before the IDMC instructed that Japanese subjects must enroll at the same dosing regimen as the rest of the world. At the 10/2 dosing level, 6 subjects have been enrolled.<br><br>Updated Figure 1.3. Study ALS-8176-503 Enrollment Status as of 21 Aug 201520 January 2016.	Updated enrollment status
**1.2 RATIONALE FOR THE STUDY**	This study is being performed to determine preliminary safety, tolerability, pharmacokinetic (PK), and pharmacodynamics (PD) data after single and multiple doses of ALS-008176 given to infants ≥ 1.0 to ≤12.0 months of age who are hospitalized with RSV infection.	This study is being performed to determine preliminary safety, tolerability, pharmacokinetic (PK), and pharmacodynamics (PD) data after single and multiple doses of ALS-008176 given to infants ≥1.0 to ≤12.0 months of age and neonates who are hospitalized with RSV infection.	Neonates are included in the development plan of ALS-008176. Based on the acceptable safety profile and lack of PK differences in 1-12 month old subjects enrolled to date, it is felt that a neonatal cohort can now be safely evaluated. A MAD dosing regimen at the 10/2 mg/kg dose level was chosen because this dose has been well tolerated in patients enrolled to date and it is projected to deliver

Section	Original Text in Version 4.0	Revised Text in Version 5.0	Reason for Change
			meaningful antiviral activity throughout the 5 day dosing duration. The actual dosing regimen for this cohort must be confirmed by the IDMC based on emerging data.
**1.3 RATIONALE FOR DOSE SELECTION**	…the pediatric PK model was coupled with semi-mechanistic pediatric lung model…The efficacious dose projections were used to justify the starting dose such that subtherapeutic concentrations are avoided in infants…Due to the inherent limitations regarding intensive PK sampling in infants, a population PK approach with optimal PK sampling calculations will be utilized.<br><br>The proposed starting dose in infants takes into consideration the following:<br>• Reversibe and monitorable nonclinical toxicology profile of ALS-008112 and margins from the nonclinical safety studies, specifically to limit the exposure of ALS-008112 AUC_{0-24} to <20,000 ng·h/mL.	…the pediatric PK model was coupled with a semi-mechanistic pediatric lung model… The efficacious dose projections were used to justify the starting dose such that subtherapeutic concentrations are avoided in infants and neonates… Due to the inherent limitations regarding intensive PK sampling in infants and neonates, a population PK approach with optimal PK sampling calculations will be utilized.<br>The proposed starting dose in infants takes into consideration the following:<br>Reversibe and monitorable nonclinical toxicology profile of ALS-008112 and margins from the nonclinical safety studies, specifically to limit the plasma exposure of ALS-008112 AUC_{0-24} to <20,000 ng·h/mL.	See 1.2<br><br>Clarification
**1.3.1 Single Ascending Dose (SAD)**	Based on a robust pediatric PK model and an estimation of human efficacious doses, a starting dose of 1.5 mg/kg ALS-008176 is proposed. At this dose in infants, the projected plasma exposure in a fasted state is predicted to be an average ALS-008112 C_{max} of 284 ng/mL and an average ALS-008112 AUC_{0-24h} of 295 ng·h/mL. The C_{max} and AUC of ALS-008112 are therefore projected to be at least 14- and 38-fold lower, respectively, than the exposures	Based on ~~a robust~~ pediatric PK modelling and an estimation of human efficacious doses, a starting dose of 1.5 mg/kg ALS-008176 ~~is proposed~~was selected for this study. ~~At t~~This dose in infants~~, the projected plasma exposure in a fasted state is predicted to be an average ALS-008112~~ ~~C_{max}~~ ~~of 284 ng/mL and an average ALS-008112~~ ~~AUC_{0-24h}~~ ~~of 295 ng·h/mL. The~~ ~~C_{max}~~ ~~and AUC of ALS-008112 are therefore projected to be at least 14- and 38-fold lower, respectively, than the exposures obtained at the highest doses tested in adults,~~ was projected to	Updated PK information

Section	Original Text in Version 4.0	Revised Text in Version 5.0	Reason for Change
	obtained at the highest doses tested in adults, 750 mg, a dose that was well tolerated with no significant adverse events in adults…<br>…The highest dose of 12 mg/kg is projected to have an average ALS-008112 C_{max} of 2232-4464 ng/mL and an average ALS-008112 AUC_{0-24h} of 2403 ng·h/mL; exposures that are well within those that were obtained in adults.	maintain ALS-008112 exposures significantly below exposures obtained with a 750 mg~~, a~~ dose in adults that was well tolerated with no significant adverse events ~~in adults~~…<br>…Based on population PK modeling, ~~T~~the highest dose of ~~12~~25 mg/kg is projected to have ~~an average ALS-008112 C_{max} of 2232-4464 ng/mL and an average~~ a median plasmaALS-008112 AUC_{0-24h} of ~~2403~~approximately 9001 - 10038 ng·h/mL; exposures that are similar to ~~well within~~ those which have been studied ~~that were obtained~~ in adults.	
**1.3.2 Multiple Ascending Dose (MAD)**	A loading dose is required to rapidly achieve these NTP levels followed by maintenance doses (Q12) which will maintain the constant levels of NTP that are required to inhibit viral replication…<br>…For cohort 1, a single loading dose of 10 mg/kg followed by 2 mg/kg Q12 as maintenance doses is projected to achieve lung NTP levels within 2 hours that are needed for EC_{99} antiviral activity.<br>…Based on observed differences in PK between Japanese and non-Japanese subjects, the initial Japanese infant regimen of 4.1 mg/kg LD followed by a 1.37 mg/kg MD is estimated to approximate the exposures of the 10 mg/kg LD followed by 2 mg/kg MD in (non-Japanese) MAD cohort 1.<br>The plasma ALS-008112 AUC_{0-24} for highest permissable dose in MAD cohort 2, 30 mg/kg LD/6 mg/kg MD, is projected to be 7,122 ng.h/ml on Day 1 and 2,810 ng.h/mL at steady state. These exposures are well within what has been studied in adult healthy volunteers in study ALS-8176-501.	A loading dose is required to rapidly achieve these NTP levels followed by maintenance doses (BID or Q12) which will maintain the constant levels of NTP that are required to inhibit viral replication…<br>…For cohort 1, a single loading dose of 10 mg/kg followed by 2 mg/kg BID or Q12 as maintenance doses is projected to achieve lung NTP levels within 2 hours that are needed for EC_{99} antiviral activity.<br>…Based on observed differences in PK between Japanese and non-Japanese subjects, the initial Japanese infant regimen of 4.1 mg/kg LD followed by a 1.37 mg/kg MD ~~is~~was estimated to approximate the exposures of the 10 mg/kg LD followed by 2 mg/kg MD in (non-Japanese) MAD cohort 1.<br>The plasma ALS-008112 AUC_{0-24h} for highest permissable dose in MAD cohort 2, 30 mg/kg LD/6 mg/kg MD, is projected to be approximately ~~7,122~~ 14,096 ng.h/ml on Day 1 and ~~2,810~~ 4,820 ng.h/mL at steady state, assuming linear pharmacokinetics. These exposures are similar to slightly higher than~~well within~~ what has been studied in adult healthy volunteers in study ALS-8176-501, where less than dose proportional exposure increases were observed.<br>For neonates, the planned dose is 10 mg/kg loading dose followed by 2 mg/kg maintenance dose MAD	Feedback from many sites indicates that dosing BID instead of Q12 in the MAD is much more feasible and preferable. Furthermore, there is no scientific or pharmacokinetic reason which would preclude BID dosing (the population PK for ALS-008176 is based on actual time of dosing and thus can evaluate data derived from either dosing regimen). The dosing regimen has therefore been modified to permit BID dosing to accommodate sites' requests.

Section	Original Text in Version 4.0	Revised Text in Version 5.0	Reason for Change
		regimen because, to date, this regimen has been well tolerated in infants 1-12 months of age. Furthermore, given that no age dependent differences in PK have been observed in 1-12 month olds, this dosing regimen is projected to deliver similar exposures in neonates as 1-12 month olds. The actual dose for the neonate cohort will be determined by the IDMC based on emerging PK and safety ± viral kinetics data from 1-12 month old cohorts and cannot exceed the doses, regimens and durations that are under evaluation in ≥1-≤12 month old subjects at that time.	
**1.3.2, 2.2, 2.3 and 5.1** *Synopsis and sections*	AUC_{0-24}	AUC_{0-24h}	Consistency with other sections
**2.1 STUDY DESIGN SUMMARY**	The preliminary dose escalation schema is presented in Figure 2-1.	The most current preliminary dose escalation schema is presented in Figure 2-1.	Update
**2.3 STUDY DESIGN PART 2 (MAD)**	In each dosing regimen, the first administered dose will consist of a single loading dose (Dose 1), followed 12 hours later by a twice daily (Q12h) maintenance dose regiment (Doses 2-10; Preliminary Dosing Schema*).<br><br>Up to two additional cohorts may be evaluated in Part 2 at the discretion of the Sponsor, upon approval by the IDMC,	~~In each dosing regimen, the first administered dose will consist of a single loading dose (Dose 1), followed 12 hours later by a twice daily (Q12h) maintenance dose regiment (Doses 2-10; Preliminary Dosing~~ Schema* ~~).~~In each dosing regimen, a single loading dose (Dose 1) will be followed by a twice daily (BID or Q12h) maintenance dose regimen (Doses 2–10; Preliminary Dosing Schema*). The 1st maintenance dose (Dose 2) may be given between 8-18 hours after the loading dose (Dose 1) to facilitate getting the subject on an established hospital dosing regimen (e.g., BID/Q12). Each dose of ALS-008176 must be separated by at least 8 hours from the prior and next dose.<br><br>Up to two additional cohorts may be evaluated in Part 2 at the discretion of the Sponsor, upon approval by the IDMC, based on the emerging PK profile and	See 1.3.2<br><br>See 1.2

Section	Original Text in Version 4.0	Revised Text in Version 5.0	Reason for Change
	based on the emerging PK profile and safety profile.<br><br>The IDMC will review emerging safety and PK data and dose escalate as appropriate.<br><br>Randomization will be stratified by age at time of hospital admission…with approximately 8 subjects being enrolled in each stratum.	safety profile. The first optional cohort will enroll up to 24 neonates (<28 days old) if supported by emerging PK, safety ± efficacy data in infants 1-12 months of age.<br><br>The IDMC will review emerging safety and PK data and dose ~~escalate~~ adjust as appropriate.<br><br>Randomization will be stratified by age (except neonate cohort) at time of hospital admission… with approximately 8 and up to 16 subjects being enrolled in each stratum.	
**3.1 STUDY OBJECTIVES**	In infants ≥ 1.0 to ≤ 12.0 months of age who are hospitalized with RSV infection:	In neonates (<28 days old) and infants (≥ 1.0 to ≤ 12.0 months of age) who are hospitalized with RSV infection:	See 1.2
**4.1 STUDY POPULATION**	Up to 48 more subjects may be enrolled into up to 2 additional dose cohorts.	Up to 24 neonates will be enrolled into one of the two optional MAD cohorts. Up to 24~~48~~ more subjects may be enrolled into ~~up to 2~~ one additional dose cohort~~s~~.	See 1.2
**4.2.3 INCLUSION CRITERIA**	3. Male or female infant who<br>• is ≥ 1.0 to ≤ 12.0 months of age (inclusive), defined at the time of hospital admission<br>• has been hospitalized for <96 hours for confirmed RSV infection (NOTE: nosocomial RSV infection is excluded)	3. Male or female infant who<br>• is ≥ 1.0 to ≤ 12.0 months of age (inclusive), defined at the time of hospital admission, or <28 days of age (neonate cohort only). Note: all subjects, including neonates, must have been discharged from the hospital after birth and are now being admitted due to an RSV related illness<br>• has been hospitalized for <96 hours (at time of randomization) for confirmed RSV infection (NOTE: nosocomial RSV infection is excluded)	See 1.2<br><br>Clarification
**4.3 EXCLUSION CRITERIA and 5.8 PROHIBITED MEDICATIONS**	5. …A single repeat laboratory evaluation is allowed for eligibility determination<br><br>7. Clinically significant abnormal ECG findings, as judged by the Investigator	5. …A single repeat laboratory evaluation under appropriate conditions, e.g. not hemolyzed) is allowed for eligibility determination<br><br>7. Clinically significant abnormal ECG findings, as judged by the Investigator or qualified designee	5. Clarification<br><br>7. Clarification

Section	Original Text in Version 4.0	Revised Text in Version 5.0	Reason for Change
	9d. Prescription medications which are known to be a strong inducer or inhibitor of CYP450 enzymes, within 21 days prior to randomization<br><br>• Prior exposure to palivizumab	9d. Prescription medications which are known to be a strong inducer or inhibitor of CYP450 enzymes or substrate for OAT3 transporter, within 21 days prior to randomization<br><br>• Prior exposure to palivizumab or other RSV prophylactic medication (approved or investigational)	9d Substrates for OAT3 transporter are included in the Prohibited Medications list<br><br>• The purpose of this criterion is:<br>-to minimize the risk that infants with serious comorbidities (and thus eligible for RSV prophylaxis) are randomized in this first-in-infant trial intended for otherwise healthy subjects<br>-to avoid the potential confounding effect of prior treatment with RSV prophylactic therapies on study outcomes.<br>As written, this exclusion criterion may inadvertently permit a subject previously exposed to an investigational RSV prophylactic therapy to be enrolled. This would undermine the intention of this criterion.
**4.4 SUBJECT SCREENING AND ENROLLMENT**	Procedures that are standard of care and performed within 96 hours prior to Screening may be used in determining protocol eligibility.<br>…over-enrollment of the cohort will be allowed, up to a maximum of 12 subjects per age group per cohort.	Procedures that are standard of care and performed within 96 hours prior to ~~Screening~~ randomization may be used in determining protocol eligibility.<br>…over-enrollment of the cohort will be allowed, up to a maximum of ~~12~~ 16 subjects per age group per cohort.	Consistency with Section 6.1.2 and 2.3

Section	Original Text in Version 4.0	Revised Text in Version 5.0	Reason for Change
**Table 5-1 Part 1 SAD Dosing Regimen**		Cohort No 4 updated to 25 mg/kg	Update
**Table 5-2 Part 2 MAD Dosing Regimen**	All cohorts:<br>…Q12 for 5 consecutive days…<br><br>Cohort No 3:<br>Up to 24 subjects (3 ALS-008176:1 placebo) - Doses to be given are TBD and will be given Q12 on 5 consecutive days.	All cohorts:<br>…BID or Q12 for 5 consecutive days…<br><br>Neonate cohort – Up to 24 subjects (3 ALS-008176:1 placebo) Planned dosing regimen – A loading dose of 10 mg/kg will be administered for Dose 1 on Day 1 followed by 2 mg/kg given BID or Q12 for 5 consecutive days (Doses 2-10). The actual regimen will be determined by the IDMC based on the emerging data but will not exceed the dosing regimen being evaluated in 1-12 month old subjects at the time the regimen is selected. ~~Doses to be given are TBD and will be given Q12 on 5 consecutive days.~~	See 1.2 and 1.3.2
**5.1.1.1 *Stopping Criteria***		Added:<br>• A dosing level may also be discontinued by the Sponsor at any time for administrative reasons – e.g., enrollment in a SAD cohort is minimal before the cohort is full because all sites are enrolling in the MAD.	Administrative update
**5.3 DOSE PREPARATION AND ADMINISTRATION And Table 6-2 Schedule of Events MAD Phase (Part 2) Footnote 4**	For MAD:<br>Study drug will be administered as a loading dose administered on Day 1 (Dose 1), followed by 9 maintenance doses given every 12 hours (Q12) on Day 1 through 5 (Doses 2-10).	For MAD:<br>~~Study drug will be administered as a loading dose administered on Day 1 (Dose 1), followed by 9 maintenance doses given every 12 hours (Q12) on Day 1 through 5 (Doses 2-10).~~<br>Footnote 1: Assessments to be performed once daily in the AM while still hospitalized. Day 5 Safety visit to be performed on Day 3, 4 or 5, regardless of whether subject is hospitalized.<br><br>Footnote 4:In each dosing regimen, a single loading dose (Dose 1) will be followed by a twice daily (BID	For consistency with Schedule of Events

Section	Original Text in Version 4.0	Revised Text in Version 5.0	Reason for Change
		or Q12h) maintenance dose regimen (Doses 2–10; Preliminary Dosing Schema*<br>). The 1st maintenance dose (Dose 2) may be given between 8-18 hours after the loading dose (Dose 1) to facilitate getting the subject on an established hospital dosing regimen (e.g., BID/Q12). Each dose of ALS-008176 must be separated by at least 8 hours from the prior and next dose.	See 1.3.2
**Table 6-1 Schedule of Events SAD Phase (Part 1) and**<br>**Table 6-2 Schedule of Events MAD Phase (Part 2)**	Assessments:<br>Nasopharyngeal Swab or Nasal Aspirate: RSV Diagnosis (Binax NOW RSV Test)<br><br>Day 2:<br>12 (±2) hr Post Dose 2 (MD)	Assessments:<br>Nasopharyngeal Swab or Nasal Aspirate: RSV Diagnosis ~~(Binax NOW RSV Test)~~<br><br>Day 2:<br>12 (±2) hr Post Dose 2* (MD)<br>* Or ±2 hours around the time of Dose 3 if on a BID rather than Q12 regimen	For consistency
**6.1.2** *Serum Chemistries and Complete Blood Count with Differential*<br>And<br>**6.1.3** *Pharmacokinetic Blood Sampling*	If blood collection volume is limited, the order of priority is the following: safety>PK>biomarkers (Screening, Day 5 Safety Visit and Follow-Up Visit (MAD only), & Completion Visit), PK>safety>biomarkers (Day 1).	If blood collection volume is limited, the order of priority is the following: safety>PK>biomarkers (Screening, Day 5 Safety Visit and Day 28 Follow-Up Visit (MAD only), & Completion Visit), PK>safety>biomarkers (Day 1).	Clarification
**6.1.5** *RSV Evaluations*	RSV Diagnosis<br>RSV diagnosis will be confirmed prior to randomization by rapid diagnostic assay using the study supplied Binax NOW RSV or any other RSV diagnostic test.	RSV Diagnosis<br>RSV diagnosis will be confirmed prior to randomization by rapid diagnostic assay using the study supplied Binax NOW RSV or any other RSV diagnostic test available at the site…Note: Informed consent must be obtained before conducting an RSV diagnostic test that is not a standard of care procedure.	Clarification

Section	Original Text in Version 4.0	Revised Text in Version 5.0	Reason for Change
**6.1.6** *12-Lead Electrocardiograms*	The investigator will be responsible for evaluating the results and determining if any findings are of clinical significance.	The investigator or qualified designee will be responsible for evaluating the results and determining if any findings are of clinical significance.	Clarification
**7.1.4** *Serious Adverse Events*	A serious adverse event is any untoward medical occurrence at any dose that:	A serious adverse event is any untoward medical occurrence at any dose that:<br>7.EU and Japan: transmission of infectious agents	Regulatory requirement
**7.2.6** *Documenting and Reporting Serious Pretreatment Events and Serious Adverse Events*	• Submit all available information to INVENTIV HEALTH Global Safety and Pharmacovigilance **by facsimile (preferred)** or email/telephone **within 24 hours** of becoming aware of the SAE, using the INVENTIV HEALTH Serious Adverse Event form (see contact information on page 2).	• Submit all available information to INVENTIV HEALTH Global Safety and Pharmacovigilance **by facsimile (preferred)** or email/telephone **within 24 hours** of becoming aware of the SAE, using the INVENTIV HEALTH Serious Adverse Event form, and inform the Medical Monitor via email (see contact information on page 2).	Clarification
**9.1** *Study Design and Objectives*	This randomized, double-blind, placebo-controlled, 2-part study will assess the safety, tolerability, pharmacokinetics (PK), and pharmacodynamics (PD) of single and multiple doses of orally administered ALS-008176 in infants hospitalized with RSV infection. Each infant will only be enrolled in a single cohort and a single part of the study…<br>Each stratum within each ascending dose cohort will consist of 8 infants, with 6 being randomized to receive ALS-008176 and 2 being randomized to receive placebo.	This randomized, double-blind, placebo-controlled, 2-part study will assess the safety, tolerability, pharmacokinetics (PK), and pharmacodynamics (PD) of single and multiple doses of orally administered ALS-008176 in neonates and infants hospitalized with RSV infection. Each ~~infant~~ subject will only be enrolled in a single cohort and a single part of the study…<br>(A separate neonatal (<28 days old) cohort will be enrolled as a stand alone cohort in the MAD only). Each stratum within each ascending dose cohort will consist of 8 and up to 16 infants~~, with 6~~ being randomized to receive ALS-008176 ~~and 2 being randomized to receive~~ or placebo in a 3:1 ratio.	See 1.2<br>Clarification
**9.4** *Determination of Sample Size*		In the neonate cohort, all subjects will be <28 days of age at the time of hospital admission.	See 1.2

Section	Original Text in Version 4.0	Revised Text in Version 5.0	Reason for Change
**9.5** *Randomization*		Each stratum within each ascending dose cohort will consist of approximately 8 and up to 16 infants~~, with 6~~ being randomized to receive ALS-008176 ~~and 2 being randomized to receive~~ or placebo in a 3:1 ratio.<br>...No stratification for age will be performed in the neonate cohort.	Clarification
**9.9** *Interim Analysis*	New section	A single interim analysis may be conducted during the conduct of the study if deemed necessary for reasons such as regulatory reporting requirements, Sponsor decision making, etc.	An interim analysis may be necessary to support certain Sponsor activities such as regulatory authority interactions and internal decision making regarding the design of subsequent studies of ALS-008176. The protocol was therefore modified to allow for one such interim analysis.
**10.2** *Informed Consent and Protected Subject Healthy Information Authorization*	Each subject must provide a signed and dated informed consent prior to enrollment into this study.<br><br>In accordance with individual local and national subject privacy regulations, the investigator or designee **must** explain to each subject's parent(s)/guardian(s) prior to screening that… As the study sponsor, Alios BioPharma will not use the subject's protected health information or disclose it to a third party without applicable subject authorization.	Each subject's parent(s)/guardian(s) must provide a signed and dated informed consent prior to enrollment into this study.<br><br>In accordance with individual local and national subject privacy regulations, the investigator or designee **must** explain to each subject's parent(s)/guardian(s) prior to screening that… As the study sponsor, Alios BioPharma will not use the subject's protected health information or disclose it to a third party without applicable subject's parent(s)/guardian(s) authorization.	Clarification

Section	Original Text in Version 4.0	Revised Text in Version 5.0	Reason for Change
**Appendix B Table 12-1, 12-2, 12-3, and 12-4**: *Estimated Blood Volume*	Screening Window (-1 to 1)	Screening Window (~~-1~~ -3 to 1)	Consistency with other sections
**Appendix B, Table12-3 and 12-4:** *Estimated Blood Volumes*	Total Volume at Follow up Visit Day 28 1.75	Total Volume at Follow up Visit Day 28 ~~1.75~~ 3.25	Correction

Appendix H. Summary of Protocol Changes from Version 5.0 dated 22 January 2016 to Version 6.0

Section	Original Text in Version 5.0	Revised Text in Version 6.0	Reason for Change
**Cover Page**<br>Page 1<br>*Date and Version Number*<br>**APPENDIX A**<br>**Investigator Signature Page**	22 January 2016<br>Version 5.0	13 September 2016<br>Version 6.0<br>Janssen Research and Development *<br>*Janssen Research & Development is a global organization that operates through different legal entities in various countries. Therefore, the legal entity acting as the sponsor for Janssen Research & Development studies may vary, such as, but not limited to Janssen Biotech, Inc.; Janssen Products, LP; Janssen Biologics, BV; Janssen-Cilag International NV; Janssen, Inc; Janssen Sciences Ireland UC; or Janssen Research & Development, LLC. The term "sponsor" is used throughout the protocol to represent these various legal entities; the sponsor is identified on the Contact Information page that accompanies the protocol.	To reflect revised version of protocol and Janssen (Alios parent company) as global sponsor
**CONTACT INFORMATION**<br>Page 2	[redacted]	Medical Monitor – Asia Pacific<br>[redacted]<br>P<br>D	Updated team member and contact information
**ABBREVIATIONS**		~~BID~~ twice daily<br>LD Loading Dose<br>MD Maintenance Dose	Added abbreviations for clarification
**1.1 BACKGROUND INFORMATION**	RNA is an RNA virus and a member of the *Paramyxoviridae* family, which also includes human metapneumovirus (hMPV) and parainfluenza virus 3 (PIV-3).	RNA is an RNA virus and a member of the *Pneumoviridae*~~*Paramyxoviridae*~~ family, which also includes human metapneumovirus (hMPV) ~~and parainfluenza virus 3 (PIV-3)~~.	RSV and hMPV are now part of the family *Pneumoviridae*. PIV-3 is still a member of the family *Paramyxoviridae*.

Section	Original Text in Version 5.0	Revised Text in Version 6.0	Reason for Change
**Figure 1, Preliminary Dosing Schema**<br>**Figure 2-1, Preliminary Dose Escalation Schema**			Single diagram replaced with Single Ascending Dose diagram and Multiple Ascending Dose diagram to reflect dose levels studied and plans for additional subjects.
**SYNOPSIS, 2.2**<br>**Study Design**<br>**Part 1 (SAD)**		As of 25 April 2016, the SAD portion of the study has been completed and is now closed.	Update
**SYNOPSIS, 2.3**<br>**Study Design**<br>**Part 2 (MAD)**<br>**5.1.1 Cohort Progression Guidelines**<br>**7.5 INDEPENDENT DATA MONITORING COMMITTEE**<br>**9.5**<br>**RANDOMIZATION**	Based on the lack of clinically relevant PK differences observed to date across the 3 age strata, infants of all 3 age strata will receive the same doses, unless otherwise instructed by the IDMC…<br>Hospitalized subjects with RSV infection will receive 1 of 2 planned MAD regimens of ALS-008176 or placebo…<br>~~Study drug will be dosed twice daily (i.e., BID or Q12) for a total of 10 doses administered over 5 days, or once daily for a total of 5 doses administered over 5 days. Dosing of study drug in the fed state is encouraged.~~<br>~~In each dosing regimen, a~~	Based on the lack of clinically relevant PK differences observed to date across the 3 age strata, infants of all 3 age strata (excluding neonates) will receive the same doses, unless otherwise instructed by the IDMC…<br>Hospitalized subjects with RSV infection will be assigned to 1 of up to 11 ~~receive 1 of 2~~ planned MAD regimens of ALS-008176 or placebo (see Figure 1). The number of dosing regimens and doses to be studied will be at the discretion of the Sponsor on the basis of the emerging PK and safety profiles, upon approval by the IDMC.<br>One or more cohorts will enroll (except when cohort enrollment in paused) ~~up to 24~~ neonates (<28 days old) if supported by emerging PK, safety, and efficacy data in infants 1-12 months of age. The dose regimen(s)	Clarification<br>To date, 122 subjects have been randomized across 4 SAD cohorts and 5 MAD cohorts. In order to fully characterize the dose response of various dosing regimens and frequencies (BID and QD) up to the maximum permitted exposure, it is estimated that up to 100 additional subjects may need to be enrolled in the MAD. The number of cohorts across which these subjects may be enrolled will be determined by the IDMC but, based on trends observed to date, it is likely all of these subjects would be enrolled in no more than 6 additional (11 total) cohorts. If data from the initial neonatal cohort appear acceptable, neonates may continue to be included among the additional up to 100 subjects to be reandomized.<br>As has always been the case in this study, the IDMC makes all final decisions as to the design of dosing regimens and cohorts based on emerging safety and PK data. Added text

Section	Original Text in Version 5.0	Revised Text in Version 6.0	Reason for Change
	~~single loading dose (Dose 1) will be followed by a twice daily (BID or Q12h) maintenance dose regimen (Doses 2–10; Preliminary Dosing~~ Schema* ~~). The 1st maintenance dose (Dose 2) may be given between 8–18 hours after the loading dose (Dose 1) to facilitate getting the subject on an established hospital dosing regimen (e.g., BID/Q12). Each dose of ALS-008176 must be separated by at least 8 hours from the prior and next dose.~~<br>~~Up to two additional cohorts may be evaluated in Part 2 at the discretion of the Sponsor, upon approval by the IDMC, based on the emerging PK profile and safety profile. The first optional~~ cohort will enroll up to 24 neonates (<28 days old) if supported by emerging PK, safety, and efficacy data in infants 1-12 months of age.<br>In addition, up to 24 Japanese infants will also	studied in neonates may be adjusted according to IDMC instructions.<br><br>In addition, ~~up to 24~~ Japanese infants will also be enrolled in Part 2 (see Section 1.1). Initially, Japanese patients ~~will~~ received a 4.1 mg/kg loading dose followed by nine 1.37 mg/kg maintenance doses (4.1/1.37). The IDMC ~~will~~ reviewed the emerging safety and PK data and ~~dose adjust as appropriate~~ due to lack of differences in exposures in Japanese vs. non-Japanese subjects in this study, Japanese infants are now eligible for inclusion in all cohorts and there is no restriction on how many Japanese infants may be enrolled.<br>Within each dosing cohort, subjects will be randomized to receive either ALS-008176 or placebo ~~(n=up to 24 per cohort; randomized~~ in a ratio of 3 ALS-008176: 1 placebo. Randomization (except in the neonate cohort) will be stratified by age… ~~with approximately 8 and up to 16 subjects being enrolled in each stratum.~~<br>Up to 6 additional cohorts may be enrolled in the MAD Part 2 with a maximum of 100 additional subjects (including neonates) in these cohorts. Additionally, the following elements of the study may be adjusted, subject to approval by the IDMC (see also sections 5.1.1 and 7.5):<br>• The size of each cohort and the frequency at which cohort data are reviewed. Enrollment will generally be allowed to continue during preparation and review of data by IDMC, unless otherwise instructed by the IDMC.<br>• Dosing frequency within a cohort may be once daily or twice daily	explicitly adds what other instructions the IDMC may provide in defining cohorts/regimens (e.g., inclusion of neonates, changing dosing frequency, modifying duration of symptoms at randomization and/or treatment duration).<br><br>Japanese subjects were initially restricted to a Japanese cohort when it was thought there may be PK differences in exposure in Japanese vs. non-Japanese subjects. Emerging data in this study indicate that Japanese subjects have similar PK to non-Japanese subjects thus Japanese subjects may now be included in all global cohorts and their enrollment is no longer capped.

Section	Original Text in Version 5.0	Revised Text in Version 6.0	Reason for Change
	be enrolled in Part 2 (see Section 1.1). Initially, Japanese patients will receive a 4.1 mg/kg loading dose followed by nine 1.37 mg/kg maintenance doses. The IDMC will review emerging safety and PK data and dose adjust as appropriate.<br>Within each dosing cohort, subjects will be randomized to receive either ALS-008176 or placebo (n=up to 24 per cohort; randomized in a ratio of 3 ALS-008176: 1 placebo. Randomization (except neonate cohort) will be stratified by age…with approximately 8 and up to 16 subjects being enrolled in each stratum.	• The maintenance doses may be the same as the loading dose<br>• The duration of dosing within a cohort will be 5 days unless otherwise instructed by the IDMC, which may modify the duration within the range of 3 to 10 days, inclusive<br>• The duration of symptoms from first onset until randomization will be ≤5 days unless otherwise instructed by the IDMC, which may modify the duration to up to 7 days<br>The sponsor will make recommendations to the IDMC based on reviews of blinded data.<br>Additionally, all subjects will receive standard supportive care as per local institution. Subjects will be evaluated over a 28-day period from the time of randomization. If they are discharged from the hospital prior to Day 28, they will be required to return for assessment as an outpatient on Day 5, if applicable, and on Days 11 and 28, when they will complete the study, assuming a 5-day treatment duration (see Table 6-2 for timing of visits for other treatment durations).<br>Throughout the conduct of the study, the pharmacokinetics of ALS-008112 and ALS-008144 will be evaluated and reviewed. An ~~established~~ pediatric population PK model will be updated with the additional data, and the plasma exposures of the subsequent dose will be simulated prior to dose escalation, as a safety check to ensure that the pharmacokinetics of ALS-008112 are not predicted to differ significantly from the intended exposures. Alterations in the planned dose escalation scheme will be made as necessary. In addition, under no circumstances will a planned pediatric dosing regimen~~e~~ exceed a projected average ALS-008112 $AUC_{0\text{-}24h}$ of	

Section	Original Text in Version 5.0	Revised Text in Version 6.0	Reason for Change
	Additionally, all subjects will receive standard supportive care as per local institution. Subjects will be evaluated over a 28-day period from the time of randomization. If they are discharged from the hospital prior to Day 28, they will be required to return for assessment as an outpatient on Day 5, if applicable, and on Days 11 and 28, when they will complete the study.<br>Throughout the conduct of the study, the pharmacokinetics of ALS-008112 and ALS-008144 will be evaluated and reviewed. An	20,000 ng•h/mL.	

Section	Original Text in Version 5.0	Revised Text in Version 6.0	Reason for Change
	established pediatric PK model will be updated with the additional data, and the plasma exposures of the subsequent dose will be simulated prior to dose escalation, as a safety check to ensure that the pharmacokinetics of ALS-008112 are not predicted to differ significantly from the intended exposures. Alterations in the planned dose escalation scheme will be made as necessary. In addition, under no circumstances will a planned pediatric dose exceed a projected average ALS-008112 AUC_{0-24h} of 20,000 ng•h/mL.		
**SYNOPSIS, 3.1.3, 9.1**<br>**Exploratory Study Objectives**		• To evaluate the relationship between viral kinetics and clinical outcomes<br>• To evaluate the PK of ALS-008112 and ALS-008144 (and other metabolites, if applicable) in nasal swabs following multiple doses of ALS-008176, if an acceptable testing methodology can be established	Additional exploratory objectives that may be informative in planning future studies were added.<br>If nasal samples may feasibly be evaluated for PK, this analysis will be performed as it defines exposures at the site of action of RSV (i.e., respiratory epithelium)
**SYNOPSIS, 3.2.2, 9.3.2**<br>**Secondary Endpoints**	• RSV viral RNA concentrations in nasal aspirates as measured by quantitative RT-	• RSV viral RNA concentrations in nasal swabs or aspirates as measured by quantitative RT-PCR	Previously, nasal aspirates were collected. The nasal sampling technique is being changed to nasal swabs because:<br>- They cause less discomfort for subjects

Section	Original Text in Version 5.0	Revised Text in Version 6.0	Reason for Change
	PCR		- They are easier for parents to do in the event sample collection post discharge is required<br>- there is no evidence that nasal swabs are an inferior technique to nasal aspiurates for quantifying viral load
**SYNOPSIS, 3.2.3, 9.3.3**<br>**Exploratory Endpoints**	• Time to resolution of RSV symptoms, such as runny nose, wheeze, cough, tachypnea…	• Time to resolution of RSV signs or symptoms, such as runny nose, wheeze, cough, tachypnea<br>• Relationship between viral kinetics and various clinical outcome measures (e.g., the relationship between RSV RNA viral load and oxygen supplementation, duration of hospitalization)…<br>• Concentrations of ALS-008112 and ALS-008144 (and other metabolites as applicable) in nasal secretions, if an acceptable testing methodology can be established	Additional exploratory endpoints that may be informative in planning future studies were added.<br>If nasal samples may feasibly be evaluated for PK, this analysis will be performed as it defines exposures at the site of action of RSV (i.e., respiratory epithelium)
**SYNOPSIS**<br>**Duration of Treatment and Study Period**	Part 2 (MAD): Twice daily (BID or Q12) dosing for 5 consecutive days; study duration is approximately 28 days.	Part 2 (MAD): Twice daily ~~(BID or Q12)~~ or once daily dosing for 5 consecutive days (unless otherwise instructed by the IDMC, which may select a dosing duration of 3-10 days); study duration is approximately 28 days.	In addition to defining the safety and efficacy of ALS-8176 with twice daily dosing, it may be useful to determine what effects, if any, a simpler once daily dosing regimen has on these parameters.<br>The additional parameters within a cohort (e.g. dosing duration, clinical parameters) that the IDMC may modify are defined in this protocol. Adjustments to text throughout the protocol are made to take into account these additional parameters.
**SYNOPSIS**<br>**Number of Sites and Location**	Approximately 70 sites in Europe (UK, France, Romania), Asia Pacific (Australia, New Zealand, Taiwan, Thailand, Japan), South Africa, and	Approximately 110~~70~~, including but not limited to, sites in Europe (UK, France, Romania), Asia Pacific (Australia, New Zealand, Taiwan, Thailand, Japan), South Africa, and North/Latin America (Canada, United States, Panama, Colombia, Chile) will participate.	More sites are expected to be required in order to be able to enroll an additional 100 subjects.

Section	Original Text in Version 5.0	Revised Text in Version 6.0	Reason for Change
	North/Latin America (Canada, United States, Panama, Colombia, Chile) will participate.		
**SYNOPSIS**<br>**Sample Size**<br>**4.1 STUDY POPULATION**<br>**Table 5-1 Part 1 SAD Dosing Regimen**<br>**Table 5-2 Part 2 MAD Dosing Regimen**<br>**9.4 DETERMINATION OF SAMPLE SIZE**	Part 1 (SAD): Up to 72 subjects will enroll in the ongoing planned cohorts (1.37, 4.1, and 12 mg/kg). Up to 72 more subjects may be enrolled into up to three additional cohorts.<br>Part 2 (MAD): Up to 48 subjects (not previously randomized in Part 1) will enroll in 2 MAD cohorts (N≤24/cohort). Up to 24 additional Japanese subjects will be enrolled in a separate MAD cohort. Up to 24 neonates will be enrolled in one of the two optional MAD cohorts. Up to 24 more subjects may be enrolled into one additional dose cohort.	Part 1 (SAD): Up to 72 subjects ~~will~~ were to enroll in SAD ~~the ongoing planned~~ cohorts. As of 25 April 2016, 70 subjects had enrolled in the SAD portion of the study, which is now closed~~. (1.37, 4.1, and 12 mg/kg). Up to 72 more subjects may be enrolled into up to three additional cohorts.~~<br>Part 2 (MAD): As of 5 August 2016, 52 subjects have enrolled across 5 MAD cohorts, some of which (e.g., 30/10) are actively recruiting (see Figure 1). Up to an additional 100 subjects will enroll in up to 6 additional cohorts. The maximum anticipated enrollment in the MAD portion of the study, taking into account these numbers as well as anticipated enrollment in ongoing cohorts during the protocol version 6 review process is 190 subjects (52 enrolled subjects + up to 23 additional neonates + up to 15 additional subjects at 30/10 dose level+100 additional subjects in future cohorts). ~~Up to 48 subjects (not previously randomized in Part 1) will enroll in 2 MAD cohorts (N≤24/cohort). Up to 24 additional Japanese subjects will be enrolled in a separate MAD cohort. Up to 24 neonates will be enrolled in one of the two optional MAD cohorts. Up to 24 more subjects may be enrolled into one additional dose cohort.~~	Text discussing the SAD section was not modified in any important way in this protocol except to state that the SAD portion of the study is closed.<br>MAD total enrollment figure updated to include additional 100 subjects plus all possible subjects that can be enrolled in ongoing plus previous cohorts.
**SYNOPSIS, 4.2**<br>**Inclusion Criteria**	3. Male or female infant who	3. Male or female infant who<br>• has had symptoms consistent with RSV infection (e.g., runny nose, cough, sneezing, fever, or tachypnea) for ≤ 5 days at the time of randomization (unless otherwise instructed by the IDMC, which may modify the duration to up to 7	The earlier in a subject's viral disease course, the more likely the subject is to benefit from an antiviral drug. A total symptom duration requirement was added to maximize the potential that future MAD subjects may benefit from future planned doses of ALS-8176 and thus maintain an acceptable risk-benefit profile.

Section	Original Text in Version 5.0	Revised Text in Version 6.0	Reason for Change
		days)	If emerging data suggest shorter or longer symptoms durations are required, the IDMC may instruct the Sponsor to adjust this duration.
**SYNOPSIS, 4.3**<br>**Exclusion Criteria**	9. Exclusionary medications include:<br>1. Herbal supplements which have evidence of adversely affecting absorption and clearance mechanisms (e.g., strong inhibitors/inducers of CYP450) within 21 days prior to randomization<br>2. The following prescription medications:<br>• Any chronically used, systemic prescription medications<br>• Use of systemic medications (either chronically or within the 21 days prior to randomization) which are known to modulate the host immune response and/or increase viral	9. Exclusionary medications include:<br>a. Herbal supplements which have evidence of adversely affecting absorption and clearance mechanisms (e.g., strong inhibitors~~/~~of OAT3~~/inducers of CYP450~~) within 21 days prior to randomization<br>b. The following prescription medications:<br>• ~~Any chronically used, systemic prescription medications~~<br>• Use of systemic medications (either chronically (i.e., >14 days for neonates and infants <2 months old, or >28 days for subjects ≥2-≤12 months of age) or within the 21 days prior to randomization) which are known to modulate the host immune response and/or increase viral shedding such as corticosteroids orother immunomodulatory therapies. The only exception is systemic corticosteroids will be acceptable if they are not taken chronically for a non-RSV-related indication.<br>• Prescription medications which are known to be~~ a~~ strong ~~inducer or~~ inhibitors of ~~CYP450 enzymes or substrate for~~ the OAT3 transporter, within 21 days prior to randomization (See Prohibited Medications, Section 5.8 ~~list in the Study Manual~~)<br><br>13. ~~Ethnically Japanese infants will not be enrolled~~	Nonclinical data indicate that only strong OAT3 inhibitors have the potential to interact in a potentially significant way with ALS-8176. Thus, the exclusionary, prohibited, and concomitant medication sections were modified to permit all drugs which are not known to be strong OAT3 inhibitors.<br>The definition of a "chronically" administered medication was defined.<br>Ethnically Japanese subjects may be enrolled anywhere globally because there are no apparent ethnic PK differences between Japanese and non-Japanese subjects.

Section	Original Text in Version 5.0	Revised Text in Version 6.0	Reason for Change
	shedding such as corticosteroids orother immunomodulatory therapies. The only exception is systemic corticosteroids will be acceptable if they are not taken chronically for a non-RSV-related indication.<br>• Prescription medications which are known to be a strong inducer or inhibitor of CYP450 enzymes or substrate for OAT3 transporter, within 21 days prior to randomization (See Prohibited Medication list in the Study Manual)<br>13. Ethnically Japanese infants will not be enrolled outside of Japan	~~outside of Japan~~	

Section	Original Text in Version 5.0	Revised Text in Version 6.0	Reason for Change
**SYNOPSIS**<br>**Dose Regimen**<br>**2.3 Part 2 (MAD)**<br>**5.3 DOSE PREPARATION AND ADMINISTRATION**	Part 2 (MAD): BID or Q12 dosing for 5 consecutive days. In each BID dosing regimen, a single loading dose (Dose 1) will be followed by a twice daily maintenance dose regimen (Doses 2–10; Figure 1). The first maintenance dose (Dose 2) may be given between 8-18 hours after the loading dose (Dose 1) to facilitate getting the subject on an established hospital dosing regimen (e.g., BID/Q12). Each dose of ALS-008176 must be separated by at least 8 hours from the prior and next dose.	Part 2 (MAD): Once daily or ~~BID~~twice daily~~or Q12~~ dosing for 5 consecutive days (for a 5-day treatment duration). Regardless of dosing frequency, the loading dose (Dose 1) should be administered as soon as possible after randomization.<br>In each ~~BID~~twice dailydosing regimen, ~~a single~~the loading dose (Dose 1) will be followed by a twice daily maintenance dose regimen (Doses 2–10; Figure 1). The first maintenance dose (Dose 2) ~~may~~ will be given between 8-18 hours after the loading dose (Dose 1) to facilitate getting the subject on an established hospital dosing regimen ~~(e.g., BID/Q12).~~ All subsequent doses will be given per the hospital's regular twice daily dosing times ±1 hour. ~~Each dose of ALS-008176 must be separated by at least 8 hours from the prior and next dose.~~<br>In once daily dosing regimens, Dose 2 will be given between 21-27 hours after Dose 1 to facilitate getting the subject on an established hospital dosing regimen. All subsequent doses will be given per the hospital's regular once daily dosing times ±1 hour.<br>Subjects will receive a total of 10 doses administered over 5 days if dosed twice daily or a total of 5 doses administered over 5 days if dosed once daily, assuming a 5-day treatment duration.<br>ALS-008176 can be administered without regard to food.<br>If instructed by the IDMC, the maintenance dose may be the same dose as the loading dose.	Regardless of dosing frequency, the loading dose should be administered as soon as possible in order to maximize the chance that subjects may benefit from antiviral effects of ALS-8176.<br>The timing of the 2nd and subsequent doses for all frequencies was defined to make it more convenient for site staff to get their study subjects onto a local hospital dosing schedule<br>Additional PK information suggest that the effects of food on PK are not sufficiently different to jusify a requirement to time dosing around food intake.<br>Additional IDMC instruction that are now permissible (loading dose may = maintenance dose) was explicitly stated.
**1.1 Background Information**	ALS-008112 and ALS-008176 are potent and highly selective inhibitors of both RSV A	ALS-008112 and ALS-008176 are potent and highly selective inhibitors of both RSV laboratory-adapted A and B strains as well as a range of diverse clinical isolates. In addition, both inhibit RSV replication in the	Clarification

Section	Original Text in Version 5.0	Revised Text in Version 6.0	Reason for Change
	and B strains as well as a range of diverse clinical isolates. In addition, both inhibit RSV replication in the replicon system with IC_{50} values of 0.15 µM and 0.26 µM, respectively. Inside cells, ALS-008112 is efficiently converted to ALS-008112-5′-triphosphate (ALS-008136 or NTP), the active metabolite of the compound, which is a potent and selective inhibitor of RSV RNA polymerase activity, via a classic chain termination mechanism…<br><br>Based on these results, following 14 days of dosing with ALS-008176, the no-observed-adverse-effect level (NOAEL) was determined to be 150 mg/kg BID or 300 mg/kg/day (ALS-008112 $AUC_{0\text{-}24\,h}$ 53,600 ng•h/mL) in adult rats; 75 mg/kg BID or 150 mg/kg/day (ALS-008112 $AUC_{0\text{-}24\,h}$ 192,500 ng•h/mL) in adult dogs. The NOAEL in juvenile rats dosed	sub-genomic replicon system with IC_{50} values of 0.15 µM and 0.26 µM, respectively. Inside cells, ALS-008112 is efficiently converted to ALS-008112-5′-triphosphate (ALS-008136 or NTP), the active metabolite of the compound, which is a potent and selective inhibitor of RSV RNA-dependent RNA polymerase activity, via a classic chain termination mechanism…<br><br>Based on these results, following 14 days of dosing with ALS-008176, the no-observed-adverse-effect level (NOAEL) was determined to be 150 mg/kg BIDtwice daily or 300 mg/kg/day (ALS-008112 $AUC_{0\text{-}24\,h}$ 53,600 ng•h/mL) in adult rats; 75 mg/kg BIDtwice daily or 150 mg/kg/day (ALS-008112 $AUC_{0\text{-}24\,h}$ 192,500 ng•h/mL) in adult dogs. The NOAEL in juvenile rats dosed from PND1-28 was 150 mg/kg ALS-008176 BIDtwice daily or 300 mg/kg/day (ALS-008112 $AUC_{0\text{-}24\,h}$ 93,900 ng•h/mL), and for rats dosed from PND21-45 was 75 mg/kg ALS-008176 BIDtwice daily or 150 mg/kg/day (ALS-008112 $AUC_{0\text{-}24\,h}$ 32,950 ng•h/mL).	

Section	Original Text in Version 5.0	Revised Text in Version 6.0	Reason for Change
	from PND1-28 was 150 mg/kg ALS-008176 BID or 300 mg/kg/day (ALS-008112 $AUC_{0\text{-}24\,h}$ 93,900 ng•h/mL), and for rats dosed from PND21-45 was 75 mg/kg ALS-008176 BID or 150 mg/kg/day (ALS-008112 $AUC_{0\text{-}24\,h}$ 32,950 ng•h/mL).		
**1.1 Background Information**	**Clinical Data – Safety**<br><br>As of 20 January2016, 165 healthy volunteers have received ALS-008176 as single doses up to 750 mg or multiple doses up to a 750 mg loading dose (LD) followed by a 500 mg maintenance dose (MD; dosed twice daily, 12 hours apart [Q12]) for a total duration of up to 14 days. Additionally, in this study 79 infants 1–12 months of age have received single doses (N=67) of up to 25 mg/kg or multiple doses (N=12) consisting of up to a 10 mg/kg loading dose followed by up to 2 mg/kg maintenance doses of ALS-008176 or	**Clinical Data ~~– Safety~~(as of 5 August 2016)**<br><br>***Study Populations, Dosing Regimens Evaluated***<br><br>As of ~~20 January~~5 August 2016, ~~165~~ 192 healthy volunteers have received ALS-008176 as single doses up to ~~750~~ 3000 mg or multiple doses up to a 750 mg loading dose (LD) followed by a 500 mg maintenance dose (MD; dosed twice daily, 12 hours apart [Q12]) for a total duration of up to 14 days.<br><br>In ongoing Study ALS-8176-510, which is assessing the safety and tolerability of a 750 mg LD followed by nine 500 mg MDs of ALS-008176 or placebo in elderly subjects hospitalized with RSV infection, 6 subjects have been dosed.<br><br>In the current study (Study ALS-8176-503), 122 infants and neonates have been enrolled. Seventy (70) subjects have enrolled in the SAD portion of the study, with 24, 24, 18, and 4 subjects enrolled in the 1.37, 4.1, 12, and 25 mg/kg cohorts, respectively. Enrollment in the SAD portion of the study is now closed. In the multiple	An update of all known data from clinical studies as of 5 August was provided to the background section.

Section	Original Text in Version 5.0	Revised Text in Version 6.0	Reason for Change
	placebo.	ascending dose (MAD) portion of the study, 6, 23, 14, and 9 subjects have enrolled in the 4.1/1.37, 10/2, 30/6, and 30/10 mg/kg cohorts, respectively. Enrollment by age stratum and dose level for the SAD and MAD portions of the study can be found in Figure 1-1.<br><br>In the MAD portion of the study, 6 Japanese subjects enrolled into the 4.1/1.37 dosing regimen before the IDMC instructed that Japanese subjects may enroll at the same dosing regimen as the rest of world.<br><br>**Figure 1-1. Study ALS-8176-503 Enrollment Status as of 5 August 2016**<br><br>~~Additionally, in this study 79 infants 1–12 months of age have received single doses (N=67) of up to 25 mg/kg or multiple doses (N=12) consisting of up to a 10 mg/kg loading dose followed by up to 2 mg/kg maintenance doses of ALS-008176 or placebo.~~	
**1.1 Background Information**	In all studies conducted to date, ALS-008176 has been well tolerated. Three serious adverse events (SAE) have been reported in the ongoing study in hospitalized infants…	**Safety – Serious Adverse Events**<br>In all studies conducted to date, ~~ALS-008176 has been well tolerated. Three~~ serious adverse events (SAE) have been reported, all of which occurred in the ongoing study in hospitalized infants… The fourth SAE (lymphadenitis) occurred with onset 16 days after an infant completed a 30/6 dosing regimen and became serious when the infant was hospitalized 24 days after completing treatment. The event was considered	An update of all known data from clinical studies as of 5 August was provided to the background section.

Section	Original Text in Version 5.0	Revised Text in Version 6.0	Reason for Change
	To date, no adverse events (AEs) in any study have led to study drug discontinuation…(see discussion in Investigator's Brochure v5 Section 6.5.1.2.2). The third severe event (increased cholesterol) occurred in Study ALS-8176-511/64041575RSV1001 (Japanese ethnic bridging study) 13 days after receiving a single dose of ALS-008176/placebo (the study remains blinded) and was considered possibly related to study drug…as discussed in the current IB (v5, Section 6.5.1.2.2).<br><br>To date, all AEs (N=73; SAD N=57; MAD N=16) in Study ALS-8176-503 except the pneumonia SAE, which was considered severe, have	unrelated to study drug.<br><br>**Safety – Adverse Events, Severities**<br><br>To date, no adverse events (AEs) in any study involving ALS-008176 have led to study drug discontinuation…(see discussion in Investigator's Brochure v5 Section 6.5.1.2.2). The third severe event (increased cholesterol low density lipoprotein) occurred in Study ALS-8176-511/64041575RSV1001 (Japanese ethnic bridging study) 13 days after receiving a single dose of ALS-008176/placebo (the study remains blinded) and was considered possibly related to study drug…as discussed in the current IB (v5, Section 6.5.1.2.2).<br><br>In Study ALS-8176-510, among the 6 elderly subjects enrolled as of 5 August 2016, all AEs (N=7), have been mild (N=6) or severe (N=1) in intensity. The severe event (neutropenia) occurred 23 days after the last dose of study drug and was considered by the investigator to be possibly related to study drug. The event has since resolved without sequelae.<br><br>To dateAs of 5 August 2016, all subjects reported 117 AEs (N=73; SAD N=57; MAD N=1660) in Study ALS-8176-503. All AEs, except the pneumonia SAE (N=1), which was considered severe, have been mild (N=5895) or moderate (N=1421) in severity. A blinded summary table of all treatment-emergent AEs reported as of 20 January5 August 2016 is presented in Table 1-1 (SAD) and Table 1-2 (MAD).	

Section	Original Text in Version 5.0	Revised Text in Version 6.0	Reason for Change
	been mild (N=58) or moderate (N=14) in severity. A blinded summary table of all treatment-emergent AEs reported as of 20 January 2016 is presented in Table 1-1 (SAD) and Table 1-2 (MAD).<br><br>Unblinded safety data from this study are regularly reviewed by an Independent Data Monitoring Committee (IDMC), which has expressed no safety concerns based on a cumulative review of safety and PK data as recently as 17 December 2015.<br><br>Table 1-1. Incidence and Severity of Treatment-emergent AEs in SAD Portion of Study ALS-8176-503 (as of 20 January 2016)<br><br>Table 1-2. Incidence and Severity of Treatment-emergent AEs in MAD Portion of Study ALS-	Unblinded safety data from this study are regularly reviewed by an Independent Data Monitoring Committee (IDMC), which has expressed no safety concerns based on a cumulative review of safety and PK data as recently as 15 August 2016~~17 December 2015~~.<br><br>Table 1-1. Blinded Incidence and Severity of Treatment-emergent AEs in SAD Portion of Study ALS-8176-503 (as of ~~20 January~~ 5 August 2016)<br><br>Table 1-2. Blinded Incidence and Severity of Treatment-emergent AEs in MAD Portion of Study ALS-8176-503 (as of ~~20 January~~ 5 August 2016)<br><br>***Safety – Laboratories, ECGs, Vital Signs, and Physical Examinations***<br><br>In all studies of ALS-008176 in ~~both~~ healthy volunteers and naturally infected elderly subjects and infants, no clinically significant laboratory, electrocardiogram (ECG), vital sign, or physical examination findings suggestive of a safety concern have been identified.<br><br>***Clinical Data – Efficacy***<br><br>…There was an accompanying decrease in RSV signs and symptoms that corresponded to these reductions in vital load (Investigator's Brochure ~~v5~~ Section 6.3).<br><br>***Clinical Data – Pharmacokinetics***<br><br>(Section updated)	

Section	Original Text in Version 5.0	Revised Text in Version 6.0	Reason for Change
	8176-503 (as of 20 January-2016)<br>In all studies of ALS-008176 in both healthy volunteers and infants, no clinically significant laboratory, electrocardiogram (ECG), vital sign, or physical exam findings suggestive of a safety concern have been identified.<br>***Clinical Data – Efficacy***<br>…There was an accompanying decrease in RSV signs and symptoms that corresponded to these reductions in vital load (Investigator's Brochure v5 Section 6.3).	Added Figure 1-3. ALS-008112 Exposures in Infants 1-12 Months of Age Receiving 30 mg/kg LD and 10 mg/kg MD Regimen in Study ALS-8176-503	
**1.2 RATIONALE FOR THE STUDY**	This study is being performed to determine preliminary safety, tolerability, pharmacokinetic (PK), and pharmacodynamic (PD) data after single and multiple doses of ALS-008176 given to infants ≥ 1.0 to ≤ 12.0 months of age and neonates who are hospitalized with RSV infection.	This study is being performed to determine preliminary safety, tolerability, ~~pharmacokinetic (~~PK~~)~~, and ~~pharmacodynamic (~~PD~~)~~ data after single and multiple doses of ALS-008176 given to infants ≥1.0 to ≤12.0 months of age and neonates who are hospitalized with RSV infection. The study is being amended (v6) to allow for additional subjects to be enrolled in order to continue to define the safety, PK, and viral kinetics of ALS-008176 and its metabolites throughout the previously defined permissible exposures range (i.e., mean projected plasma ALS-008112 $AUC_{0\text{-}24h}$ up to 20,000 ng•h/mL). An additional 100 subjects may be needed to fully characterize this exposure range depending on the rate of dose escalation, as defined by	Rationale updated to reflect that an additional up to 100 subjects may be enrolled in order to continue to define the safety, PK, and viral kinetics of ALS-008176 and its metabolites throughout the previously defined permissible exposures range.

Section	Original Text in Version 5.0	Revised Text in Version 6.0	Reason for Change
		the IDMC, and in the event the Sponsor is also authorized by IDMC to explore additional dosing regimens (e.g. once daily).	
**1.3 RATIONALE FOR STARTING DOSE SELECTION**	For estimating of starting doses…predicted for a variety of dose regimens.<br><br>The proposed starting dose in infants takes into consideration the following:…<br><br>Reversible and monitorable nonclinical toxicology profile of ALS-008112 and margins from the nonclinical safety studies, specifically to limit the mean plasma exposure of ALS-008112 AUC_{0-24} to <20,000 ng•h/mL.	For estimation~~estimating~~ of starting doses…predicted for a variety of dosing~~e~~ regimens.<br><br>The proposed starting dose in infants takes into consideration the following:…<br><br>Reversible and monitorable nonclinical toxicology profile of ALS-008112 and margins from the nonclinical safety studies, specifically to limit the ~~mean~~ average plasma exposure of ALS-008112 AUC_{0-24} to <20,000 ng•h/mL. (Note: All references to average plasma exposures in infants refer to the geometric mean exposure.)	Clarification
**1.3.2 Multiple Ascending Dose**	The 3 dose regimens span the project therapeutic dose ranges.<br><br>The plasma ALS-008112 AUC_{0-24h} for highest permissable dose in MAD Cohort 2, 30 mg/kg LD/6 mg/kg MD, is projected to be	The ~~3~~planned dosing~~e~~ regimens span the projected therapeutic dose ranges.<br><br>The geometric mean plasma ALS-008112 AUC_{0-24h} for ~~highest permissable dose~~the 30/6 mg/kg dosing regimen in MAD Cohort 2~~, 30 mg/kg LD/6 mg/kg MD,~~ is ~~projected~~ estimated to be approximately 11,538 ~~14,096~~ ng•h/mL on Day 1 and 6,025 ~~4,820~~ ng•h/mL ~~at steady state~~on Day 5. ~~assuming linear pharmacokinetics.~~ These exposures are similar to (at	The pediatric popPK model was updated with more recent data. Updated PK results and projections for higher doses, including the highest projected dose that remains less than the maximum permissible ALS-8112 (0-24h) exposure (i.e., <20,0000 ng*hr/mL) were added.

Section	Original Text in Version 5.0	Revised Text in Version 6.0	Reason for Change
	approximately 14,096 ng.h/mL on Day 1 and 4,820 ng.h/mL at steady state, assuming linear pharmacokinetics. These exposures are similar to slightly higher than what has been studied in adult healthy volunteers in Study ALS-8176-501 , where less than dose proportional exposure increases were observed. Two optional cohorts may be studied to further define dose-response relationships.	Day 1) and approximately half (Day 5) of the exposures ~~slightly higher than what has been~~that were ~~studied~~ found to be efficacious in adult healthy volunteers in Study ALS-8176-~~501~~502 (Challenge Study). ~~, where less than dose proportional exposure increases were observed. Two optional cohorts may be studied to further define dose-response relationships.~~The 30/10 mg/kg dosing regimen is projected to deliver a geometric mean Day 1 and Day 5 ALS-008112 AUC_{0-24h} plasma exposure of 12,683 and 9579 ng•h/mL, respectively. Based on the pediatric population PK model, the highest dosing regimen which is projected to deliver an average ALS-008112_{0-24h} AUC <20,000 ng•h/mL is a 40/20 mg/kg regimen, which is projected to have geometric mean Day 1 and Day 5 exposures of 19,062 ng•h/mL and 18,929 ng•h/mL, respectively. These exposures maintain a safety margin of >2.7-fold relative to the lowest NOAEL of the most sensitive toxicology species.	
**4.4 SUBJECT SCREENING AND ENROLLMENT**<br>**5.1 TREATMENT REGIMENS**<br>**6.0 STUDY PROCEDURES**	Subjects must receive the first dose of study drug within 8 hours after randomization.<br><br>If multiple potential subjects are being screened on the same day for the final position in a cohort, over-enrollment of the cohort will be allowed, up to a maximum of 16 subjects	Subjects must receive the first dose of study drug within 18 hours after randomization…Cohort sizes and dosing regimens will be determined by the IDMC based on emerging safety and PK data within the constraints defined in Section 5.1.1.<br><br>If multiple potential subjects are being screened on the same day for the final position in a cohort, over-enrollment of the cohort will be allowed, up to a maximum of ~~16~~ 4 extra subjects ~~per age group~~ per cohort.	Updated to reflect additional parameters the IDMC may modify in subsequent cohorts.<br><br>With all age strata enrolling concurrently, overenrollment at a moment should not exceed 4 subjects. 16 subjects only applied when all 4 age strata enrolled at different points in time.

Section	Original Text in Version 5.0	Revised Text in Version 6.0	Reason for Change
	per age group per cohort.		
**5.1.2 Treatment Delay or Missed Doses**	If a dose is missed, then it should be administered within 6 hours after the scheduled dosing time.	For twice daily dosing, if a dose is missed, then it should be administered within 6 hours after the scheduled dosing time…<br><br>For once daily dosing, if a dose is missed, then it should be administered within 12 hours after the scheduled dosing time. If a missed dose cannot be administered within 12 hours after the scheduled dosing time, then it should be skipped, and the next dose should be given at the scheduled time.	How to handle delayed or missed doses for a once daily regimen was added now that a once daily regimen is permitted.
**5.3 DOSE PREPARATION AND ADMINISTRATION**	Feeding is recommended within 1 hour prior to study drug administration…First study drug dose should be given as soon as possible but no later than 8 hours after randomization.	~~Feeding is recommended within 1 hour prior to study drug administration.~~ ALS-008176 can be administered without regard to food… First study drug dose should be given as soon as possible but no later than 18 hours after randomization.	Additional PK information suggest that the effects of food on PK are not sufficiently different to jusify a requirement to time dosing around food intake.
**5.8 PROHIBITED MEDICATIONS**	Prohibited medications during the conduct of this study include:<br><br>• Herbal supplements which have evidence of adversely affecting absorption and clearance mechanisms (e.g., strong inhibitors/inducers of	Prohibited medications during the conduct of this study include:<br><br>• Herbal supplements which have evidence of adversely affecting absorption and clearance mechanisms (e.g., strong inhibitors of OAT3~~/inducers of CYP450~~)…<br><br>• Prescription medications which ~~is~~are known to be~~ a~~ strong ~~inducer or~~ inhibitors of ~~CYP450 enzymes or substrate of~~ the OAT3 transporter	Nonclinical data indicate that only strong OAT3 inhibitors have the potential to interact in a potentially significant way with ALS-8176. Thus, the exclusionary, prohibited, and concomitant medication sections were modified to permit all drugs which are not known to be strong OAT3 inhibitors.

Section	Original Text in Version 5.0	Revised Text in Version 6.0	Reason for Change
	CYP450…<br><br>• Prescription medication which is known to be a strong inducer or inhibitor of CYP450 enzymes or substrate of OAT3 transporter	are prohibited:<br><br>○ Cimetidine<br>○ Diclofenac<br>○ Probenecid<br><br>NOTE: The list of Prohibited Medications above is subject to change (e.g., if new OAT3 substrates or inhibitors are identified in the medical literature after finalization of the protocol). Any revisions to the list above will be available in the Study Manual.	
**Table 6-2** ***Schedule of Events MAD Phase (Part 2) Twice Daily Dosing***		Window for 7 hour post dose 2 assessments increased to 7 (±4) hr<br><br>Safety Visit on last day of dosing<br><br>Completion Visit 6 days after the last day of dosing<br><br>Randomization ≤18 hrs predose<br><br>Nasal Swab[10] or Aspirate: PD (RSV Viral RNA), resistance, PK[11], biomarkers<br><br>Footnotes 1-8 updated; new footnotes 10,11	Previously, nasal aspirates were collected. The nasal sampling technique is being changed to nasal swabs because:<br>- They cause less discomfort for subjects<br>- They are eaiser for parents to do in the event sample collection post discharge is required<br>- there is no evidence that nasal swabs are an inferior technique to nasal aspiurates for quantifying viral load<br><br>If nasal samples may feasibly be evaluated for PK, this analysis will be performed as it defines exposures at the site of action of RSV (i.e., respiratory epithelium)
**Table 6-3** ***Schedule of Events MAD Phase (Part 2) Once Daily Dosing***		New table	

Section	Original Text in Version 5.0	Revised Text in Version 6.0	Reason for Change
**6.1 ON-STUDY EVALUATIONS, PROCEDURES, AND DOSING**<br>**6.1.6 12-Lead ECG**<br>**6.1.7 Vital Signs and Oxygen Saturation**	…nasal aspirates…	…nasal <u>swabs or</u> aspirates	Previously, nasal aspirates were collected. The nasal sampling technique is being changed to nasal swabs because:<br>- They cause less discomfort for subjects<br>- They are eaiser for parents to do in the event sample collection post discharge is required<br>- there is no evidence that nasal swabs are an inferior technique to nasal aspiurates for quantifying viral load
**6.1.1 Diet, Fluid, and Activity**	Dose selection for this study is based on the assumption that subjects will be in a fasted state which, in healthy adults, provides the higher ALS-008112 C_{max} compared to fed state. Because the presence of food lowers the C_{max} of ALS-008112 without affecting its AUC, dosing of study drug in the fed state is encouraged. See also Section 1.11.1, *Background Information*.<br>Feeding is recommended within 1 hour prior to every dose. Feeding times will be recorded. There are no requirements for fasting. Study drug must not be added to a feeding	~~Dose selection for this study is based on the assumption that subjects will be in a fasted state which, in healthy adults, provides the higher ALS-008112 C_{max} compared to fed state. Because the presence of food lowers the C_{max} of ALS-008112 without affecting its AUC, dosing of study drug in the fed state is encouraged. See also Section 1.11.1, *Background Information*.~~<br>~~Feeding is recommended within 1 hour prior to every dose.~~ <u>ALS-008176 can be administered without regard to food.</u> Feeding times will be recorded. ~~There are no requirements for fasting.~~ Study drug must not be added to a feeding bottle or pacifier.	Additional PK information suggest that the effects of food on PK are not sufficiently different to jusify a requirement to time dosing around food intake.

Section	Original Text in Version 5.0	Revised Text in Version 6.0	Reason for Change
	bottle or pacifier.		
**6.1.2 Serum Chemistries and Complete Blood Count with Differential**	If blood collection volume is limited, the order of priority is the following: safety>PK>biomarkers (Screening, Day 5 Safety Visit and Day 28 Follow-up Visit (MAD only), and Completion Visit), PK>safety>biomarkers (Day 1).	If blood collection volume is limited, the order of priority is the following: safety>PK>biomarkers (Screening, ~~Day 5~~ Safety Visit/Last Day of Dosing and Day 28 Follow-up Visit (MAD only), and Completion Visit), PK>safety>biomarkers (Day 1).	The names of study visits were modified to more easily identify them in the event that dosing duration changes based on IDMC insutrction (e.g. Day 5 visit now Last Day of Dosing Visit)
**6.1.3 Pharmacokinetic ~~Blood~~ Sampling**	Each subject will have a maximum of 4 (for SAD) or 6 (for MAD) study-mandated blood samples collected over the course of the study in order to determine the safety and PK of ALS-008176…<br>Subjects participating in Part 2 (MAD) will have one additional PK lab sample collected on Day 5 and one blood sample, if necessary, for safety labs and biomarkers collected on Day 28…<br>If blood collection volume is limited, the order of priority is the following: safety>PK>biomarkers	Each subject will have a maximum of 4 (for SAD) or 6 (for MAD for dosing duration <9 days) or 7 (for MAD dosing duration of 9-10 days) study-mandated blood samples collected over the course of the study in order to determine the safety and PK of ALS-008176…<br>Subjects participating in Part 2 (MAD) will have one additional PK lab sample collected on Last Day of Dosing~~Day 5~~ (and one on Day 5 if dosing duration is 9-10 days) and one blood sample, if necessary, for safety labs and biomarkers collected on Day 28…<br>If blood collection volume is limited, the order of priority is the following: safety>PK>biomarkers (Screening, ~~Day 5 Safety Visit~~Last Day of Dosing/Day 5 for 9-10 days dosing duration and Day 28 Follow-up Visit (MAD only), and Completion Visit), PK>safety>biomarkers (Day 1)…<br>Concentrations of ALS-008112 and ALS-008144 (and other metabolites as applicable) may also be evaluated in nasal swabs if a sensitive method can be established.	The schedule of events was updated to reflect possibly changes based on IDMC instructions. This includes adding additional assessments in the event the treatment duration is extended to 9 or 10 days.<br>The names of study visits were modified to more easily identify them in the event that dosing duration changes based on IDMC insutrction (e.g. Day 5 visit now Last Day of Dosing Visit)<br>If nasal samples may feasibly be evaluated for PK, this analysis will be performed as it defines exposures at the site of action of RSV (i.e., respiratory epithelium)

Section	Original Text in Version 5.0	Revised Text in Version 6.0	Reason for Change
	(Screening, Day 5 Safety Visit and Day 28 Follow-up Visit (MAD only), and Completion Visit), PK>safety>biomarkers (Day 1).		
**6.1.4 Central Laboratory Evaluations**	Central laboratories will be used for qualitative PCR RSV diagnostic confirmation…	Central laboratories will be used for confirmatory qualitative PCR RSV diagnostic confirmation…	Clarification
**6.1.5 RSV Evaluations**	**Qualitative PCR**<br><br>Qualitative PCR will be performed by a central lab, using predose nasal aspirate sample, to confirm RSV diagnosis, however, study eligibility is determined by the results of the local RSV test.	**Nasal swabs**<br><br>Upon receiving approval of protocol v6, nasal swab specimens will be collected. The procedure for sample collection is defined in the Study Manual. Supplies to perform the nasal swab procedure will be provided to sites by the Sponsor. Parents/guardians may be asked to collect nasal swab specimens; site personnel will provide all needed supplies, procedure instructions, and storage requirements, all of which will be defined in the Study Manual.<br><br>**Qualitative PCR**<br><br>Qualitative PCR will be performed by a central lab, using predose nasal swab or nasal aspirate sample, to confirm RSV diagnosis, however, study eligibility is determined by the results of the local RSV test.	Text defining nasal swabs that are now planned to be collected was added to define the sampling technique and procedures in place to assure proper sample collection
**8.2 SAFETY VARIABLES/**	In this study, an estimated total of up to 5.65 or 16.85 mL of	In this study, an estimated total of up to 5.65 or 16.85 mL of blood will be drawn in each SAD or MAD study subject (dosing duration <9 days), respectively, over the	Updated to reflect changes made in this protocol amendment.

Section	Original Text in Version 5.0	Revised Text in Version 6.0	Reason for Change
MEASUREMENTS	blood will be drawn in each SAD or MAD study subject, respectively, over the course of the study. This blood volume represents 1.66 or 4.96 mL/kg for a 3.4-kg infant (~10th percentile for a 1-month old) and is within the generally accepted range for blood draws for pediatric clinical research studies (Howie 2011). Actual blood volumes may vary at each institution.<br>If the MAD blood volume exceeds local blood volume limits, do not collect biomarker samples. Then total MAD volume 9.35 mL represents 2.75 mL/kg for a 3.4 kg child.	course of the study. This blood volume represents 1.661.77 or 4.965.27 mL/kg for a 3.23.4-kg infant (~10th percentile for a 1-month oldsmallest subject enrolled (neonate) as of 15 August 2016) and is within the generally accepted range for blood draws for pediatric clinical research studies (Howie 2011). If the IDMC requires that the MAD treatment duration be 9 or 10 days, a maximum of 20.3 mL of blood will be drawn per subject, over the course of the study. This blood volume represents 6.34 mL/kg for a 3.2-kg infant. Actual blood volumes may vary at each institution.<br><br>If the MAD blood volume exceeds local blood volume limits, do not collect biomarker samples. Then the total MAD blood volume will be 9.35 mL represents (which is 2.92 mL/kg for a 3.2 kg child; 5-day dosing duration) or 11.3 mL (which is 3.53 mL/kg for a 3.2-kg child; 9- or 10-day dosing duration).<br><br>Added Table: Blood volume per body weight in a 3.2 kg infant	
8.3 PHARMACOKINETIC MEASUREMENTS		Nasal samples may be analyzed to determine exposures of ALS-008112 and ALS-008144 (and other metabolites, if applicable) if a sensitive method can be established.	If nasal samples may feasibly be evaluated for PK, this analysis will be performed as it defines exposures at the site of action of RSV (i.e., respiratory epithelium)
9.9 INTERIM ANALYSIS	A single interim analysis may be conducted during the conduct of the study if deemed necessary for reasons such as regulatory reporting	IA single interim analyses may be conducted during the conduct of the study if deemed necessary for reasons such as regulatory reporting requirements, Sponsor decision making, etc. An unblinded Sponsor team may review efficacy data on an ongoing basis. Study team	Unblinded viral kinetic data in a multiple dose setting may be informative in guiding dose selection and determining what parameters in the study may be best suited to be modified to maximize the potential for subjects to benefit from participation in this study. Unblinded

Section	Original Text in Version 5.0	Revised Text in Version 6.0	Reason for Change
	requirements, Sponsor decision making, etc.	members involved in study management will remain blinded to individual subject information and not participate in data reviews which could potentially unblind them.	Sponsor members may look at these data on an ongoing basis. If this team wishes to propose modifications to the study based on these data, they will ensure that their communication to the unblinded Sponsor team conducting the study does not unblind them. Similarly, they will only propose study modification which are within the constraints defined by this protocol.
**Table 12-3**<br>**Maximum Estimated Blood Volumes (MAD Part 2 PK Group A)***<br>**Table 12-4**<br>**Maximum Estimated Blood Volumes (MAD Part 2 PK Group B)***		Safety Visit ~~Day 5~~ Last day of dosing<br>Completion Visit 6 days after the last day of dosing<br>[&]If the IDMC requires that the treatment duration be 9 or 10 days, the additional blood volume collected on Day 5 would result in total blood volume collected of 16.85 mL + 3.45 mL=20.3 mL<br>7 hours post dose 2 assessment window increased to ±4 hours	Updated to reflect changes made in this protocol amendment.
