# Supplementary material for: Lumicitabine, an orally administered nucleoside analog, in infants hospitalized with respiratory syncytial virus (RSV) infection: Safety, efficacy, and pharmacokinetic results
Source: PLoS One. 2023 Jul 19;18(7):e0288271. doi: 10.1371/journal.pone.0288271 (PMC10355467; doi:10.1371/journal.pone.0288271)
Supplement: S2 Appendix — (PDF) [file pone.0288271.s002.pdf]

Janssen Research & Development *

Clinical Protocol

A Phase 2, Randomized, Double-blind, Placebo-controlled Study to Evaluate the Antiviral Activity, Clinical Outcomes, Safety, Tolerability, and Pharmacokinetics of Orally Administered Lumicitabine (JNJ-64041575) Regimens in Hospitalized Infants and Children Aged 28 Days to 36 Months Infected with Respiratory Syncytial Virus

Protocol 64041575RSV2004; Phase 2
AMENDMENT 1

Lumicitabine (JNJ-64041575, ALS-008176)

*Janssen Research & Development is a global organization that operates through different legal entities in various countries. Therefore, the legal entity acting as the sponsor for Janssen Research & Development studies may vary, such as, but not limited to Janssen Biotech, Inc.; Janssen Products, LP; Janssen Biologics, BV; Janssen-Cilag International NV; Janssen Pharmaceutica NV; Janssen, Inc; Janssen Sciences Ireland UC; or Janssen Research & Development, LLC. The term “sponsor” is used throughout the protocol to represent these various legal entities; the sponsor is identified on the Contact Information page that accompanies the protocol.

This study will be conducted under US Food & Drug Administration IND regulations (21 CFR Part 312).

EudraCT NUMBER: 2017-001862-56

Status: Approved
Date: 21 August 2017
Prepared by: Janssen Research & Development, a division of Janssen Pharmaceutica NV
EDMS number: EDMS-ERI-142047085, 3.0

GCP Compliance: This study will be conducted in compliance with Good Clinical Practice, and applicable regulatory requirements.

**Protocol History 64041575RSV2004**		
Document Type and *File Name*	Issued Date	Comments
Initial Clinical Protocol *64041575RSV2004_Protocol*	26 June 2017	-
Protocol Amendment 1 *64041575RSV2004_Protocol_Amend_1*	This document	For details, please refer to Section Amendment 1

TABLE OF CONTENTS

TABLE OF CONTENTS 3

LIST OF ATTACHMENTS 5

LIST OF IN-TEXT TABLES AND FIGURES 6

PROTOCOL AMENDMENT 7

SYNOPSIS 8

TIME AND EVENTS SCHEDULE: DURING HOSPITALIZATION (UP TO DISCHARGE) 20

TIME AND EVENTS SCHEDULE: AFTER HOSPITAL DISCHARGE 24

ABBREVIATIONS 27

DEFINITIONS OF TERMS 28

1. INTRODUCTION 29
1.1. Background 29
1.1.1. Respiratory Syncytial Virus 29
1.1.2. Information on the Product 30
1.2. Overall Rationale for the Study 37
1.3. Benefit-risk Evaluation 37
1.3.1. Known Benefits 37
1.3.2. Potential Benefits 37
1.3.3. Known Risks 37
1.3.4. Potential Risks 38
1.3.5. Overall Benefit/Risk Assessment 39

2. OBJECTIVES, ENDPOINTS, AND HYPOTHESIS 40
2.1. Objectives and Endpoints 40
2.1.1. Objectives 40
2.1.2. Endpoints 42
2.2. Hypothesis 43

3. STUDY DESIGN AND RATIONALE 44
3.1. Overview of Study Design 44
3.1.1. Option A 45
3.1.2. Option B 48
3.2. Study Design Rationale 52

4. SUBJECT POPULATION 56
4.1. Inclusion Criteria 56
4.2. Exclusion Criteria 57
4.3. Prohibitions and Restrictions 59

5. TREATMENT ALLOCATION AND BLINDING 60

6. DOSAGE AND ADMINISTRATION 61

7. TREATMENT COMPLIANCE 62

8. PRESTUDY AND CONCOMITANT THERAPY 62

9. STUDY EVALUATIONS 63
9.1. Study Procedures 63
9.1.1. Overview 63

9.1.2. Screening Phase ... 65
9.1.3. Double-blind Treatment Phase ... 65
9.1.4. Posttreatment Phase (Follow-up) ... 66
9.2. Efficacy Evaluations ... 66
9.3. Pharmacokinetics ... 68
9.3.1. Evaluations ... 68
9.3.2. Analytical Procedures ... 69
9.3.3. Pharmacokinetic Parameters ... 69
9.4. Pharmacokinetic/Pharmacodynamic Evaluations ... 69
9.5. Biomarkers ... 69
9.6. Medical Resource Utilization and Health Economics ... 70
9.7. Safety Evaluations ... 70
9.8. Specific Toxicities ... 74
9.9. Sample Collection and Handling ... 74

10. SUBJECT COMPLETION/DISCONTINUATION OF STUDY TREATMENT/ WITHDRAWAL FROM THE STUDY ... 75
10.1. Completion ... 75
10.2. Discontinuation of Study Treatment/Withdrawal From the Study ... 75
10.3. Withdrawal From the Use of Research Samples ... 77

11. STATISTICAL METHODS ... 77
11.1. Subject Information ... 78
11.2. Sample Size Determination ... 78
11.3. Efficacy Analyses ... 79
11.4. Pharmacokinetic Analyses ... 80
11.5. Biomarker Analyses ... 81
11.6. Pharmacokinetic/Pharmacodynamic Analyses ... 81
11.7. Medical Resource Utilization and Health Economics Analyses ... 81
11.8. Safety Analyses ... 81
11.9. Interim Analysis ... 83
11.10. Independent Data Monitoring Committee ... 83

12. ADVERSE EVENT REPORTING ... 84
12.1. Definitions ... 85
12.1.1. Adverse Event Definitions and Classifications ... 85
12.1.2. Attribution Definitions ... 86
12.1.3. Severity Criteria ... 87
12.2. Special Reporting Situations ... 87
12.3. Procedures ... 87
12.3.1. All Adverse Events ... 87
12.3.2. Serious Adverse Events ... 88
12.4. Contacting Sponsor Regarding Safety ... 89

13. PRODUCT QUALITY COMPLAINT HANDLING ... 90
13.1. Procedures ... 90
13.2. Contacting Sponsor Regarding Product Quality ... 90

14. STUDY DRUG INFORMATION ... 90
14.1. Physical Description of Study Drug(s) ... 90
14.2. Packaging ... 91
14.3. Labeling ... 91
14.4. Preparation, Handling, and Storage ... 91
14.5. Drug Accountability ... 91

15. STUDY-SPECIFIC MATERIALS ... 92

16. ETHICAL ASPECTS ... 92
16.1. Study-specific Design Considerations ... 92

16.2. Regulatory Ethics Compliance 93
16.2.1. Investigator Responsibilities 93
16.2.2. Independent Ethics Committee or Institutional Review Board 94
16.2.3. Informed Consent Form 95
16.2.4. Privacy of Personal Data 96
16.2.5. Long-term Retention of Samples for Additional Future Research 97
16.2.6. Country Selection 97

17. ADMINISTRATIVE REQUIREMENTS 97
17.1. Protocol Amendments 97
17.2. Regulatory Documentation 98
17.2.1. Regulatory Approval/Notification 98
17.2.2. Required Prestudy Documentation 98
17.3. Subject Identification, Enrollment, and Screening Logs 99
17.4. Source Documentation 99
17.5. Case Report Form Completion 100
17.6. Clinical Outcome Assessments 101
17.6.1. Investigator 101
17.6.2. Parent(s)/Caregiver(s) 101
17.7. Data Quality Assurance/Quality Control 102
17.8. Record Retention 102
17.9. Monitoring 102
17.10. Study Completion/Termination 103
17.10.1. Study Completion/End of Study 103
17.10.2. Study Termination 103
17.11. On-site Audits 104
17.12. Use of Information and Publication 104

REFERENCES 106

INVESTIGATOR AGREEMENT 137

LIST OF ATTACHMENTS

Attachment 1: Division of Microbiology and Infectious Diseases (DMID) Pediatric Toxicity Tables (November 2007) 107
Attachment 2: Cardiovascular Safety – Abnormalities 119
Attachment 3: Guideline for the Estimation of Glomerular Filtration Rate (GFR) Based on Serum Creatinine in Children 120
Attachment 4: Visit Schedule for Rash Management in Pediatric Subjects 121
Attachment 5: Pediatric RSV Electronic Severity and Outcome Rating System (PRESORS)v6 – Clinician's Electronic Clinical Outcome Assessment and Caregiver Diary 123
Attachment 6: General Guidelines for Measuring Vital Signs and SpO_2 135

LIST OF IN-TEXT TABLES AND FIGURES

TABLES

Table 1: Investigational Product and Its Metabolites 29
Table 2: NOAEL Doses of Lumicitabine and Exposure of JNJ-63549109 in Adult and Juvenile Rats and Dogs After Repeated Dosing of Lumicitabine 31
Table 3: Projected Plasma Exposures of JNJ-63549109 at the Proposed Doses 54

FIGURES

Figure 1: Study Design: Option Selection 45
Figure 2: Schematic Study Overview: Option A 47
Figure 3: Schematic Study Overview: Option B 51

PROTOCOL AMENDMENT

Amendment 1 (This document)

This amendment is considered to be substantial based on the criteria set forth in Article 10(a) of Directive 2001/20/EC of the European Parliament and the Council of the European Union.

The overall reason for the amendment: The overall reason for the amendment was to remove furosemide, ibuprofen, and trimethoprim/sulfametoxazole from the list of prohibited moderate/strong inhibitors of organic anion transporter (OAT) 3.

The table below gives an overview of the rationale for each change and all applicable sections.

**Rationale:** A clinical drug-drug interaction study (Study 64041575RSV1002) between probenecid (strong OAT3 inhibitor) and lumicitabine showed that probenecid increased the maximum concentration and area under the concentration-time curve (AUC) from the time of drug administration to the time of the last observation of JNJ-63549109 by 1.28-fold and 1.74-fold, respectively. Based on these data, together with pharmacokinetic (PK)/pharmacodynamic modeling data, furosemide, ibuprofen, and trimethoprim/sulfametoxazole were removed from the list of prohibited moderate/strong inhibitors of OAT3 as the expected increase on JNJ-63549109 AUC caused by these drugs was less than 26%, a very mild increase deemed acceptable based on expected exposures using popPK modeling and simulation.
8 PRESTUDY AND CONCOMITANT THERAPY
**Rationale:** No food intake information with regard to study drug administration will be collected for discharged subjects due to the minimal impact of food intake on trough levels (PK sample at Day 7).
SYNOPSIS<br>TIME AND EVENTS SCHEDULE: AFTER HOSPITAL DISCHARGE<br>6 DOSAGE AND ADMINISTRATION<br>9.1.3 Double-blind Treatment Phase
**Rationale:** Only leftover samples from nasal swabs and PK testing, but not from laboratory safety testing (hematology, biochemistry and urinalysis) may be used for exploratory biomarker analyses.
SYNOPSIS<br>TIME AND EVENTS SCHEDULE: DURING HOSPITALIZATION (up to discharge)<br>TIME AND EVENTS SCHEDULE: AFTER HOSPITAL DISCHARGE<br>3.2 Study Design Rationale<br>9.5 Biomarkers
**Rationale:** Minor editorial changes, clarifications, and corrections were made.
Throughout the protocol

SYNOPSIS

A Phase 2, Randomized, Double-blind, Placebo-controlled Study to Evaluate the Antiviral Activity, Clinical Outcomes, Safety, Tolerability, and Pharmacokinetics of Orally Administered Lumicitabine (JNJ-64041575) Regimens in Hospitalized Infants and Children Aged 28 Days to 36 Months Infected with Respiratory Syncytial Virus

Lumicitabine (also known as JNJ-64041575 or ALS-008176) is a 3',5'-bisisobutyrate prodrug, which is rapidly converted to the cytidine nucleoside analog, JNJ-63549109 (also known as ALS-008112). Inside cells, JNJ-63549109 is subsequently converted to its nucleoside triphosphate (NTP), JNJ-65409136 (also known as ALS-008136). This NTP is a potent and selective inhibitor of respiratory syncytial virus (RSV) ribonucleic acid (RNA) polymerase activity via a classic chain termination mechanism. JNJ-64167896 (also known as ALS-008144), the uridine metabolite of JNJ-63549109, is the inactive major metabolite detected in systemic circulation.

Investigational Product and Its Metabolites

Compound Name/Number	Description
Lumicitabine (JNJ-64041575, ALS-008176)	3',5'-bisisobutyrate prodrug of JNJ-63549109
JNJ-63549109 (ALS-008112)	parent nucleoside, major metabolite of lumicitabine
JNJ-64412309 (ALS-008206)	3'-isobutyrate monoester of JNJ-63549109, minor metabolite
JNJ-64412296 (ALS-008207)	5'-isobutyrate monoester of JNJ-63549109, minor metabolite
JNJ-64167896 (ALS-008144)	uridine metabolite of JNJ-63549109, major metabolite
JNJ-65409136 (ALS-008136)	5'-triphosphate of JNJ-63549109 (NTP), intracellular metabolite, active
JNJ-65409123 (ALS-008137)	5'-monophosphate of JNJ-63549109, intracellular metabolite

Abbreviation: NTP: nucleoside triphosphate.

OBJECTIVES, ENDPOINTS, AND HYPOTHESIS

Objectives

Primary Objective

The primary objective is to determine in hospitalized infants and children who are infected with RSV the dose-response relationship of multiple regimens of lumicitabine on antiviral activity based on nasal RSV shedding using quantitative real-time reverse transcriptase-polymerase chain reaction (qRT-PCR).

Secondary Objectives

The secondary objectives are to determine in hospitalized infants and children who are infected with RSV:

- The safety and tolerability of lumicitabine.
- The pharmacokinetics (PK) of JNJ-63549109 in whole blood.
- The impact of lumicitabine on the clinical course of RSV infection.
- The impact of lumicitabine on the duration and severity of signs and symptoms of RSV infection as assessed by the Pediatric RSV Electronic Severity and Outcome Rating System (PRESORS) questionnaire completed by the clinician in the electronic clinical outcome assessment (eCOA) device.
- The impact of lumicitabine on the time to undetectable nasal RSV viral load.
- The impact of lumicitabine on the emergence of RSV strains with resistance-associated mutations.

- The relationship between the PK of JNJ−63549109 and the pharmacodynamics (PD; antiviral activity, clinical symptoms, and selected safety parameters) after single (loading dose [LD]) and repeated oral dosing (maintenance dose [MD]) of lumicitabine.
- The acceptability and palatability of the lumicitabine formulation.

Exploratory Objectives

The exploratory objectives are to evaluate in hospitalized infants and children who are infected with RSV:

- The relationship between viral load and clinical outcome, including the relationship between RSV RNA levels and:
    - Requirement for and duration of supplemental oxygen.
    - Time to hospital discharge or readiness for hospital discharge, with readiness for discharge evaluated by the investigator.
    - Time to clinical stability defined as the time from initiation of study treatment until the time at which the following criteria are met: return to pre-RSV infection status (hereafter referred to as “normalization”) of blood oxygen level (without additional requirement of supplemental oxygen compared with pre-RSV infection status), normalization of oral feeding, normalization of respiratory rate, and normalization of heart rate.
- The relationship between viral load and duration and severity of signs and symptoms of RSV infection as assessed by the PRESORS questionnaire completed by the clinician in the eCOA device.
- The impact of the baseline viral subtype and genotype on the antiviral activity.
- The impact of lumicitabine on the duration and severity of signs and symptoms of RSV infection as assessed by the PRESORS questionnaire completed by the subject’s parent(s)/caregiver(s) in the eCOA device.
- The relationship between the clinician eCOA and parent(s)/caregiver(s) eCOA responses.
- Medical resource utilization.
- The impact of lumicitabine on the infectious viral load using a quantitative culture of RSV (plaque assay) may be performed in a central laboratory at timepoints selected by the virologist.
- The comparison of the RSV viral loads measured in the mid-turbinate nasal swabs and endotracheal samples from intubated subjects.

Endpoints

Primary Endpoint

The primary endpoint is RSV viral load (measured by qRT-PCR in the mid-turbinate nasal swab specimens) area under the concentration-time curve (AUC) from immediately prior to first dose of study drug (baseline) until Day 7.

Secondary Endpoints

The secondary endpoints are:

- Safety/tolerability including adverse events (AEs), physical examinations, vital signs/peripheral capillary oxygen saturation (SpO_2), electrocardiogram (ECG), and clinical laboratory results.
- PK parameters of JNJ-63549109.

- RSV clinical course endpoints:
    - Length of hospital stay from admission to discharge and to readiness for discharge and from study treatment initiation to discharge and to readiness for discharge, with readiness for discharge evaluated by the investigator.
    - Requirement for and duration of intensive care unit (ICU) stay.
    - Requirement for and duration of oxygen supplementation/noninvasive mechanical ventilation support (eg, nasal cannula, face mask, continuous positive airway pressure) and/or invasive mechanical ventilation support (eg, endotracheal-mechanical ventilation or mechanical ventilation via tracheostomy) above pre-RSV infection status.
    - Time to no longer requiring supplemental oxygen above pre-RSV infection status.
    - Time to clinical stability defined as the time from initiation of study treatment until the time at which the following criteria are met: return to pre-RSV infection status (hereafter referred to as "normalization") of blood oxygen level (ie, without additional requirement of supplemental oxygen compared with pre-RSV infection status), normalization of oral feeding, normalization of respiratory rate, and normalization of heart rate.
    - Time from initiation of study treatment until SpO_2 ≥93% on room air among subjects who were not on supplemental oxygen prior to the onset of respiratory symptoms.
    - Time to respiratory rate, SpO_2, and body temperature return to pre-RSV infection status.
    - Incidence of acute otitis media (defined by the investigator).
- The duration and severity of signs and symptoms of RSV infection as assessed by the PRESORS questionnaire completed by the clinician in the eCOA device.
- RSV viral load as measured by qRT-PCR in the mid-turbinate nasal swab specimens, which will be used to determine the following:
    - RSV viral load over time.
    - Peak viral load, time to peak viral load, rate of decline of viral load, and time to RSV RNA being undetectable.
    - Proportion of subjects with undetectable RSV viral load at each timepoint.
    - RSV viral load AUC from immediately prior to first dose of study drug (baseline) until Day 10 and until Day 14.
    - RSV viral load AUC in subjects assigned to a longer dosing duration, if dosing duration is increased by the Independent Data Monitoring Committee (IDMC), from baseline until 1 day (+2 days) after the last dose of study drug.
- Sequence changes (postbaseline) in the RSV polymerase L-gene and other regions (if warranted) of the RSV genome compared with baseline sequences.
- Acceptability and palatability of the lumicitabine formulation as assessed by the parent(s)/caregiver(s) eCOA.

Exploratory Endpoints

The exploratory endpoints of this study are:

- The duration and severity of signs and symptoms of RSV infection as assessed by the PRESORS questionnaire completed by the parent(s)/caregiver(s) in the eCOA device.
- Number of hours the subject's parent(s)/caregiver(s) missed from usual activities.

- Medical resource utilization.
- RSV infectious viral load as measured using a quantitative viral culture (plaque assay).
- RSV viral load as measured by qRT-PCR of mid-turbinate nasal swabs and endotracheal samples in intubated subjects.

Hypothesis

The primary hypothesis of this study is that there is a positive dose-response relationship of active treatment on the average RSV viral load AUC over 7 days, meaning that either the average AUC on the pooled active treatments is lower than on placebo, or the average AUC on the high dose is lower than the average AUC on placebo, using multiple contrast testing.

OVERVIEW OF STUDY DESIGN

This is a randomized, double-blind, placebo-controlled, multicenter study of lumicitabine in hospitalized infants and children who are infected with RSV.

The selection of the design of this study (Option A or Option B) will be based on the number of subjects randomized and on the IDMC review of the data from the currently ongoing Study ALS-8176-503 of the subjects treated with 40 mg/kg LD/20 mg/kg MD lumicitabine dose regimen:

- **Option A:** This study design will be chosen if ≥9 subjects are randomized to the 40 mg/kg LD/20 mg/kg MD lumicitabine regimen in Study ALS-8176-503 (≥12 subjects in total) and if the IDMC recommends using the 60 mg/kg LD/40 mg/kg MD lumicitabine regimen based on their review of the data from Study ALS-8176-503. Based on the data available at the time of protocol writing, 60 mg/kg LD/40 mg/kg MD is the most likely dose regimen the IDMC will recommend. Option A will use the 40 mg/kg LD/20 mg/kg MD as the low lumicitabine dose and the 60 mg/kg LD/40 mg/kg MD (based on IDMC recommendation) as the high lumicitabine dose.
- **Option B:** This study design will be chosen if <9 subjects are randomized to the 40 mg/kg LD/20 mg/kg MD lumicitabine regimen in Study ALS-8176-503 (<12 subjects in total) and thus no IDMC-recommended dose will be available at the start of the study. Option B will use the 40 mg/kg LD/20 mg/kg MD as the low lumicitabine dose. This study design option may also be selected once ≥9 subjects are randomized to the 40 mg/kg LD/20 mg/kg MD lumicitabine regimen in Study ALS-8176-503 (≥12 subjects in total) if:
    - Sponsor determines that additional doses should be evaluated.
    - IDMC-recommended lumicitabine dose is lower than the 60 mg/kg LD/40 mg/kg MD lumicitabine regimen.

Option A

Hospitalized infants and children who are infected with RSV will be randomly assigned to a treatment regimen in this study, with 60 subjects planned per regimen. At least 120 subjects are planned to be enrolled up to a maximum of 180 subjects. An effort will be made to enroll at least 90 subjects aged between 28 days and 24 months and at least 12 subjects aged between 24 and 36 months. The number of subjects with comorbidities will be limited to 20%; additional comorbid subjects aged between 24 and 36 months may be allowed to enroll if the 20% threshold is reached before 12 subjects aged between 24 and 36 months are enrolled into the study.

Subjects will be randomized in a 1:1:1 ratio to Regimen A, B, or C:

- Regimen A (low-dose lumicitabine): a single 40 mg/kg LD (Dose 1) followed by nine 20 mg/kg MDs (Doses 2 to 10) of lumicitabine, administered twice daily.

- Regimen B (high-dose lumicitabine): a single 60 mg/kg LD (Dose 1) followed by nine 40 mg/kg MDs (Doses 2 to 10) of lumicitabine, administered twice daily.
- Regimen C (placebo):those randomized to a placebo regimen are subsequently randomized in a 1:1 ratio (to match volumes) to either:
    - A single 40 mg/kg placebo LD (Dose 1) followed by nine 20 mg/kg MDs (Doses 2 to 10), administered twice daily.
    - A single 60 mg/kg placebo LD (Dose 1) followed by nine 40 mg/kg placebo MDs (Doses 2 to 10), administered twice daily.

Randomization will be stratified by duration of RSV symptoms from onset till time of randomization (≤3 days; >3 days to ≤5 days), and by presence or absence of at least 1 comorbid condition for severe RSV disease (prematurity at birth [subject's gestational age was <37 weeks; for infants <1 year old at randomization], bronchopulmonary dysplasia, congenital heart disease, other congenital diseases, Down syndrome, neuromuscular impairment, or cystic fibrosis). Recruitment of subjects with comorbidities will not be allowed if the data of the highest dose regimen of lumicitabine have not yet been reviewed by the IDMC.

The study will be conducted in 3 phases: a screening phase, a treatment phase from Day 1 to Day 5/6 (depending on the timing of the LD), and a follow-up phase ending 28 days post randomization. Screening for eligible subjects will be performed as soon as possible following admission to the emergency room (ER) or hospitalization, such that subjects are randomized within 5 days of RSV symptom onset. Special attention will be paid to determine the duration of RSV symptoms from onset till time of randomization as accurately as possible. Subjects will have assessments completed during hospitalization and at the Day 7, Day 10, Day 14, and Day 28 visits. For hospitalized subjects, additional assessments are done compared with subjects who have already been discharged. The duration of the subject's participation will be approximately 28 days, screening period not included. The study will be considered completed with the last visit for the last subject participating in the study.

An unblinded IDMC will be commissioned for this study and a Sponsor Committee will be established. The IDMC will review the safety and PK data initially once the first 12 subjects and 30 subjects have completed treatment (Day 5/6) to assess if enrollment of additional subjects may safely continue in each arm. After at least 9 subjects have completed treatment with the 60 mg/kg LD/40 mg/kg MD lumicitabine regimen, the IDMC may recommend that sparse PK sampling be used for the other subjects. Subject recruitment and enrollment may continue while the data are being reviewed. The IDMC will also assess PK, safety, and efficacy data during the conduct of the study. Based on the review of this data, changes to randomization ratios, enrollment based on time from onset of RSV symptoms (≤3 days and >3 to ≤5 days) till randomization, dose regimen adjustments, or an increase in treatment duration to 10 days may be implemented.

Up to 2 formal interim analyses may be performed during the study. The first interim analysis will be performed once 36 subjects with ≤3 days of RSV symptoms have completed treatment (Day 5/6) or once 90 subjects with >3 to ≤5 days of RSV symptoms have been enrolled and have completed treatment. The following situations will be considered based on the results at this stage: that the study is considered futile; that superiority can be concluded; that subjects with >3 to ≤5 days of RSV symptoms at randomization may not achieve benefit (which may also be limited to ≤4 days of onset to symptoms); or that the study can continue unchanged. The treatment regimen may be modified after the first interim analysis, including increasing the treatment duration up to 10 days. After this analysis, randomization to 1 of the lumicitabine doses may be discontinued, the randomization ratio may change, and the study may not continue in subjects with >3 to ≤5 days of RSV symptoms at randomization. The second interim analysis may be performed at the discretion of the sponsor once at least 120 subjects have completed treatment (Day 5/6) (or at least 60 subjects with ≤3 days of RSV symptoms). The following situations will be considered based on the results at this stage: that the study is considered futile; that superiority can be

concluded; or that the study can continue unchanged. Based on the recommendations of the IDMC following these interim analyses/reviews of PK, efficacy, and safety data, changes to randomization ratios in the treatment arms or an increase in treatment duration to 10 days may be implemented.

Option B

Hospitalized infants or children who are infected with RSV will be sequentially enrolled into different cohorts and will be randomized to receive either lumicitabine or placebo. At least 120 subjects are planned to be enrolled up to a maximum of 180 subjects. An effort will be made to enroll at least 90 subjects aged between 28 days and 24 months and at least 12 subjects aged between 24 and 36 months. The number of subjects with comorbidities will be limited to 20%; additional comorbid subjects aged between 24 and 36 months may be allowed to enroll if the 20% threshold is reached before 12 subjects aged between 24 and 36 months are enrolled into the study.

Subjects will be enrolled into Cohort 1 and randomized in a 2:1 ratio to lumicitabine Regimen 1 or placebo:

- Lumicitabine Regimen 1: a single 40 mg/kg LD followed by nine 20 mg/kg MDs of lumicitabine, administered twice daily.
- Placebo: a single 40 mg/kg LD followed by nine 20 mg/kg MDs of placebo, administered twice daily.

An IDMC will review the safety and PK data once at least 9 subjects in Cohort 1 have completed treatment (Day 5/6) with the 40 mg/kg LD/20 mg/kg MD lumicitabine regimen (including subjects recruited in Study ALS-8176-503) to assess if recruitment to a new treatment regimen (Cohort 2) may safely start. Subject recruitment and enrollment for Cohort 1 may continue while the data are being reviewed.

The sponsor may skip recruitment into Cohort 1 (ie, no subjects would be enrolled into Cohort 1) and recruit subjects directly into Cohort 2 if ≥9 subjects have been treated with the 40 mg/kg LD/20 mg/kg MD lumicitabine regimen in Study ALS-8176-503 (≥12 subjects in total) and if those data have been reviewed by the IDMC.

Subjects will be enrolled into Cohort 2 and randomized in a 1:1:1 ratio to lumicitabine Regimen 1, lumicitabine Regimen 2, or placebo:

- Lumicitabine Regimen 1: a single 40 mg/kg LD followed by nine 20 mg/kg MDs of lumicitabine, administered twice daily.
- Lumicitabine Regimen 2: a single LD followed by 9 MDs of lumicitabine Regimen 2, administered twice daily. The IDMC will provide a dose recommendation for Regimen 2.
- Placebo: those randomized to a placebo regimen are subsequently randomized in a 1:1 ratio (to match volumes) to either:
    - A single 40 mg/kg placebo LD (Dose 1) followed by nine 20 mg/kg MDs (Doses 2 to 10) of placebo, administered twice daily.
    - A single placebo LD (Dose 1) followed by nine MDs (Doses 2 to 10) of placebo matching Regimen 2, administered twice daily.

An IDMC will review the safety and PK data from Cohort 2 once at least 9 subjects in Cohort 2 have completed treatment (Day 5/6) with the active lumicitabine Regimen 2. Subject recruitment and enrollment for Cohort 2 may continue while the data are being reviewed.

A third dose may be evaluated, upon IDMC recommendation, based on the emerging safety and PK profile. Subjects will be enrolled into Cohort 3 and randomized in a 1:3:2 ratio to lumicitabine Regimen 1, lumicitabine Regimen 3, or placebo:

- Lumicitabine Regimen 1: a single 40 mg/kg LD followed by nine 20 mg/kg MDs of lumicitabine, administered twice daily.
- Lumicitabine Regimen 3: a single LD followed by 9 MDs of lumicitabine Regimen 3, administered twice daily. The IDMC will provide a dose recommendation for the lumicitabine Regimen 3.
- Placebo: those randomized to a placebo regimen are subsequently randomized in a 1:3 ratio (to match volumes) to either:
    - A single 40 mg/kg placebo LD (Dose 1) followed by nine 20 mg/kg MDs (Doses 2 to 10) of placebo, administered twice daily.
    - A single placebo LD (Dose 1) followed by nine MDs (Doses 2 to 10) of placebo matching Regimen 3, administered twice daily.

An IDMC will review safety and PK data from Cohort 3 once 9 subjects in that cohort have completed treatment (Day 5/6) with the active lumicitabine Regimen 3, at which stage the IDMC will select either lumicitabine Regimen 2 or lumicitabine Regimen 3 to be used with placebo and lumicitabine Regimen 1 for the remainder of the study. Depending on the selected doses, other randomization schemes may be selected for the remainder of the study. Subject recruitment and enrollment for Cohort 3 may continue while the data are being reviewed. Subjects will continue to be enrolled and treated in Cohort 2 up to a maximum of 180 subjects if a third dose regimen is not evaluated.

The sponsor/IDMC may recommend that the cohort regimens be adjusted or removed, given the active nature of the development program and the ongoing assessment of all the available data.

Randomization will be stratified by duration of RSV symptoms from onset till time of randomization (≤3 days; >3 days to ≤5 days), and by presence or absence of at least 1 comorbid condition for severe RSV disease (prematurity at birth [subject's gestational age was <37 weeks; for infants <1 year old at randomization], bronchopulmonary dysplasia, congenital heart disease, other congenital diseases, Down syndrome, neuromuscular impairment, or cystic fibrosis). Recruitment of subjects with comorbidities will not be allowed if the data of the highest dose regimen of lumicitabine have not yet been reviewed by the IDMC.

The study will be conducted in 3 phases: a screening phase, a treatment phase from Day 1 to Day 5/6 (depending on the timing of the LD), and a follow-up phase ending 28 days postrandomization. Screening for eligible subjects will be performed as soon as possible following admission to the ER or hospitalization, such that subjects are randomized within 5 days of RSV symptom onset. Special attention will be paid to determine the duration of RSV symptoms from onset till time of randomization as accurately as possible. Subjects will have assessments completed during hospitalization and at the Day 7, Day 10, Day 14, and Day 28 visits. For hospitalized subjects, additional assessments are done compared with subjects who have already been discharged. The duration of the subject's participation will be approximately 28 days, screening period not included. The study will be considered completed with the last visit for the last subject participating in the study.

Up to 2 formal interim analyses will be performed during the study. The first interim analysis will be performed once 36 subjects with ≤3 days of RSV symptoms have completed treatment or once 90 subjects with >3 to ≤5 days of RSV symptoms have been enrolled and have completed treatment. The following situations will be considered based on the data analysis at this stage: that the study is considered futile; that superiority can be concluded; that subjects with >3 to ≤5 days of RSV symptoms at randomization may not achieve benefit (which may also be limited to ≤4 days of onset to symptoms); or that the study can continue unchanged. The treatment regimen may be modified after the first interim

analysis, including increasing the treatment duration up to 10 days. After this analysis, randomization to 1 of the lumicitabine doses may be discontinued, the randomization ratios may change, and the study may not continue in subjects with >3 to ≤5 days of RSV symptoms at randomization. The second interim analysis may be performed at the discretion of the sponsor once at least 120 subjects have completed treatment (Day 5/6) (or 60 subjects with ≤3 days of RSV symptoms). The following situations will be considered based on the data analysis at this stage: that the study is considered futile; that superiority can be concluded; or that the study can continue unchanged. Based on the recommendations of the IDMC following these interim analyses/reviews of PK, efficacy, and safety data, changes to randomization ratios in the treatment arms or an increase in treatment duration to 10 days may be implemented.

SUBJECT POPULATION

Screening for eligible subjects will be performed as soon as possible following admission to the ER or hospitalization, such that subjects are randomized within 5 days of RSV symptom onset. Special attention will be paid to determine the duration of RSV symptoms from onset till time of randomization as accurately as possible. Male or female subjects must be aged ≥28 days to ≤36 months and diagnosed with RSV infection based on a polymerase chain reaction (PCR)-based diagnostic assay (with or without coinfection with another respiratory pathogen) who have been (or will be) admitted to the hospital and whose parents have signed informed consent will be enrolled. Subjects who were admitted to the hospital for another reason but develop an acute respiratory illness while being hospitalized are also eligible for screening.

Subjects who are admitted to hospital due to RSV infection, including those who are otherwise healthy and those with underlying comorbidities (prematurity at birth [subject's gestational age was <37 weeks; for infants <1 year old at randomization], bronchopulmonary dysplasia, congenital heart disease, other congenital diseases, Down syndrome, neuromuscular impairment, or cystic fibrosis) can participate. However, enrollment of subjects with comorbidities in the Option A design can only start after at least 9 subjects have completed treatment with the 60 mg/kg LD/40 mg/kg MD lumicitabine regimen, after their safety and PK data have been reviewed by the IDMC, and after the IDMC has recommended that the recruitment of subjects with underlying comorbidities may start. Enrollment of subjects with comorbidities in the Option B design can only start once at least 9 subjects in Cohort 2 have completed treatment with the active lumicitabine Regimen 2 and after their safety and PK data have been reviewed by the IDMC. If subjects are enrolled into Cohort 3, then enrollment of subjects with comorbidities can only start once at least 9 subjects in this cohort have completed treatment with the active lumicitabine Regimen 3 and after their safety and PK data have been reviewed by the IDMC.

DOSAGE AND ADMINISTRATION

The study drug will be provided as a powder for suspension for oral administration. Study drug administration should start as soon as possible, but no later than 4 hours after randomization in order to maximize the opportunity for the compound to inhibit viral replication and potentially improve outcomes.

Subjects will be dosed with a single LD followed by 9 MDs twice daily (minimum 8 hours and maximum 16 hours apart with no more than 2 doses per calendar day) during Day 1 to Day 5/6 (depending on the timing of the LD).

Administration of each dose should occur at approximately the same time each day and after the once-daily collection of the mid-turbinate nasal swab. Lumicitabine can be administered without regard to food. The date and time of dose administration, the date and time of food intake (limited to the food ingested within 30 minutes before or after study drug administration), and the type of food will be recorded in the electronic case report form (eCRF) for hospitalized subjects.

Lumicitabine and placebo will be supplied as a powder in amber glass bottles containing 1,891 mg lumicitabine or placebo a child-resistant cap. The powder should be suspended with solvent prior to oral administration, providing a 60 mg/mL suspension, under the supervision of the investigator or a qualified

member of the study-site personnel, or by a hospital/clinic pharmacist. Instructions for preparation of weight-based study formulations are provided in the pharmacy manual.

EFFICACY EVALUATIONS

Summary of Efficacy Evaluations

Evaluation	Purpose
**Viral Load Determination**	Evaluation of antiviral activity of lumicitabine; RSV RNA viral load will be measured in mid-turbinate nasal swabs (obtained from non-intubated subjects) or in mid-turbinate nasal swabs and endotracheal samples (obtained from intubated subjects or via suction through tracheostomy or other sampling methods) using qRT-PCR performed at the central laboratory.
**Viral Resistance**	To identify preexisting sequence polymorphisms and to characterize emerging RSV variants: viral sequencing analysis will be performed by sequencing the polymerase L-gene and other regions of the RSV genome (if warranted) of the RSV genome in samples taken before treatment (at baseline), during treatment, and posttreatment.
**Clinical Course of RSV Evaluation**	Evaluations of the clinical course of RSV infection including, but not limited to:<br>• Requirement for and duration of oxygen supplementation/noninvasive mechanical ventilation support (eg, nasal cannula, face mask, continuous positive airway pressure) and/or invasive mechanical ventilation support (eg, endotracheal-mechanical ventilation or mechanical ventilation via tracheostomy).<br>• Body weight.<br>• Respiratory rate, heart rate, systolic blood pressure, diastolic blood pressure, SpO_2, and body temperature (method should be noted in the eCRF).<br>• Level of hospital care (eg, ICU, translational care unit, ward floor).<br>• Duration of hospitalization.<br>• Requirement for hydration and feeding by IV catheter/nasogastric tube.
**Clinician-reported Evaluation of Clinical Outcomes**	• Clinician evaluation of the duration and severity of signs and symptoms of RSV infection (clinician eCOA; PRESORS).
**Parent(s)/Caregiver(s)-reported Evaluation of Clinical Outcomes**	• Parent(s)/caregiver(s) evaluation of the severity of signs and symptoms of RSV infection and the impact on the subject's functioning (parent[s]/caregiver[s] eCOA; PRESORS) which will be performed if possible by the same parent/caregiver at each timepoint.

Abbreviations: eCOA: electronic clinical outcome assessment; eCRF: electronic case report form; ICU: intensive care unit; IV: intravenous; PRESORS: Pediatric RSV Electronic Severity and Outcome Rating System; qRT-PCR: quantitative real-time reverse transcriptase-polymerase chain reaction; RSV: respiratory syncytial virus; RNA: ribonucleic acid; SpO_2: peripheral capillary oxygen saturation.

PHARMACOKINETIC EVALUATIONS

Whole blood samples will be used to determine the concentrations of JNJ-63549109 and JNJ-64167896. Concentrations of JNJ-63549109 will be used to estimate the PK parameters of JNJ-63549109 using a population-derived PK (popPK) model.

PHARMACOKINETIC/PHARMACODYNAMIC EVALUATIONS

The relationship between the PK of JNJ-63549109 and PD (antiviral activity, clinical outcomes, and selected safety parameters) after single and repeated oral dosing of lumicitabine will be evaluated.

BIOMARKER EVALUATIONS

Leftover mid-turbinate nasal swabs and blood samples collected for PK testing may be used for exploratory biomarker analyses (eg, proteins including cytokines), on the premise that these markers may play a role in the treatment response, safety of lumicitabine, or the status and change of the RSV-related disease. Analyses of biomarkers may be conducted at the sponsor’s discretion and reported separately from this study.

No human DNA analyses will take place on these samples.

MEDICAL RESOURCE UTILIZATION AND HEALTH ECONOMICS

The data collected may be used to conduct exploratory economic analyses and will include:

- Number and duration of medical care encounters, including surgeries and other selected procedures (inpatient and outpatient).
- Duration of hospitalization (total days length of stay, including duration by wards [eg, ICU]).
- Outpatient medical encounters and treatments (including physician or ER visits, tests and procedures, and medications).
- The number of subjects (proportion) who started antibiotic use after the first dose of the study drug up to the Day 28 follow-up visit.
- Requirement for, and duration of, hospital readmission for respiratory reasons from discharge up to the Day 28 follow-up visit.

SAFETY EVALUATIONS

Safety and tolerability will be evaluated throughout the study from signing of the informed consent form onwards until the last study-related activity (end of study/early withdrawal).

An IDMC will be established to monitor the safety of subjects and will review data in an unblinded manner on a regular basis to ensure the continuing safety of the subjects enrolled in this study. The IDMC will review the data and make recommendations to the Sponsor Committee, which will be responsible for identifying appropriate actions based on the recommendations of the IDMC. The IDMC will review the safety data:

- Option A: once the first 12 subjects and 30 subjects have completed treatment.
- Option B: once at least 9 subjects in each cohort have completed treatment with the highest active lumicitabine regimen available in their respective cohort.

The IDMC will also assess PK, safety, and efficacy data during the conduct of the study.

Any clinically relevant changes occurring during the study must be recorded on the AE section of the electronic case report form.

Any clinically significant abnormalities persisting at the end of the study/early withdrawal will be followed by the investigator until resolution or until a clinically stable endpoint is reached.

Safety assessments will be based on medical review of AE reports and the results of vital sign measurements, ECGs, physical examinations, clinical laboratory tests, and other safety evaluations.

STATISTICAL METHODS

The primary hypothesis is a positive dose-response relationship of active treatment on the average RSV viral load AUC over 7 days, meaning that either the average AUC on the pooled active treatments is lower than on placebo, or the average AUC on the high active dose is lower than the average AUC on placebo, using multiple contrast testing. On high dose, the hypothesis of the lumicitabine antiviral activity assumes a viral load reduction on the AUC of the log viral load over 7 days of at least 30% when compared to placebo in subjects with onset of symptoms ≤3 days and of at least 20% in subjects with onset of symptoms >3 days. The antiviral activity on low dose is assumed to be 80% when compared to the antiviral activity on the high dose. The positive dose-response relationship assumes that dose regimens with higher exposure with respect to MD will have at least an equal or better effect on viral load. Therefore 2 contrasts will be tested at each of the interim analysis points and final analysis: a contrast with no difference between the 2 active regimens tested against placebo (high equal to low, better than placebo) and a contrast with a positive 'linear' dose-response relationship (high better than low, low better than placebo) with respect to active regimens (where the effect of the low dose is exactly in between that of placebo and the high dose regimen). With respect to multiple contrast testing, multiplicity of the 2 contrasts will be controlled at the prespecified (interim) alpha level by calculating adjusted p-values from the simulated distribution of the maximum or maximum absolute value of a multivariate t random vector (ie, using the correlation between the contrasts to optimally control for alpha). Based on the observed data of other in-house studies, the estimated SD is assumed to be approximately 34% of the AUC for subjects treated with placebo and the SD is assumed to be equal in each of the treatment regimens. The overall (family-wise) type 1 error rate of 2.5% (1-sided) will be adjusted for multiple testing due to formal interim analyses using a Pocock alpha-spending function with 3 sequential tests (2 interim, 1 final). Based on 10,000 simulations and using a general linear model with treatment regimen as fixed factors, and under the assumption that 40% of the recruited subjects have onset of symptoms ≤3 days, a sample size of 180 subjects randomized in a 1:1:1 ratio will offer approximately 97% power to detect a positive dose-response relationship using multiple contrast testing. With the second interim analysis planned after at least 120 subjects, there will be at least 80% power to detect a positive dose-response relationship at the time of this analysis.

The primary endpoint in this study is RSV RNA $\log_{10}$ viral load (measured by qRT-PCR assay in the mid-turbinate nasal swab specimens) AUC immediately prior to first dose of study drug (baseline) over 7 days. Mean $\log_{10}$ viral load values over time will be analyzed using a restricted maximum likelihood-based repeated measures approach. Analyses will include the fixed, categorical effects of treatment, strata, visit, and treatment-by-visit interaction, as well as the continuous, fixed covariates of baseline $\log_{10}$ viral load and baseline $\log_{10}$ viral load by visit interaction. An unstructured (co)variance structure will be used to model the within subject errors over time. The Kenward Roger method will be used to approximate the degrees of freedom. The difference in the AUCs for active versus placebo will be derived using appropriate contrasts deriving least square mean differences, including the 95% 2-sided confidence intervals. For inferential purposes, p-values will be compared with significance levels controlling for the (family-wise) type 1 error rate.

For differences between treatment groups on qRT-PCR, the same methods as for the primary endpoint will be used, using appropriate contrasts to estimate treatment differences between the various treatment groups by treatment day using nominal 95% confidence intervals. The time to virus not detected is defined as the time from initiation of study treatment until the time at which it is observed that the virus is undetectable in an assessment and after which time no virus positive assessment follows. The time to clinical stability is defined as the time from initiation of study treatment until the time at which the

following criteria are met: return to pre-RSV infection status (hereafter referred to as "normalization") of blood oxygen level (ie, no additional requirement of supplemental oxygen compared with pre-RSV infection status), normalization of oral feeding, normalization of respiratory rate, and normalization of heart rate. Additionally, statistical modeling will be performed on the secondary endpoint of length of hospital stay, defined as the time from treatment initiation to hospital discharge in hours. Parent/caregiver-reported and clinician-reported eCOA (PRESORS) will be descriptively summarized by treatment group and compared across treatment groups.

Population PK analysis of concentration-time data of JNJ-63549109 will be performed using nonlinear mixed-effects modeling. Subjects will be excluded from the PK analysis if their data do not allow for accurate assessment of the PK.

Statistical approaches to explore correlations between clinical outcome and biomarkers vary and depend on the different data types of the applied technology platforms, as well as on the extent of observed differences among study subjects. Analyses may be conducted at the sponsor's discretion and reported separately from this study. Selected efficacy and safety parameters will be subject to PK/PD analysis. JNJ-63549109 exposures will be evaluated as an independent variable, with selected efficacy and selected safety parameters considered as dependent variables. Various approaches, including graphical analysis, linear, nonlinear, and logistic regression methods may be utilized.

Medical resource utilization and health economics will be descriptively summarized by treatment regimen and compared across treatment regimens.

Safety data will be presented descriptively. No statistical testing of safety data is planned. For safety, baseline is defined as the last assessment prior to the first intake of study drug.

TIME AND EVENTS SCHEDULE: DURING HOSPITALIZATION (UP TO DISCHARGE)

**Phase**	**Screening**	**Treatment Phase**			**Follow-up Phase** [a]						
**Day**	**0**	**1**	**2**	**3 to 6** [b]	**7** [c] **(+2 days)**	**8 to 9**	**10 (+2days)**	**11 to 13**	**14 (+2 days)**	**15 to 27**	**28 (±2 days) EoS**
**Study Procedure** [d]											
Screening/Administrative											
Informed consent form	X										
Demographics	X										
Medical history	X										
Inclusion/exclusion criteria [e]	X										
Body weight	X				X		X		X		X
Body length and head circumference	X										X
Study Drug Administration [f]											
Randomization		X									
Dispense/administer study drug [g]		X	X	X							
Clinical Efficacy Evaluations											
Parent(s)/caregiver(s) eCOA [h]	X	bid [i,j]	bid[i]	bid	bid	bid	bid	bid	bid	bid	bid
Clinical evaluation [k]	X	bid	bid	bid	bid	bid	bid	bid	bid	bid	bid
Clinician eCOA	X	bid	bid	bid	bid	bid	bid	bid	bid	bid	bid
Oxygen saturation (SpO_2) [l]	X (Q4h)	X (Q4h)	X (Q4h)	X (Q4h)	X (Q4h)	X (Q8h)	X (Q8h)	X (Q8h)	X (Q8h)	X (Q8h)	X (Q8h)
Medical resource utilization and health economics assessment	X	X	X	X	X	X	X	X	X	X	X
Clinical Safety Evaluations											
Complete physical examination [m]	X										X
Directed physical examination [n]		X	X		X		X		X		
Electrocardiogram [o]	X			X [p]							X
Vital signs	X	bid	bid	bid	bid	bid	bid	bid	bid	bid	bid

<table>
<tr><th>Phase</th><th>Screening</th><th colspan="4">Treatment Phase</th><th colspan="7">Follow-up Phase [a]</th></tr>
<tr><th>Day</th><th>0</th><th>1</th><th colspan="2">2</th><th>3 to 6 [b]</th><th>7 [c]
(+2 days)</th><th>8 to 9</th><th>10
(+2days)</th><th>11 to 13</th><th>14
(+2 days)</th><th>15 to 27</th><th>28
(±2 days)
EoS</th></tr>
<tr><td colspan="13">Study Procedure [d]</td></tr>
<tr><td colspan="13">Nasal Swabs [q]</td></tr>
<tr><td>Mid-turbinate nasal swab (if non-intubated) or endotracheal sample (if intubated): RSV diagnosis confirmation, viral and bacterial coinfection [r]</td><td>X</td><td></td><td colspan="2"></td><td></td><td></td><td></td><td></td><td></td><td></td><td></td><td></td></tr>
<tr><td>Mid-turbinate nasal swab (if non-intubated) or mid-turbinate nasal swab and endotracheal sample (if intubated): RSV viral load, viral resistance (genome sequencing), biomarker, and infectious RSV viral load (plaque assay) [s]</td><td></td><td>X [t]</td><td colspan="2">X</td><td>X</td><td>X</td><td></td><td>X</td><td></td><td>X</td><td></td><td>X</td></tr>
<tr><td colspan="13">Clinical Laboratory Assessments</td></tr>
<tr><td>Blood sampling for hematology [u]</td><td>X</td><td></td><td colspan="2">X</td><td></td><td>X</td><td></td><td></td><td></td><td>X</td><td></td><td></td></tr>
<tr><td>Blood sampling for biochemistry [u]</td><td>X</td><td></td><td colspan="2"></td><td></td><td>X</td><td></td><td></td><td></td><td>X</td><td></td><td>X</td></tr>
<tr><td>Urinalysis [v]</td><td>X</td><td></td><td colspan="2"></td><td>X</td><td></td><td></td><td></td><td></td><td>X</td><td></td><td></td></tr>
<tr><td colspan="13">Pharmacokinetics [q]</td></tr>
<tr><td rowspan="3">Blood sampling for pharmacokinetics</td><td></td><td colspan="3">Day 1 to Day 2</td><td></td><td></td><td></td><td></td><td></td><td></td><td></td><td></td></tr>
<tr><td></td><td>Post Dose 1</td><td colspan="2">Post Dose 2</td><td></td><td></td><td></td><td></td><td></td><td></td><td></td><td></td></tr>
<tr><td></td><td>0.25 - 2h</td><td>0.5 - 1h</td><td>7h (±4)</td><td></td><td></td><td></td><td></td><td></td><td></td><td></td><td></td></tr>
<tr><td>Group X [w]:</td><td></td><td>X</td><td>(X) [x]</td><td></td><td></td><td>X</td><td></td><td></td><td></td><td></td><td></td><td></td></tr>
<tr><td>Group Y [w]:</td><td></td><td>X</td><td></td><td>(X) [x]</td><td></td><td>X</td><td></td><td></td><td></td><td></td><td></td><td></td></tr>
<tr><td colspan="13">Ongoing Subject Review</td></tr>
<tr><td>Prior and Concomitant therapy</td><td>X</td><td>X</td><td colspan="2">X</td><td>X</td><td>X</td><td>X</td><td>X</td><td>X</td><td>X</td><td>X</td><td>X</td></tr>
<tr><td>Adverse events</td><td>X</td><td>X</td><td colspan="2">X</td><td>X</td><td>X</td><td>X</td><td>X</td><td>X</td><td>X</td><td>X</td><td>X</td></tr>
</table>

Abbreviations: bid: twice daily; ECG: electrocardiogram; eCOA: electronic clinical outcome assessment; eCRF: electronic case report form; EoS: end of study; IDMC: Independent Data Monitoring Committee; LD: loading dose; MD: maintenance dose; PK: pharmacokinetic(s); Q4h: every 4 hours; Q8h: every 8 hours; RSV: respiratory syncytial virus; SpO_2: peripheral capillary oxygen saturation.

Footnotes:

a. If a subject is discontinued from study drug, a follow-up visit will be scheduled 2 days (+2 days), 5 days (+2 days), 9 days (+2 days), and 23 days (±2 days) after the last dose of study drug was administered. Assessments should be performed as indicated in the Time and Events Schedule, respectively for the Day 7, Day 10, Day 14, and Day 28 visit.
b. The subject may be discharged after Day 2 study procedures, but may also remain hospitalized if deemed appropriate by the investigator. Hospitalization duration will not be extended solely for study purposes.
c. If treatment duration is increased to up to 10 days, then all assessments performed on Day 7 will again be performed 1 day (+2 days) after administration of the last MD.
d. The results of clinical/safety evaluations that are performed as part of routine clinical care while hospitalized will be collected in the eCRF for viral, safety, and/or clinical evaluation.
e. Procedures performed as part of standard of care within approximately 72 hours prior to screening completion (ie, randomization) may be used in determining study eligibility.
f. One LD followed by 9 MDs twice daily. MDs may be extended to up to 19 doses over 10 days if recommended by the IDMC.
g. Study drug LD should be given as soon as possible but no later than 4 hours after randomization. Subjects will be dosed with a single LD followed by 9 MDs twice daily (minimum 8 hours and maximum 16 hours apart with no more than 2 doses per calendar day) from Day 1 to Day 5/6 (depending on the timing of the LD). Lumicitabine may be given without regard to food. The date and time of dose administration, the date and time of food intake (limited to the food ingested within 30 minutes before or after study drug administration), and the type of food will be recorded in the eCRF.
h. An electronic device will be provided to the parent(s)/caregiver(s) at screening and the investigator/study-site personnel will provide sufficient information to enable the parent(s)/caregiver(s) to complete the parent(s)/caregiver(s) eCOA. Parent(s)/caregiver(s) will complete the eCOA screening and twice daily for the entire duration of hospitalization, at approximately the same time each day. On the day of discharge, parent(s)/caregiver(s) will complete the eCOA before the subject leaves the hospital.
i. The assessment on Day 1 must be recorded predose, before the LD. If the screening assessment was performed less than 8 hours prior to the LD, the predose assessment does not need to be performed.
j. The first postdose assessment must be performed between 6 and 12 hours after the LD (on Day 1 or Day 2, depending on the time of LD).
k. Clinical evaluation includes, but is not limited to, RSV disease symptoms, vital signs, oxygen requirement (type and duration), level of hospital care, duration of hospitalization, respiratory rate, body temperature, and SpO_2.
l. SpO_2 will be assessed Q4h (±30 minutes) while the subject is hospitalized through Day 7. In case the last SpO_2 assessment on Day 7 was performed after 8 PM, the first SpO_2 assessment on Day 8 should be performed within 8 hours from the last SpO_2 assessment on Day 7. For subjects who remain hospitalized after Day 7, SpO_2 assessment should then continue Q8h (±1 hour) until 24 hours after the cessation of supplemental oxygen.
m. A complete physical examination includes all body systems.
n. A directed physical examination includes respiratory system, nose, ear, throat, facial and neck lymph nodes, and skin examination.
o. A 12-lead ECG is strongly preferred but in cases where a 12-lead ECG is not possible or feasible, then a 6-lead ECG may be performed.
p. An ECG needs to be recorded on Day 4 for subjects who remain hospitalized at this time. If discharged earlier than Day 4, the subjects need to have an ECG recorded on the day of discharge. The ECG will be performed 30 minutes to 3 hours after the first dose of that day.
q. Leftover blood samples and nasal swabs may be used for exploratory biomarker analyses.
r. The mid-turbinate nasal swab or endotracheal sample taken during screening is placed in 3 mL of transport media and split into several aliquots. Assessments for RSV diagnosis at screening will be performed by a local laboratory and confirmed by a central laboratory. Other assessments will be done by a central laboratory.
s. Mid-turbinate nasal swabs (from the same nostril throughout the study) and/or endotracheal samples to assess RSV viral load and resistance should be taken by a trained study team member from Day 1 to Day 6 and on Day 7, Day 10, Day 14, and Day 28. Samples should be taken at approximately the same time each day prior to study drug administration. In case of nostril bleeding or irritation during the collection of a mid-turbinate nasal swab, all subsequent nasal swabs will be

performed on the contralateral nostril. Nasal swabs and endotracheal samples should be frozen as soon as possible after being collected. The actual times of sample collection and sample freezing must be recorded in the eCRF. Should the study drug duration be increased to up to 10 days (following review of the data by the IDMC), samples will be taken every day up to and including 1 day (+2 days) following the last dose of study drug. Quantitative culture of RSV using plaque assay may be performed centrally at timepoints selected by the virologist.

t. Mid-turbinate nasal swabs and/or endotracheal sample must be taken as close as possible to and before the LD on Day 1.

u. Only 1 blood sample will be collected if the biochemistry and hematology analyses are performed on the same day.

v. A urinalysis can be performed during the screening period or on Day 1 prior to the LD dose. A sample will also be collected on the last day of dosing, within 3 hours of the last dose.

w. At randomization, each subject will be randomly assigned to 1 of 2 PK sampling schemes, Group X or Y, with 3 PK timepoints per group: 2 samples will be taken on Days 1 to 2 and 1 random PK sample will be taken on Day 7. The actual dates and times of sample collection must be recorded in the eCRF. The IDMC may recommend that only 2 PK samples be used under the conditions of footnote w.

x. The IDMC may recommend that only 2 PK samples (post Dose 1 and Day 7) be collected after at least 9 subjects have completed treatment with the 60 mg/kg LD/40 mg/kg MD lumicitabine regimen if the Option A design is chosen or after at least 9 subjects in each cohort have completed treatment with the highest active lumicitabine regimen available in their respective cohort if the Option B design is chosen. The post Dose 2 sample will not be collected in this scenario.

TIME AND EVENTS SCHEDULE: AFTER HOSPITAL DISCHARGE

**Phase**	**Treatment Phase**	**Follow-up Phase** [a]						
**Day**	**3 to 6** [b]	**7** [c] **(+2 days)**	**8 to 9**	**10 (+2 days)**	**11 to 13**	**14 (+2 days)**	**15 to 27**	**28 (±2 days) EoS**
**Study Procedure**								
Screening/Administrative								
Body weight		X		X		X		X
Body length and head circumference								X
Study Drug Administration [d]								
Dispense/administer study drug [e] and study medication log	X							
Clinical Efficacy Evaluations								
Parent(s)/caregiver(s) eCOA [f]	bid	bid	bid	bid	bid	bid	X	X
Clinical evaluation and clinician eCOA [g]		X		X		X		X
Oxygen saturation (SpO_2)		X		X		X		X
Medical resource utilization and health economics assessment	X	X				X		X
Clinical Safety Evaluations								
Complete physical examination [h]								X
Directed physical examination [i]		X		X		X		
Electrocardiogram [j]								X
Vital signs		X		X		X		X
Nasal Swabs [k]								
Mid-turbinate nasal swab: RSV viral load, viral resistance (genome sequencing), biomarker, and infectious viral load (plaque assay) [l]	X	X		X		X		X
Clinical Laboratory Assessments								
Blood sampling for hematology [m]		X				X		
Blood sampling for biochemistry [m]		X				X		X
Urinalysis [n]		X				X		

Phase	Treatment Phase	Follow-up Phase [a]						
Day	3 to 6 [b]	7 [c] (+2 days)	8 to 9	10 (+2 days)	11 to 13	14 (+2 days)	15 to 27	28 (±2 days) EoS
**Study Procedure**								
Pharmacokinetics [k]								
Blood sampling for pharmacokinetics [o]: Group X or Group Y:		X						
Ongoing Subject Review								
Concomitant therapy	X	X	X	X	X	X	X	X
Adverse events	X	X	X	X	X	X	X	X

Abbreviations: bid: twice daily; ECG: electrocardiogram; eCOA: electronic clinical outcome assessment; eCRF: electronic case report form; EoS: end of study; IDMC: Independent Data Monitoring Committee; MD: maintenance dose; PK: pharmacokinetic(s); RSV: respiratory syncytial virus; SpO_2: peripheral capillary oxygen saturation.

Footnotes:

a. If a subject is discontinued from study drug, a follow-up visit will be scheduled 2 days (+2 days), 5 days (+2 days), 9 days (+2 days), and 23 days (±2 days) after the last dose of study drug was administered. Assessments should be performed as indicated in the Time and Events Schedule, respectively for the Day 7, Day 10, Day 14, and Day 28 visit.

b. The subject may be discharged after Day 2 study procedures. Telephone calls to parent(s)/caregiver(s) to facilitate compliance with study procedures between outpatient study visits are encouraged at the discretion of the investigator. Should the treatment duration be increased, all assessments during that period will be performed at the same frequency as indicated in the Time and Events Schedule for Day 3 to Day 6; follow-up assessments are to be started 1 (+2) day after the last MD.

c. If treatment duration is increased to up to 10 days, then all assessments performed on Day 7 will again be performed 1 day (+2 days) after administration of the last MD.

d. MDs given twice daily. MDs may be extended to up to 19 doses over 10 days if recommended by the IDMC.

e. Subjects will be dosed with MDs twice daily (minimum 8 hours and maximum 16 hours apart with no more than 2 doses per calendar day). Lumicitabine may be given without regard to food.

f. An electronic device will be provided to the parent(s)/caregiver(s) the investigator/study-site personnel will provide sufficient information to enable the parent(s)/caregiver(s) to complete the parent(s)/caregiver(s) eCOA. Parent(s)/caregiver(s) will complete the eCOA twice daily (in the morning and in the evening) until the Day 14 visit, and once daily (in the evening) up to the Day 28 visit, at approximately the same time each day. Whenever possible, the same parent(s)/caregiver(s) should complete the eCOA throughout the study.

g. Clinical evaluation includes, but is not limited to, RSV disease symptoms, vital signs, oxygen requirement (type and duration), level of hospital care, duration of hospitalization, respiratory rate, body temperature, and SpO_2.

h. A complete physical examination includes all body systems.

i. A directed physical examination includes respiratory system, nose, ear, throat, facial and neck lymph nodes, and skin examination.

j. A 12-lead ECG is strongly preferred but in cases where a 12-lead ECG is not possible or feasible, then a 6-lead ECG may be performed.

k. Leftover blood samples and nasal swabs may be used for exploratory biomarker analyses.

l. Mid-turbinate nasal swabs (from the same nostril throughout the study) to assess RSV viral load and resistance should be taken by parent(s)/caregiver(s) from Day 3 to Day 6 and by a trained study team member on Day 7, Day 10, Day 14, and Day 28. Samples should be taken once daily at approximately the same time

each day prior to study drug administration. In case of nostril bleeding or irritation during the collection of a mid-turbinate nasal swab, all subsequent nasal swabs will be performed on the contralateral nostril. Should the study drug duration be increased to up to 10 days (following review of the data by the IDMC), samples will be taken every day at approximately the same time, up to and including 1 day (+2 days) following the last dose of study drug. The parent(s)/caregiver(s) should collect nasal swab specimens after being trained by the investigator/study-site personnel. The actual dates and times of nasal swab collection at home must be recorded in the eCOA by the parent(s)/caregiver(s). Nasal swabs collected at home should be stored immediately between 2°C and 8°C (in the refrigerator) and brought to the site at the next visit. Quantitative culture (plaque assay) will not be performed on samples collected at home but might be performed on samples collected at the hospital on Day 14 and/or Day 28 visit upon the request of the virologist.

m. Only 1 blood sample will be collected if the biochemistry and hematology analyses are performed on the same day.
n. A urinalysis will be performed on Day 7 if the subject is discharged before the sample on the last day of dosing can be collected.
o. A random PK sample will be taken on Day 7. The actual dates and times of sample collection must be recorded in the eCRF.

ABBREVIATIONS

AE	adverse event
ALT	alanine aminotransferase
AUC	area under the plasma concentration-versus time curve
AUC_{0-24h}	area under the plasma concentration-versus time curve from time 0 to 24 hours after dosing
AUC_{0-last}	are under the plasma curve from time 0 to the time of the last quantifiable concentration
BCRP	breast cancer resistance protein
C_{12h}	predicted concentration at 12 hours post dose
C_{max}	maximum observed (or predicted) analyte concentration
CYP	cytochrome P450
DBP	diastolic blood pressure
DMID	Division of Microbiology and Infectious Diseases
DNA	deoxyribonucleic acid
EC_{90}	90% of maximal effective concentration
ECG	electrocardiogram
eCOA	electronic clinical outcome assessment
eCRF	electronic case report form
eDC	electronic data capture
eGFR	estimated glomerular filtration rate
ER	emergency room
GCP	Good Clinical Practice
hMPV	human metapneumovirus
IB	Investigator's Brochure
ICF	informed consent form
ICH	International Conference on Harmonisation of Technical Requirements for Pharmaceuticals for Human Use
ICU	intensive care unit
IDMC	Independent Data Monitoring Committee
IEC	Independent Ethics Committee
IRB	Institutional Review Board
ITT	intent-to-treat
ITT-i	intent-to-treat infected
IV	intravenous
IWRS	interactive web response system
LD	loading dose
LRTI	lower respiratory tract infection
MAD	multiple ascending dose
MD	maintenance dose
NOAEL	no-observed-adverse-effect level
NOEL	no-observed-effect level
NTP	nucleoside triphosphate
OAT	organic anion transporter
OATP	organic anion-transporting polypeptide
PCR	polymerase chain reaction
PD	pharmacodynamic(s)
PK	pharmacokinetic(s)
popPK	population-derived pharmacokinetic(s)
PQC	product quality complaint
PRESORS	Pediatric RSV Electronic Severity and Outcome Rating System
qRT-PCR	quantitative real-time reverse transcriptase-polymerase chain reaction
QTcF	QT interval corrected for heart rate according to Fridericia's formula
RBC	red blood cell
RNA	ribonucleic acid
RSV	respiratory syncytial virus
SAE	serious adverse event
SAP	statistical analysis plan
SBP	systolic blood pressure

SD	standard deviation
SpO_2	peripheral capillary oxygen saturation
SUSAR	suspected unexpected serious adverse reaction
$t_{1/2}$	half-life
ULN	upper limit of normal
WBC	white blood cell

DEFINITIONS OF TERMS

Electronic Clinical Outcome Assessment (eCOA)	Includes parent/caregiver-reported outcomes, clinician-reported outcomes, observer-reported outcomes and performance-reported outcomes collected on an electronic device.

1. INTRODUCTION

Lumicitabine (also known as JNJ-64041575 and ALS-008176) is a 3′,5′-bisisobutyrate prodrug of the cytidine nucleoside analog JNJ-63549109 (also known as ALS-008112), which is being developed as an orally administered antiviral therapy for the treatment of infants, children, and adults infected with respiratory syncytial virus (RSV) and human metapneumovirus (hMPV). Once administered, lumicitabine is rapidly and efficiently converted by esterases to JNJ-63549109. Inside cells, JNJ-63549109 is subsequently converted to the JNJ-63549109-5′-triphosphate JNJ-65409136 (also known as ALS-008136), which, as the active metabolite of the compound, is a potent and selective inhibitor of the RSV and hMPV RNA polymerase activity via a classic chain termination mechanism. JNJ-64167896 (also known as ALS-008144), the uridine metabolite of JNJ-63549109, is the inactive major metabolite detected in systemic circulation. Table 1 gives an overview of the investigational product used in this study and its metabolites, including the prior and current compound numbers.

Table 1: Investigational Product and Its Metabolites

Compound Name/Number	Description
Lumicitabine (JNJ-64041575, ALS-008176)	3',5'-bisisobutyrate prodrug of JNJ-63549109
JNJ-63549109 (ALS-008112)	parent nucleoside, major metabolite of lumicitabine
JNJ-64412309 (ALS-008206)	3'-isobutyrate monoester of JNJ-63549109, minor metabolite
JNJ-64412296 (ALS-008207)	5'-isobutyrate monoester of JNJ-63549109, minor metabolite
JNJ-64167896 (ALS-008144)	uridine metabolite of JNJ-63549109, major metabolite
JNJ-65409136 (ALS-008136)	5'-triphosphate of JNJ-63549109 (NTP), intracellular metabolite, active
JNJ-65409123 (ALS-008137)	5'-monophosphate of JNJ-63549109, intracellular metabolite

Abbreviation: NTP: nucleoside triphosphate.

For the most comprehensive nonclinical and clinical information regarding lumicitabine, refer to the latest version of the Investigator's Brochure (IB) for lumicitabine.[16]

The term "sponsor" used throughout this document refers to the entities listed in the Contact Information page(s), which will be provided as a separate document.

1.1. Background

1.1.1. Respiratory Syncytial Virus

Respiratory syncytial virus is an RNA virus and a member of the *Pneumoviridae* family, which also includes hMPV. The RSV season occurs during winter months in regions with temperate climates in the Northern and Southern hemispheres and throughout the year or peaks semiannually in tropical regions.[5,22]

Respiratory syncytial virus causes acute lower respiratory tract infection (LRTI) and is a major cause of hospital admissions and death in young children worldwide. RSV is a leading cause of lower respiratory disease in infants.[18] In 2005, an estimated 33.8 million episodes of RSV LRTI occurred worldwide in infants. Of these, at least 3.4 million severe cases of LRTI required hospitalization, and an estimated 66,000 to 199,000 deaths occurred, mostly in the developing world.[18] In the United States, it has been estimated that annually RSV infection causes 57,527 hospitalizations and 2.1 million emergency room (ER) or outpatient visits.[11] Infants that

are born prematurely or close to the RSV season and/or suffering from bronchopulmonary dysplasia or congenital heart disease are at the highest risk of developing severe RSV-related acute LRTI.[9]

Treatment of infants and children with severe RSV disease is largely supportive, consisting of oxygen therapy, nutrition, and fluids. Aerosolized ribavirin, a synthetic guanosine analog and broad spectrum antiviral agent, is approved for treatment of infants with severe LRTIs, but its use is limited due to questionable efficacy and complexity of administration.[1,10] Hence, there is a significant unmet need for a novel antiviral agent against RSV.

1.1.2. Information on the Product

Lumicitabine is a 3',5'-bisisobutyrate prodrug, which is readily metabolized to the cytidine nucleoside analog, JNJ-63549109. Subsequently, inside cells, JNJ-63549109 is efficiently converted to its nucleoside triphosphate (NTP), JNJ-65409136. This NTP is a potent inhibitor of both RSV and hMPV RNA polymerase activity via a classic chain termination mechanism. JNJ-64167896, the uridine metabolite of JNJ-63549109, is the inactive major metabolite detected in systemic circulation. A summary of the major findings from nonclinical and clinical studies available at the time of initial protocol writing is presented below. Please refer to the IB[16] for more details.

Nonclinical Studies

Pharmacologic Profile

The antiviral activity and selectivity of the parent nucleoside JNJ-63549109 and its prodrug, lumicitabine, were evaluated in vitro using a combination of cell-based RSV infectious and subgenomic replicon reporter systems, together with cell-extracted replicase and recombinant RSV polymerase L-P complex. In vivo, lumicitabine was evaluated in the African Green monkey model of RSV infection.

The major findings from these studies were:

- JNJ-63549109 and lumicitabine inhibit RSV laboratory-adapted strains, clinical isolates, and RSV replication in the subgenomic replicon system.
- The JNJ-63549109-5′-triphosphate (JNJ-65409136), the active metabolite of the compound, is a potent and selective inhibitor of RSV RNA polymerase activity, demonstrating an inhibition constant (K_i) of 0.09 µM, via a classic chain termination mechanism.
- JNJ-65409136 is formed in high amounts in A549 lung epithelial cells, primary human nasal and bronchial epithelial cells, and monkey lung, and demonstrates an intracellular half-life ($t_{1/2}$) of approximately 17.6 hours in vitro (in normal human bronchial epithelial cells) and 29 hours in vivo (in monkey lungs).
- In vivo, orally administered lumicitabine significantly suppressed RSV replication in the African Green monkey model.
- In vitro inhibition of human deoxyribonucleic acid (DNA) polymerases α, β, or γ, and of human RNA polymerase II by JNJ-65409136 was insignificant (>100 µM) under the conditions used. In addition, JNJ-65409136 was not an efficient substrate for the human

mitochondrial RNA polymerase. Further, JNJ-63549109 did not inhibit mitochondrial protein synthesis in either 8- or 28-day assays in the human hepatoma G2, human hepatoma type 7, and human T-cell leukemia derived MT-4 cell lines. In addition, JNJ-63549109 did not significantly inhibit the replication of a panel of viruses unrelated to RSV.

- Reduced progenitor differentiation and proliferation by JNJ-63549109 was seen for the human granulocyte-monocyte (50% inhibitory concentration =8.71 µM or 2,558 ng/mL) and erythroid precursor progenitors (50% inhibitory concentration =9.21 µM or 2,705 ng/mL) from bone marrow stem cells (14 days assay duration).

The major safety pharmacology findings were:

- No human ether-a-go-go gene potassium channel current blockage of lumicitabine or its metabolites.
- No central nervous system effects in rats up to 500 mg/kg/day.
- No cardiovascular and respiratory effects in dogs up to 500 mg/kg/day.

Toxicology Profile

Once administered, lumicitabine is rapidly and efficiently converted to JNJ-63549109. Thus, the toxicity profile of lumicitabine is primarily driven by JNJ-63549109.

The major toxicity findings were:

Repeat-dose Toxicity

- Target organ for toxicity was the hematopoietic system in adult and juvenile rats and dogs (JNJ-63549109 area under the plasma concentration versus time curve from time 0 to 24 hours after dosing [$AUC_{0\text{-}24h}$] ≥212,000 ng.h/mL). The no-observed-adverse-effect level (NOAEL) doses of lumicitabine and exposure of JNJ-63549109 are presented in Table 2.

Table 2: NOAEL Doses of Lumicitabine and Exposure of JNJ-63549109 in Adult and Juvenile Rats and Dogs After Repeated Dosing of Lumicitabine

Target Organ	Key Finding	Species/Age Group	Dose (mg/kg bid)	NOAEL (JNJ-63549109 $AUC_{0\text{-}24h}$) (ng.h/mL)
Hematology	↓ reticulocytes, ↓ red blood cell parameters, reversible	Juvenile rat (PND1-28)[a]	150	93,900[b]
		Juvenile dog (PND7-34)	25	108,228
Bone marrow	Cytological changes in bone marrow cells, ↓ myeloid-to-erythroid ratios, reversible	Adult rat (14 days)	150	53,600
		Adult dog (14 days)	75	192,500

Abbreviations: $AUC_{0\text{-}24h}$: area under the plasma concentration versus time curve from time 0 to 24 hours after dosing; bid: twice daily; NOAEL: no-observed-adverse-effect level; PND: postnatal day.

a. Effects in rats aged PND21-45 were similar to adult rats.
b. Estimated on PND28 predose values.

- The effect on reticulocytes was considered an early marker of toxicity, a monitorable parameter for humans. After 14 days of repeated dosing, these changes were reversible in both adult and juvenile animals.

- No effects on early developmental markers, neurobehaviour, respiratory function, bone geometry, bone densitometry, ophthalmological evaluations, or electrocardiograms (ECGs) in juvenile rats (150 mg/kg twice daily; JNJ-63549109 AUC_{0-24h} of 55,450 ng.h/mL) and dogs (75 mg/kg twice daily; JNJ-63549109 at AUC_{0-24h} of 258,862 ng.h/mL).

Genotoxicity

- Non-mutagenic (negative Ames test) and no DNA damage (COMET assay; 1,750 mg/kg/day for 3 days) potential for lumicitabine or its metabolites.
- Increases in structural chromosomal aberration in human peripheral blood lymphocytes induced by JNJ-63549109 (≥10 µg/mL). Positive micronucleus test in rats (≥425 mg/kg/day lumicitabine for 3 days) at high exposure of JNJ-63549109 (AUC_{0-24h} ≥107,983 ng.h/mL). The NOEL for in vitro chromosomal aberrations was 5 µg/mL and for in vivo micronucleus induction was an AUC_{0-24h} of 57,595 ng.h/mL.
- The mechanism of clastogenicity is an indirect, threshold-based mechanism of intracellular NTP pool disturbances rather than direct DNA reactivity, as described for other nucleoside analogs in the public domain.
- A maximum average threshold of JNJ-63549109 exposure in humans has been defined as an AUC_{0-24h} of 20,000 ng.h/mL (or 25,200 ng.h/mL in whole blood) ie, 2.7-fold lower than the NOAEL in the repeat-dose toxicity studies and well above the effective antiviral exposure in the human RSV challenge Study ALS-8176-502 (see Section Clinical Studies below).

Reproductive Toxicity

- The NOAEL for male reproductive toxicity/fertility was 250 mg/kg twice daily (JNJ-63549109 AUC_{0-24h} of 165,000 ng.h/mL). In view of the slight decrease in corpora lutea and corresponding decrease in implantations and viable embryos (without overall impact on female fertility or in utero survival), the NOEL for female reproductive toxicity as well as early embryonic toxicity was 250 mg/kg twice daily (JNJ-63549109 AUC_{0-24h} of 193,000 ng.h/mL).
- The effects of lumicitabine on developmental and reproductive parameters were a decrease in fetal weight in rats at 400 mg/kg twice daily and an increase in post-implantation losses in rabbits at ≥20 mg/kg twice daily, with no teratogenic effects in fetuses in either rats or rabbits. The NOAEL for maternal toxicity and embryo-fetal development in rats was 125 mg/kg twice daily (JNJ-63549109 maximum observed (or predicted) analyte concentration [C_{max}] of 11,400 ng/mL and AUC_{0-24h} of 87,800 ng.h/mL). The NOAEL for embryo-fetal development in rabbits was 10 mg/kg twice daily (JNJ-63549109 AUC_{0-24h} of 8,220 ng.h/mL) and the maternal NOAEL in rabbits was 20 mg/kg twice daily.

Other Toxicity

- There was no measurable cytotoxicity of JNJ-63549109 (>100 µM, 3 days) in the human epithelial type 2 and human hepatoma type 7 cell lines. There was a dose-dependent decrease in cell viability in 3/7 cell lines (8 days).

Pharmacokinetic and Metabolism Profile

The major nonclinical pharmacokinetic (PK) and metabolism findings were:

- The prodrug, lumicitabine, was well absorbed after oral administration and rapidly and efficiently converted to the parent nucleoside JNJ-63549109 by esterases. Plasma exposures of JNJ-63549109 were comparable between adult and juvenile animals at the same dose levels. The uridine metabolite JNJ-4167896 was a major circulating metabolite in monkeys.
- Food slowed down the rate of oral absorption (lowered C_{max} values) of lumicitabine, but did not change the extent of oral absorption (no change in area under the plasma concentration versus time curve [AUC]) in monkeys.
- The oral bioavailability, in terms of exposure of the parent nucleoside JNJ-63549109, was 44% in dog and monkey.
- Inside cells JNJ-63549109 is extensively phosphorylated. In lung, high levels of the monophosphate JNJ-654109123 and especially the active triphosphate JNJ-65409136 were measured.
- Cytochrome P450 (CYP) enzymes play a minor role in lumicitabine metabolism. Besides no relevant inhibition nor induction of CYP enzymes by lumicitabine, JNJ-63549109, or other metabolites was observed.
- Lumicitabine JNJ-63549109 and JNJ-64167896 are neither substrates nor inhibitors of P-glycoprotein. Lumicitabine but not JNJ-63549109 and JNJ-64167896, might be weak inhibitors of organic anion-transporting polypeptide (OATP) 1B1/1B3, but none of them are substrates of these OATP1B transporters. Data indicated that JNJ-63549109 and JNJ-64167896 are neither substrates nor inhibitors of organic cation transporter 2, organic anion transporter (OAT) 1, and bile salt export pump. There is no clear indication that lumicitabine and JNJ-63549109, but not JNJ-64167896, are substrates of breast cancer resistance protein (BCRP), but none of them are inhibitors of BCRP. JNJ-63549109 is a substrate but not an inhibitor of OAT3 (K_i >258 µM). JNJ-64167896 is not a substrate or inhibitor of OAT3.
- Based on the in vitro data, lumicitabine and its major metabolites are predicted to have a low potential for CYP- and transporter-mediated drug-drug interactions, with the possible exception of strong OAT3 inhibitors.

Clinical Studies

Human Pharmacokinetics

Single-dose PK in healthy adult subjects was characterized in Study ALS-8176-501. When administered orally to healthy adult subjects as a suspension formulation in the fasted state, lumicitabine was rapidly converted to JNJ-63549109. The only other major metabolite measurable in plasma was JNJ-64167896, the inactive uridine metabolite of JNJ-63549109. While the terminal $t_{1/2}$ of JNJ-63549109 was calculated to be approximately 63 hours, the majority of the plasma concentrations decreased to low levels within 6 hours postdose, indicating rapid and extensive distribution of JNJ-63549109. Following single doses of up to 750 mg, the C_{max} and AUC of JNJ-63549109 increased linearly, but less than dose proportionally with increasing doses of lumicitabine. The plasma PK profile of the metabolite, JNJ-64167896, was

similar to that noted with JNJ-63549109 and exposure was approximately 18% to 33% of that of JNJ-63549109. In Study 64041575RSV1003, JNJ-63549109 AUC showed no deviation from dose proportionality within the lumicitabine 1,500 mg to 3,000 mg dose range, after a single dose. JNJ-63549109 C_{max} increased with the dose, but the increase was less than dose proportional for the 2,500 mg and 3,000 mg doses as compared to the 1,500 mg dose. JNJ-64167896 AUC and C_{max} showed no deviation from dose proportionality.

Urinary excretion was assessed following a single 500-mg oral dose of lumicitabine in the fasted state. No prodrug was recovered in urine, which is probably due to extensive metabolism. The major metabolites measured in urine were JNJ-63549109 and JNJ-64167896, which accounted for approximately 25% and 7% of the administered dose, respectively.

Food effect was assessed following a single 250-mg oral dose of a lumicitabine suspension. Intake of lumicitabine with a high fat, high calorie meal lowered JNJ-63549109 C_{max} by approximately 50% as compared with the fasted state, without affecting the JNJ-63549109 AUC. Study ALS-8176-509, administering lumicitabine as 500-mg tablets, indicated that food has a minor effect on JNJ-63549109 exposure and plasma peaks with an average increase in AUC of 19% and decrease in C_{max} of 16%.

Study ALS-8176-504 determined the absolute bioavailability of JNJ-63549109 and mass balance of lumicitabine in healthy adult subjects. Following oral administration of a 375 mg ^{14}C-lumicitabine solution, 84% of the total administered radioactive dose was recovered in the urine and feces during the 312-hour collection period. Approximately half of the administered dose was recovered within the first 24 hours. Approximately two-thirds of the total radioactivity was recovered in the urine suggesting renal elimination is the major route of elimination for the prodrug and its metabolites. Metabolite profiling and identification confirmed that JNJ-63549109 and JNJ-64167896 are the 2 major metabolites in plasma, urine, and feces. The absolute oral bioavailability of lumicitabine, measured by the formation of the parent nucleoside, JNJ-63549109 (AUC from time zero to the time of the last quantifiable concentration [$AUC_{0\text{-}last}$]), was determined to be approximately 60%.

Based on an integrated population-derived PK (popPK) approach, race (including Japanese) does not affect exposures to JNJ-63549109.

Multiple dose PK was characterized in healthy adult subjects in Study ALS-8176-501, in healthy adult subjects challenged with RSV in Study ALS-8176-502, and in hospitalized adult subjects with RSV infection in Study ALS-8176-510. Following multiple oral doses of lumicitabine in healthy adult subjects in Study ALS-8176-501, including 2 loading doses (LDs) up to 750 mg on Day 1 followed by administration of multiple doses up to 500 mg up to 13 days every 12 hours (Q12h), the plasma profiles of JNJ-63549109 and JNJ-64167896 were similar to that obtained following single doses of lumicitabine. Accumulation of either JNJ-63549109 or JNJ-64167896 was insignificant after 5 days of dosing. Steady-state minimum (or predicted) observed analyte concentration of JNJ-63549109 was reached by the second dose (ie, approximately 24 hours after the first dose) and minimal changes in minimum (or predicted) observed analyte concentration were noted between Days 1 and 5 or 14. In general, the plasma profiles for JNJ-63549109

following the afternoon dose were lower than following the morning dose. Study ALS-8176-502 was an RSV challenge study in healthy adult subjects. Subjects received 5-day dose regimens of lumicitabine as an oral suspension. The 3 dose regimens were: 375 mg Q12h (n=11), 750 mg LD/500 mg MD Q12h (n=14), and 750 mg LD/150 mg MD Q12h (n=19). Intensive PK samples were evaluated on Days 1 and 5 of the study and the highest mean plasma $AUC_{0\text{-}24h}$ for JNJ-63549109 was 10,794 ng.h/mL for the 750 mg LD/500 mg MD group on Day 5. There was no difference between the average exposures after oral dosing of lumicitabine in healthy adult subjects in Study ALS-8176-501 as compared with healthy adult subjects challenged with RSV in Study ALS-8176-502. Limited PK data from Study ALS-8176-510 suggests that the exposure to JNJ-63549109 in adults infected with RSV appears to be higher than in healthy subjects (1.86-fold higher exposure seen in Study ALS-8176-510 versus healthy adult subjects in Study ALS-8176-502).

Study ALS-8176-503 is a Phase 1b, randomized, double-blind, placebo-controlled, 2-part study designed to evaluate the safety, tolerability, PK, and pharmacodynamics (PD) of single ascending doses and multiple ascending doses (MAD) of orally administered lumicitabine in infants hospitalized with RSV infection. In this study, subjects are randomized in a 3:1 ratio to receive lumicitabine as a single dose up to 25 mg/kg, or a multiple dose regimen consisting of an LD (up to 40 mg/kg) followed by 5-day MDs (up to 20 mg/kg) twice daily, or a matching placebo regimen. This study is ongoing at the time of protocol writing. Limited PK data from the MAD part of Study ALS‑8176-503 shows that the mean plasma JNJ‑63549109 $AUC_{0\text{-}24h}$ increased linearly with the dose. The highest mean $AUC_{0\text{-}24h}$ value reached in this population was an average of 7,763 ng.h/mL for the 40 mg/kg LD/20 mg/kg MD regimen.

Efficacy/Safety Studies

The efficacy of lumicitabine in naturally infected populations has not yet been demonstrated; however, it has been assessed in healthy adult subjects infected with RSV in a human challenge model (Study ALS-8176-502). In this study, 62 subjects were enrolled into 3 study periods, inoculated with the RSV-A Memphis 37b strain, and monitored for RSV infection using a quantitative reverse transcriptase-polymerase chain reaction (qRT-PCR) assay. Twelve hours after infection was detected by qualitative polymerase chain reaction (PCR) or 6 days after inoculation, whichever occurred first, subjects were randomized to placebo or 1 of 3 lumicitabine dose regimens: 750 mg LD/500 mg MD (Q12h), 750 mg LD/150 mg MD (Q12h), or 375 mg, (Q12h) for 5 days. Data from this study demonstrate that MDs of 150 and 500 mg lumicitabine following a 750 mg LD, resulted in rapid, substantial declines in RSV viral load with an accompanying comparable improvement in signs and symptoms of RSV infection compared with placebo-treated subjects.[6] There was a statistically significant difference in RSV AUC in subjects treated with 375 mg twice daily compared with placebo but the effect was more pronounced when the regimens included a LD.

Data from 6 Phase 1/2a studies (ALS-8176-501, ALS-8176-502, ALS-8176-504, ALS-8176-509, 64041575RSV1001 [ALS-8176-511], and 64041575RSV1003) in healthy adult subjects (N=234), indicate that lumicitabine was well tolerated and that no safety concerns were identified, after receiving lumicitabine as single doses up to 3,000 mg or as multiple doses up to

750 mg twice daily on Day 1 followed by up to 500 mg Q12h MDs for up to 13 days. In these studies, there were no deaths, no serious adverse events (SAEs) or adverse events (AEs) leading to study drug discontinuation reported. All AEs were mild or moderate in severity, except for 3 events, all of which were severe. Two of these severe events were asymptomatic laboratory abnormalities (increased alanine aminotransferase (ALT) and increased blood creatine phosphokinase) and occurred in Study ALS-8176-502; neither event was considered related to lumicitabine. The third severe AE (increased cholesterol) occurred in Study 64041575RSV1001 (ALS-8176-511; single ascending dose study in healthy Japanese adult subjects) on Day 14 after the subject received a single oral dose of lumicitabine (750 mg) on Day 1 and was considered possibly related to lumicitabine. Among healthy adult subjects receiving multiple doses of lumicitabine, the most commonly reported AEs (ie, ≥3 subjects) that occurred more often in lumicitabine-treated versus placebo-treated subjects were: epistaxis, ALT increased, headache, oropharyngeal pain, platelet count decreased, and upper respiratory tract infection.

No prolongation of the QT interval corrected for heart rate according to Fridericia's formula (QTcF) of clinical or regulatory concern was noted in Study 64041575RSV1003.

As of 20 April 2017, there have been 2 hematologic SAEs reported in the ongoing clinical studies in hospitalized adult or pediatric subjects who are infected with RSV.

- In Study 64041575RSV2003, a Grade 3 SAE of pancytopenia (laboratory Grade 4) that started 2 days after the last dose of lumicitabine (1,000 mg LD/500 mg MD twice daily) was reported in a subject with a medical history of pneumonia, bronchiectasis, anemia, emaciation, and non-tuberculous mycobacteriosis. The event was considered probably related to lumicitabine by the investigator.
- In Study ALS-8176-503, a Grade 4 SAE of neutropenia (laboratory Grade 4) was reported within 17 hours after the first dose of lumicitabine (40 mg/kg LD/20 mg/kg MD twice daily). The event was considered possibly related to lumicitabine by the investigator.

In addition, there has been 1 non-serious Grade 4 hematologic AE (neutropenia, laboratory Grade 4) in Study ALS-8176-510 in adult hospitalized subjects who are infected with RSV. The event started 22 days after the last dose of lumicitabine (750 mg LD/500 mg MD) and was reported in a subject with a complex past medical history. The event was considered possibly related to lumicitabine by the investigator.

In addition to the above-described serious/severe hematological AEs, up to 20 April 2017, 7 SAEs have been reported in these 3 studies in hospitalized adult or pediatric subjects infected with RSV. In Study ALS-8176-503, 6 unrelated SAEs have been reported after single (phlebitis, bacterial pneumonia with respiratory failure, and sinus tachycardia) or multiple doses (lymphadenitis, and bronchiolitis [2 events in 1 child]) of the study drug. One of these SAEs (bacterial pneumonia with respiratory failure) was also considered severe. In Study ALS-8176-510, a single, unrelated SAE (abdominal pain) was reported. The safety data for all 3 studies are regularly reviewed by unblinded Independent Data Monitoring Committees (IDMCs), which have identified no other potential safety concerns.

Factors other than treatment with lumicitabine, including underlying RSV infection, are more likely to have contributed to these serious/severe hematologic AEs. However, a potential contribution of lumicitabine cannot be excluded. The risk-benefit assessment for the continued development of lumicitabine remains favorable given the population targeted (hospitalized subjects) and the lack of available treatment for RSV infection.

1.2. Overall Rationale for the Study

Globally, RSV is the most common cause of childhood lower respiratory disease.[18] Palivizumab is approved for the prevention of RSV-related hospitalizations in high-risk infants and aerosolized ribavirin is approved for treatment of infants with severe LRTIs, but its use is limited due to questionable efficacy and complexity of administration.[1,11] Current treatment of RSV infection is largely limited to supportive care, consisting of supplemental oxygen therapy, nutrition, hydration and, in some cases, mechanical ventilation. Hence, there is a significant unmet medical need for a novel antiviral agent against RSV.

This study is designed to determine the antiviral activity, clinical outcomes, safety, tolerability, and PK of multiple lumicitabine dose regimens in a population of hospitalized infants and children aged ≥28 days to ≤36 months naturally infected with RSV.

1.3. Benefit-risk Evaluation

1.3.1. Known Benefits

Lumicitabine demonstrated a significant antiviral effect and reduced the signs and symptoms of RSV infection in the RSV human challenge model. However, the clinical benefit of this compound in the treatment of RSV infection remains to be fully established.

1.3.2. Potential Benefits

Lumicitabine has demonstrated potent antiviral activity against RSV in vitro, in the African Green Monkey animal model and in a human challenge model. Subjects participating in this study might have a benefit regarding the clinical course of their RSV infection. Results from lumicitabine clinical studies may be useful in developing a new therapy for RSV infection.

1.3.3. Known Risks

All therapies have the potential to cause adverse experiences. No formal adverse drug reaction determination has been performed.

In healthy adult subjects (N=234 dosed with lumicitabine), data from Phase 1/2a studies (ALS-8176-501, ALS-8176-502, ALS-8176-504, AL-8176-509, 64041575RSV1001/ ALS-8176-511, and from 64041575RSV1003) indicate that lumicitabine was well tolerated with no safety concerns identified after receiving single doses up to 3,000 mg or multiple doses up to 750 mg twice daily on Day 1, followed by up to 500 mg twice daily MD for up to 13 days. In adult or pediatric subjects hospitalized with RSV, 3 hematological serious/severe AEs have occurred and subsequently resolved (see Section 1.3.4, Potential Risks).

1.3.4. Potential Risks

The nonclinical toxicity profile for JNJ-63549109 was similar to that previously observed for other marketed nucleoside analogs, where hematopoietic changes and genotoxicity were noted.

The toxicity profile established for JNJ-63549109 in nonclinical toxicology studies indicates a reversible and dose-related effect on the hematopoietic system where changes in bone marrow cytology were associated with changes in hematological parameters; specifically, decreased circulating reticulocytes and hemoglobin, and hypocellularity of erythroid cell lines in the bone marrow. In these studies, no significant impact on neutrophils, or platelets was observed at exposures approximately 10-fold higher than those assessed in clinical studies (plasma JNJ-63549109 AUC of approximately 20,000 ng.h/mL).

As of 20 April 2017, in adult or pediatric subjects hospitalized with RSV, 3 hematological serious/severe AEs have occurred and subsequently resolved. In all 3 cases, the subject received lumicitabine, but the 2 cases in adult subjects (pancytopenia SAE in Study 64041575RSV2003; severe neutropenia in Study ALS-8176-510) were both confounded by multiple medical comorbidities and recent/concomitant use of multiple medications that have been reported to cause bone marrow toxicity. Furthermore, the clinical course of neutrophil counts varies substantially across these 3 cases, with onset occurring within 24 hours, 6 days, and 27 days after the first dose of lumicitabine in the pediatric neutropenia case (Study ALS-8176-503), the adult pancytopenia case (Study 64041575RSV2003), and the adult severe neutropenia case (Study ALS-8176-510), respectively. Moreover, the presence of comorbidities, ie, non-tuberculous mycobacteriosis and underlying RSV infection, might provide an alternative plausible etiology for the SAEs of pancytopenia and neutropenia in Study 64041575RSV2003 and Study ALS-8176-503, respectively. In light of the 3 serious/severe AEs related to neutropenia/pancytopenia that have occurred in RSV-infected subjects exposed to lumicitabine, continued close monitoring of hematological parameters and clinical status in both RSV-infected and healthy subjects is warranted, as additional data are being collected in ongoing/future studies with lumicitabine. These hematological side effects observed in both nonclinical and clinical studies with lumicitabine were reversible.

In addition, JNJ-63549109 has been shown to cause clastogenicity, specifically breakage of chromosomes, through a non-DNA-reactive, indirect mechanism, resulting from an imbalance of intracellular nucleotide pools. The threshold of clastogenicity for JNJ-63549109, as defined by the lowest concentration where an effect was observed, 10 μg/mL for in vitro studies and an AUC of 107,983 ng.h/mL for in vivo studies. The NOEL for genotoxicity of JNJ-63549109 was 5 μg/mL for in vitro studies and an AUC of 57,595 ng.h/mL was reported for in vivo studies.

Considering the clastogenicity changes in animal studies, JNJ-63549109 plasma exposures for human subjects will be limited to a maximum average $AUC_{0\text{-}24h}$ of 20,000 ng.h/mL (or 25,200 ng.h/mL in whole blood) for any given dose. This threshold provides safety margins that are at least 2.7-fold lower than the lowest systemic NOAEL exposure obtained in nonclinical toxicity studies.

Potential risks associated with lumicitabine administration that might occur in humans are based on theoretical known risks associated with other nucleoside analogs. Theoretical risks for nucleoside analogs are neuropathy, mitochondrial dysfunction, myopathy (including cardiomyopathy), lactic acidosis, hepatomegaly with steatosis and pancreatitis. None of these theoretical risks have been observed in lumicitabine studies to date.

All antivirals carry the potential risk of inducing resistance. However, it is not expected that the clinical course of the subject will be substantially influenced by the emergence of resistance, as RSV infection is typically an acute illness in otherwise healthy and non-immunocompromised pediatric subjects whose immune system eradicates the virus.

Study procedures such as blood sampling carry a potential risk to the subject because of their age and corresponding higher discomfort and lower blood volume. Several design characteristics of the study aim to minimize the stress and risks for the subject. The number of subjects exposed as part of the study has been limited to the absolute minimum to reach the PK objectives of the trial: for each of the subjects enrolled, the number of PK blood samples has been limited to 3, which have been carefully selected to yield the maximum information with the least possible number of blood samples. Pharmacokinetic sampling can be performed by venipuncture, or by heel or finger prick with collection of capillary blood. Alternatively they may be obtained through a venous or arterial line. It is permissible for investigators to use local anesthetics prior to sampling and investigators will be informed about this during the investigator meeting. Blood sampling for safety is also limited, with screening safety assessments in view of eligibility determination allowed to be performed using standard of care samples taken prior to study entry. Also, safety assessments performed as part of standard of care, when within the protocol visit window, can be used as for the safety follow-up during the study. Both of these measures aim at minimizing the number of specific sampling timepoints as well as blood volume drawn. Furthermore, the type of laboratory assessments for safety have been limited to the relevant ones in view of the (pre)clinical safety profile of lumicitabine, limiting the amount of blood needed and hence the duration of the sampling.

1.3.5. Overall Benefit/Risk Assessment

Currently the only available treatment for RSV is supportive care for infants and children requiring hospitalization. Based on the available data and proposed safety measures, the overall risk/benefit assessment for this study is acceptable for the following reasons:

- Continued careful study of the safety and PK is done in this study as well as evaluation of the preliminary efficacy of lumicitabine as an antiviral treatment for RSV.
- No safety concerns have previously been raised based on the safety information from healthy adult subjects and most observed AEs and laboratory abnormalities were mild to moderate in severity and considered not related to lumicitabine by the investigator (see Section 1.1, Background).
- Safety concerns have been raised based on the safety data from adult and pediatric subjects infected with RSV (see Section 1.1.2, Information on the Product, Subsection Clinical Studies). To reduce risk to subjects:
    - An IDMC will be established to monitor data in an unblinded manner on a regular basis to ensure the continuing safety of the subjects enrolled in this study.
    - JNJ-63549109 plasma target exposures for human subjects will be limited to a maximum average $AUC_{0\text{-}24h}$ of 20,000 ng.h/mL (or 25,200 ng.h/mL in whole blood) for any given dose. This threshold provides safety margins that are at least 2.7-fold lower than the lowest systemic NOAEL exposure obtained in nonclinical toxicity studies.

- Several safety measures have been proposed to minimize potential risk and stress to subjects, including:
  - Only subjects who meet all of the inclusion criteria and none of the exclusion criteria (as specified in the protocol) will be allowed to participate in this study. The selection criteria include adequate provisions to minimize the risk and protect the well-being of subjects in the study.
  - Subjects are to be hospitalized upon treatment initiation allowing for intensive monitoring.
  - Close monitoring of subjects throughout the study (see Section 17.9, Monitoring).
  - Customary measures taken by investigational staff to ensure that study-specific assessments such as blood sampling are performed with as little additional stress as possible for the subject.
  - Utilization of data obtained from standard of care samples.
  - Safety surveillance will be performed in a manner that minimizes the total number of required invasive procedures (eg, blood draws) to minimize discomfort to study subjects. Laboratory monitoring of hematologic parameters is included as a safety assessment in all clinical studies with lumicitabine. Careful follow-up by the investigators and the sponsor study physician with frequent oversight by the study-specific IDMCs will continue as planned.
  - Utilization of discontinuation and withdrawal criteria (see Section 10.2, Discontinuation of Study Treatment/Withdrawal From the Study).

2. OBJECTIVES, ENDPOINTS, AND HYPOTHESIS

2.1. Objectives and Endpoints

2.1.1. Objectives

Primary Objective

The primary objective is to determine in hospitalized infants and children who are infected with RSV the dose-response relationship of multiple regimens of lumicitabine on antiviral activity based on nasal RSV shedding using qRT-PCR.

Secondary Objectives

The secondary objectives are to determine in hospitalized infants and children who are infected with RSV:

- The safety and tolerability of lumicitabine.
- The PK of JNJ-63549109 in whole blood.
- The impact of lumicitabine on the clinical course of RSV infection.
- The impact of lumicitabine on the duration and severity of signs and symptoms of RSV infection as assessed by the Pediatric RSV Electronic Severity and Outcome Rating System (PRESORS) questionnaire completed by the clinician in the electronic clinical outcome assessment (eCOA) device.

- The impact of lumicitabine on the time to undetectable nasal RSV viral load.
- The impact of lumicitabine on the emergence of RSV strains with resistance-associated mutations.
- The relationship between the PK of JNJ−63549109 and the PD (antiviral activity, clinical symptoms, and selected safety parameters) after single (LD) and repeated oral dosing (MD) of lumicitabine.
- The acceptability and palatability of the lumicitabine formulation.

Exploratory Objectives

The exploratory objectives are to evaluate in hospitalized infants and children who are infected with RSV:

- The relationship between viral load and clinical outcome, including the relationship between RSV RNA levels and:
    - Requirement for and duration of supplemental oxygen.
    - Time to hospital discharge or readiness for hospital discharge, with readiness for discharge as evaluated by the investigator.
    - Time to clinical stability defined as the time from initiation of study treatment until the time at which the following criteria are met: return to pre-RSV infection status (hereafter referred to as "normalization") of blood oxygen level (without additional requirement of supplemental oxygen compared with pre-RSV infection status), normalization of oral feeding, normalization of respiratory rate, and normalization of heart rate.
- The relationship between viral load and duration and severity of signs and symptoms of RSV infection as assessed by the PRESORS questionnaire completed by the clinician in the eCOA device.
- The impact of the baseline viral subtype and genotype on the antiviral activity.
- The impact of lumicitabine on the duration and severity of signs and symptoms of RSV infection as assessed by the PRESORS questionnaire completed by the subject's parent(s)/caregiver(s) in the eCOA device.
- The relationship between the clinician eCOA and parent(s)/caregiver(s) eCOA responses.
- Medical resource utilization.
- The impact of lumicitabine on the infectious viral load using a quantitative culture of RSV (plaque assay) may be performed in a central laboratory at timepoints selected by the virologist.
- The comparison of the RSV viral loads measured in the mid-turbinate nasal swabs and endotracheal samples from intubated subjects.

2.1.2. Endpoints

Primary Endpoint

The primary endpoint is RSV viral load (measured by qRT-PCR in the mid-turbinate nasal swab specimens) AUC from immediately prior to first dose of study drug (baseline) until Day 7.

Secondary Endpoints

The secondary endpoints are:

- Safety/tolerability including AEs, physical examinations, vital signs/peripheral capillary oxygen saturation (SpO_2), ECGs, and clinical laboratory results.
- PK parameters of JNJ-63549109.
- RSV clinical course endpoints:
  - Length of hospital stay from admission to discharge and to readiness for discharge and from study treatment initiation to discharge and to readiness for discharge, with readiness for discharge evaluated by the investigator.
  - Requirement for and duration of intensive care unit (ICU) stay.
  - Requirement for and duration of oxygen supplementation/noninvasive mechanical ventilation support (eg, nasal cannula, face mask, continuous positive airway pressure) and/or invasive mechanical ventilation support (eg, endotracheal-mechanical ventilation or mechanical ventilation via tracheostomy) above pre-RSV infection status.
  - Time to no longer requiring supplemental oxygen above pre-RSV infection status.
  - Time to clinical stability defined as the time from initiation of study treatment until the time at which the following criteria are met: return to pre-RSV infection status (hereafter referred to as "normalization") of blood oxygen level (ie, without additional requirement of supplemental oxygen compared with pre-RSV infection status), normalization of oral feeding, normalization of respiratory rate, and normalization of heart rate.
  - Time from initiation of study treatment until SpO_2 ≥93% on room air among subjects who were not on supplemental oxygen prior to the onset of respiratory symptoms.
  - Time to respiratory rate, SpO_2, and body temperature return to pre-RSV infection status.
  - Incidence of acute otitis media (defined by the investigator).
- The duration and severity of signs and symptoms of RSV infection as assessed by the PRESORS questionnaire completed by the clinician in the eCOA device.
- RSV viral load as measured by qRT-PCR in the mid-turbinate nasal swab specimens, which will be used to determine the following:
  - RSV viral load over time.

- Peak viral load, time to peak viral load, rate of decline of viral load, and time to RSV RNA being undetectable.
- Proportion of subjects with undetectable RSV viral load at each timepoint.
- RSV viral load AUC from immediately prior to first dose of study drug (baseline) until Day 10 and until Day 14.
- RSV viral load AUC in subjects assigned to a longer dosing duration, if dosing duration is increased by the IDMC, from baseline until 1 day (+2 days) after the last dose of study drug.

- Sequence changes (postbaseline) in the RSV polymerase L-gene and other regions (if warranted) of the RSV genome compared with baseline sequences.
- Acceptability and palatability of the lumicitabine formulation as assessed by the parent(s)/caregiver(s) eCOA.

Exploratory Endpoints

The exploratory endpoints of this study are:

- The duration and severity of signs and symptoms of RSV infection as assessed by the PRESORS questionnaire completed by the parent(s)/caregiver(s) in the eCOA device.
- Number of hours the subject's parent(s)/caregiver(s) missed from usual activities.
- Medical resource utilization.
- RSV infectious viral load as measured using a quantitative viral culture (plaque assay).
- RSV viral load as measured by qRT-PCR of mid-turbinate nasal swabs and endotracheal samples in intubated subjects.

Refer to Section 9, Study Evaluations, for evaluations related to endpoints.

2.2. Hypothesis

The primary hypothesis of this study is that there is a positive dose-response relationship of active treatment on the average RSV viral load AUC over 7 days, meaning that either the average AUC on the pooled active treatments is lower than on placebo, or the average AUC on the high dose is lower than the average AUC on placebo, using multiple contrast testing.

3. STUDY DESIGN AND RATIONALE

3.1. Overview of Study Design

This is a randomized, double-blind, placebo-controlled, multicenter study of lumicitabine in hospitalized infants and children who are infected with RSV.

The selection of the design of this study (Option A or Option B) will be based on the number of subjects randomized and on the IDMC review of the data from the currently ongoing Study ALS-8176-503 of the subjects treated with 40 mg/kg LD/20 mg/kg MD lumicitabine dose regimen (Figure 1):

- **Option A** (Section 3.1.1)**:** This study design will be chosen if ≥9 subjects are randomized to the 40 mg/kg LD/20 mg/kg MD lumicitabine regimen in Study ALS-8176-503 (≥12 subjects in total) and if the IDMC recommends using the 60 mg/kg LD/40 mg/kg MD lumicitabine regimen based on their review of the data from Study ALS-8176-503. Based on the data available at the time of protocol writing, 60 mg/kg LD/40 mg/kg MD is the most likely dose regimen the IDMC will recommend. Option A will use the 40 mg/kg LD/20 mg/kg MD as the low lumicitabine dose and the 60 mg/kg LD/ 40 mg/kg MD (based on IDMC recommendation) as the high lumicitabine dose.
- **Option B** (Section 3.1.2): This study design will be chosen if <9 subjects are randomized to the 40 mg/kg LD/20 mg/kg MD lumicitabine regimen in Study ALS-8176-503 (<12 subjects in total) and thus no IDMC-recommended dose will be available at the start of the study. Option B will use the 40 mg/kg LD/20 mg/kg MD as the low lumicitabine dose. This study design option may also be selected once ≥9 subjects are randomized to the 40 mg/kg LD/20 mg/kg MD lumicitabine regimen in Study ALS-8176-503 (≥12 subjects in total) if:
    - Sponsor determines that additional doses should be evaluated.
    - IDMC-recommended lumicitabine dose is lower than the 60 mg/kg LD/40 mg/kg MD lumicitabine regimen.

Figure 1: Study Design: Option Selection

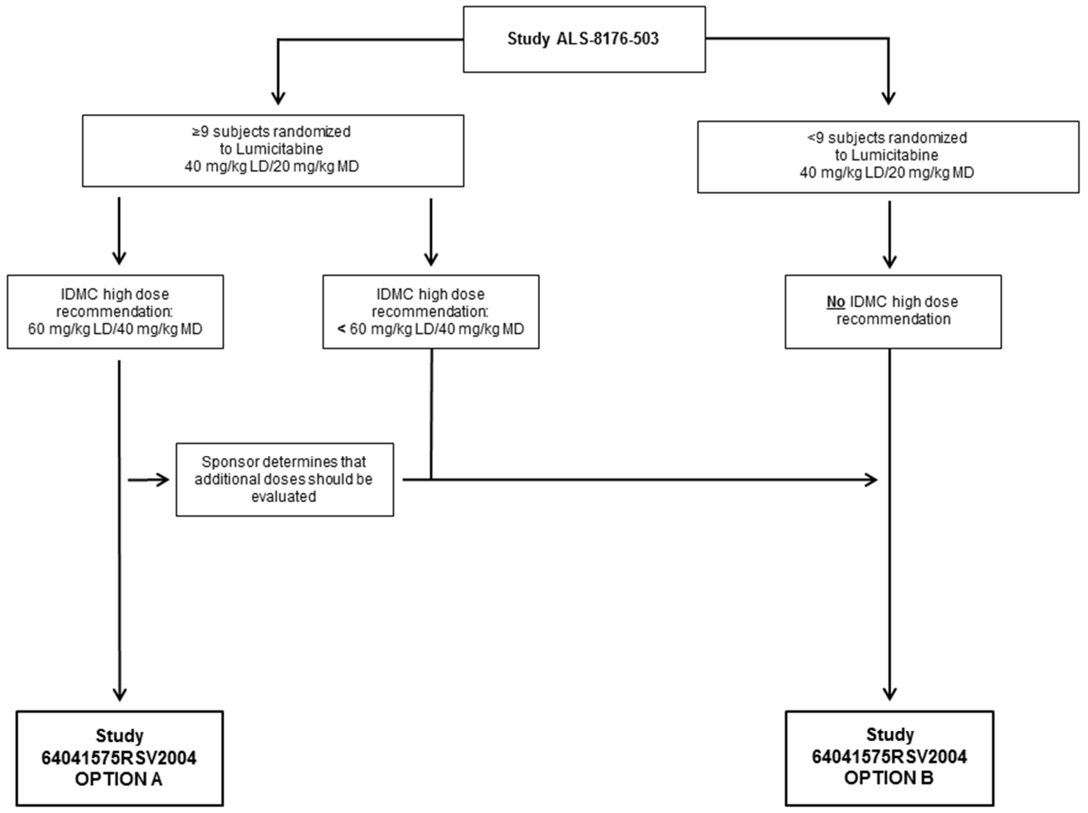

Abbreviations: IDMC: Independent Data Monitoring Committee; LD: loading dose; MD: maintenance dose.
Based on the data available at the time of protocol writing, 60 mg/kg LD/40 mg/kg MD is the most likely lumicitabine dose regimen the IDMC will recommend.

Please refer to Section 1.1.2, Information on the Product, for more details about Study ALS-8176-503.

3.1.1. Option A

Hospitalized infants and children who are infected with RSV will be randomly assigned to a treatment regimen in this study, with 60 subjects planned per regimen. At least 120 subjects are planned to be enrolled up to a maximum of 180 subjects. An effort will be made to enroll at least 90 subjects aged between 28 days and 24 months and at least 12 subjects aged between 24 and 36 months. The number of subjects with comorbidities will be limited to 20%; additional comorbid subjects aged between 24 and 36 months may be allowed to enroll if the 20% threshold is reached before 12 subjects aged between 24 and 36 months are enrolled into the study.

Subjects will be randomized in a 1:1:1 ratio to Regimen A, B, or C:

- Regimen A (low-dose lumicitabine): a single 40 mg/kg LD (Dose 1) followed by nine 20 mg/kg MDs (Doses 2 to 10) of lumicitabine, administered twice daily.
- Regimen B (high-dose lumicitabine): a single 60 mg/kg LD (Dose 1) followed by nine 40 mg/kg MDs (Doses 2 to 10) of lumicitabine, administered twice daily.

- Regimen C (placebo): those randomized to a placebo regimen are subsequently randomized in a 1:1 ratio (to match volumes) to either:
  - A single 40 mg/kg placebo LD (Dose 1) followed by nine 20 mg/kg MDs (Doses 2 to 10), administered twice daily.
  - A single 60 mg/kg placebo LD (Dose 1) followed by nine 40 mg/kg placebo MDs (Doses 2 to 10), administered twice daily.

An IDMC (refer to Section 11.10, Independent Data Monitoring Committee, for details) will review the safety and PK data once the first 12 subjects and 30 subjects have completed treatment (Day 5/6) to assess if enrollment of additional subjects may safely continue in each arm. After at least 9 subjects have completed treatment with the 60 mg/kg LD/40 mg/kg MD lumicitabine regimen, the IDMC may recommend that sparse PK sampling be used for the other subjects. Subject recruitment and enrollment may continue while the data are being reviewed.

Subjects who are admitted to hospital due to RSV infection, including those who are otherwise healthy and those with underlying comorbidities (prematurity at birth [subject's gestational age was <37 weeks; for infants <1 year old at randomization], bronchopulmonary dysplasia, congenital heart disease, other congenital diseases, Down syndrome, neuromuscular impairment, or cystic fibrosis) can participate. However, enrollment of subjects with comorbidities can only start after at least 9 subjects have completed treatment with the 60 mg/kg LD/40 mg/kg MD lumicitabine regimen, after their safety and PK data have been reviewed by the IDMC, and after the IDMC has recommended that the recruitment of subjects with underlying comorbidities may start. Screening should be completed as soon as possible and the subject randomized within ≤5 days of RSV symptom onset. Procedures that are standard of care and performed within approximately 72 hours prior to randomization may be used in determining study eligibility or determining baseline values.

Randomization will be stratified by duration of RSV symptoms from onset till time of randomization (≤3 days; >3 days to ≤5 days), and by presence or absence of at least 1 comorbid condition for severe RSV disease (prematurity at birth [subject's gestational age was <37 weeks; for infants <1 year old at randomization], bronchopulmonary dysplasia, congenital heart disease, other congenital diseases, Down syndrome, neuromuscular impairment, or cystic fibrosis).

Treatment with lumicitabine should be initiated as soon as possible but no later than 4 hours after randomization to maximize the opportunity for the compound to inhibit viral replication and potentially improve outcomes. The parent(s)/caregiver(s) will administer the study drug at home until all doses are administered if the subject is discharged prior to the completion of treatment. Subjects will be evaluated for a total of 28 days postrandomization. Depending on the discharge date, assessments will be completed either in the hospital or during outpatient visits. On Day 28, all subjects will complete the study either as an inpatient or outpatient. The total study duration for each subject will be 28 days, screening phase not included.

The doses will not exceed those resulting in an expected average JNJ-63549109 plasma $AUC_{0\text{-}24h}$ of 20,000 ng.h/mL (or 25,200 ng.h/mL in whole blood), a limit that is 2.7-fold lower than the lowest systemic NOAEL observed in nonclinical toxicity studies.

Assessments performed during the study will include: antiviral activity and an evaluation of the clinical course of RSV infection; safety and tolerability assessments (AEs, laboratory assessments, ECGs, physical examination, and vital signs/SpO_2); PK assessments based on sparse sampling will be performed using a popPK model; sequence analysis of the L-gene to identify preexisting sequence polymorphisms, to characterize RSV variants and other regions of the RSV genome if warranted, and to evaluate emergence of any resistance-associated mutations; and exploratory biomarkers to determine the effects of lumicitabine on markers of RSV disease.

Given the challenges of assuring study-site preparation in time to recruit for an acute seasonal infection like RSV and the delays caused by implementing an amendment during an RSV season, the duration of dosing of lumicitabine may be modified by recommendation of the IDMC based on the review of this study and data from other ongoing studies of lumicitabine.

Up to 2 formal interim analyses may be performed during the study. The first interim analysis will be performed once 36 subjects with ≤3 days of RSV symptoms have completed treatment (Day 5/6) or once 90 subjects with >3 to ≤5 days of RSV symptoms have been enrolled and have completed treatment. The following situations will be considered based on the results at this stage: that the study is considered futile; that superiority can be concluded; that subjects with >3 to ≤5 days of RSV symptoms at randomization may not achieve benefit (which may also be limited to ≤4 days of onset to symptoms); or that the study can continue unchanged. The treatment regimen may be modified after the first interim analysis, including increasing the treatment duration up to 10 days. After this analysis, randomization to 1 of the lumicitabine doses may be discontinued, the randomization ratio may change, and the study may not continue in subjects with >3 to ≤5 days of RSV symptoms at randomization. The second interim analysis may be performed at the discretion of the sponsor once at least 120 subjects have completed treatment (Day 5/6) (or at least 60 subjects with ≤3 days of RSV symptoms). The following situations will be considered based on the results at this stage: that the study is considered futile; that superiority can be concluded; or that the study can continue unchanged. Based on the recommendations of the IDMC following these interim analyses/reviews of PK, efficacy, and safety data, changes to randomization ratios in the treatment arms or an increase in treatment duration to 10 days may be implemented.

The design for Option A is provided in Figure 2.

Figure 2: Schematic Study Overview: Option A

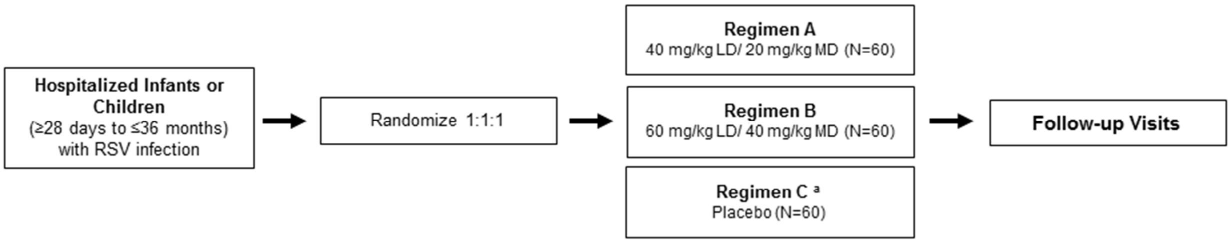

Abbreviations: LD: loading dose; MD: maintenance dose; RSV: respiratory syncytial virus.

a. Subjects randomized to placebo will be subsequently randomized in a 1:1 ratio to the placebo regimen matching Regimen A or Regimen B.

3.1.2. Option B

Hospitalized infants or children who are infected with RSV will be sequentially enrolled into different cohorts and will be randomized to receive either lumicitabine or placebo. At least 120 subjects are planned to be enrolled up to a maximum of 180 subjects. An effort will be made to enroll at least 90 subjects aged between 28 days and 24 months and at least 12 subjects aged between 24 and 36 months. The number of subjects with comorbidities will be limited to 20%; additional comorbid subjects aged between 24 and 36 months may be allowed to enroll if the 20% threshold is reached before 12 subjects aged between 24 and 36 months are enrolled into the study.

Subjects will be enrolled into Cohort 1 and randomized in a 2:1 ratio to lumicitabine Regimen 1 or placebo:

- Lumicitabine Regimen 1: a single 40 mg/kg LD followed by nine 20 mg/kg MDs of lumicitabine, administered twice daily.
- Placebo: a single 40 mg/kg LD followed by nine 20 mg/kg MDs of placebo, administered twice daily.

An IDMC will review the safety and PK data once at least 9 subjects in Cohort 1 have completed treatment (Day 5/6) with the 40 mg/kg LD/20 mg/kg MD lumicitabine regimen (including subjects recruited in Study ALS-8176-503) to assess if recruitment to a new treatment regimen (Cohort 2) may safely start. Subject recruitment and enrollment for Cohort 1 may continue while the data are being reviewed.

The sponsor may skip recruitment into Cohort 1 (ie, no subjects would be enrolled into Cohort 1) and recruit subjects directly into Cohort 2 if ≥9 subjects have been treated with the 40 mg/kg LD/20 mg/kg MD lumicitabine regimen in Study ALS-8176-503 (≥12 subjects in total) and if those data have been reviewed by the IDMC.

Subjects will be enrolled into Cohort 2 and randomized in a 1:1:1 ratio to lumicitabine Regimen 1, lumicitabine Regimen 2, or placebo:

- Lumicitabine Regimen 1: a single 40 mg/kg LD followed by nine 20 mg/kg MDs of lumicitabine, administered twice daily.
- Lumicitabine Regimen 2: a single LD followed by 9 MDs of lumicitabine Regimen 2, administered twice daily. The IDMC will provide a dose recommendation for Regimen 2.
- Placebo: those randomized to a placebo regimen are subsequently randomized in a 1:1 ratio (to match volumes) to either:
    - A single 40 mg/kg placebo LD (Dose 1) followed by nine 20 mg/kg MDs (Doses 2 to 10) of placebo, administered twice daily.
    - A single placebo LD (Dose 1) followed by nine MDs (Doses 2 to 10) of placebo matching Regimen 2, administered twice daily.

An IDMC will review the safety and PK data from Cohort 2 once at least 9 subjects in Cohort 2 have completed treatment (Day 5/6) with the active lumicitabine Regimen 2. Subject recruitment and enrollment for Cohort 2 may continue while the data are being reviewed.

A third dose may be evaluated, upon IDMC recommendation, based on the emerging safety and PK profile. Subjects will be enrolled into Cohort 3 and randomized in a 1:3:2 ratio to lumicitabine Regimen 1, lumicitabine Regimen 3, or placebo:

- Lumicitabine Regimen 1: a single 40 mg/kg LD followed by nine 20 mg/kg MDs of lumicitabine, administered twice daily.
- Lumicitabine Regimen 3: a single LD followed by 9 MDs of lumicitabine Regimen 3, administered twice daily. The IDMC will provide a dose recommendation for the lumicitabine Regimen 3.
- Placebo: Those randomized to a placebo regimen are subsequently randomized in a 1:3 ratio (to match volumes) to either:
    - A single 40 mg/kg placebo LD (Dose 1) followed by nine 20 mg/kg MDs (Doses 2 to 10) of placebo, administered twice daily.
    - A single placebo LD (Dose 1) followed by nine MDs (Doses 2 to 10) of placebo matching Regimen 3, administered twice daily.

An IDMC will review safety and PK data from Cohort 3 once 9 subjects in that cohort have completed treatment (Day 5/6) with the active lumicitabine Regimen 3, at which stage the IDMC will select either lumicitabine Regimen 2 or lumicitabine Regimen 3 to be used with placebo and lumicitabine Regimen 1 for the remainder of the study. Depending on the selected doses, other randomization schemes may be selected for the remainder of the study. Subject recruitment and enrollment for Cohort 3 may continue while the data are being reviewed. Subjects will continue to be enrolled and treated in Cohort 2 up to a maximum of 180 subjects if a third dose regimen is not evaluated.

The sponsor/IDMC may recommend that the cohort regimens be adjusted or removed, given the active nature of the development program and the ongoing assessment of all the available data.

Subjects who are admitted to hospital due to RSV infection, including those who are otherwise healthy and those with underlying comorbidities (prematurity at birth [subject's gestational age was <37 weeks; for infants <1 year old at randomization], bronchopulmonary dysplasia, congenital heart disease, other congenital diseases, Down syndrome, neuromuscular impairment, or cystic fibrosis) can participate. However, enrollment of subjects with comorbidities can only start once at least 9 subjects in Cohort 2 have completed treatment with the active lumicitabine Regimen 2 and after their safety and PK data have been reviewed by the IDMC. If subjects are enrolled into Cohort 3, then enrollment of subjects with comorbidities can only start once at least 9 subjects in this cohort have completed treatment with the active lumicitabine Regimen 3 and after their safety and PK data have been reviewed by the IDMC. Screening should be completed as soon as possible and the subject randomized within ≤5 days of RSV symptom onset.

Procedures that are standard of care and performed within approximately 72 hours prior to randomization may be used in determining study eligibility or determining baseline values.

Randomization will be stratified by duration of RSV symptoms from onset till time of randomization (≤3 days; >3 days to ≤5 days), and by presence or absence of at least 1 comorbid condition for severe RSV disease (prematurity at birth [subject's gestational age was <37 weeks; for infants <1 year old at randomization], bronchopulmonary dysplasia, congenital heart disease, other congenital diseases, Down syndrome, neuromuscular impairment, or cystic fibrosis).

Treatment with lumicitabine should be initiated as soon as possible but no later than 4 hours after randomization to maximize the opportunity for the compound to inhibit viral replication and potentially improve outcomes. The parent(s)/caregiver(s) will administer the study drug at home until all doses are administered if the subject is discharged prior to the completion of treatment. Subjects will be evaluated for a total of 28 days postrandomization. Depending on the discharge date, assessments will be completed either in the hospital or during outpatient visits. On Day 28, all subjects will complete the study either as an inpatient or outpatient. The total study duration for each subject will be 28 days, screening phase not included.

The doses will not exceed those resulting in an expected average JNJ-63549109 plasma $AUC_{0\text{-}24h}$ of 20,000 ng.h/mL (or 25,200 ng.h/mL in whole blood), a limit that is 2.7-fold lower than the lowest systemic NOAEL observed in nonclinical toxicity studies.

Assessments performed during the study will include: antiviral activity and an evaluation of the clinical course of RSV infection; safety and tolerability assessments (AEs, laboratory assessments, ECGs, physical examination, and vital signs/SpO_2); PK assessments based on sparse sampling will be performed using a popPK model; sequence analysis of the L-gene to identify preexisting sequence polymorphisms, to characterize RSV variants and other regions of the RSV genome if warranted, and to evaluate emergence of any resistance-associated mutations; and exploratory biomarkers to determine the effects of lumicitabine on markers of RSV disease.

Given the challenges of assuring study-site preparation in time to recruit for an acute seasonal infection like RSV and the delays caused by implementing an amendment during an RSV season, the duration of dosing of lumicitabine may be modified by recommendation of the IDMC based on the review of this study and data from other ongoing studies of lumicitabine.

Up to 2 formal interim analyses will be performed during the study. The first interim analysis will be performed once 36 subjects with ≤3 days of RSV symptoms have completed treatment or once 90 subjects with >3 to ≤5 days of RSV symptoms have been enrolled and have completed treatment. The following situations will be considered based on the data analysis at this stage: that the study is considered futile; that superiority can be concluded; that subjects with >3 to ≤5 days of RSV symptoms at randomization may not achieve benefit (which may also be limited to ≤4 days of onset to symptoms); or that the study can continue unchanged. The treatment regimen may be modified after the first interim analysis, including increasing the treatment duration up to 10 days. After this analysis, randomization to 1 of the lumicitabine doses may be discontinued, the randomization ratios may change, and the study may not continue in subjects

with >3 to ≤5 days of RSV symptoms at randomization. The second interim analysis may be performed at the discretion of the sponsor once at least 120 subjects have completed treatment (Day 5/6) (or 60 subjects with ≤3 days of RSV symptoms). The following situations will be considered based on the data analysis at this stage: that the study is considered futile; that superiority can be concluded; or that the study can continue unchanged. Based on the recommendations of the IDMC following these interim analyses/reviews of PK, efficacy, and safety data, changes to randomization ratios in the treatment arms or an increase in treatment duration to 10 days may be implemented.

The design for Option B is provided in Figure 3.

Figure 3: Schematic Study Overview: Option B

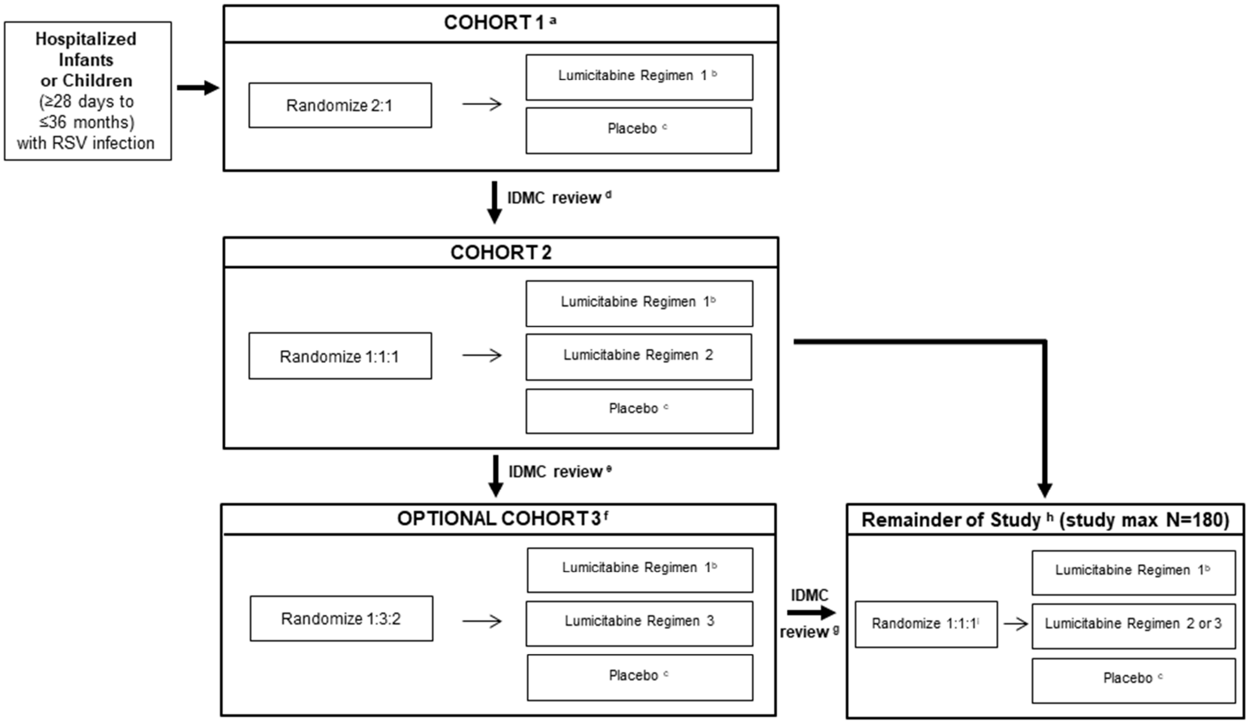

Abbreviations: IDMC: Independent Data Monitoring Committee; LD: loading dose; max: maximum; MD: maintenance dose; N: number of subjects; PK: pharmacokinetic(s); RSV: respiratory syncytial virus.

The sponsor/IDMC may recommend that the cohort regimens be adjusted or removed, given the active nature of the development program and the ongoing assessment of all the available data. The number of subjects that needs to be recruited to each cohort will depend on the doses used and on the number of subjects recruited in Study ALS-8176-503.

a. The sponsor may skip recruitment into Cohort 1 (ie, no subjects would be enrolled into Cohort 1) and recruit subjects directly into Cohort 2 if ≥9 subjects have been treated with the 40 mg/kg LD/20 mg/kg MD lumicitabine regimen in Study ALS-8176-503 (≥12 subjects in total) and if those data have been reviewed by the IDMC.
b. Lumicitabine Regimen 1: a single 40 mg/kg LD followed by nine 20 mg/kg MDs of lumicitabine, administered twice daily.
c. Subjects randomized to placebo will be subsequently randomized in to a placebo regimen matching the active lumicitabine regimen(s).
d. An IDMC will review the safety and PK data of Cohort 1 once at least 9 subjects in that cohort have completed treatment with the 40 mg/kg LD/20 mg/kg MD lumicitabine regimen (Regimen 1). Subject recruitment and enrollment for Cohort 1 may continue while the data are being reviewed.
e. An IDMC will review the safety and PK data of Cohort 2 once at least 9 subjects in that cohort have completed treatment with the active lumicitabine Regimen 2.
f. A third dose may be evaluated upon IDMC recommendation based on the emerging safety and PK profile.
g. An IDMC will review the safety and PK data from Cohort 3 once at least 9 subjects in that cohort have completed treatment with the active lumicitabine Regimen 3, at which stage the IDMC will select either lumicitabine Regimen 2 or lumicitabine Regimen 3 to be used with placebo and lumicitabine Regimen 1 for the remainder of the study.
h. Subjects will continue to be enrolled and treated in Cohort 2 up to a maximum of 180 subjects if a third dose regimen is not evaluated.
i. The IDMC may recommend changing the randomization ratio for the remainder during the study.

3.2. Study Design Rationale

Blinding, Control, Randomization, and Stratification

There is no approved treatment routinely used for RSV infection. A placebo control will be used to establish the frequency and magnitude of changes in virologic and clinical endpoints that may occur in the absence of active treatment. The use of a placebo control will allow for any AEs or laboratory abnormalities observed during the course of the study to be evaluated properly ie, to be able to differentiate between events potentially related to the use of lumicitabine versus those related to the underlying disease.

Randomization will be used to minimize bias in the assignment of subjects to treatment regimens, to increase the likelihood that known and unknown subject attributes (eg, demographic and baseline characteristics) are evenly balanced across treatment regimens, and to enhance the validity of statistical comparisons across treatment regimens.

Randomization will be stratified by duration of RSV symptoms from onset till time of randomization (≤3 days; >3 days to ≤5 days), and by presence or absence of at least 1 comorbid condition for severe RSV disease (prematurity at birth [subject's gestational age was <37 weeks; for infants <1 year old at randomization], bronchopulmonary dysplasia, congenital heart disease, other congenital diseases, Down syndrome, neuromuscular impairment, or cystic fibrosis). Presence or absence of a comorbid condition is selected as a stratification factor due to the more severe nature of RSV infections in subjects with these conditions. Duration of symptoms onset is selected because it is anticipated that there will be a larger treatment effect in subjects with onset of symptoms ≤3 days. Stratification on duration of symptom onset will allow a well-balanced assessment to be made at the first interim analysis if the treatment effect depends on the onset of RSV symptoms.

Blinded treatment will be used to reduce potential bias during data collection and evaluation of clinical endpoints.

Doses Selected

The doses selected for this study are based on PK/PD modeling, which was developed to characterize the in vivo antiviral activity of lumicitabine, based on the data from the RSV challenge study in healthy adult subjects (Study ALS-8176-502) and on the interim data from a study in infants hospitalized with RSV infection (Study ALS-8176-503). The model was used to characterize the exposure-response relationships of JNJ-63549109 in adults, and then scaled to pediatrics to account for differences in PK. To characterize the exposure-response relationships, a semi-mechanistic model was utilized to describe the conversion of plasma JNJ-63549109 to the active NTP in lung tissue. The model was fit simultaneously to the PK and viral kinetic data for both placebo- and lumicitabine-receiving subjects from Study ALS-8176-502. A sigmoid maximum efficacy relationship described the NTP inhibition of RSV production. The model estimated the NTP 50% effective concentration and 90% effective concentration to be 1.79 μM and 2.64 μM, respectively, which is generally consistent with the reported in vitro half maximal inhibitory concentration (0.23 μM) and 90% inhibitory concentration (3 μM) against a different strain of RSV. To scale these results to pediatric subjects, simulations were conducted using a popPK model developed for adults and pediatric subjects, to estimate pediatric doses that would result in NTP lung exposures exceeding the model-determined 90% effective concentration (EC_{90}) and 99% of maximal effective concentration and $3 \times EC_{90}$ targets, and that would be similar to adult exposures. The proposed dose regimens are projected to rapidly (within 1 to 2 hours following dosing) achieve and maintain lung tissue concentrations at least $3 \times EC_{90}$ throughout the duration of treatment in the majority of the subjects.

Because RSV is an acute infection, achieving therapeutic concentrations rapidly is an important consideration for treatment. Therefore, LD regimens were evaluated, considering not only achievement of the above targets, but also time to achieve these targets. To account for popPK variability, the probability of and time to target attainment across the population (median and 95% percentiles) were also considered. The assessment of twice daily dose regimens in the study is supported by the long in vitro intracellular $t_{1/2}$ of the NTP (17.6 hours in normal human bronchial epithelial cells) and the observed sustained levels of the NTP in monkey lungs (estimated $t_{1/2}$ of approximately 29 hours).

Overall, based on the currently available data, it is expected that the proposed dose regimens are in the effective range of lumicitabine in infants and children, which includes a single 40 mg/kg LD followed by an MD regimen of nine 20 mg/kg doses given twice daily or a single 60 mg/kg LD followed by an MD regimen of nine 40 mg/kg doses given twice daily. At the proposed dose of 40 mg/kg LD/20 mg/kg MD, plasma JNJ-63549109 exposures in infants and children are projected to be at or slightly above levels observed in healthy adult subjects in the RSV challenge study where potent antiviral activity was noted ($AUC_{0\text{-}24h}$ approximately 10,000 ng.h/mL, ALS-8176-502) (Table 3). Based on the current popPK model and simulations, a viable high dose regimen is 60 mg/kg LD followed by nine 40 mg/kg MDs given twice daily as it provides plasma exposures (mean $AUC_{0\text{-}24h}$) that are projected to be <20,000 ng.h/mL for all

age groups (or 25,200 ng.h/mL in whole blood). Simulations of the typical lung NTP exposure showed that both dose regimens rapidly achieved concentrations above EC_{90} (the highest concentration required to achieve 90% inhibition of virus replication across all RSV strains tested in vitro).

Table 3: Projected Plasma Exposures of JNJ-63549109 at the Proposed Doses

Dose (mg/kg)		Age Group (Months)	Average Plasma $AUC_{0\text{-}24h}$ JNJ-63549109 (ng.h/mL)	
LD	MD		Day 1	Day 5
40	20	1 to <2	8,295	8,438
		2 to <6	9,712	9,504
		6 to <12	10,740	10,055
		12 to ≤36	12,100	10,948
60	40	1 to <2	12,846	14,713
		2 to <6	14,984	16,525
		6 to <12	16,492	17,417
		12 to ≤36	18,514	18,923

Abbreviations: $AUC_{0\text{-}24h}$: area under the plasma concentration versus time curve from time 0 to 24 hours after dosing; LD: loading dose; MD: maintenance dose.

It is important to note that these projections are based on predicted pediatric PK, and will continue to be revised and updated as pediatric data are collected in the ongoing Study ALS-8176-503. Given the active nature of the ongoing development program, the IDMC may recommend that dose regimens be removed or adjusted based on safety and/or viral activity considerations from ongoing studies. Irrespective of the dose, the average projected JNJ-63549109 plasma exposure (Table 3) is expected not to exceed a projected average $AUC_{0\text{-}24h}$ of 20,000 ng.h/mL (or 25,200 ng.h/mL in whole blood), a limit that is 2.7-fold lower than the lowest systemic NOAEL observed in nonclinical toxicity studies.

The active regimens evaluated in this design will be selected to provide a range of plasma JNJ-63549109 and intracellular JNJ-63549109-nucleoside triphosphate exposures aimed at providing information on efficacy, safety, tolerability, and PK in the target population of infants and children who are aged ≥28 days to ≤36 months and who are hospitalized with RSV.

Treatment Duration

Treatment will last for 5 days as this was the duration of treatment assessed in healthy adult subjects infected with RSV in a human challenge model (Study ALS-8176-502), which demonstrated substantial declines in RSV viral load and improvement in RSV signs and symptoms (see Section 1.1). Five days is also the duration being studied in the MAD part of Study ALS-8176-503 in pediatric subjects. The long terminal $t_{1/2}$ of the NTP is anticipated to provide therapeutic levels of the active NTP beyond the 5-day treatment duration. The treatment duration may be extended to up to 10 days if recommended by the IDMC, based on emerging data from ongoing clinical studies with lumicitabine.

Clinical Outcomes of RSV Treatment

Treatment with lumicitabine may reduce the severity and duration of RSV signs and symptoms and their impact on functioning, reduce the need for and duration of supportive care (eg, oxygen supplementation, intravenous (IV) fluids, days of hospitalization), and accelerate the subjects' return to pre-RSV health status. The study will compare treatment regimens to evaluate the impact of treatment with lumicitabine on the clinical course of RSV disease.

Pharmacokinetic Sampling Schedule

To maximize the information and design of ongoing and future pediatric studies where PK samples are collected, optimal sampling strategies have been utilized. The approach for PK sample optimization utilizes the population Fisher information matrix software package (Version 4), which is specifically designed for optimizing sampling strategies for nonlinear mixed-effects models.[4] Briefly, the popPK model for lumicitabine is put into the software package and optimal sampling timepoints for key model parameters are derived. The Fisher information matrix uses D-optimality criteria for design optimization, where a design is considered to be optimal if it maximizes the determinant of population Fischer information matrix, resulting in minimization of the confidence region for parameter estimates. In addition to optimizing sampling times based on information content, logistics and feasibility are also considered as part of the final PK timepoint selection. Based on these criteria, PK samples will be taken in this study, where sampling will be done in conjunction with other laboratory assessments when possible to further minimize blood collection; each PK sample only requires a minimal volume of blood.

Biomarker Collection

Biomarker samples can be obtained from leftover mid-turbinate nasal swab samples and blood samples collected for PK testing to evaluate the role biomarkers (eg, proteins including cytokines) may play in the treatment response, safety of lumicitabine, or the status and change of the RSV-related disease. They may help to explain interindividual variability in clinical outcomes or to identify population subgroups that respond differently to a drug.

Biomarker samples may be used to help address emerging issues and to enable the development of safer, more effective, and ultimately individualized therapies.

Biomarker analyses may be performed upon the discretion of the sponsor and will be reported separately.

Medical Resource Utilization and Health Economics Data Collection

Treatment with lumicitabine may result in lower utilization of hospital or outpatient healthcare services.

4. SUBJECT POPULATION

Screening for eligible subjects will be performed as soon as possible following admission to the ER or hospitalization, such that subjects are randomized within 5 days of RSV symptom onset. Special attention will be paid to determine the duration of RSV symptoms from onset till time of randomization as accurately as possible. Male or female infants and children ≥28 days to ≤36 months of age diagnosed with RSV infection based on a PCR-based diagnostic assay (with or without coinfection with another respiratory pathogen) who have been (or will be) admitted to the hospital and whose parents have signed informed consent will be enrolled. Subjects who were admitted to the hospital for another reason but develop an acute respiratory illness while being hospitalized are also eligible for screening.

The inclusion and exclusion criteria for enrolling subjects in this study are described in the following 2 subsections. If there is a question about the inclusion or exclusion criteria below, the investigator must consult with the appropriate sponsor representative and resolve any issues before enrolling a subject in the study. Waivers are not allowed.

For a discussion of the statistical considerations of subject selection, refer to Section 11.2, Sample Size Determination.

4.1. Inclusion Criteria

Each potential subject must satisfy all of the following criteria to be enrolled in the study:

1. Male or female infants and children ≥28 days to ≤36 months of age, defined at the time of randomization.

2. Subjects hospitalized (or in ER) at the time of randomization and unlikely to be discharged for the first 24 hours after randomization.

3. Subjects diagnosed with RSV infection using a PCR-based molecular diagnostic assay, with or without coinfection with another respiratory pathogen (respiratory virus or bacteria).

   NOTE: In cases where commercial PCR-based assays are not available at the site, the sponsor should be consulted for agreement on the assay to be used.

4. Subjects who have an acute respiratory illness with signs and symptoms consistent with a viral infection (eg, fever, cough, nasal congestion, runny nose, sore throat, myalgia, lethargy, shortness of breath, or wheezing) with onset ≤5 days from the anticipated time of randomization. Onset of symptoms is defined as the first time (within 1 hour) the parent(s)/caregiver(s) becomes aware of respiratory or systemic symptoms of RSV infection.

5. With the exception of the symptoms related to the RSV infection or defined comorbid condition for severe RSV disease (prematurity at birth [subject's gestational age was <37 weeks; for infants <1 year old at randomization], bronchopulmonary dysplasia,

congenital heart disease, other congenital diseases, Down syndrome, neuromuscular impairment, or cystic fibrosis), subjects must be medically stable on the basis of physical examination, medical history, vital signs/SpO_2, and ECG performed at screening. If there are abnormalities, they must be consistent with the underlying condition in the study population and/or the RSV infection. This determination must be recorded in the subject's source documents and initialed by the investigator. Subjects with comorbidities will be allowed to be enrolled once the IDMC has reviewed the PK and safety data of the highest dose that will be used in this study and once the IDMC has recommended opening recruitment to this group. Sites will be notified when the restriction is lifted.

6. Subjects must be medically stable on the basis of clinical laboratory tests performed at screening. If the results of the serum chemistry panel or hematology are outside the normal reference ranges, the subject may be included only if the investigator judges the abnormalities or deviations from normal do not pose an unacceptable risk to the subject, are not clinically significant, or are appropriate and reasonable for the population under study, and have no specific cutoff per the exclusion criteria. This determination must be recorded in the subject's source documents and initialed by the investigator. A single repeat laboratory evaluation is allowed for eligibility determination.

7. The subject's estimated glomerular filtration rate (eGFR) is not below the lower limit of normal for the subject's age (Schwartz equation calculation see Attachment 3).

8. In the investigator's opinion, the subject's legally acceptable representative understands and is able to comply with protocol requirements, instructions, and protocol-stated restrictions, and the subject is likely to complete the study as planned.

9. The subject's legally acceptable representative must sign an informed consent form (ICF) indicating that he or she understands the purpose of, and procedures required for, the study and is willing for the subject to participate in the study.

4.2. Exclusion Criteria

Any potential subject who meets any of the following criteria will be excluded from participating in the study:

1. Subjects who are not expected to survive for more than 48 hours.

2. Subjects who have had major thoracic or abdominal surgery in the 6 weeks prior to randomization.

3. Subjects who received (within 12 months prior to screening) or who are currently on a waiting list for a bone marrow, stem cell, or solid organ transplant, who received radiation or chemotherapy (within 12 months prior to screening), or who are currently taking immunosuppressive medication (refer to Section 8, Pre-study and Concomitant Therapy).

4. Subjects who have a known or suspected immunodeficiency (except immunoglobulin A [IgA] deficiency), such as a known human immunodeficiency virus infection.

5. Subjects who have a history of or concurrent illness (beyond a defined comorbid condition for severe RSV disease) that in the opinion of the investigator, might confound the results of the study or pose an additional risk in administering study drug to the subject, or that could prevent, limit, or confound the protocol-specified assessments such as liver or renal insufficiency; significant cardiac, vascular, pulmonary, gastrointestinal, endocrine, neurologic, hematologic, rheumatologic, metabolic disturbances, or genetic diseases that result in immunosuppression. These may include, but are not limited to bacteremia, organ dysfunction, or other severe comorbidities.

6. Subjects who have a poorly functioning gastrointestinal tract (ie, unable to absorb drugs or nutrition via enteral route).

   NOTE: The use of IV fluids is not exclusionary so long as the investigator believes the subject's gastrointestinal tract still functions properly (ie, is able to absorb drugs or nutrition).

7. Subjects being treated with extracorporeal membrane oxygenation.

8. Subject receiving chronic oxygen therapy at home prior to admission.

9. Subjects who have taken any disallowed therapies as noted in Section 8, Prestudy and Concomitant Therapy, before the planned first dose of study drug.

10. Subjects who received prescription medications intended to prevent or treat the RSV infection itself (eg, ribavirin, IV immunoglobulin, palivizumab), an investigational drug, an investigational vaccine, or used an invasive investigational medical device within 30 days or 5 half-lives (whichever is longer) before the planned first dose of study drug, or is currently enrolled in an investigational study.

11. Subjects who have known allergies, hypersensitivity, or intolerance to lumicitabine or its excipients (refer to Investigator's Brochure).[16]

12. Subjects legally acceptable representative unwilling to have subject undergo mid-turbinate nasal swab procedures or subjects with any physical abnormality which limits the ability to collect nasal swabs.

13. Subjects who are being breastfed by a mother taking any of the excluded medications as noted in Section 8, Prestudy and Concomitant Therapy.

14. Subject's legally acceptable representative ie, parent(s)/legal guardian/caregiver(s), is not able to communicate reliably with the investigator.

15. Subjects with any condition for which, in the opinion of the investigator, participation would not be in the best interest of the subject (eg, compromise the well-being) or that could prevent, limit, or confound the protocol-specified assessments.

16. Subjects aged ≥28 days to 35 days with <8.0 g/dL (Grade 3) hemoglobin or a cardiac failure secondary to anemia (Grade 4) and subjects aged ≥36 days to 36 months with <7.0 g/dL (Grade 3) hemoglobin or cardiac failure secondary to anemia (Grade 4).

17. Subjects aged ≥28 days to 60 days with <899/mm^3 absolute neutrophil count) and subjects aged ≥61 days to ≤36 months with <399/mm^3 absolute neutrophil count.

18. Subjects aged ≥28 days to ≤36 months with <49,999/mm^3 platelet count (Grade 3/4).

NOTE: Investigators should ensure that all study enrollment criteria have been met at screening. If a subject's clinical status changes (including any available laboratory results or receipt of additional medical records) after screening but before the first dose of study drug is given such that he or she no longer meets all eligibility criteria, then the subject should be excluded from participation in the study. Section 9.1.2, Screening Phase, describes options for retesting. Section 17.4, Source Documentation, describes the required documentation to support meeting the enrollment criteria.

4.3. Prohibitions and Restrictions

Parent(s)/caregiver(s) must be willing and able to adhere to the following prohibitions and restrictions during the course of the study for the subject to be eligible for participation:

1. Refer to Section 8, Prestudy and Concomitant Therapy for details regarding prohibited and restricted therapy during the study.

2. Agree to follow all requirements that must be met during the study as noted in the Inclusion and Exclusion Criteria.

3. Concurrent administration of medications/use of licensed devices is allowed as supportive therapy per local standard of care, as long as the medication/licensed device will not affect the subject's participation in the study and is in accordance with allowed concomitant therapy. Parent(s)/caregiver(s) must agree to refrain from administering any new prescription or nonprescription medications other than study drug or those allowed as concomitant medications. If a disallowed medication is required it must be first discussed with investigational staff. Breastfeeding mothers also need to comply with the guidelines regarding concomitant medication.

5. TREATMENT ALLOCATION AND BLINDING

Treatment Allocation

Procedures for Randomization and Stratification

Central randomization will be implemented in this study. Subjects will be randomly assigned based on a computer-generated randomization schedule prepared before the start of the study by or under the supervision of the sponsor. The randomization will be balanced by using randomly permuted blocks and will be stratified by duration of RSV symptoms from onset till time of randomization (≤3 days versus >3 to ≤5 days) and by presence or absence of comorbidity. The interactive web response system (IWRS) will assign a unique treatment code, which will dictate the treatment assignment and matching study drug kit for the subject. The requestor must use his or her own user identification and personal identification number when contacting the IWRS, and will then give the relevant subject details to uniquely identify the subject.

Blinding

The investigator will not be provided with randomization codes. The codes will be maintained within the IWRS, which has the functionality to allow the investigator to break the blind for an individual subject.

Data that may potentially unblind the treatment assignment (ie, study drug serum concentrations, study drug preparation/accountability data, treatment allocation, biomarker or other specific laboratory data) will be handled with special care to ensure that the integrity of the blind is maintained and the potential for bias is minimized. This can include making special provisions, such as segregating the data in question from view by the investigators, clinical team, or others as appropriate until the time of database lock and unblinding.

Under normal circumstances, the blind should not be broken until the unblinded IDMC review takes place (see Section 11.10, Data Monitoring Committee). The blind will be broken for the interim analyses (see Section 11.9, Interim Analysis) or when all subjects have completed the study and the database is finalized. Otherwise, the blind should be broken only if specific emergency treatment/course of action would be dictated by knowing the treatment status of the subject. In such cases, the investigator may in an emergency determine the identity of the treatment by contacting the IWRS. It is strongly recommended that the investigator contact the sponsor or its designee if possible to discuss the particular situation, before breaking the blind. Telephone contact with the sponsor or its designee will be available 24 hours per day, 7 days per week. In the event the blind is broken, the sponsor must be informed as soon as possible. The date, time, and reason for the unblinding must be documented by the IWRS, in the appropriate section of the electronic case report form (eCRF) and in the source document. The documentation received from the IWRS indicating the code break must be retained with the subject's source documents in a secure manner.

Subjects who have had their treatment assignment unblinded should continue to return for scheduled evaluations. Discontinuation of study treatment should be done only for the reasons

stated in Section 10.2, Discontinuation of Study Treatment/Withdrawal From the Study; unblinding of study treatment should not necessarily lead to study drug discontinuation.

In general, randomization codes will be disclosed fully only if the study is completed and the clinical database is closed. However, if an interim analysis is specified, the randomization codes and, if required, the translation of randomization codes into treatment and control groups will be disclosed to those authorized and only for those subjects included in the interim analysis.

In the event that the subject continues into the long-term follow-up study, then the subject and investigator will remain blinded until the follow-up study is completed.

6. DOSAGE AND ADMINISTRATION

The study drug will be provided as a powder for suspension for oral administration. Study drug administration should start as soon as possible, but no later than 4 hours after randomization in order to maximize the opportunity for the compound to inhibit viral replication and potentially improve outcomes.

Subjects will be dosed with a single LD followed by 9 MDs twice daily (minimum 8 hours and maximum 16 hours apart with no more than 2 doses per calendar day) during Day 1 to Day 5/6 (depending on the timing of the LD).

Administration of each dose should occur at approximately the same time each day and after the once-daily collection of the mid-turbinate nasal swab. Lumicitabine can be administered without regard to food. The date and time of dose administration, the date and time of food intake (limited to the food ingested within 30 minutes before or after study drug administration), and the type of food will be recorded in the eCRF for hospitalized subjects.

Lumicitabine and placebo will be supplied as a powder in amber glass bottles containing 1,891 mg lumicitabine or placebo with a child-resistant cap. The powder should be suspended with solvent prior to oral administration, providing a 60 mg/mL suspension, under the supervision of the investigator or a qualified member of the study-site personnel, or by a hospital/clinic pharmacist. Instructions for preparation of weight-based study formulations are provided in the pharmacy manual.

In case a dose is missed, the dose may be given up to 6 hours after the scheduled time. If more time has elapsed the dose should be skipped and the next dose should be given at the next scheduled timepoint per the initial dose schedule.

In case the subject is discharged prior to the completion of dosing, study drug administration will continued at home by the parent(s)/caregiver(s) until all doses are administered (Day 5/6). The investigator/study-site personnel will instruct parent(s)/caregiver(s) on how to store study drug for at-home use as indicated for this protocol (see pharmacy manual).

An overdose in this study is defined as a calculated dose exceeding the planned dose.

7. TREATMENT COMPLIANCE

Study drug will be administered orally or via nasogastric tube (if applicable) by designated study-site personnel at the study-site during hospitalization. The investigator/study-site personnel will maintain a log of all study drug administered. Drug supplies for each subject will be inventoried and accounted for.

In the event of the subject being discharged (after Day 2 procedures) from hospital during the dosing period, the parent(s)/caregiver(s) will administer study drug at home and will be required to document the dose of study drug in a study medication log on the electronic device provided during screening.

Parent(s)/caregiver(s) will receive instructions on compliance with study drug administration on the day of hospital discharge. During the course of the study, the investigator or designated study-site personnel will be responsible for providing additional instruction to re-educate any parent(s)/caregiver(s) who is not able to ensure compliance with the study drug.

In case of vomiting or regurgitation, the subject should not be redosed. If the subject vomits, regurgitates, or does not completely swallow the study drug, it should be recorded in the eCRF, source documents (during hospitalization) and in the electronic parent(s)/caregiver(s) eCOA device (post discharge).

8. PRESTUDY AND CONCOMITANT THERAPY

Prestudy therapies administered up to 7 days before first dose of study drug must be recorded at screening.

All subjects will receive supportive care according to local institution standards and applicable guidelines. While treatment guidelines and standards vary based on local practice and should be considered in the management of subjects, within the parameters of this study, it is recommended that supplemental oxygen can be administered or withdrawn, as appropriate, to maintain an SpO_2 ≥93% as long as it is medically indicated (for subjects whose baseline SpO2 is ≥93% when clinically stable).

Concomitant therapies must be recorded throughout the study beginning with start of the first dose of study drug to the Day 28 visit (±2 days). During hospitalization, concomitant medication will be recorded by the site staff in the eCRF. Upon discharge, parent(s)/caregiver(s) will be required to document use of concomitant medication in the electronic device in a medication log, which will serve as basis for recording in the eCRF by the site staff. Concomitant therapies should also be recorded beyond Day 28 in the subject's medical notes and reported by SAE forms (if applicable).

All therapies (prescription or over-the-counter medications, including vaccines, vitamins, herbal supplements, home oxygen therapy and non-pharmacologic therapies such as electrical stimulation, acupuncture, special diets, exercise regimens) different from the study drug must be recorded in the eCRF. Recorded information will include a description of the type of the drug/therapy, treatment phase, dose regimen, route of administration, and its indication.

Modification of an effective preexisting chronic therapy should not be made for the explicit purpose of entering a subject into the study; however, if a subject has received acute doses of a prohibited drug, switching to an alternative drug chosen at the discretion of the investigator should be allowed.

Concomitant medications are permitted as indicated for the management of study subjects with the exception of the medications noted here. The following medications are not permitted during the study and for the time period prior to randomization as noted:

- Moderate/strong inhibitors of OAT3: diclofenac, diflunisal, gemfibrozil, mycophenolic acid, piperacillin, probenecid, and teriflunomide (all within 24 hours prior to randomization).
- Systemic medications (either chronically [more than 14 days] or within 21 days prior to randomization), which are known to modulate the host's immune response or increase viral shedding, such as immunomodulatory therapies, except for systemic corticosteroids as noted below.
- Systemic corticosteroids are acceptable if they are taken acutely (14 days or less prior to randomization).
- Prescription medications intended to prevent or treat the RSV infection itself (eg, ribavirin, IV immunoglobulin, palivizumab), an investigational drug, an investigational vaccine, or use of an invasive investigational medical device within 30 days or 5 half-lives (whichever is longer) before the planned first dose of study drug. Prescription medications intended to treat the symptoms/sequelae of the RSV infection are permitted.
- Measles, mumps, and rubella (single or combination) vaccine within 1 week before screening, according to parent report.

The sponsor must be notified in advance (or as soon as possible thereafter) of any instances in which prohibited therapies are administered.

9. STUDY EVALUATIONS

9.1. Study Procedures

9.1.1. Overview

The Time and Events Schedules summarize the frequency and timing of efficacy, PK, biomarker, medical resource utilization, health economic, and safety measurements applicable to this study. There are 2 Time and Events Schedules: 1 schedule for subjects who are hospitalized (up to their hospital discharge); 1 schedule for subjects after they are discharged from the hospital. Repeat or unscheduled samples may be taken for safety reasons or for technical issues with the samples.

The eCOA device will be provided to the parent(s)/caregiver(s) at screening to record the parent(s)/caregiver(s) ratings of the severity of the subject's symptoms (PRESORS) (see Attachment 5). The parent(s)/caregiver(s) will complete the eCOA twice daily (in the morning and in the evening); after Day 14, eCOA will be completed once daily in the evening (at approximately the same time each day) until the end of the study for subjects who have been discharged from the hospital. Whenever possible, the same parent(s)/caregiver(s) should

complete the eCOA throughout the study. The first parent/caregiver assessment on Day 1 needs to be recorded predose, before the LD. If the screening assessment was performed less than 8 hours prior to the LD, the predose assessment does not need to be performed. The first postdose parent/caregiver assessment must be performed between 6 and 12 hours after the LD (on Day 1 or Day 2, depending on the time of LD).

The investigator/study-site personnel will provide sufficient information to enable the parent(s)/caregiver(s) to complete the parent(s)/caregiver(s) eCOA and will ensure that the eCOA is completed correctly and on schedule to avoid missing or incorrect data.

Prior to completing the screening assessment, the parent(s)/caregiver(s) must complete a training module (included as part of the eCOA device) on how to enter responses to questions in the eCOA device. Study-site personnel will review the completion of the parent(s)/caregiver(s) eCOA on a daily basis.

The clinician eCOA (Attachment 5) will be completed by the investigator twice daily for the entire duration of hospitalization and once daily at the Day 7, Day 10, Day 14, and Day 28 visits during outpatient follow-up.

If multiple assessments are scheduled for the same timepoint, it is recommended that procedures be performed in the following sequence: noninvasive procedures should be collected first before invasive procedures (eCOA [parent/caregiver and clinician eCOA can be completed at the same time]; ECG first, then vital signs/SpO_2, then mid-turbinate nasal swab, with blood draw last). However, mid-turbinate nasal swabs for RSV viral load and blood collections for PK assessments should be kept as close to the specified time as possible. Other measurements may be done earlier than specified timepoints if needed. Actual dates and times of assessments will be recorded in the source documentation and eCRF. Electronic COA devices will automatically record the date and time of the eCOA assessments.

If treatment duration is extended up to 10 days, subjects will have an additional clinic visit 1 day (+2 days) after their last dose.

Medical resource utilization and health economics data will be collected. Refer to Section 9.6, Medical Resource Utilization and Health Economics, for details.

One additional PK assessment will be performed as indicated in the Time and Events Schedules for the first 9 subjects treated with the 60 mg/kg LD/40 mg/kg MD lumicitabine regimen if the Option A design (Section 3.1.1) is chosen and for the first 9 subjects in each cohort treated with the highest active lumicitabine regimen available in their respective cohort if the Option B design (Section 3.1.2) is chosen.

The results of clinical/safety evaluations that are performed as part of routine clinical care while hospitalized will be collected in the eCRF for viral, safety, and/or clinical evaluations.

9.1.2. Screening Phase

The procedures specified in the Time and Events Schedules will only be performed after the parent(s)/caregiver(s) written informed consent has been obtained.

Screening should be completed as soon as possible and the subject will be randomized within 5 days of RSV symptom onset to ensure the eligibility of the subject. Special attention should be paid to determine the duration of RSV symptoms from onset till time of randomization as accurately as possible. Procedures performed as part of standard of care within approximately 72 hours prior to randomization may be used in determining study eligibility or determining baseline values. The results of the safety laboratory tests performed at the local laboratory must be in accordance with Section 4, Subject Population. The investigator may consider the subject eligible if the previously abnormal laboratory test result is within normal range on a repeat testing in the local laboratory. Repeat testing is only allowed once.

During screening, 1 mid-turbinate nasal swab (if non-intubated) or 1 endotracheal sample (if intubated) will be collected and aliquoted for:

- RSV diagnosis using a PCR-based assay (at the local laboratory).
- RSV diagnosis confirmation using a PCR-based assay (at the central laboratory).
- Examination of viral and bacterial coinfection using multiplex PCR (at the central laboratory).
- Leftover mid-turbinate nasal swab samples will be stored and may be used for biomarker research if warranted.

9.1.3. Double-blind Treatment Phase

Day 1 (Day of Randomization and Loading Dose)

Eligible subjects will be randomized on Day 1. Study drug administration should start as soon as possible, but no later than 4 hours after randomization. Subjects will be dosed with a single oral LD on Day 1. Depending on the time of screening/enrollment, subjects will receive the first MD of study drug on Day 1. Subjects will receive no more than 2 doses per calendar day.

Assessments to be performed on Day 1 are specified in the Time and Events Schedule. The mid-turbinate nasal swab (if non-intubated) or the mid-turbinate nasal swab and endotracheal sample (if intubated) should be taken as close as possible to and before the LD on Day 1.

Day 2 to Day 5/6 (End of Treatment)

Study drug administration continues until 10 doses have been taken unless treatment duration is extended. Subjects will receive 9 MDs without regard to food intake twice daily (at least 8 hours apart and maximum 16 hours apart, with no more than 2 doses per calendar day) through Day 5/6 (depending on the timing of the LD). The date and time of dose administration, the date and time of food intake (limited to the food ingested within 30 minutes before or after study drug administration), and the type of food will be recorded in the eCRF for hospitalized subjects. The subject may be discharged after Day 2 following completion of study procedures. Subjects may

also remain hospitalized if warranted by the subject's clinical status. In case the subject is discharged prior to the completion of treatment, study drug will continue to be administered at home by the parent(s)/caregiver(s) until all doses are administered. Instructions regarding study drug administration will be given to the parent(s)/caregiver(s) by the investigator or a qualified member of the study-site personnel.

Assessments will be performed as detailed in the Time and Events Schedules. The parent(s)/caregiver(s) eCOA and the mid-turbinate nasal swabs (if non-intubated) (from the same nostril throughout the study) or endotracheal samples (if intubated) are to be taken once daily prior to study drug administration at approximately the same time each day. In case of nostril bleeding or irritation during the collection of a mid-turbinate nasal swab, all subsequent nasal swabs will be performed on the contralateral nostril. Should the study drug duration be increased to up to 10 days (following review of the data by the IDMC), samples will be taken every day up to and including 1 day (+2 days) following the last dose of study drug. Samples will be collected through Day 6 whether the subject is in hospital or has been discharged. In case mid-turbinate nasal swabs are collected at home, the subject's parent(s)/caregiver(s) should collect nasal swab specimens after being trained by the investigator/study-site personnel. Parent(s)/Caregiver(s) of subjects will be required to document the actual dates and times of collection of the nasal swab at home on the electronic device used for eCOA. Nasal swabs collected at home should be stored immediately between 2°C and 8°C (in the refrigerator) and brought to the site at the next visit.

Hospitalization duration will not be extended solely for study purposes. Telephone calls to parent(s)/caregiver(s) to facilitate compliance with study procedures between outpatient study visits are encouraged.

9.1.4. Posttreatment Phase (Follow-up)

Days 7 to 28

Subjects will be evaluated for a total of 28 days after randomization. If they are not hospitalized they will be required to return to the hospital for follow-up assessments as an outpatient on Day 7, Day 10, Day 14, and Day 28 as indicated in the Time and Events Schedules. On Day 28 all subjects will complete the study either as an inpatient or outpatient.

Should the study drug duration be increased to up to 10 days (following review of the data by the IDMC, refer to Section 11.10, Independent Data Monitoring Committee), additional study drug may be provided if needed. If a subject is discontinued from study drug, a follow-up visit will be scheduled 2 days (+2 days), 5 days (+2 days), 9 days (+2 days), and 23 days (±2 days) after the last dose of study drug was administered. Assessments should be performed as indicated in the Time and Events Schedules, respectively for the Day 7, Day 10, Day 14, and Day 28 visits.

9.2. Efficacy Evaluations

Viral Load Determination

As an evaluation of antiviral activity of lumicitabine, RSV viral load will be measured in mid-turbinate nasal swabs (obtained from non-intubated subjects) or in mid-turbinate nasal

swabs and endotracheal samples (obtained from intubated subjects or via suction through tracheostomy or other sampling methods) using qRT-PCR performed at the central laboratory. Samples for the determination of RSV viral load will be taken as close as possible and before the LD on Day 1 and once daily at approximately the same time each day prior to study drug administration between Day 2 and Day 6 (whether hospitalized or not), and at the Day 7, Day 10, Day 14, and Day 28 visits. For each subject, the different mid-turbinate nasal swabs should be collected from the same nostril. However, in case of nostril bleeding or irritation during the collection of a mid-turbinate nasal swab, all subsequent nasal swabs will be performed on the contralateral nostril.

Quantitative culture of RSV using plaque assay may be performed centrally at timepoints selected by the virologist.

Should the study drug duration be increased to up to 10 days (following review of the data by the IDMC), 1 mid-turbinate nasal swab (if non-intubated) or 1 mid-turbinate nasal swab and 1 endotracheal sample (if intubated) will be taken every day, at approximately the same time, up to and including 1 day (+2 days) following the last dose of study drug.

Viral L-gene Sequencing for Viral Resistance

Viral sequencing analysis will be performed by sequencing the polymerase L-gene and other regions of the RSV genome (if warranted) in samples taken before treatment (at baseline), during treatment, and posttreatment, to identify preexisting sequence polymorphisms and to characterize emerging RSV variants.

The L-gene will be sequenced pretreatment in all subjects and postbaseline in subjects not achieving viral suppression. The sequencing of postbaseline samples may be triggered at the discretion of the sponsor’s virologist, based on the changes in RSV viral load observed in each individual subject and the limits of the sequencing assay and taking into account the limits of the sequencing assay. The impact of newly identified mutations on in vitro antiviral activity of lumicitabine will be assessed.

Other regions of the RSV genome may also be sequenced at the sponsor’s discretion. The impact of the baseline viral genotype and subtype on the antiviral activity will be explored.

The sequencing methods of the L-gene and other regions of the RSV genome will be described in a separate laboratory manual.

Clinical Course of RSV Infection

The study will include, but may not be limited to, the following evaluations of the clinical course of RSV infection:

- Requirement for and duration of oxygen supplementation/noninvasive mechanical ventilation support (eg, nasal cannula, face mask, continuous positive airway pressure) and/or invasive mechanical ventilation support (eg, endotracheal-mechanical ventilation or mechanical ventilation via tracheostomy).

- Body weight.
- Respiratory rate, heart rate, systolic blood pressure (SBP), and diastolic blood pressure (DBP), SpO_2, and body temperature. An overview of the guidelines for vital sign (and SpO_2) measurement is provided in Attachment 6. The method should be noted in the eCRF.
- Level of hospital care (eg, ICU, translational care unit, ward floor).
- Duration of hospitalization.
- Requirement for hydration and feeding by IV catheter/nasogastric tube.

Clinician-reported Evaluation of Clinical Outcomes

- Clinician evaluation of the duration and severity of signs and symptoms of RSV infection (clinician eCOA; PRESORS).

Parent(s)/Caregiver(s)-reported Evaluation of Clinical Outcomes

- Parent(s)/caregiver(s) evaluation of the severity of signs and symptoms of RSV infection and the impact on the subject's functioning (parent[s]/caregiver[s] eCOA; PRESORS) which will be performed if possible by the same parent/caregiver at each timepoint.

9.3. Pharmacokinetics

Whole blood samples will be used to determine the concentrations of JNJ-63549109 and JNJ-64167896. Concentrations of JNJ-63549109 will be used to estimate the PK parameters of JNJ-63549109 using a popPK model. Samples collected for PK may additionally be used to evaluate safety or efficacy aspects that address concerns arising during or after the study period. Genetic analyses will not be performed on these samples. Subject confidentiality will be maintained.

9.3.1. Evaluations

Whole blood samples for PK analysis will be collected at the scheduled timepoints specified in the Time and Events Schedules, via venipuncture, heel or finger prick, or a venous or arterial line. The exact time of sample collection will be noted on the source document and the data collection tool.

If blood samples are obtained at other times or by other methods as a part of routine clinical care, every effort should be made to "scavenge" leftover blood samples for PK analysis. The date and time of the scavenged blood specimen collection should be documented. All scavenged samples should be stored at -80ºC (preferred) or -20ºC as soon as possible.

Additional PK samples may also be collected on an ad hoc basis in the event that a safety event arises for which an understanding of drug exposure is clinically important, at the discretion of the investigator or at the request of the sponsor. Additional information about the collection, handling, and shipment of biologic samples can be found in the laboratory manual.

9.3.2. Analytical Procedures

Whole blood samples will be analyzed to determine concentrations of JNJ-63549109 and JNJ-64167896 using a validated, specific, and sensitive liquid chromatography/mass spectrometry, method by or under the supervision of the sponsor.

To maintain the study blind during the study, results of the individual PK analyses will not be shared with the investigator or sponsor representatives directly involved in managing the study.

Results from all scavenged samples used for PK analysis will be reported and included in the PK calculations.

9.3.3. Pharmacokinetic Parameters

Based on the individual concentration-time data, using the actual dose taken and the actual intake and sampling times, PK parameters and exposure information will be derived using popPK modeling. The estimated model parameters and individual post hoc exposure parameters (AUC, predicted concentration at 12 hours postdose [C_{12h}], and C_{max}) will be provided for each subject as appropriate. Baseline covariates (eg, body weight, age, sex, creatinine clearance, and race) may be included in the model, if relevant.

Exposure estimates (AUC, C_{max}, and C_{12h}) will be obtained by empirical Bayesian feedback using the popPK model for JNJ-63549109 to assess the appropriateness of the popPK model and the selected doses, throughout the conduct of the study. Concentration data versus time after dose of each individual will be plotted. A previously developed popPK model based on adult and pediatric data from several studies will be used to overlay with the raw data. If the data fall outside the prediction interval of the model, the model will be updated. Furthermore, potential covariates (not present in the previously developed model) will be investigated graphically and can be included into the popPK model based on a significant decrease in objective function using the likelihood ratio test for nested models.

9.4. Pharmacokinetic/Pharmacodynamic Evaluations

The relationship between the PK of JNJ‑63549109 and PD (antiviral activity, clinical outcomes, and selected safety parameters) after single and repeated oral dosing of lumicitabine will be evaluated.

9.5. Biomarkers

Leftover mid-turbinate nasal swabs and blood samples collected for PK testing may be used for exploratory biomarker analyses (eg, proteins including cytokines), on the premise that these markers may play a role in the treatment response, safety of lumicitabine, or the status and change of the RSV-related disease. Analyses of biomarkers may be conducted at the sponsor’s discretion and reported separately from this study.

No human DNA analyses will take place on these samples.

9.6. Medical Resource Utilization and Health Economics

Medical resource utilization and health economics data, associated with medical encounters related to RSV and/or respiratory reasons, will be collected in the eCRF by the investigator and study-site personnel for all subjects at the timepoints specified in the Time and Events Schedules.

Protocol-mandated procedures, tests, and encounters are excluded. The data collected may be used to conduct exploratory economic analyses and will include:

- Number and duration of medical care encounters, including surgeries and other selected procedures (inpatient and outpatient).
- Duration of hospitalization (total days length of stay, including duration by wards [eg, ICU]).
- Outpatient medical encounters and treatments (including physician or ER visits, tests and procedures, and medications).
- The number of subjects (proportion) who started antibiotic use after the first dose of the study drug up to the Day 28 follow-up visit.
- Requirement for, and duration of, hospital readmission for respiratory reasons from discharge up to the Day 28 follow-up visit.

9.7. Safety Evaluations

Safety and tolerability will be evaluated throughout the study from signing of the ICF onwards until the last study-related activity (end of study/early withdrawal).

An IDMC will be established to monitor the safety of subjects and will review data in an unblinded manner on a regular basis to ensure the continuing safety of the subjects enrolled in this study. The IDMC will review the data and make recommendations to the Sponsor Committee, which will be responsible for identifying appropriate actions based on the recommendations of the IDMC. Details regarding the IDMC are provided in Section 11.10, Independent Data Monitoring Committee.

Any clinically relevant changes occurring during the study must be recorded on the AE section of the eCRF.

Any clinically significant abnormalities persisting at the end of the study/early withdrawal will be followed by the investigator until resolution or until a clinically stable endpoint is reached.

Safety assessments will be based on medical review of AE reports and the results of vital signs/SpO_2 measurements, ECGs, physical examinations, clinical laboratory tests, and other safety evaluations at specified timepoints as described in the Time and Events Schedules. The results of unscheduled clinical/safety evaluations performed as part of routine clinical care while the subject is hospitalized will be collected in the eCRF for safety and/or clinical evaluation.

In the event that at least 2 invasive procedures such as a blood draw, mid-turbinate nasal swab, vital signs assessments, and/or ECG are required at the same time, the order of completion should be ECG first, then vital signs/SpO_2, then mid-turbinate nasal swab, then blood draw, with the blood draw for PK being obtained as close to the scheduled time point as possible.

The study will include the following evaluations of safety and tolerability according to the timepoints provided in the Time and Events Schedules.

Adverse Events

Adverse events will be reported by the parent(s)/caregiver(s) or the subject's legally acceptable representative for the duration of the study. Adverse events will be followed by the investigator as specified in Section 12, Adverse Event Reporting.

Clinical Laboratory Tests

Blood samples for serum chemistry and hematology will be collected and analyzed by a local laboratory. The investigator must review the laboratory results, document this review, and record any clinically relevant changes occurring during the study in the AE section of the eCRF. The laboratory reports must be filed with the source documents.

Blood samples may be obtained by venipuncture or by heel or finger prick. Alternatively, they may be obtained through a venous or arterial line. Blood samples obtained as a part of routine clinical care within the protocol visit window may be used to meet planned study evaluations. Additionally, any blood samples left over from sampling done for routine clinical care within the protocol visit window may be "scavenged" to be used in the study-specific safety evaluations.

The following tests will be performed:

- Hematology Panel

-hemoglobin	Percentage and absolute counts of:
-hematocrit	-basophils
-mean corpuscular hemoglobin	-eosinophils
-mean corpuscular hemoglobin concentration	-lymphocytes
-platelet count	-monocytes
-red cell distribution width	-neutrophils
-reticulocytes	
-red blood cell (RBC) count	
-white blood cell (WBC) count	

Note: A WBC evaluation may include any abnormal cells, which will then be reported by the laboratory. A RBC evaluation may include abnormalities in the RBC count, RBC parameters, or RBC morphology, which will then be reported by the laboratory. In addition, any other abnormal cells in a blood smear will also be reported.

- Serum Chemistry Panel

-alkaline phosphatase	-creatinine
-ALT	-creatine kinase
-aspartate aminotransferase	-glucose
-bicarbonate (optional)	-potassium
-blood urea nitrogen	-sodium
-chloride	-total (direct and indirect) bilirubin

- Urinalysis

Dipstick	Sediment (if dipstick result is abnormal)
-specific gravity	-RBCs
-pH	-WBCs
-glucose	-epithelial cells
-protein	-crystals
-blood	-casts
-ketones	-bacteria
-bilirubin	
-urobilinogen	
-nitrite	
-leukocyte esterase	

If dipstick result is abnormal, microscopy will be used to measure sediment. In the microscopic examination, observations other than the presence of WBCs, RBCs, and casts may also be reported by the laboratory and need to be captured in the eCRF.

Sites should attempt to collect all safety labs as defined in this protocol. In some instances, individual laboratory assessments are not routinely run at a local site. If it is not practical to

obtain the results for a particular assessment due to local considerations, the sponsor's study responsible physician or delegate may on a case by case basis permit individual sites to not collect that assessment.

Electrocardiogram

Electrocardiograms will be collected at the timepoints specified in the Time of Events Schedule and when clinically indicated by the investigator.

Electrocardiograms should be performed using procedures commensurate with the subject's age. A 12-lead ECG is strongly preferred but in cases where a 12-lead ECG is not possible or feasible, then a 6-lead ECG may be performed.

During the collection of ECGs, subjects should be calm, resting quietly, or sleeping if possible. If blood sampling or vital sign measurement is scheduled for the same timepoint as ECG recording, the procedures should be performed in the following order: ECG(s), vital signs/SpO_2, mid-turbinate nasal swabs, and then blood draw.

Electrocardiograms may be repeated at the investigator's discretion to account for erroneous readings.

Clinically relevant abnormalities (as defined in Attachment 2) occurring during the study should be recorded by the investigator in the AE section of the eCRF.

Vital Signs/SpO_2

Body temperature, heart rate, respiratory rate, SBP, DBP, and SpO_2 are to be collected at the timepoints specified in the Time and Events Schedules.

Blood pressure and heart rate measurements will be assessed using procedures commensurate with the subject's age, preferably with a completely automated device. Manual techniques will be used only if an automated device is not available. Blood pressure and heart rate measurements should be preceded by at least 5 minutes of rest in a quiet setting without distractions and be taken while the subject is calm (resting or sleeping and not crying). They should not be performed immediately following the administration of beta-mimetics or any supportive drug, which could affect the measurement.

Clinically relevant abnormalities (as defined in Attachment 2) occurring during the study should be recorded by the investigator in the AE section of the eCRF.

Physical Examination

A complete physical examination (including all body systems) or a directed physical examination (including respiratory system, nose, ear, throat, facial and neck lymph nodes, and skin examination) will be performed at the timepoints specified in the Time and Events Schedules. Length, head circumference, and body weight will be measured at the timepoints specified in the Time and Events Schedules.

A skin examination includes an examination of the mucous membranes, but does not include a vaginal or rectal examination. However, if the subject develops a cutaneous reaction/rash, vaginal and rectal examinations may be done if clinically relevant.

The investigator must review the physical examination results, document this review, and record any clinically relevant changes occurring during the study in the AE section of the eCRF.

9.8. Specific Toxicities

Subjects reporting skin changes or subjects with laboratory parameter changes should be followed until resolution of the toxicity and necessary standard management should be undertaken.

Skin Changes

In case there are skin changes, a correct diagnosis has to be made by the investigator or a dermatologist, preferably within 24 hours of detection. An assessment of severity grade should be made using the criteria specified in the draft Division of Microbiology and Infectious Diseases (DMID) pediatric toxicity tables (see Attachment 1).

Diaper dermatitis should be managed as per standard of care. Other rashes should be managed as indicated in Attachment 4.

Laboratory Parameter Changes

If one (or more) of the following changes occur, the subject should discontinue study drug and should be followed until resolution (return to baseline) or stabilization of change (to be agreed upon with the sponsor). The sponsor should be informed even if the change occurs outside of the treatment period (but before the end of the study). Refer to Section 10.2, Discontinuation of Study Treatment/Withdrawal from the Study.

- Liver enzymes: ALT ≥3x upper limit of normal (ULN) AND bilirubin ≥2×ULN (direct >35%) OR ALT ≥5×ULN.

9.9. Sample Collection and Handling

The actual dates and times of sample collection must be recorded in the eCRF.

For the PK samples, the time of the preceding meal, whether the suspension was completely ingested or any vomiting/regurgitation of the suspension occurred, will be collected in the eCRF.

Refer to the Time and Events Schedules for the timing and frequency of all sample collections. Information regarding the collection of the mid-turbinate nasal swabs during hospitalization or at home should be entered in the eCRF or eCOA, respectively.

Instructions for the collection, handling, storage, and shipment of samples are found in the laboratory manual that will be provided. Collection, handling, storage, and shipment of samples must be under the specified, and where applicable, controlled temperature conditions as indicated in the laboratory manual.

10. SUBJECT COMPLETION/DISCONTINUATION OF STUDY TREATMENT/ WITHDRAWAL FROM THE STUDY

10.1. Completion

A subject will be considered to have completed the treatment phase of the study if he or she has completed the treatment regimen and the Day 7 visit or the visit 1 day (+2 days) after the final dose of study drug if the study drug administration is increased to up to 10 days following IDMC review of the data. A subject will be considered to have completed the study if he or she has also completed assessments of the last follow-up visit.

10.2. Discontinuation of Study Treatment/Withdrawal From the Study

Discontinuation of Study Treatment

A subject will not be automatically withdrawn from the study if he or she has to discontinue treatment before the end of the treatment regimen.

A subject's study treatment must be discontinued if:

- The subject's parent(s)/caregiver(s) withdraws consent.
- The investigator believes that for safety reasons or tolerability reasons (eg, AE) it is in the best interest of the subject to discontinue study treatment.
- The subject experiences any of the laboratory abnormalities specified in Section 9.8, Specific Toxicities.

If a subject discontinues study treatment for any reason before the end of the treatment phase, the subjects' parent(s)/caregiver(s) will be encouraged to continue with end-of-treatment and follow-up assessments in accordance with the Time and Events Schedules.

Individual Subject Discontinuation Criteria

If one (or more) of the following changes occur, the subject should discontinue study drug and should be followed until resolution (return to baseline) or stabilization of change (to be agreed upon with the sponsor). The sponsor should be informed even if the change occurs outside of the treatment period (but before the end of the study).

- A treatment-emergent AE which is considered clinically significant (eg, meeting Grade 3 [severe] or 4 [potentially life threatening] criteria) and is attributed to study drug and/or which is thought to preclude further safe administration of study drug.
- An AE/SAE, drug reaction, or complication, whether related or not to study drug, which precludes continuation of treatment with study drug.
- Liver enzymes: ALT ≥3xULN AND bilirubin ≥2×ULN (direct >35%) OR ALT ≥5×ULN.
- Noncompliance with study drug dosing.
- Noncompliance with study procedures.
- Lost to follow-up.

- The principal investigator's opinion that it is not in the subject's best interest to continue study participation.
- Sponsor's decision to terminate the study.

Cohort Discontinuation Criteria (Option B)

Further enrollment within a cohort or enrollment to subsequent cohorts will be discontinued once it has been established that safety risk(s) such as the following occur:

- If any unacceptable toxicity (as determined by the Sponsor Medical Monitor) occurs.
- If the IDMC considers an event or multiple events to represent an unacceptable risk to the health and well-being of subjects at that or higher dose levels.

Withdrawal From the Study

Each parent/caregiver has the right to withdraw the subject at any time for whatever reason without affecting the right to treatment by the investigator. The investigator should make an attempt to contact the parent(s)/caregiver(s) of subjects who did not return for scheduled visits or follow-up. Although the subject's parent(s)/caregiver(s) are not obliged to give reason(s) for withdrawing early, the investigator should make a reasonable effort to ascertain the reason(s) while fully respecting the subject's rights.

A subject will be withdrawn from the study for any of the following reasons:

- Lost to follow-up.
- Withdrawal of consent.
- Death.
- The subject's parent(s)/caregiver(s) is poorly compliant with study procedures, study drug administration, visits, and assessments, after evaluation and discussion between the investigator and the sponsor.
- Decision by the sponsor to stop or cancel the study.
- Decision by the investigator to withdraw subjects.
- Decision by local regulatory authorities and Independent Ethics Committee (IEC)/Institutional Review Board (IRB) to stop or cancel the study.

If parent(s)/caregiver(s) withdraw the subject from the study before the end of the treatment phase, the subject's parent(s)/caregiver(s) will be encouraged to continue with end-of-treatment and follow-up assessments in accordance with the Time and Events Schedules.

If a subject is lost to follow-up, every reasonable effort must be made by the investigator/study-site personnel to contact the parent(s)/caregiver(s) and determine the reason for discontinuation/withdrawal. The measures taken to follow up must be documented.

When a subject is withdrawn by his/her parent(s)/caregiver(s) before completing the study, the reason for withdrawal is to be documented in the eCRF and in the source document. Study drug assigned to the withdrawn subject may not be assigned to another subject. Subjects who have withdrawn before all RSV mid-turbinate nasal swabs (for RSV viral load) or PK samples have been taken may be replaced. If a subject withdraws from the study before the end of the treatment phase, end-of-treatment and follow-up assessments should be obtained, unless the reason for withdrawal was withdrawal of consent.

10.3. Withdrawal From the Use of Research Samples

A subject who withdraws from the study will have the following options regarding exploratory biomarker research samples:

- The collected (scavenged) sample(s) will be retained and used in accordance with the subject's parent(s)/caregiver(s)' original informed consent for exploratory biomarker research.
- The parent(s)/caregiver(s) may withdraw consent for exploratory biomarker research, in which case the exploratory biomarker research samples will be destroyed and no further testing will take place. To initiate the sample destruction process, the investigator must notify the sponsor study-site contact of withdrawal of consent for the research samples and to request sample destruction. The sponsor study-site contact will, in turn, contact the biomarker representative to execute sample destruction. If requested, the investigator will receive written confirmation from the sponsor that the samples have been destroyed.

Withdrawal From the Use of Samples in Future Research

The parent(s)/caregiver(s) may withdraw consent for use of samples for research (refer to Section 16.2.5, Long-term Retention of Samples for Additional Future Research). In such a case, samples will be destroyed after they are no longer needed for the clinical study. Details of the sample retention for research are presented in the main ICF.

11. STATISTICAL METHODS

Statistical analysis will be done by the sponsor or under the authority of the sponsor. A general description of the statistical methods to be used to analyze the efficacy and safety data is outlined below. Specific details will be provided in the statistical analysis plan (SAP). The SAP for the IDMC will be separate from the SAP for the final analysis. In addition, a separate SAP for the interim analyses will be prepared.

All subjects who are randomized, have an RSV infection confirmed by central laboratory assessment, and receive at least 1 dose of study drug will constitute the intent-to-treat (ITT) infected population (ITT-i). Treatment assignment for this population will be defined by randomization assignment. The ITT-i population will be used for all efficacy/virologic analyses.

The per-protocol population will be defined as all subjects included in the ITT-i population without protocol deviations that affect efficacy assessment. The per-protocol population of ITT-i subjects with a postbaseline value will be established during the blinded data review and may be

used to perform sensitivity analysis on the primary endpoint. A blinded data review will be performed before each formal interim analysis.

All subjects enrolled into the study who receive at least 1 dose of study drug will be included in the safety population. Subjects in this population will be defined by the drug actually received, not by randomization assignment. Any subject who receives a single dose of lumicitabine will thus be included in the lumicitabine arm and all subjects that receive only placebo will be included in the placebo arm. The safety population will be used for all safety analyses.

11.1. Subject Information

All demographic characteristics and other initial subject characteristics will be tabulated and analyzed descriptively. Treatment adherence to lumicitabine will be derived from the collected information.

11.2. Sample Size Determination

The primary hypothesis is a positive dose-response relationship of active treatment on the average RSV viral load AUC over 7 days, meaning that either the average AUC on the pooled active treatments is lower than on placebo, or the average AUC on the high active dose is lower than the average AUC on placebo using multiple contrast testing. On high dose, the hypothesis of the lumicitabine antiviral activity assumes a viral load reduction on the AUC of the log viral load over 7 days of at least 30% when compared to placebo in subjects with onset of symptoms ≤3 days and of at least 20% in subjects with onset of symptoms >3 days. The antiviral activity on low dose is assumed to be 80% when compared to the antiviral activity on the high dose. The positive dose-response relationship assumes that dose regimens with higher exposure with respect to MD will have at least an equal or better effect on viral load. Therefore 2 contrasts will be tested at each of the interim analysis points and final analysis; a contrast with no difference between the 2 active regimens tested against placebo (high equal to low, better than placebo) and a contrast with a positive 'linear' dose-response relationship (high better than low, low better than placebo) with respect to active regimens (where the effect of the low dose is exactly in between that of placebo and the high dose regimen). With respect to multiple contrast testing, multiplicity (2 contrasts) will be controlled at the prespecified (interim) alpha level by calculating adjusted p-values from the simulated distribution of the maximum or maximum absolute value of a multivariate t random vector (ie, using the correlation between the contrasts to optimally control for alpha).[19] Based on the observed data of other in-house studies, the estimated SD is assumed to be approximately 34% of the AUC for subjects treated with placebo and the SD is equal in each of the treatment regimens. The overall (family wise) type 1 error rate of 2.5% (1-sided) will be adjusted for multiple testing due to formal interim analyses using a Pocock alpha spending function with 3 sequential tests (2 interim, 1 final).[15] Based on 10,000 simulations and using a general linear model with treatment regimen as fixed factors, and under the assumption that 40% of the recruited subjects have onset of symptoms ≤3 days, a sample size of 180 subjects randomized in a 1:1:1 ratio will offer approximately 97% power to detect a positive dose-response relationship using multiple contrast testing. With the second interim analysis planned after at least 120 subjects, there will be at least 80% power to detect a positive dose-response relationship at the time of this analysis.

These sample size calculations were done under the assumption that option A is selected. Under option B, using similar assumptions, the total power is somewhat lowered but still sufficient for the total sample size of 180 subjects.

11.3. Efficacy Analyses

Primary Endpoint

The primary endpoint in this study is RSV RNA $\log_{10}$ viral load (measured by qRT-PCR assay in the mid-turbinate nasal swab specimens) AUC immediately prior to first dose of study drug (baseline) over 7 days. Mean $\log_{10}$ viral load values over time will be analyzed using a restricted maximum likelihood-based repeated measures approach. Analyses will include the fixed, categorical effects of treatment, strata, visit, and treatment-by-visit interaction, as well as the continuous, fixed covariates of baseline $\log_{10}$ viral load and baseline $\log_{10}$ viral load by-visit interaction. An unstructured (co)variance structure will be used to model the within subject errors over time. The Kenward-Roger method will be used to approximate the degrees of freedom.[16] The difference in the AUCs for active versus placebo will be derived using appropriate contrasts deriving least square mean differences, including the 95% 2-sided confidence intervals. For inferential purposes, p-values will be compared with significance levels controlling for the (family-wise) type 1 error rate. Details will be provided in the SAP that will be finalized before the first formal interim analysis.

Secondary Endpoints

For differences between treatment groups on qRT-PCR the same methods as for the primary endpoint will be used, using appropriate contrasts to estimate treatment differences between the various treatment groups by treatment day using nominal 95% confidence intervals.

The time to virus not detected is defined as the time from initiation of study treatment until the time at which it is observed that the virus is undetectable in an assessment and after which time no virus positive assessment follows. Subjects will be (right-)censored at the last time observation of a detectable viral load. Actual sample time will be used. Data will be analyzed using Kaplan-Meier plots and using an accelerated failure time model to estimate difference between treatments, adjusting for strata and baseline viral load. Other baseline covariates may be added when statistically significant. In case the accelerated failure time model provides a poor model fit, appropriate sensitivity analyses will be performed as detailed in the SAP.

The time to clinical stability is defined as the time from initiation of study treatment until the time at which the following criteria are met: return to pre-RSV infection status (hereafter referred to as "normalization") of blood oxygen level (ie, no additional requirement of supplemental oxygen compared with pre-RSV infection status), normalization of oral feeding, normalization of respiratory rate, and normalization of heart rate. Normalization criteria will be specified in the SAP. Subjects for which no timepoint could be established (eg, withdrawal of informed consent before reaching the endpoint or no clinical stability at last observation) will be (right-)censored at the latest timepoint at which it could be established that clinical stability was not reached. The differences in time to clinical stability of active dose regimens versus placebo treatment will be estimated using a Cox proportional hazard model; as baseline covariates, stratification factors,

absence/presence of other viral or bacterial infections, and baseline log_{10} viral load will be added. Treatment effect versus placebo will be reported, including 95% confidence intervals. In case the proportional hazards assumption is not satisfied, appropriate sensitivity analyses will be performed as detailed in the SAP.

Additionally, statistical modeling will be performed on the secondary endpoint of length of hospital stay, defined as the time from treatment initiation to hospital discharge in hours. Subjects for whom no timepoint could be established (eg, withdrawal of informed consent before reaching the endpoint) will be (right-)censored at the latest timepoint at which it could be established that the endpoint was not reached. The differences in length of hospital stay of active dose regimens versus placebo treatment will be estimated using the accelerated failure time model using appropriate contrasts; as baseline covariates stratification factors, absence/presence of other viral or bacterial infections, and baseline log_{10} viral load will be added. Treatment effect versus placebo will be reported as acceleration factors including 95% confidence intervals. In case the model assumptions are not adequately met, appropriate sensitivity analyses will be performed as detailed in the SAP. For the secondary endpoint of length of hospital stay, defined as the time from study treatment initiation to readiness for discharge in hours, with readiness for discharge defined by the investigator, an analogous approach will be used. The length of hospital stay from admission to discharge and from admission to readiness for discharge (as defined by the investigator), measured in days, will be analyzed using an analogous approach. Analytical details will be provided in the SAP.

Analysis of the sequencing results of the L-gene or other genes (only if no mutations are seen in the L-gene) will be described in the SAP. Statistical analysis of the viral load, that may be generated with the plaque assay, will be described in the SAP.

Parent/Caregiver-reported Evaluation of Clinical Outcomes

Parent/caregiver-reported eCOA (PRESORS) will be descriptively summarized by treatment group and compared across treatment groups.

Clinician-reported Evaluation of Clinical Outcomes

Clinician-reported eCOA (PRESORS) will be descriptively summarized by treatment group and compared across treatment groups.

11.4. Pharmacokinetic Analyses

Population PK analysis of concentration-time data of JNJ-63549109 will be performed using nonlinear mixed-effects modeling. Data may be combined with those of other selected studies to support a relevant structural model. Available baseline subject characteristics (eg, demographics, laboratory variables, and race) will be tested as potential covariates affecting PK parameters. Details will be given in a popPK analysis plan and the key findings of the popPK analysis will be presented in the final clinical study report. The popPK report will be provided separately.

A snapshot date for PK samples to be analyzed for the planned interim analysis will be defined. Samples collected before this date will be analyzed for JNJ-63549109 and JNJ-64167896 and

included in the popPK analysis (for JNJ-63549109 only). Samples collected after the snapshot date will be analyzed at a later date, and may be included in a popPK reanalysis when they become available after interim analysis database lock.

Subjects will be excluded from the PK analysis if their data do not allow for accurate assessment of the PK (eg, incomplete administration of the study drug; missing information of dose and sampling times; concentration data not sufficient for PK parameter calculation).

All concentrations below the lowest quantifiable concentration or missing data will be labeled as such in the concentration database. All subjects and samples excluded from the analysis will be clearly documented in the popPK report. The estimated model parameters and individual post hoc exposure parameters (AUC, C_{max}, and C_{12h}) will be provided for each subject as appropriate. Baseline covariates (eg, body weight, age, sex, creatinine clearance, and race) may be included in the model, if relevant.

For each treatment regimen, descriptive statistics, including arithmetic mean, SD, coefficient of variation, median, minimum, and maximum will be calculated for all individual derived PK parameters including exposure information of JNJ-63549109.

11.5. Biomarker Analyses

Statistical approaches to explore correlations between clinical outcome and biomarkers vary and depend on the different data types of the applied technology platforms, as well as on the extent of observed differences among study subjects. Analyses may be conducted at the sponsor's discretion and reported separately from this study.

11.6. Pharmacokinetic/Pharmacodynamic Analyses

Selected efficacy and safety parameters will be subject to PK/PD analysis. JNJ-63549109 exposures will be evaluated as an independent variable, with selected efficacy and selected safety parameters considered as dependent variables. Various approaches, including graphical analysis, linear, nonlinear, and logistic regression methods may be utilized.

The key findings of the PK/PD analyses will be presented in the final clinical study report.

11.7. Medical Resource Utilization and Health Economics Analyses

Medical resource utilization and health economics will be descriptively summarized by treatment regimen and compared across treatment regimens.

11.8. Safety Analyses

Safety data will be presented descriptively. No statistical testing of safety data is planned. For safety, baseline is defined as the last assessment prior to the first intake of study drug.

Adverse Events

The verbatim terms used in the eCRF by investigators to identify AEs will be coded using the Medical Dictionary for Regulatory Activities. Treatment-emergent AEs are AEs with onset during the treatment phase or that are a consequence of a preexisting condition that has worsened since baseline. All reported AEs will be included in the analysis. For each AE, the percentage of

subjects who experience at least 1 occurrence of the given event will be summarized by treatment regimen. In addition, comparisons between treatment regimens will be provided if appropriate.

Summaries, listings, datasets, or subject narratives may be provided, as appropriate, for those subjects who die, who discontinue treatment due to an AE, or who experience a severe or an SAE.

Clinical Laboratory Tests

Laboratory data will be summarized by type of laboratory test. Reference ranges and markedly abnormal results (specified in the SAP) will be used in the summary of laboratory data. Descriptive statistics will be calculated for each laboratory analyte at baseline and for observed values and changes from baseline at each scheduled timepoint. Changes from baseline results will be presented in pre- versus posttreatment cross-tabulations (with classes for below, within, and above normal ranges). Frequency tabulations of the abnormalities will be made. A listing of subjects with any laboratory results outside the reference ranges will be provided. A listing of subjects with any markedly abnormal laboratory results will also be provided.

The laboratory abnormalities will be determined according to the criteria specified in the draft DMID pediatric toxicity tables (see Attachment 1) and in accordance with the normal ranges of the clinical laboratory if no gradings are available.

Electrocardiogram

The effects on cardiovascular variables will be evaluated by means of descriptive statistics and frequency tabulations. These tables will include observed values and changes from baseline at each scheduled timepoint (the predose ECG will be used as baseline). Frequency tabulations of the abnormalities will be made.

The ECG variables that will be analyzed are heart rate, PR interval, QRS interval, QT interval, and QTcF.[12,14,19] The percentage of subjects with abnormalities as defined in Attachment 2 will be tabulated.

Vital Signs/SpO_2

Descriptive statistics of body temperature, heart rate, respiratory rate, SBP, DBP, SpO_2, and changes from baseline will be summarized at each scheduled timepoint. For SpO_2 an overview will be presented for the number of subjects (proportion) requiring supplemental oxygen. The percentage of subjects with values beyond clinically important limits, as judged by the investigator, will be summarized.

Physical Examination

Physical examination findings will be summarized at each scheduled timepoint. Descriptive statistics will be calculated at baseline and for observed values and changes from baseline at each scheduled timepoint. Frequency tabulations of the abnormalities will be made.

11.9. Interim Analysis

An Interim Analysis Committee will be responsible for performing the formal interim analysis and will consist of sponsor personnel who are not otherwise involved in the study. The IDMC will review the data and make recommendations to the Sponsor Committee, which will be responsible for identifying appropriate actions based on the recommendations of the IDMC.

Two types of interim analyses are distinguished: formal interim analysis and interim analyses performed by the IDMC only. The IDMC is described in Section 11.10, Independent Data Monitoring Committee, and Section 3.1, Overview of Study Design. At any formal interim analysis, the IDMC will also perform a data review.

The Interim Analysis Committee will be responsible for performing any formal interim analysis and will consist of sponsor personnel who are not otherwise involved in study activities. The first formal interim analysis will be performed when 36 subjects with ≤3 days of RSV symptoms have completed treatment or when 90 subjects with >3 to ≤5 days of RSV symptoms have been enrolled and have completed treatment. The second interim analysis may be performed at the discretion of the sponsor when at least 120 subjects have completed treatment (or at least 60 subjects with ≤3 days of RSV symptoms), which may allow an analysis at the end of a season.

The following situations will be considered based on the results of any formal interim analysis: that the study is considered futile; that superiority can be concluded; that subjects with >3 to ≤5 days of RSV symptoms at randomization may not achieve benefit (which may also be limited to ≤4 days of onset to symptoms); that the treatment duration needs to be increased to up to 10 days; or that the study should continue unchanged. In the case of the (unbinding) futility boundary or the superiority boundary being crossed, the IDMC may recommend early termination to the Sponsor Committee. If early superiority on the primary endpoint can be established, the study may be continued in order to accumulate data to allow the selection of the dose regimen or further substantiate the benefit-risk profile in this population; in this case the randomization ratio of active to placebo treatment may be altered for example, if dose selection is evident from the dose-response analysis, subjects randomized to active treatment may all receive the optimal dose. Alternative randomization ratios can be endorsed by the IDMC and implemented by the Sponsor Committee for Option A are 1:2:0, 1:0:2, 1:2:2, and 2:1:3 for the placebo, low dose, and high dose, respectively. The IDMC may recommend that the randomization ratios for Option B be changed.

Results of the formal interim analyses can be used in interactions with Health Authorities.

11.10. Independent Data Monitoring Committee

An IDMC will be established to monitor data and will review data in an unblinded manner on a regular basis to ensure the continuing safety of the subjects enrolled in this study. The IDMC will review the data and make recommendations to the Sponsor Committee, which will be responsible for identifying appropriate actions based on the recommendations of the IDMC. Details are provided in the IDMC Charter.

The IDMC will consist of at least 1 medical expert in the relevant therapeutic area and at least 1 statistician, 1 of whom will chair the committee. The IDMC responsibilities, authorities, and procedures will be documented in the IDMC Charter. Other members may be required dependent on the nature of the interim analysis. The intent is to have the same IDMC monitoring this study as for Study ALS-8176-503 given their familiarity with the compound. The IDMC will make dose recommendations at the beginning of the study, which will help determine which design option is chosen.

The IDMC will review the PK and safety data at the timepoints specified in Section 3.1, Overview of Study Design, and will continue to assess the PK, safety, and (selected) efficacy data during the conduct of the study. The frequency of IDMC reviews will be detailed in the IDMC Charter. At any formal interim analysis, the IDMC will also perform a data review.

Given the challenges of ensuring study-site preparation in time to recruit for an acute seasonal infection like RSV and the delays caused by implementing an amendment during an RSV season, the duration of dosing of lumicitabine may be modified by recommendation of the IDMC based on the review of this study and data from other ongoing studies of lumicitabine. The duration of dosing if extended will not exceed 10 days and the doses will not exceed those resulting in an expected average JNJ-63549109 plasma AUC_{0-24h} of 20,000 ng.h/mL (or 25,200 ng.h/mL in whole blood), a limit that is 2.7-fold lower than the lowest systemic NOAEL exposure observed in nonclinical toxicity studies. If the IDMC determines that subjects with a duration of RSV symptoms of 5 days prior to randomization may not achieve benefit based on interim results, they may recommend that the time of onset of RSV symptoms to the time of randomization is reduced. In addition, if the IDMC determines that subjects with a duration of RSV symptoms longer than 5 days prior to randomization may achieve benefit, this duration may be increased to up to 7 days (ie, 7 days from symptom onset to randomization). If such modifications, including changes to the randomization ratio, are recommended by the IDMC and endorsed by the Sponsor Committee, these changes will be communicated in writing to investigators, health authorities, and IEC/IRB and may be implemented without amendment to this protocol. Sponsor personnel involved in this process will not be involved in the conduct of the study. Details are provided in the IDMC Charter.

12. ADVERSE EVENT REPORTING

Timely, accurate, and complete reporting and analysis of safety information from clinical studies are crucial for the protection of subjects, investigators, and the sponsor, and are mandated by regulatory agencies worldwide. The sponsor has established Standard Operating Procedures in conformity with regulatory requirements worldwide to ensure appropriate reporting of safety information; all clinical studies conducted by the sponsor or its affiliates will be conducted in accordance with those procedures.

Method of Detecting Adverse Events and Serious Adverse Events

Care will be taken not to introduce bias when detecting AEs or SAEs. Open-ended and nonleading verbal questioning of the subject's parent(s)/caregiver(s) is the preferred method to inquire about AE occurrence.

12.1. Definitions

12.1.1. Adverse Event Definitions and Classifications

Adverse Event

An AE is any untoward medical occurrence in a clinical study subject administered a medicinal (investigational or non-investigational) product. An AE does not necessarily have a causal relationship with the treatment. An AE can therefore be any unfavorable and unintended sign (including an abnormal finding), symptom, or disease temporally associated with the use of a medicinal (investigational or non-investigational) product, whether or not related to that medicinal (investigational or non-investigational) product. (Definition per International Conference on Harmonisation of Technical Requirements for Pharmaceuticals for Human Use [ICH]).

This includes any occurrence that is new in onset or aggravated in severity or frequency from the baseline condition, or abnormal results of diagnostic procedures, including laboratory test abnormalities.

Note: The sponsor collects AEs starting with the signing of the ICF (refer to Section 12.3.1, All Adverse Events, for time of last AE recording).

Serious Adverse Event

An SAE based on ICH and European Union Guidelines on Pharmacovigilance for Medicinal Products for Human Use is any untoward medical occurrence that at any dose:

- Results in death.
- Is life-threatening (The subject was at risk of death at the time of the event. It does not refer to an event that hypothetically might have caused death if it were more severe.)
- Requires inpatient hospitalization or prolongation of existing hospitalization.
- Results in persistent or significant disability/incapacity.
- Is a congenital anomaly/birth defect.
- Is a suspected transmission of any infectious agent via a medicinal product.
- Is medically important*.

*Medical and scientific judgment should be exercised in deciding whether expedited reporting is also appropriate in other situations, such as important medical events that may not be immediately life-threatening or result in death or hospitalization but may jeopardize the subject or may require intervention to prevent one of the other outcomes listed in the definition above. These should usually be considered serious.

If a serious and unexpected AE occurs for which there is evidence suggesting a causal relationship between the study drug and the event (eg, death from anaphylaxis), the event must

be reported as a serious and unexpected suspected adverse reaction even if it is a component of the study endpoint (eg, all-cause mortality).

Unlisted (Unexpected) Adverse Event/Reference Safety Information

An AE is considered unlisted if the nature or severity is not consistent with the applicable product reference safety information. For lumicitabine, the expectedness of an AE will be determined by whether or not it is listed in the IB.

Adverse Event Associated With the Use of the Drug

An AE is considered associated with the use of the drug if the attribution is possible, probable, or very likely by the definitions listed in Section 12.1.2, Attribution Definitions.

12.1.2. Attribution Definitions

Not Related

An AE that is not related to the use of the drug.

Doubtful

An AE for which an alternative explanation is more likely, eg, concomitant drug(s), concomitant disease(s), or the relationship in time suggests that a causal relationship is unlikely.

Possible

An AE that might be due to the use of the drug. An alternative explanation, eg, concomitant drug(s), concomitant disease(s), is inconclusive. The relationship in time is reasonable; therefore, the causal relationship cannot be excluded.

Probable

An AE that might be due to the use of the drug. The relationship in time is suggestive (eg, confirmed by dechallenge). An alternative explanation is less likely, eg, concomitant drug(s), concomitant disease(s).

Very Likely

An AE that is listed as a possible adverse reaction and cannot be reasonably explained by an alternative explanation, eg, concomitant drug(s), concomitant disease(s). The relationship in time is very suggestive (eg, it is confirmed by dechallenge and rechallenge).

12.1.3. Severity Criteria

An assessment of severity grade will be made using the DMID categorical descriptors; for abnormalities NOT found elsewhere in the toxicity tables (see Attachment 1) the scale below is to be used for the estimation of severity:

GRADE 1	**Mild**: Transient of mild discomfort (<48 hours); no medical intervention/therapy required.
GRADE 2	**Moderate**: Mild to moderate limitation in activity, some assistance may be needed; no or minimal medical intervention/therapy required.
GRADE 3	**Severe**: Marked limitation in activity, some assistance usually required; medical intervention/therapy required, hospitalizations possible.
GRADE 4	**Life-threatening or death**: Extreme limitation in activity, significant assistance required; significant medical intervention/therapy required, hospitalization (or hospice) care probable.

12.2. Special Reporting Situations

Safety events of interest on a sponsor study drug that may require expedited reporting and/or safety evaluation include, but are not limited to:

- Overdose of a sponsor study drug.
- Suspected abuse/misuse of a sponsor study drug.
- Accidental or occupational exposure to a sponsor study drug.
- Medication error involving a sponsor product (with or without subject exposure to the sponsor study drug, eg, name confusion).
- Exposure to a sponsor study drug from breastfeeding.

Special reporting situations should be recorded in the eCRF. Any special reporting situation that meets the criteria of an SAE should be recorded on the SAE page of the eCRF.

12.3. Procedures

12.3.1. All Adverse Events

All AEs and special reporting situations, whether serious or non-serious, will be reported from the time a signed and dated ICF is obtained until completion of the subject’s last study-related procedure, which may include contact for follow-up of safety. Serious adverse events, including those spontaneously reported to the investigator within 30 days after the last dose of study drug, must be reported using the SAE form. The sponsor will evaluate any safety information that is spontaneously reported by an investigator beyond the time frame specified in the protocol.

All events that meet the definition of an SAE will be reported as SAEs, regardless of whether they are protocol-specific assessments.

All AEs, regardless of seriousness, severity, or presumed relationship to study drug, must be recorded using medical terminology in the source document and the eCRF. Whenever possible, diagnoses should be given when signs and symptoms are due to a common etiology (eg, cough, runny nose, sneezing, sore throat, and head congestion should be reported as “upper respiratory infection”). Investigators must record in the eCRF their opinion concerning the relationship of the AE to study therapy. All measures required for AE management must be recorded in the source document and reported according to sponsor instructions.

The sponsor assumes responsibility for appropriate reporting of AEs to the regulatory authorities. The sponsor will also report to the investigator (and the head of the investigational institute where required) all suspected unexpected serious adverse reactions (SUSARs). The investigator (or sponsor where required) must report SUSARs to the appropriate IEC/IRB that approved the protocol unless otherwise required and documented by the IEC/IRB. A SUSAR will be reported to regulatory authorities unblinded. Participating investigators and IEC/IRB will receive a blinded SUSAR summary, unless otherwise specified.

For all studies with an outpatient phase, the subject’s parent(s)/caregiver(s) must be provided with a “wallet (study) card” and instructed to carry this card with them for the duration of the study indicating the following:

- Study number.
- Statement, in the local language(s), that the subject is participating in a clinical study.
- Investigator’s name and 24-hour contact telephone number.
- Local sponsor’s name and 24-hour contact telephone number (for medical staff only).
- Site number.
- Subject number.
- Any other information that is required to do an emergency breaking of the blind.

12.3.2. Serious Adverse Events

All SAEs occurring during the study must be reported to the appropriate sponsor contact person by the investigator/study-site personnel within 24 hours of their knowledge of the event.

Information regarding SAEs will be transmitted to the sponsor using the SAE form, which must be completed and signed by a physician from the study site, and transmitted to the sponsor within 24 hours. The initial and follow-up reports of an SAE should be made by facsimile (fax).

All SAEs that have not resolved by the end of the study, or that have not resolved upon discontinuation of the subject's participation in the study, must be followed until any of the following occurs:

- The event resolves.
- The event stabilizes.
- The event returns to baseline, if a baseline value/status is available.
- The event can be attributed to agents other than the study drug or to factors unrelated to study conduct.
- It becomes unlikely that any additional information can be obtained (subject or health care practitioner refusal to provide additional information, lost to follow-up after demonstration of due diligence with follow-up efforts).

Suspected transmission of an infectious agent by a medicinal product will be reported as an SAE. Any event requiring hospitalization (or prolongation of hospitalization) that occurs during the course of a subject's participation in a study must be reported as an SAE, except hospitalizations for the following:

- Hospitalizations not intended to treat an acute illness or AE (eg, social reasons such as pending placement in long-term care facility).
- Surgery or procedure planned before entry into the study (must be documented in the eCRF). Note: Hospitalizations that were planned before the signing of the ICF, and where the underlying condition for which the hospitalization was planned has not worsened, will not be considered SAEs. Any AE that results in a prolongation of the originally planned hospitalization is to be reported as a new SAE.
- For convenience the investigator may choose to hospitalize the subject for the duration of the treatment period.

If the subject is rehospitalized after discharge for the same RSV disease, this will constitute an SAE.

The cause of death of a subject in a study within 30 days of the last dose of study drug, whether or not the event is expected or associated with the study drug, is considered an SAE.

12.4. Contacting Sponsor Regarding Safety

The names (and corresponding telephone numbers) of the individuals who should be contacted regarding safety issues or questions regarding the study are listed in the Contact Information page(s), which will be provided as a separate document.

13. PRODUCT QUALITY COMPLAINT HANDLING

A product quality complaint (PQC) is defined as any suspicion of a product defect related to manufacturing, labeling, or packaging, ie, any dissatisfaction relative to the identity, quality, durability, or reliability of a product, including its labeling or package integrity. A PQC may have an impact on the safety and efficacy of the product. Timely, accurate, and complete reporting and analysis of PQC information from studies are crucial for the protection of subjects, investigators, and the sponsor, and are mandated by regulatory agencies worldwide. The sponsor has established procedures in conformity with regulatory requirements worldwide to ensure appropriate reporting of PQC information; all studies conducted by the sponsor or its affiliates will be conducted in accordance with those procedures.

13.1. Procedures

All initial PQCs must be reported to the sponsor by the investigator/study-site personnel within 24 hours after being made aware of the event.

If the defect is combined with an SAE, the investigator/study-site personnel must report the PQC to the sponsor according to the SAE reporting timelines (refer to Section 12.3.2, Serious Adverse Events). A sample of the suspected product should be maintained for further investigation if requested by the sponsor.

13.2. Contacting Sponsor Regarding Product Quality

The names (and corresponding telephone numbers) of the individuals who should be contacted regarding product quality issues are listed in the Contact Information page(s), which will be provided as a separate document.

14. STUDY DRUG INFORMATION

14.1. Physical Description of Study Drug(s)

The lumicitabine and matching placebo supplied for this study is the pediatric formulation, which is a 1,819 mg powder for oral suspension. The powder for oral suspension (active and matching placebo) will be reconstituted with 30 mL of lumicitabine solvent (for a final concentration of 60 mg/ml) by the study-site pharmacist prior to administration, as described in the pharmacy manual.

Lumicitabine and placebo powders and solvent for oral suspension will be manufactured and provided under the responsibility of the sponsor. Excipient of the active powder for oral suspension: colloidal anhydrous silica. Excipients of the placebo powder for oral suspension: silicified micro crystalline cellulose. Excipients of the solvent for oral suspension: microcrystalline cellulose and carboxymethylcellulose sodium, simethicone, sucralose, polysorbate 20, sodium methyl parahydroxybenzoate, sodium ethyl parahydroxybenzoate, trisodium citrate dehydrate, citric acid anhydrous, strawberry flavor PHS-120116, and purified water.

14.2. Packaging

The active and placebo powders and solvent for oral suspension are packaged in amber glass bottles (type III) with a polypropylene/low density polyethylene child-resistant screw cap.

14.3. Labeling

Study drug labels will contain information to meet the applicable regulatory requirements.

14.4. Preparation, Handling, and Storage

All study drug must be stored according to instructions on the clinical label. The 60 mg/mL reconstituted suspension formulation can be kept at room temperature.

Refer to the pharmacy manual/study-site investigational product and procedures manual for additional guidance on study drug preparation, handling, and storage.

14.5. Drug Accountability

The investigator is responsible for ensuring that all study drug received at the site is inventoried and accounted for throughout the study. The study drug administered to the subject while the subject is hospitalized must be documented on the drug accountability form. The dispensing of study drug to the subject's parent(s)/caregiver(s), and the return of study drug from the subject's parent(s)/caregiver(s) (if applicable), must be documented on the drug accountability form. The subject's parent(s)/caregiver(s) must be instructed to return all original containers, whether empty or containing study drug. All study drug will be stored and disposed of according to the sponsor's instructions. The investigator/study-site personnel must not combine contents of the study drug containers.

Study drug must be handled in strict accordance with the protocol and the container label, and must be stored at the study site in a limited-access area or in a locked cabinet under appropriate environmental conditions. Unused study drug, and study drug returned by the subject's parent(s)/caregiver(s) must be available for verification by the sponsor's study-site monitor during on-site monitoring visits. The return to the sponsor of unused study drug or used returned study drug for destruction will be documented on the drug return form. When the study site is an authorized destruction unit and study drug supplies are destroyed on-site, this must also be documented on the drug return form.

Study drug should be dispensed under the supervision of the investigator or a qualified member of the study-site personnel, or by a hospital/clinic pharmacist. Study drug will be supplied only to subjects participating in the study. Returned study drug must not be dispensed again, even to the same subject. Study drug may not be relabeled or reassigned for use by other subjects. The investigator agrees neither to dispense the study drug from, nor store it at, any site other than the study sites agreed upon with the sponsor.

15. STUDY-SPECIFIC MATERIALS

The investigator will be provided with the following supplies:

- Lumicitabine IB.
- Pharmacy manual/study-site investigational product and procedures manual.
- Laboratory manual.
- Electronic device for clinician and parent(s)/caregiver(s) eCOA.
- Completion guide(s) for clinician and parent(s)/caregiver(s) eCOA.
- PCR assay for RSV diagnosis (if needed).
- Specimen collection kits for blood (PK and biomarker host RNA) and mid-turbinate nasal swab.
- Contact information page(s).
- Oral dispensers.
- IWRS manual.
- eCRF completion guidelines.
- Sample ICF.

16. ETHICAL ASPECTS

16.1. Study-specific Design Considerations

The potential subject's legally acceptable representative will be fully informed of the risks and requirements of the study and, during the study, subjects' parent(s)/caregiver(s) will be given any new information that may affect their decision to continue participation. They will be told that their consent to participate in the study is voluntary and may be withdrawn at any time with no reason given and without penalty or loss of benefits to which they would otherwise be entitled. Only subjects whose parent(s)/caregiver(s) are fully able to understand the risks, benefits, and potential AEs of the study and provide their consent voluntarily will be enrolled.

When referring to the signing of the ICF, the terms legal guardian and legally acceptable representative refer to the legally appointed guardian of the child with authority to authorize participation in research. For each subject, his or her parent(s) (both parents, if available or if required according to local regulations) or a legally acceptable representative(s), as required by local regulations, must give written consent (permission) according to local requirements after the nature of the study has been fully explained and before the performance of any study-related assessments. For the purposes of this study, all references to subjects who have provided consent (and assent as applicable) refers to the subjects and his or her parent(s) or the subject's legal guardian(s) or legally acceptable representative(s) who have provided consent according to this process.

The total blood volume to be collected is considered to be in line with generally acceptable guidelines for the collection of blood samples for these age groups.[13] For each PK sample, 0.2 mL of blood for capillary sampling and 1.0 mL of blood for sampling via arterial line are required. The amount of blood for the safety samples will depend on local practice (eg, heel or finger prick versus venous sample); however, sites are encouraged to limit the volume as much as possible, while still performing the required safety testing.

Blood sampling has been minimized in this study to reduce the discomfort to the subjects. The maximum amount of blood drawn from each subject for study-specific purposes will not exceed 3 mL/kg body weight over the duration of the study in line with recommendations collated by the World Health Organization[13] or less if per local practice guidelines. Sampling of blood for PK is limited to a maximum of 3 samples at different timepoints which have been carefully selected to yield the maximum information with the least number of samples. Pharmacokinetic sampling can be performed by venipuncture or by heel or finger prick or alternatively through a venous/arterial line with collection of capillary blood as detailed in the laboratory manual.

Sampling for safety is also limited, with screening safety assessments in view of eligibility determination allowed to be performed using standard of care samples taken prior to study entry. Also safety assessments performed as part of standard of care, when within the protocol visit window, can be used as for the safety follow-up during the study. Both of these measures aim at minimizing the number of specific sampling timepoints as well as blood volume drawn. Furthermore, type of laboratory assessments for safety have been limited to the relevant ones in view of the (pre)clinical safety profile of lumicitabine, limiting the amount of blood needed and hence the duration of the sampling.

In addition, investigational staff should take the customary measures to ensure that study-specific assessments such as blood sampling are performed with as little additional stress as possible for the subject.

16.2. Regulatory Ethics Compliance

16.2.1. Investigator Responsibilities

The investigator is responsible for ensuring that the study is performed in accordance with the protocol, current ICH guidelines on Good Clinical Practice (GCP), and applicable regulatory and country-specific requirements.

Good Clinical Practice is an international ethical and scientific quality standard for designing, conducting, recording, and reporting studies that involve the participation of human subjects. Compliance with this standard provides public assurance that the rights, safety, and well-being of study subjects are protected, consistent with the principles that originated in the Declaration of Helsinki, and that the study data are credible.

16.2.2. Independent Ethics Committee or Institutional Review Board

Before the start of the study, the investigator (or sponsor where required) will provide the IEC/IRB with current and complete copies of the following documents (as required by local regulations):

- Final protocol and, if applicable, amendments.
- Sponsor-approved ICF (and any other written materials to be provided to the subjects).
- IB (or equivalent information) and amendments/addenda.
- Sponsor-approved subject recruiting materials.
- Information on compensation for study-related injuries or payment to subjects for participation in the study, if applicable.
- Investigator's curriculum vitae or equivalent information (unless not required, as documented by the IEC/IRB).
- Information regarding funding, name of the sponsor, institutional affiliations, other potential conflicts of interest, and incentives for subjects.
- Written information provided to subjects' parent(s)/caregiver(s) (eg, diaries, eCOA).
- Any other documents that the IEC/IRB requests to fulfill its obligation.

This study will be undertaken only after the IEC/IRB has given full approval of the final protocol, amendments (if any, excluding the ones that are purely administrative, with no consequences for subjects, data, or study conduct unless required locally), the ICF, applicable recruiting materials, and subject compensation programs, and the sponsor has received a copy of this approval. This approval letter must be dated and must clearly identify the IEC/IRB and the documents being approved.

During the study, the investigator (or sponsor where required) will send the following documents and updates to the IEC/IRB for their review and approval, where appropriate:

- Protocol amendments (excluding the ones that are purely administrative, with no consequences for subjects, data, or study conduct).
- Revision(s) to ICF and any other written materials to be provided to subjects.
- If applicable, new or revised subject recruiting materials approved by the sponsor.
- Revisions to compensation for study-related injuries or payment to subjects for participation in the study, if applicable.
- New edition(s) of the IB and amendments/addenda.
- Summaries of the status of the study at intervals stipulated in guidelines of the IEC/IRB (at least annually).
- Reports of AEs that are serious, unlisted/unexpected, and associated with the study drug.
- New information that may adversely affect the safety of the subjects or the conduct of the study.

- Deviations from or changes to the protocol to eliminate immediate hazards to the subjects.
- Report of deaths of subjects under the investigator's care.
- Notification if a new investigator is responsible for the study at the site.
- Development Safety Update Report and Line Listings, where applicable.
- Any other requirements of the IEC/IRB.

For all protocol amendments (excluding the ones that are purely administrative, with no consequences for subjects, data, or study conduct), the amendment and applicable ICF revisions must be submitted promptly to the IEC/IRB for review and approval before implementation of the change(s).

At least once a year, the IEC/IRB will be asked to review and reapprove this study, where required.

At the end of the study, the investigator (or sponsor where required) will notify the IEC/IRB about the study completion (if applicable, the notification will be submitted through the head of investigational institution).

16.2.3. Informed Consent Form

Each legally acceptable representative must give written consent according to local requirements after the nature of the study has been fully explained. The ICF must be signed before performance of any study-related activity. The ICF that is used must be approved by both the sponsor and the reviewing IEC/IRB and be in a language that the parent(s)/caregiver(s) can read and understand. The informed consent should be in accordance with principles that originated in the Declaration of Helsinki, current ICH and GCP guidelines, applicable regulatory requirements, and sponsor policy.

Before enrollment in the study, the investigator or an authorized member of the study-site personnel must explain to potential subjects or their legally acceptable representatives the aims, methods, reasonably anticipated benefits, potential hazards of the study, and any discomfort participation in the study may entail. Subject's legally acceptable representatives will be informed that subject participation is voluntary and that they may withdraw consent to participate at any time. They will be informed that choosing not to participate will not affect the care the subject will receive for the treatment of his or her disease. Finally, they will be told that the investigator will maintain a subject identification register for the purposes of long-term follow-up if needed, and that their records may be accessed by health authorities and authorized sponsor personnel without violating the confidentiality of the subject, to the extent permitted by the applicable law(s) or regulations. By signing the ICF the subject's legally acceptable representative is authorizing such access, which includes permission to obtain information about the subject's survival status. It also denotes that the subject's legally acceptable representative agrees to allow his or her study physician to re-contact the subject for the purpose of obtaining consent for additional safety evaluations, and subsequent disease-related treatments, if needed.

The subject's legally acceptable representative will be given sufficient time to read the ICF and the opportunity to ask questions. After this explanation and before entry into the study, consent

should be appropriately recorded by means of the subject's legally acceptable representative's personally dated signature. After having obtained the consent, a copy of the ICF must be given to the subject's legally acceptable representative.

If the subject's legally acceptable representative is unable to read or write, an impartial witness should be present for the entire informed consent process (which includes reading and explaining all written information) and should personally date and sign the ICF after the oral consent of the subject's legally acceptable representative is obtained.

When prior consent of the subject's legally acceptable representative is not possible, enrollment procedures should be described in the protocol with documented approval/favorable opinion by the IEC/IRB to protect the rights, safety, and well-being of the subject and to ensure compliance with applicable regulatory requirements. The subject's legally acceptable representative must be informed about the study as soon as possible and give consent to continue.

16.2.4. Privacy of Personal Data

The collection and processing of personal data from subjects enrolled in this study will be limited to those data that are necessary to fulfill the objectives of the study.

These data must be collected and processed with adequate precautions to ensure confidentiality and compliance with applicable data privacy protection laws and regulations. Appropriate technical and organizational measures to protect the personal data against unauthorized disclosures or access, accidental or unlawful destruction, or accidental loss or alteration must be put in place. Sponsor personnel whose responsibilities require access to personal data agree to keep the identity of subjects confidential.

The informed consent obtained from the subject's legally acceptable representative includes explicit consent for the processing of personal data and for the investigator/institution to allow direct access to his or her original medical records (source data/documents) for study-related monitoring, audit, IEC/IRB review, and regulatory inspection. This consent also addresses the transfer of the data to other entities and to other countries.

The subject's legally acceptable representative has the right to request through the investigator access to his or her personal data and the right to request rectification of any data that are not correct or complete. Reasonable steps will be taken to respond to such a request, taking into consideration the nature of the request, the conditions of the study, and the applicable laws and regulations.

Exploratory biomarker and PK research is not conducted under standards appropriate for the return of data to subjects. In addition, the sponsor cannot make decisions as to the significance of any findings resulting from exploratory research. Therefore, exploratory research data will not be returned to subjects or investigators, unless required by law or local regulations. Privacy and confidentiality of data generated in the future on stored samples will be protected by the same standards applicable to all other clinical data.

16.2.5. Long-term Retention of Samples for Additional Future Research

Samples collected in this study may be stored for up to 15 years (or according to local regulations) for additional research. Samples will only be used to understand lumicitabine, to understand RSV, to understand differential drug responders, and to develop tests/assays related to lumicitabine or other RSV compounds and RSV disease. The research may begin at any time during the study or the poststudy storage period and might be conducted by the sponsor or with sponsor's collaborators and/or vendors.

Stored samples will be coded throughout the sample storage and analysis process and will not be labeled with personal identifiers. Subjects may withdraw their consent for their samples to be stored for research (refer to Section 10.3, Withdrawal From the Use of Samples in Future Research.

16.2.6. Country Selection

This study will only be conducted in those countries where the intent is to launch or otherwise help ensure access to the developed product if the need for the product persists, unless explicitly addressed as a specific ethical consideration in Section 16.1, Study-specific Design Considerations.

17. ADMINISTRATIVE REQUIREMENTS

17.1. Protocol Amendments

Neither the investigator nor the sponsor will modify this protocol without a formal amendment by the sponsor. All protocol amendments must be issued by the sponsor, and signed and dated by the investigator. Protocol amendments must not be implemented without prior IEC/IRB approval, or when the relevant competent authority has raised any grounds for non-acceptance, except when necessary to eliminate immediate hazards to the subjects, in which case the amendment must be promptly submitted to the IEC/IRB and relevant competent authority. Documentation of amendment approval by the investigator and IEC/IRB must be provided to the sponsor. When the change(s) involves only logistic or administrative aspects of the study, the IEC/IRB (where required) only needs to be notified.

The doses or treatment regimen of lumicitabine may be modified by recommendation of the IDMC at any time for PK, efficacy, or safety reasons based on the review of this and other ongoing studies of lumicitabine. If such modifications are recommended based on IDMC review, these changes will be communicated in writing to investigators, health authorities, and ethics committees and may be implemented without amendment to this protocol.

During the course of the study, in situations where a departure from the protocol is unavoidable, the investigator or other physician in attendance will contact the appropriate sponsor representative listed in the Contact Information page(s), which will be provided as a separate document. Except in emergency situations, this contact should be made before implementing any departure from the protocol. In all cases, contact with the sponsor must be made as soon as possible to discuss the situation and agree on an appropriate course of action. The data recorded

in the eCRF and source documents will reflect any departure from the protocol, and the source documents will describe this departure and the circumstances requiring it.

17.2. Regulatory Documentation

17.2.1. Regulatory Approval/Notification

This protocol and any amendment(s) must be submitted to the appropriate regulatory authorities in each respective country, if applicable. A study may not be initiated until all local regulatory requirements are met.

17.2.2. Required Prestudy Documentation

The following documents must be provided to the sponsor before shipment of study drug to the study site:

- Protocol and amendment(s), if any, signed and dated by the Principal Investigator.
- A copy of the dated and signed (or sealed, where appropriate per local regulations), written IEC/IRB approval of the protocol, amendments, ICF, any recruiting materials and if applicable, subject compensation programs. This approval must clearly identify the specific protocol by title and number and must be signed (or sealed, where appropriate per local regulations) by the chairman or authorized designee.
- Name and address of the IEC/IRB, including a current list of the IEC/IRB members and their function, with a statement that it is organized and operates according to GCP and the applicable laws and regulations. If accompanied by a letter of explanation, or equivalent, from the IEC/IRB, a general statement may be substituted for this list. If an investigator or a member of the study-site personnel is a member of the IEC/IRB, documentation must be obtained to state that this person did not participate in the deliberations or in the vote/opinion of the study.
- Regulatory authority approval or notification, if applicable.
- Signed and dated statement of investigator (eg, Form FDA 1572), if applicable.
- Documentation of investigator qualifications (eg, curriculum vitae).
- Completed investigator financial disclosure form from the Principal Investigator, where required.
- Signed and dated clinical study agreement, which includes the financial agreement.
- Any other documentation required by local regulations.

The following documents must be provided to the sponsor before enrollment of the first subject:

- Completed investigator financial disclosure forms from all subinvestigators.
- Documentation of subinvestigator qualifications (eg, curriculum vitae).
- Name and address of any local laboratory conducting tests for the study, and a dated copy of current laboratory normal ranges for these tests, if applicable.

- Local laboratory documentation demonstrating competence and test reliability (eg, accreditation/license), if applicable.

17.3. Subject Identification, Enrollment, and Screening Logs

The investigator agrees to complete a subject identification and enrollment log to permit easy identification of each subject during and after the study. This document will be reviewed by the sponsor study-site contact for completeness.

The subject identification and enrollment log will be treated as confidential and will be filed by the investigator in the study file. To ensure subject confidentiality, no copy will be made. All reports and communications relating to the study will identify subjects by subject identification and date of birth. In cases where the subject is not randomized into the study, the date seen and date of birth will be used.

The investigator must also complete a subject screening log, which reports on all subjects who were seen to determine eligibility for inclusion in the study.

17.4. Source Documentation

At a minimum, source documents consistent in the type and level of detail with that commonly recorded at the study site as a basis for standard medical care must be available for the following: subject identification, eligibility, and study identification; study discussion and date of signed informed consent; dates of visits; results of safety and efficacy parameters as required by the protocol; record of all AEs and follow-up of AEs; concomitant medication(s); drug receipt/dispensing/return records; study drug administration information; and date of study completion and reason for early discontinuation of study drug or withdrawal from the study, if applicable.

Additionally, investigator-completed scales and assessments and parent(s)/caregiver(s)-reported outcomes (data for the clinician or parent[s]/caregiver[s] eCOA) will be captured directly in the electronic device, which will serve as source documentation.

The author of an entry in the source documents should be identifiable.

Specific details required as source data for the study and source data collection methods will be reviewed with the investigator before the start of the study and will be described in the monitoring guidelines (or other equivalent document).

The following data will be recorded directly into the eCRF and will be considered source data:

- Race.
- Blood pressure and heart rate.
- Height and weight.
- Details of physical examination.

The minimum source documentation requirements for Section 4.1, Inclusion Criteria, and Section 4.2, Exclusion Criteria, that specify a need for documented medical history are as follows:

- Discharge summaries.

Inclusion and exclusion criteria not requiring documented medical history must be verified at a minimum by subject interview or other protocol-required assessment (eg, physical examination, laboratory assessment) and documented in the source documents.

An electronic source system may be utilized, which contains data traditionally maintained in a hospital or clinic record to document medical care (eg, electronic source documents) as well as the clinical study-specific data fields as determined by the protocol. These data are electronically extracted for use by the sponsor. If the electronic source system is utilized, references made to the eCRF in the protocol include the electronic source system but information collected through the electronic source system may not be limited to that found in the eCRF. Data in this system may be considered source documentation.

17.5. Case Report Form Completion

Case report forms are prepared and provided by the sponsor for each subject in electronic format. All eCRF entries, corrections, and alterations must be made by the investigator or authorized study-site personnel. The investigator must verify that all data entries in the eCRF are accurate and correct.

The study data will be transcribed by the investigator/study-site personnel from the source documents into the eCRF, if applicable. Study-specific data will be transmitted in a secure manner to the sponsor.

Worksheets may be used for the capture of some data to facilitate completion of the eCRF. Any such worksheets will become part of the subject's source documents. Data must be entered into eCRF in English. The eCRF must be completed as soon as possible after a subject visit and the forms should be available for review at the next scheduled monitoring visit.

All subjective measurements (eg, pain scale information or other questionnaires) will be completed by the same individual who made the initial baseline determinations whenever possible.

If necessary, queries will be generated in the electronic data capture (eDC) tool. If corrections to an eCRF are needed after the initial entry into the eCRF, this can be done in either of the following ways:

- Investigator and study-site personnel can make corrections in the eDC tool at their own initiative or as a response to an auto query (generated by the eDC tool).
- Sponsor or sponsor delegate can generate a query for resolution by the investigator and study-site personnel.

17.6. Clinical Outcome Assessments

Clinical Outcome Assessments (see Attachment 5) will be completed by the clinician and parent(s)/caregiver(s) on an electronic device provided at the study site. Responses provided will be recorded directly in the electronic database. Each day from screening through end of follow-up, the investigator/study-site personnel will monitor whether the subject's eCOA data were uploaded by the parent(s)/caregiver(s) to the eCOA website provided for this study. If more than 36 hours have elapsed since the last eCOA assessment was recorded on the eCOA website, the investigator/study-site personnel will contact the subject's parent(s)/caregiver(s) to ensure that the eCOA questionnaire is completed and transmitted to the server every day, to identify problems collecting or transmitting the assessments, and to instruct the parent/caregiver entering the subject's responses on the eCOA device on the way to correct any problems with collecting or transmitting the eCOA data. Telephone calls to the subject's parent(s)/caregiver(s) to facilitate compliance with the study procedures between outpatient study visits are encouraged.

Study monitors should monitor completion of the eCOAs and follow-up with sites immediately should there be any indication that any eCOAs are not being completed or if data transfer is not being done as scheduled.

The eCOA device includes built-in training for study-site personnel, investigators, and parent(s)/caregiver(s).

17.6.1. Investigator

The investigator will provide information about the subject's status, symptoms, and behavior on an electronic device at timepoints noted in the Time and Events Schedules. Before completing the first assessment of any subject in the study, the investigator must complete the assigned eCOA training. Practical training on the electronic device will be available on demand.

17.6.2. Parent(s)/Caregiver(s)

The subject's parent(s)/caregiver(s) with routine and frequent experience caring for the subject will provide information about the subject's status, symptoms, and behaviors on an electronic device at timepoints noted in the Time and Events Schedules. In addition, after hospital discharge, the parent(s)/caregiver(s) will record study drug and concomitant medications administered on the electronic device in a medication log. All parent(s)/caregiver(s) assessments and logs will be provided in the native language of the parent(s)/caregiver(s). The electronic device will include instructions and training that will be completed upon first use by the parent(s)/caregiver(s) and will be available on demand thereafter if the parent(s)/caregiver(s) chooses this option. If the parent/caregiver is unable to complete the assessment on the device without assistance, the investigator/study-site personnel can read the questions and responses aloud to the parent/caregiver and enter the parent/caregiver's responses in the eCOA device on their behalf during hospitalization. Once a subject is discharged from the hospital, sites will follow procedures detailed in the eCOA manual for administration of the parent/caregiver eCOA by telephone interview post discharge. The investigator/study-site personnel will instruct the parent/caregiver to complete the eCOA questionnaire and transmit the eCOA data at approximately the same time each day from screening through end of follow-up.

17.7. Data Quality Assurance/Quality Control

Steps to be taken to ensure the accuracy and reliability of data include the selection of qualified investigators and appropriate study sites, review of protocol procedures with the investigator and study-site personnel before the study, and periodic monitoring visits by the sponsor and direct transmission of clinical laboratory data from a central laboratory into the sponsor's data base. Written instructions will be provided for collection, handling, storage, and shipment of samples.

Guidelines for eCRF completion will be provided and reviewed with the investigator/study-site personnel before the start of the study. The sponsor will review eCRFs for accuracy and completeness during on-site monitoring visits and after transmission to the sponsor; any discrepancies will be resolved with the investigator or designee, as appropriate. After upload of the data into the study database, the data will be verified for accuracy and consistency with the data sources.

17.8. Record Retention

In compliance with the ICH/GCP guidelines, the investigator/institution will maintain all eCRF and all source documents that support the data collected from each subject, as well as all study documents as specified in ICH/GCP Section 8, Essential Documents for the Conduct of a Clinical Trial, and all study documents as specified by the applicable regulatory requirement(s). The investigator/institution will take measures to prevent accidental or premature destruction of these documents.

Essential documents must be retained until at least 2 years after the last approval of a marketing application in an ICH region and until there are no pending or contemplated marketing applications in an ICH region or until at least 2 years have elapsed since the formal discontinuation of clinical development of the investigational product. These documents will be retained for a longer period if required by the applicable regulatory requirements or by an agreement with the sponsor. It is the responsibility of the sponsor to inform the investigator/institution as to when these documents no longer need to be retained.

If the responsible investigator retires, relocates, or for other reasons withdraws from the responsibility of keeping the study records, custody must be transferred to a person who will accept the responsibility. The sponsor must be notified in writing of the name and address of the new custodian. Under no circumstance shall the investigator relocate or dispose of any study documents before having obtained written approval from the sponsor.

If it becomes necessary for the sponsor or the appropriate regulatory authority to review any documentation relating to this study, the investigator/institution must permit access to such reports.

17.9. Monitoring

The sponsor will use a combination of monitoring techniques (central, remote, or on-site monitoring) to monitor this study.

The sponsor will perform on-site monitoring visits as frequently as necessary. The monitor will record dates of the visits in a study-site visit log that will be kept at the study site. The first post-initiation visit will be made as soon as possible after enrollment has begun. At these visits, the monitor will compare data entered into the eCRF with the source documents (eg, hospital/clinic/physician's office medical records); a sample may be reviewed. The nature and location of all source documents will be identified to ensure that all sources of original data required to complete the eCRF are known to the sponsor and the investigator/study-site personnel and are accessible for verification by the sponsor study-site contact. If electronic records are maintained at the study site, the method of verification must be discussed with the investigator/study-site personnel.

Direct access to source documents (medical records) must be allowed for the purpose of verifying that the recorded data are consistent with the original source data. Findings from this review will be discussed with the investigator/study-site personnel. The sponsor expects that, during monitoring visits, the relevant investigator/study-site personnel will be available, the source documents will be accessible, and a suitable environment will be provided for review of study-related documents. The monitor will meet with the investigator on a regular basis during the study to provide feedback on the study conduct.

In addition to on-site monitoring visits, remote contacts can occur. It is expected that during these remote contacts, the investigator/study-site personnel will be available to provide an update on the progress of the study at the site.

Central monitoring will take place for data identified by the sponsor as requiring central review.

17.10. Study Completion/Termination

17.10.1. Study Completion/End of Study

The study is considered completed with the last visit for the last subject participating in the study. The final data from the study site will be sent to the sponsor (or designee) after completion of the final subject visit at that study site, in the time frame specified in the Clinical Trial Agreement.

17.10.2. Study Termination

The sponsor reserves the right to close the study site or terminate the study at any time for any reason at the sole discretion of the sponsor. Study sites will be closed upon study completion. A study site is considered closed when all required documents and study supplies have been collected and a study-site closure visit has been performed.

The investigator may initiate study-site closure at any time, provided there is reasonable cause and sufficient notice is given in advance of the intended termination.

Reasons for the early closure of a study site by the sponsor or investigator may include but are not limited to:

- Failure of the investigator to comply with the protocol, the requirements of the IEC/IRB or local health authorities, the sponsor's procedures, or GCP guidelines.
- Inadequate recruitment of subjects by the investigator.
- Discontinuation of further study drug development.

17.11. On-site Audits

Representatives of the sponsor's clinical quality assurance department may visit the study site at any time during or after completion of the study to conduct an audit of the study in compliance with regulatory guidelines and company policy. These audits will require access to all study records, including source documents, for inspection. Subject privacy must, however, be respected. The investigator and study-site personnel are responsible for being present and available for consultation during routinely scheduled study-site audit visits conducted by the sponsor or its designees.

Similar auditing procedures may also be conducted by agents of any regulatory body, either as part of a national GCP compliance program or to review the results of this study in support of a regulatory submission. The investigator should immediately notify the sponsor if he or she has been contacted by a regulatory agency concerning an upcoming inspection.

17.12. Use of Information and Publication

The investigator understands that the information developed in the study will be used by the sponsor in connection with the continued development of lumicitabine, and thus may be disclosed as required to other clinical investigators or regulatory agencies. To permit the information derived from the clinical studies to be used, the investigator is obligated to provide the sponsor with all data obtained in the study.

The results of the study will be reported in a clinical study report generated by the sponsor and will contain data from all study sites that participated in the study as per-protocol. Recruitment performance or specific expertise related to the nature and the key assessment parameters of the study will be used to determine a coordinating investigator. Results of exploratory biomarker analyses performed after the clinical study report has been issued will be reported in a separate report and will not require a revision of the clinical study report. Study subject identifiers will

not be used in publication of results. Any work created in connection with performance of the study and contained in the data that can benefit from copyright protection (except any publication by the investigator as provided for below) shall be the property of the sponsor as author and owner of copyright in such work.

Consistent with Good Publication Practices and International Committee of Medical Journal Editors guidelines, the sponsor shall have the right to publish such primary (multicenter) data and information without approval from the investigator. The investigator has the right to publish study site-specific data after the primary data are published. If an investigator wishes to publish information from the study, a copy of the manuscript must be provided to the sponsor for review at least 60 days before submission for publication or presentation. Expedited reviews will be arranged for abstracts, poster presentations, or other materials. If requested by the sponsor in writing, the investigator will withhold such publication for up to an additional 60 days to allow for filing of a patent application. In the event that issues arise regarding scientific integrity or regulatory compliance, the sponsor will review these issues with the investigator. The sponsor will not mandate modifications to scientific content and does not have the right to suppress information. For multicenter study designs and substudy approaches, secondary results generally should not be published before the primary endpoints of a study have been published. Similarly, investigators will recognize the integrity of a multicenter study by not submitting for publication data derived from the individual study site until the combined results from the completed study have been submitted for publication, within 12 months of the availability of the final data (tables, listings, graphs), or the sponsor confirms there will be no multicenter study publication. Authorship of publications resulting from this study will be based on the guidelines on authorship, such as those described in the Uniform Requirements for Manuscripts Submitted to Biomedical Journals, which state that the named authors must have made a significant contribution to the design of the study or analysis and interpretation of the data, provided critical review of the paper, and given final approval of the final version.

Registration of Clinical Studies and Disclosure of Results

The sponsor will register and disclose the existence of and the results of clinical studies as required by law.

REFERENCES

1. American Academy of Pediatrics, Subcommittee on Diagnosis and Management of Bronchiolitis. Diagnosis and Management of Bronchiolitis. Pediatrics. 2006;118:1774-1793.

2. Adamo-Trigiani M. A 28/34-day study of ALS-008176 by oral gavage in juvenile rats with a 28/22-day recovery. Charles River 9000524 (Alios No. 518-R-14-ALS008176-PO-TX) 21 August 2015. >>

3. Adamo-Trigiani M. A 28-day toxicity and toxicokinetic study of ALS-008176 by twice daily oral gavage in the juvenile beagle dog with a 21-day recovery period. Charles River 9000508 (Alios No. 519-D-14-ALS008176-PO-TX). 19 August 2015.

4. Bazzoli C, Retout S, Mentré F. Design evaluation and optimisation in multiple response nonlinear mixed effect models: PFIM 3.0. Comput Methods Programs Biomed. 2010; 98(1):55-65.

5. Bloom-Feshbach K, Alonso WJ, Charu V, et al. Latitudinal variations in seasonal activity of influenza and respiratory syncytial virus (RSV): a global comparative review. PLoS One. 2013;8(2):e54445.

6. DeVincenzo JP, McClure MW, Symons JA, et al. Activity of oral ALS-008176 in a respiratory syncytial virus challenge study. N Eng J Med 2015; 373(21):2048-58.

7. Edwards T. An oral (gavage) dose range-finding toxicity study of ALS-008176 in juvenile rats. Alios Report 405-R-13-ALS008176-PO-TXR (WIL-800054). April 2014.

8. Edwards T. An oral (gavage) toxicity study of ALS-008176 in juvenile rats. Alios Report 500-R-13-ALS008176-PO-TX (WIL-800055). April 2014.

9. Feltes TF, Sondheimer HM. Palivizumab and the prevention of respiratory syncytial virus illness in pediatric patients with congenital heart disease. Expert Opin Biol Ther. 2007;7(9):1471-1480.

10. Hall CB. Respiratory syncytial virus and parainfluenza virus. N Engl J Med. 2001;344(25):1917-1928.

11. Hall CB, Weinberg GA, Iwane MK, et al. The burden of respiratory syncytial virus infection in young children. N Engl J Med. 2009; 360(6):588-598.

12. Hodges M, Salerno D, Erlien D. Bazett's QT correction reviewed: evidence that a linear QT correction for heart rate is better. J Am Coll Cardiol. 1983;1:694.

13. Howie SRC. Blood sample volumes in child health research: review of safe limits. 2011; 89(1):46-53 http://www.who.int/bulletin/volumes/89/1/BLT-10-080010-table-T2.html

14. International Conference on Harmonisation of Technical Requirements for Registration of Pharmaceuticals for Human Use. ICH Harmonized Tripartite Guideline E14: Clinical evaluation of QT/QTc interval prolongation and proarrhythmic potential for non-antiarrhythmic drugs. ICH 12 May 2005.

15. Lan KKG, DeMets DL. Discrete sequential boundaries for clinical trials. Biometrika. 1983;70:659-663.

16. Lumicitabine Investigator's Brochure Edition 7, June 2017.

17. Mallinckrodt CH, Lane PW, Schnell D, Peng Y, Mancuso JP, Recommendations for the Primary Analysis of Continuous Endpoints in Longitudinal Clinical Trials, Drug Information Journal, 2008; 42: 303-319,

18. Nair H, Nokes DJ, Gessner BD, et al. Global burden of acute lower respiratory infections due to respiratory syncytial virus in young children: a systematic review and meta-analysis. Lancet. 2010;375(9725):1545-1555.

19. Sagie A, Larson MG, Goldberg RJ, Bengston JR, Levy D. An improved method for adjusting the QT interval for heart rate (the Framingham Heart Study). Am J Cardiol. 1992;70(7):797-801.

20. SAS STAT® 9.22 User's Guide.

21. Synagis (palivizumab) EU Summary of Product Characteristics. AbbVie Ltd; 24 July 2015. https://www.medicines.org.uk/emc/medicine/5676, accessed 08 January 2016.

22. Yusuf S, Piedimonte G, Auais A, et al. The relationship of meteorological conditions to the epidemic activity of respiratory syncytial virus. Epidemiol Infect. 2007;135(7):1077-1090.

Attachment 1: Division of Microbiology and Infectious Diseases (DMID) Pediatric Toxicity Tables (November 2007)

DIVISION OF MICROBIOLOGY AND INFECTIOUS DISEASES (DMID) PEDIATRIC TOXICITY TABLES NOVEMBER 2007

ABBREVIATIONS: Abbreviations utilized in the Table:

ULN = Upper Limit of Normal	LLN = Lower Limit of Normal
R_x = Therapy	Req = Required
Mod = Moderate	IV = Intravenous
ADL = Activities of Daily Living	Dec = Decreased

ESTIMATING SEVERITY GRADE

For abnormalities NOT found elsewhere in the Toxicity Tables use the scale below to estimate grade of severity:

GRADE 1	Mild: Transient or mild discomfort (<48 hours); no medical intervention/therapy required
GRADE 2	Moderate: Mild to moderate limitation in activity - some assistance may be needed; no or minimal medical intervention/therapy required
GRADE 3	Severe: Marked limitation in activity, some assistance usually required; medical intervention/therapy required, hospitalizations possible
GRADE 4	Life-threatening or death*: Extreme limitation in activity, significant assistance required; significant medical intervention/therapy required, hospitalization or hospice care probable

* The draft DMID pediatric toxicity tables characterize death as a Grade 5 event, for the purposes of this study the sponsor will categorize events into 4 grades and has included death with life-threatening in the Grade 4 category.

SERIOUS OR LIFE-THREATENING ADVERSE EVENTS

ANY clinical event deemed by the clinician to be serious or life-threatening should be considered a grade 4 event. Clinical events considered to be serious or life-threatening include, but are not limited to: seizures, coma, tetany, diabetic ketoacidosis, disseminated intravascular coagulation, diffuse petechiae, paralysis, acute psychosis, severe depression.

COMMENTS REGARDING THE USE OF THESE TABLES

- Standardized and commonly used toxicity tables (Division of AIDS, NCI's Common Toxicity Criteria [CTC], and WHO) have been adapted for use by the DMID and modified to better meet the needs of participants in DMID trials.

- For parameters not included in the following Toxicity Tables, sites should refer to the "Guide For Estimating Severity Grade" located above.
- Criteria are generally grouped by body system.
- Some protocols may have additional protocol-specific grading criteria, which will supersede the use of these tables for specified criteria.

DIVISION OF MICROBIOLOGY AND INFECTIOUS DISEASES (DMID) PEDIATRIC TOXICITY TABLES NOVEMBER 2007

(Selected Values for children less than or equal to 3 months of age – does not apply to preterm infants)

For all parameters not listed in this table, please refer to the DMID Toxicity Table for children >3 months of age

**HEMATOLOGY**				
	**Grade 1**	**Grade 2**	**Grade 3**	**Grade 4**
**Hemoglobin**				
1-7 days old	13.0-14.0 g/dL	12.0-12.9 g/dL	<12 g/dL	Cardiac Failure secondary to Anemia
8-21 days old	12.0-13.0 g/dL	10.0-11.9 g/dL	<10.0 g/dL	Cardiac Failure secondary to Anemia
22-35 days old	9.5-10.5 g/dL	8.0-9.4 g/dL	<8.0 g/dL	Cardiac Failure secondary to Anemia
36-60 days old	8.5-9.4 g/dL	7.0-8.4 g/dL	<7.0 g/dL	Cardiac Failure secondary to Anemia
61-90 days old	9.0-9.9 g/dL	7.0-8.9 g/dL	<7.0 g/dL	Cardiac Failure secondary to Anemia
**Absolute Neutrophil Count**				
1 day old	5000-7000/mm^3	3000-4999/mm^3	1500-2999/mm^3	<1500/mm^3
2-6 days old	1750-2500/mm^3	1250-1749/mm^3	750-1249/mm^3	<750/mm^3
7-60 days old	1200-1800/mm^3	900-1199/mm^3	500-899/mm^3	<500/mm^3
61-90 days old	750-1200/mm^3	400-749/mm^3	250-399/mm^3	<250/mm^3

DIVISION OF MICROBIOLOGY AND INFECTIOUS DISEASES (DMID) PEDIATRIC TOXICITY TABLES NOVEMBER 2007

(Selected values for children younger than or aged 3 months)

**HEMATOLOGY (continued)**				
	**Grade 1**	**Grade 2**	**Grade 3**	**Grade 4**
**Bilirubin** (fractionated bilirubin test must be performed when total bilirubin is elevated)				
<7 days old	-	20-25 mg/dL	26-30 mg/dL	>30 mg/dL
7-60 days old	1.1-1.9xN	2.0-2.9xN	3.0-7.5xN	>7.5xN
61-90 days old	1.1-1.9xN	2.0-2.9xN	3.0-7.5xN	>7.5xN
**Creatinine**				
<7 days old	1.0-1.7 mg/dL	1.8-2.4 mg/dL	2.5-3.0 mg/dL	>3.0 mg/dL
7-60 days old	0.5-0.9 mg/dL	1.0-1.4 mg/dL	1.5-2.0 mg/dL	>2.0 mg/dL
61-90 days old	0.6-0.8 mg/dL	0.9-1.1 mg/dL	1.2-1.5 mg/dL	>1.5 mg/dL
**Creatinine Clearance**				
<7 days old	35-40 mL/min	30-34 mL/min	25-29 mL/min	<25 mL/min
7-60 days old	45-50 mL/min	40-44 mL/min	35-39 mL/min	<35 mL/min
61-90 days old	60-75 mL/min	50-59 mL/min	35-49 mL/min	<35 mL/min
**Hypocalcemia**				
<7 days old	6.5-6.9 mEq/L	6.0-6.4 mEq/L	5.5-5.9 mEq/L	<5.5 mEq/L
7-60 days old	7.6-8.0 mEq/L	7.0-7.5 mEq/L	6.0-6.9 mEq/L	<6.0 mEq/L
61-90 days old	7.8-8.4 mEq/L	7.0-7.7 mEq/L	6.0-6.9 mEq/L	<6.0 mEq/L
**Hypercalcemia**				
<7 days old	12.0-12.4 mEq/L	12.5-12.9 mEq/L	13.0-13.5 mEq/L	>13.5 mEq/L
7-60 days old	10.5-11.2 mEq/L	11.3-11.9 mEq/L	12.0-13.0 mEq/L	>13.0 mEq/L
61-90 days old	10.5-11.2 mEq/L	11.3-11.9 mEq/L	12.0-13.0 mEq/L	>13.0 mEq/L

DIVISION OF MICROBIOLOGY AND INFECTIOUS DISEASES (DMID) PEDIATRIC TOXICITY TABLES NOVEMBER 2007

(Older than 3 months of age)

LOCAL REACTIONS				
	**Grade 1**	**Grade 2**	**Grade 3**	**Grade 4**
Induration	<10 mm	10-25 mm	26-50 mm	>50 mm
Erythema	<10 mm	10-25 mm	26-50 mm	>50 mm
Edema	<10 mm	10-25 mm	26-50 mm	>50 mm
Rash at Injection Site	<10 mm	10-25 mm	26-50 mm	>50 mm
Pruritus	Slight itching at injection site	Moderate itching at injection extremity	Itching at injection extremity and other sites	Itching over entire body

HEMATOLOGY				
	**Grade 1**	**Grade 2**	**Grade 3**	**Grade 4**
Hemoglobin for children older than 3 months and younger than 2 years of age	9.0 - 9.9 g/dL	7.0 - 8.9 g/dL	<7.0 g/dL	Cardiac Failure secondary to anemia
Hemoglobin for children older than 2 years of age	10 - 10.9 g/dL	7.0 - 9.9 g/dL	<7.0 g/dL	Cardiac Failure secondary to anemia
Absolute Neutrophil Count	750 - 1200/mm^3	400 - 749/mm^3	250 - 399/mm^3	<250/mm^3
Platelets	-------	50,000 - 75,000/mm^3	25,000 - 49,999/mm^3	<25,000/mm^3
Prothrombin Time (PT)	1.1 - 1.2 x ULN	1.3 - 1.5 x ULN	1.6 - 3.0 x ULN	>3.0 x ULN
Partial Thromboplastin Time (PTT)	1.1 - 1.6 x ULN	1.7 - 2.3 x ULN	2.4 - 3.0 x ULN	>3.0 x ULN

DIVISION OF MICROBIOLOGY AND INFECTIOUS DISEASES (DMID) PEDIATRIC TOXICITY TABLES NOVEMBER 2007

(Older than 3 months of age)

GASTROINTESTINAL				
	**Grade 1**	**Grade 2**	**Grade 3**	**Grade 4**
Bilirubin (when accompanied by any increase in other liver function test)	1.1 - <1.25 x ULN	1.25 - <1.5 x ULN	1.5 - 1.75 x ULN	>1.75 x ULN
Bilirubin (when other liver function are in the normal range)	1.1 - <1.5 x ULN	1.5 - <2.0 x ULN	2.0 - 3.0 x ULN	>3.0 x ULN
AST (SGOT)	1.1 - <2.0 x ULN	2.0 - <3.0 x ULN	3.0 - 8.0 x ULN	>8 x ULN
ALT (SGPT)	1.1 - <2.0 x ULN	2.0 - <3.0 x ULN	3.0 - 8.0 x ULN	>8 x ULN
GGT	1.1 - <2.0 x ULN	2.0 - <3.0 x ULN	3.0 - 8.0 x ULN	>8 x ULN
Pancreatic Amylase	1.1 - 1.4 x ULN	1.5 - 1.9 x ULN	2.0 - 3.0 x ULN	>3.0 x ULN
Uric Acid	7.5 - 9.9 mg/dL	10 - 12.4 mg/dL	12.5 - 15.0 mg/dL	>15.0 mg/dL
CPK	See Neuromuscular Toxicity			
Appetite	-	Decreased appetite	Appetite very decreased, no solid food taken	No solid or liquid taken
Abdominal Pain	Mild	Moderate- No Treatment Needed	Moderate- Treatment Needed	Severe- Hospitalized for treatment
Diarrhea	Slight change in consistency and/or frequency of stools	Liquid stools	Liquid stools greater that 4x the amount or number normal for this child	Liquid stools greater than 8x the amount or number normal for this child

DIVISION OF MICROBIOLOGY AND INFECTIOUS DISEASES (DMID) PEDIATRIC TOXICITY TABLES NOVEMBER 2007

(Older than 3 months of age)

GASTROINTESTINAL (continued)				
	**Grade 1**	**Grade 2**	**Grade 3**	**Grade 4**
Constipation	Slight change in the consistency/frequency of stool	Hard, dry stools with a change in frequency	Abdominal pain	Distention and Vomiting
Nausea	Mild	Moderate-Decreased oral intake	Severe-Little oral intake	Unable to ingest food or fluid for more than 24 hours
Vomiting	1 episode/day	2-3 episodes per day	4-6 episodes per day	Greater than 6 episodes per day or Intractable Vomiting

DIVISION OF MICROBIOLOGY AND INFECTIOUS DISEASES (DMID) PEDIATRIC TOXICITY TABLES NOVEMBER 2007

(Older than 3 months of age)

**ELECTROLYTES**				
	**Grade 1**	**Grade 2**	**Grade 3**	**Grade 4**
**CREATININE**				
3 months - 2 years of age	0.6 - 0.8 x ULN	0.9 - 1.1 x ULN	1.2 - 1.5 x ULN	>1.5 x ULN
2 years - 12 years of age	0.7 - 1.0 x ULN	1.1 - 1.6 x ULN	1.7 - 2.0 x ULN	>2.0 x ULN
Older than 12 years of age	1.0 - 1.7 x ULN	1.8 - 2.4 x ULN	2.5 - 3.5 x ULN	>3.5 x ULN
Hypernatremia	-	<145 - 149 mEq/L	150 - 155 mEq/L	>155 mEq/L or abnormal sodium AND mental status changes
Hyponatremia	-	130 - 135 mEq/L	129 - 124 mEq/L	<124 mEq/L or abnormal sodium AND mental status changes
Hyperkalemia	5.0 - 5.9 mEq/L	6.0 - 6.4 mEq/L	6.5 - 7.0 mEq/L	>7.0 mEq/L or abnormal potassium AND cardiac arrhythmia
Hypokalemia	3.0-3-5 mEq/L	2.5-2.9 mEq/L	2.0-2.4 mEq/L	<2.0 mEq/L or abnormal potassium AND cardiac arrhythmia
Hypercalcemia	10.5 - 11.2 mg/dL	11.3 - 11.9 mg/dL	12.0 - 12.9 mg/dL	>13.0 mg/dL
Hypocalcemia	7.8 - 8.4 mg/dL	7.0 - 7.7 mg/dL	6.0 - 6.9 mg/dL	<6.0 mg/dL
Hypomagnesemia	1.2 - 1.4 mEq/L	0.9 - 1.1 mEq/L	0.6 - 0.8 mEq/L	<0.6 mEq/L or abnormal magnesium AND cardiac arrhythmia
Hypoglycemia	55 - 65 mg/dL	40 - 54 mg/dL	30 - 39 mg/dL	<30 mg/dL or abnormal glucose AND mental status changes
Hyperglycemia	116 - 159 mg/dL	160 - 249 mg/dL	250 - 400 mg/dL	>400 mg/dL or ketoacidosis
Proteinuria	Tr-1+ or <150 mg/day	2+ or 150-499 mg/day	3+ or 500-1000 mg/day	4+ or Nephrotic syndrome >1000 mg/day
Hematuria	Microscopic <25 cells/hpf	Microscopic >25 cells/hpf	----	Gross hematuria

DIVISION OF MICROBIOLOGY AND INFECTIOUS DISEASES (DMID) PEDIATRIC TOXICITY TABLES NOVEMBER 2007

(Older than 3 months of age)

ELECTROLYTES (continued)				
	**Grade 1**	**Grade 2**	**Grade 3**	**Grade 4**
Hypernatremia	-	<145 - 149 mEq/L	150 - 155 mEq/L	>155 mEq/L or abnormal sodium AND mental status changes
Hyponatremia	-	130 - 135 mEq/L	129 - 124 mEq/L	<124 mEq/L or abnormal sodium AND mental status changes
Hyperkalemia	5.0 - 5.9 mEq/L	6.0 - 6.4 mEq/L	6.5 - 7.0 mEq/L	>7.0 mEq/L or abnormal potassium AND cardiac arrhythmia
Hypokalemia	3.0 - 3.5 mEq/L	2.5 - 2.9 mEq/L	2.0 - 2.4 mEq/L	<2.0 mEq/L or abnormal potassium AND cardiac arrhythmia
Hypercalcemia	10.5 - 11.2 mg/dL	11.3 - 11.9 mg/dL	12.0 - 12.9 mg/dL	>13.0 mg/dL
Hypocalcemia	7.8 - 8.4 mg/dL	7.0 - 7.7 mg/dL	6.0 - 6.9 mg/dL	<6.0 mg/dL
Hypomagnesemia	1.2 - 1.4 mEq/L	0.9 - 1.1 mEq/L	0.6 - 0.8 mEq/L	<0.6 mEq/L or abnormal magnesium AND cardiac arrhythmia
Hypoglycemia	55 - 65 mg/dL	40 - 54 mg/dL	30 - 39 mg/dL	<30 mg/dL or abnormal glucose AND mental status changes
Hyperglycemia	116 - 159 mg/dL	160 - 249 mg/dL	250 - 400 mg/dL	>400 mg/dL or ketoacidosis
Proteinuria	Tr-1+ or <150 mg/day	2+ or 150-499 mg/day	3+ or 500-1000 mg/day	4+ or Nephrotic syndrome >1000 mg/day
Hematuria	Microscopic <25 cells/hpf	Microscopic >25 cells/hpf	-	Gross hematuria

DIVISION OF MICROBIOLOGY AND INFECTIOUS DISEASES (DMID) PEDIATRIC TOXICITY TABLES NOVEMBER 2007

(Older than 3 months of age)

CENTRAL NERVOUS SYSTEM (CNS)				
	**Grade 1**	**Grade 2**	**Grade 3**	**Grade 4**
Generalized CNS Symptoms	-	-	Dizziness	Hypotonic, hyporesponsive episodes; Seizures; Apnea/Bradycardia ; Inconsolable crying >3 hrs
Headache	Mild	Moderate, Responds to non-narcotic analgesia	Moderate to Severe, Responds to narcotic analgesia	Intractable
Level of Activity	-	Slightly irritable OR slightly subdued	Very irritable OR Lethargic	Inconsolable OR Obtunded
Visual	-	Blurriness, diplopia, or horizontal nystagmus of <1 hour duration, with spontaneous resolution	More than 1 episode of Grade 2 symptoms per week, or an episode of Grade 2 symptoms lasting more than 1 hour with spontaneous resolution by 4 hours or vertical nystagmus	Decrease in visual acuity, visual field deficit, or oculogyric crisis
Myelopathy	-	None	None	Myelopathic/spinal cord symptoms, such as: pyramidal tract weakness and disinhibition, sensory level, loss of proprioception, bladder/bowel dysfunction

DIVISION OF MICROBIOLOGY AND INFECTIOUS DISEASES (DMID) PEDIATRIC TOXICITY TABLES NOVEMBER 2007

(Older than 3 months of age)

PERIPHERAL NERVOUS SYSTEM				
	**Grade 1**	**Grade 2**	**Grade 3**	**Grade 4**
Neuropathy/ Lower Motor Neuropathy	-	Mild transient Paresthesia only	Persistent or progressive paresthesias, burning sensation in feet, or mild dysesthesia; no weakness; mild to moderate deep tendon reflex changes; no sensory loss	Onset of significant weakness, decrease or loss of DTRs, sensory loss in “stocking glove” distribution, radicular sensory loss, multiple cranial nerve involvement; bladder or bowel dysfunction, fasciculations, respiratory embarrassment from chest wall weakness.
Myopathy or Neuromuscular Junction Impairment	Normal or mild (<2 x ULN) CPK elevation	Mild proximal weakness and/or atrophy not affecting gross motor function. Mild myalgias, +/- mild CPK elevation (<2 x ULN)	Proximal muscle weakness and/or atrophy affecting motor function +/- CPK elevation; or severe myalgias with CPK >2 x ULN;	Onset of myasthenia-like symptoms (fatigable weakness with external, variable ophthalmoplegia and/or ptosis), or neuromuscular junction blockade (acute paralysis) symptoms

DIVISION OF MICROBIOLOGY AND INFECTIOUS DISEASES (DMID) PEDIATRIC TOXICITY TABLES NOVEMBER 2007

(Older than 3 months of age)

**OTHER**				
	**Grade 1**	**Grade 2**	**Grade 3**	**Grade 4**
Allergy	Pruritus without Rash	Pruritic Rash	Mild Urticaria	Severe Urticaria Anaphylaxis, Angioedema
Drug Fever (Rectal)	-	38.5 - 40.0°C 101.3 - 104.0 °F	Greater than 40.0°C Greater than 104.0°F	Sustained Fever: Equal or greater than 40.0°C (104.0°F) for longer than 5 days
Cutaneous	Localized rash	Diffuse maculopapular Rash	Generalized urticaria	Stevens-Johnson Syndrome or Erythema multiforme
Stomatitis	Mild discomfort	Painful, difficulty swallowing, but able to eat and drink	Painful: unable to swallow solids	Painful: unable to swallow liquids; requires IV fluids
Clinical symptoms *not otherwise specified* in this table	No therapy; monitor condition	May require minimal intervention and monitoring	Requires medical care and possible hospitalization	Requires active medical intervention, hospitalization, or hospice care
Laboratory values *not otherwise specified* in this table	Abnormal, but requiring no immediate intervention; follow	Sufficiently abnormal to require evaluation as to causality and perhaps mild therapeutic intervention, but not of sufficient severity to warrant immediate changes in study drug	Sufficiently severe to require evaluation and treatment, including at least temporary suspension of study drug	Life-threatening severity; Requires immediate evaluation, treatment, and usually hospitalization; Study drug must be stopped immediately and should not be restarted until the abnormality is clearly felt to be caused by some other mechanism that study drug

Attachment 2: Cardiovascular Safety – Abnormalities

Electrocardiogram

All important abnormalities from the ECG readings will be listed.

Parameter (unit)	Age class	Abnormally low	Abnormally high
PR (msec)	0 - 2 years	NA	>150
	2 - <3 years	<100	>150
QRS (msec)	0 - 2 years	NA	>79
	2 - <3 years	<40	>79
QT (msec)	0 - 2 years	NA	>500
	2 - <18 years	<320	>450
RR (msec)	0 - 3 months	<333	>750
	3 - 12 months	<400	>860
	1 - 2 years	<430	>1000
	2 - <18 years	<600	>1200

Vital Signs

Normal ranges:

Parameter (unit)	Age class				
	0 - 3 months	3 - 6 months	6 - 12 months	1 - 2 years	2- <3 years
Diastolic BP (mmHg)	45 - 55	50 - 65	55 - 65	55 - 70	45 - 60
Systolic BP (mmHg)	65 -85	70 - 80	80 - 100	90 - 105	85 - 100
Heart rate HR (bpm)	100 - 150	90 - 120	80 - 120	70 - 110	95 - 125
Respiratory rate	35 - 55	30 - 45	25 - 40	20 - 30	22 - 30
Oxygen saturation SpO_2 (%)	≥96	≥96	≥96	≥96	≥96

The following clinically relevant abnormalities will be defined for vital signs

Parameter (unit)		Age class			
		0 - 3 months	3 - 12 months	1 - 2-years	2- <3 years
Diastolic BP (mmHg)	abnormally low	<35	<40	<40	<40
	abnormally high	>65	>85	>90	>70
Systolic BP (mmHg)	abnormally low	<60	<60	<75	<80
	abnormally high	>110	>110	>120	>110
Heart rate HR (bpm)	abnormally low	<80	<70	<60	<90
	abnormally high	>180	>150	>140	>130
Respiratory rate	abnormally low	<25	<20	<18	<20
	abnormally high	>70	>60	>50	>35
Oxygen saturation SpO_2 (%)	abnormally low	<92	<92	<92	<92

References: Normal ranges for vital signs based on the following references and internal pediatrician insight:

Fleming S, Thompson M, Stevens R, et al. Normal ranges of heart rate in children from birth to 18 years: a systematic review of observational studies. Lancet. 2011;377(9770):1011–1018.
Kliegman RM, Stanton B, St Geme J, Schor N, Behrman RE. Nelson Textbook of Pediatrics, 19th Edition.

Attachment 3: Guideline for the Estimation of Glomerular Filtration Rate (GFR) Based on Serum Creatinine in Children

- The production of creatinine is proportional to muscle mass, which is in turn related to body mass and height (for normally proportioned individuals). Therefore in a steady-state situation the GFR can be estimated from the plasma creatinine according to Schwartz formula (for serum creatinine measured by Jaffe methodology):

Children 1-12 years[1]:

$$eGFR = \frac{0.55 \text{ x length (cm)}}{\text{Serum creatinine (mg/dL)}}$$

- The Schwartz formula is modified for different age groups and sexes:

Infants less than 1 year of age[2] (except neonates):

$$eGFR = \frac{0.45 \text{ x length (cm)}}{\text{Serum creatinine (mg/dL)}}$$

- The Schwartz formula has been updated for enzymatic serum creatinine determinations referenced to isotope dilution mass spectroscopy standards[3]:

$$eGFR = \frac{0.41 \text{ x length (cm)}}{\text{Serum creatinine (mg/dL)}}$$

OR: $eGFR = 40.7 \text{ x } (\text{height(m)/Scr (mg/dL)})^{0.640} \text{ x } (30/\text{BUN (mg/dL)})^{0.202}$

References:

1. Schwartz GJ, Haycock GB, Edelmann CM et al. A Simple Estimate of Glomerular Filtration Rate in Children Derived from Body Length and Plasma Creatinine. Pediatrics. 1976; 58: 259-263
2. Schwartz GJ, Feld LG, Langford DJ. A Simple Estimate of Glomerular Filtration Rate in Full-term Infants During the First Year of Life. J. Pediatr. 1984; 104(6):849-853
3. Schwartz GJ, Muñoz A, Schneider MF et al. New Equations to Estimate GFR in Children with CKD. J Am Soc Nephrol. 2009; 20:629-637

Attachment 4: Visit Schedule for Rash Management in Pediatric Subjects

This visit schedule summarizes the visits and assessments to be performed in case of rash. At the investigator's discretion, additional visits and assessments can be performed.

	Grade 1 Rash	Grade 2 Rash	Grade 3 or 4 Rash
**Day 1 of rash**[1]	• Study medication may be continued.<br>• Unscheduled visit for initial rash evaluation **REQUIRED**.<br>• Digital pictures **REQUIRED** (preferably within 24h).<br>• Referral to dermatologist (preferably within 24h) **ONLY IF** rash diagnosis or relationship with study medication is uncertain.	• Study medication **MUST** be permanently **DISCONTINUED**. Rechallenge is **NOT ALLOWED**.<br>• Unscheduled visit for initial rash evaluation **REQUIRED**.<br>• Digital pictures **REQUIRED** (preferably within 24h).<br>• Referral to dermatologist (preferably within 24h) **ONLY IF** rash diagnosis or relationship with study medication is uncertain.	• Study medication **MUST** be permanently **DISCONTINUED**. Rechallenge is **NOT ALLOWED**.<br>• Unscheduled visit for initial rash evaluation **REQUIRED**.<br>• Digital pictures **REQUIRED** (within 24h).<br>• Referral to dermatologist **REQUIRED** (preferably within 24h).<br>• At discretion of the investigator or at request of the dermatologist, assessment of safety blood sample.<br>• Biopsy **ONLY IF** required by dermatologist.
**Day 2**	• Follow-up visit **REQUIRED**.[2]<br>• Digital pictures **REQUIRED**.	• Follow-up visit **REQUIRED**.[2]<br>• Digital pictures **REQUIRED**.	• Follow-up visit **REQUIRED**.<br>• Digital pictures **REQUIRED**.
**Day 3**	No rash follow-up visit required.[2]	No rash follow-up visit required.[2]	• Follow-up visit **REQUIRED**.<br>• Digital pictures **REQUIRED**.<br>• At discretion of the investigator or at request of the dermatologist, assessment of safety blood sample.

[1] Note that Day 1 of the rash is the first day of investigator assessment and not the first day of rash as reported by the subject and that the following days are in relation to this first assessment (not study days)

[2] In case rash progresses from a grade 1 or a grade 2 to a higher grade, start follow-up schedule for grade 2, 3, or 4 rash as appropriate.

	Grade 1 Rash	Grade 2 Rash	Grade 3 or 4 Rash
**Day 4**	No rash follow-up visit required.[2]	No rash follow-up visit required.[2]	• Follow-up visit **REQUIRED**.<br>• Digital pictures **REQUIRED**.<br>• At discretion of the investigator or at request of the dermatologist, assessment of safety blood sample.
**Day 5**	No rash follow-up visit required.	No rash follow-up visit required.[2]	• Follow-up visit **REQUIRED**.<br>• Digital pictures **REQUIRED**.<br>• At discretion of the investigator or at request of the dermatologist, assessment of safety blood sample.
**Day 6**	No rash follow-up visit required.[2]	No rash follow-up visit required.[2]	• Follow-up visit **REQUIRED**.<br>• Digital pictures **REQUIRED**.<br>• At discretion of the investigator or at request of the dermatologist, assessment of safety blood sample.
**Day 7**	No rash follow-up visit required.[2]	No rash follow-up visit required.[2]	No Rash follow-up visit required.
**Day 8**	• Follow-up visit **REQUIRED**.[2]<br>• Digital pictures **REQUIRED**.	• Follow-up visit **REQUIRED**.[2]<br>• Digital pictures **REQUIRED.**	No Rash follow-up visit required.
**Further Visits**	If rash is unresolved after second follow-up visit, further visits (with digital pictures) at the investigator's discretion.[2]	If rash is unresolved after second follow-up visit, further visits (with digital pictures) at the investigator's discretion.[2]	Weekly follow-up visits **REQUIRED** (with digital pictures) until resolution of grade 3-4 rash to grade ≤2 rash (further follow-up visits according to grade 1 or grade 2 rash instructions).

[2] In case rash progresses from a grade 1 or a grade 2 to a higher grade, start follow-up schedule for grade 2, 3, or 4 rash as appropriate.

Attachment 5: Pediatric RSV Electronic Severity and Outcome Rating System (PRESORS)v6 – Clinician's Electronic Clinical Outcome Assessment and Caregiver Diary

Clinician's electronic clinical outcome assessment (eCOA)

[Assessment initiation step – Use eCOA Clinician log in screen but ensure the following are recorded ____________ indicates instructions for programming eCOA ____________ indicates entry in the device]

Site ID ________

Clinician ID ____________

Clinician PW ____________

Enter/confirm patient ID ____________

Confirm date __________

Confirm time ____________

First question (asked at screening only):

1. Why was this subject hospitalized?
   - 1.1 Was the subject hospitalized due to any of these clinical signs? (note all that apply)
     - ☐ Apnoea
     - ☐ Respiratory distress (eg, cyanosis, tachypnea, retractions)
     - ☐ Low oxygen saturation
     - ☐ Tachycardia
     - ☐ None of these
   - 1.2 Was the subject hospitalized due to any of these clinical signs? (note all that apply)
     - ☐ Dehydration/difficulty feeding
     - ☐ Listless or unresponsive
     - ☐ Fever
     - ☐ Need for clinical monitoring
     - ☐ None of these
   - 1.3 Was the subject hospitalized for any other reason?
     - ☐ No
     - ☐ Yes → *(specify pop-up: please specify [256 character free-text])*

[Intro screen for current/recent status assessment]

Please record the following information regarding the subject's status during the past 12 hours.

1. Describe the subject's reported worst activity level during the past 12 hours (select one)
   - ☐ Alert and active/normal sleep
   - ☐ Irritable/restless/agitated
   - ☐ Floppy/lethargic/poor interaction
   - ☐ Only responds to pain/unresponsive

2. Describe how the subject slept during the past 12 hours (select one)
   - ☐ Normal
   - ☐ Occasional restlessness/disturbed
   - ☐ Restless/disturbed much of the time
   - ☐ Comatose

3. Signs of increased work of breathing during the past 12 hours

   3.1. Did the subject show any of these signs (or reports) of increased work of breathing during the past 12 hours? (note all that apply)
   - ☐ Subcostal retractions
   - ☐ Intercostal retractions
   - ☐ Tracheosternal retractions
   - ☐ Tachypnea *[tachypnea needs defined within the system WHO values pop-up?]*
   - ☐ None of the above *[cannot be checked if any other options selected]*

   3.2. Did the subject show any of these signs (or reports) of increased work of breathing during the past 12 hours? (note all that apply)
   - ☐ Nasal flaring
   - ☐ Head bobbing
   - ☐ Grunting
   - ☐ Other signs of increased work of breathing not already reported
   - ☐ None of the above *[cannot be checked if any other options selected]*

4. During the past 12 hours, are there observations/reports of the subject experiencing any of the following? (note all that apply)
   - ☐ Cyanosis
   - ☐ Apnoea
   - ☐ Cough
   - ☐ Nasal secretions
   - ☐ Wheezing
   - ☐ None of the above reported *[cannot be checked if any other options selected]*

5. How did the subject feed in the past 12 hours? (select one)
   - ☐ >75% of normal amount of feeds via usual route
   - ☐ 50% to 75% of normal amounts of feeds via usual route
   - ☐ <50 % of normal feeds or needing NG feeds or IV fluids

Please record the following information regarding the subject's status now.

6. Describe the subject's activity level now (select one)
   - ☐ Alert and active/normal sleep
   - ☐ Irritable/restless/agitated
   - ☐ Floppy/lethargic/poor interaction
   - ☐ Only responds to pain/unresponsive

7. Signs of increased work of breathing now

   7.1. Does the subject show any of these signs of increased work of breathing now? (note all that apply)
   - ☐ Subcostal retractions
   - ☐ Intercostal retractions
   - ☐ Tracheosternal retractions
   - ☐ Tachypnea *[tachypnea needs defined within the system WHO values pop-up?]*
   - ☐ None of the above *[cannot be checked if any other options selected]*

   7.2. Does the subject show any of these signs of increased work of breathing now? (note all that apply)
   - ☐ Nasal flaring
   - ☐ Head bobbing
   - ☐ Grunting
   - ☐ Other signs of increased work of breathing not already reported
   - ☐ None of the above *[cannot be checked if any other options selected]*

8. Does the subject show any signs of cyanosis?
   - ☐ Yes
   - ☐ No

9. Describe the subject's coughing now (select one)
   - ☐ Little or no coughing
   - ☐ Occasional strong cough, sometimes productive
   - ☐ Frequent cough, sometimes causing choking, gagging, or vomiting

10. Describe the subject's nasal secretions now (select one)
   - ☐ None
   - ☐ Minimal, easily cleared with suctioning
   - ☐ Moderate, but could be cleared with suctioning
   - ☐ Extensive, requires frequent suctioning

11. Describe the subject's wheezing now (select one)
   - ☐ No wheezing
   - ☐ Terminal expiratory wheezing or only with stethoscope
   - ☐ Entire expiration or audible during expiration without stethoscope
   - ☐ Inspiration and expiration without stethoscope

12. Does the subject appear to be dehydrated now?
   - ☐ Yes
   - ☐ No

13. Do you have any concerns relating to the subject's overall condition? (select one)
   - ☐ No concerns (condition is stable or improving)
   - ☐ Some concerns (may become unstable/requires close observation)
   - ☐ Extremely concerned (unstable, requires immediate medical review)

14. Overall, how would you rate the subject's current health status? (select one)
   - ☐ Excellent
   - ☐ Good
   - ☐ Fair
   - ☐ Poor

During hospitalization only:

15. Based on the clinical status of the subject, is the subject ready to be discharged?
   - ☐ Yes
   - ☐ No

At the Day 7 visit only (only for studies in which study drug is administered):

16. In general, was the oral dosing of the study medication well tolerated by the subject?
   - ☐ Yes
   - ☐ No

17. Study drug administration

17.1 During study drug administration, were any of the following reported? (note all that apply)

- ☐ Disgusted expressions after tasting medicine
- ☐ Cried after tasting medicine
- ☐ Refused (would not open mouth or turned head away to avoid medicine)
- ☐ None of these were reported [*cannot be checked if other options selected*]

17.2 During study drug administration, were any of the following reported? (note all that apply)

- ☐ Spit out or coughed out medicine
- ☐ Gagged or wretched
- ☐ Vomited (within 2 minutes of swallowing medicine)
- ☐ None of these were reported [*cannot be checked if other options selected*]

At Day 7 and during the end-of-study visits only, clinicians will be asked the following clinician's global rating of change (CGRC) question:

18. With respect to the child's RSV infection, how would you describe the child's health now compared to the baseline assessment?

-5	-4	-3	-2	-1	0	1	2	3	4	5
Very much worse					Unchanged					Very much better

Screen shown after final question for the assessment has been entered.
Thank you for completing this assessment.

Pediatric RSV Electronic Severity & Outcome Rating System (PRESORS) v6

Caregiver diary

[completed at screening and throughout the study until the end of follow-up]

Enter your user ID ________

Enter your passcode________

Caregiver demographic questions 1-7 to be completed the first time the caregiver completes the diary

1. How are you related to the child in this study? (select one)

   ☐ Parent, step parent, or foster parent
   ☐ Grandparent
   ☐ Other family member
   ☐ Nanny or day care provider
   ☐ Neighbor or friend of the parent
   ☐ Other

2. Do you live with the child in this study? ☐ Yes ☐ No

3. There are 168 hours in a week. Counting time even when the child is asleep, how many hours do you usually take care of the child each week? ______ hours/week

4. What year were you born? ______ year born

5. Are you male or female? ☐ Male ☐ Female

6. How many years of education have you completed? ______ years

7. What best describes your employment status? (select one)
   ☐ Full-time paid employment
   ☐ Full-time homemaker
   ☐ Part-time paid employment
   ☐ Retired
   ☐ Disabled, unable to work due to health problems
   ☐ Unemployed, looking for work
   ☐ Other

Caregiver diary entry to be completed by caregiver at each assessment except as noted

PRESORS Caregiver diary

It is now time to complete your caregiver diary.

For each question, please mark the answer or answers that best describe how the child has been [*during the appropriate recall period for the assessment, see table below*]

Assessment timing	Recall language
Screening and baseline (If ≥12 hours post screening)	“over the last 12 hours”
Twice daily post baseline through Day 14	
Upon waking in the morning	“since bedtime last night”
Evening at the child’s usual bedtime	“since the child woke up this morning”
For studies with more than 7 days follow-up post baseline, once daily in the evening assessments from Day 15 through end of follow-up	“since this time yesterday”
At the Day 28 post-treatment follow-up visit	“over the last 24 hours”

1. Overall, how has the child’s health been [*recall period*]?
   - ☐ Excellent
   - ☐ Good
   - ☐ Fair
   - ☐ Poor

2. If the child was awake at all [*recall period*], how active was the child?
   - ☐ As active as usual
   - ☐ A little less active than usual
   - ☐ A lot less active than usual
   - ☐ Floppy or limp, not responding to you as usual

For 12-hour recall phase use Question 3a

3a. How much did the child sleep [*recall period*]? (select one)
   - ☐ A lot less than usual
   - ☐ A little less than usual
   - ☐ About as much as usual
   - ☐ A little more than usual
   - ☐ A lot more than usual

For 24-hour recall phase use Questions 3b and 3c

3b. How much did the child sleep during the day during the past 24 hours? (select one)

- ☐ A lot less than usual
- ☐ A little less than usual
- ☐ About as much as usual
- ☐ A little more than usual
- ☐ A lot more than usual

3c. How much did the child sleep during the night during the past 24 hours? (select one)

- ☐ A lot less than usual
- ☐ A little less than usual
- ☐ About as much as usual
- ☐ A little more than usual
- ☐ A lot more than usual

4. Was the child crying more than usual [*recall period*]? (select one)
   - ☐ Normal, no more crying than usual
   - ☐ Cried more than usual but calmed if held or soothed
   - ☐ Cried a lot, difficult to calm even if held or soothed
   - ☐ Cried a lot, would not stop crying even if held or soothed

5. Do you hear wheezing or whistling sounds in the child's chest when the child breathes? (select one)
   - ☐ No
   - ☐ Yes, but only at the end of when the child breathes out
   - ☐ Yes, throughout breathing out
   - ☐ Yes, throughout breathing in and out

6. There are signs you can see when a child has breathing problems.
   6.1. Did the child show these signs of breathing problems [*recall period*]? (note all that apply)
   - ☐ Breathing through mouth because of difficulties breathing through stuffy or runny nose
   - ☐ Gasping for air and/or long pauses between breaths
   - ☐ Breathing faster than usual
   - ☐ None of these *[option cannot be checked if any of the other options are selected]*

   6.2. Did the child show any of these signs of breathing problems [recall period]? (note all that apply)
   - ☐ Ribs more visible than usual when child breathes in
   - ☐ Skin at the base of the throat sucked in when breathing in
   - ☐ Belly sucked in when breathing in
   - ☐ None of these *[option cannot be checked if any of the other options are selected]*

   6.3. Did the child show any of these signs of breathing problems [*recall period*]? (note all that apply)
   - ☐ Nostrils flared out when breathing in
   - ☐ Head bobbed back and forth when breathing
   - ☐ Made grunting sounds when breathing
   - ☐ None of these *[option cannot be checked if any of the other options are selected]*

7. Did the child's lips, skin or fingernails look pale or blue [*recall period*]? (select one)
   - ☐ No
   - ☐ Yes, but only when the child was crying
   - ☐ Yes, even when the child had not been crying

8. Did the child cough [*recall period*]? (select one)
   - ☐ No coughing
   - ☐ A little coughing
   - ☐ Coughing a lot
   - ☐ Coughing almost all the time

9. Did the child spit up more often than usual [*recall period*]? (select one)
   - ☐ spit up about as often as usual
   - ☐ spit up a little more often than usual
   - ☐ spit up a lot more often than usual

10. Did the child vomit [*recall period*]? (select one)
   - ☐ No [→ *Question 10*]
   - ☐ Yes [→ *Question 9.1*]

10.1. Did the child only vomit when coughing [*recall period*]? (select one)
- ☐ No
- ☐ Yes

11. Did the child eat, drink or nurse as usual [*recall period*]? (select one)
- ☐ Yes, ate, drank or nursed as usual
- ☐ No, a little less than usual
- ☐ No, a lot less than usual
- ☐ No, did not eat, drink or nurse at all

12. Was the child receiving liquids other than medicine through tubes in the nose, mouth or stomach [*recall period*]? (select one)
- ☐ Yes
- ☐ No

13. Did the child urinate as usual (number of wet diapers or trips to the toilet) [*recall period*]? (select one)
- ☐ Yes, as usual
- ☐ No, a little less than usual
- ☐ No, a lot less than usual
- ☐ No, did not wet a diaper or use the toilet

14. Did the child show any of these signs [*recall period*]? (note all that apply)
- ☐ Dry skin or lips
- ☐ Soft spot on top of the head sunk in
- ☐ Sunken eyes
- ☐ Dark yellow urine
- ☐ Little or no urine
- ☐ None of these *[cannot be checked if other options are selected*]

15. Did the child's heart seem to be beating faster than usual?
- ☐ Yes
- ☐ No

Questions 16-17 are to be completed on the day of hospital discharge and during the morning assessment only through follow-up Day 14

16. Please take and record the child's temperature now

Temperature: ______________° Fahrenheit (*or Celsius as used locally*)

17. [*15 second countdown timer*]. Please count how many times the child breathes. Press the button to start the timer. When you hear the first tone, begin counting the child's breaths. **Each time the child breathes in and out counts as 1 breath**. When you hear the second tone, stop counting and record how many breaths you counted.

    Start Timer [*note: 5 second delay after activating the timer before tone indicating begin timing*]

    Do you need to count again?

    ☐Yes [*back to Start Timer*]
    ☐ No [*go to Enter Count*]

    How many breaths did you count? __________ breaths

At every assessment

18. How many hours did you spend with the child [*recall period*]?
    ______________ hours

19. How many hours were you unable to do things you usually do such as go to work, household chores, or care for other family members because the child had RSV [*recall period*]?
    I missed ___________ hours from my usual activities because of the child's RSV

For clinical trials only during AM assessment on Day 7 only

20. In general, how did the child react when he/she was given the medicine? (note all that apply)
    - ☐ Child took medicine easily [*cannot be checked if other options selected]*
    - ☐ Disgusted expressions after tasting medicine
    - ☐ Cried after tasting medicine
    - ☐ Would not open mouth or turned head away to avoid medicine
    - ☐ Spit out or coughed out medicine
    - ☐ Gagged
    - ☐ Vomited (within 2 minutes of swallowing medicine)

For morning on day of hospital discharge, AM Day 7, AM Day 14, and final assessment (end of study or early withdrawal from the study)

21. How is the child's health now compared to when she/he entered this study?
    - ☐ Very much better
    - ☐ Much better
    - ☐ A little better
    - ☐ No change
    - ☐ A little worse
    - ☐ Much worse
    - ☐ Very much worse

22. Has the child's health returned to how it was before the RSV infection?
    - ☐ Yes
    - ☐ No

After each assessment throughout post-discharge follow-up

Please record all medicines the child was given [*recall period*] in the medication log.

At the end of every assessment

If you have any concerns about the child's health, speak with the child's doctor.

Thank you for completing this diary.

End PRESORS caregiver diary assessment

Attachment 6: General Guidelines for Measuring Vital Signs and SpO_2

General Principles

It is anticipated that variability in the measurement of vital signs and SpO_2 is to be expected due to a number of reasons; therefore, general guidelines for measuring vital signs and SpO_2 have been developed to have a more consistent approach across sites and countries related to the methodology for measuring these clinical parameters.

General methodology for obtaining and measuring vital signs and SpO_2

Parameter	General Instructions
Blood Pressure	• The subject should be allowed to play with the device or feel the cuff inflate to gain his or her cooperation<br>• The cuff size should have a bladder width that is approximately 40% of the circumference of the upper arm, measured midway between the olecranon and the acromion. The length of the cuff bladder should encircle 80 to 100% of the circumference of the upper arm midway between the olecranon and the acromion. The bladder width-to-length should be at least 1:2.<br>• The blood pressure should be measured after 5 minutes of rest in a quiet environment. The child should be seated with his/her back and feet in a supported position. In infants, the blood pressure is measured in a supine position. Measurements made in noncooperative, agitated children are often misleading.<br>• Blood pressure measurements should preferentially be assessed with a completely automated device. Manual techniques will be used only if an automated device is not available.<br>• If an automated device is not available, a properly maintained mercury sphygmomanometer is preferred over aneroid and hybrid sphygmomanometers. When using a mercury sphygmomanometer, the mercury column should be deflated at 2 to 3 mm/heart beat, and the first and last audible sounds should be taken as systolic and diastolic pressure. The column should be read to the nearest 2 mm Hg.<br>• When the blood pressure is measured by auscultation, the stethoscope needs to be placed over the brachial artery pulse in the cubital fossa. The blood pressure should be taken with the subject's right arm supported at the level of the heart. The right arm is preferred in repeated measures of blood pressure for consistency. Clothing that covers the arm should be removed prior to the placement of the cuff.
Heart Rate	• While subject is hospitalized, if possible, use the same heart rate measurement methodology for all subjects enrolled at the site.<br>• The heart rate should be measured after 5 minutes of rest in a quiet environment. The child should be seated with his/her back and feet in a supported position. Measurements made in noncooperative, agitated children are often misleading.<br>• Subject can be in either a sitting or supine position.<br>• Heart rate measurements should preferentially be assessed with a completely automated device. Manual techniques will be used only if an automated device is not available.<br>• If manual measurement, auscultation of the heart or pulse (radial, brachial) determination are considered acceptable.<br>• If manual measurement, 30 seconds (minimum) or 1 minute (preferred) count are considered acceptable.

Parameter	General Instructions
Respiratory Rate	• While subject is hospitalized, if possible, use the same respiratory rate measurement methodology for all subjects enrolled at the site.<br>• The respiratory rate should be measured after 5 minutes of rest in a quiet environment.<br>• Subject can be in either a sitting or supine position.<br>• Respiratory rate measurements can be assessed with an automated device or with manual measurement (no preference).<br>• If manual measurement is used, inspection (preferred) or lung auscultation (alternative) are considered acceptable.<br>• If manual measurement, 30 seconds (minimum) or 1 minute (preferred) count are considered acceptable.
Temperature	• While subject is hospitalized, if possible, use the same type of temperature measurement methodology for all subjects enrolled at the site.<br>• Electronic devices (tympanic, oral) are preferred over traditional mercury thermometers (for oral temperature)<br>• Tympanic (preferred) or oral (alternative) temperature measurements are considered acceptable. Axillary temperature should be avoided since it provides the worst estimate of core temperature and it is largely influenced by environmental conditions.<br>• Rectal temperature recordings in infants and young children are also an option. Rectal temperature measurements should be taken with the subject in the prone position with the legs slightly flexed at the hips and knees; the thermometer is directed anteriorly at an angle of approximately 20° to the surface of the examination table.
SpO_2	• While subject is hospitalized, if possible, use the same type of probe for all subjects enrolled at the site.<br>• The SpO_2 should be measured after 5 minutes of rest in a quiet environment.<br>• Subject can be in either a sitting or supine position.<br>• Pulse oximetry measurements using finger, toe, earlobe or frontal sensors are considered acceptable. If using the digits, assess for warmth and capillary refill, since adequate arterial pulse strength is necessary for obtaining accurate SpO2 measurements.<br>• Avoid placing the sensor on sites distal to indwelling arterial catheters, blood pressure cuffs, or venous engorgement (eg, arteriovenous fistulas, blood transfusions).

INVESTIGATOR AGREEMENT

I have read this protocol and agree that it contains all necessary details for carrying out this study. I will conduct the study as outlined herein and will complete the study within the time designated.

I will provide copies of the protocol and all pertinent information to all individuals responsible to me who assist in the conduct of this study. I will discuss this material with them to ensure that they are fully informed regarding the study drug, the conduct of the study, and the obligations of confidentiality.

Coordinating Investigator (where required):

Name (typed or printed): ____________________

Institution and Address: ____________________

Signature: ____________________ Date: __________

(Day Month Year)

Principal (Site) Investigator:

Name (typed or printed): ____________________

Institution and Address: ____________________

Telephone Number: ____________________

Signature: ____________________ Date: __________

(Day Month Year)

Sponsor's Responsible Medical Officer:

Name (typed or printed): [redacted]

Institution:

Signature: electronic signature appended at the end of the protocol Date: __________

(Day Month Year)

Note: if the address or telephone number of the investigator changes during the course of the study, written notification will be provided by the investigator to the sponsor, and a protocol amendment will not be required.

SIGNATURES

**Signed by'**	**Date**	**Justification**
[redacted]	21Aug2017, 16:40:12 PM, UTC	Document Approval
