# Supplementary material for: Lumicitabine, an orally administered nucleoside analog, in infants hospitalized with respiratory syncytial virus (RSV) infection: Safety, efficacy, and pharmacokinetic results
Source: PLoS One. 2023 Jul 19;18(7):e0288271. doi: 10.1371/journal.pone.0288271 (PMC10355467; doi:10.1371/journal.pone.0288271)
Supplement: S4 Appendix — (DOCX) [file pone.0288271.s004.docx]

**S4 Appendix**

**Table 1. Number of Patients with Detection of Respiratory Viruses Other Than RSV in SAD Part (Safety Analysis Set)**

|  | **4.1/1.37 mg/kg (N=5)**  **n (%)** | **10/2 mg/kg (N=14)**  **n (%)** | **30/6 mg/kg (N=8)**  **n (%)** | **30/10 mg/kg (N=17)**  **n (%)** | **40/20 mg/kg (N=18)**  **n (%)** | **60/40 mg/kg (N=16)**  **n (%)** | **Overall (N=78)**  **n (%)** | **Placebo (N=33)**  **n (%)** |
| --- | --- | --- | --- | --- | --- | --- | --- | --- |
| Number of patients without viral coinfection | 4 (80.0) | 10 (71.4) | 7 (87.5) | 7 (41.2) | 15 (83.3) | 14 (87.5) | 57 (73.1) | 23 (69.7) |
| Number of patients with viral coinfection | 1 (20.0) | 4 (28.6) | 1 (12.5) | 10 (58.8) | 3 (16.7) | 2 (12.5) | 21 (26.9) | 10 (30.3) |
| Adenovirus B/E | 0 | 2 (14.3) | 0 | 2 (11.8) | 0 | 0 | 4 (5.1) | 2 (6.1) |
| Adenovirus C | 0 | 1 (7.1) | 1 (12.5) | 1 (5.9) | 0 | 0 | 3 (3.8) | 1 (3.0) |
| Coronavirus 229E | 0 | 0 | 0 | 1 (5.9) | 0 | 0 | 1 (1.3) | 0 |
| Coronavirus HKU1 | 0 | 0 | 0 | 0 | 0 | 0 | 0 | 2 (6.1) |
| Coronavirus NL63 | 0 | 0 | 0 | 1 (5.9) | 0 | 1 (6.3) | 2 (2.6) | 0 |
| Coronavirus OC43 | 0 | 0 | 0 | 0 | 0 | 0 | 0 | 0 |
| Human Metapneumovirus | 0 | 0 | 0 | 2 (11.8) | 0 | 0 | 2 (2.6) | 0 |
| Human Rhinovirus | 1 (20.0) | 2 (14.3) | 1 (12.5) | 6 (35.3) | 2 (11.1) | 1 (6.3) | 13 (16.7) | 7 (21.2) |
| Influenza A | 0 | 0 | 0 | 1 (5.9) | 0 | 0 | 1 (1.3) | 0 |
| Influenza A 2009 H1N1 | 0 | 0 | 0 | 0 | 0 | 0 | 0 | 0 |
| Influenza A H1 | 0 | 0 | 0 | 0 | 0 | 0 | 0 | 0 |
| Influenza A H3 | 0 | 0 | 0 | 1 (5.9) | 0 | 0 | 1 (1.3) | 0 |
| Influenza B | 0 | 0 | 0 | 0 | 0 | 0 | 0 | 0 |
| Parainfluenza Virus 1 | 0 | 1 (7.1) | 0 | 0 | 0 | 0 | 1 (1.3) | 1 (3.0) |
| Parainfluenza Virus 2 | 0 | 0 | 0 | 0 | 0 | 0 | 0 | 0 |
| Parainfluenza Virus 3 | 0 | 0 | 0 | 2 (11.8) | 0 | 0 | 2 (2.6) | 0 |
| Parainfluenza Virus 4 | 0 | 0 | 0 | 0 | 1 (5.6) | 0 | 1 (1.3) | 1 (3.0) |
| FDA, Food and Drug Administration; RSV, respiratory syncytial virus; SAD, single-ascending dose.  Based on the Genmark Respiratory Viral Panel. Note that Coronavirus 229E, Coronavirus HKU1, Coronavirus NL63, Coronavirus OC43, and Parainfluenza Virus 4 have been analyzed using this assay but are not part of the FDA−cleared panel. | | | | | | | | |

**Table 2. Number of Patients with Detection of Respiratory Viruses Other Than RSV in MAD Part (Safety Analysis Set)**

|  | **4.1/1.37 mg/kg (N=5)**  **n (%)** | **10/2 mg/kg (N=14)**  **n (%)** | **30/6 mg/kg (N=8)**  **n (%)** | **30/10 mg/kg (N=17)**  **n (%)** | **40/20 mg/kg (N=18)**  **n (%)** | **60/40 mg/kg (N=16)**  **n (%)** | **Overall (N=78)**  **n (%)** | **Placebo (N=33)**  **n (%)** |
| --- | --- | --- | --- | --- | --- | --- | --- | --- |
| Number of patients without viral coinfection | 4 (80.0) | 10 (71.4) | 7 (87.5) | 7 (41.2) | 15 (83.3) | 14 (87.5) | 57 (73.1) | 23 (69.7) |
| Number of patients with viral coinfection | 1 (20.0) | 4 (28.6) | 1 (12.5) | 10 (58.8) | 3 (16.7) | 2 (12.5) | 21 (26.9) | 10 (30.3) |
| Adenovirus B/E | 0 | 2 (14.3) | 0 | 2 (11.8) | 0 | 0 | 4 (5.1) | 2 (6.1) |
| Adenovirus C | 0 | 1 (7.1) | 1 (12.5) | 1 (5.9) | 0 | 0 | 3 (3.8) | 1 (3.0) |
| Coronavirus 229E | 0 | 0 | 0 | 1 (5.9) | 0 | 0 | 1 (1.3) | 0 |
| Coronavirus HKU1 | 0 | 0 | 0 | 0 | 0 | 0 | 0 | 2 (6.1) |
| Coronavirus NL63 | 0 | 0 | 0 | 1 (5.9) | 0 | 1 (6.3) | 2 (2.6) | 0 |
| Coronavirus OC43 | 0 | 0 | 0 | 0 | 0 | 0 | 0 | 0 |
| Human Metapneumovirus | 0 | 0 | 0 | 2 (11.8) | 0 | 0 | 2 (2.6) | 0 |
| Human Rhinovirus | 1 (20.0) | 2 (14.3) | 1 (12.5) | 6 (35.3) | 2 (11.1) | 1 (6.3) | 13 (16.7) | 7 (21.2) |
| Influenza A | 0 | 0 | 0 | 1 (5.9) | 0 | 0 | 1 (1.3) | 0 |
| Influenza A 2009 H1N1 | 0 | 0 | 0 | 0 | 0 | 0 | 0 | 0 |
| Influenza A H1 | 0 | 0 | 0 | 0 | 0 | 0 | 0 | 0 |
| Influenza A H3 | 0 | 0 | 0 | 1 (5.9) | 0 | 0 | 1 (1.3) | 0 |
| Influenza B | 0 | 0 | 0 | 0 | 0 | 0 | 0 | 0 |
| Parainfluenza Virus 1 | 0 | 1 (7.1) | 0 | 0 | 0 | 0 | 1 (1.3) | 1 (3.0) |
| Parainfluenza Virus 2 | 0 | 0 | 0 | 0 | 0 | 0 | 0 | 0 |
| Parainfluenza Virus 3 | 0 | 0 | 0 | 2 (11.8) | 0 | 0 | 2 (2.6) | 0 |
| Parainfluenza Virus 4 | 0 | 0 | 0 | 0 | 1 (5.6) | 0 | 1 (1.3) | 1 (3.0) |
| FDA, Food and Drug Administration; RSV, respiratory syncytial virus; MAD, multiple-ascending dose.  Based on the Genmark Respiratory Viral Panel. Note that Coronavirus 229E, Coronavirus HKU1, Coronavirus NL63, Coronavirus OC43, and Parainfluenza Virus 4 have been analyzed using this assay but are not part of the FDA-cleared panel. | | | | | | | | |

**Table 3. Summary of Vital Signs – SAD Part (Safety Analysis Set)**

| **Parameter** | **1.37 mg/kg (N=18)**  **n (%)** | **4.1 mg/kg (N=18) n (%)** | **12 mg/kg (N=14)**  **n (%)** | **25 mg/kg (N=3)**  **n (%)** | **Overall (N=53)**  **n (%)** | **Placebo (N=17)**  **n (%)** |
| --- | --- | --- | --- | --- | --- | --- |
| **Systolic blood pressure (mmHg)** | | | | | | |
| Baseline |  |  |  |  |  |  |
| N | 17 | 18 | 13 | 3 | 51 | 16 |
| Mean (SD) | 100.5 (10.17) | 94.4 (9.43) | 96.3 (17.04) | 100.0 (6.56) | 97.2 (11.91) | 87.7 (13.95) |
| Median (range) | 100.0 (88, 123) | 91.0 (82, 118) | 100.0 (48, 114) | 101.0 (93, 106) | 98.0 (48, 123) | 90.0 (52, 108) |
| Endpoint |  |  |  |  |  |  |
| N | 16 | 16 | 13 | 2 | 47 | 15 |
| Mean (SD) | 90.7 (11.31) | 96.9 (18.45) | 100.6 (16.46) | 98.0 (7.07) | 95.9 (15.52) | 96.1 (11.73) |
| Median (range) | 90.0 (66, 111) | 92.0 (73, 144) | 98.0 (77, 139) | 98.0 (93, 103) | 93.0 (66, 144) | 93.0 (74 114) |
| Change from Baseline |  |  |  |  |  |  |
| N | 16 | 16 | 12 | 2 | 46 | 14 |
| Mean (SD) | −9.8 (14.80) | 2.6 (21.07) | 4.9 (31.61) | 1.0 (12.73) | −1.2 (22.60) | 7.1 (13.95) |
| Median (range) | −9.0 (−54, 7) | 1.5 (−25, 54) | −3.0 (−33, 91) | 1.0 (−8, 10) | −3.5 (−54, 91) | 3.0 (−12, 43) |
| **Diastolic blood pressure (mmHg)** | | | | | | |
| Baseline |  |  |  |  |  |  |
| N | 17 | 18 | 13 | 3 | 51 | 16 |
| Mean (SD) | 61.2 (5.62) | 55.1 (11.73) | 56.8 (12.08) | 51.3 (9.07) | 57.4 (10.22) | 53.9 (11.18) |
| Median (range) | 60.0 (52, 73) | 56.0 (33, 76) | 56.0 (28, 76) | 50.0 (43, 61) | 59.0 (28, 76) | 53.5 (36, 78) |
| Endpoint |  |  |  |  |  |  |
| N | 16 | 16 | 13 | 2 | 47 | 15 |
| Mean (SD) | 57.3 (11.60) | 56.3 (12.47) | 56.0 (5.16) | 71.0 (8.49) | 57.2 (10.59) | 58.9 (8.63) |
| Median (range) | 61.0 (31, 76) | 53.0 (41, 88) | 58.0 (48, 63) | 71.0 (65, 77) | 58.0 (31, 88) | 59.0 (43, 70) |
| Change from Baseline |  |  |  |  |  |  |
| N | 16 | 16 | 12 | 2 | 46 | 14 |
| Mean (SD) | −3.7 (12.99) | 2.1 (15.82) | −1.3 (14.28) | 15.5 (0.71) | −0.2 (14.39) | 6.4 (13.21) |
| Median (range) | 0.0 (−36, 11) | 1.5 (−22, 42) | 1.5 (−24, 30) | 15.5 (15, 16) | 0.5 (−36, 42) | 3.5 (−8, 30) |
| **Respiratory rate (breaths/min)** | | | | | | |
| Baseline |  |  |  |  |  |  |
| N | 18 | 18 | 14 | 3 | 53 | 17 |
| Mean (SD) | 46.9 (9.85) | 40.4 (7.14) | 42.8 (7.16) | 38.0 (11.14) | 43.1 (8.67) | 42.2 (12.58) |
| Median (range) | 46.0 (30, 62) | 42.5 (25, 53) | 42.0 (29, 60) | 40.0 (26, 48) | 42.0 (25, 62) | 40.0 (26, 68) |
| Endpoint |  |  |  |  |  |  |
| N | 18 | 17 | 14 | 2 | 51 | 16 |
| Mean (SD) | 37.7 (10.28) | 37.2 (5.95) | 37.5 (7.44) | 34.0 (11.31) | 37.3 (8.04) | 36.8 (9.98) |
| Median (range) | 39.0 (20, 56) | 36.0 (26, 48) | 35.0 (30, 56) | 34.0 (26, 42) | 36.0 (20, 56) | 38.0 (20, 52) |
| Change from Baseline |  |  |  |  |  |  |
| N | 18 | 17 | 14 | 2 | 51 | 16 |
| Mean (SD) | −9.2 (12.50) | −3.0 (9.05) | −5.3 (6.76) | 1.0 (1.41) | −5.7 (10.00) | −5.0 (12.77) |
| Median (range) | −11.0 (−28, 22) | −1.0 (−23, 11) | −7.0 (−14, 8) | 1.0 (0, 2) | −6.0 (−28, 22) | −3.0 (−28, 18) |
| **Hearts rate (beats/min)** | | | | | | |
| Baseline |  |  |  |  |  |  |
| N | 18 | 18 | 14 | 3 | 53 | 17 |
| Mean (SD) | 152.7 (18.04) | 140.9 (17.26) | 143.1 (24.38) | 137.3 (10.07)) | 145.3 (19.66) | 146.8 (19.44) |
| Median (range) | 159.0 (115, 181) | 136.0 (114, 171) | 134.5 (111, 185) | 136.0 (128, 148) | 140.0 (111 185) | 147.0 (110, 180) |
| Endpoint |  |  |  |  |  |  |
| N | 18 | 17 | 14 | 3 | 51 | 16 |
| Mean (SD) | 135.4 (15.43) | 133.1 (17.18)) | 132.4 (17.55) | 140.0 (4.24) | 134.0 (16.09) | 132.6 (19.42) |
| Median (range) | 129.0 (110, 165) | 131.0 (104, 170) | 132.5 (100, 161) | 140.0 (137, 143) | 131.0 (100, 170) | 133.5 (102, 176) |
| Change from Baseline |  |  |  |  |  |  |
| N | 18 | 17 | 14 | 2 | 51 | 16 |
| Mean (SD) | −17.2 (25.76) | −6.1 (26.30) | −10.7 (28.48) | 8.0 (1.41) | −10.7 (26.36) | −14.4 (18.86) |
| Median (range) | −15.5 (−51, 45) | −7.0 (−39, 45) | −5.5 (−50, 36) | 8.0 (7, 9) | −11.0 (−51, 45) | −12.5 (−45, 22) |
| **Weight (kg)** | | | | | | |
| Baseline |  |  |  |  |  |  |
| N | 18 | 18 | 14 | 3 | 53 | 17 |
| Mean (SD) | 6.2 (1.76) | 6.6 (1.87) | 7.1 (1.41)) | 6.6 (1.16) | 6.6 (1.67) | 7.1 (2.01) |
| Median (range) | 5.7 (4, 11) | 6.7 (4, 10) | 7.6 (5, 9) | 6.0 (6, 8) | 6.3 (4, 11) | 6.7 (4, 11) |
| Endpoint |  |  |  |  |  |  |
| N | 18 | 17 | 14 | 2 | 51 | 16 |
| Mean (SD) | 6.2 (1.58) | 6.2 (1.49) | 7.0 (1.38) | 7.0 (1.78) | 6.4 (1.50) | 7.0 (1.99) |
| Median (range) | 6.1 (4, 10) | 6.2 (4, 9) | 7.3 (5, 9) | 7.0 (6, 8) | 6.3 (4, 10) | 6.7 (4, 11) |
| Change from Baseline |  |  |  |  |  |  |
| N | 18 | 17 | 14 | 2 | 51 | 16 |
| Mean (SD) | −0.1 (0.45) | −0.2 (0.45) | −0.1 (0.14) | 0.2 (0.30) | −0.1 (0.38) | −0.1 (0.27) |
| Median (range) | 0.0 (−1, 1) | 0.0 (−2, 0) | −0.1 (−0, 0) | 0.2 (−0, 0) | 0.0 (−2, 1) | −0.1 (−1, 0) |
| **BMI (kg/m^2^)** | | | | | | |
| Baseline |  |  |  |  |  |  |
| N | 17 | 17 | 14 | 3 | 51 | 17 |
| Mean (SD) | 16.7 (2.11) | 16.9 (1.95) | 16.8 (2.05) | 17.6 (0.76) | 16.8 (1.95) | 16.7 (2.33) |
| Median (range) | 15.8 (14, 21) | 16.7 (14, 20) | 17.0 (12, 19) | 17.2 (17, 18) | 16.8 (12, 21) | 16.5 (13, 21) |
| Endpoint |  |  |  |  |  |  |
| N | 17 | 16 | 14 | 2 | 49 | 16 |
| Mean (SD) | 16.5 (1.90) | 16.2 (1.49) | 16.6 (1.96) | 17.6 (0.54) | 16.5 (1.74) | 16.6 (2.45) |
| Median (range) | 15.8 (13, 20) | 16.2 (14, 19) | 16.9 (12, 20) | 17.6 (17, 18) | 16.5 (12, 20) | 16.6 (12, 22) |
| Change from Baseline |  |  |  |  |  |  |
| N | 17 | 16 | 14 | 2 | 49 | 16 |
| Mean (SD) | −0.2 (1.25) | −0.5 (1.06) | −0.2 (0.35) | 0.4 (0.65) | −0.3 (0.98) | −0.1 (0.63) |
| Median (range) | 0.0 (−3, 3) | −0.2 (−3, 1) | −0.2 (−1, 0) | 0.4 (−0, 1) | −0.1 (−3, 3) | −0.2 (−1, 1) |

SAD, single-ascending dose; SD, standard deviation.

**Table 4. Summary of Vital Signs – MAD Part (Safety Analysis Set)**

| **Parameter** | **4.1/1.37 mg/kg**  **(N=5)** | **10/2 mg/kg**  **(N=14)** | **30/6 mg/kg**  **(N=8)** | **30/10 mg/kg**  **(N=17)** | **40/20 mg/kg**  **(N=18)** | **60/40 mg/kg**  **(N=16)** | **Overall**  **(N=78)** | **Placebo**  **(N=33)** |
| --- | --- | --- | --- | --- | --- | --- | --- | --- |
| **Systolic blood pressure (mmHg)** | | | | | | | | |
| Baseline |  |  |  |  |  |  |  |  |
| N | 5 | 14 | 8 | 15 | 17 | 16 | 75 | 33 |
| Mean (SD) | 95.2 (5.63) | 95.6 (17.61) | 91.3 (10.89) | 98.0 (13.50) | 97.4 (9.49) | 94.7 (9.94) | 95.8 (12.06) | 98.2 (13.67) |
| Median (range) | 94.0 (90, 104) | 92.0 (64, 137) | 89.0 (80, 113) | 100.0 (74, 120) | 98.0 (80, 119) | 93.0 (82, 110) | 96.0 (64, 137) | 98.0 (70, 126) |
| Endpoint |  |  |  |  |  |  |  |  |
| N | 4 | 4 | 2 | 9 | 8 | 11 | 38 | 16 |
| Mean (SD) | 101.5 (13.18) | 108.0 (8.76) | 97.5 (20.51) | 95.9 (18.64) | 96.5 (13.46) | 93.9 (7.92) | 97.4 (13.28) | 108.0 (17.19) |
| Median (range) | 99.5 (89, 118) | 109.5 (96, 117) | 97.5 (83, 112) | 93.0 (62, 130) | 93.0 (78, 119) | 92.0 (85, 112) | 93.5 (62, 130) | 106.5 (81, 160) |
| Change from Baseline | |  |  |  |  |  |  |  |
| N | 4 | 4 | 2 | 8 | 8 | 11 | 37 | 16 |
| Mean (SD) | 6.0 (19.20) | 10.3 (20.07) | −5.0 (35.36) | 2.6 (19.61) | −1.8 (15.71) | −2.1 (10.96) | 1.1 (16.57) | 7.9 (20.96) |
| Median (range) | 5.5 (−15, 28) | 11.5 (−10, 28) | −5.0 (−30, 20) | 11.0 (−37, 23) | −8.0 (−18, 19) | 0.0 (−20, 14) | 0.0 (−37, 28) | 1.5 (−21, 57) |
| **Diastolic blood pressure (mmHg)** | | | | | | | | |
| Baseline |  |  |  |  |  |  |  |  |
| N | 5 | 14 | 8 | 15 | 17 | 16 | 75 | 33 |
| Mean (SD) | 66.8 (5.81) | 57.3 (16.44) | 52.5 (5.40) | 57.0 (11.41) | 61.0 (12.03) | 55.8 (9.77) | 57.9 (11.80) | 56.7(10.37) |
| Median (range) | 64.0 (62, 76) | 54.5 (38, 98) | 52.0 (44, 63) | 52.0 (42, 86) | 60.0 (42, 85) | 56.5 (37, 70) | 56.0 (37, 98) | 56.0 (31, 83) |
| Endpoint |  |  |  |  |  |  |  |  |
| N | 4 | 4 | 2 | 9 | 8 | 11 | 38 | 15 |
| Mean (SD) | 61.3 (14.80) | 56.5 (4.65) | 61.5 (28.99) | 52.1 (13.89) | 54.4 (12.26) | 56.9 (8.34) | 55.9 (11.84) | 61.7 (11.24) |
| Median (range) | 61.5 (47, 75) | 56.5 (51, 62) | 61.5 (41, 82) | 51.0 (33, 80) | 52.5 (38, 76) | 54.0 (50, 78) | 54.5 (33, 82) | 61.0 (43, 81) |
| Change from Baseline | |  |  |  |  |  |  |  |
| N | 4 | 4 | 2 | 8 | 8 | 11 | 37 | 15 |
| Mean (SD) | −6.3 (9.81) | −6.0 (17.42) | 2.5 (34.65) | −6.4 (17.15) | −10.8 (17.19) | −0.7 (10.75) | −5.1 (15.17) | 5.1 (16.32) |
| Median (range) | −7.5 (−16, 6) | −5.5 (−23, 10) | 2.5 (−22, 27) | −4.0 (−44, 13) | −10.5 (−35, 11) | −5.0 (−13, 23) | −5.0 (−44, 27) | 2.0 (−15, 33) |
| **Respiratory rate (breaths/min)** | | |  |  |  |  |  |  |
| Baseline |  |  |  |  |  |  |  |  |
| N | 5 | 14 | 7 | 17 | 18 | 16 | 77 | 33 |
| Mean (SD) | 44.2 (8.14) | 47.2 (17.58) | 48.1 (10.42) | 44.7 (9.69) | 46.2 (10.67) | 46.3 (9.60) | 46.1 (11.39) | 44.7 (9.41) |
| Median (range) | 44.0 (33, 52) | 48.5 (25, 99) | 45.0 (36, 65) | 42.0 (30, 62) | 44.0 (26, 64) | 45.0 (28, 68) | 44.0 (25, 99) | 44.0 (25, 68) |
| Endpoint |  |  |  |  |  |  |  |  |
| N | 3 | 4 | 2 | 11 | 10 | 12 | 42 | 20 |
| Mean (SD) | 44.3 (7.23) | 37.5 (8.54) | 44.0 (11.31) | 40.3 (9.45) | 38.0 (15.92) | 36.0 (8.93) | 38.7 (10.80) | 41.8 (9.45) |
| Median (range) | 48.0 (36, 49) | 34.0 (32, 50) | 44.0 (36, 52) | 40.0 (28, 60) | 35.0 (24, 80) | 34.5 (26, 56) | 37.0 (24, 80) | 40.0 (28, 62) |
| Change from Baseline | |  |  |  |  |  |  |  |
| N | 3 | 4 | 2 | 11 | 10 | 12 | 42 | 20 |
| Mean (SD) | 1.3 (4.73) | 2.8 (17.25) | −0.5 (9.19) | −4.3 (14.40) | −8.4 (11.54) | −9.7 (15.82) | −5.5 (13.81) | −3.9 (11.32) |
| Median (range) | 3.0 (−4, 5) | 1.5 (−17, 25) | −0.5 (−7, 6) | −5.0 (−32, 22) | −11.0 (−20, 20) | −10.0 (−40, 15) | −6.5 (−40, 25) | −4.0 (−26, 16) |
| **Hearts rate (beats/min)** | | |  |  |  |  |  |  |
| Baseline |  |  |  |  |  |  |  |  |
| N | 5 | 14 | 8 | 17 | 18 | 16 | 78 | 33 |
| Mean (SD) | 149.2 (19.77) | 139.9 (17.22) | 146.5 (24.40) | 141.4 (19.75) | 141.4 (17.23) | 149.4 (19.74) | 143.8 (19.02) | 145.4 (18.81) |
| Median (range) | 149.0 (120, 174) | 136.5 (119, 166) | 156.0 (104, 170) | 143.0 (105, 170) | 144.0 (95, 161) | 150.0 (119, 189) | 144.5 (95, 189) | 143.0 (108, 182) |
| Endpoint |  |  |  |  |  |  |  |  |
| N | 4 | 5 | 2 | 11 | 11 | 12 | 45 | 20 |
| Mean (SD) | 139.0 (14.85) | 140.6 (27.15) | 118.5 (9.19) | 133.0 (16.38) | 128.3 (13.65) | 120.7 (22.56) | 129.3 (19.16) | 137.9 (14.51) |
| Median (range) | 135.5 (125, 160) | 149.0 (110, 175) | 118.5 (112, 125) | 130.0 (100, 159) | 122.0 (110, 152) | 126.0 (80, 152) | 128.0 (80, 175) | 136.0 (116, 170) |
| Change from Baseline | |  |  |  |  |  |  |  |
| N | 4 | 5 | 2 | 11 | 11 | 12 | 45 | 20 |
| Mean (SD) | −17.5 (22.58) | 7.6 (23.04) | −27.0 (22.63) | −8.5 (23.75) | −13.2 (21.61) | −30.1 (30.53) | −15.2 (26.47) | −11.0 (26.38) |
| Median (range) | −21.5 (−38, 11) | −4.0 (−13, 39) | −27.0 (−43, −11) | −13.0 (−42, 26) | −8.0 (−43, 25) | −32.0 (−100, 14) | −13.0 (−100, 39) | −4.0 (−66, 54) |
| **Weight (kg)** | | |  |  |  |  |  |  |
| Baseline |  |  |  |  |  |  |  |  |
| N | 5 | 14 | 8 | 17 | 18 | 15 | 77 | 33 |
| Mean (SD) | 6.8 (2.13) | 6.3 (2.29) | 5.7 (1.75) | 6.3 (1.65) | 6.8 (1.42) | 6.6 (1.62)) | 6.5 (1.74) | 6.2 (1.59) |
| Median (range) | 7.1 4, 9 | 6.2 (3, 12) | 5.8 (4, 8) | 6.3 (4, 11) | 6.6 (4, 10) | 6.8 (5, 10) | 6.4 (3, 12) | 5.7 (4, 10) |
| Endpoint |  |  |  |  |  |  |  |  |
| N | 5 | 14 | 8 | 16 | 17 | 16 | 76 | 31 |
| Mean (SD) | 7.4 (2.19) | 6.8 (1.92) | 6.5 (1.63) | 7.0 (1.55) | 7.5 (1.38) | 7.4 (1.48) | 7.1 (1.60) | 6.9 (1.42) |
| Median (range) | 7.2 (4, 10) | 6.6 (4, 12) | 6.4 (5, 9) | 6.6 (5, 11) | 7.3 (5, 10) | 7.3 (5, 10) | 7.1 (4, 12) | 6.7 (5, 10) |
| Change from Baseline |  |  |  |  |  |  |  |  |
| N | 5 | 14 | 8 | 16 | 16 | 15 | 74 | 31 |
| Mean (SD) | 0.6 (0.32) | 0.5 (0.44) | 0.8 (0.31) | 0.6 (0.44) | 0.7 (0.34) | 0.7 (0.40) | 0.6 (0.38) | 0.6 (0.40) |
| Median (range) | 0.6 (0, 1) | 0.6 (−0, 1) | 0.7 (0, 1) | 0.6 (−0, 1) | 0.7 (0, 1) | 0.9 (0, 1) | 0.6 (−0, 1) | 0.7 (−0, 2) |
| **BMI (kg/m^2^)** | | |  |  |  |  |  |  |
| Baseline |  |  |  |  |  |  |  |  |
| N | 5 | 13 | 8 | 17 | 18 | 14 | 75 | 33 |
| Mean (SD) | 16.0 (1.59) | 16.6 (2.13) | 16.4 (3.03) | 16.7 (1.97) | 17.3 (2.04) | 16.8 (2.24) | 16.8 (2.13) | 17.0 (5.02) |
| Median (range) | 17.1 (14, 17) | 16.6 (12, 21) | 15.8 (13, 22) | 16.0 (15, 21) | 17.0 (14, 21) | 16.4 (13, 20) | 16.6 (12, 22) | 16.0 (13, 43) |
| Endpoint |  |  |  |  |  |  |  |  |
| N | 5 | 13 | 8 | 16 | 17 | 15 | 74 | 31 |
| Mean (SD) | 17.6 (1.22) | 18.2 (1.26) | 18.8 (2.71) | 18.5 (1.42) | 19.0 (2.24) | 18.8 (2.32) | 18.6 (1.93) | 19.2 (5.75) |
| Median (range) | 17.5 (16, 19) | 18.1 (16, 21) | 18.4 (16, 24) | 18.8 (16, 20) | 19.2 (14, 23) | 18.7 (16, 23) | 18.7 (14, 24) | 18.0 (14, 48) |
| Change from Baseline |  |  |  |  |  |  |  |  |
| N | 5 | 3 | 8 | 16 | 16 | 14 | 72 | 31 |
| Mean (SD) | 1.5 (0.94) | 1.6 (1.54) | 2.4 (1.27) | 1.6 (1.35) | 1.7 (1.00) | 1.9 (1.22) | 1.8 (1.24) | 2.0 (1.44) |
| Median (range) | 1.5 (0, 3) | 1.4 (−1, 4) | 2.1 (1, 4) | 1.3 (−1, 4) | 1.7 (0, 4) | 2.0 (0, 3) | 1.6 (−1, 4) | 1.8 (−0, 5) |

MAD, multiple-ascending dose; SD, standard deviation.

**Table 5. Need for Oxygen Support – SAD Part (Safety Analysis Set)**

|  | **Lumicitabine** | | | | **Placebo** |
| --- | --- | --- | --- | --- | --- |
|  | **1.37 mg/kg** | **4.1 mg/kg** | **12 mg/kg** | **25 mg/kg** |  |
| **Duration of Oxygen Support (hours)** | | | | | |
| N | 18 | 18 | 14 | 3 | 17 |
| Mean (SD) | 41.9 (31.25) | 35.4 (41.95) | 49.0 (55.28) | 17.6 (15.63) | 30.4 (63.85) |
| Median (range) | 44.8 (0, 103) | 22.5 (0, 133) | 25.2 (0, 165) | 23.2 (0, 30) | 9.5 (0, 266) |
| Censored, n (%) | 0 (0.0) | 1 (5.6) | 0 (0.0) | 0 (0.0) | 0 (0.0) |
| **Oxygen Support Used** | | | | | |
| No, n (%) | 3 (16.7) | 5 (27.8) | 2 (14.3) | 1 (33.3) | 6 (35.3) |
| Yes, n (%) | 15 (83.3) | 13 (72.2) | 12 (85.7) | 2 (66.7) | 11 (64.7) |
| Lumicitabine vs Placebo, OR (95%CI) | 2.62 (0.52, 13.26) | 1.33 (0.30, 5.77) | 3.34 (0.54, 20.81) | 0.90 (0.06, 13.03) |  |

OR, Odds ratio; SAD, single-ascending dose; SD, standard deviation.

**Table 6. Need for Oxygen Support – MAD Part (Safety Analysis Set)**

|  | **Lumicitabine** | | | | | | **Placebo** |
| --- | --- | --- | --- | --- | --- | --- | --- |
|  | **4.1/1.37 mg/kg** | **10/2 mg/kg** | **30/6 mg/kg** | **30/10 mg/kg** | **40/20 mg/kg** | **60/40 mg/kg** |  |
| **Duration of Oxygen Support (hours)** | | | | | | | |
| N | 5 | 14 | 8 | 17 | 18 | 16 | 33 |
| Mean (SD) | 17.5 (27.15) | 48.9 41.19) | 53.3 (39.66) | 49.0 (64.31) | 39.8 (66.78) | 20.8 (26.85) | 44.9 (44.86) |
| Median (range) | 9.3 (0, 65) | 48.4 (0, 134) | 42.5 (0, 130) | 1.2 (0, 205) | 14.3 (0, 285) | 11.27 (0, 82.5) | 33.60 (0, 131.7) |
| Censored, n (%) | 0 (0.0) | 0 (0.0) | 0 (0.0) | 0 (0.0) | 0 (0.0) | 0 (0.0) | 0 (0.0) |
| **Oxygen Support Used** | | | | | | | |
| No, n (%) | 2 (40.0) | 3 (21.4) | 1 (12.5) | 8 (47.1) | 6 (33.3) | 7 (43.8) | 9 (27.3) |
| Yes, n (%) | 3 (60.0) | 11 (78.6) | 7 (87.5) | 9 (52.9) | 12 (66.7) | 9 (56.2) | 24 (72.7) |
| Lumicitabine vs Placebo, OR (95%CI) | 0.56 (0.08, 3.94) | 1.37 (0.31, 6.09) | 2.62 (0.28, 24.42) | 0.42 (0.2, 1.43) | 0.75 (0.22, 2.60) | 0.48 (0.14, 1.68) |  |

MAD, multiple-ascending dose; OR, Odds ratio; SD, standard deviation.
