# Supplementary material for: Lumicitabine, an orally administered nucleoside analog, in infants hospitalized with respiratory syncytial virus (RSV) infection: Safety, efficacy, and pharmacokinetic results
Source: PLoS One. 2023 Jul 19;18(7):e0288271. doi: 10.1371/journal.pone.0288271 (PMC10355467; doi:10.1371/journal.pone.0288271)
Supplement: S5 Appendix — (DOCX) [file pone.0288271.s005.docx]

S5 Appendix

Independent Ethics Committees or Institutional Review Boards

**Study 1 (NCT02202356)**

| **Study** | **Investigator(s)** | **Name, Address, Chair (if applicable) of IRB/IEC** |
| --- | --- | --- |
| MAD | Hsin Chi | Mackay Memorial Hospital Institutional Review Board  No. 92 Section 2, Shongshan North Road Zhongshan District  Taipei 10449 Taiwan  Chair: |
| MAD | Cheng-Hsun Chiu | Chang Gung Medical Foundation Institutional Review Board  B2F., No. 123, Dinghu Road, Guishan District Taoyuan, 33305  Taiwan  Chair: |
| MAD | Christopher Cannavino, MD | University of California San Diego Human Research Protections Program  9500 Gilman Drive  Mail Code 0052  La Jolla, California 92093-0052 United States  Chair: |
| MAD | John DeVincenzo, MD | UTHSC Institutional Review Board 910 Madison  Suite 600  Memphis, Tennessee 38163 United States  Chair: Terrence Ackerman, PhD |
| MAD | Coleen K. Cunningham, MD | Duke University Health System Institutional Review Board Office  2424 Erwin Road; Hock Plaza, Suite 405 Campus Box 2712  Durham, North Carolina 27705 United States  Chairs: Donna Marie Cookmeyer, PhD Jody Power, MS, MBA  John M. Kessler, PharmD  Marilyn Hockenberry, PhD, PNP-BC, F Mark P. Donahue, MD  Sharon L. Ellison, PharmD |
| MAD | Kwabena Krow Ampofo, M.B.Ch.B. | University of Utah Institutional Review Board Research Administration Building 512  75 South 2000 East  Salt Lake City, Utah 84112 United States  Chair: Gerald Treiman |
| MAD | Barbara Pahud, MD, MPH | Children's Mercy Hospital Institutional Review Board  2405 Grand -14th Floor Kansas City, Missouri 64108 United States  Chair: |
| MAD | David Kimberlin | UAB Institutional Review Board for Human Use Room 470 Administration Building  701 20th Street South  Birmingham, Alabama 35294-0104 United States  Chair:  Western Institutional Review Board 1019 39th Avenue South East Suite #120 Puyallup, Washington 98374-2115 United States  Chair: |
| MAD | Flor Munoz-Rivas, MD | Western Institutional Review Board 1019 39th Avenue South East  Suite 120  Puyallup, Washington 98375-2115 United States  Chair:  Institutional Review Board for Human Subject Research for Baylor College of Medicine and Affiliated Hospitals  Office of Research  One Baylor Plaza, Room 600 D Houston, Texas 77030  United States  Chair: |
| MAD | John A. Vanchiere, MD, PhD | LSUHSC - Shreveport Institutional Review Board 1501 Kings Highway  Shreveport, Louisiana 71103 United States  Chair: |
| MAD | Audra Deveikis, MD | Western Institutional Review Board 1019 39th Avenue South East Suite #120 Puyallup, Washington 98374-2115 United States  Chair: |
| MAD | Yoshiyuki Yamada, MD | Clinical Trial Network for pediatrics 2-10-1 Okura  Setagaya-ku, Tokyo 157-8535 Japan  Chair: |
| MAD | Yuho Horikoshi, MD | Clinical Trial Network for pediatrics 2-10-1 Okura  Setagaya-ku, Tokyo 157-8535 Japan  Chair: |
| MAD | Takafumi Okada, MD | Pediatric Clinical Trials Network Institutional Review Board  2-10-1 Okura  Setagaya-ku, Tokyo 157-8535 Japan  Chair: |
| MAD | Kenji Furuno, MD, Ph-D | Clinical Trial Network for pediatrics 2-10-1 Okura  Setagaya-ku, Tokyo 157-8535 Japan  Chair: |
| MAD | Tooru Araki, MD | Pediatric Clinical Trials Network Central Institutional Review Board  2-10-1 Okura  Setagaya-ku, Tokyo 157-8535 Japan  Chair: |
| MAD | Tadashi Hoshino | Japanese Pediatric Clinical Trial network 2-10-1 Okura  Setagaya-ku, Tokyo 157-8535 Japan  Chair: |
| MAD | Kazuhiko Sugimoto, MD | Clinical Trial Network for pediatrics 2-10-1 Okura  Setagaya-ku, Tokyo 157-8535 Japan  Chair: |
| MAD | Kazuhide Ohta, MD, Ph-D | National Hospital Organization Kanazawa Medical Center Institutional Review Board  1-1 Shimoishibiki-machi  Kanazawa, Ishikawa 920-8650 Japan  Chair: |
| MAD | Hiroshi Odajima, MD | National Hospital Organization Fukuoka Hospital 4-39-1 Yakatabaru, Minami-ku  Fukuoka-shi, Fukuoka 811-1394 Japan  Chair: |
| MAD | Ritsuo Nishiuchi, MD | Kochi Health Sciences Center IRB 2125-1 Ike, Okura  Kochi-city, Kochi 781-8555 Japan  Chair: |
| MAD | Isamu Kamimaki, MD | National Hospital Organization Saitama National Hospital Institutional Review Board  2-1 Suwa  Wakho-shi, Saitama 351-0102 Japan  Chair: |
| MAD and SAD | Dr. Jolanta Bernatoniene | NRES Committee West Midlands - Edgbaston The Old Chapel  Royal Standard Place  Nottingham, Nottinghamshire NGI 6FS United Kingdom  Chair: Paul Hamilton |
| MAD and SAD | Professor Saul Faust | NRES Committee West Midlands - Edgbaston The Old Chapel  Royal Standard Place  Nottingham, Nottinghamshire NGI 6FS United Kingdom  Chair: Paul Hamilton |
| SAD | Dr. Steven Cunningham | NRES Committee West Midlands - Edgbaston The Old Chapel  Royal Standard Place  Nottingham, Nottinghamshire NGI 6FS United Kingdom  Chair: Paul Hamilton |
| SAD | Matthew Snape | NRES Committee West Midlands - Edgbaston The Old Chapel  Royal Standard Place  Nottingham, Nottinghamshire NGI 6FS United Kingdom  Chair: Paul Hamilton |
| MAD and SAD | Dr. Shaila Sukthankar | NRES Committee West Midlands - Edgbaston The Old Chapel  Royal Standard Place  Nottingham, Nottinghamshire NGI 6FS United Kingdom  Chair: Paul Hamilton |
| SAD | Paul Seddon | NRES Committee West Midlands - Edgbaston The Old Chapel  Royal Standard Place  Nottingham, Nottinghamshire NGI 6FS United Kingdom  Chair: Paul Hamilton |
| SAD | Professor Harish Vyas | NRES Committee West Midlands - Edgbaston The Old Chapel  Royal Standard Place  Nottingham, Nottinghamshire NGI 6FS United Kingdom  Chair: Paul Hamilton |
| MAD and SAD | Maria Luz Endeiza, MD | Comite de Evaluacion Etico Cientifico del Servicio de Salud Metropolitano Sur Oriente  Av. Concha y Toro 3459 - Paradero 30, Vic. Mackenna  Puente Alto  Santiago, Region Metropolitana 8880465 Chile  Chair: Dr. Patricio Michaud Ch., SSMSO |
| SAD | Tamara Viviani S., MD | Comite de Evaluacion Etico Cientifico del Servicio de Salud Metropolitano Sur Oriente  Av. Concha y Toro 3459 - Paradero 30, Vic. Mackenna  Puente Alto  Santiago, Region Metropolitana 8880465 Chile  Chair: Dr. Patricio Michaud, Ch., SSMSO |
| SAD | Jaime Aurelio Cespedes Londono | Comite de etica en Investigacion Clinica  Foundacion Cardio - Infantil Instituto de Cardiologia  Calle 163A No. 13B - 64  Bogota, Cundinamarca 1101131 Columbia  Chair: J. Sinay Arevalo, MD |
| MAD | Monica Rosa Trujillo Honeysberg | Comite de Investigaciones y Etica en Investigaciones Hospital Pablo Tobon Uribe  Calle 78 B #69-240  Medellin, Antioquia 050034 Columbia  Chair: Jaime Alberto Lopez Monsalve |
| MAD and SAD | Xavier Juan Saez Llorens, MD | Comite de Bioetica en Investigacion del Hospital del Nino  Avenida Balboa, Calle 34 Panama  Chair: |
| MAD and SAD | Kathia Linett Luciani Chiu, MD | Comite Institucional de Etica de la Investigacion de la Caja de Seguro Social  Policlinica Manuel Ferrer Valdes 3er piso, No. 386  Ciudad de Panama, Panama Panama  Chair: Marna Caicedo |
| MAD | Carlos Alberto Daza Timana | Comite de Bioetica en Investigacion Hospital del Nino Dr. Jose Renan Esquivel  Ave. Balboa, calle 34, Panama Panama  Chair: Dr. Luis Coronado |
| MAD and SAD | Assistant Professor Rattapon Uppala | The Khon Kaen University Ethics Committee in Human Research  123 Mitraphap Road  Muang, Khonkaen 40002 Thailand  Chair: Professor Polasak Jeeravipoolvarn, MD |
| SAD | Colonel Veerachai Watanaveeradej | The Institutional Review Board  Royal Thai Army, Medical Department 317/5 Rajavithi Road  Rajathevee, Bangkok 10400 Thailand  Chair: Col. Yawana Tanapat, MD |
| MAD and SAD | Professor Kulkanya Chokephaibulkit | Siriraj Institutional Review Board 2 Wangland Road  Bangkoknoi, Bangkok 10700 Thailand  Chair: Jarupim Soongswang, MD |
| MAD and SAD | Associate Professor Thanyawee Puthanakit | Institutional Review Board, Faculty of Medicine, Chulalongkorn University  1873 Rama IV Road Pathumwan, Bangkok 10330 Thailand  Chair: Tada Sueblinvong, MD |
| MAD and SAD | Dr. Thorsten Villiers Stanley | Health and Disability Ethics Committee Ministry of Health  No 1 The Terrace PO Box 5013  Wellington New Zealand  Chair: |
| SAD | Dr. Tony Walls | Health and Disability Ethics Committee Ministry of Health Ethics Department 20 Aitken Street  Thorndon, Wellington 6011 New Zealand  Chair: |
| MAD | Assistant Professor Peter Craig Richmond | Princess Margaret Hospital for Children Ethics Committee  Roberts Road Subiaco, WA 6008 Australia  Chair: |
| SAD | Assistant Professor Graham Reynolds | ACT Health Human Research Ethics Committee ACT Health Research Office  Level 6 Building 10 Canberra Hospital Australia  Chair: Professor John Biggs |
|  | Professor Brigitte Fauroux | CPP IDF2 - Hopital Necker 149 rue de Sevres  Porte N2 - 1 er etage Paris Cedex 15, 75743` France  Chair: |
| MAD and SAD | John Michael Fayon | PP Ile de France II 149 rue de Sevres Paris Cedex 15, 75743 France  Chair: |

**Study 2 (NCT03333317)**

| **Site** | **IRB/EC Name/Address** |
| --- | --- |
| Philippe Lepage  Huderf  Avenue J. J. Crocq 15 Bruxelles, 1020 Belgium | Central IRB/IEC - Institution Details: Comité voor Medische Ethiek UZA Wilrijkstraat 10  Edegem 2650 Belgium |
| Jeffrey Pernica  McMaster Children's Hospital 1280 Main Street  West Suite 3A-30 Hamilton, ON, L85 4K1 Canada | Local IRB/IEC - Institution Details: Hamilton Integrated Research Ethics Board (HIREB)  293 Wellington Street, Suite 102  Hamilton Integrated Research Ethics Board (HIREB)  Hamilton, ON, L8L 8E7 Canada |
| Jacques Brouard  Pédiatrie Médicale CHU de Caenavenue Côte de Nacre  CAEN, 14000  France | Central IRB/IEC - Institution Details:  CPP Est-II CHRU  Hôpital Saint Jacques 2 place Saint Jacques  25030 Besançon Cedex France |
| Michael Fayon  Pneumologie pédiatrique - Hôpital Pellegrin- Enfants  Pl Amelie Raba Leon Bordeaux Cedex, 33076 France | Central IRB/IEC - Institution Details:  CPP Est-II CHRU  Hôpital Saint Jacques 2 place Saint Jacques  25030 Besançon Cedex France |
| Vincent Gajdos  HOPITAL ANTOINE BECLERE  157, rue porte Trivaux CLAMART Cedex, 92141  France | Central IRB/IEC - Institution Details:  CPP Est-II CHRU  Hôpital Saint Jacques 2 place Saint Jacques  25030 Besançon Cedex France |
| Christophe Marguet  CHU de Rouen - Hôpital Charles Nicolle 1 rue de germont  Rouen, Cedex, 76031 France | Central IRB/IEC - Institution Details:  CPP Est-II CHRU  Hôpital Saint Jacques 2 place Saint Jacques  25030 Besançon Cedex France |
| Wolfgang Kamin  Ev. Krankenhaus Hamm gGmbH Werler Str. 110 Kinderheilkunde Hamm, 59063  Germany | Local IRB/IEC - Institution Details: Ethik-Kommission der Ärztekammer Westfalen-Lippe und der Medizinischen Fakultät der Westfälischen Wilhelms- Universität Münster  Gartenstr. 210-214  Münster, Nordrhein-Westfalen Germany |
| Johannes Hubner  Dr. von Haunersches Kinderspital Lindwurmstr. 4 Eingang F/G München, 80337  Germany | Local IRB/IEC - Institution Details: Ethikkommission der Medizinischen Fakultät der Ludwig-Maximilians-Universität München  Pettenkoferstr. 8a Germany |
| Kyoko Watanabe  National Hospital Organization Kokura Medical Center  10-1, Harugaoka, Kokuraminami-ku,  Kitakyushu, Fukuoka, 802-8533 Japan | Local IRB/IEC - Institution Details: National Hospital Organization Kokura Medical Center IRB  10-1 Harugaoka Kokuraminami-ku Kitakyuushuu-shi  Fukuoka, 802-0803 Japan |
| Yoshiyuki Yamada  Gunma Children’s Medical Center 779 Shimohakoda Hokkitsumachi  Shibukawa, Gunma, 377-8577 Japan | Local IRB/IEC - Institution Details: Review Board of Human Rights and Ethics for Clinical Studies Institutional Review Board  13-2  Ichibancho Chiyoda-ku Tokyo, 102-0082  Japan |
| Hiroshi Odajima  National Hospital Organization Fukuoka Hospital 4-39-1 Yakatabaru Minami-ku  Fukuoka-shi, Fukuoka, 811-1394 Japan | Local IRB/IEC - Institution Details: National Hospital Organization Fukuoka Hospital IRB  4-39-1 Yakatabaru Minami-ku Fukuoka-shi Fukuoka, 811-1394 Japan |
| Isamu Kamimaki  NHO Saitama National Hospital 2-1 Suwa, Wako-shi  Saitama, 351-0102 Japan | Local IRB/IEC - Institution Details: National Hospital Organization Saitama National Hospital IRB  2-1 Suwa Wakho-shi  Saitama, 351-0102 Japan |
| Kazuhiko Sugimoto  Hirosaki National Hospital 1 Tomino-cho, Oaza  Hirosaki, Aomori, 036-8545 Japan | Local IRB/IEC - Institution Details:  Hirosaki National Hospital IRB 1 Tomino-cho  Oaza Hirosaki-shi  Aomori, 036-8545 Japan |
| Takafumi Okada  Shikoku Medical Center for Children and Adults 2-1-1, Senyu-cho  Zentsuji, Kagawa, 765-8507 Japan | Local IRB/IEC - Institution Details: National Hospital Organization Shikoku Medical Center for Children and Adults IRB 2-1-1 Senyu-cho  Zentsuji-shi Kagawa, 765-8507 Japan |
| Kenji Furuno  Fukuoka Children’s Hospital  5-1-1, Kashiiteriha, Higashi-ku  Fukuoka, Fukuoka, 813-0017 Japan | Local IRB/IEC - Institution Details:  Fukuoka Children's Hospital IRB 5-1-1 Kashiiteriha  Higashi-ku Fukuoka-shi Fukuoka, 813-0017 Japan |
| Naoaki Hori  Ota Memorial Hospital 455-1, Oshimacho  Ota, Gunma, 373-8585 Japan | Local IRB/IEC - Institution Details: SUBARU Health Insurance Society Ota Memorial Hospital Institutional Review Board  455-1  Oshimacho Ota, 373-8585  Japan |
| Tetsuro Tsuji  JA Hiroshima General Hospital 1-3-3, jigozen  Hatsukaichi, Hiroshima, 738-8503 Japan | Local IRB/IEC - Institution Details: Koseiren Hospital Central Institutional Review Board  2-5-5 Yoyogi Shibuya-ku Tokyo, 151-0053  Japan |
| Kiyotaka Murakami Nakano Children’s Hospital 4-13-17 Shinmori Asashi-ku  Osaka, Osaka, 535-0022 Japan | Local IRB/IEC - Institution Details: Medical Corporation Shintokai Yokohama Minoru Clinic Institutional Review Board 1-13-8  Bessyo  Minami-ku, Yokohama-City Kanagawa, 232-0064 Japan |
| Kazuhide Ohta  National Hospital Organization Kanazawa Medical Center  1-1, Shimo-ishibiki  Kanazawa, Ishikawa, 920-8650 Japan | Local IRB/IEC - Institution Details: National Hospital Organization Kanazawa Medical Center IRB  1-1 Shimoishibiki-machi Kanazawa  Ishikawa, 920-8650 Japan |
| Toshiaki Suzuki  National Hospital Organization Niigata National Hospital  3-52 Akasaka-cho  Niigata, Kashiwazaki, 945-8585 Japan | Local IRB/IEC - Institution Details:  Niigata National Hospital, National Hospital Organization IRB  3-52 Akasaka-cho Kashiwazaki city  Niigata prefecture, 945-8585 Japan |
| Tooru Araki  National Hospital Organization Fukuyama Medical Center  4-14-17, Okinogami-cho  Fukuyama, Hiroshima, 720-8520 Japan | Local IRB/IEC - Institution Details: National Hospital Organization Fukuyama Medical Center IRB  4-14-17  Okinogami-cho Fukuyama Hiroshima, 720-8520 Japan |
| Masanori Ikeda  Fukuyama City Hospital  5-23-1 Zao-cho Pediatrics  Fukuyama, Hiroshima, 721-8511 Japan | Local IRB/IEC - Institution Details:  Fukuyama City Hospital IRB 5-23-1 Zao-cho  Fukuyama city Hiroshima, 721-8511 Japan |
| Hiroshi Koga  NHO Beppu Medical Center  1473, Ooazanaikamado, Beppu-shi  Oita, 874-0011  Japan | Local IRB/IEC - Institution Details: National Hospital Organaization Beppu Medical Center IRB  1473  Ooazauchikamado Beppu-shi  Oita, 874-0011  Japan |
| Yuya Hashimura  Takatsuki General Hospital  1-3-13, Kosobecho Pediatrics  Osaka, 569-1192  Japan | Local IRB/IEC - Institution Details: Takatsuki General Hospital Institutional Review Board  1-3-13  Kosobe-cho Takatsuki  Osaka, 569-1192  Japan |
| Branko Takac  DFNsP Banska Bystrica Nám. L. Svobodu č. 4 Banska Bystrica, 97401 Slovakia | Central IRB/IEC - Institution Details:  Eticka komisia UNM Univerzitna nemocnica Martin  Kolllarova 2 Martin 03659  Slovakia |
| Ivona Bacmanakova  Klinika detí a dorastu, UNM Martin Kollarova, 2  Martin, Žilinský kraj, 03659 Slovakia | Central IRB/IEC - Institution Details:  Eticka komisia UNM Univerzitna nemocnica Martin  Kolllarova 2 Martin 03659  Slovakia |
| Branko Takac  DFNsP Banska Bystrica Nám. L. Svobodu č. 4 Banska Bystrica, 97401 Slovakia | Local IRB/IEC - Institution Details:  Etická komisia DFNsP BB  Detská fakultná nemocnica s poliklinikou Banská Bystrica  Námestie Ludvika Svobodu 4 Banská Bystrica  974 09  Slovakia |
| Ivona Bacmanakova  Klinika detí a dorastu, UNM Martin Kollarova, 2  Martin, Žilinský kraj, 03659 Slovakia | Local IRB/IEC - Institution Details:  Eticka komisia UNM Univerzitna nemocnica Martin  Kolllarova 2 Martin 03659  Slovakia |
| Rosa Rodriguez  HOSP. GRAL. UNIV. GREGORIO MARANON  Calle O´Donnell 50  Madrid, Spain, 28009 Spain | Central IRB/IEC - Institution Details:  CEI de los Hospitales Universitarios Virgen Macarena-Virgen del Rocío de Sevilla Avda. Manuel Siurot, s/n.  Edificio de Laboratorios - Planta 6ª SEVILLA (C.P 41013)  Spain |
| Ana Cordon  HOSP. REGIONAL UNIV. DE MALAGA  Avda Arroyo De Los Ángeles s/n Malaga, 29011  Spain | Central IRB/IEC - Institution Details:  CEI de los Hospitales Universitarios Virgen Macarena-Virgen del Rocío de Sevilla Avda. Manuel Siurot, s/n.  Edificio de Laboratorios - Planta 6ª SEVILLA (C.P 41013)  Spain |
| Francisco Gimenez-sanchez  HOSP. MEDITERRANEO DE ALMERIA  C/ Nueva Musa s/n Almeria, Almeria, 4007 Spain | Central IRB/IEC - Institution Details:  CEI de los Hospitales Universitarios Virgen Macarena-Virgen del Rocío de Sevilla Avda. Manuel Siurot, s/n.  Edificio de Laboratorios - Planta 6ª SEVILLA (C.P 41013)  Spain |
| Federico Martinon Torres  HOSP. CLINICO UNIV. DE SANTIAGO  c/ A Choupana s.n.  Santiago de Compostela, A Coruña, 15706 Spain | Central IRB/IEC - Institution Details:  CEI de los Hospitales Universitarios Virgen Macarena-Virgen del Rocío de Sevilla Avda. Manuel Siurot, s/n.  Edificio de Laboratorios - Planta 6ª SEVILLA (C.P 41013)  Spain |
| Ignacio Salamanca  INSTITUTO HISPALENSE DE PEDIATRIA  C/ Jardin de la isla, nº 6. Edificio Expolocal Sevilla, Sevilla, 41014  Spain | Central IRB/IEC - Institution Details:  CEI de los Hospitales Universitarios Virgen Macarena-Virgen del Rocío de Sevilla Avda. Manuel Siurot, s/n.  Edificio de Laboratorios - Planta 6ª SEVILLA (C.P 41013)  Spain |
| Jesus Lucas Garcia  HOSP. GRAL. UNIV. DE CASTELLON  Planta 3 E, UCIPN Avda Benicassim s/n Castellon, Castellon  Spain | Central IRB/IEC - Institution Details: CEI de los Hospitales Universitarios Virgen Macarena-Virgen del Rocío de Sevilla Avda. Manuel Siurot, s/n.  Edificio de Laboratorios - Planta 6ª SEVILLA (C.P 41013)  Spain |
| Pablo Rojo Conejo  HOSP. UNIV. 12 DE OCTUBRE  Avenida de Córdoba s/n Madrid, Madrid, 28041 Spain | Central IRB/IEC - Institution Details:  CEI de los Hospitales Universitarios Virgen Macarena-Virgen del Rocío de Sevilla Avda. Manuel Siurot, s/n.  Edificio de Laboratorios - Planta 6ª SEVILLA (C.P 41013)  Spain |
| Cheng-hsun Chiu  Chang Gung Memorial Hospital- Linkou  5, Fusing St., Guishan Dist. Dep. Pediatrics, Taoyuan City, 33305  Taiwan, Province Of China | Central IRB/IEC - Institution Details: Chang Gung Medical Foundation Institutional Review Board  199, Tung Hwa North Road, Taipei  10507  Taiwan |
| Manika Suryadevara  SUNY Upstate Medical University  750 East Adams Street Dept of Pediatrics Syracuse, NY, 13210  United States of America | Local IRB/IEC - Institution Details:  SUNY Upstate Medical University Institutional Review Board for the Protection of Human Subjects  750 East Adams Street Syracuse  NY 13210  United States of America |
| Andrew Wiznia  Jacobi Medical Center  1400 Pelham Parkway South Bldg 1, Rm 1W5 Bronx, NY, 10461  United States Of America | Local IRB/IEC - Institution Details:  BRANY IRB  1981 Marcus Avenue, Suite 210 Lake Success  NY 11042  United States of America |
| Ellen Wald  American Family Children's Hospital1675 Highland Avenue  Madison, WI, 53792 United States Of America | Local IRB/IEC - Institution Details: University of Wisconsin IRB-WIRB 1675 Highland Avenue  Madison WI, 53792  United States of America |
| Kathryn Moffett  West Virginia University 9214-HSC  Morgantown, WV, 26506 United States Of America | Local IRB/IEC - Institution Details: WVU Office of Research Integrity & Compliance  886 Chesnut Ridge Road Morgantown  WV 26506  United States of America |
| Audra Deveikis  Memorial Care Research Miller Children's and Women’s Hospital Long Beach  2801 Atlantic Avenue Long Beach, CA, 90806 United States Of America | Central IRB/IEC - Institution Details: Western Institutional Review Board 1019 39th Avenue SE Suite 120 Puyallup  WA  98374-2115  United States of America |
| Barbara Pahud  The Children's Mercy Hospital  2401 Gillham Road Infectious Diseases Kansas City, MO, 64108  United States Of America | Local IRB/IEC - Institution Details:  The Children’s Mercy Hospital Pediatric Institutional Review Board  2401 Gilham Road Kansas City  MO 64108  United States of America |
